# Investigating for bias in healthcare algorithms: a sex-stratified analysis of supervised machine learning models in liver disease prediction

**DOI:** 10.1136/bmjhci-2021-100457

**Published:** 2022-04-24

**Authors:** Isabel Straw, Honghan Wu

**Affiliations:** Institute of Health Informatics, University College London, London, UK

**Keywords:** Artificial intelligence, BMJ Health Informatics, Health Equity, Machine Learning, Public health informatics

## Abstract

**Objectives:**

The Indian Liver Patient Dataset (ILPD) is used extensively to create algorithms that predict liver disease. Given the existing research describing demographic inequities in liver disease diagnosis and management, these algorithms require scrutiny for potential biases. We address this overlooked issue by investigating ILPD models for sex bias.

**Methods:**

Following our literature review of ILPD papers, the models reported in existing studies are recreated and then interrogated for bias. We define four experiments, training on sex-unbalanced/balanced data, with and without feature selection. We build random forests (RFs), support vector machines (SVMs), Gaussian Naïve Bayes and logistic regression (LR) classifiers, running experiments 100 times, reporting average results with SD.

**Results:**

We reproduce published models achieving accuracies of >70% (LR 71.31% (2.37 SD) – SVM 79.40% (2.50 SD)) and demonstrate a previously unobserved performance disparity. Across all classifiers females suffer from a higher false negative rate (FNR). Presently, RF and LR classifiers are reported as the most effective models, yet in our experiments they demonstrate the greatest FNR disparity (RF; −21.02%; LR; −24.07%).

**Discussion:**

We demonstrate a sex disparity that exists in published ILPD classifiers. In practice, the higher FNR for females would manifest as increased rates of missed diagnosis for female patients and a consequent lack of appropriate care. Our study demonstrates that evaluating biases in the initial stages of machine learning can provide insights into inequalities in current clinical practice, reveal pathophysiological differences between the male and females, and can mitigate the digitisation of inequalities into algorithmic systems.

**Conclusion:**

Our findings are important to medical data scientists, clinicians and policy-makers involved in the implementation medical artificial intelligence systems. An awareness of the potential biases of these systems is essential in preventing the digital exacerbation of healthcare inequalities.

**Summary**

**What is already known on this topic**

- Machine learning models that leverage biochemical data for modelling patient trajectories are rapidly increasing, yet these algorithms are rarely scrutinised for demographic bias or their impact on health inequalities.

**What this study adds**

- Our study demonstrates a previously unobserved sex disparity in model performance for algorithms built from a commonly used liver disease dataset. We highlight how biochemical algorithms may reinforce and exacerbate existing healthcare inequalities.

**How this study might affect research, practice or policy**

- Bias in biochemical algorithms is an overlooked issue. In clinical practice, the higher rate of false negatives for female patients would manifest as an increased rate of missed diagnosis for female patients and a consequent lack of appropriate care.
- Furthermore, sex differences in biochemical feature importance reinforces existing research that suggests unisex biochemical thresholds may disadvantage female patients in current practice. These findings are important to medical data scientists, clinicians and policy-makers involved in the implementation medical artificial intelligence systems. An awareness of the potential biases of these systems is essential in preventing the digital exacerbation of healthcare inequalities

## Background

Liver cirrhosis accounts for 1.8% of deaths in Europe, a number which has grown significantly over the past decade as rates of alcohol consumption, chronic hepatitis infections and obesity-related liver disease have increased.^1^ Yet, liver disease does not affect all populations equally. Recent research has demonstrated sex differences in the prevalence, diagnosis and management of various hepatic illnesses.^2–5^ A key determinant of patient outcomes from liver disease is the early detection of pathology, yet when it comes to diagnosis and referral, female patients appear to be at a significant disadvantage.^2–5^

In alcohol related liver disease, Vatsalya *et al* report that women are less likely to be suspected of alcohol abuse, diagnosed and often experience more severe disease with worse outcomes.^2 3^ Sex differences in diagnosis are compounded by inequalities in the liver disease management. Mathur *et al* report disparities in access to liver transplantation that result in females having markedly lower transplant rates than their male counterparts.^4^ The problem extends beyond hepatology. In 2021, the UK parliamentary report on the gender health gap highlighted that the UK has the largest female health gap in the G20 and the 12th largest globally.^5^ The exclusion of females from research trials (extending to animal research), the neglect of female bodies throughout medical pedagogy and the unconscious biases of practitioners are a few of the intersecting factors that result in worse health outcomes for female patients.^6–10^

Liver function tests are integral to patient diagnosis and monitoring. These ‘biochemical markers’ include proteins made by the liver (eg, albumin), and enzymes required for metabolism (eg, aspartate aminotransferase (AST)). Bias research has illustrated that biochemical markers are not equally effective for all patient groups.^3 7 10–12^ Suthahar *et al* describe how sex differences in biomarker thresholds affect objectivity in management, as what is considered ‘normal’ in one sex, may not be so in the other.^12^ Grimm *et al* investigate the relationship between albumin and mortality, reporting that albumin offers a higher predictive power for males compared with females.^11^ Furthermore, Vatsalya *et al* and Stepien *et al* describe sex differences in biochemical cut offs, highlighting that the milder expression of liver injury for females may result in female disease going undetected.^3 13^ Such disparities in the predictive potential of clinical biomarkers have the potential to exacerbate healthcare inequalities.^6 7 10 12^

The rise in healthcare artificial intelligence (AI) has resulted in the increasing use of large clinical datasets for machine learning (ML).^14^ ML classifiers that use biochemical markers to model patient trajectories have consistently outperformed traditional statistical models.^14^ However, despite the promise of ML tools, the presence of demographic biases in AI algorithms has indicated that historical harms may materialise in digital systems and worsen population inequalities.^7 15–17^ The development of predictive models from biomarkers is one area in which medical ML models are at risk of encoding the errors of current practice. In our paper we explore for this possibility in liver disease prediction by examining models built from a commonly cited dataset: The Indian Liver Patient Dataset (ILPD).

The ILPD is a widely used open-source dataset that provides the biochemical markers of a sample of patients, some of whom have liver disease.^18–22^ BanuPriya and Tamilselvi provide an overview of classification models built from this dataset, since which time further models have been published from both academics and major industry.^18 19 21^ Authors consistently report accuracies of >70% for identifying liver patients, with logistic regression (LR) models and random forests (RFs) giving the best results. Jin *et al*^23^ demonstrate accuracies of 72.7% with LR models, similarly Adil *et al* achieve 74% accuracy with their LR model, outperforming artificial neural networks and support vector machines (SVMs).^24^ A recent study from Intel reproduces these models and performs additional feature selection giving model accuracies of 74.6% (RF) and 71.2% (SVM).^19^

Predictive ML models may benefit patient care if they can diagnose liver disease at an earlier stage.^25^ Yet, despite the existing literature that describes biases in clinical medicine, biochemical tests and algorithmic performance, none of the ML studies on the ILPD focus on sex disparities in model performance.^4 7 8 10–12 16 17^ We seek to address this gap in the research by investigating the ILPD dataset and its respective models for sex bias.^18–20^

## Methodology

The ILPD was originally collected from India and consists of 583 patient records, of which 416 have liver disease. We imported the ILPD from the UCI repository (full codebook available in online supplemental material C).^19 22^

10.1136/bmjhci-2021-100457.supp1 Supplementary data

### Data exploration and initial analysis

Data exploration is the primary stage of the ML process and involves file importation, formatting, descriptive statistics and configuring datatypes. Online supplemental table 1 gives the variables included in our dataset and their initial datatypes.

10.1136/bmjhci-2021-100457.supp2 Supplementary data

### Feature exploration

Online supplemental table 2 presents the sex-stratified feature importance ranked by Pearson’s correlation coefficient. For females, the enzymes ALT and AST are ranked fourth and fifth, whereas for males they are ranked seventh and eighth. Further, albumin and A/G ratio are ranked higher for male patients compared with female patients. These subtle differences in feature importance may reflect underlying sex differences in hepatic pathophysiology and biomarker expression.^3 4 26^ Further, online supplemental table 2 demonstrates that the mean IQR across all biomarkers is less for females, suggesting that these biomarkers may have less of a predictive power for female patients overall (mean IQR; female 0.145, male 0.175).

### Data preprocessing

Data preparation steps reflected existing studies.^19 20^ Mean imputation was used to address missing values, gender was mapped to a 0/1 numerical datatype, normalisation was performed using minimum-maximum scaler function and the target variable was recoded to binary variable, such that 1 represents diseased patients (n=416).

### Addressing class imbalance

The original dataset demonstrated significant class imbalance (167 healthy vs 416) diseased patients) and sex imbalance (142 females vs 441 males). Similarly to existing models, we implement the imblearn SMOTE() package to address these imbalances; oversampling both the minority class and under-represented females as detailed in table 1.^19^ The sex-unbalanced dataset is retained to compare the impact of female representation in the training data on sex disparities in performance.

**Table 1:** Summary counts of classes in the Indian liver patient dataset dataset, including counts after the dataset is balanced

	Target (disease=1)	Dataset 1 (original)	Total counts for sexes	Dataset 2 (oversampled minority class)	Total counts for sexes	Dataset 3 (sex balanced, oversampled females)	Total counts for sexes
Female	0	50	142	145	237	408	595
	1	92		92		187	
Male	0	117	441	271	595	271	595
	1	324		324		324	
Total			583		832		1190

### Model development and implementation

Gulia and Praveen Rani review the classification algorithms that have been built from the ILPD, including RFs and SVMs.^20^ A more recent review from BanuPriya and Tamilselvi describe the accuracies of additional models including Bayesian Networks, which is further built on by the work of Aswathy who evaluates the performance of LR models on the ILPD.^18 19^ We replicate the methods of these studies, reproducing RF, SVM, Gaussian Naïve Bayes (GNB) and LR classifiers. We implement these models across four experiments, in which we evaluate the overall and sex-stratified performance of the classifiers.

#### Experiment 1: models trained on unbalanced dataset, without feature selection

Initially, we reproduce existing studies, building a predictive algorithm on the full unbalanced dataset to predict liver disease. Data were divided into test and training subsets (30%/70%), hyperparameters were tuned using GridSearchCV(), the model was trained on the mixed-sex data and results were stratified by sex to give the evaluation metrics for males/females separately. We do this 100 times (building, training and testing separate models) and report average results with SD over the 100 runs. This is done for all four classifiers resulting in four results tables (online supplemental material B Spreadsheets, ‘Experiment 3.1.1—RF’—‘Experiment 3.1.1 GNB’).

10.1136/bmjhci-2021-100457.supp3 Supplementary data

#### Experiment 2: models trained on sex-balanced dataset, without feature selection

The methodology of experiment 1 is repeated using the sex-balanced dataset defined in Table 1. We ensure sex balance in the training data by taking random subsets from the male and females separately, which are appended together to form the full sex-balanced training data for each individual experiment (online supplemental file 3 Spreadsheets, ‘Experiment 3.1.2—RF’—‘Experiment 3.1.2 GNB’).

#### Experiment 3: models trained on unbalanced dataset, with feature selection

In experiment 3, we perform feature selection based on the unbalanced dataset, in experiment 4, we perform feature selection on the sex-balanced dataset. Feature selection is performed using Recursive Feature Elimination (RFE) sklearn package, which returns the top five ranked features (online supplemental material B Spreadsheets, ‘Experiment 3.1.3—RF’—‘Experiment 3.1.3 GNB’).

#### Experiment 4: models trained on balanced dataset, with feature selection

Lastly, models and feature selection are fitted to the sex-balanced dataset. Our aim was to investigate whether feature selection would differ once the representation of females was addressed, and whether this would influence any performance disparities.

### Model evaluation

Evaluation metrics are reported for all patients and separately for the sexes (equations 1–3). We examine the mean difference between the male and females for each evaluation metric to demonstrate any disparities (equation 4). Two-sample paired t-tests are run on the series of 100 experiments for the male and female patients to assess whether the mean difference between sexes, for each of the evaluation metrics, is statistically significant (p<0.05).

#### Equation 1: accuracy evaluation metric

Accuracy gives the proportion of correct predictions produced by a model.

$$Accuracy=\frac{Truepositives+TrueNegatives}{Truepositives+TrueNegatives+FalsePositives+FalseNegatives}$$

#### Equation 2: F-score evaluation metric, precision and recall

The F-score is the average of precision and recall, with a value of 1 being a perfect score.

$$Recall=\frac{TP}{TP+FN}$$

$$FScore=\frac{2\times Precision\times Recall}{Precision+Recall}$$

#### Equation 3: performance error rates

The following error rates are used throughout our evaluation.^21^

- True positive: Predicted yes and they do have disease.
- True negative: Predicted no and they do not have disease.
- False positive: Predicted yes, but they do not have disease.
- False negative: Predicted no, but they actually do have disease.

$$TrueNegativeRate\left(TNR\right)=\frac{TN}{TN+FP}$$

$$TruePositiveRate\left(TPR\right)=\frac{TP}{TP+FN}$$

#### Equation 4: sex performance disparity

Sexperformancedisparity=Maleevaluationmetric(mean)−Femaleevaluationmetric(mean)

## Results

We ran 16 experiments: experiments 1–4, with each of the four classifiers. The detailed results tables with the 100 experiment runs are provided in the spreadsheet files in online supplemental material B. In online supplemental material A ‘Tables in Text’, we provide summary in several condensed tables, which give the average evaluation metrics and the statistical significance of any male-female differences.

### Results for experiment 1

Online supplemental table 3 demonstrates that our four models reflect the existing literature, achieving accuracies >70% (71.31% (2.37 SD) LR – 79.40% (2.50 SD) SVM). Table 2 details the disparities for each evaluation metric, from which we observe a statistically significant sex disparity in Accuracy for all classifiers, with mixed results regarding the direction of the disparity (performance disparity −2.98 SVM to 2.96% RF, p<0.05). In the case of the ROC_AUC score, we observe a significant disparity that negatively impacts females for the RF (6.80%, p<0.05), LR (2.93%, p<0.05) and GNB (5.53%, p<0.05) classifiers.

**Table 2:** Experiment 3.1.1—unbalanced training data without feature selection, sex performance disparities

Mean difference averaged over n=100	Random forest classifier		Logistic regression classifier		Support vector machine		Gaussian Naïve Bayes	
	Sex performance disparities (%)	t-test p value	Sex performance disparities (%)	t-test p value	Sex performance disparities (%)	t-test p value	Sex performance disparities (%)	t-test p Value
Accuracy	2.96	0.00	−2.85	0.01	−2.98	0.02	−2.72	0.02
FScore	15.63	0.00	15.86	0.00	4.14	0.00	16.19	0.00
ROC_AUC*	6.80	0.00	2.93	0.00	−2.41	0.08	5.53	0.00
Precision	5.25	0.00	−4.87	0.00	3.41	0.00	−3.13	0.05
Recall	21.02	0.00	24.07	0.00	2.58	0.04	19.31	0.00
False negative rate	−21.02	0.00	−24.07	0.00	−2.58	0.08	−19.31	0.00
True negative rate	−7.42	0.00	−18.20	0.00	−7.40	0.00	−8.24	0.00
False positive rate	7.42	0.00	18.20	0.00	7.40	0.00	8.24	0.00
True positive rate	21.02	0.00	24.07	0.00	2.58	0.04	19.31	0.00

*ROC AUC score is a measure of the separation between classes in a binary classifier, derived from the area under the ROC curve.

The accuracy and ROC_AUC disparities fluctuate depending on the balance between the different error rates, however, on examining the error rates individually, we see a consistency in error trends for each sex. Across all classifiers females suffer from a higher false negative rate (FNR), while males suffer from a higher false positive rate. The disparity demonstrates a consistently higher recall for males, with females experience a lower recall and correspondingly higher FNR disparity, −2.58% to −24.07%, table 2)

### Results for experiment 2

In experiment 2, we trained on sex-balanced data, improving overall accuracy across all four classifiers (RF 81.66% (2.33 SD) vs 78.17 (2.36 SD); LR 74.53% (1.96 SD) vs 71.31% (2.37 SD); SVM 83.30% (1.75 SD) vs 79.40% (2.50 SD); GNB 74.75% (1.9 SD) vs 71.53% (2.61 SD)—online supplemental table 4). We now see a consistent accuracy disparity that benefits females across all four classifiers (−11.47% to −6.17%, p<0.05−table 3). Disparities in the ROC_AUC scores are less consistent (LR unbalanced ROC disparity 2.93%, LR balanced ROC disparity 4.79%; GNB unbalanced ROC disparity 5.53%, GNB balanced disparity 5.45%).

**Table 3:** Experiment 3.1.2—balanced training data without feature selection, sex performance disparities

Mean difference averaged over n=100	Random forest classifier		Logistic regression classifier		Support vector machine		Gaussian Naïve Bayes	
	Sex performance disparities (%)	t-test p value	Sex performance disparities (%)	t-test p value	Sex performance disparities (%)	t-test p value	Sex performance disparities (%)	t-test p value
Accuracy	−6.17	0.00	−6.36	0.00	−11.47	0.00	−7.43	0.00
FScore	7.69	0.00	20.17	0.00	−3.40	0.00	16.65	0.00
ROC_AUC	0.60	0.13	4.79	0.00	−9.06	0.00	5.45	0.00
Precision	−0.94	0.88	−4.75	0.00	−2.32	0.14	0.24	0.37
Recall	12.88	0.00	29.22	0.00	−4.64	0.00	19.82	0.00
False negative rate	−12.88	0.00	−29.22	0.00	4.64	0.00	−19.82	0.00
True negative rate	−11.69	0.00	−19.65	0.00	−13.49	0.00	−8.93	0.00
False positive rate	11.69	0.00	19.65	0.00	13.49	0.00	8.93	0.00
True positive rate	12.88	0.00	29.22	0.00	−4.64	0.00	19.82	0.00

Online supplemental table 5 presents a comparison of the evaluation metrics with/without balancing of the training data. In one case, we observe an improvement in performance for all patients. When trained on the balanced dataset, the LR accuracy improves overall (74.53% (1.96 SD) vs 71.31% (2.37 SD)), for females (77.71% (2.42 SD) vs 73.33% (3.95 SD)) and for males (71.35% (3.22 SD) vs 70.49% (2.74 SD)).

### Results for experiment 3

We did not see an improvement in overall performance or a reduction in disparities with RFE. A significant ROC_AUC disparity is apparent across all four classifiers (3.60%–6.61%, p<0.05) that negatively impacts females. We see the same error rate findings as earlier, with a higher FNR for females (FNR Disparity −18.21 to −21.24%, p<0.05, table 4 and online supplemental table 6).

**Table 4:** Experiment 3.1.3—unbalanced training data with feature selection, sex performance disparities

	Random forest classifier		Logistic regression classifier		Support vector machine		Gaussian Naïve Bayes	
	Sex performance disparities (%)	t-test p value	Sex performance disparities (%)	t-test p value	Sex performance disparities (%)	t-test p value	Sex performance disparities (%)	t-test p value
Accuracy	3.42	0.00	−2.90	0.01	−2.75	0.01	−3.31	0.00
FScore	15.36	0.00	15.79	0.00	16.50	0.00	15.29	0.00
ROC_AUC	6.61	0.00	3.60	0.00	4.90	0.00	4.99	0.00
Precision	9.85	0.00	0.24	0.44	−0.87	0.90	−3.41	0.03
Recall	18.21	0.00	21.24	0.00	20.30	0.00	18.54	0.00
False negative rate	−18.21	0.00	−21.24	0.00	−20.30	0.00	−18.54	0.00
True negative rate	−4.99	0.00	−14.04	0.00	−10.50	0.00	−8.57	0.00
False positive rate	4.99	0.00	14.04	0.00	10.50	0.00	8.57	0.00
True positive rate	18.21	0.00	21.24	0.00	20.30	0.00	18.54	0.00

### Results for experiment 4

Experiment 4 gives mixed results. The accuracy disparity benefits females across all classifiers (−4.64% to −6.80%, p<0.05), whereas the ROC_AUC disparity demonstrates a benefit for males in three out of four classifiers (−0.05% to 5.95%, p<0.05, table 5) The results relate to the subtle changes in error rates with each model, however, across all classifiers the FNR is consistently higher for females (−9.70% to −22.78%, p<0.05 (online supplemental table 7).

**Table 5:** Experiment 3.1.4—balanced training data with feature selection, sex performance disparities

	Random forest classifier		Logistic regression classifier		Support vector machine		Gaussian Naïve Bayes	
	Sex performance disparities (%)	t-test p value	Sex performance disparities (%)	t-test p value	Sex performance disparities (%)	t-test p value	Sex performance disparities (%)	t-test p value
Accuracy	−5.62	0.00	−6.80	0.00	−6.19	0.00	−4.64	0.00
FScore	7.86	0.00	14.39	0.00	16.46	0.00	21.63	0.00
ROC_AUC	−0.05%	0.46	3.57%	0.00	5.95%	0.00	8.17%	0.00
Precision	4.60%	0.00	9.28%	0.00	12.82%	0.00	9.35%	0.00
Recall	9.70%	0.00	15.51%	0.00	15.38%	0.00	22.78%	0.00
False negative rate	−9.70	0.00	−15.51	0.00	−15.38	0.00	−22.78	0.00
True negative rate	−9.79	0.00	−8.37	0.00	−3.47	0.00	−6.44	0.00
False positive rate	9.79	0.00	8.37	0.00	3.47	0.00	6.44	0.00
True positive rate	9.70	0.00	15.51	0.00	15.38	0.00	22.78	0.00

### Analysis of feature selection

Online supplemental table 8 gives the feature rankings assigned by the RFE model when fitted to unbalanced and balanced data, focusing on RF classifiers. When we address the under-representation of females in the training data, ALP and gender are included as the top two features, while A/G ratio and total bilirubin are removed. This finding may reflect existing research that describes sex differences in biomarker expression. In their analysis gender-specific references intervals for hepatic biomarkers, Li *et al* highlight sex differences in ALP, ALT and GGT, indicating that differing thresholds may be appropriate for diagnosis.^27^ Sex differences in biochemical disease profiles may explain why integrating more female patients affects the feature selection in experiment 4.

## Discussion

In recent years, research has highlighted that medical biases and female under-representation may significantly contribute to differences in healthcare outcomes; in our paper, we have examined how this phenomena may extend into ML.^6–8 10 28^ We present several key findings:

- Model reproduction and demonstration of disparity: We have demonstrated a previously unobserved sex disparity that exists in published ML classifiers based on the ILPD dataset.
- Error disparities: Sex disparities in Accuracy and ROC_AUC fluctuate depending on model and the balance between error rates, however, sex differences in specific error rates are consistent. We observe a consistently lower recall and correspondingly higher FNR for females. Of note, RF and LR classifiers are reported as the most effective on the ILPD dataset, however, these models demonstrate the greatest disparity in the FNR when trained on the original dataset (RF, FNR disparity −21.02% (p<0.05); LR, FNR disparity −24.07%, (p<0.05)). Clinically, this FNR disparity would materialise as an inequality in disease detection that negatively impacts females, with higher instances of missed disease.
- Balanced training: Training on sex-balanced data improved overall performance for all classifiers. In the case of the LR classifier, accuracy improves overall and for the sexes separately, indicating that with the right model selection addressing poor performance for the under-represented group does not need to come at the expense of the majority group.
- Impact of model architecture on disparity: Our experimental outcomes were not consistent across models, indicating that bias mitigation techniques may need to be tailored to model choice.
- Analysis of feature ranking: Our comparison of feature importance reinforces existing clinical research that highlights the sex differences in the role of liver biomarkers.

### Implications for data science

Our experiments demonstrated that sex-specific feature selection and addressing under-representation of females may be an important bias mitigation technique when developing ML algorithms in medicine. Furthermore, we illustrate that there is no consistent solution across all classifiers, suggesting techniques need to be tailored to model choice. ML models also present novel opportunities for improving existing practice and addressing health disparities that relate to biochemical discrepancies between the sexes. Given the evolving evidence that critiques the use of ‘unisex’ biochemical thresholds, ML models that do not rely on these defined thresholds may pose a superior alternative if developed with an awareness of the subtle sex differences in disease manifestation.

### Implications for clinical medicine and public health

Classification algorithms are being increasingly used in healthcare settings to assist clinicians in medical diagnosis.^20^ Unless these algorithms are evaluated for biases, they may only improve care for a subset of patients and consequently increase healthcare inequalities.^7^ By evaluating ML models for demographic biases before they are implemented in digital medicine, we can mitigate the perpetuation of these inequalities into digital systems.

Furthermore, insights from model development can be used to inform current clinical care. Our data exploration of feature correlation demonstrated sex differences in feature importance. Such research can inform practising clinicians on the relevance of different indicators for the patient in front of them, for example, albumin may be more indicative of pathology in males.^11^ Lastly, examining disparities in algorithmic performance offers an opportunity to reflect on which patients may be being missed in current practice. Throughout our analysis, we demonstrated a persistently high FNR for females, suggesting that female disease is at risk of being overlooked. Examining the physiological profile of algorithmic false negatives presents an opportunity to better understand which patients are at risk of being misdiagnosed.

It should be noted that the ILPD does not include demographic information on race or ethnicity.^22^ Racial biases have been reported in the biochemical tests used across different subspecialties, resulting in worse care for marginalised racial groups.^29 30^ A key limitation of our study is that we cannot perform a race stratified analysis. Furthermore, we are unable to evaluate the relevance of other demographic features. An intersectional approach to healthcare inequalities would consider the mediating impact of socioeconomic class, or the compounding impact of gender (as opposed to sex) and sexuality on marginalised patients. Accounting for the complex nature of these intersectional relationships requires more advanced modelling and new bias evaluation techniques.

## Conclusions

The historic absence of women from the healthcare profession and from clinical research resulted in domain knowledge that centres around the male body and neglects female physiological differences. To ensure sex-based inequalities do not manifest in medical AI, an evaluation of demographic performance disparities must be integrated into model development. Evaluating biases in the initial stages of ML can provide insights into inequalities in existing practice, reveal pathophysiological differences between the sexes and can mitigate the digitisation of healthcare inequalities in algorithmic systems.

## Data Availability

Data are available in a public, open access repository.
