# Supplementary material for: Investigating for bias in healthcare algorithms: a sex-stratified analysis of supervised machine learning models in liver disease prediction
Source: BMJ Health Care Inform. 2022 Apr 24;29(1):e100457. doi: 10.1136/bmjhci-2021-100457 (PMC9039354; doi:10.1136/bmjhci-2021-100457)
Supplement: Supplementary data [file bmjhci-2021-100457supp001.pdf]

Investigating for bias in healthcare algorithms: A sex stratified analysis of supervised machine learning models in liver disease prediction

Dr I Straw, Dr Honghan Wu

Unviersity College London, Institute of Health Informatics

Codebook and manuscript currently under submission for the British Medical Journal (BMJ).

```
!pip install imbalanced-learn

pip install --upgrade scipy

import pandas as pd
import numpy as np
import matplotlib.pyplot as plt
from sklearn.metrics import plot_confusion_matrix
from sklearn.metrics import f1_score
from sklearn.model_selection import GridSearchCV
from sklearn.model_selection import train_test_split
from sklearn.svm import SVC
from sklearn.linear_model import LogisticRegression
from sklearn.svm import SVC, LinearSVC

import imblearn
from imblearn.over_sampling import SMOTE
from tqdm import tqdm
from sklearn.pipeline import Pipeline
from sklearn.decomposition import PCA


from sklearn.model_selection import RepeatedStratifiedKFold
from sklearn.preprocessing import PowerTransformer
from sklearn.feature_selection import RFE
import seaborn as sns
from sklearn.utils import resample
from sklearn.preprocessing import MinMaxScaler
from sklearn import neighbors
from sklearn.metrics import precision_score, recall_score,
classification_report, confusion_matrix, roc_auc_score, auc,
roc_curve, make_scorer
from sklearn.base import clone
from itertools import combinations
from sklearn.model_selection import train_test_split, cross_val_score,
cross_validate
from keras.models import Sequential
from keras.layers import Dense
```

```
from keras.wrappers.scikit_learn import KerasClassifier
from sklearn.model_selection import GridSearchCV
from sklearn.metrics import accuracy_score
from sklearn.model_selection import train_test_split
from sklearn.metrics import roc_auc_score
from sklearn.metrics import classification_report,confusion_matrix
from sklearn import linear_model
from sklearn.ensemble import RandomForestClassifier,
AdaBoostClassifier, BaggingClassifier
from sklearn.neighbors import KNeighborsClassifier
from sklearn.naive_bayes import GaussianNB
from scipy.stats import kendalltau
from scipy.stats import ttest_ind

import warnings
warnings.filterwarnings("ignore")
```

Data Import and Exploration

```
# Importing dataset from URL
URL =
"https://archive.ics.uci.edu/ml/machine-learning-databases/00225/India
n%20Liver%20Patient%20Dataset%20(ILPD).csv"
ILPD = pd.read_csv(URL, names=["Age", "Gender", "Total Bilirubin
(TB)", "Direct Bilirubin (DB)", "ALP (Alkphos Alkaline Phosphotase)",
"SGPT Alamine Aminotransferate", "SGOT Aspartate Aminotransferase",
"Total Protiens (TP)", "Albumin (ALB)", "A/G Ratio", "Target"])
ILPD.head(5)

print("The Shape of the Dataset:", ILPD.shape)
ILPD.describe()
ILPD.info() # gender datatype needs to be changed

ILPD['Target'] = (ILPD['Target']).astype(int)
ILPD.info()
```

Descriptives statistics performed before data pre-processing

```
ILPD.groupby(['Gender', 'Target']).size()

# Descriptive statistics
ILPD.groupby(['Gender']).mean()

# Addressing null values
ILPD['A/G Ratio'].fillna(ILPD['A/G Ratio'].mean(), inplace=True)
ILPD[ILPD["A/G Ratio"].isnull()]

ILPD.isnull().sum()

# Converting Gender to a numerical variable
gender_mapping = {'Male':1, 'Female':0}

# target 2 = healthy, 1 = diseased
```

```
target_mapping = {2:0, 1:1}
ILPD['Gender'] = ILPD['Gender'].map(gender_mapping)
ILPD['Target'] = ILPD['Target'].map(target_mapping)
ILPD.info()
# Need to be an integer, or becomes decimals in the upsampling
ILPD['Target'] = (ILPD['Target']).astype(int)

ILPD.groupby(['Gender', 'Target']).size()


F_ILPD1 = ILPD[ILPD['Gender']==0]
print('Female data:', len(F_ILPD1))
M_ILPD1 = ILPD[ILPD['Gender']==1]
print('Male data:', len(M_ILPD1))

# Correlating features for males and females
ILPDCorrelation = pd.DataFrame(ILPD.corr(method='pearson'))
ILPDCorrelation['Target Correlations'] =
ILPDCorrelation['Target'].abs()
ILPDCorrelation = ILPDCorrelation.iloc[:, -1:]
ILPDCorrelation.sort_values(by='Target Correlations', ascending=False,
inplace=True)
ILPDCorrelation.reset_index(level=0, inplace=True)
ILPDCorrelation.reset_index(level=0, inplace=True)
ILPDCorrelation.rename(columns = {'level_0':'All Rank',
'index':'Feature'}, inplace=True)

F_ILPDCorrelation = pd.DataFrame(F_ILPD1.corr(method='pearson'))
F_ILPDCorrelation['F Target Correlations'] =
F_ILPDCorrelation['Target'].abs()
F_ILPDCorrelation = F_ILPDCorrelation.iloc[:, -1:]
F_ILPDCorrelation.sort_values(by='F Target Correlations',
ascending=False, inplace=True)
F_ILPDCorrelation.reset_index(level=0, inplace=True)
F_ILPDCorrelation.reset_index(level=0, inplace=True)
F_ILPDCorrelation.rename(columns = {'level_0':'Female Rank',
'index':'Feature'}, inplace=True)

M_ILPDCorrelation = pd.DataFrame(M_ILPD1.corr(method='pearson'))
M_ILPDCorrelation['M Target Correlations'] =
M_ILPDCorrelation['Target'].abs()
M_ILPDCorrelation = M_ILPDCorrelation.iloc[:, -1:]
M_ILPDCorrelation.sort_values(by='M Target Correlations',
ascending=False, inplace=True)
M_ILPDCorrelation.reset_index(level=0, inplace=True)
M_ILPDCorrelation.reset_index(level=0, inplace=True)
M_ILPDCorrelation.rename(columns = {'level_0':'Male Rank',
'index':'Feature'}, inplace=True)
ILPDCorrelation
```

```
FinalTable1 = pd.merge(ILPDCorrelation, F_ILPDCorrelation,
on='Feature', how='left')
FinalTable = pd.merge(FinalTable1, M_ILPDCorrelation, on='Feature',
how='left')
FinalTable = FinalTable.iloc[1:, :]
FRanks = FinalTable['Female Rank']
MRanks = FinalTable['Male Rank']
AllRanks = FinalTable['All Rank']
print(AllRanks)
print(FRanks)
print(MRanks)
FinalTable
Mcorr, _ = kendalltau(MRanks, AllRanks)
Fcorr, _ = kendalltau(FRanks, AllRanks)
print(Mcorr, Fcorr)
```

Data Preperation

```
# Addressing Skewed Data
skewed = ['Total Bilirubin (TB)', 'Direct Bilirubin (DB)', 'ALP
(Alkphos Alkaline Phosphotase)', 'SGPT Alamine Aminotransferate',
'SGOT Aspartate Aminotransferase', 'Total Protiens (TP)', 'A/G Ratio']
skewed
for column in skewed:
  ILPD[column] = ILPD[column].apply('log1p')

# Addressing null values
ILPD['A/G Ratio'].fillna(ILPD['A/G Ratio'].mean(), inplace=True)
ILPD[ILPD["A/G Ratio"].isnull()]

ILPD.isnull().sum()

Scaler = MinMaxScaler()
ScaledILPD = ILPD
ScaledILPD[['Total Bilirubin (TB)', 'Direct Bilirubin (DB)',
       'ALP (Alkphos Alkaline Phosphotase)', 'SGPT Alamine
Aminotransferate',
       'SGOT Aspartate Aminotransferase', 'Total Protiens (TP)',
       'Albumin (ALB)', 'A/G Ratio',
'Target']]=Scaler.fit_transform(ILPD[['Total Bilirubin (TB)', 'Direct
Bilirubin (DB)',
       'ALP (Alkphos Alkaline Phosphotase)', 'SGPT Alamine
Aminotransferate',
       'SGOT Aspartate Aminotransferase', 'Total Protiens (TP)',
       'Albumin (ALB)', 'A/G Ratio', 'Target']])
```

Defining Datasets

A described in the accompanying thesis, we seperate the Indian Liver Patient Dataset (ILPD) into sex-stratified datasets to perform our model evaluation.

```
print('Female data:', len(F_ILPD1))
print('Male data:', len(M_ILPD1))

# Oversampling to address class imbalance
oversample = SMOTE()

y_ILPDTarget = ILPD.iloc[:, -1]
X_AllILPD = ILPD.iloc[:, :-1]
All_ILPDX_over, All_ILPDy_over = oversample.fit_resample(X_AllILPD,
y_ILPDTarget)

# Reform this into a dataframe
ILPD_ClassBal = All_ILPDX_over
ILPD_ClassBal['Target'] = All_ILPDy_over
ILPD_ClassBal

# Print after class imbalance address

print("Before addressing class imbalance")
print(ILPD.groupby(['Target']).size())

print("After addressing class imbalance")
print(ILPD_ClassBal.groupby(['Target']).size())

ILPD = ILPD_ClassBal

ILPD_ClassBal.groupby(['Gender', 'Target']).size()

ILPD['Target'] = ILPD['Target'].astype(int)
ILPD.info()

oversample = SMOTE()

y_ILPDSexTarget = ILPD.iloc[:, 1]
X_ILPD = ILPD.loc[:, ['Age', 'Total Bilirubin (TB)', 'Direct Bilirubin
(DB)',
       'ALP (Alkphos Alkaline Phosphotase)', 'SGPT Alamine
Aminotransferate',
       'SGOT Aspartate Aminotransferase', 'Total Protiens (TP)',
       'Albumin (ALB)', 'A/G Ratio', 'Target']]

ILPDX_over, ILPDy_over = oversample.fit_resample(X_ILPD,
y_ILPDSexTarget)
ILPD_Final = ILPDX_over
ILPD_Final['Gender'] = ILPDy_over
ILPD_Final
F_ILPD2 = ILPD_Final[ILPD_Final['Gender']==0]
print('Female data:', len(F_ILPD2))
M_ILPD2 = ILPD_Final[ILPD_Final['Gender']==1] # With 1 as suffix as
will reasmple for balancing
```

```
print('Female data:', len(F_ILPD2))
print('Male data:', len(M_ILPD2))

# These results may differ slightly to the manuscript, as the exact
counts will differ depending on the SMOTE Sampling
F_Sick1 = F_ILPD2[F_ILPD2['Target']==1]
F_Well1 = F_ILPD2[F_ILPD2['Target']==0]
M_Sick1 = M_ILPD2[M_ILPD2['Target']==1]
M_Well1 = M_ILPD2[M_ILPD2['Target']==0]

print('Sick Females:', len(F_Sick1), 'Sick Males:', len(M_Sick1))
print('Well Females:', len(F_Well1), 'Well Males', len(M_Well1))
```

3.0 Defining Datasets: Balanced and Unbalanced

```
AllX = ILPD.iloc[:, :-1]
Ally = ILPD.iloc[:, -1]

print(M_ILPD2.groupby(['Gender', 'Target']).size())
print(F_ILPD2.groupby(['Gender', 'Target']).size())
F_ILPD2

# For the balanced experiments we need Fx Fy seperate
FX = F_ILPD2.loc[:, ['Age', 'Total Bilirubin (TB)', 'Direct Bilirubin
(DB)',
       'ALP (Alkphos Alkaline Phosphotase)', 'SGPT Alamine
Aminotransferate',
       'SGOT Aspartate Aminotransferase', 'Total Protiens (TP)',
       'Albumin (ALB)', 'A/G Ratio', 'Gender']]
Fy = F_ILPD2.loc[:, ['Target']]

MX = M_ILPD2.loc[:, ['Age', 'Total Bilirubin (TB)', 'Direct Bilirubin
(DB)',
       'ALP (Alkphos Alkaline Phosphotase)', 'SGPT Alamine
Aminotransferate',
       'SGOT Aspartate Aminotransferase', 'Total Protiens (TP)',
       'Albumin (ALB)', 'A/G Ratio', 'Gender']]
My = M_ILPD2.loc[:, ['Target']]
print(len(FX), len(MX), len(Fy), len(My))

Bal_ILPD = M_ILPD2.append(F_ILPD2)

## Define these so can fit to balanced data for hyperparameter tuning
Bal_X = Bal_ILPD.iloc[:, :-1]
Bal_y = Bal_ILPD.iloc[:, -1]

Bal_ILPD.groupby(['Gender', 'Target']).size()
print(len(FX), len(Fy), len(MX), len(My))

Bal_ILPD.groupby(['Gender', 'Target']).size()
```

Model Development and Hyperparameter Tuning

(Deleted previous way of tuning, see downloadeds in file - now done in function)

```
# For reference we now have our Unbalanced Data and Balanced Data
print(Bal_ILPD.shape, len(Bal_X), len(Bal_y))
print(ILPD.shape, len(AllX), len(Ally))

print(AllX.shape, Ally.shape)
print(AllX.groupby(['Gender']).size())
```

3.0 Experiments

Model Experiments: Defining functions for model expeirments

3.1.1: Experiment 1 - Unbalanced without feature selection (4 models) 3.1.2: Experiment 2 - Balanced without feature selection (4 models) 3.1.3: Experiment 3 - Unbalanced **with** feature selection (4 models) 3.1.4: Experiment 4 - Balanced **with** feature seleciton (4 models)

```
# Defined Feature Set
## Defining feature subsets
AllFeatures = ['Age', 'Gender', 'Total Bilirubin (TB)', 'Direct
Bilirubin (DB)',
       'ALP (Alkphos Alkaline Phosphotase)', 'SGPT Alamine
Aminotransferate',
       'SGOT Aspartate Aminotransferase', 'Total Protiens (TP)',
       'Albumin (ALB)', 'A/G Ratio']

NB = GaussianNB()
LR = LogisticRegression()
SVM = SVC()
RF = RandomForestClassifier()
```

3.1 Unbalanced Training Data, No Feature Selection

```
# 1.
def unbalanced_run(X, y, FeatureSet, Classifier):
    AllX_train, AllX_test, Ally_train, Ally_test = train_test_split(X,
y, test_size=0.30)
    X_train = AllX_train[FeatureSet]
    X_test = AllX_test[FeatureSet]

    # Experiment specific hyperparameter tuning - Needed for
parameters
    depth=[2, 8, 16]
    n_estimators = [64, 128, 256]
    RFParams = dict(max_depth=depth, n_estimators=n_estimators)
    LRParams = {"C":np.logspace(-3,3,7), "penalty":["l1","l2"]}
```

```
    SVMParams = {'C': [0.001,0.01,0.1,1,10,100,1000],
                 'gamma': [0.001,0.01,0.1,1,10,100,1000]
                }
    NBParams = {'var_smoothing': np.logspace(0,-9, num=100)}

    # Name of Parameters
    if Classifier == LR:
        params = LRParams
    if Classifier == RF:
        params = RFParams
    if Classifier == SVM:
        params = SVMParams
    if Classifier == NB:
        params = NBParams

    ClassifierParams = GridSearchCV(Classifier, params, cv=5,
scoring='accuracy')
    ClassifierParams.fit(AllX_train, Ally_train)

    if Classifier == LR:
        Model1 = LogisticRegression(**ClassifierParams.best_params_)
    if Classifier == SVM:
        Model1 = SVC(**ClassifierParams.best_params_)
    if Classifier == RF:
        Model1 =
RandomForestClassifier(**ClassifierParams.best_params_)
    if Classifier == NB:
        Model1 = GaussianNB(**ClassifierParams.best_params_)

    Model = Model1.fit(AllX_train, Ally_train)
    Ally_pred = Model.predict(AllX_test)

    # Printing out results - same for each function
    Accuracy = (accuracy_score(Ally_test,Ally_pred))*100
    FScore = (f1_score(Ally_test, Ally_pred))*100
    ROC_AUC = roc_auc_score(Ally_test, Ally_pred, multi_class='ovo')
    Precision = precision_score(Ally_test, Ally_pred)
    Recall = recall_score(Ally_test, Ally_pred)

    Table = AllX_test
    Table['By_pred']=Ally_pred
    Table['By_true']=Ally_test
    Table['Accuracy']=Accuracy
    Table['FScore']=FScore
    Table['ROC_AUC']=ROC_AUC
    Table['Precision']=Precision
    Table['Recall']=Recall

    # Calculting true positives and negatives
    conditions = [
```

```
            Table['By_pred'].eq(0) & Table['By_true'].eq(0),
            Table['By_pred'].eq(0) & Table['By_true'].eq(1),
            Table['By_pred'].eq(1) & Table['By_true'].eq(0),
            Table['By_pred'].eq(1) & Table['By_true'].eq(1)
        ]
        choices = ['TN', 'FN', 'FP', 'TP']
        Table['Result'] = np.select(conditions, choices, default=0)

        FemaleDF = Table[Table['Gender']==0.0]
        F_Accuracy = (accuracy_score(FemaleDF['By_true'],
    FemaleDF['By_pred']))*100
        F_FScore = (f1_score(FemaleDF['By_true'],
    FemaleDF['By_pred']))*100
        F_ROC_AUC = roc_auc_score(FemaleDF['By_true'],
    FemaleDF['By_pred'], multi_class='ovo')
        F_Precision = precision_score(FemaleDF['By_true'],
    FemaleDF['By_pred'])
        F_Recall = recall_score(FemaleDF['By_true'], FemaleDF['By_pred'])

        F_FNs = len(FemaleDF[FemaleDF['Result']=='FN'])
        F_TNs = len(FemaleDF[FemaleDF['Result']=='TN'])
        F_FPs = len(FemaleDF[FemaleDF['Result']=='FP'])
        F_TPs = len(FemaleDF[FemaleDF['Result']=='TP'])
        F_All = FemaleDF['Result'].sum()

        # ** EDITING IN HERE, TRUE VALUES FOR FALSE POSITIVE ETC
        F_FalseNegativeRate = (F_FNs/(F_FNs+F_TPs))*100
        F_TrueNegativeRate = (F_TNs/(F_TNs+F_FPs))*100
        F_FalsePositiveRate = (F_FPs/(F_FPs+F_TNs))*100
        F_TruePositiveRate = (F_TPs/(F_TPs+F_FNs))*100

        # Males
        MaleDF = Table[Table['Gender']==1.0]
        M_Accuracy = (accuracy_score(MaleDF['By_true'],
    MaleDF['By_pred']))*100
        M_FScore = (f1_score(MaleDF['By_true'], MaleDF['By_pred']))*100
        M_ROC_AUC = roc_auc_score(MaleDF['By_true'], MaleDF['By_pred'],
    multi_class='ovo')
        M_Precision = precision_score(MaleDF['By_true'],
    MaleDF['By_pred'])
        M_Recall = recall_score(MaleDF['By_true'], MaleDF['By_pred'])

        M_FNs = len(MaleDF[MaleDF['Result']=='FN'])
        M_TNs = len(MaleDF[MaleDF['Result']=='TN'])
        M_FPs = len(MaleDF[MaleDF['Result']=='FP'])
        M_TPs = len(MaleDF[MaleDF['Result']=='TP'])
        M_All = MaleDF['Result'].sum()

        M_FalseNegativeRate = (M_FNs/(M_FNs+M_TPs))*100
        M_TrueNegativeRate = (M_TNs/(M_TNs+M_FPs))*100
```

```
    M_FalsePositiveRate = (M_FPs/(M_FPs+M_TNs))*100
    M_TruePositiveRate = (M_TPs/(M_TPs+M_FNs))*100

    #print("Female False Negative Rate", F_FalseNegativeRate)
    #print("Male False Negative Rate", M_FalseNegativeRate)

    #print("Female True Positive Rate", F_TruePositiveRate)
    #print("Male True PositiveRate", M_TruePositiveRate)

    #print("Female False Positive Rate", F_FalsePositiveRate)
    #print("Male False Positive Rate", M_FalsePositiveRate)

    return Accuracy, FScore, ROC_AUC, Precision, Recall, F_Accuracy,
F_FScore, F_ROC_AUC, F_Precision, F_Recall, F_FNs, F_TNs, F_FPs,
F_TPs, F_FalseNegativeRate, F_TrueNegativeRate, F_FalsePositiveRate,
F_TruePositiveRate, M_Accuracy, M_FScore, M_ROC_AUC, M_Precision,
M_Recall, M_FNs, M_TNs, M_FPs, M_TPs, M_FalseNegativeRate,
M_TrueNegativeRate, M_FalsePositiveRate, M_TruePositiveRate

# Order of results for reference
#Accuracy, FScore, ROC_AUC, Precision, Recall

#F_Accuracy, F_FScore, F_ROC_AUC, F_Precision, F_Recall,
#F_FNs, F_TNs, F_FPs, F_TPs
#F_FalseNegativeRate, F_TrueNegativeRate, F_FalsePositiveRate,
F_TruePositiveRate

#M_Accuracy, M_FScore, M_ROC_AUC, M_Precision, M_Recall,
#M_FNs, M_TNs, M_FPs, M_TPs
#M_FalseNegativeRate, M_TrueNegativeRate, M_FalsePositiveRate,
M_TruePositiveRate

# Example - Can swap in the classifiers here
unbalanced_run(AllX, Ally, AllFeatures, LR)
```

3.1.1 Random Forest - Unbalanced, No Feature Selection

```
experiment_number = np.arange(100)

## RF Results
cols = ['UB_Accuracy', 'UB_FScore', 'UB_ROC_AUC', 'UB_Precision',
'UB_Recall',

        'UB_F_Accuracy', 'UB_F_FScore', 'UB_F_ROC_AUC',
'UB_F_Precision', 'UB_F_Recall',
        'UB_F_FNs', 'UB_F_TNs', 'UB_F_FPs', 'UB_F_TPs',
        'UB_F_FNR', 'UB_F_TNR', 'UB_F_FPR', 'UB_F_TPR',

        'UB_M_Accuracy', 'UB_M_FScore', 'UB_M_ROC_AUC',
'UB_M_Precision', 'UB_M_Recall',
```

```
        'UB_M_FNs', 'UB_M_TNs', 'UB_M_FPs', 'UB_M_TPs',
        'UB_M_FNR', 'UB_M_TNR', 'UB_M_FPR', 'UB_M_TPR',
       ]

Unbal_No_FS_RFdf = pd.DataFrame(columns=cols)

# Scroll left here to see the function that defines it as a balanced
experiment
for i in tqdm(experiment_number):
    UB_Accuracy, UB_FScore, UB_ROC_AUC, UB_Precision, UB_Recall,
UB_F_Accuracy, UB_F_FScore, UB_F_ROC_AUC, UB_F_Precision, UB_F_Recall,
UB_F_FNs, UB_F_TNs, UB_F_FPs, UB_F_TPs, UB_F_FNR, UB_F_TNR, UB_F_FPR,
UB_F_TPR, UB_M_Accuracy, UB_M_FScore, UB_M_ROC_AUC, UB_M_Precision,
UB_M_Recall, UB_M_FNs, UB_M_TNs, UB_M_FPs, UB_M_TPs, UB_M_FNR,
UB_M_TNR, UB_M_FPR, UB_M_TPR = unbalanced_run(AllX, Ally, AllFeatures,
RF)

    list = UB_Accuracy, UB_FScore, UB_ROC_AUC, UB_Precision,
UB_Recall, UB_F_Accuracy, UB_F_FScore, UB_F_ROC_AUC, UB_F_Precision,
UB_F_Recall, UB_F_FNs, UB_F_TNs, UB_F_FPs, UB_F_TPs, UB_F_FNR,
UB_F_TNR, UB_F_FPR, UB_F_TPR, UB_M_Accuracy, UB_M_FScore,
UB_M_ROC_AUC, UB_M_Precision, UB_M_Recall, UB_M_FNs, UB_M_TNs,
UB_M_FPs, UB_M_TPs, UB_M_FNR, UB_M_TNR, UB_M_FPR, UB_M_TPR
    Unbal_No_FS_RFdf.loc[len(Unbal_No_FS_RFdf)] = list

# reset the name of the df so can make multiple outputs with same code
df = Unbal_No_FS_RFdf
print('All Accuracy (Mean)', df['UB_Accuracy'].mean(), 'SD',
df['UB_Accuracy'].std())
print('All FScore (Mean)', df['UB_FScore'].mean(), 'SD',
df['UB_FScore'].std())
print('All ROC_AUC (Mean)', df['UB_ROC_AUC'].mean(), 'SD',
df['UB_ROC_AUC'].std())
print('All Precision (Mean)', df['UB_Precision'].mean(), 'SD',
df['UB_Precision'].std())
print('All Recall (Mean)', df['UB_Recall'].mean(), 'SD',
df['UB_Recall'].std())
print('          ')

print('F Accuracy (Mean)', df['UB_F_Accuracy'].mean(), 'SD',
df['UB_F_Accuracy'].std())
print('F FScore (Mean)', df['UB_F_FScore'].mean(), 'SD',
df['UB_F_FScore'].std())
print('F ROC_AUC (Mean)', df['UB_F_ROC_AUC'].mean(),
df['UB_F_ROC_AUC'].std())
print('F Precision (Mean)', df['UB_F_Precision'].mean(),
df['UB_F_Precision'].std())
print('F Recall (Mean)', df['UB_F_Recall'].mean(),
df['UB_F_Recall'].std())
```

```
print('F FNR (Mean)', df['UB_F_FNR'].mean(), df['UB_F_FNR'].std())
print('F TNR (Mean)', df['UB_F_TNR'].mean(), df['UB_F_TNR'].std())
print('F FPR (Mean)', df['UB_F_FPR'].mean(), df['UB_F_FPR'].std())
print('F TPR (Mean)', df['UB_F_TPR'].mean(), df['UB_F_TPR'].std())
print('          ')


print('M Accuracy (Mean)', df['UB_M_Accuracy'].mean(),
df['UB_M_Accuracy'].std())
print('M FScore (Mean)', df['UB_M_FScore'].mean(),
df['UB_M_FScore'].std())
print('M ROC_AUC (Mean)', df['UB_M_ROC_AUC'].mean(),
df['UB_M_ROC_AUC'].std())
print('M Precision (Mean)', df['UB_M_Precision'].mean(),
df['UB_M_Precision'].std())
print('M Recall (Mean)', df['UB_M_Recall'].mean(),
df['UB_M_Recall'].std())

print('M FNR (Mean)', df['UB_M_FNR'].mean(), df['UB_M_FNR'].std())
print('M TNR (Mean)', df['UB_M_TNR'].mean(), df['UB_M_TNR'].std())
print('M FPR (Mean)', df['UB_M_FPR'].mean(), df['UB_M_FPR'].std())
print('M TPR (Mean)', df['UB_M_TPR'].mean(), df['UB_M_TPR'].std())
print('          ')


print("Female/Male T Tests:")

# This gives us each run
Unbal_No_FS_RFdf.to_csv('Unbal_No_FS_RFdf.csv')
```

3.1.2 Logistic Regression - Unbalanced, No Feature Selection

```
# Logistic Regression, No Feature Selection, Unbalanced Data
experiment_number = np.arange(100)

## RF Results
cols = ['UB_Accuracy', 'UB_FScore', 'UB_ROC_AUC', 'UB_Precision',
'UB_Recall',

        'UB_F_Accuracy', 'UB_F_FScore', 'UB_F_ROC_AUC',
'UB_F_Precision', 'UB_F_Recall',
        'UB_F_FNs', 'UB_F_TNs', 'UB_F_FPs', 'UB_F_TPs',
        'UB_F_FNR', 'UB_F_TNR', 'UB_F_FPR', 'UB_F_TPR',

        'UB_M_Accuracy', 'UB_M_FScore', 'UB_M_ROC_AUC',
'UB_M_Precision', 'UB_M_Recall',
        'UB_M_FNs', 'UB_M_TNs', 'UB_M_FPs', 'UB_M_TPs',
        'UB_M_FNR', 'UB_M_TNR', 'UB_M_FPR', 'UB_M_TPR',
       ]
```

```
Unbal_No_FS_LRdf = pd.DataFrame(columns=cols)

# Scroll left here to see the function that defines it as a balanced
experiment

# NEEDED TO ENSURE THERE WAS AN INDENT

for i in tqdm(experiment_number):
    UB_Accuracy, UB_FScore, UB_ROC_AUC, UB_Precision, UB_Recall,
UB_F_Accuracy, UB_F_FScore, UB_F_ROC_AUC, UB_F_Precision, UB_F_Recall,
UB_F_FNs, UB_F_TNs, UB_F_FPs, UB_F_TPs, UB_F_FNR, UB_F_TNR, UB_F_FPR,
UB_F_TPR, UB_M_Accuracy, UB_M_FScore, UB_M_ROC_AUC, UB_M_Precision,
UB_M_Recall, UB_M_FNs, UB_M_TNs, UB_M_FPs, UB_M_TPs, UB_M_FNR,
UB_M_TNR, UB_M_FPR, UB_M_TPR = unbalanced_run(AllX, Ally, AllFeatures,
LR)

    list = UB_Accuracy, UB_FScore, UB_ROC_AUC, UB_Precision,
UB_Recall, UB_F_Accuracy, UB_F_FScore, UB_F_ROC_AUC, UB_F_Precision,
UB_F_Recall, UB_F_FNs, UB_F_TNs, UB_F_FPs, UB_F_TPs, UB_F_FNR,
UB_F_TNR, UB_F_FPR, UB_F_TPR, UB_M_Accuracy, UB_M_FScore,
UB_M_ROC_AUC, UB_M_Precision, UB_M_Recall, UB_M_FNs, UB_M_TNs,
UB_M_FPs, UB_M_TPs, UB_M_FNR, UB_M_TNR, UB_M_FPR, UB_M_TPR
    Unbal_No_FS_LRdf.loc[len(Unbal_No_FS_LRdf)] = list

# reset the name of the df so can make multiple outputs with same code
df = Unbal_No_FS_LRdf
print('All Accuracy (Mean)', df['UB_Accuracy'].mean(), 'SD',
df['UB_Accuracy'].std())
print('All FScore (Mean)', df['UB_FScore'].mean(), 'SD',
df['UB_FScore'].std())
print('All ROC_AUC (Mean)', df['UB_ROC_AUC'].mean(), 'SD',
df['UB_ROC_AUC'].std())
print('All Precision (Mean)', df['UB_Precision'].mean(), 'SD',
df['UB_Precision'].std())
print('All Recall (Mean)', df['UB_Recall'].mean(), 'SD',
df['UB_Recall'].std())
print('          ')

print('F Accuracy (Mean)', df['UB_F_Accuracy'].mean(), 'SD',
df['UB_F_Accuracy'].std())
print('F FScore (Mean)', df['UB_F_FScore'].mean(), 'SD',
df['UB_F_FScore'].std())
print('F ROC_AUC (Mean)', df['UB_F_ROC_AUC'].mean(),
df['UB_F_ROC_AUC'].std())
print('F Precision (Mean)', df['UB_F_Precision'].mean(),
df['UB_F_Precision'].std())
print('F Recall (Mean)', df['UB_F_Recall'].mean(),
df['UB_F_Recall'].std())

print('F FNR (Mean)', df['UB_F_FNR'].mean(), df['UB_F_FNR'].std())
```

```
print('F TNR (Mean)', df['UB_F_TNR'].mean(), df['UB_F_TNR'].std())
print('F FPR (Mean)', df['UB_F_FPR'].mean(), df['UB_F_FPR'].std())
print('F TPR (Mean)', df['UB_F_TPR'].mean(), df['UB_F_TPR'].std())
print('           ')


print('M Accuracy (Mean)', df['UB_M_Accuracy'].mean(),
df['UB_M_Accuracy'].std())
print('M FScore (Mean)', df['UB_M_FScore'].mean(),
df['UB_M_FScore'].std())
print('M ROC_AUC (Mean)', df['UB_M_ROC_AUC'].mean(),
df['UB_M_ROC_AUC'].std())
print('M Precision (Mean)', df['UB_M_Precision'].mean(),
df['UB_M_Precision'].std())
print('M Recall (Mean)', df['UB_M_Recall'].mean(),
df['UB_M_Recall'].std())

print('M FNR (Mean)', df['UB_M_FNR'].mean(), df['UB_M_FNR'].std())
print('M TNR (Mean)', df['UB_M_TNR'].mean(), df['UB_M_TNR'].std())
print('M FPR (Mean)', df['UB_M_FPR'].mean(), df['UB_M_FPR'].std())
print('M TPR (Mean)', df['UB_M_TPR'].mean(), df['UB_M_TPR'].std())
print('           ')


print("Female/Male T Tests:")

# This gives us each run
Unbal_No_FS_LRdf.to_csv('Unbal_No_FS_LRdf.csv')
```

3.1.3 Support Vector Machine - Unbalanced, No Feature Selection

```
## SVM Results
cols = ['UB_Accuracy', 'UB_FScore', 'UB_ROC_AUC', 'UB_Precision',
'UB_Recall',

        'UB_F_Accuracy', 'UB_F_FScore', 'UB_F_ROC_AUC',
'UB_F_Precision', 'UB_F_Recall',
        'UB_F_FNs', 'UB_F_TNs', 'UB_F_FPs', 'UB_F_TPs',
        'UB_F_FNR', 'UB_F_TNR', 'UB_F_FPR', 'UB_F_TPR',

        'UB_M_Accuracy', 'UB_M_FScore', 'UB_M_ROC_AUC',
'UB_M_Precision', 'UB_M_Recall',
        'UB_M_FNs', 'UB_M_TNs', 'UB_M_FPs', 'UB_M_TPs',
        'UB_M_FNR', 'UB_M_TNR', 'UB_M_FPR', 'UB_M_TPR',
       ]

Unbal_No_FS_SVMdf = pd.DataFrame(columns=cols)
for i in tqdm(experiment_number):
    UB_Accuracy, UB_FScore, UB_ROC_AUC, UB_Precision, UB_Recall,
UB_F_Accuracy, UB_F_FScore, UB_F_ROC_AUC, UB_F_Precision, UB_F_Recall,
```

```
UB_F_FNs, UB_F_TNs, UB_F_FPs, UB_F_TPs, UB_F_FNR, UB_F_TNR, UB_F_FPR,
UB_F_TPR, UB_M_Accuracy, UB_M_FScore, UB_M_ROC_AUC, UB_M_Precision,
UB_M_Recall, UB_M_FNs, UB_M_TNs, UB_M_FPs, UB_M_TPs, UB_M_FNR,
UB_M_TNR, UB_M_FPR, UB_M_TPR = unbalanced_run(AllX, Ally, AllFeatures,
SVM)

    list = UB_Accuracy, UB_FScore, UB_ROC_AUC, UB_Precision,
UB_Recall, UB_F_Accuracy, UB_F_FScore, UB_F_ROC_AUC, UB_F_Precision,
UB_F_Recall, UB_F_FNs, UB_F_TNs, UB_F_FPs, UB_F_TPs, UB_F_FNR,
UB_F_TNR, UB_F_FPR, UB_F_TPR, UB_M_Accuracy, UB_M_FScore,
UB_M_ROC_AUC, UB_M_Precision, UB_M_Recall, UB_M_FNs, UB_M_TNs,
UB_M_FPs, UB_M_TPs, UB_M_FNR, UB_M_TNR, UB_M_FPR, UB_M_TPR
    Unbal_No_FS_SVMdf.loc[len(Unbal_No_FS_SVMdf)] = list

# reset the name of the df so can make multiple outputs with same code
df = Unbal_No_FS_SVMdf
print('All Accuracy (Mean)', df['UB_Accuracy'].mean(), 'SD',
df['UB_Accuracy'].std())
print('All FScore (Mean)', df['UB_FScore'].mean(), 'SD',
df['UB_FScore'].std())
print('All ROC_AUC (Mean)', df['UB_ROC_AUC'].mean(), 'SD',
df['UB_ROC_AUC'].std())
print('All Precision (Mean)', df['UB_Precision'].mean(), 'SD',
df['UB_Precision'].std())
print('All Recall (Mean)', df['UB_Recall'].mean(), 'SD',
df['UB_Recall'].std())
print('          ')

print('F Accuracy (Mean)', df['UB_F_Accuracy'].mean(), 'SD',
df['UB_F_Accuracy'].std())
print('F FScore (Mean)', df['UB_F_FScore'].mean(), 'SD',
df['UB_F_FScore'].std())
print('F ROC_AUC (Mean)', df['UB_F_ROC_AUC'].mean(),
df['UB_F_ROC_AUC'].std())
print('F Precision (Mean)', df['UB_F_Precision'].mean(),
df['UB_F_Precision'].std())
print('F Recall (Mean)', df['UB_F_Recall'].mean(),
df['UB_F_Recall'].std())

print('F FNR (Mean)', df['UB_F_FNR'].mean(), df['UB_F_FNR'].std())
print('F TNR (Mean)', df['UB_F_TNR'].mean(), df['UB_F_TNR'].std())
print('F FPR (Mean)', df['UB_F_FPR'].mean(), df['UB_F_FPR'].std())
print('F TPR (Mean)', df['UB_F_TPR'].mean(), df['UB_F_TPR'].std())
print('          ')


print('M Accuracy (Mean)', df['UB_M_Accuracy'].mean(),
df['UB_M_Accuracy'].std())
print('M FScore (Mean)', df['UB_M_FScore'].mean(),
df['UB_M_FScore'].std())
```

```
print('M ROC_AUC (Mean)', df['UB_M_ROC_AUC'].mean(),
df['UB_M_ROC_AUC'].std())
print('M Precision (Mean)', df['UB_M_Precision'].mean(),
df['UB_M_Precision'].std())
print('M Recall (Mean)', df['UB_M_Recall'].mean(),
df['UB_M_Recall'].std())

print('M FNR (Mean)', df['UB_M_FNR'].mean(), df['UB_M_FNR'].std())
print('M TNR (Mean)', df['UB_M_TNR'].mean(), df['UB_M_TNR'].std())
print('M FPR (Mean)', df['UB_M_FPR'].mean(), df['UB_M_FPR'].std())
print('M TPR (Mean)', df['UB_M_TPR'].mean(), df['UB_M_TPR'].std())
print('          ')


print("Female/Male T Tests:")

# This gives us each run
Unbal_No_FS_SVMdf.to_csv('Unbal_No_FS_SVMdf.csv')
```

3.1.3 Gaussian NB - Unbalanced, No Feature Selection

```
# Gaussian NB, No Feature Selection, Unbalanced Data
experiment_number = np.arange(100)

## RF Results
cols = ['UB_Accuracy', 'UB_FScore', 'UB_ROC_AUC', 'UB_Precision',
'UB_Recall',

        'UB_F_Accuracy', 'UB_F_FScore', 'UB_F_ROC_AUC',
'UB_F_Precision', 'UB_F_Recall',
        'UB_F_FNs', 'UB_F_TNs', 'UB_F_FPs', 'UB_F_TPs',
        'UB_F_FNR', 'UB_F_TNR', 'UB_F_FPR', 'UB_F_TPR',

        'UB_M_Accuracy', 'UB_M_FScore', 'UB_M_ROC_AUC',
'UB_M_Precision', 'UB_M_Recall',
        'UB_M_FNs', 'UB_M_TNs', 'UB_M_FPs', 'UB_M_TPs',
        'UB_M_FNR', 'UB_M_TNR', 'UB_M_FPR', 'UB_M_TPR',
       ]

Unbal_No_FS_GNBdf = pd.DataFrame(columns=cols)

# Scroll left here to see the function that defines it as a balanced
experiment

# NEEDED TO ENSURE THERE WAS AN INDENT

for i in tqdm(experiment_number):
    UB_Accuracy, UB_FScore, UB_ROC_AUC, UB_Precision, UB_Recall,
UB_F_Accuracy, UB_F_FScore, UB_F_ROC_AUC, UB_F_Precision, UB_F_Recall,
UB_F_FNs, UB_F_TNs, UB_F_FPs, UB_F_TPs, UB_F_FNR, UB_F_TNR, UB_F_FPR,
```

```
UB_F_TPR, UB_M_Accuracy, UB_M_FScore, UB_M_ROC_AUC, UB_M_Precision,
UB_M_Recall, UB_M_FNs, UB_M_TNs, UB_M_FPs, UB_M_TPs, UB_M_FNR,
UB_M_TNR, UB_M_FPR, UB_M_TPR = unbalanced_run(AllX, Ally, AllFeatures,
NB)

    list = UB_Accuracy, UB_FScore, UB_ROC_AUC, UB_Precision,
UB_Recall, UB_F_Accuracy, UB_F_FScore, UB_F_ROC_AUC, UB_F_Precision,
UB_F_Recall, UB_F_FNs, UB_F_TNs, UB_F_FPs, UB_F_TPs, UB_F_FNR,
UB_F_TNR, UB_F_FPR, UB_F_TPR, UB_M_Accuracy, UB_M_FScore,
UB_M_ROC_AUC, UB_M_Precision, UB_M_Recall, UB_M_FNs, UB_M_TNs,
UB_M_FPs, UB_M_TPs, UB_M_FNR, UB_M_TNR, UB_M_FPR, UB_M_TPR
    Unbal_No_FS_GNBdf.loc[len(Unbal_No_FS_GNBdf)] = list

# reset the name of the df so can make multiple outputs with same code
df = Unbal_No_FS_GNBdf
print('All Accuracy (Mean)', df['UB_Accuracy'].mean(), 'SD',
df['UB_Accuracy'].std())
print('All FScore (Mean)', df['UB_FScore'].mean(), 'SD',
df['UB_FScore'].std())
print('All ROC_AUC (Mean)', df['UB_ROC_AUC'].mean(), 'SD',
df['UB_ROC_AUC'].std())
print('All Precision (Mean)', df['UB_Precision'].mean(), 'SD',
df['UB_Precision'].std())
print('All Recall (Mean)', df['UB_Recall'].mean(), 'SD',
df['UB_Recall'].std())
print('          ')

print('F Accuracy (Mean)', df['UB_F_Accuracy'].mean(), 'SD',
df['UB_F_Accuracy'].std())
print('F FScore (Mean)', df['UB_F_FScore'].mean(), 'SD',
df['UB_F_FScore'].std())
print('F ROC_AUC (Mean)', df['UB_F_ROC_AUC'].mean(),
df['UB_F_ROC_AUC'].std())
print('F Precision (Mean)', df['UB_F_Precision'].mean(),
df['UB_F_Precision'].std())
print('F Recall (Mean)', df['UB_F_Recall'].mean(),
df['UB_F_Recall'].std())

print('F FNR (Mean)', df['UB_F_FNR'].mean(), df['UB_F_FNR'].std())
print('F TNR (Mean)', df['UB_F_TNR'].mean(), df['UB_F_TNR'].std())
print('F FPR (Mean)', df['UB_F_FPR'].mean(), df['UB_F_FPR'].std())
print('F TPR (Mean)', df['UB_F_TPR'].mean(), df['UB_F_TPR'].std())
print('          ')


print('M Accuracy (Mean)', df['UB_M_Accuracy'].mean(),
df['UB_M_Accuracy'].std())
print('M FScore (Mean)', df['UB_M_FScore'].mean(),
df['UB_M_FScore'].std())
print('M ROC_AUC (Mean)', df['UB_M_ROC_AUC'].mean(),
```

```
df['UB_M_ROC_AUC'].std())
print('M Precision (Mean)', df['UB_M_Precision'].mean(),
df['UB_M_Precision'].std())
print('M Recall (Mean)', df['UB_M_Recall'].mean(),
df['UB_M_Recall'].std())

print('M FNR (Mean)', df['UB_M_FNR'].mean(), df['UB_M_FNR'].std())
print('M TNR (Mean)', df['UB_M_TNR'].mean(), df['UB_M_TNR'].std())
print('M FPR (Mean)', df['UB_M_FPR'].mean(), df['UB_M_FPR'].std())
print('M TPR (Mean)', df['UB_M_TPR'].mean(), df['UB_M_TPR'].std())
print('          ')


print("Female/Male T Tests:")

# This gives us each run
Unbal_No_FS_GNBdf.to_csv('Unbal_No_FS_GNBdf.csv')
```

3.2 Balanced, without Feature Selection

```
# 2. BALANCED, WITHOUT FEATURE SELECTION
def balanced_run(MX, My, FX, Fy, FeatureSet, Classifier):
    MX_train, MX_test, My_train, My_test = train_test_split(MX, My,
test_size=0.30)
    FX_train, FX_test, Fy_train, Fy_test = train_test_split(FX, Fy,
test_size=0.30)

    # Redefine X train so its just desired features (i.e. No Sex)
    BX_train = MX_train.append(FX_train)
    BX_test = MX_test.append(FX_test)
    By_train = My_train.append(Fy_train)
    By_test = My_test.append(Fy_test)

    X_train = BX_train[FeatureSet]
    X_test = BX_test[FeatureSet]

    # Experiment specific hyperparameter tuning - Needed for
parameters
    depth=[2, 8, 16]
    n_estimators = [64, 128, 256]
    RFParams = dict(max_depth=depth, n_estimators=n_estimators)
    LRParams = {"C":np.logspace(-3,3,7), "penalty":["l1","l2"]}
    SVMParams = {'C': [0.001,0.01,0.1,1,10,100,1000],
                 'gamma': [0.001,0.01,0.1,1,10,100,1000]
                }
    NBParams = {'var_smoothing': np.logspace(0,-9, num=100)}

    # Name of Parameters
    if Classifier == LR:
        params = LRParams
    if Classifier == RF:
```

```
        params = RFParams
    if Classifier == SVM:
        params = SVMParams
    if Classifier == NB:
        params = NBParams

    ClassifierParams = GridSearchCV(Classifier, params, cv=5,
scoring='accuracy')
    ClassifierParams.fit(BX_train, By_train)

    if Classifier == LR:
        Model1 = LogisticRegression(**ClassifierParams.best_params_)
    if Classifier == SVM:
        Model1 = SVC(**ClassifierParams.best_params_)
    if Classifier == RF:
        Model1 =
RandomForestClassifier(**ClassifierParams.best_params_)
    if Classifier == NB:
        Model1 = GaussianNB(**ClassifierParams.best_params_)

    Model = Model1.fit(BX_train, By_train)
    By_pred = Model.predict(BX_test)

    # Printing out results - same for each function
    Accuracy = (accuracy_score(By_test,By_pred))*100
    FScore = (f1_score(By_test, By_pred))*100
    ROC_AUC = roc_auc_score(By_test, By_pred, multi_class='ovo')
    Precision = precision_score(By_test, By_pred)
    Recall = recall_score(By_test, By_pred)

    Table = BX_test
    Table['By_pred']=By_pred
    Table['By_true']=By_test
    Table['Accuracy']=Accuracy
    Table['FScore']=FScore
    Table['ROC_AUC']=ROC_AUC
    Table['Precision']=Precision
    Table['Recall']=Recall

    # Calculting true positives and negatives
    conditions = [
        Table['By_pred'].eq(0) & Table['By_true'].eq(0),
        Table['By_pred'].eq(0) & Table['By_true'].eq(1),
        Table['By_pred'].eq(1) & Table['By_true'].eq(0),
        Table['By_pred'].eq(1) & Table['By_true'].eq(1)
    ]
    choices = ['TN', 'FN', 'FP', 'TP']
    Table['Result'] = np.select(conditions, choices, default=0)

    FemaleDF = Table[Table['Gender']==0.0]
```

```
    F_Accuracy = (accuracy_score(FemaleDF['By_true'],
FemaleDF['By_pred']))*100
    F_FScore = (f1_score(FemaleDF['By_true'],
FemaleDF['By_pred']))*100
    F_ROC_AUC = roc_auc_score(FemaleDF['By_true'],
FemaleDF['By_pred'], multi_class='ovo')
    F_Precision = precision_score(FemaleDF['By_true'],
FemaleDF['By_pred'])
    F_Recall = recall_score(FemaleDF['By_true'], FemaleDF['By_pred'])

    F_FNs = len(FemaleDF[FemaleDF['Result']=='FN'])
    F_TNs = len(FemaleDF[FemaleDF['Result']=='TN'])
    F_FPs = len(FemaleDF[FemaleDF['Result']=='FP'])
    F_TPs = len(FemaleDF[FemaleDF['Result']=='TP'])
    F_All = FemaleDF['Result'].sum()

    # ** EDITING IN HERE, TRUE VALUES FOR FALSE POSITIVE ETC
    F_FalseNegativeRate = (F_FNs/(F_FNs+F_TPs))*100
    F_TrueNegativeRate = (F_TNs/(F_TNs+F_FPs))*100
    F_FalsePositiveRate = (F_FPs/(F_FPs+F_TNs))*100
    F_TruePositiveRate = (F_TPs/(F_TPs+F_FNs))*100

    # Males
    MaleDF = Table[Table['Gender']==1.0]
    M_Accuracy = (accuracy_score(MaleDF['By_true'],
MaleDF['By_pred']))*100
    M_FScore = (f1_score(MaleDF['By_true'], MaleDF['By_pred']))*100
    M_ROC_AUC = roc_auc_score(MaleDF['By_true'], MaleDF['By_pred'],
multi_class='ovo')
    M_Precision = precision_score(MaleDF['By_true'],
MaleDF['By_pred'])
    M_Recall = recall_score(MaleDF['By_true'], MaleDF['By_pred'])

    M_FNs = len(MaleDF[MaleDF['Result']=='FN'])
    M_TNs = len(MaleDF[MaleDF['Result']=='TN'])
    M_FPs = len(MaleDF[MaleDF['Result']=='FP'])
    M_TPs = len(MaleDF[MaleDF['Result']=='TP'])
    M_All = MaleDF['Result'].sum()

    M_FalseNegativeRate = (M_FNs/(M_FNs+M_TPs))*100
    M_TrueNegativeRate = (M_TNs/(M_TNs+M_FPs))*100
    M_FalsePositiveRate = (M_FPs/(M_FPs+M_TNs))*100
    M_TruePositiveRate = (M_TPs/(M_TPs+M_FNs))*100

    return Accuracy, FScore, ROC_AUC, Precision, Recall, F_Accuracy,
F_FScore, F_ROC_AUC, F_Precision, F_Recall, F_FNs, F_TNs, F_FPs,
F_TPs, F_FalseNegativeRate, F_TrueNegativeRate, F_FalsePositiveRate,
F_TruePositiveRate, M_Accuracy, M_FScore, M_ROC_AUC, M_Precision,
M_Recall, M_FNs, M_TNs, M_FPs, M_TPs, M_FalseNegativeRate,
M_TrueNegativeRate, M_FalsePositiveRate, M_TruePositiveRate
```

```
# testing experiment
balanced_run(MX, My, FX, Fy, AllFeatures, RF)
```

3.2.1 Random Forest - Balanced, without Feature Selection

```
# RANDOM FOREST, No Feature Selection, Unbalanced Data
experiment_number = np.arange(100)

## RF Results
cols = ['B_Accuracy', 'B_FScore', 'B_ROC_AUC', 'B_Precision',
'B_Recall',

        'B_F_Accuracy', 'B_F_FScore', 'B_F_ROC_AUC', 'B_F_Precision',
'B_F_Recall',
        'B_F_FNs', 'B_F_TNs', 'B_F_FPs', 'B_F_TPs',
        'B_F_FNR', 'B_F_TNR', 'B_F_FPR', 'B_F_TPR',

        'B_M_Accuracy', 'B_M_FScore', 'B_M_ROC_AUC', 'B_M_Precision',
'B_M_Recall',
        'B_M_FNs', 'B_M_TNs', 'B_M_FPs', 'B_M_TPs',
        'B_M_FNR', 'B_M_TNR', 'B_M_FPR', 'B_M_TPR',
       ]

Bal_No_FS_RFdf = pd.DataFrame(columns=cols)
for i in tqdm(experiment_number):
    B_Accuracy, B_FScore, B_ROC_AUC, B_Precision, B_Recall,
B_F_Accuracy, B_F_FScore, B_F_ROC_AUC, B_F_Precision, B_F_Recall,
B_F_FNs, B_F_TNs, B_F_FPs, B_F_TPs, B_F_FNR, B_F_TNR, B_F_FPR,
B_F_TPR, B_M_Accuracy, B_M_FScore, B_M_ROC_AUC, B_M_Precision,
B_M_Recall, B_M_FNs, B_M_TNs, B_M_FPs, B_M_TPs, B_M_FNR, B_M_TNR,
B_M_FPR, B_M_TPR = balanced_run(MX, My, FX, Fy, AllFeatures, RF)

    list = B_Accuracy, B_FScore, B_ROC_AUC, B_Precision, B_Recall,
B_F_Accuracy, B_F_FScore, B_F_ROC_AUC, B_F_Precision, B_F_Recall,
B_F_FNs, B_F_TNs, B_F_FPs, B_F_TPs, B_F_FNR, B_F_TNR, B_F_FPR,
B_F_TPR, B_M_Accuracy, B_M_FScore, B_M_ROC_AUC, B_M_Precision,
B_M_Recall, B_M_FNs, B_M_TNs, B_M_FPs, B_M_TPs, B_M_FNR, B_M_TNR,
B_M_FPR, B_M_TPR
    Bal_No_FS_RFdf.loc[len(Bal_No_FS_RFdf)] = list

# reset the name of the df so can make multiple outputs with same code
df = Bal_No_FS_RFdf
print('All Accuracy (Mean)', df['B_Accuracy'].mean(), 'SD',
df['B_Accuracy'].std())
print('All FScore (Mean)', df['B_FScore'].mean(), 'SD',
df['B_FScore'].std())
print('All ROC_AUC (Mean)', df['B_ROC_AUC'].mean(), 'SD',
df['B_ROC_AUC'].std())
print('All Precision (Mean)', df['B_Precision'].mean(), 'SD',
df['B_Precision'].std())
```

```
print('All Recall (Mean)', df['B_Recall'].mean(), 'SD',
df['B_Recall'].std())
print('           ')

print('F Accuracy (Mean)', df['B_F_Accuracy'].mean(), 'SD',
df['B_F_Accuracy'].std())
print('F FScore (Mean)', df['B_F_FScore'].mean(), 'SD',
df['B_F_FScore'].std())
print('F ROC_AUC (Mean)', df['B_F_ROC_AUC'].mean(),
df['B_F_ROC_AUC'].std())
print('F Precision (Mean)', df['B_F_Precision'].mean(),
df['B_F_Precision'].std())
print('F Recall (Mean)', df['B_F_Recall'].mean(),
df['B_F_Recall'].std())

print('F FNR (Mean)', df['B_F_FNR'].mean(), df['B_F_FNR'].std())
print('F TNR (Mean)', df['B_F_TNR'].mean(), df['B_F_TNR'].std())
print('F FPR (Mean)', df['B_F_FPR'].mean(), df['B_F_FPR'].std())
print('F TPR (Mean)', df['B_F_TPR'].mean(), df['B_F_TPR'].std())
print('           ')

print('M Accuracy (Mean)', df['B_M_Accuracy'].mean(),
df['B_M_Accuracy'].std())
print('M FScore (Mean)', df['B_M_FScore'].mean(),
df['B_M_FScore'].std())
print('M ROC_AUC (Mean)', df['B_M_ROC_AUC'].mean(),
df['B_M_ROC_AUC'].std())
print('M Precision (Mean)', df['B_M_Precision'].mean(),
df['B_M_Precision'].std())
print('M Recall (Mean)', df['B_M_Recall'].mean(),
df['B_M_Recall'].std())

print('M FNR (Mean)', df['B_M_FNR'].mean(), df['B_M_FNR'].std())
print('M TNR (Mean)', df['B_M_TNR'].mean(), df['B_M_TNR'].std())
print('M FPR (Mean)', df['B_M_FPR'].mean(), df['B_M_FPR'].std())
print('M TPR (Mean)', df['B_M_TPR'].mean(), df['B_M_TPR'].std())
print('           ')

# This gives us each run
Bal_No_FS_RFdf.to_csv('Bal_No_FS_RFdf.csv')
```

3.2.2 Logistic Regression - Balanced No Feature Selection

```
# RANDOM FOREST, No Feature Selection, Unbalanced Data
experiment_number = np.arange(100)

## RF Results
cols = ['B_Accuracy', 'B_FScore', 'B_ROC_AUC', 'B_Precision',
'B_Recall',

        'B_F_Accuracy', 'B_F_FScore', 'B_F_ROC_AUC', 'B_F_Precision',
```

```
'B_F_Recall',
        'B_F_FNs', 'B_F_TNs', 'B_F_FPs', 'B_F_TPs',
        'B_F_FNR', 'B_F_TNR', 'B_F_FPR', 'B_F_TPR',

        'B_M_Accuracy', 'B_M_FScore', 'B_M_ROC_AUC', 'B_M_Precision',
'B_M_Recall',
        'B_M_FNs', 'B_M_TNs', 'B_M_FPs', 'B_M_TPs',
        'B_M_FNR', 'B_M_TNR', 'B_M_FPR', 'B_M_TPR',
       ]

Bal_No_FS_LRdf = pd.DataFrame(columns=cols)
for i in tqdm(experiment_number):
    B_Accuracy, B_FScore, B_ROC_AUC, B_Precision, B_Recall,
B_F_Accuracy, B_F_FScore, B_F_ROC_AUC, B_F_Precision, B_F_Recall,
B_F_FNs, B_F_TNs, B_F_FPs, B_F_TPs, B_F_FNR, B_F_TNR, B_F_FPR,
B_F_TPR, B_M_Accuracy, B_M_FScore, B_M_ROC_AUC, B_M_Precision,
B_M_Recall, B_M_FNs, B_M_TNs, B_M_FPs, B_M_TPs, B_M_FNR, B_M_TNR,
B_M_FPR, B_M_TPR = balanced_run(MX, My, FX, Fy, AllFeatures, LR)

    list = B_Accuracy, B_FScore, B_ROC_AUC, B_Precision, B_Recall,
B_F_Accuracy, B_F_FScore, B_F_ROC_AUC, B_F_Precision, B_F_Recall,
B_F_FNs, B_F_TNs, B_F_FPs, B_F_TPs, B_F_FNR, B_F_TNR, B_F_FPR,
B_F_TPR, B_M_Accuracy, B_M_FScore, B_M_ROC_AUC, B_M_Precision,
B_M_Recall, B_M_FNs, B_M_TNs, B_M_FPs, B_M_TPs, B_M_FNR, B_M_TNR,
B_M_FPR, B_M_TPR
    Bal_No_FS_LRdf.loc[len(Bal_No_FS_LRdf)] = list

# reset the name of the df so can make multiple outputs with same code
df = Bal_No_FS_LRdf
print('All Accuracy (Mean)', df['B_Accuracy'].mean(), 'SD',
df['B_Accuracy'].std())
print('All FScore (Mean)', df['B_FScore'].mean(), 'SD',
df['B_FScore'].std())
print('All ROC_AUC (Mean)', df['B_ROC_AUC'].mean(), 'SD',
df['B_ROC_AUC'].std())
print('All Precision (Mean)', df['B_Precision'].mean(), 'SD',
df['B_Precision'].std())
print('All Recall (Mean)', df['B_Recall'].mean(), 'SD',
df['B_Recall'].std())
print('           ')

print('F Accuracy (Mean)', df['B_F_Accuracy'].mean(), 'SD',
df['B_F_Accuracy'].std())
print('F FScore (Mean)', df['B_F_FScore'].mean(), 'SD',
df['B_F_FScore'].std())
print('F ROC_AUC (Mean)', df['B_F_ROC_AUC'].mean(),
df['B_F_ROC_AUC'].std())
print('F Precision (Mean)', df['B_F_Precision'].mean(),
df['B_F_Precision'].std())
print('F Recall (Mean)', df['B_F_Recall'].mean(),
```

```
df['B_F_Recall'].std())

print('F FNR (Mean)', df['B_F_FNR'].mean(), df['B_F_FNR'].std())
print('F TNR (Mean)', df['B_F_TNR'].mean(), df['B_F_TNR'].std())
print('F FPR (Mean)', df['B_F_FPR'].mean(), df['B_F_FPR'].std())
print('F TPR (Mean)', df['B_F_TPR'].mean(), df['B_F_TPR'].std())
print('          ')

print('M Accuracy (Mean)', df['B_M_Accuracy'].mean(),
df['B_M_Accuracy'].std())
print('M FScore (Mean)', df['B_M_FScore'].mean(),
df['B_M_FScore'].std())
print('M ROC_AUC (Mean)', df['B_M_ROC_AUC'].mean(),
df['B_M_ROC_AUC'].std())
print('M Precision (Mean)', df['B_M_Precision'].mean(),
df['B_M_Precision'].std())
print('M Recall (Mean)', df['B_M_Recall'].mean(),
df['B_M_Recall'].std())

print('M FNR (Mean)', df['B_M_FNR'].mean(), df['B_M_FNR'].std())
print('M TNR (Mean)', df['B_M_TNR'].mean(), df['B_M_TNR'].std())
print('M FPR (Mean)', df['B_M_FPR'].mean(), df['B_M_FPR'].std())
print('M TPR (Mean)', df['B_M_TPR'].mean(), df['B_M_TPR'].std())
print('          ')

# This gives us each run
Bal_No_FS_LRdf.to_csv('Bal_No_FS_LRdf.csv')
```

3.2.3 Support Vector Machine - Balanced No Feature Selection

```
experiment_number = np.arange(100)
cols = ['B_Accuracy', 'B_FScore', 'B_ROC_AUC', 'B_Precision',
'B_Recall',

        'B_F_Accuracy', 'B_F_FScore', 'B_F_ROC_AUC', 'B_F_Precision',
'B_F_Recall',
        'B_F_FNs', 'B_F_TNs', 'B_F_FPs', 'B_F_TPs',
        'B_F_FNR', 'B_F_TNR', 'B_F_FPR', 'B_F_TPR',

        'B_M_Accuracy', 'B_M_FScore', 'B_M_ROC_AUC', 'B_M_Precision',
'B_M_Recall',
        'B_M_FNs', 'B_M_TNs', 'B_M_FPs', 'B_M_TPs',
        'B_M_FNR', 'B_M_TNR', 'B_M_FPR', 'B_M_TPR',
       ]

Bal_No_FS_SVMdf = pd.DataFrame(columns=cols)
for i in tqdm(experiment_number):
    B_Accuracy, B_FScore, B_ROC_AUC, B_Precision, B_Recall,
B_F_Accuracy, B_F_FScore, B_F_ROC_AUC, B_F_Precision, B_F_Recall,
B_F_FNs, B_F_TNs, B_F_FPs, B_F_TPs, B_F_FNR, B_F_TNR, B_F_FPR,
B_F_TPR, B_M_Accuracy, B_M_FScore, B_M_ROC_AUC, B_M_Precision,
```

```
B_M_Recall, B_M_FNs, B_M_TNs, B_M_FPs, B_M_TPs, B_M_FNR, B_M_TNR,
B_M_FPR, B_M_TPR = balanced_run(MX, My, FX, Fy, AllFeatures, SVM)

    list = B_Accuracy, B_FScore, B_ROC_AUC, B_Precision, B_Recall,
B_F_Accuracy, B_F_FScore, B_F_ROC_AUC, B_F_Precision, B_F_Recall,
B_F_FNs, B_F_TNs, B_F_FPs, B_F_TPs, B_F_FNR, B_F_TNR, B_F_FPR,
B_F_TPR, B_M_Accuracy, B_M_FScore, B_M_ROC_AUC, B_M_Precision,
B_M_Recall, B_M_FNs, B_M_TNs, B_M_FPs, B_M_TPs, B_M_FNR, B_M_TNR,
B_M_FPR, B_M_TPR
    Bal_No_FS_SVMdf.loc[len(Bal_No_FS_SVMdf)] = list

# reset the name of the df so can make multiple outputs with same code
df = Bal_No_FS_SVMdf
print('All Accuracy (Mean)', df['B_Accuracy'].mean(), 'SD',
df['B_Accuracy'].std())
print('All FScore (Mean)', df['B_FScore'].mean(), 'SD',
df['B_FScore'].std())
print('All ROC_AUC (Mean)', df['B_ROC_AUC'].mean(), 'SD',
df['B_ROC_AUC'].std())
print('All Precision (Mean)', df['B_Precision'].mean(), 'SD',
df['B_Precision'].std())
print('All Recall (Mean)', df['B_Recall'].mean(), 'SD',
df['B_Recall'].std())
print('          ')

print('F Accuracy (Mean)', df['B_F_Accuracy'].mean(), 'SD',
df['B_F_Accuracy'].std())
print('F FScore (Mean)', df['B_F_FScore'].mean(), 'SD',
df['B_F_FScore'].std())
print('F ROC_AUC (Mean)', df['B_F_ROC_AUC'].mean(),
df['B_F_ROC_AUC'].std())
print('F Precision (Mean)', df['B_F_Precision'].mean(),
df['B_F_Precision'].std())
print('F Recall (Mean)', df['B_F_Recall'].mean(),
df['B_F_Recall'].std())

print('F FNR (Mean)', df['B_F_FNR'].mean(), df['B_F_FNR'].std())
print('F TNR (Mean)', df['B_F_TNR'].mean(), df['B_F_TNR'].std())
print('F FPR (Mean)', df['B_F_FPR'].mean(), df['B_F_FPR'].std())
print('F TPR (Mean)', df['B_F_TPR'].mean(), df['B_F_TPR'].std())
print('          ')

print('M Accuracy (Mean)', df['B_M_Accuracy'].mean(),
df['B_M_Accuracy'].std())
print('M FScore (Mean)', df['B_M_FScore'].mean(),
df['B_M_FScore'].std())
print('M ROC_AUC (Mean)', df['B_M_ROC_AUC'].mean(),
df['B_M_ROC_AUC'].std())
print('M Precision (Mean)', df['B_M_Precision'].mean(),
df['B_M_Precision'].std())
```

```
print('M Recall (Mean)', df['B_M_Recall'].mean(),
df['B_M_Recall'].std())

print('M FNR (Mean)', df['B_M_FNR'].mean(), df['B_M_FNR'].std())
print('M TNR (Mean)', df['B_M_TNR'].mean(), df['B_M_TNR'].std())
print('M FPR (Mean)', df['B_M_FPR'].mean(), df['B_M_FPR'].std())
print('M TPR (Mean)', df['B_M_TPR'].mean(), df['B_M_TPR'].std())
print('          ')

# This gives us each run
Bal_No_FS_SVMdf.to_csv('Bal_No_FS_SVMdf.csv')
```

3.2.4 Gaussian NB - Balanced No Feature Selection

```
experiment_number = np.arange(100)

## RF Results
cols = ['B_Accuracy', 'B_FScore', 'B_ROC_AUC', 'B_Precision',
'B_Recall',

        'B_F_Accuracy', 'B_F_FScore', 'B_F_ROC_AUC', 'B_F_Precision',
'B_F_Recall',
        'B_F_FNs', 'B_F_TNs', 'B_F_FPs', 'B_F_TPs',
        'B_F_FNR', 'B_F_TNR', 'B_F_FPR', 'B_F_TPR',

        'B_M_Accuracy', 'B_M_FScore', 'B_M_ROC_AUC', 'B_M_Precision',
'B_M_Recall',
        'B_M_FNs', 'B_M_TNs', 'B_M_FPs', 'B_M_TPs',
        'B_M_FNR', 'B_M_TNR', 'B_M_FPR', 'B_M_TPR',
       ]

Bal_No_FS_NBdf = pd.DataFrame(columns=cols)
for i in tqdm(experiment_number):
    B_Accuracy, B_FScore, B_ROC_AUC, B_Precision, B_Recall,
B_F_Accuracy, B_F_FScore, B_F_ROC_AUC, B_F_Precision, B_F_Recall,
B_F_FNs, B_F_TNs, B_F_FPs, B_F_TPs, B_F_FNR, B_F_TNR, B_F_FPR,
B_F_TPR, B_M_Accuracy, B_M_FScore, B_M_ROC_AUC, B_M_Precision,
B_M_Recall, B_M_FNs, B_M_TNs, B_M_FPs, B_M_TPs, B_M_FNR, B_M_TNR,
B_M_FPR, B_M_TPR = balanced_run(MX, My, FX, Fy, AllFeatures, NB)

    list = B_Accuracy, B_FScore, B_ROC_AUC, B_Precision, B_Recall,
B_F_Accuracy, B_F_FScore, B_F_ROC_AUC, B_F_Precision, B_F_Recall,
B_F_FNs, B_F_TNs, B_F_FPs, B_F_TPs, B_F_FNR, B_F_TNR, B_F_FPR,
B_F_TPR, B_M_Accuracy, B_M_FScore, B_M_ROC_AUC, B_M_Precision,
B_M_Recall, B_M_FNs, B_M_TNs, B_M_FPs, B_M_TPs, B_M_FNR, B_M_TNR,
B_M_FPR, B_M_TPR
    Bal_No_FS_NBdf.loc[len(Bal_No_FS_NBdf)] = list

# reset the name of the df so can make multiple outputs with same code
df = Bal_No_FS_NBdf
```

```
print('All Accuracy (Mean)', df['B_Accuracy'].mean(), 'SD',
df['B_Accuracy'].std())
print('All FScore (Mean)', df['B_FScore'].mean(), 'SD',
df['B_FScore'].std())
print('All ROC_AUC (Mean)', df['B_ROC_AUC'].mean(), 'SD',
df['B_ROC_AUC'].std())
print('All Precision (Mean)', df['B_Precision'].mean(), 'SD',
df['B_Precision'].std())
print('All Recall (Mean)', df['B_Recall'].mean(), 'SD',
df['B_Recall'].std())
print('          ')

print('F Accuracy (Mean)', df['B_F_Accuracy'].mean(), 'SD',
df['B_F_Accuracy'].std())
print('F FScore (Mean)', df['B_F_FScore'].mean(), 'SD',
df['B_F_FScore'].std())
print('F ROC_AUC (Mean)', df['B_F_ROC_AUC'].mean(),
df['B_F_ROC_AUC'].std())
print('F Precision (Mean)', df['B_F_Precision'].mean(),
df['B_F_Precision'].std())
print('F Recall (Mean)', df['B_F_Recall'].mean(),
df['B_F_Recall'].std())

print('F FNR (Mean)', df['B_F_FNR'].mean(), df['B_F_FNR'].std())
print('F TNR (Mean)', df['B_F_TNR'].mean(), df['B_F_TNR'].std())
print('F FPR (Mean)', df['B_F_FPR'].mean(), df['B_F_FPR'].std())
print('F TPR (Mean)', df['B_F_TPR'].mean(), df['B_F_TPR'].std())
print('          ')

print('M Accuracy (Mean)', df['B_M_Accuracy'].mean(),
df['B_M_Accuracy'].std())
print('M FScore (Mean)', df['B_M_FScore'].mean(),
df['B_M_FScore'].std())
print('M ROC_AUC (Mean)', df['B_M_ROC_AUC'].mean(),
df['B_M_ROC_AUC'].std())
print('M Precision (Mean)', df['B_M_Precision'].mean(),
df['B_M_Precision'].std())
print('M Recall (Mean)', df['B_M_Recall'].mean(),
df['B_M_Recall'].std())

print('M FNR (Mean)', df['B_M_FNR'].mean(), df['B_M_FNR'].std())
print('M TNR (Mean)', df['B_M_TNR'].mean(), df['B_M_TNR'].std())
print('M FPR (Mean)', df['B_M_FPR'].mean(), df['B_M_FPR'].std())
print('M TPR (Mean)', df['B_M_TPR'].mean(), df['B_M_TPR'].std())
print('          ')

# This gives us each run
Bal_No_FS_NBdf.to_csv('Bal_No_FS_NBdf.csv')
```

Experiment 3.3.1 Unbalanced Training Data with Feature Selection

```
# Feature Selection Experiment

# 1.
def FSunbalanced_run(X, y, FeatureSet, Classifier):
    AllX_train, AllX_test, Ally_train, Ally_test = train_test_split(X,
y, test_size=0.30)
    X_train = AllX_train[FeatureSet]
    X_test = AllX_test[FeatureSet]

    # Experiment specific hyperparameter tuning - Needed for
parameters
    depth=[2, 8, 16]
    n_estimators = [64, 128, 256]
    RFParams = dict(max_depth=depth, n_estimators=n_estimators)
    LRParams = {"C":np.logspace(-3,3,7), "penalty":["l1","l2"]}
    kernel = ['linear']
    C = [50, 10, 1.0, 0.1, 0.01]
    gamma = ['scale']
    SVMparams_grid = dict(kernel=kernel,C=C,gamma=gamma)
    NBParams = {'var_smoothing': np.logspace(0,-9, num=100)}

    # have to move the GridSearch insive the loop, because SVC needs
the kernel defined as linear
    if Classifier == LR:
        params = LRParams
        grid_search = GridSearchCV(Classifier, params, cv=5,
scoring='accuracy')
        ClassifierParams = grid_search.fit(AllX_train, Ally_train)

    if Classifier == RF:
        params = RFParams
        grid_search = GridSearchCV(Classifier, params, cv=5,
scoring='accuracy')
        ClassifierParams = grid_search.fit(AllX_train, Ally_train)

    #if Classifier == SVM:
        # SVC has to have a linear kernel for RFE to work
        #grid_search = GridSearchCV(estimator=SVC(),
param_grid=SVMparams_grid, n_jobs=-1, cv=5, scoring='accuracy',
error_score=0)

    if Classifier == NB:
        grid_search = GridSearchCV(estimator=GaussianNB(),
param_grid=NBParams, n_jobs=-1, cv=5, scoring='accuracy')
        ClassifierParams = grid_search.fit(AllX_train, Ally_train)
# Grid search results for all giving best parameters

    if Classifier == LR:
```

```
        #Model1 = LogisticRegression(**ClassifierParams.best_params_)
        # Correction here to add in Feature Selection
        Model1 =
RFE(LogisticRegression(**ClassifierParams.best_params_),
n_features_to_select=5, verbose=5)

    if Classifier == SVM:
        #Model1 = SVC(**ClassifierParams.best_params_)
        Model1 = PreppedModel1

    if Classifier == RF:
        #Model1 =
RandomForestClassifier(**ClassifierParams.best_params_)
        Model1 =
RFE(RandomForestClassifier(**ClassifierParams.best_params_),
n_features_to_select=5, verbose=5)

    if Classifier == NB:
        #Model1 = GaussianNB(**ClassifierParams.best_params_)
        Model1 = RFE(GaussianNB(**ClassifierParams.best_params_),
n_features_to_select=250, verbose=5)

    Model = Model1.fit(AllX_train, Ally_train)
    Ally_pred = Model.predict(AllX_test)

    # Printing out results - same for each function
    Accuracy = (accuracy_score(Ally_test,Ally_pred))*100
    FScore = (f1_score(Ally_test, Ally_pred))*100
    ROC_AUC = roc_auc_score(Ally_test, Ally_pred, multi_class='ovo')
    Precision = precision_score(Ally_test, Ally_pred)
    Recall = recall_score(Ally_test, Ally_pred)

    Table = AllX_test
    Table['By_pred']=Ally_pred
    Table['By_true']=Ally_test
    Table['Accuracy']=Accuracy
    Table['FScore']=FScore
    Table['ROC_AUC']=ROC_AUC
    Table['Precision']=Precision
    Table['Recall']=Recall

    # Calculting true positives and negatives
    conditions = [
        Table['By_pred'].eq(0) & Table['By_true'].eq(0),
        Table['By_pred'].eq(0) & Table['By_true'].eq(1),
        Table['By_pred'].eq(1) & Table['By_true'].eq(0),
        Table['By_pred'].eq(1) & Table['By_true'].eq(1)
    ]
    choices = ['TN', 'FN', 'FP', 'TP']
    Table['Result'] = np.select(conditions, choices, default=0)
```

```
    FemaleDF = Table[Table['Gender']==0.0]
    F_Accuracy = (accuracy_score(FemaleDF['By_true'],
FemaleDF['By_pred']))*100
    F_FScore = (f1_score(FemaleDF['By_true'],
FemaleDF['By_pred']))*100
    F_ROC_AUC = roc_auc_score(FemaleDF['By_true'],
FemaleDF['By_pred'], multi_class='ovo')
    F_Precision = precision_score(FemaleDF['By_true'],
FemaleDF['By_pred'])
    F_Recall = recall_score(FemaleDF['By_true'], FemaleDF['By_pred'])

    F_FNs = len(FemaleDF[FemaleDF['Result']=='FN'])
    F_TNs = len(FemaleDF[FemaleDF['Result']=='TN'])
    F_FPs = len(FemaleDF[FemaleDF['Result']=='FP'])
    F_TPs = len(FemaleDF[FemaleDF['Result']=='TP'])
    F_All = FemaleDF['Result'].sum()

    # ** EDITING IN HERE, TRUE VALUES FOR FALSE POSITIVE ETC
    F_FalseNegativeRate = (F_FNs/(F_FNs+F_TPs))*100
    F_TrueNegativeRate = (F_TNs/(F_TNs+F_FPs))*100
    F_FalsePositiveRate = (F_FPs/(F_FPs+F_TNs))*100
    F_TruePositiveRate = (F_TPs/(F_TPs+F_FNs))*100

    # Males
    MaleDF = Table[Table['Gender']==1.0]
    M_Accuracy = (accuracy_score(MaleDF['By_true'],
MaleDF['By_pred']))*100
    M_FScore = (f1_score(MaleDF['By_true'], MaleDF['By_pred']))*100
    M_ROC_AUC = roc_auc_score(MaleDF['By_true'], MaleDF['By_pred'],
multi_class='ovo')
    M_Precision = precision_score(MaleDF['By_true'],
MaleDF['By_pred'])
    M_Recall = recall_score(MaleDF['By_true'], MaleDF['By_pred'])

    M_FNs = len(MaleDF[MaleDF['Result']=='FN'])
    M_TNs = len(MaleDF[MaleDF['Result']=='TN'])
    M_FPs = len(MaleDF[MaleDF['Result']=='FP'])
    M_TPs = len(MaleDF[MaleDF['Result']=='TP'])
    M_All = MaleDF['Result'].sum()

    M_FalseNegativeRate = (M_FNs/(M_FNs+M_TPs))*100
    M_TrueNegativeRate = (M_TNs/(M_TNs+M_FPs))*100
    M_FalsePositiveRate = (M_FPs/(M_FPs+M_TNs))*100
    M_TruePositiveRate = (M_TPs/(M_TPs+M_FNs))*100
    return Accuracy, FScore, ROC_AUC, Precision, Recall, F_Accuracy,
F_FScore, F_ROC_AUC, F_Precision, F_Recall, F_FNs, F_TNs, F_FPs,
F_TPs, F_FalseNegativeRate, F_TrueNegativeRate, F_FalsePositiveRate,
F_TruePositiveRate, M_Accuracy, M_FScore, M_ROC_AUC, M_Precision,
M_Recall, M_FNs, M_TNs, M_FPs, M_TPs, M_FalseNegativeRate,
```

```
M_TrueNegativeRate, M_FalsePositiveRate, M_TruePositiveRate

# Example - Can swap in the classifiers here
FSunbalanced_run(AllX, Ally, AllFeatures, RF)
```

Analysis of differences in feature selection

```
# Feature Selection Experiment

# 1.
def Analyse_FSunbalanced_run(X, y, FeatureSet, Classifier):
    AllX_train, AllX_test, Ally_train, Ally_test = train_test_split(X, 
y, test_size=0.30)
    X_train = AllX_train[FeatureSet]
    X_test = AllX_test[FeatureSet]

    # Experiment specific hyperparameter tuning - Needed for 
parameters
    depth=[2, 8, 16]
    n_estimators = [64, 128, 256]
    RFParams = dict(max_depth=depth, n_estimators=n_estimators)

    if Classifier == RF:
        params = RFParams
        grid_search = GridSearchCV(Classifier, params, cv=5, 
scoring='accuracy')
        ClassifierParams = grid_search.fit(AllX_train, Ally_train)

    if Classifier == RF:
        #Model1 = 
RandomForestClassifier(**ClassifierParams.best_params_)
        Model1 = 
RFE(RandomForestClassifier(**ClassifierParams.best_params_), 
n_features_to_select=5, verbose=5)

    Model = Model1.fit(AllX_train, Ally_train)
    Ally_pred = Model.predict(AllX_test)

    # Printing out results - same for each function
    Accuracy = (accuracy_score(Ally_test,Ally_pred))*100
    FScore = (f1_score(Ally_test, Ally_pred))*100
    ROC_AUC = roc_auc_score(Ally_test, Ally_pred, multi_class='ovo')
    Precision = precision_score(Ally_test, Ally_pred)
    Recall = recall_score(Ally_test, Ally_pred)

    Table = AllX_test
    Table['By_pred']=Ally_pred
    Table['By_true']=Ally_test
    Table['Accuracy']=Accuracy
```

```
    Table['FScore']=FScore
    Table['ROC_AUC']=ROC_AUC
    Table['Precision']=Precision
    Table['Recall']=Recall

    # Calculting true positives and negatives
    conditions = [
        Table['By_pred'].eq(0) & Table['By_true'].eq(0),
        Table['By_pred'].eq(0) & Table['By_true'].eq(1),
        Table['By_pred'].eq(1) & Table['By_true'].eq(0),
        Table['By_pred'].eq(1) & Table['By_true'].eq(1)
    ]
    choices = ['TN', 'FN', 'FP', 'TP']
    Table['Result'] = np.select(conditions, choices, default=0)

    FemaleDF = Table[Table['Gender']==0.0]
    F_Accuracy = (accuracy_score(FemaleDF['By_true'],
FemaleDF['By_pred']))*100
    F_FScore = (f1_score(FemaleDF['By_true'],
FemaleDF['By_pred']))*100
    F_ROC_AUC = roc_auc_score(FemaleDF['By_true'],
FemaleDF['By_pred'], multi_class='ovo')
    F_Precision = precision_score(FemaleDF['By_true'],
FemaleDF['By_pred'])
    F_Recall = recall_score(FemaleDF['By_true'], FemaleDF['By_pred'])

    F_FNs = len(FemaleDF[FemaleDF['Result']=='FN'])
    F_TNs = len(FemaleDF[FemaleDF['Result']=='TN'])
    F_FPs = len(FemaleDF[FemaleDF['Result']=='FP'])
    F_TPs = len(FemaleDF[FemaleDF['Result']=='TP'])
    F_All = FemaleDF['Result'].sum()

    # ** EDITING IN HERE, TRUE VALUES FOR FALSE POSITIVE ETC
    F_FalseNegativeRate = (F_FNs/(F_FNs+F_TPs))*100
    F_TrueNegativeRate = (F_TNs/(F_TNs+F_FPs))*100
    F_FalsePositiveRate = (F_FPs/(F_FPs+F_TNs))*100
    F_TruePositiveRate = (F_TPs/(F_TPs+F_FNs))*100

    # Males
    MaleDF = Table[Table['Gender']==1.0]
    M_Accuracy = (accuracy_score(MaleDF['By_true'],
MaleDF['By_pred']))*100
    M_FScore = (f1_score(MaleDF['By_true'], MaleDF['By_pred']))*100
    M_ROC_AUC = roc_auc_score(MaleDF['By_true'], MaleDF['By_pred'],
multi_class='ovo')
    M_Precision = precision_score(MaleDF['By_true'],
MaleDF['By_pred'])
    M_Recall = recall_score(MaleDF['By_true'], MaleDF['By_pred'])

    M_FNs = len(MaleDF[MaleDF['Result']=='FN'])
```

```
    M_TNs = len(MaleDF[MaleDF['Result']=='TN'])
    M_FPs = len(MaleDF[MaleDF['Result']=='FP'])
    M_TPs = len(MaleDF[MaleDF['Result']=='TP'])
    M_All = MaleDF['Result'].sum()

    M_FalseNegativeRate = (M_FNs/(M_FNs+M_TPs))*100
    M_TrueNegativeRate = (M_TNs/(M_TNs+M_FPs))*100
    M_FalsePositiveRate = (M_FPs/(M_FPs+M_TNs))*100
    M_TruePositiveRate = (M_TPs/(M_TPs+M_FNs))*100

    rfe_features = AllX_train.columns[Model.support_]
    print(rfe_features.sort_values())
    return Accuracy, FScore, ROC_AUC, Precision, Recall, F_Accuracy,
F_FScore, F_ROC_AUC, F_Precision, F_Recall, F_FNs, F_TNs, F_FPs,
F_TPs, F_FalseNegativeRate, F_TrueNegativeRate, F_FalsePositiveRate,
F_TruePositiveRate, M_Accuracy, M_FScore, M_ROC_AUC, M_Precision,
M_Recall, M_FNs, M_TNs, M_FPs, M_TPs, M_FalseNegativeRate,
M_TrueNegativeRate, M_FalsePositiveRate, M_TruePositiveRate

## Example for feature Selection - RF as this has largest sex
disparity
Analyse_FSunbalanced_run(AllX, Ally, AllFeatures, RF)
```

Experiment 3.3.1 Random Forest (Unbalanced Training Data with Feature Selection)

```
experiment_number = np.arange(100)

## RF Results
cols = ['UB_Accuracy', 'UB_FScore', 'UB_ROC_AUC', 'UB_Precision',
'UB_Recall',

        'UB_F_Accuracy', 'UB_F_FScore', 'UB_F_ROC_AUC',
'UB_F_Precision', 'UB_F_Recall',
        'UB_F_FNs', 'UB_F_TNs', 'UB_F_FPs', 'UB_F_TPs',
        'UB_F_FNR', 'UB_F_TNR', 'UB_F_FPR', 'UB_F_TPR',

        'UB_M_Accuracy', 'UB_M_FScore', 'UB_M_ROC_AUC',
'UB_M_Precision', 'UB_M_Recall',
        'UB_M_FNs', 'UB_M_TNs', 'UB_M_FPs', 'UB_M_TPs',
        'UB_M_FNR', 'UB_M_TNR', 'UB_M_FPR', 'UB_M_TPR',
       ]

Unbal_W_FS_RFdf = pd.DataFrame(columns=cols)

# Scroll left here to see the function that defines it as a balanced
experiment

# NEEDED TO ENSURE THERE WAS AN INDENT

for i in tqdm(experiment_number):
    UB_Accuracy, UB_FScore, UB_ROC_AUC, UB_Precision, UB_Recall,
```

```
UB_F_Accuracy, UB_F_FScore, UB_F_ROC_AUC, UB_F_Precision, UB_F_Recall,
UB_F_FNs, UB_F_TNs, UB_F_FPs, UB_F_TPs, UB_F_FNR, UB_F_TNR, UB_F_FPR,
UB_F_TPR, UB_M_Accuracy, UB_M_FScore, UB_M_ROC_AUC, UB_M_Precision,
UB_M_Recall, UB_M_FNs, UB_M_TNs, UB_M_FPs, UB_M_TPs, UB_M_FNR,
UB_M_TNR, UB_M_FPR, UB_M_TPR = FSunbalanced_run(AllX, Ally,
AllFeatures, RF)

    list = UB_Accuracy, UB_FScore, UB_ROC_AUC, UB_Precision,
UB_Recall, UB_F_Accuracy, UB_F_FScore, UB_F_ROC_AUC, UB_F_Precision,
UB_F_Recall, UB_F_FNs, UB_F_TNs, UB_F_FPs, UB_F_TPs, UB_F_FNR,
UB_F_TNR, UB_F_FPR, UB_F_TPR, UB_M_Accuracy, UB_M_FScore,
UB_M_ROC_AUC, UB_M_Precision, UB_M_Recall, UB_M_FNs, UB_M_TNs,
UB_M_FPs, UB_M_TPs, UB_M_FNR, UB_M_TNR, UB_M_FPR, UB_M_TPR
    Unbal_W_FS_RFdf.loc[len(Unbal_W_FS_RFdf)] = list

# reset the name of the df so can make multiple outputs with same code
df = Unbal_W_FS_RFdf
print('All Accuracy (Mean)', df['UB_Accuracy'].mean(), 'SD',
df['UB_Accuracy'].std())
print('All FScore (Mean)', df['UB_FScore'].mean(), 'SD',
df['UB_FScore'].std())
print('All ROC_AUC (Mean)', df['UB_ROC_AUC'].mean(), 'SD',
df['UB_ROC_AUC'].std())
print('All Precision (Mean)', df['UB_Precision'].mean(), 'SD',
df['UB_Precision'].std())
print('All Recall (Mean)', df['UB_Recall'].mean(), 'SD',
df['UB_Recall'].std())
print('          ')

print('F Accuracy (Mean)', df['UB_F_Accuracy'].mean(), 'SD',
df['UB_F_Accuracy'].std())
print('F FScore (Mean)', df['UB_F_FScore'].mean(), 'SD',
df['UB_F_FScore'].std())
print('F ROC_AUC (Mean)', df['UB_F_ROC_AUC'].mean(),
df['UB_F_ROC_AUC'].std())
print('F Precision (Mean)', df['UB_F_Precision'].mean(),
df['UB_F_Precision'].std())
print('F Recall (Mean)', df['UB_F_Recall'].mean(),
df['UB_F_Recall'].std())

print('F FNR (Mean)', df['UB_F_FNR'].mean(), df['UB_F_FNR'].std())
print('F TNR (Mean)', df['UB_F_TNR'].mean(), df['UB_F_TNR'].std())
print('F FPR (Mean)', df['UB_F_FPR'].mean(), df['UB_F_FPR'].std())
print('F TPR (Mean)', df['UB_F_TPR'].mean(), df['UB_F_TPR'].std())
print('          ')


print('M Accuracy (Mean)', df['UB_M_Accuracy'].mean(),
df['UB_M_Accuracy'].std())
print('M FScore (Mean)', df['UB_M_FScore'].mean(),
```

```
df['UB_M_FScore'].std())
print('M ROC_AUC (Mean)', df['UB_M_ROC_AUC'].mean(),
df['UB_M_ROC_AUC'].std())
print('M Precision (Mean)', df['UB_M_Precision'].mean(),
df['UB_M_Precision'].std())
print('M Recall (Mean)', df['UB_M_Recall'].mean(),
df['UB_M_Recall'].std())

print('M FNR (Mean)', df['UB_M_FNR'].mean(), df['UB_M_FNR'].std())
print('M TNR (Mean)', df['UB_M_TNR'].mean(), df['UB_M_TNR'].std())
print('M FPR (Mean)', df['UB_M_FPR'].mean(), df['UB_M_FPR'].std())
print('M TPR (Mean)', df['UB_M_TPR'].mean(), df['UB_M_TPR'].std())
print('          ')


print("Female/Male T Tests:")

# This gives us each run
Unbal_W_FS_RFdf.to_csv('Unbal_W_FS_RFdf.csv')
```

Experiment 3.1.3 Logistic Regression (Unbalanced Training Data with Feature Selection)

```
experiment_number = np.arange(100)

## RF Results
cols = ['UB_Accuracy', 'UB_FScore', 'UB_ROC_AUC', 'UB_Precision',
'UB_Recall',

        'UB_F_Accuracy', 'UB_F_FScore', 'UB_F_ROC_AUC',
'UB_F_Precision', 'UB_F_Recall',
        'UB_F_FNs', 'UB_F_TNs', 'UB_F_FPs', 'UB_F_TPs',
        'UB_F_FNR', 'UB_F_TNR', 'UB_F_FPR', 'UB_F_TPR',

        'UB_M_Accuracy', 'UB_M_FScore', 'UB_M_ROC_AUC',
'UB_M_Precision', 'UB_M_Recall',
        'UB_M_FNs', 'UB_M_TNs', 'UB_M_FPs', 'UB_M_TPs',
        'UB_M_FNR', 'UB_M_TNR', 'UB_M_FPR', 'UB_M_TPR',
       ]

Unbal_W_FS_LRdf = pd.DataFrame(columns=cols)

# Scroll left here to see the function that defines it as a balanced
experiment

# NEEDED TO ENSURE THERE WAS AN INDENT

for i in tqdm(experiment_number):
    UB_Accuracy, UB_FScore, UB_ROC_AUC, UB_Precision, UB_Recall,
UB_F_Accuracy, UB_F_FScore, UB_F_ROC_AUC, UB_F_Precision, UB_F_Recall,
UB_F_FNs, UB_F_TNs, UB_F_FPs, UB_F_TPs, UB_F_FNR, UB_F_TNR, UB_F_FPR,
```

```
UB_F_TPR, UB_M_Accuracy, UB_M_FScore, UB_M_ROC_AUC, UB_M_Precision,
UB_M_Recall, UB_M_FNs, UB_M_TNs, UB_M_FPs, UB_M_TPs, UB_M_FNR,
UB_M_TNR, UB_M_FPR, UB_M_TPR = FSunbalanced_run(AllX, Ally,
AllFeatures, LR)

    list = UB_Accuracy, UB_FScore, UB_ROC_AUC, UB_Precision,
UB_Recall, UB_F_Accuracy, UB_F_FScore, UB_F_ROC_AUC, UB_F_Precision,
UB_F_Recall, UB_F_FNs, UB_F_TNs, UB_F_FPs, UB_F_TPs, UB_F_FNR,
UB_F_TNR, UB_F_FPR, UB_F_TPR, UB_M_Accuracy, UB_M_FScore,
UB_M_ROC_AUC, UB_M_Precision, UB_M_Recall, UB_M_FNs, UB_M_TNs,
UB_M_FPs, UB_M_TPs, UB_M_FNR, UB_M_TNR, UB_M_FPR, UB_M_TPR
    Unbal_W_FS_LRdf.loc[len(Unbal_W_FS_LRdf)] = list

# reset the name of the df so can make multiple outputs with same code
df = Unbal_W_FS_LRdf
print('All Accuracy (Mean)', df['UB_Accuracy'].mean(), 'SD',
df['UB_Accuracy'].std())
print('All FScore (Mean)', df['UB_FScore'].mean(), 'SD',
df['UB_FScore'].std())
print('All ROC_AUC (Mean)', df['UB_ROC_AUC'].mean(), 'SD',
df['UB_ROC_AUC'].std())
print('All Precision (Mean)', df['UB_Precision'].mean(), 'SD',
df['UB_Precision'].std())
print('All Recall (Mean)', df['UB_Recall'].mean(), 'SD',
df['UB_Recall'].std())
print('          ')

print('F Accuracy (Mean)', df['UB_F_Accuracy'].mean(), 'SD',
df['UB_F_Accuracy'].std())
print('F FScore (Mean)', df['UB_F_FScore'].mean(), 'SD',
df['UB_F_FScore'].std())
print('F ROC_AUC (Mean)', df['UB_F_ROC_AUC'].mean(),
df['UB_F_ROC_AUC'].std())
print('F Precision (Mean)', df['UB_F_Precision'].mean(),
df['UB_F_Precision'].std())
print('F Recall (Mean)', df['UB_F_Recall'].mean(),
df['UB_F_Recall'].std())

print('F FNR (Mean)', df['UB_F_FNR'].mean(), df['UB_F_FNR'].std())
print('F TNR (Mean)', df['UB_F_TNR'].mean(), df['UB_F_TNR'].std())
print('F FPR (Mean)', df['UB_F_FPR'].mean(), df['UB_F_FPR'].std())
print('F TPR (Mean)', df['UB_F_TPR'].mean(), df['UB_F_TPR'].std())
print('          ')


print('M Accuracy (Mean)', df['UB_M_Accuracy'].mean(),
df['UB_M_Accuracy'].std())
print('M FScore (Mean)', df['UB_M_FScore'].mean(),
df['UB_M_FScore'].std())
print('M ROC_AUC (Mean)', df['UB_M_ROC_AUC'].mean(),
```

```
df['UB_M_ROC_AUC'].std())
print('M Precision (Mean)', df['UB_M_Precision'].mean(),
df['UB_M_Precision'].std())
print('M Recall (Mean)', df['UB_M_Recall'].mean(),
df['UB_M_Recall'].std())

print('M FNR (Mean)', df['UB_M_FNR'].mean(), df['UB_M_FNR'].std())
print('M TNR (Mean)', df['UB_M_TNR'].mean(), df['UB_M_TNR'].std())
print('M FPR (Mean)', df['UB_M_FPR'].mean(), df['UB_M_FPR'].std())
print('M TPR (Mean)', df['UB_M_TPR'].mean(), df['UB_M_TPR'].std())
print('          ')


print("Female/Male T Tests:")

# This gives us each run
Unbal_W_FS_LRdf.to_csv('Unbal_W_FS_LRdf.csv')
```

Experiment 3.3.3 Support Vector Machine (Unbalanced training data, with Feature Selection)

```
# Prepping parameters for SVM

# SVM Parameters are tuned here due to the duration of the function
when done inside
ExMX_train, ExMX_test, ExMy_train, ExMy_test = train_test_split(MX,
My, test_size=0.30)
ExFX_train, ExFX_test, ExFy_train, ExFy_test = train_test_split(FX,
Fy, test_size=0.30)
EBX_train = ExMX_train.append(ExFX_train)
EBX_test = ExMX_test.append(ExFX_test)
EBy_train = ExMy_train.append(ExFy_train)
EBy_test = ExMy_test.append(ExFy_test)
EX_train = EBX_train[AllFeatures]
EX_test = EBX_test[AllFeatures]
kernel = ['linear']
C = [50, 10, 1.0, 0.1, 0.01]
gamma = ['scale']
SVMparams_grid = dict(kernel=kernel,C=C,gamma=gamma)
grid_search = GridSearchCV(estimator=SVC(), param_grid=SVMparams_grid,
n_jobs=-1, cv=5, scoring='accuracy', error_score=0)
grid_search.fit(EBX_train, EBy_train)
SVCParams = grid_search.fit(EBX_train, EBy_train)
print(SVCParams)
SVCParams.best_params_
PreppedModel1 = RFE(SVC(C=50, gamma='scale', kernel='linear'),
n_features_to_select=5, verbose=5)
PreppedModel1.fit(EBX_train, EBy_train)
predictions = PreppedModel1.predict(EX_test)
```

```
experiment_number = np.arange(20)

## RF Results
cols = ['UB_Accuracy', 'UB_FScore', 'UB_ROC_AUC', 'UB_Precision',
'UB_Recall',

        'UB_F_Accuracy', 'UB_F_FScore', 'UB_F_ROC_AUC',
'UB_F_Precision', 'UB_F_Recall',
        'UB_F_FNs', 'UB_F_TNs', 'UB_F_FPs', 'UB_F_TPs',
        'UB_F_FNR', 'UB_F_TNR', 'UB_F_FPR', 'UB_F_TPR',

        'UB_M_Accuracy', 'UB_M_FScore', 'UB_M_ROC_AUC',
'UB_M_Precision', 'UB_M_Recall',
        'UB_M_FNs', 'UB_M_TNs', 'UB_M_FPs', 'UB_M_TPs',
        'UB_M_FNR', 'UB_M_TNR', 'UB_M_FPR', 'UB_M_TPR',
       ]

Unbal_W_FS_SVMdf1 = pd.DataFrame(columns=cols)

# Scroll left here to see the function that defines it as a balanced
experiment

# NEEDED TO ENSURE THERE WAS AN INDENT

for i in tqdm(experiment_number):
    UB_Accuracy, UB_FScore, UB_ROC_AUC, UB_Precision, UB_Recall,
UB_F_Accuracy, UB_F_FScore, UB_F_ROC_AUC, UB_F_Precision, UB_F_Recall,
UB_F_FNs, UB_F_TNs, UB_F_FPs, UB_F_TPs, UB_F_FNR, UB_F_TNR, UB_F_FPR,
UB_F_TPR, UB_M_Accuracy, UB_M_FScore, UB_M_ROC_AUC, UB_M_Precision,
UB_M_Recall, UB_M_FNs, UB_M_TNs, UB_M_FPs, UB_M_TPs, UB_M_FNR,
UB_M_TNR, UB_M_FPR, UB_M_TPR = FSunbalanced_run(AllX, Ally,
AllFeatures, SVM)

    list = UB_Accuracy, UB_FScore, UB_ROC_AUC, UB_Precision,
UB_Recall, UB_F_Accuracy, UB_F_FScore, UB_F_ROC_AUC, UB_F_Precision,
UB_F_Recall, UB_F_FNs, UB_F_TNs, UB_F_FPs, UB_F_TPs, UB_F_FNR,
UB_F_TNR, UB_F_FPR, UB_F_TPR, UB_M_Accuracy, UB_M_FScore,
UB_M_ROC_AUC, UB_M_Precision, UB_M_Recall, UB_M_FNs, UB_M_TNs,
UB_M_FPs, UB_M_TPs, UB_M_FNR, UB_M_TNR, UB_M_FPR, UB_M_TPR
    Unbal_W_FS_SVMdf1.loc[len(Unbal_W_FS_SVMdf1)] = list

# reset the name of the df so can make multiple outputs with same code
df = Unbal_W_FS_SVMdf1
print('All Accuracy (Mean)', df['UB_Accuracy'].mean(), 'SD',
df['UB_Accuracy'].std())
print('All FScore (Mean)', df['UB_FScore'].mean(), 'SD',
df['UB_FScore'].std())
print('All ROC_AUC (Mean)', df['UB_ROC_AUC'].mean(), 'SD',
df['UB_ROC_AUC'].std())
```

```
print('All Precision (Mean)', df['UB_Precision'].mean(), 'SD',
df['UB_Precision'].std())
print('All Recall (Mean)', df['UB_Recall'].mean(), 'SD',
df['UB_Recall'].std())
print('           ')

print('F Accuracy (Mean)', df['UB_F_Accuracy'].mean(), 'SD',
df['UB_F_Accuracy'].std())
print('F FScore (Mean)', df['UB_F_FScore'].mean(), 'SD',
df['UB_F_FScore'].std())
print('F ROC_AUC (Mean)', df['UB_F_ROC_AUC'].mean(),
df['UB_F_ROC_AUC'].std())
print('F Precision (Mean)', df['UB_F_Precision'].mean(),
df['UB_F_Precision'].std())
print('F Recall (Mean)', df['UB_F_Recall'].mean(),
df['UB_F_Recall'].std())

print('F FNR (Mean)', df['UB_F_FNR'].mean(), df['UB_F_FNR'].std())
print('F TNR (Mean)', df['UB_F_TNR'].mean(), df['UB_F_TNR'].std())
print('F FPR (Mean)', df['UB_F_FPR'].mean(), df['UB_F_FPR'].std())
print('F TPR (Mean)', df['UB_F_TPR'].mean(), df['UB_F_TPR'].std())
print('           ')


print('M Accuracy (Mean)', df['UB_M_Accuracy'].mean(),
df['UB_M_Accuracy'].std())
print('M FScore (Mean)', df['UB_M_FScore'].mean(),
df['UB_M_FScore'].std())
print('M ROC_AUC (Mean)', df['UB_M_ROC_AUC'].mean(),
df['UB_M_ROC_AUC'].std())
print('M Precision (Mean)', df['UB_M_Precision'].mean(),
df['UB_M_Precision'].std())
print('M Recall (Mean)', df['UB_M_Recall'].mean(),
df['UB_M_Recall'].std())

print('M FNR (Mean)', df['UB_M_FNR'].mean(), df['UB_M_FNR'].std())
print('M TNR (Mean)', df['UB_M_TNR'].mean(), df['UB_M_TNR'].std())
print('M FPR (Mean)', df['UB_M_FPR'].mean(), df['UB_M_FPR'].std())
print('M TPR (Mean)', df['UB_M_TPR'].mean(), df['UB_M_TPR'].std())
print('           ')

# This gives us each run
Unbal_W_FS_SVMdf1.to_csv('Unbal_W_FS_SVMdf1.csv')
```

Experiment 3.3.4 Gaussian Naive Bayes (Unbalanced training data, with Feature Selection)

```
experiment_number = np.arange(100)

## RF Results
```

```
cols = ['UB_Accuracy', 'UB_FScore', 'UB_ROC_AUC', 'UB_Precision',
'UB_Recall',

        'UB_F_Accuracy', 'UB_F_FScore', 'UB_F_ROC_AUC',
'UB_F_Precision', 'UB_F_Recall',
        'UB_F_FNs', 'UB_F_TNs', 'UB_F_FPs', 'UB_F_TPs',
        'UB_F_FNR', 'UB_F_TNR', 'UB_F_FPR', 'UB_F_TPR',

        'UB_M_Accuracy', 'UB_M_FScore', 'UB_M_ROC_AUC',
'UB_M_Precision', 'UB_M_Recall',
        'UB_M_FNs', 'UB_M_TNs', 'UB_M_FPs', 'UB_M_TPs',
        'UB_M_FNR', 'UB_M_TNR', 'UB_M_FPR', 'UB_M_TPR',
       ]

Unbal_W_FS_GNBdf = pd.DataFrame(columns=cols)

# Scroll left here to see the function that defines it as a balanced
experiment

# NEEDED TO ENSURE THERE WAS AN INDENT

for i in tqdm(experiment_number):
    UB_Accuracy, UB_FScore, UB_ROC_AUC, UB_Precision, UB_Recall,
UB_F_Accuracy, UB_F_FScore, UB_F_ROC_AUC, UB_F_Precision, UB_F_Recall,
UB_F_FNs, UB_F_TNs, UB_F_FPs, UB_F_TPs, UB_F_FNR, UB_F_TNR, UB_F_FPR,
UB_F_TPR, UB_M_Accuracy, UB_M_FScore, UB_M_ROC_AUC, UB_M_Precision,
UB_M_Recall, UB_M_FNs, UB_M_TNs, UB_M_FPs, UB_M_TPs, UB_M_FNR,
UB_M_TNR, UB_M_FPR, UB_M_TPR = FSunbalanced_run(AllX, Ally,
AllFeatures, NB)

    list = UB_Accuracy, UB_FScore, UB_ROC_AUC, UB_Precision,
UB_Recall, UB_F_Accuracy, UB_F_FScore, UB_F_ROC_AUC, UB_F_Precision,
UB_F_Recall, UB_F_FNs, UB_F_TNs, UB_F_FPs, UB_F_TPs, UB_F_FNR,
UB_F_TNR, UB_F_FPR, UB_F_TPR, UB_M_Accuracy, UB_M_FScore,
UB_M_ROC_AUC, UB_M_Precision, UB_M_Recall, UB_M_FNs, UB_M_TNs,
UB_M_FPs, UB_M_TPs, UB_M_FNR, UB_M_TNR, UB_M_FPR, UB_M_TPR
    Unbal_W_FS_GNBdf.loc[len(Unbal_W_FS_GNBdf)] = list

# reset the name of the df so can make multiple outputs with same code
df = Unbal_W_FS_GNBdf
print('All Accuracy (Mean)', df['UB_Accuracy'].mean(), 'SD',
df['UB_Accuracy'].std())
print('All FScore (Mean)', df['UB_FScore'].mean(), 'SD',
df['UB_FScore'].std())
print('All ROC_AUC (Mean)', df['UB_ROC_AUC'].mean(), 'SD',
df['UB_ROC_AUC'].std())
print('All Precision (Mean)', df['UB_Precision'].mean(), 'SD',
df['UB_Precision'].std())
print('All Recall (Mean)', df['UB_Recall'].mean(), 'SD',
df['UB_Recall'].std())
```

```
print('           ')

print('F Accuracy (Mean)', df['UB_F_Accuracy'].mean(), 'SD',
df['UB_F_Accuracy'].std())
print('F FScore (Mean)', df['UB_F_FScore'].mean(), 'SD',
df['UB_F_FScore'].std())
print('F ROC_AUC (Mean)', df['UB_F_ROC_AUC'].mean(),
df['UB_F_ROC_AUC'].std())
print('F Precision (Mean)', df['UB_F_Precision'].mean(),
df['UB_F_Precision'].std())
print('F Recall (Mean)', df['UB_F_Recall'].mean(),
df['UB_F_Recall'].std())

print('F FNR (Mean)', df['UB_F_FNR'].mean(), df['UB_F_FNR'].std())
print('F TNR (Mean)', df['UB_F_TNR'].mean(), df['UB_F_TNR'].std())
print('F FPR (Mean)', df['UB_F_FPR'].mean(), df['UB_F_FPR'].std())
print('F TPR (Mean)', df['UB_F_TPR'].mean(), df['UB_F_TPR'].std())
print('           ')


print('M Accuracy (Mean)', df['UB_M_Accuracy'].mean(),
df['UB_M_Accuracy'].std())
print('M FScore (Mean)', df['UB_M_FScore'].mean(),
df['UB_M_FScore'].std())
print('M ROC_AUC (Mean)', df['UB_M_ROC_AUC'].mean(),
df['UB_M_ROC_AUC'].std())
print('M Precision (Mean)', df['UB_M_Precision'].mean(),
df['UB_M_Precision'].std())
print('M Recall (Mean)', df['UB_M_Recall'].mean(),
df['UB_M_Recall'].std())

print('M FNR (Mean)', df['UB_M_FNR'].mean(), df['UB_M_FNR'].std())
print('M TNR (Mean)', df['UB_M_TNR'].mean(), df['UB_M_TNR'].std())
print('M FPR (Mean)', df['UB_M_FPR'].mean(), df['UB_M_FPR'].std())
print('M TPR (Mean)', df['UB_M_TPR'].mean(), df['UB_M_TPR'].std())
print('           ')

# This gives us each run
Unbal_W_FS_GNBdf.to_csv('Unbal_W_FS_GNBdf.csv')
```

Experiment 3.4.1 Balaned Training Data with Feature Selection

First - Prepearing SVM model due to speed of RFE and Grid Search when inside the function - tends to crash the operation

```
# SVM Parameters are tuned here due to the duration of the function
when done inside
ExMX_train, ExMX_test, ExMy_train, ExMy_test = train_test_split(MX,
My, test_size=0.30)
ExFX_train, ExFX_test, ExFy_train, ExFy_test = train_test_split(FX,
```

```
Fy, test_size=0.30)
EBX_train = ExMX_train.append(ExFX_train)
EBX_test = ExMX_test.append(ExFX_test)
EBy_train = ExMy_train.append(ExFy_train)
EBy_test = ExMy_test.append(ExFy_test)
EX_train = EBX_train[AllFeatures]
EX_test = EBX_test[AllFeatures]

kernel = ['linear']
C = [50, 10, 1.0, 0.1, 0.01]
gamma = ['scale']
SVMparams_grid = dict(kernel=kernel,C=C,gamma=gamma)
grid_search = GridSearchCV(estimator=SVC(), param_grid=SVMparams_grid,
n_jobs=-1, cv=5, scoring='accuracy', error_score=0)
grid_search.fit(EBX_train, EBy_train)
SVCParams = grid_search.fit(EBX_train, EBy_train)
print(SVCParams)

SVCParams.best_params_

PreppedModel1 = RFE(SVC(C=1, gamma='scale', kernel='linear'),
n_features_to_select=5, verbose=5)

PreppedModel1.fit(EBX_train, EBy_train)
predictions = PreppedModel1.predict(EX_test)

predictions

# 4. Defining Function, Balanced data with feature selection

def FSbalanced_run(MX, My, FX, Fy, FeatureSet, Classifier):
    MX_train, MX_test, My_train, My_test = train_test_split(MX, My,
test_size=0.30)
    FX_train, FX_test, Fy_train, Fy_test = train_test_split(FX, Fy,
test_size=0.30)

    # Redefine X train so its just desired features (i.e. No Sex)
    BX_train = MX_train.append(FX_train)
    BX_test = MX_test.append(FX_test)
    By_train = My_train.append(Fy_train)
    By_test = My_test.append(Fy_test)

    X_train = BX_train[FeatureSet]
    X_test = BX_test[FeatureSet]

    # Experiment specific hyperparameter tuning - Needed for
parameters
    depth=[2, 8, 16]
    n_estimators = [64, 128, 256]
    RFParams = dict(max_depth=depth, n_estimators=n_estimators)
    LRParams = {"C":np.logspace(-3,3,7), "penalty":["l1","l2"]}
```

```
    NBParams = {'var_smoothing': np.logspace(0,-9, num=100)}

    # Name of Parameters
    # have to move the GridSearch insive the loop, because SVC needs
the kernel defined as linear
    if Classifier == LR:
        params = LRParams
        grid_search = GridSearchCV(Classifier, params, cv=5,
scoring='accuracy')
        ClassifierParams = grid_search.fit(X_train, By_train)

    if Classifier == RF:
        params = RFParams
        grid_search = GridSearchCV(Classifier, params, cv=5,
scoring='accuracy')
        ClassifierParams = grid_search.fit(X_train, By_train)

    #if Classifier == SVM:
        # SVC has to have a linear kernel for RFE to work
        #grid_search = GridSearchCV(estimator=SVC(),
param_grid=SVMparams_grid, n_jobs=-1, cv=5, scoring='accuracy',
error_score=0)

    if Classifier == NB:
        grid_search = GridSearchCV(estimator=GaussianNB(),
param_grid=NBParams, n_jobs=-1, cv=5, scoring='accuracy')
        ClassifierParams = grid_search.fit(X_train, By_train)
# Grid search results for all giving best parameters

    if Classifier == LR:
        #Model1 = LogisticRegression(**ClassifierParams.best_params_)
        # Correction here to add in Feature Selection
        Model1 =
RFE(LogisticRegression(**ClassifierParams.best_params_),
n_features_to_select=5, verbose=5)

    if Classifier == SVM:
        #Model1 = SVC(**ClassifierParams.best_params_)
        Model1 = PreppedModel1

    if Classifier == RF:
        #Model1 =
RandomForestClassifier(**ClassifierParams.best_params_)
        Model1 =
RFE(RandomForestClassifier(**ClassifierParams.best_params_),
n_features_to_select=5, verbose=5)

    if Classifier == NB:
        #Model1 = GaussianNB(**ClassifierParams.best_params_)
        Model1 = RFE(GaussianNB(**ClassifierParams.best_params_),
```

```
n_features_to_select=250, verbose=5)

    Model = Model1.fit(X_train, By_train)
    Ally_pred = Model.predict(X_test)

    # Printing out results - same for each function
    Accuracy = (accuracy_score(By_test,Ally_pred))*100
    FScore = (f1_score(By_test, Ally_pred))*100
    ROC_AUC = roc_auc_score(By_test, Ally_pred, multi_class='ovo')
    Precision = precision_score(By_test, Ally_pred)
    Recall = recall_score(By_test, Ally_pred)

    Table = X_test
    Table['By_pred']=Ally_pred
    Table['By_true']=By_test
    Table['Accuracy']=Accuracy
    Table['FScore']=FScore
    Table['ROC_AUC']=ROC_AUC
    Table['Precision']=Precision
    Table['Recall']=Recall

    # Calculting true positives and negatives
    conditions = [
        Table['By_pred'].eq(0) & Table['By_true'].eq(0),
        Table['By_pred'].eq(0) & Table['By_true'].eq(1),
        Table['By_pred'].eq(1) & Table['By_true'].eq(0),
        Table['By_pred'].eq(1) & Table['By_true'].eq(1)
    ]
    choices = ['TN', 'FN', 'FP', 'TP']
    Table['Result'] = np.select(conditions, choices, default=0)

    FemaleDF = Table[Table['Gender']==0.0]
    F_Accuracy = (accuracy_score(FemaleDF['By_true'],
FemaleDF['By_pred']))*100
    F_FScore = (f1_score(FemaleDF['By_true'],
FemaleDF['By_pred']))*100
    F_ROC_AUC = roc_auc_score(FemaleDF['By_true'],
FemaleDF['By_pred'], multi_class='ovo')
    F_Precision = precision_score(FemaleDF['By_true'],
FemaleDF['By_pred'])
    F_Recall = recall_score(FemaleDF['By_true'], FemaleDF['By_pred'])

    F_FNs = len(FemaleDF[FemaleDF['Result']=='FN'])
    F_TNs = len(FemaleDF[FemaleDF['Result']=='TN'])
    F_FPs = len(FemaleDF[FemaleDF['Result']=='FP'])
    F_TPs = len(FemaleDF[FemaleDF['Result']=='TP'])
    F_All = FemaleDF['Result'].sum()

    # ** EDITING IN HERE, TRUE VALUES FOR FALSE POSITIVE ETC
    F_FalseNegativeRate = (F_FNs/(F_FNs+F_TPs))*100
```

```
    F_TrueNegativeRate = (F_TNs/(F_TNs+F_FPs))*100
    F_FalsePositiveRate = (F_FPs/(F_FPs+F_TNs))*100
    F_TruePositiveRate = (F_TPs/(F_TPs+F_FNs))*100

    # Males
    MaleDF = Table[Table['Gender']==1.0]
    M_Accuracy = (accuracy_score(MaleDF['By_true'],
MaleDF['By_pred']))*100
    M_FScore = (f1_score(MaleDF['By_true'], MaleDF['By_pred']))*100
    M_ROC_AUC = roc_auc_score(MaleDF['By_true'], MaleDF['By_pred'],
multi_class='ovo')
    M_Precision = precision_score(MaleDF['By_true'],
MaleDF['By_pred'])
    M_Recall = recall_score(MaleDF['By_true'], MaleDF['By_pred'])

    M_FNs = len(MaleDF[MaleDF['Result']=='FN'])
    M_TNs = len(MaleDF[MaleDF['Result']=='TN'])
    M_FPs = len(MaleDF[MaleDF['Result']=='FP'])
    M_TPs = len(MaleDF[MaleDF['Result']=='TP'])
    M_All = MaleDF['Result'].sum()

    M_FalseNegativeRate = (M_FNs/(M_FNs+M_TPs))*100
    M_TrueNegativeRate = (M_TNs/(M_TNs+M_FPs))*100
    M_FalsePositiveRate = (M_FPs/(M_FPs+M_TNs))*100
    M_TruePositiveRate = (M_TPs/(M_TPs+M_FNs))*100
    return Accuracy, FScore, ROC_AUC, Precision, Recall, F_Accuracy,
F_FScore, F_ROC_AUC, F_Precision, F_Recall, F_FNs, F_TNs, F_FPs,
F_TPs, F_FalseNegativeRate, F_TrueNegativeRate, F_FalsePositiveRate,
F_TruePositiveRate, M_Accuracy, M_FScore, M_ROC_AUC, M_Precision,
M_Recall, M_FNs, M_TNs, M_FPs, M_TPs, M_FalseNegativeRate,
M_TrueNegativeRate, M_FalsePositiveRate, M_TruePositiveRate

# testing experiment
FSbalanced_run(MX, My, FX, Fy, AllFeatures, SVM)
```

Analysing Feature Selection with RF

```
## Example for feature Selection - RF as this has largest sex
disparity
# Feature Selection Experiment

# 1.
def Analyse_FSbalanced_run(MX, My, FX, Fy, FeatureSet, Classifier):
    MX_train, MX_test, My_train, My_test = train_test_split(MX, My,
test_size=0.30)
    FX_train, FX_test, Fy_train, Fy_test = train_test_split(FX, Fy,
test_size=0.30)

    # Redefine X train so its just desired features (i.e. No Sex)
    BX_train = MX_train.append(FX_train)
    BX_test = MX_test.append(FX_test)
```

```
    By_train = My_train.append(Fy_train)
    By_test = My_test.append(Fy_test)

    X_train = BX_train[FeatureSet]
    X_test = BX_test[FeatureSet]

    # Experiment specific hyperparameter tuning - Needed for
parameters
    depth=[2, 8, 16]
    n_estimators = [64, 128, 256]
    RFParams = dict(max_depth=depth, n_estimators=n_estimators)
    if Classifier == RF:
        params = RFParams
        grid_search = GridSearchCV(Classifier, params, cv=5,
scoring='accuracy')
        ClassifierParams = grid_search.fit(X_train, By_train)

    #if Classifier == SVM:
        # SVC has to have a linear kernel for RFE to work
        #grid_search = GridSearchCV(estimator=SVC(),
param_grid=SVMparams_grid, n_jobs=-1, cv=5, scoring='accuracy',
error_score=0)

    if Classifier == RF:
        #Model1 =
RandomForestClassifier(**ClassifierParams.best_params_)
        Model1 =
RFE(RandomForestClassifier(**ClassifierParams.best_params_),
n_features_to_select=5, verbose=5)

    Model = Model1.fit(X_train, By_train)
    Ally_pred = Model.predict(X_test)

    # Printing out results - same for each function
    Accuracy = (accuracy_score(By_test,Ally_pred))*100
    FScore = (f1_score(By_test, Ally_pred))*100
    ROC_AUC = roc_auc_score(By_test, Ally_pred, multi_class='ovo')
    Precision = precision_score(By_test, Ally_pred)
    Recall = recall_score(By_test, Ally_pred)

    Table = X_test
    Table['By_pred']=Ally_pred
    Table['By_true']=By_test
    Table['Accuracy']=Accuracy
    Table['FScore']=FScore
    Table['ROC_AUC']=ROC_AUC
    Table['Precision']=Precision
    Table['Recall']=Recall

    # Calculting true positives and negatives
```

```
    conditions = [
        Table['By_pred'].eq(0) & Table['By_true'].eq(0),
        Table['By_pred'].eq(0) & Table['By_true'].eq(1),
        Table['By_pred'].eq(1) & Table['By_true'].eq(0),
        Table['By_pred'].eq(1) & Table['By_true'].eq(1)
    ]
    choices = ['TN', 'FN', 'FP', 'TP']
    Table['Result'] = np.select(conditions, choices, default=0)

    FemaleDF = Table[Table['Gender']==0.0]
    F_Accuracy = (accuracy_score(FemaleDF['By_true'],
FemaleDF['By_pred']))*100
    F_FScore = (f1_score(FemaleDF['By_true'],
FemaleDF['By_pred']))*100
    F_ROC_AUC = roc_auc_score(FemaleDF['By_true'],
FemaleDF['By_pred'], multi_class='ovo')
    F_Precision = precision_score(FemaleDF['By_true'],
FemaleDF['By_pred'])
    F_Recall = recall_score(FemaleDF['By_true'], FemaleDF['By_pred'])

    F_FNs = len(FemaleDF[FemaleDF['Result']=='FN'])
    F_TNs = len(FemaleDF[FemaleDF['Result']=='TN'])
    F_FPs = len(FemaleDF[FemaleDF['Result']=='FP'])
    F_TPs = len(FemaleDF[FemaleDF['Result']=='TP'])
    F_All = FemaleDF['Result'].sum()

    # ** EDITING IN HERE, TRUE VALUES FOR FALSE POSITIVE ETC
    F_FalseNegativeRate = (F_FNs/(F_FNs+F_TPs))*100
    F_TrueNegativeRate = (F_TNs/(F_TNs+F_FPs))*100
    F_FalsePositiveRate = (F_FPs/(F_FPs+F_TNs))*100
    F_TruePositiveRate = (F_TPs/(F_TPs+F_FNs))*100

    # Males
    MaleDF = Table[Table['Gender']==1.0]
    M_Accuracy = (accuracy_score(MaleDF['By_true'],
MaleDF['By_pred']))*100
    M_FScore = (f1_score(MaleDF['By_true'], MaleDF['By_pred']))*100
    M_ROC_AUC = roc_auc_score(MaleDF['By_true'], MaleDF['By_pred'],
multi_class='ovo')
    M_Precision = precision_score(MaleDF['By_true'],
MaleDF['By_pred'])
    M_Recall = recall_score(MaleDF['By_true'], MaleDF['By_pred'])

    M_FNs = len(MaleDF[MaleDF['Result']=='FN'])
    M_TNs = len(MaleDF[MaleDF['Result']=='TN'])
    M_FPs = len(MaleDF[MaleDF['Result']=='FP'])
    M_TPs = len(MaleDF[MaleDF['Result']=='TP'])
    M_All = MaleDF['Result'].sum()

    M_FalseNegativeRate = (M_FNs/(M_FNs+M_TPs))*100
```

```
    M_TrueNegativeRate = (M_TNs/(M_TNs+M_FPs))*100
    M_FalsePositiveRate = (M_FPs/(M_FPs+M_TNs))*100
    M_TruePositiveRate = (M_TPs/(M_TPs+M_FNs))*100

    rfe_features = BX_train.columns[Model.support_]
    print(rfe_features.sort_values())
    return Accuracy, FScore, ROC_AUC, Precision, Recall, F_Accuracy,
F_FScore, F_ROC_AUC, F_Precision, F_Recall, F_FNs, F_TNs, F_FPs,
F_TPs, F_FalseNegativeRate, F_TrueNegativeRate, F_FalsePositiveRate,
F_TruePositiveRate, M_Accuracy, M_FScore, M_ROC_AUC, M_Precision,
M_Recall, M_FNs, M_TNs, M_FPs, M_TPs, M_FalseNegativeRate,
M_TrueNegativeRate, M_FalsePositiveRate, M_TruePositiveRate
```

```
Analyse_FSbalanced_run(MX, My, FX, Fy, AllFeatures, RF)
```

Experiment 3.4.1 Random Forest (Balanced training data, with Feature Selection)

3.4.1 Random Forest

```
experiment_number = np.arange(100)

## RF Results
cols = ['UB_Accuracy', 'UB_FScore', 'UB_ROC_AUC', 'UB_Precision',
'UB_Recall',

        'UB_F_Accuracy', 'UB_F_FScore', 'UB_F_ROC_AUC',
'UB_F_Precision', 'UB_F_Recall',
        'UB_F_FNs', 'UB_F_TNs', 'UB_F_FPs', 'UB_F_TPs',
        'UB_F_FNR', 'UB_F_TNR', 'UB_F_FPR', 'UB_F_TPR',

        'UB_M_Accuracy', 'UB_M_FScore', 'UB_M_ROC_AUC',
'UB_M_Precision', 'UB_M_Recall',
        'UB_M_FNs', 'UB_M_TNs', 'UB_M_FPs', 'UB_M_TPs',
        'UB_M_FNR', 'UB_M_TNR', 'UB_M_FPR', 'UB_M_TPR',
       ]

Bal_W_FS_RFdf = pd.DataFrame(columns=cols)

# Scroll left here to see the function that defines it as a balanced
experiment

# NEEDED TO ENSURE THERE WAS AN INDENT

for i in tqdm(experiment_number):
    UB_Accuracy, UB_FScore, UB_ROC_AUC, UB_Precision, UB_Recall,
UB_F_Accuracy, UB_F_FScore, UB_F_ROC_AUC, UB_F_Precision, UB_F_Recall,
UB_F_FNs, UB_F_TNs, UB_F_FPs, UB_F_TPs, UB_F_FNR, UB_F_TNR, UB_F_FPR,
UB_F_TPR, UB_M_Accuracy, UB_M_FScore, UB_M_ROC_AUC, UB_M_Precision,
UB_M_Recall, UB_M_FNs, UB_M_TNs, UB_M_FPs, UB_M_TPs, UB_M_FNR,
```

```
UB_M_TNR, UB_M_FPR, UB_M_TPR = FSbalanced_run(MX, My, FX, Fy,
AllFeatures, RF)

    list = UB_Accuracy, UB_FScore, UB_ROC_AUC, UB_Precision,
UB_Recall, UB_F_Accuracy, UB_F_FScore, UB_F_ROC_AUC, UB_F_Precision,
UB_F_Recall, UB_F_FNs, UB_F_TNs, UB_F_FPs, UB_F_TPs, UB_F_FNR,
UB_F_TNR, UB_F_FPR, UB_F_TPR, UB_M_Accuracy, UB_M_FScore,
UB_M_ROC_AUC, UB_M_Precision, UB_M_Recall, UB_M_FNs, UB_M_TNs,
UB_M_FPs, UB_M_TPs, UB_M_FNR, UB_M_TNR, UB_M_FPR, UB_M_TPR
    Bal_W_FS_RFdf.loc[len(Bal_W_FS_RFdf)] = list

# reset the name of the df so can make multiple outputs with same code
df = Bal_W_FS_RFdf
print('All Accuracy (Mean)', df['UB_Accuracy'].mean(), 'SD',
df['UB_Accuracy'].std())
print('All FScore (Mean)', df['UB_FScore'].mean(), 'SD',
df['UB_FScore'].std())
print('All ROC_AUC (Mean)', df['UB_ROC_AUC'].mean(), 'SD',
df['UB_ROC_AUC'].std())
print('All Precision (Mean)', df['UB_Precision'].mean(), 'SD',
df['UB_Precision'].std())
print('All Recall (Mean)', df['UB_Recall'].mean(), 'SD',
df['UB_Recall'].std())
print('          ')

print('F Accuracy (Mean)', df['UB_F_Accuracy'].mean(), 'SD',
df['UB_F_Accuracy'].std())
print('F FScore (Mean)', df['UB_F_FScore'].mean(), 'SD',
df['UB_F_FScore'].std())
print('F ROC_AUC (Mean)', df['UB_F_ROC_AUC'].mean(),
df['UB_F_ROC_AUC'].std())
print('F Precision (Mean)', df['UB_F_Precision'].mean(),
df['UB_F_Precision'].std())
print('F Recall (Mean)', df['UB_F_Recall'].mean(),
df['UB_F_Recall'].std())

print('F FNR (Mean)', df['UB_F_FNR'].mean(), df['UB_F_FNR'].std())
print('F TNR (Mean)', df['UB_F_TNR'].mean(), df['UB_F_TNR'].std())
print('F FPR (Mean)', df['UB_F_FPR'].mean(), df['UB_F_FPR'].std())
print('F TPR (Mean)', df['UB_F_TPR'].mean(), df['UB_F_TPR'].std())
print('          ')


print('M Accuracy (Mean)', df['UB_M_Accuracy'].mean(),
df['UB_M_Accuracy'].std())
print('M FScore (Mean)', df['UB_M_FScore'].mean(),
df['UB_M_FScore'].std())
print('M ROC_AUC (Mean)', df['UB_M_ROC_AUC'].mean(),
df['UB_M_ROC_AUC'].std())
print('M Precision (Mean)', df['UB_M_Precision'].mean(),
```

```
df['UB_M_Precision'].std())
print('M Recall (Mean)', df['UB_M_Recall'].mean(),
df['UB_M_Recall'].std())

print('M FNR (Mean)', df['UB_M_FNR'].mean(), df['UB_M_FNR'].std())
print('M TNR (Mean)', df['UB_M_TNR'].mean(), df['UB_M_TNR'].std())
print('M FPR (Mean)', df['UB_M_FPR'].mean(), df['UB_M_FPR'].std())
print('M TPR (Mean)', df['UB_M_TPR'].mean(), df['UB_M_TPR'].std())
print('          ')

# This gives us each run
Bal_W_FS_RFdf.to_csv('Bal_W_FS_RFdf.csv')
```

3.4.2 Logistic Regression (Balanced training data with Feature Selection)

```
experiment_number = np.arange(100)

## RF Results
cols = ['UB_Accuracy', 'UB_FScore', 'UB_ROC_AUC', 'UB_Precision',
'UB_Recall',

        'UB_F_Accuracy', 'UB_F_FScore', 'UB_F_ROC_AUC',
'UB_F_Precision', 'UB_F_Recall',
        'UB_F_FNs', 'UB_F_TNs', 'UB_F_FPs', 'UB_F_TPs',
        'UB_F_FNR', 'UB_F_TNR', 'UB_F_FPR', 'UB_F_TPR',

        'UB_M_Accuracy', 'UB_M_FScore', 'UB_M_ROC_AUC',
'UB_M_Precision', 'UB_M_Recall',
        'UB_M_FNs', 'UB_M_TNs', 'UB_M_FPs', 'UB_M_TPs',
        'UB_M_FNR', 'UB_M_TNR', 'UB_M_FPR', 'UB_M_TPR',
       ]

Bal_W_FS_LRdf = pd.DataFrame(columns=cols)

# Scroll left here to see the function that defines it as a balanced
experiment

# NEEDED TO ENSURE THERE WAS AN INDENT

for i in tqdm(experiment_number):
    UB_Accuracy, UB_FScore, UB_ROC_AUC, UB_Precision, UB_Recall,
UB_F_Accuracy, UB_F_FScore, UB_F_ROC_AUC, UB_F_Precision, UB_F_Recall,
UB_F_FNs, UB_F_TNs, UB_F_FPs, UB_F_TPs, UB_F_FNR, UB_F_TNR, UB_F_FPR,
UB_F_TPR, UB_M_Accuracy, UB_M_FScore, UB_M_ROC_AUC, UB_M_Precision,
UB_M_Recall, UB_M_FNs, UB_M_TNs, UB_M_FPs, UB_M_TPs, UB_M_FNR,
UB_M_TNR, UB_M_FPR, UB_M_TPR = FSbalanced_run(MX, My, FX, Fy,
AllFeatures, LR)

    list = UB_Accuracy, UB_FScore, UB_ROC_AUC, UB_Precision,
UB_Recall, UB_F_Accuracy, UB_F_FScore, UB_F_ROC_AUC, UB_F_Precision,
```

```
UB_F_Recall, UB_F_FNs, UB_F_TNs, UB_F_FPs, UB_F_TPs, UB_F_FNR,
UB_F_TNR, UB_F_FPR, UB_F_TPR, UB_M_Accuracy, UB_M_FScore,
UB_M_ROC_AUC, UB_M_Precision, UB_M_Recall, UB_M_FNs, UB_M_TNs,
UB_M_FPs, UB_M_TPs, UB_M_FNR, UB_M_TNR, UB_M_FPR, UB_M_TPR
    Bal_W_FS_LRdf.loc[len(Bal_W_FS_LRdf)] = list

# reset the name of the df so can make multiple outputs with same code
df = Bal_W_FS_LRdf
print('All Accuracy (Mean)', df['UB_Accuracy'].mean(), 'SD',
df['UB_Accuracy'].std())
print('All FScore (Mean)', df['UB_FScore'].mean(), 'SD',
df['UB_FScore'].std())
print('All ROC_AUC (Mean)', df['UB_ROC_AUC'].mean(), 'SD',
df['UB_ROC_AUC'].std())
print('All Precision (Mean)', df['UB_Precision'].mean(), 'SD',
df['UB_Precision'].std())
print('All Recall (Mean)', df['UB_Recall'].mean(), 'SD',
df['UB_Recall'].std())
print('          ')

print('F Accuracy (Mean)', df['UB_F_Accuracy'].mean(), 'SD',
df['UB_F_Accuracy'].std())
print('F FScore (Mean)', df['UB_F_FScore'].mean(), 'SD',
df['UB_F_FScore'].std())
print('F ROC_AUC (Mean)', df['UB_F_ROC_AUC'].mean(),
df['UB_F_ROC_AUC'].std())
print('F Precision (Mean)', df['UB_F_Precision'].mean(),
df['UB_F_Precision'].std())
print('F Recall (Mean)', df['UB_F_Recall'].mean(),
df['UB_F_Recall'].std())

print('F FNR (Mean)', df['UB_F_FNR'].mean(), df['UB_F_FNR'].std())
print('F TNR (Mean)', df['UB_F_TNR'].mean(), df['UB_F_TNR'].std())
print('F FPR (Mean)', df['UB_F_FPR'].mean(), df['UB_F_FPR'].std())
print('F TPR (Mean)', df['UB_F_TPR'].mean(), df['UB_F_TPR'].std())
print('          ')


print('M Accuracy (Mean)', df['UB_M_Accuracy'].mean(),
df['UB_M_Accuracy'].std())
print('M FScore (Mean)', df['UB_M_FScore'].mean(),
df['UB_M_FScore'].std())
print('M ROC_AUC (Mean)', df['UB_M_ROC_AUC'].mean(),
df['UB_M_ROC_AUC'].std())
print('M Precision (Mean)', df['UB_M_Precision'].mean(),
df['UB_M_Precision'].std())
print('M Recall (Mean)', df['UB_M_Recall'].mean(),
df['UB_M_Recall'].std())

print('M FNR (Mean)', df['UB_M_FNR'].mean(), df['UB_M_FNR'].std())
```

```
print('M TNR (Mean)', df['UB_M_TNR'].mean(), df['UB_M_TNR'].std())
print('M FPR (Mean)', df['UB_M_FPR'].mean(), df['UB_M_FPR'].std())
print('M TPR (Mean)', df['UB_M_TPR'].mean(), df['UB_M_TPR'].std())
print('          ')

# This gives us each run
Bal_W_FS_LRdf.to_csv('Bal_W_FS_LRdf.csv')
```

3.1.4 Support Vector Machine (Balanced Training Data with Feature Selection)

```
experiment_number = np.arange(10)

## RF Results
cols = ['UB_Accuracy', 'UB_FScore', 'UB_ROC_AUC', 'UB_Precision',
'UB_Recall',

        'UB_F_Accuracy', 'UB_F_FScore', 'UB_F_ROC_AUC',
'UB_F_Precision', 'UB_F_Recall',
        'UB_F_FNs', 'UB_F_TNs', 'UB_F_FPs', 'UB_F_TPs',
        'UB_F_FNR', 'UB_F_TNR', 'UB_F_FPR', 'UB_F_TPR',

        'UB_M_Accuracy', 'UB_M_FScore', 'UB_M_ROC_AUC',
'UB_M_Precision', 'UB_M_Recall',
        'UB_M_FNs', 'UB_M_TNs', 'UB_M_FPs', 'UB_M_TPs',
        'UB_M_FNR', 'UB_M_TNR', 'UB_M_FPR', 'UB_M_TPR',
       ]

Bal_W_FS_SVMdf1 = pd.DataFrame(columns=cols)

# Scroll left here to see the function that defines it as a balanced
experiment

# NEEDED TO ENSURE THERE WAS AN INDENT

for i in tqdm(experiment_number):
    UB_Accuracy, UB_FScore, UB_ROC_AUC, UB_Precision, UB_Recall,
UB_F_Accuracy, UB_F_FScore, UB_F_ROC_AUC, UB_F_Precision, UB_F_Recall,
UB_F_FNs, UB_F_TNs, UB_F_FPs, UB_F_TPs, UB_F_FNR, UB_F_TNR, UB_F_FPR,
UB_F_TPR, UB_M_Accuracy, UB_M_FScore, UB_M_ROC_AUC, UB_M_Precision,
UB_M_Recall, UB_M_FNs, UB_M_TNs, UB_M_FPs, UB_M_TPs, UB_M_FNR,
UB_M_TNR, UB_M_FPR, UB_M_TPR = FSbalanced_run(MX, My, FX, Fy,
AllFeatures, SVM)

    list = UB_Accuracy, UB_FScore, UB_ROC_AUC, UB_Precision,
UB_Recall, UB_F_Accuracy, UB_F_FScore, UB_F_ROC_AUC, UB_F_Precision,
UB_F_Recall, UB_F_FNs, UB_F_TNs, UB_F_FPs, UB_F_TPs, UB_F_FNR,
UB_F_TNR, UB_F_FPR, UB_F_TPR, UB_M_Accuracy, UB_M_FScore,
UB_M_ROC_AUC, UB_M_Precision, UB_M_Recall, UB_M_FNs, UB_M_TNs,
UB_M_FPs, UB_M_TPs, UB_M_FNR, UB_M_TNR, UB_M_FPR, UB_M_TPR
    Bal_W_FS_SVMdf1.loc[len(Bal_W_FS_SVMdf1)] = list
```

```
# reset the name of the df so can make multiple outputs with same code
df = Bal_W_FS_SVMdf1
print('All Accuracy (Mean)', df['UB_Accuracy'].mean(), 'SD',
df['UB_Accuracy'].std())
print('All FScore (Mean)', df['UB_FScore'].mean(), 'SD',
df['UB_FScore'].std())
print('All ROC_AUC (Mean)', df['UB_ROC_AUC'].mean(), 'SD',
df['UB_ROC_AUC'].std())
print('All Precision (Mean)', df['UB_Precision'].mean(), 'SD',
df['UB_Precision'].std())
print('All Recall (Mean)', df['UB_Recall'].mean(), 'SD',
df['UB_Recall'].std())
print('          ')

print('F Accuracy (Mean)', df['UB_F_Accuracy'].mean(), 'SD',
df['UB_F_Accuracy'].std())
print('F FScore (Mean)', df['UB_F_FScore'].mean(), 'SD',
df['UB_F_FScore'].std())
print('F ROC_AUC (Mean)', df['UB_F_ROC_AUC'].mean(),
df['UB_F_ROC_AUC'].std())
print('F Precision (Mean)', df['UB_F_Precision'].mean(),
df['UB_F_Precision'].std())
print('F Recall (Mean)', df['UB_F_Recall'].mean(),
df['UB_F_Recall'].std())

print('F FNR (Mean)', df['UB_F_FNR'].mean(), df['UB_F_FNR'].std())
print('F TNR (Mean)', df['UB_F_TNR'].mean(), df['UB_F_TNR'].std())
print('F FPR (Mean)', df['UB_F_FPR'].mean(), df['UB_F_FPR'].std())
print('F TPR (Mean)', df['UB_F_TPR'].mean(), df['UB_F_TPR'].std())
print('          ')


print('M Accuracy (Mean)', df['UB_M_Accuracy'].mean(),
df['UB_M_Accuracy'].std())
print('M FScore (Mean)', df['UB_M_FScore'].mean(),
df['UB_M_FScore'].std())
print('M ROC_AUC (Mean)', df['UB_M_ROC_AUC'].mean(),
df['UB_M_ROC_AUC'].std())
print('M Precision (Mean)', df['UB_M_Precision'].mean(),
df['UB_M_Precision'].std())
print('M Recall (Mean)', df['UB_M_Recall'].mean(),
df['UB_M_Recall'].std())

print('M FNR (Mean)', df['UB_M_FNR'].mean(), df['UB_M_FNR'].std())
print('M TNR (Mean)', df['UB_M_TNR'].mean(), df['UB_M_TNR'].std())
print('M FPR (Mean)', df['UB_M_FPR'].mean(), df['UB_M_FPR'].std())
print('M TPR (Mean)', df['UB_M_TPR'].mean(), df['UB_M_TPR'].std())
print('          ')
```

```
# This gives us each run
Bal_W_FS_SVMdf1.to_csv('Bal_W_FS_SVMdf1.csv')
```

3.1.4 Gaussian Naive Bayes (Balanced Training Data with Feature Selection)

```
experiment_number = np.arange(100)

## RF Results
cols = ['UB_Accuracy', 'UB_FScore', 'UB_ROC_AUC', 'UB_Precision',
'UB_Recall',

        'UB_F_Accuracy', 'UB_F_FScore', 'UB_F_ROC_AUC',
'UB_F_Precision', 'UB_F_Recall',
        'UB_F_FNs', 'UB_F_TNs', 'UB_F_FPs', 'UB_F_TPs',
        'UB_F_FNR', 'UB_F_TNR', 'UB_F_FPR', 'UB_F_TPR',

        'UB_M_Accuracy', 'UB_M_FScore', 'UB_M_ROC_AUC',
'UB_M_Precision', 'UB_M_Recall',
        'UB_M_FNs', 'UB_M_TNs', 'UB_M_FPs', 'UB_M_TPs',
        'UB_M_FNR', 'UB_M_TNR', 'UB_M_FPR', 'UB_M_TPR',
       ]

Bal_W_FS_NBdf = pd.DataFrame(columns=cols)

# Scroll left here to see the function that defines it as a balanced
experiment

# NEEDED TO ENSURE THERE WAS AN INDENT

for i in tqdm(experiment_number):
    UB_Accuracy, UB_FScore, UB_ROC_AUC, UB_Precision, UB_Recall,
UB_F_Accuracy, UB_F_FScore, UB_F_ROC_AUC, UB_F_Precision, UB_F_Recall,
UB_F_FNs, UB_F_TNs, UB_F_FPs, UB_F_TPs, UB_F_FNR, UB_F_TNR, UB_F_FPR,
UB_F_TPR, UB_M_Accuracy, UB_M_FScore, UB_M_ROC_AUC, UB_M_Precision,
UB_M_Recall, UB_M_FNs, UB_M_TNs, UB_M_FPs, UB_M_TPs, UB_M_FNR,
UB_M_TNR, UB_M_FPR, UB_M_TPR = FSbalanced_run(MX, My, FX, Fy,
AllFeatures, NB)

    list = UB_Accuracy, UB_FScore, UB_ROC_AUC, UB_Precision,
UB_Recall, UB_F_Accuracy, UB_F_FScore, UB_F_ROC_AUC, UB_F_Precision,
UB_F_Recall, UB_F_FNs, UB_F_TNs, UB_F_FPs, UB_F_TPs, UB_F_FNR,
UB_F_TNR, UB_F_FPR, UB_F_TPR, UB_M_Accuracy, UB_M_FScore,
UB_M_ROC_AUC, UB_M_Precision, UB_M_Recall, UB_M_FNs, UB_M_TNs,
UB_M_FPs, UB_M_TPs, UB_M_FNR, UB_M_TNR, UB_M_FPR, UB_M_TPR
    Bal_W_FS_NBdf.loc[len(Bal_W_FS_NBdf)] = list

# reset the name of the df so can make multiple outputs with same code
df = Bal_W_FS_NBdf
print('All Accuracy (Mean)', df['UB_Accuracy'].mean(), 'SD',
```

```
df['UB_Accuracy'].std())
print('All FScore (Mean)', df['UB_FScore'].mean(), 'SD',
df['UB_FScore'].std())
print('All ROC_AUC (Mean)', df['UB_ROC_AUC'].mean(), 'SD',
df['UB_ROC_AUC'].std())
print('All Precision (Mean)', df['UB_Precision'].mean(), 'SD',
df['UB_Precision'].std())
print('All Recall (Mean)', df['UB_Recall'].mean(), 'SD',
df['UB_Recall'].std())
print('          ')

print('F Accuracy (Mean)', df['UB_F_Accuracy'].mean(), 'SD',
df['UB_F_Accuracy'].std())
print('F FScore (Mean)', df['UB_F_FScore'].mean(), 'SD',
df['UB_F_FScore'].std())
print('F ROC_AUC (Mean)', df['UB_F_ROC_AUC'].mean(),
df['UB_F_ROC_AUC'].std())
print('F Precision (Mean)', df['UB_F_Precision'].mean(),
df['UB_F_Precision'].std())
print('F Recall (Mean)', df['UB_F_Recall'].mean(),
df['UB_F_Recall'].std())

print('F FNR (Mean)', df['UB_F_FNR'].mean(), df['UB_F_FNR'].std())
print('F TNR (Mean)', df['UB_F_TNR'].mean(), df['UB_F_TNR'].std())
print('F FPR (Mean)', df['UB_F_FPR'].mean(), df['UB_F_FPR'].std())
print('F TPR (Mean)', df['UB_F_TPR'].mean(), df['UB_F_TPR'].std())
print('          ')


print('M Accuracy (Mean)', df['UB_M_Accuracy'].mean(),
df['UB_M_Accuracy'].std())
print('M FScore (Mean)', df['UB_M_FScore'].mean(),
df['UB_M_FScore'].std())
print('M ROC_AUC (Mean)', df['UB_M_ROC_AUC'].mean(),
df['UB_M_ROC_AUC'].std())
print('M Precision (Mean)', df['UB_M_Precision'].mean(),
df['UB_M_Precision'].std())
print('M Recall (Mean)', df['UB_M_Recall'].mean(),
df['UB_M_Recall'].std())

print('M FNR (Mean)', df['UB_M_FNR'].mean(), df['UB_M_FNR'].std())
print('M TNR (Mean)', df['UB_M_TNR'].mean(), df['UB_M_TNR'].std())
print('M FPR (Mean)', df['UB_M_FPR'].mean(), df['UB_M_FPR'].std())
print('M TPR (Mean)', df['UB_M_TPR'].mean(), df['UB_M_TPR'].std())
print('          ')

# This gives us each run
df = Bal_W_FS_NBdf.to_csv('df = Bal_W_FS_NBdf.csv')
```
