# Supplementary material for: Investigating for bias in healthcare algorithms: a sex-stratified analysis of supervised machine learning models in liver disease prediction
Source: BMJ Health Care Inform. 2022 Apr 24;29(1):e100457. doi: 10.1136/bmjhci-2021-100457 (PMC9039354; doi:10.1136/bmjhci-2021-100457)
Supplement: Supplementary data [file bmjhci-2021-100457supp002.pdf]

Supplementary Material (A), 'Tables in Text'

Manuscript – "Investigating for bias in healthcare algorithms: A sex stratified analysis of supervised machine learning models in liver disease prediction"

Section 2.1 Supplementary: Data Exploration and Initial Analysis

Supplementary Table 1.0 gives the variables included in our dataset and their initial datatypes (gender is later converted to binary numerical form).

Supplementary Table 1.0 Features of the Indian Liver Patient Dataset

Column	Datatype	Description
**Age**	Int64	Age of patient
**Gender**	Object	Sex of patient
**Total Bilirubin (TB)**	Float64	Bilirubin is synthesised by the liver and acts as a marker of liver function.
**Direct Bilirubin (DB)**	Float64	Bilirubin is synthesised by the liver and acts as a marker of function.
**ALP (Alkphos Alkaline Phosphotase)**	Int64	An enzyme produced by the liver acting as a marker of function.
**SGPT Alamine Aminotransferase**	Int64	An enzyme produced by the liver acting as a marker of function.
**SGOT Aspartate Aminotransferase**	Int64	An enzyme produced by the liver acting as a marker of function.
**Total Proteins (TP)**	Float64	The liver synthesis proteins, this count reduces with disease.
**Albumin (ALB)**	Float64	The liver synthesis proteins, this count reduces with disease.
**Selector Field**	Float64	Column that indicates whether a patient has liver disease or not, this has been previously labelled by experts.

2.2 Supplementary; Feature Exploration

Supplementary Table 2.0 presents sex-stratified feature importance ranked by Pearson's correlation coefficient.

Supplementary Table 2.0 Feature importance ranked by Pearson Correlation Coefficient

**Feature Rankings: Pearson Correlation with Target Variable (Diagnosis of Liver Disease)**					
**All Patients**		**Female Patients**		**Male Patients**	
**Feature**	**Pearson Correlation Coefficient**	**Feature**	**Pearson Correlation Coefficient**	**Feature**	**Pearson Correlation Coefficient**
**1. Direct Bilirubin (DB)**	0.25	1. Alkaline Phosphotase (ALP)	0.24	1. Direct Bilirubin (DB)	0.25
**2. Total Bilirubin (TB)**	0.22	2. Direct Bilirubin (DB)	0.22	2. Total Bilirubin (TB)	0.22
**3. Alkaline Phosphotase (ALP)**	0.18	3. Total Bilirubin (TB)	0.22	3. Albumin (ALB)	0.19
**4. SGPT Alamine Aminotransferase**	0.16	4. SGPT Aspartate Aminotransferase (AST)	0.22	4. Age	0.17
**5. A/G Ratio**	0.16	5. SGPT Alamine Aminotransferase	0.19	5. A/G Ratio	0.17
**6. Albumin (ALB)**	0.16	6. A/G Ratio	0.15	6. Alkaline Phosphotase (ALP)	0.16
**7. SGPT Aspartate Aminotransferase (AST)**	0.15	7. Albumin (ALB)	0.07	7. SGPT Alamine Aminotransferase	0.16
**8. Age**	0.14	8. Total Proteins (TP)	0.05	8. SGPT Aspartate Aminotransferase (AST)	0.14
**9. Gender**	0.08	9. Age	0.02	9. Total Proteins (TP)	0.06
**10. Total Proteins (TP)**	0.04	10. Gender	N/A	Gender	N/A
**Sum of correlation**			**1.38**		**1.52**
**Q1**			0.07		0.16
**Q3**			0.22		0.19
**Mean IQR**			**0.145**		**0.175**

Supplementary Table 3.0: Summary Experiment 1 (Unbalanced Training Data, No Feature Selection)

	Random Forest Classifier			Logistic Regression Classifier			Support Vector Machine			Gaussian Naive Bayes		
	Mean	Standard Deviation	T Test Significance (Female to Male)	Mean	Standard Deviation	T Test Significance (Female to Male)	Mean	Standard Deviation	T Test Significance (Female to Male)	Mean	Standard Deviation	T Test Significance (Female to Male)
**All Accuracy**	78.17	2.36		71.31	2.37		79.40	2.50		71.53	2.61	
**All FScore**	76.57	2.86		68.18	2.88		79.29	2.91		64.18	3.65	
**All ROC_AUC**	78.25	2.30		71.42	2.28		79.44	2.47		71.49%	2.33%	
**All Precision**	82.65	3.69		77.04	3.98		80.46	3.95		85.98%	4.12%	
**All Recall**	71.58	4.69		61.37	4.19		78.59	6.01		51.37%	4.36%	
**Females Accuracy**	76.06	4.40	0.00	73.33	3.95	0.01	81.55	4.80	0.02	73.45	4.57	0.02
**Females FScore**	64.09	7.50	0.00	55.24	7.28	0.00	76.03	7.18	0.00	51.11	7.93	0.00
**Females ROC_AUC**	72.55	4.76	0.00	68.04	3.91	0.00	80.91	5.46	0.08	66.66%	3.91%	0.00
**Females Precision**	78.52	10.63	0.00	81.31	11.31	0.30	78.01	9.48	0.00	88.66%	8.11%	0.05
**Females Recall**	55.27	9.49	0.00	42.70	7.85	0.00	76.68	13.47	0.04	36.37%	7.58%	0.00
**Females FNR**	44.73	9.49	0.00	57.30	7.85	0.00	23.32	13.47	0.08	63.63	7.58	0.00
**Females TNR**	89.84	5.69	0.00	93.38	4.64	0.00	85.13	7.89	0.00	96.95	2.26	0.00
**Females FPR**	10.16	5.69	0.00	6.62	4.64	0.00	14.87	7.89	0.00	3.05	2.26	0.00
**Females TPR**	55.27	9.49	0.00	42.70	7.85	0.00	76.68	13.47	0.04	36.37	7.58	0.00
**Males Accuracy**	79.02	2.81		70.49	2.74		78.57	2.85		70.74	3.08	
**Males FScore**	79.72	2.98		71.11	3.14		80.17	2.94		67.30	3.90	
**Males ROC_AUC**	79.36	2.71		70.97	2.71		78.50	2.92		72.20%	2.79%	
**Males Precision**	83.76	3.75		76.44	4.52		81.42	4.09		85.53%	4.25%	
**Males Recall**	76.29	4.82		66.76	4.80		79.26	5.04		55.68%	4.83%	
**Males FNR**	23.71	4.82		33.24	4.80		20.74	5.04		44.32	4.83	
**Males TNR**	82.42	3.99		75.18	4.77		77.73	5.85		88.71	3.66	
**Males FPR**	17.58	3.99		24.82	4.77		22.27	5.85		11.29	3.66	
**Males TPR**	76.29	4.82		66.76	4.80		79.26	5.04		55.68	4.83	

Supplementary Table 4.0 Summary Experiment 2 (Balanced Training Data, No Feature Selection)

	Random Forest Classifier			Logistic Regression Classifier			Support Vector Machine			Gaussian Naive Bayes		
	Mean	Standard Deviation	T Test Significance (Female to Male)	Mean	Standard Deviation	T Test Significance (Female to Male)	Mean	Standard Deviation	T Test Significance (Female to Male)	Mean	Standard Deviation	T Test Significance (Female to Male)
**All Accuracy**	81.66	2.33		74.53	1.96		83.30	1.75		74.75	1.97	
**All FScore**	76.52	3.09		65.63	2.88		80.07	2.25		62.80	3.26	
**All ROC_AUC**	80.22	2.41		72.40	1.97		82.70	1.85		71.67	1.97	
**All Precision**	84.80	3.38		78.52	4.30		82.71	3.84		85.28	3.80	
**All Recall**	69.87	4.42		56.57	3.85		77.91	4.71		49.83	3.77	
**Females Accuracy**	84.75	3.03	0.00	77.71	2.42	0.00	89.03	2.23	0.00	78.47	2.35	0.00
**Females FScore**	71.41	5.74	0.00	51.69	5.87	0.00	82.18	3.72	0.00	51.55	5.38	0.00
**Females ROC_AUC**	78.47	3.76	0.13	67.10	2.86	0.00	86.87	3.01	0.00	67.12	2.59	0.00
**Females Precision**	85.44	6.07	0.88	82.22	8.04	0.00	84.22	5.64	0.14	85.13	7.00	0.37
**Females Recall**	61.70	7.03	0.00	38.12	6.09	0.00	80.88	6.90	0.00	37.19	5.02	0.00
**Females FNR**	38.30	7.03	0.00	61.88	6.09	0.00	19.12	6.90	0.00	62.81	5.02	0.00
**Females TNR**	95.24	2.03	0.00	96.09	2.04	0.00	92.86	3.00	0.00	97.05	1.48	0.00
**Females FPR**	4.76	2.03	0.00	3.91	2.04	0.00	7.14	3.00	0.00	2.95	1.48	0.00
**Females TPR**	61.70	7.03	0.00	38.12	6.09	0.00	80.88	6.90	0.00	37.19	5.02	0.00
**Males Accuracy**	78.58	3.05		71.35	3.22		77.56	2.93		71.04	3.22	
**Males FScore**	79.10	3.21		71.86	3.41		78.78	3.10		68.20	3.96	
**Males ROC_AUC**	79.07	2.93		71.89	3.07		77.80	2.95		72.57	2.82	
**Males Precision**	84.49	3.92		77.47	4.44		81.90	4.62		85.37	4.05	
**Males Recall**	74.59	4.93		67.34	5.33		76.24	5.26		57.01	5.09	
**Males FNR**	25.41	4.93		32.66	5.33		23.76	5.26		42.99	5.09	
**Males TNR**	83.55	4.16		76.44	4.82		79.36	5.69		88.12	3.48	
**Males FPR**	16.45	4.16		23.56	4.82		20.64	5.69		11.88	3.48	
**Males TPR**	74.59	4.93		67.34	5.33		76.24	5.26		57.01	5.09	

Supplementary Table 5.0 - Experiment 3.1.2 – Comparison of evaluation metrics before and after balancing the training data

	Random Forest Classifier				Logistic Regression Classifier				Support Vector Machine				Gaussian Naïve Bayes			
	Unbalanced Training Data		Balanced Training Data		Unbalanced Training Data		Balanced Training Data		Unbalanced Training Data		Balanced Training Data		Unbalanced Training Data		Balanced Trained Data	
	Mean (%)	SD	Mean (%)	SD	Mean (%)	SD	Mean (%)	SD	Mean (%)	SD	Mean (%)	SD	Mean (%)	SD	Mean (%)	SD
**All Accuracy**	78.17	2.36	81.66	2.33	71.31	2.37	74.53	1.96	79.40	2.50	83.30	1.75	71.53	2.61	74.75	1.97
**All ROC_AUC**	78.25	2.30	80.22	2.41	71.42	2.28	72.40	1.97	79.44	2.47	82.70	1.85	71.49	2.33	71.67	1.97
**All Precision**	82.65	3.69	84.80	3.38	77.04	3.98	78.52	4.30	80.46	3.95	82.71	3.84	85.98	4.12	85.28	3.80
**All Recall**	71.58	4.69	69.87	4.42	61.37	4.19	56.57	3.85	78.59	6.01	77.91	4.71	51.37	4.36	49.83	3.77
**Females Accuracy**	76.06	4.40	84.75	3.03	73.33	3.95	77.71	2.42	81.55	4.80	89.03	2.23	73.45	4.57	78.47	2.35
**Females ROC_AUC**	72.55	4.76	78.47	3.76	68.04	3.91	67.10	2.86	80.91	5.46	86.87	3.01	66.66	3.91	67.12	2.59
**Females Precision**	78.52	10.63	85.44	6.07	81.31	11.31	82.22	8.04	78.01	9.48	84.22	5.64	88.66	8.11	85.13	7.00
**Females Recall**	55.27	9.49	61.70	7.03	42.70	7.85	38.12	6.09	76.68	13.47	80.88	6.90	36.37	7.58	37.19	5.02
**Males Accuracy**	79.02	2.81	78.58	3.05	70.49	2.74	71.35	3.22	78.57	2.85	77.56	2.93	70.74	3.08	71.04	3.22
**Males ROC_AUC**	79.36	2.71	79.07	2.93	70.97	2.71	71.89	3.07	78.50	2.92	77.80	2.95	72.20	2.79	72.57	2.82
**Males Precision**	83.76	3.75	84.49	3.92	76.44	4.52	77.47	4.44	81.42	4.09	81.90	4.62	85.53	4.25	85.37	4.05
**Males Recall**	76.29	4.82	74.59	4.93	66.76	4.80	67.34	5.33	79.26	5.04	76.24	5.26	55.68	4.83	57.01	5.09

Supplementary Table 6.0: Summary Experiment 3 (Unbalanced Training Data, With Feature Selection)

	Random Forest Classifier			Logistic Regression Classifier			Support Vector Machine			Gaussian Naive Bayes		
	Mean	Standard Deviation	T Test Significance (Female to Male)	Mean	Standard Deviation	T Test Significance (Female to Male)	Mean	Standard Deviation	T Test Significance (Female to Male)	Mean	Standard Deviation	T Test Significance (Female to Male)
**All Accuracy**	76.88	2.68		70.34	2.31		70.24	2.36		71.56	2.60	
**All FScore**	75.65	2.94		65.88	2.86		64.00	3.24		64.57	3.62	
**All ROC_AUC**	76.92	2.65		70.35	2.21		70.38	2.25		71.67	2.24	
**All Precision**	80.32	4.24		77.38	4.23		81.75	4.61		86.07	3.68	
**All Recall**	71.72	4.10		57.56	3.84		52.77	3.98		51.86	4.50	
**Females Accuracy**	74.45	4.79	0.00	72.40	4.26	0.01	72.22	4.03	0.01	73.93	4.45	0.00
**Females FScore**	63.50	7.71	0.00	53.13	7.06	0.00	50.66	7.56	0.00	52.19	8.13	0.00
**Females ROC_AUC**	71.49	5.08	0.00	66.65	3.92	0.00	65.92	3.96	0.00	67.24	4.06	0.00
**Females Precision**	72.51	9.58	0.00	77.21	8.66	0.44	82.53	9.80	0.90	88.92	7.98	0.03
**Females Recall**	57.62	10.12	0.00	41.05	7.62	0.00	36.98	7.22	0.00	37.43	7.92	0.00
**Females FNR**	42.38	10.12	0.00	58.95	7.62	0.00	63.02	7.22	0.00	62.57	7.92	0.00
**Females TNR**	85.37	6.39	0.00	92.26	3.47	0.00	94.86	3.28	0.00	97.06	2.18	0.00
**Females FPR**	14.63	6.39	0.00	7.74	3.47	0.00	5.14	3.28	0.00	2.94	2.18	0.00
**Females TPR**	57.62	10.12	0.00	41.05	7.62	0.00	36.98	7.22	0.00	37.43	7.92	0.00
**Males Accuracy**	77.87	3.12		69.51	3.19		69.47	2.85		70.62	3.04	
**Males FScore**	78.87	3.14		68.92	3.34		67.16	3.36		67.48	3.98	
**Males ROC_AUC**	78.10	3.19		70.25	3.15		70.82	2.67		72.23	2.73	
**Males Precision**	82.37	4.43		77.45	4.66		81.66	4.77		85.51	4.30	
**Males Recall**	75.83	3.85		62.29	4.25		57.27	4.47		55.97	5.15	
**Males FNR**	24.17	3.85		37.71	4.25		42.73	4.47		44.03	5.15	
**Males TNR**	80.38	5.24		78.22	5.08		84.36	4.50		88.49	3.54	
**Males FPR**	19.62	5.24		21.78	5.08		15.64	4.50		11.51	3.54	
**Males TPR**	75.83	3.85		62.29	4.25		57.27	4.47		55.97	5.15	

Supplementary Table 7.0: Summary Experiment 4 (Balanced Training Data, With Feature Selection)

	Random Forest Classifier			Logistic Regression Classifier			Support Vector Machin			Gaussian Naive Bayes		
	Mean	Standard Deviation	T Test Significance (Female to Male)	Mean	Standard Deviation	T Test Significance (Female to Male)	Mean	Standard Deviation	T Test Significance (Female to Male)	Mean	Standard Deviation	T Test Significance (Female to Male)
**All Accuracy**	80.37	2.83		73.28	2.03		71.60	2.20		73.04	1.77	
**All FScore**	75.34	3.77		62.61	3.27		57.44	3.28		60.22	3.19	
**All ROC_AUC**	79.14	2.95		70.72	2.11		68.43	1.92		69.99	1.75	
**All Precision**	81.66	4.30		78.15	4.40		81.91	4.45		83.13	4.49	
**All Recall**	70.14	4.99		52.42	4.14		44.37	3.66		47.44	4.10	
**Females Accuracy**	83.18	3.55	0.00	76.69	2.55	0.00	74.70	3.06	0.00	75.36	2.89	0.00
**Females FScore**	70.21	6.99	0.00	53.18	5.80	0.00	46.78	5.96	0.00	45.78	6.45	0.00
**Females ROC_AUC**	78.02	4.71	0.46	67.48	3.15	0.00	64.32	2.97	0.00	64.09	3.10	0.00
**Females Precision**	78.65	6.76	0.00	71.91	7.85	0.00	73.34	8.33	0.00	76.37	9.99	0.00
**Females Recall**	63.99	9.10	0.00	42.59	6.27	0.00	34.74	5.85	0.00	33.05	5.84	0.00
**Females FNR**	36.01	9.10	0.00	57.41	6.27	0.00	65.26	5.85	0.00	66.95	5.84	0.00
**Females TNR**	92.05	2.72	0.00	92.37	2.27	0.00	93.90	2.23	0.01	95.12	2.35	0.00
**Females FPR**	7.95	2.72	0.00	7.63	2.27	0.00	6.10	2.23	0.00	4.88	2.35	0.00
**Females TPR**	63.99	9.10	0.00	42.59	6.27	0.00	34.74	5.85	0.00	33.05	5.84	0.00
**Males Accuracy**	77.56	3.23		69.88	2.92		68.50	2.93		70.73	2.73	
**Males FScore**	78.07	3.31		67.57	3.45		63.24	3.93		67.41	3.87	
**Males ROC_AUC**	77.98	3.24		71.05	2.79		70.27	2.67		72.25	2.33	
**Males Precision**	83.25	4.48		81.19	4.86		86.16	4.79		85.72	3.83	
**Males Recall**	73.69	4.33		58.10	4.50		50.11	4.35		55.83	5.34	
**Males FNR**	26.31	4.33		41.90	4.50		49.89	4.35		44.17	5.34	
**Males TNR**	82.26	5.02		84.00	4.40		90.43	3.39		88.68	3.56	
**Males FPR**	17.74	5.02		16.00	4.40		9.57	3.39		11.32	3.56	
**Males TPR**	73.69	4.33		58.10	4.50		50.11	4.35		55.83	5.34	

3. 2 Supplementary, Analysis of Feature Selection

Supplementary Table 8.0 gives the feature rankings assigned by the RFE model when fitted to unbalanced data (Experiment 3) and balanced data (Experiment 4), focusing on RF classifiers.

Supplementary Table 8.0 – Comparison of RFE Feature Rankings when trained on unbalanced and balanced training data

Top 5 feature selected by Recursive Feature Elimination (RFE) on unbalanced and balanced training data			
Experiment 3 (Unbalanced Training Data)		Experiment 4 (Balanced Training Data)	
Rank	Feature	Rank	Feature
1.	A/G Ratio	1.	ALP (Alkphos Alkaline Phosphotase)
2.	ALP (Alkphos Alkaline Phosphotase)	2.	Gender
3.	SGOT Aspartate Aminotransferase	3.	SGOT Aspartate Aminotransferase
4.	SGPT Alamine Aminotransferate	4.	SGPT Alamine Aminotransferate
5.	Total Bilirubin (TB)	5.	Total Protiens (TP)
