# Supplementary material for: Investigating for bias in healthcare algorithms: a sex-stratified analysis of supervised machine learning models in liver disease prediction
Source: BMJ Health Care Inform. 2022 Apr 24;29(1):e100457. doi: 10.1136/bmjhci-2021-100457 (PMC9039354; doi:10.1136/bmjhci-2021-100457)
Supplement: Supplementary data [file bmjhci-2021-100457supp003.pdf]

Summary of Results for Experiment 3.1.1

	**Random Forest Classifier**			**Logistic Regression Classifier**			**Support Vector Machine**			**Gaussian Naive Bayes**		
	Mean	Standard Deviation	T Test Significance (Female to Male)	Mean	Standard Deviation	T Test Significance (Female to Male)	Mean	Standard Deviation	T Test Significance (Female to Male)	Mean	Standard Deviation	T Test Significance (Female to Male)
**All Accuracy**	78.17	2.36		71.31	2.37		79.40	2.50		71.53	2.61	
**All FScore**	76.57	2.86		68.18	2.88		79.29	2.91		64.18	3.65	
**All ROC_AUC**	78.25%	2.30%		71.42%	2.28%		79.44%	2.47%		71.49%	2.33%	
**All Precision**	82.65%	3.69%		77.04%	3.98%		80.46%	3.95%		85.98%	4.12%	
**All Recall**	71.58%	4.69%		61.37%	4.19%		78.59%	6.01%		51.37%	4.36%	
Females Accuracy	76.06	4.40	0.00	73.33	3.95	0.01	81.55	4.80	0.02	73.45	4.57	0.02
Females FScore	64.09	7.50	0.00	55.24	7.28	0.00	76.03	7.18	0.00	51.11	7.93	0.00
Females ROC_AUC	72.55%	4.76%	0.00	68.04%	3.91%	0.00	80.91%	5.46%	0.08	66.66%	3.91%	0.00
Females Precision	78.52%	10.63%	0.00	81.31%	11.31%	0.00	78.01%	9.48%	0.00	88.66%	8.11%	0.05
Females Recall	55.27%	9.49%	0.00	42.70%	7.85%	0.00	76.68%	13.47%	0.04	36.37%	7.58%	0.00
**Females FNR**	44.73	9.49	0.00	57.30	7.85	0.00	23.32	13.47	0.08	63.63	7.58	0.00
**Females TNR**	89.84	5.69	0.00	93.38	4.64	0.00	85.13	7.89	0.00	96.95	2.26	0.00
**Females FPR**	10.16	5.69	0.00	6.62	4.64	0.00	14.87	7.89	0.00	3.05	2.26	0.00
**Females TPR**	55.27	9.49	0.00	42.70	7.85	0.00	76.68	13.47	0.04	36.37	7.58	0.00
**Males Accuracy**	79.02	2.81		70.49	2.74		78.57	2.85		70.74	3.08	
**Males FScore**	79.72	2.98		71.11	3.14		80.17	2.94		67.30	3.90	
**Males ROC_AUC**	79.36%	2.71%		70.97%	2.71%		78.50%	2.92%		72.20%	2.79%	
**Males Precision**	83.76%	3.75%		76.44%	4.52%		81.42%	4.09%		85.53%	4.25%	
**Males Recall**	76.29%	4.82%		66.76%	4.80%		79.26%	5.04%		55.68%	4.83%	
**Males FNR**	23.71	4.82		33.24	4.80		20.74	5.04		44.32	4.83	
**Males TNR**	82.42	3.99		75.18	4.77		77.73	5.85		88.71	3.66	
**Males FPR**	17.58	3.99		24.82	4.77		22.27	5.85		11.29	3.66	
**Males TPR**	76.29	4.82		66.76	4.80		79.26	5.04		55.68	4.83	
	**Random Forest Results**			**Logistic Regression Results**			**Support Vector Machine Results**			**Gaussian Naive Bayes Results**		
**Sex Performance Disparities (Male Mean - Female Mean, n=100)**	Sex Performance Disparities (Male Mean - Female Mean, n=100)		**T Test P Value**	Sex Performance Disparities (Male Mean - Female Mean, n=100)		**T Test P Value**	Sex Performance Disparities (Male Mean - Female Mean, n=100)		**T Test P Value**	Sex Performance Disparities (Male Mean - Female Mean, n=100)		**T Test P Value**
**Accuacy**	2.96		0.00	-2.85		0.01	-2.98		0.02	-2.72		0.02
**FScore**	15.63		0.00	15.86		0.00	4.14		0.00	16.19		0.00
**ROC_AUC**	6.80%		0.00	2.93%		0.00	-2.41%		0.08	5.53%		0.00
**Precision**	5.25%		0.00	-4.87%		0.00	3.41%		0.00	-3.13%		0.05
**Recall**	21.02%		0.00	24.07%		0.00	2.58%		0.04	19.31%		0.00
**False Negative Rate**	-21.02		0.00	-24.07		0.00	-2.58		0.08	-19.31		0.00
**True Negative Rate**	-7.42		0.00	-18.20		0.00	-7.40		0.00	-8.24		0.00
**False Positive Rate**	7.42		0.00	18.20		0.00	7.40		0.00	8.24		0.00
**True Positive Rate**	21.02		0.00	24.07		0.00	2.58		0.04	19.31		0.00

Experiment 3.1.1 (Unbalanced training data, no feature selection) - Random Forest Classifier

	UB_Accuracy	UB_FScore	UB_ROC_AUC	UB_Precision	UB_Recall	UB_F_Accuracy
0	78.0	73.93364928909950	0.7722523219814240	0.8041237113402060	0.6842105263157900	78.57142857142860
1	78.0	75.55555555555550	0.777218782249742	0.7657657657657660	0.7456140350877190	87.14285714285710
2	79.2	77.39130434782610	0.7953744634505730	0.8811881188118810	0.689922480620155	75.80645161290320
3	77.2	74.88986784140970	0.7702356557377050	0.8095238095238100	0.6967213114754100	75.0
4	76.4	75.31380753138080	0.7669871794871800	0.8256880733944960	0.6923076923076920	78.37837837837840
5	79.60000000000000	77.33333333333330	0.792910447761194	0.7981651376146790	0.75	77.02702702702700
6	76.8	75.0	0.7680000000000000	0.8130841121495330	0.696	77.14285714285720
7	79.60000000000000	79.01234567901240	0.8010731958100380	0.8727272727272730	0.7218045112781950	71.23287671232880
8	76.4	73.77777777777780	0.7625952243774410	0.8137254901960780	0.6747967479674800	73.07692307692310
9	76.4	73.54260089686100	0.768242680504837	0.8723404255319150	0.6356589147286820	71.01449275362320
10	78.8	77.25321888412020	0.7871135010562700	0.8181818181818180	0.7317073170731710	75.67567567567570
11	80.0	77.47747747747750	0.796062789500772	0.8113207547169810	0.7413793103448280	82.43243243243240
12	78.0	76.98744769874480	0.7809039114013190	0.8214285714285710	0.7244094488188980	80.26315789473690
13	78.4	77.50000000000000	0.7854123975409840	0.8303571428571430	0.7265625	74.64788732394370
14	81.6	79.82456140350880	0.8136506511001350	0.8348623853211010	0.7647058823529410	80.55555555555560
15	79.60000000000000	78.66108786610880	0.7956989247311830	0.8173913043478260	0.7580645161290320	77.21518987341770
16	78.0	74.17840375586860	0.7761904761904760	0.7314814814814820	0.7523809523809520	68.0
17	82.40000000000000	82.11382113821140	0.8274910118130460	0.8859649122807020	0.7651515151515150	80.0
18	78.0	75.33632286995520	0.7762675920570660	0.7924528301886790	0.717948717948718	77.41935483870970
19	79.60000000000000	79.6812749003984	0.8018575851393190	0.8695652173913040	0.7352941176470590	77.14285714285720
20	77.60000000000000	75.86206896551720	0.7801282051282050	0.8627450980392160	0.676923076923077	75.0
21	76.4	74.67811158798280	0.7669613684412840	0.8365384615384620	0.6744186046511630	77.77777777777780
22	76.4	74.8936170212766	0.7658811475409840	0.822429906542056	0.6875	79.71014492753620
23	77.60000000000000	75.65217391304350	0.7843326264378900	0.8969072164948450	0.6541353383458650	76.71232876712330
24	79.2	79.6875	0.7993389072525760	0.8717948717948720	0.7338129496402880	73.97260273972600
25	76.4	74.45887445887450	0.7654119454580370	0.8269230769230770	0.6771653543307090	77.14285714285720
26	75.2	73.72881355932200	0.7586916007968640	0.8446601941747570	0.6541353383458650	71.05263157894740
27	76.0	75.60975609756100	0.7641539746802900	0.8230088495575220	0.6992481203007520	73.01587301587300
28	78.8	75.79908675799090	0.7870583717357910	0.8736842105263160	0.6693548387096770	82.89473684210530
29	77.2	75.32467532467530	0.7742699795081970	0.8446601941747570	0.6796875	74.64788732394370
30	80.0	78.44827586206900	0.8000000000000000	0.8504672897196260	0.728	81.08108108108110
31	83.2	82.20338983050850	0.8311347336065570	0.8508771929824560	0.7950819672131150	84.93150684931510
32	82.0	81.78137651821860	0.8204020229178670	0.8416666666666670	0.7952755905511810	81.03448275862070
33	78.0	76.79324894514770	0.7843030341907760	0.8584905660377360	0.6946564885496180	76.81159420289860
34	79.60000000000000	78.83817427385890	0.7987179487179490	0.8558558558558560	0.7307692307692310	74.57627118644070
35	80.4	77.62557077625570	0.8001490699332430	0.7870370370370370	0.7657657657657660	72.6027397260274
36	78.4	78.22580645161290	0.7920676958852790	0.8738738738738740	0.708029197080292	68.57142857142860
37	81.2	80.65843621399180	0.813140368852459	0.8521739130434780	0.765625	76.11940298507460
38	72.40000000000000	70.12987012987010	0.7262423155737710	0.7864077669902910	0.6328125	68.11594202898550
39	74.8	69.26829268292680	0.7370860068701800	0.7553191489361700	0.6396396396396400	81.81818181818180
40	78.8	77.82426778242680	0.7880000000000000	0.8157894736842110	0.744	81.94444444444440
41	78.4	75.45454545454550	0.7794762641898870	0.7830188679245280	0.7280701754385970	74.24242424242430
42	77.60000000000000	77.60000000000000	0.7777920328436720	0.8151260504201680	0.7404580152671760	75.0
43	76.8	75.2136752136752	0.766737138830162	0.7787610619469030	0.7272727272727270	74.64788732394370
44	77.2	76.5432098765432	0.7717725409836070	0.768595041322314	0.7622950819672130	75.9493670886076
45	79.2	76.78571428571430	0.7912186379928320	0.86	0.6935483870967740	76.38888888888890
46	78.4	77.5	0.7897950003213160	0.8691588785046730	0.6992481203007520	74.24242424242430
47	78.4	77.8688524590164	0.7838581669226830	0.7916666666666670	0.7661290322580650	76.47058823529410
48	80.4	79.49790794979080	0.804	0.8333333333333330	0.76	78.26086956521740
49	81.6	79.46428571428570	0.8127251672671130	0.8240740740740740	0.7672413793103450	81.33333333333330
50	81.6	80.67226890756300	0.8155750849958300	0.8067226890756300	0.8067226890756300	79.71014492753620
51	73.6	69.44444444444450	0.7349590373783920	0.8152173913043480	0.6048387096774190	71.01449275362320
52	72.8	70.43478260869570	0.7323717948717950	0.81	0.6230769230769230	66.15384615384620
53	73.6	72.03389830508480	0.7370846936815830	0.7798165137614680	0.6692913385826770	82.25806451612900
54	77.2	75.74468085106380	0.7725294418842810	0.8165137614678900	0.7063492063492060	70.0
55	84.0	81.81818181818180	0.8357432041642570	0.8737864077669900	0.7692307692307690	89.70588235294120
56	78.8	78.18930041152260	0.7903846153846160	0.8407079646017700	0.7307692307692310	80.88235294117650
57	81.2	80.65843621399180	0.811935421871997	0.8032786885245900	0.8099173553719010	71.23287671232880
58	78.8	78.71485943775100	0.7945046439628480	0.8672566371681420	0.7205882352941180	79.71014492753620
59	74.4	73.10924369747900	0.7471153846153850	0.8055555555555560	0.6692307692307690	70.66666666666670
60	78.0	76.98744769874480	0.7915186357470450	0.9019607843137260	0.6715328467153280	80.88235294117650
61	76.0	72.47706422018350	0.7560897435897440	0.8061224489795920	0.6583333333333330	77.14285714285720
62	80.0	75.72815533980580	0.790422278612792	0.8041237113402060	0.7155963302752290	78.37837837837840
63	79.2	77.58620689655170	0.7914746543778800	0.8333333333333330	0.7258064516129030	72.85714285714290
64	79.2	78.33333333333330	0.7957534158701650	0.8623853211009180	0.7175572519083970	76.38888888888890
65	79.60000000000000	79.01234567901240	0.7957557134626470	0.8	0.7804878048780490	78.08219178082190
66	76.8	75.42372881355930	0.768	0.8018018018018020	0.712	70.83333333333330
67	78.0	73.93364928909950	0.7721400426329050	0.7959183673469390	0.6902654867256640	81.9672131147541
68	78.0	76.98744769874480	0.7849255264509500	0.8598130841121500	0.696969696969697	75.38461538461540
69	76.0	72.72727272727270	0.7610087045570920	0.851063829787234	0.6349206349206350	75.67567567567570
70	72.8	71.42857142857140	0.7319905061261150	0.794392523364486	0.648854961832061	66.66666666666670
71	75.2	73.72881355932200	0.7545646742264080	0.8130841121495330	0.6744186046511630	67.64705882352940
72	76.4	75.10548523206750	0.7636328725038400	0.7876106194690270	0.717741935483871	72.15189873417720
73	78.4	76.31578947368420	0.7888495807127880	0.7131147540983610	0.8207547169811320	75.32467532467530
74	82.8	82.5910931174089	0.8312788906009240	0.8869565217391300	0.7727272727272730	76.81159420289860
75	80.4	78.02690582959640	0.8009615384615390	0.8446601941747570	0.725	75.60975609756100
76	76.8	75.63025210084030	0.7690821256038650	0.7317073170731710	0.782608695652174	70.27027027027030
77	77.60000000000000	75.65217391304350	0.7739431650522800	0.7837837837837840	0.7310924369747900	79.1044776119403
78	77.60000000000000	76.66666666666670	0.7817620975515710	0.8598130841121500	0.6917293233082710	76.05633802816900
79	74.0	74.1035856573705	0.7489467885151340	0.8303571428571430	0.6690647482014390	75.64102564102560
80	78.4	77.11864406779660	0.7875	0.8584905660377360	0.7	81.15942028985510
81	78.0	76.79324894514770	0.7800000000000000	0.8125	0.728	71.01449275362320
82	76.0	73.45132743362830	0.76	0.8217821782178220	0.664	73.23943661971830
83	76.8	76.42276422764230	0.7713148433487420	0.8245614035087720	0.7121212121212120	76.71232876712330
84	76.0	76.37795275590550	0.7628023674729800	0.8083333333333330	0.7238805970149250	73.52941176470590
85	81.2	78.13953488372090	0.8066897791474060	0.865979381443299	0.711864406779661	74.32432432432430
86	78.8	75.34883720930230	0.7819737519300050	0.8181818181818180	0.6982758620689660	74.35897435897440
87	80.80000000000000	78.57142857142860	0.805968237704918	0.8627450980392160	0.7213114754098360	80.59701492537310
88	78.4	76.92307692307690	0.7840000000000000	0.8256880733944960	0.72	72.22222222222220
89	78.0	77.17842323651450	0.7826923076923080	0.8378378378378380	0.7153846153846150	72.0
90	75.2	73.50427350427350	0.7635448916408670	0.8775510204081630	0.6323529411764710	69.84126984126980
91	79.2	77.58620689655170	0.7910505089302860	0.8256880733944960	0.7317073170731710	76.31578947368420
92	77.60000000000000	76.27118644067800	0.7794871794871800	0.8490566037735850	0.6923076923076920	72.0
93	80.80000000000000	80.16528925619840	0.8083077316948280	0.8362068965517240	0.7698412698412700	79.45205479452060
94	78.8	77.63713080168780	0.7871420334422450	0.7931034482758620	0.7603305785123970	77.27272727272730
95	76.4	75.31380753138080	0.7636968766001020	0.782608695652174	0.7258064516129030	76.38888888888890
96	77.2	75.9493670886076	0.7711256326478310	0.7758620689655170	0.743801652892562	76.0
97	75.6	71.889400921659	0.7583061263683500	0.8666666666666670	0.6141732283464570	71.05263157894740
98	82.40000000000000	82.67716535433070	0.8256933744221880	0.860655737704918	0.7954545454545450	82.89473684210530
99	82.8	82.73092369477910	0.8280849974398360	0.8373983739837400	0.8174603174603170	77.94117647058820
**Mean**	**78.2**	**76.57499511468320**	**78.2540699822069000%**	**82.6461387935722000%**	**71.5804918406232000%**	**76.0634288588317**
**Standard Deviation**	2.4	2.86283545620196	2.29549229392733300%	3.68683461739709900%	4.68667170226994400%	4.40477428676004
**T Test Significance (Female to Male)**						**0.0000505176687049689**

	UB_F_FScore	UB_F_ROC_AUC	UB_F_Precision	UB_F_Recall	UB_F_FNR	UB_F_TNR	UB_F_FPR	UB_F_TPR
0	65.38461538461540	0.737037037037037	0.7727272727272730	0.5666666666666670	43.333333333333300	90.74074074074080	9.25925925925926	56.666666666666700
1	79.06976744186050	0.8376503237742830	0.85	0.7391304347826090	26.08695652173910	93.61702127659580	6.382978723404260	73.91304347826090
2	59.45945945945950	0.7028508771929820	0.8461538461538460	0.4583333333333330	54.166666666666700	94.73684210526320	5.263157894736840	45.83333333333330
3	57.77777777777780	0.6922230374174620	0.8125	0.4482758620689660	55.172413793103400	93.61702127659580	6.382978723404260	44.827586206896600
4	65.21739130434780	0.7419354838709680	1.0	0.4838709677419360	51.61290322580650	100.0	0.0	48.38709677419360
5	60.465116279069800	0.7185314685314680	0.6190476190476190	0.5909090909090910	40.909090909090900	84.61538461538460	15.384615384615400	59.09090909090910
6	55.55555555555560	0.6856060606060610	0.7142857142857140	0.45454545454545500	54.54545454545450	91.66666666666670	8.333333333333330	45.45454545454550
7	58.82352941176470	0.694947209653092	0.8823529411764710	0.4411764705882350	55.88235294117650	94.87179487179490	5.128205128205130	44.11764705882350
8	57.14285714285710	0.6801548205489090	0.7	0.4827586206896550	51.724137931034500	87.75510204081630	12.244897959183700	48.275862068965500
9	58.33333333333330	0.6982323232323230	0.9333333333333330	0.42424242424242400	57.57575757575760	97.22222222222220	2.7777777777777800	42.42424242424240
10	65.38461538461540	0.7265151515151520	0.7727272727272730	0.5666666666666670	43.333333333333300	88.63636363636360	11.363636363636400	56.666666666666700
11	72.3404255319149	0.7889795918367350	0.7727272727272730	0.68	32.0	89.79591836734690	10.204081632653100	68.0
12	72.72727272727270	0.7789855072463770	0.8	0.6666666666666670	33.33333333333330	89.13043478260870	10.869565217391300	66.66666666666670
13	57.14285714285710	0.6856521739130440	0.7058823529411770	0.48	52.0	89.13043478260870	10.869565217391300	48.0
14	70.83333333333330	0.7741935483870970	1.0	0.5483870967741940	45.16129032258060	100.0	0.0	54.83870967741940
15	70.0	0.7532679738562090	0.8076923076923080	0.6176470588235290	38.23529411764710	88.88888888888890	11.11111111111110	61.76470588235290
16	58.620689655172400	0.6626686656671660	0.5862068965517240	0.5862068965517240	41.37931034482760	73.91304347826090	26.08695652173910	58.620689655172400
17	68.42105263157900	0.7555816686251470	0.8666666666666670	0.5652173913043480	43.47826086956520	94.5945945945946	5.405405405405410	56.52173913043480
18	56.25	0.6903304773561810	0.6923076923076920	0.47368421052631600	52.63157894736840	90.69767441860470	9.30232558139535	47.368421052631600
19	63.63636363636360	0.7237762237762240	0.7777777777777780	0.5384615384615380	46.15384615384620	90.9090909090909	9.090909090909090	53.84615384615390
20	63.82978723404260	0.7178571428571430	0.7894736842105260	0.5357142857142860	46.42857142857140	90.0	10.0	53.57142857142860
21	63.1578947368421	0.7243589743589740	0.8571428571428570	0.5	50.0	94.87179487179490	5.128205128205130	50.0
22	70.83333333333330	0.76698606271777	0.85	0.6071428571428570	39.285714285714300	92.6829268292683	7.317073170731710	60.71428571428570
23	63.82978723404260	0.7234126984126980	0.7894736842105260	0.5357142857142860	46.42857142857140	91.11111111111110	8.88888888888889	53.57142857142860
24	65.45454545454550	0.7227272727272730	0.8181818181818180	0.5454545454545450	45.45454545454550	90.0	10.0	54.54545454545450
25	68.0	0.7485525227460710	0.8947368421052630	0.5483870967741940	45.16129032258060	94.87179487179490	5.128205128205130	54.83870967741940
26	50.0	0.6507246376811590	0.7857142857142860	0.36666666666666700	63.33333333333330	93.47826086956520	6.521739130434780	36.666666666666700
27	62.22222222222220	0.7037037037037040	0.7777777777777780	0.5185185185185190	48.148148148148100	88.88888888888890	11.11111111111110	51.85185185185190
28	74.50980392156860	0.7949275362318840	0.9047619047619050	0.6333333333333330	36.666666666666700	95.65217391304350	4.3478260869565200	63.33333333333330
29	60.8695652173913	0.7025641025641030	0.7	0.5384615384615380	46.15384615384620	86.66666666666670	13.333333333333300	53.84615384615390
30	66.66666666666670	0.7483974358974360	0.875	0.5384615384615380	46.15384615384620	95.83333333333330	4.166666666666670	53.84615384615390
31	75.55555555555560	0.8087500000000000	0.85	0.68	32.0	93.75	6.25	68.0
32	70.27027027027030	0.7676767676767680	0.8666666666666670	0.5909090909090910	40.909090909090900	94.44444444444440	5.555555555555560	59.09090909090910
33	68.0	0.7431034482758620	0.8095238095238100	0.5862068965517240	41.37931034482760	90.0	10.0	58.620689655172400
34	65.11627906976740	0.7237762237762240	0.8235294117647060	0.5384615384615380	46.15384615384620	90.9090909090909	9.090909090909090	53.84615384615390
35	56.52173913043480	0.6789965986394560	0.5909090909090910	0.5416666666666670	45.83333333333330	81.63265306122450	18.367346938775500	54.166666666666700
36	52.17391304347830	0.6550868486352360	0.8	0.3870967741935480	61.29032258064520	92.3076923076923	7.6923076923076900	38.70967741935480
37	65.21739130434780	0.7285714285714290	0.7142857142857140	0.6	40.0	85.71428571428570	14.285714285714300	60.0
38	47.61904761904760	0.6204545454545460	0.5882352941176470	0.4	60.0	84.0909090909091	15.909090909090900	40.0
39	61.111111111111100	0.7206119162640900	0.8461538461538460	0.4782608695652170	52.17391304347830	96.29629629629630	3.7037037037037000	47.82608695652170
40	69.76744186046510	0.766722408026756	0.8823529411764710	0.5769230769230770	42.30769230769230	95.65217391304350	4.3478260869565200	57.692307692307700
41	65.3061224489796	0.7201923076923080	0.6956521739130440	0.6153846153846150	38.46153846153850	82.5	17.5	61.53846153846150
42	68.0	0.7337337337337340	0.7391304347826090	0.6296296296296300	37.03703703703700	83.78378378378380	16.216216216216200	62.96296296296300
43	62.5	0.7167553191489360	0.625	0.625	37.5	80.85106382978720	19.148936170212800	62.5
44	72.46376811594200	0.7548701298701300	0.7352941176470590	0.7142857142857140	28.57142857142860	79.54545454545460	20.454545454545500	71.42857142857140
45	63.829787234042600	0.7237369687249400	0.8333333333333330	0.5172413793103450	48.275862068965500	93.02325581395350	6.976744186046510	51.724137931034500
46	62.22222222222220	0.7143522833178010	0.875	0.4827586206896550	51.724137931034500	94.5945945945946	5.405405405405410	48.275862068965500
47	66.66666666666670	0.7357142857142860	0.8	0.5714285714285710	42.857142857142900	90.0	10.0	57.14285714285710
48	70.58823529411770	0.7604529616724740	0.782608695652174	0.6428571428571430	35.714285714285700	87.8048780487805	12.195121951219500	64.28571428571430
49	73.07692307692310	0.7833333333333330	0.8636363636363640	0.6333333333333330	36.666666666666700	93.33333333333330	6.666666666666670	63.33333333333330
50	69.56521739130430	0.7785299806576400	0.6666666666666670	0.7272727272727270	27.27272727272730	82.97872340425530	17.02127659574470	72.72727272727270
51	41.1764705882353	0.6086956521739130	0.6363636363636360	0.30434782608695700	69.56521739130430	91.30434782608700	8.695652173913040	30.434782608695700
52	47.61904761904760	0.646584440227704	0.9090909090909090	0.3225806451612900	67.74193548387100	97.05882352941180	2.941176470588240	32.25806451612900
53	76.59574468085110	0.8044871794871790	0.8571428571428570	0.6923076923076920	30.76923076923080	91.66666666666670	8.333333333333330	69.23076923076920
54	57.14285714285720	0.6679561573178590	0.6956521739130440	0.48484848484848500	51.515151515151500	85.1063829787234	14.893617021276600	48.484848484848500
55	82.05128205128200	0.880236305048335	0.8	0.8421052631578950	15.789473684210500	91.83673469387760	8.16326530612245	84.21052631578950
56	71.11111111111110	0.7732142857142860	0.9411764705882350	0.5714285714285710	42.857142857142900	97.5	2.5	57.14285714285710
57	57.14285714285710	0.679421768707483	0.56	0.5833333333333330	41.66666666666670	77.55102040816330	22.448979591836700	58.333333333333300
58	73.07692307692310	0.7833614864864870	0.95	0.59375	40.625	97.2972972972973	2.7027027027027000	59.375
59	62.06896551724140	0.6888888888888890	0.6428571428571430	0.6	40.0	77.77777777777780	22.22222222222220	60.0
60	75.47169811320750	0.7955536181342630	0.9090909090909090	0.6451612903225810	35.483870967741900	94.5945945945946	5.405405405405410	64.51612903225810
61	66.66666666666670	0.7381567614125750	0.7619047619047620	0.5925925925925930	40.74074074074070	88.37209302325580	11.627906976744200	59.25925925925930
62	63.63636363636360	0.7289795918367350	0.7368421052631580	0.56	44.0	89.79591836734690	10.204081632653100	56.00000000000000
63	59.57446808510640	0.6894918173987940	0.7	0.5185185185185190	48.148148148148100	86.04651162790700	13.953488372093000	51.85185185185190
64	67.9245283018868	0.751547987616099	0.9473684210526320	0.5294117647058820	47.05882352941180	97.36842105263160	2.631578947368420	52.94117647058820
65	72.41379310344830	0.7703619909502260	0.875	0.6176470588235290	38.23529411764710	92.3076923076923	7.6923076923076900	61.76470588235290
66	60.37735849056600	0.6828388131515640	0.6666666666666670	0.5517241379310350	44.827586206896600	81.3953488372093	18.6046511627907	55.172413793103400
67	62.06896551724140	0.725	1.0	0.45	55.00000000000000	100.0	0.0	45.0
68	70.37037037037040	0.7566287878787880	0.9047619047619050	0.5757575757575760	42.42424242424240	93.75	6.25	57.57575757575760
69	64.00000000000000	0.7231807951988000	0.8421052631578950	0.5161290322580650	48.38709677419360	93.02325581395350	6.976744186046510	51.61290322580650
70	44.44444444444450	0.6071964017991000	0.625	0.3448275862068970	65.51724137931040	86.95652173913040	13.043478260869600	34.48275862068970
71	56.00000000000000	0.6495031616982840	0.6086956521739130	0.5185185185185190	48.148148148148100	78.04878048780490	21.951219512195100	51.85185185185190
72	56.00000000000000	0.6715686274509800	0.6363636363636360	0.5	50.0	84.31372549019610	15.686274509803900	50.0
73	62.7450980392157	0.7295597484276730	0.5925925925925930	0.6666666666666670	33.33333333333330	79.24528301886790	20.754716981132100	66.66666666666670
74	65.21739130434780	0.7347222222222220	0.6818181818181820	0.625	37.5	84.44444444444440	15.555555555555600	62.5
75	66.66666666666670	0.736936936936937	0.8695652173913040	0.5405405405405410	45.94594594594600	93.33333333333330	6.666666666666670	54.054054054054100
76	56.00000000000000	0.6650641025641030	0.5833333333333330	0.5384615384615380	46.15384615384620	79.16666666666670	20.833333333333300	53.84615384615390
77	73.07692307692310	0.7825892857142860	0.95	0.59375	40.625	97.14285714285710	2.857142857142860	59.375
78	71.1864406779661	0.7583333333333330	0.875	0.6	40.0	91.66666666666670	8.333333333333330	60.0
79	64.15094339622640	0.7208333333333330	0.7391304347826090	0.5666666666666670	43.333333333333300	87.5	12.5	56.666666666666700
80	71.1111111111111	0.7724867724867720	0.8888888888888890	0.5925925925925930	40.74074074074070	95.23809523809520	4.761904761904760	59.25925925925930
81	60.0	0.6825396825396830	0.6521739130434780	0.5555555555555560	44.44444444444440	80.95238095238100	19.047619047619000	55.55555555555560
82	53.658536585365800	0.6696127946127950	0.7857142857142860	0.4074074074074070	59.25925925925930	93.18181818181820	6.8181818181818200	40.74074074074070
83	63.82978723404260	0.7234126984126980	0.7894736842105260	0.5357142857142860	46.42857142857140	91.11111111111110	8.88888888888889	53.57142857142860
84	66.66666666666670	0.7256944444444440	0.8181818181818180	0.5625	43.75	88.88888888888890	11.11111111111110	56.25
85	61.224489795918400	0.7045454545454550	0.7894736842105260	0.5	50.0	90.9090909090909	9.090909090909090	50.0
86	58.333333333333300	0.6900000000000000	0.7	0.5	50.0	88.0	12.0	50.0
87	66.66666666666670	0.747577519379845	0.8666666666666670	0.5416666666666670	45.83333333333330	95.34883720930230	4.651162790697680	54.166666666666700
88	66.66666666666670	0.7151702786377710	0.7692307692307690	0.5882352941176470	41.17647058823530	84.21052631578950	15.789473684210500	58.82352941176470
89	61.81818181818180	0.7053571428571430	0.85	0.4857142857142860	51.42857142857140	92.5	7.5	48.57142857142860
90	51.28205128205130	0.6724137931034480	1.0	0.3448275862068970	65.51724137931040	100.0	0.0	34.48275862068970
91	64.00000000000000	0.7247311827956990	0.8421052631578950	0.5161290322580650	48.38709677419360	93.33333333333330	6.666666666666670	51.61290322580650
92	57.14285714285710	0.6803519061583580	0.7777777777777780	0.45161290322580600	54.83870967741940	90.9090909090909	9.090909090909090	45.16129032258060
93	72.72727272727270	0.7766457680250790	0.7692307692307690	0.6896551724137930	31.03448275862070	86.36363636363640	13.636363636363600	68.96551724137930
94	63.41463414634150	0.7272727272727270	0.6842105263157900	0.5909090909090910	40.909090909090900	86.36363636363640	13.636363636363600	59.09090909090910
95	67.9245283018868	0.7444444444444440	0.6923076923076920	0.6666666666666670	33.33333333333330	82.22222222222220	17.77777777777780	66.66666666666670
96	66.66666666666670	0.7395833333333330	0.6666666666666670	0.6666666666666670	33.33333333333330	81.25	18.75	66.66666666666670
97	57.692307692307700	0.6775568181818180	0.75	0.46875	53.125	88.63636363636360	11.363636363636400	46.875
98	79.36507936507940	0.8224431818181820	0.8064516129032260	0.78125	21.875	86.36363636363640	13.636363636363600	78.125
99	65.11627906976740	0.7334883720930230	0.7777777777777780	0.56	44.0	90.69767441860470	9.30232558139535	56.00000000000000
**Mean**	**64.08815805662320**	**72.554974999381%**	**78.5164844470442%**	**55.271346675458%**	**44.728653324542**	**89.8386033233041**	**10.1613966766959**	**55.271346675458**
**Standard Deviation**	7.49573870099384	4.76113569455828%	10.6310474232942%	9.49233826556301%	9.49233826556302	5.69158776925875	5.69158776925875	9.49233826556301
**T Test Significance (Female to Male)**	**0.0000000000000000**	**0.00000000000001589464**	**0.00116835993316475%**	**0.0000000000000000**	**0.0000000000000000**	**0.0000000086801420**	**0.0000000000000000**	**0.0000000000000000**

	UB_M_Accuracy	UB_M_FScore	UB_M_ROC_AUC	UB_M_Precision	UB_M_Recall	UB_M_FNR	UB_M_TNR	UB_M_FPR	UB_M_TPR
0	77.71084337349400	76.72955974842770	0.7777293844367020	0.8133333333333330	0.7261904761904760	27.380952380952400	82.92682926829270	17.073170731707300	72.61904761904760
1	74.44444444444440	74.72527472527470	0.7444128904803060	0.7472527472527470	0.7472527472527470	25.274725274725300	74.15730337078650	25.842696629213500	74.72527472527470
2	80.31914893617020	80.82901554404150	0.8111876075731500	0.8863636363636360	0.7428571428571430	25.71428571428570	87.95180722891570	12.048192771084300	74.28571428571430
3	78.16091954022990	79.12087912087910	0.78215850258861	0.8089887640449440	0.7741935483870970	22.58064516129030	79.01234567901230	20.98765432098770	77.41935483870970
4	75.56818181818180	77.720207253886	0.7554112554112550	0.7978723404255320	0.7575757575757580	24.242424242424200	75.32467532467530	24.675324675324700	75.75757575757580
5	80.68181818181820	81.31868131868130	0.8082511676180590	0.8409090909090910	0.7872340425531920	21.27659574468090	82.92682926829270	17.073170731707300	78.72340425531920
6	76.66666666666670	78.57142857142860	0.7698903038708860	0.8279569892473120	0.7475728155339810	25.24271844660190	79.22077922077920	20.77922077922080	74.75728155339810
7	83.05084745762710	84.375	0.8321678321678320	0.8709677419354840	0.8181818181818180	18.181818181818200	84.61538461538460	15.384615384615400	81.81818181818180
8	77.90697674418610	78.4090909090909	0.7836879432624110	0.8414634146341460	0.7340425531914890	26.595744680851100	83.33333333333330	16.666666666666700	73.40425531914890
9	78.45303867403320	77.71428571428570	0.7894607843137260	0.8607594936708860	0.7083333333333330	29.166666666666700	87.05882352941180	12.941176470588200	70.83333333333330
10	80.11363636363640	80.66298342541440	0.8021116724964370	0.8295454545454550	0.7849462365591400	21.50537634408600	81.92771084337350	18.072289156626500	78.49462365591400
11	78.97727272727270	78.85714285714290	0.790885585003232	0.8214285714285710	0.7582417582417580	24.175824175824200	82.35294117647060	17.647058823529400	75.82417582417580
12	77.01149425287360	78.26086956521740	0.7737314232159590	0.8275862068965520	0.7422680412371130	25.773195876288700	80.51948051948050	19.480519480519500	74.22680412371140
13	79.88826815642460	81.81818181818180	0.8010986203372510	0.8526315789473680	0.7864077669902910	21.35922330097090	81.57894736842110	18.421052631578900	78.64077669902910
14	82.02247191011240	82.22222222222220	0.8204545454545450	0.8043478260869570	0.8409090909090910	15.909090909090900	80.0	20.0	84.0909090909091
15	80.7017543859649	81.56424581005590	0.80679012345679	0.8202247191011240	0.8111111111111110	18.88888888888890	80.24691358024690	19.753086419753100	81.11111111111110
16	82.28571428571430	80.0	0.8220361509835200	0.7848101265822780	0.8157894736842110	18.421052631578900	82.82828282828280	17.17171717171720	81.57894736842110
17	83.15789473684210	84.61538461538460	0.8357684902027410	0.8888888888888890	0.8073394495412840	19.26605504587160	86.41975308641980	13.580246913580200	80.73394495412850
18	78.19148936170210	78.53403141361260	0.7826530612244900	0.8064516129032260	0.7653061224489800	23.46938775510200	80.0	20.0	76.53061224489800
19	80.55555555555560	83.09178743961350	0.8123376623376620	0.8865979381443300	0.7818181818181820	21.818181818181800	84.28571428571430	15.714285714285700	78.18181818181820
20	78.57142857142860	78.91891891891890	0.795343137254902	0.8795180722891570	0.7156862745098040	28.431372549019600	87.5	12.5	71.56862745098040
21	75.93582887700540	76.92307692307690	0.7656794425087110	0.8333333333333330	0.7142857142857140	28.57142857142860	81.70731707317070	18.29268292682930	71.42857142857140
22	75.13812154696130	75.93582887700540	0.7562345679012350	0.8160919540229890	0.71	29.000000000000000	80.24691358024690	19.753086419753100	71.0
23	77.96610169491530	78.68852459016400	0.8011904761904760	0.9230769230769230	0.6857142857142860	31.428571428571400	91.66666666666670	8.333333333333330	68.57142857142860
24	81.35593220338980	83.58208955223880	0.8187616263619450	0.8842105263157890	0.7924528301886790	20.754716981132100	84.50704225352110	15.492957746478900	79.24528301886790
25	76.11111111111110	76.24309392265190	0.7641369047619050	0.8117647058823530	0.71875	28.125	80.95238095238100	19.047619047619000	71.875
26	77.01149425287360	79.16666666666670	0.7773827430603040	0.8539325842696630	0.7378640776699030	26.21359223300970	81.69014084507040	18.30985915492960	73.7864077669903
27	77.00534759358290	78.60696517412930	0.7738760773351970	0.8315789473684210	0.7452830188679250	25.471698113207500	80.24691358024690	19.753086419753100	74.52830188679250
28	77.01149425287360	76.19047619047620	0.7779255319148940	0.8648648648648650	0.6808510638297870	31.914893617021300	87.5	12.5	68.08510638297870
29	78.2122905027933	78.91891891891890	0.7929080723198370	0.8795180722891570	0.7156862745098040	28.431372549019600	87.01298701298700	12.987012987013000	71.56862745098040
30	79.54545454545460	81.05263157894740	0.7979797979797980	0.8461538461538460	0.7777777777777780	22.22222222222220	81.81818181818180	18.181818181818200	77.77777777777780
31	82.48587570621470	83.76963350785340	0.8248711340206190	0.851063829787234	0.8247422680412370	17.525773195876300	82.5	17.5	82.4742268041237
32	82.29166666666670	83.80952380952380	0.8213464696223320	0.8380952380952380	0.8380952380952380	16.19047619047620	80.45977011494250	19.54022988505750	83.80952380952380
33	78.45303867403320	79.14438502673800	0.7931248448746590	0.8705882352941180	0.7254901960784310	27.450980392156900	86.07594936708860	13.924050632911400	72.54901960784310
34	81.15183246073300	81.81818181818180	0.814710433244916	0.8617021276595740	0.7788461538461540	22.115384615384600	85.0574712643678	14.942528735632200	77.88461538461540
35	83.61581920903950	83.23699421965320	0.8360153256704980	0.8372093023255810	0.8275862068965520	17.24137931034480	84.44444444444440	15.555555555555600	82.75862068965520
36	82.22222222222220	84.15841584158420	0.8266190719020910	0.8854166666666670	0.8018867924528300	19.81132075471700	85.13513513513510	14.864864864864900	80.18867924528300
37	83.06010928961750	84.26395939086300	0.8341626213592230	0.8829787234042550	0.8058252427184470	19.41747572815530	86.25	13.750000000000000	80.58252427184470
38	74.03314917127070	75.13227513227510	0.7485063480209110	0.8255813953488370	0.6893203883495150	31.06796116504850	80.76923076923080	19.230769230769200	68.93203883495150
39	71.67630057803470	71.0059171597633	0.7173796791443850	0.7407407407407410	0.6818181818181820	31.818181818181800	75.29411764705880	24.705882352941200	68.18181818181820
40	77.52808988764050	79.59183673469390	0.773686229382432	0.8041237113402060	0.7878787878787880	21.21212121212120	75.9493670886076	24.050632911392400	78.78787878787880
41	79.8913043478261	78.3625730994152	0.7973484848484850	0.8072289156626510	0.7613636363636360	23.863636363636400	83.33333333333330	16.666666666666700	76.13636363636360
42	78.49462365591400	80.0	0.7870544090056290	0.8333333333333330	0.7692307692307690	23.076923076923100	80.48780487804880	19.51219512195120	76.92307692307690
43	77.6536312849162	78.49462365591400	0.7787276841840580	0.8202247191011240	0.7525773195876290	24.742268041237100	80.48780487804880	19.51219512195120	75.25773195876290
44	77.77777777777780	78.1609195402299	0.7777093596059110	0.7816091954022990	0.7816091954022990	21.839080459770100	77.38095238095240	22.61904761904760	78.16091954022990
45	80.33707865168540	80.22598870056500	0.8074191502853520	0.8658536585365850	0.7473684210526320	25.263157894736800	86.74698795180720	13.253012048192800	74.73684210526320
46	79.8913043478261	81.02564102564100	0.8048076923076920	0.8681318681318680	0.7596153846153850	24.03846153846150	85.0	15.0	75.96153846153850
47	79.12087912087910	80.6122448979592	0.7893653100775190	0.79	0.8229166666666670	17.708333333333300	75.5813953488372	24.418604651162800	82.29166666666670
48	81.21546961325970	81.91489361702130	0.813573883161512	0.8461538461538460	0.7938144329896910	20.618556701030900	83.33333333333330	16.666666666666700	79.38144329896910
49	81.71428571428570	81.39534883720930	0.8170891037366080	0.813953488372093	0.813953488372093	18.6046511627907	82.02247191011240	17.97752808988760	81.3953488372093
50	82.32044198895030	83.33333333333330	0.8230854197349040	0.8421052631578950	0.8247422680412370	17.525773195876300	82.14285714285710	17.857142857142900	82.4742268041237
51	74.58563535911600	74.72527472527470	0.7553836633663370	0.8395061728395060	0.6732673267326730	32.67326732673270	83.75	16.25	67.32673267326730
52	75.13513513513510	75.53191489361700	0.7539346957951610	0.797752808988764	0.7171717171717170	28.28282828282830	79.06976744186050	20.930232558139500	71.71717171717170
53	70.74468085106380	70.8994708994709	0.7109935131444180	0.7613636363636360	0.6633663366336630	33.663366336633700	75.86206896551720	24.137931034482800	66.33663366336630
54	80.58823529411770	81.56424581005590	0.8080575338639860	0.8488372093023260	0.7849462365591400	21.50537634408600	83.11688311688310	16.883116883116900	78.49462365591400
55	81.86813186813190	81.76795580110500	0.8239795918367350	0.891566265060241	0.7551020408163270	24.489795918367300	89.28571428571430	10.714285714285700	75.51020408163270
56	78.02197802197800	79.7979797979798	0.7810049019607840	0.8229166666666670	0.7745098039215690	22.54901960784310	78.75	21.25	77.45098039215690
57	85.31073446327680	86.59793814432990	0.8517396907216500	0.865979381443299	0.865979381443299	13.402061855670100	83.75	16.25	86.5979381443299
58	78.45303867403320	80.20304568527920	0.7888986013986010	0.8494623655913980	0.7596153846153850	24.03846153846150	81.81818181818180	18.181818181818200	75.96153846153850
59	76.0	76.66666666666670	0.7716666666666670	0.8625	0.69	31.0	85.33333333333330	14.666666666666700	69.0
60	76.92307692307690	77.41935483870970	0.7869910625620650	0.9	0.6792452830188680	32.075471698113200	89.47368421052630	10.526315789473700	67.9245283018868
61	75.55555555555560	74.11764705882350	0.7582499073044120	0.8181818181818180	0.6774193548387100	32.25806451612900	83.9080459770115	16.091954022988500	67.74193548387100
62	80.68181818181820	79.01234567901230	0.8048654244306420	0.8205128205128210	0.7619047619047620	23.809523809523800	84.78260869565220	15.217391304347800	76.19047619047620
63	81.66666666666670	82.16216216216220	0.8194634206930820	0.8636363636363640	0.7835051546391750	21.649484536082500	85.54216867469880	14.457831325301200	78.35051546391750
64	80.33707865168540	81.28342245989310	0.8053328242331680	0.8444444444444440	0.7835051546391750	21.649484536082500	82.71604938271610	17.28395061728400	78.35051546391750
65	80.22598870056500	81.08108108108110	0.8020301327885600	0.78125	0.8426966292134830	15.730337078651700	76.13636363636360	23.863636363636400	84.26966292134830
66	79.21348314606740	79.78142076502730	0.7948424796747970	0.8390804597701150	0.7604166666666670	23.958333333333300	82.92682926829270	17.073170731707300	76.04166666666670
67	76.71957671957670	75.82417582417580	0.7668010752688170	0.7752808988764050	0.7419354838709680	25.806451612903200	79.16666666666670	20.833333333333300	74.19354838709680
68	78.91891891891890	78.91891891891890	0.7931054733380320	0.8488372093023260	0.7373737373737370	26.262626262626300	84.88372093023260	15.11627906976740	73.73737373737370
69	76.13636363636360	75.29411764705880	0.7689408706952570	0.8533333333333330	0.6736842105263160	32.631578947368400	86.41975308641980	13.580246913580200	67.36842105263160
70	75.42857142857140	77.72020725388600	0.758058017727639	0.8241758241758240	0.7352941176470590	26.47058823529410	78.08219178082190	21.91780821917810	73.52941176470590
71	78.02197802197800	78.49462365591400	0.789093137254902	0.8690476190476190	0.7156862745098040	28.431372549019600	86.25	13.750000000000000	71.56862745098040
72	78.3625730994152	80.21390374331550	0.7839583333333330	0.8241758241758240	0.78125	21.875	78.66666666666670	21.333333333333300	78.125
73	79.76878612716760	80.22598870056500	0.8010586974001610	0.7473684210526320	0.8658536585365850	13.414634146341500	73.62637362637360	26.373626373626400	86.58536585365850
74	85.0828729281768	86.56716417910450	0.8616818873668190	0.9354838709677420	0.8055555555555560	19.444444444444400	91.78082191780820	8.21917808219178	80.55555555555560
75	82.73809523809520	82.20858895705520	0.8271438695960310	0.8375	0.8072289156626510	19.27710843373490	84.70588235294120	15.294117647058800	80.72289156626510
76	79.54545454545460	80.85106382978720	0.7947823840888540	0.7676767676767680	0.8539325842696630	14.606741573033700	73.5632183908046	26.436781609195400	85.39325842696630
77	77.04918032786890	76.40449438202250	0.7710129310344830	0.7472527472527470	0.7816091954022990	21.839080459770100	76.04166666666670	23.958333333333300	78.16091954022990
78	78.2122905027933	78.45303867403320	0.7881708238851100	0.8554216867469880	0.7244897959183670	27.55102040816330	85.18518518518520	14.814814814814800	72.44897959183670
79	73.25581395348840	76.76767676767680	0.745449250036406	0.8539325842696630	0.6972477064220180	30.275229357798200	79.36507936507940	20.634920634920600	69.72477064220180
80	77.34806629834250	78.53403141361260	0.7807443365695790	0.8522727272727270	0.7281553398058250	27.184466019417500	83.33333333333330	16.666666666666700	72.81553398058250
81	80.66298342541440	81.28342245989310	0.8094418490287680	0.8539325842696630	0.7755102040816330	22.448979591836700	84.33734939759040	15.66265060240960	77.55102040816330
82	77.09497206703910	77.83783783783780	0.7747543461829180	0.8275862068965520	0.7346938775510200	26.53061224489800	81.48148148148150	18.51851851851850	73.46938775510210
83	76.8361581920904	79.39698492462310	0.7702186512118020	0.8315789473684210	0.7596153846153850	24.03846153846150	78.08219178082190	21.91780821917810	75.96153846153850
84	76.92307692307690	79.0	0.7685049019607840	0.8061224489795920	0.7745098039215690	22.54901960784310	76.25	23.75	77.45098039215690
85	84.0909090909091	83.13253012048190	0.8409090909090910	0.8846153846153850	0.7840909090909090	21.59090909090910	89.77272727272730	10.227272727272700	78.4090909090909
86	80.81395348837210	80.23952095808380	0.8092532467532470	0.8481012658227850	0.7613636363636360	23.863636363636400	85.71428571428570	14.285714285714300	76.13636363636360
87	80.87431693989070	81.08108108108110	0.8120648259303720	0.8620689655172410	0.7653061224489800	23.46938775510200	85.88235294117650	14.117647058823500	76.53061224489800
88	80.89887640449440	80.45977011494250	0.8099027409372240	0.8433734939759040	0.7692307692307690	23.076923076923100	85.0574712643678	14.942528735632200	76.92307692307690
89	80.57142857142860	81.72043010752690	0.8062500000000000	0.8351648351648350	0.8	20.0	81.25	18.75	80.0
90	77.00534759358290	77.94871794871790	0.7801401869158880	0.8636363636363640	0.7102803738317760	28.971962616822400	85.0	15.0	71.02803738317760
91	80.45977011494250	81.31868131868130	0.8046129374337220	0.8222222222222220	0.8043478260869570	19.565217391304300	80.48780487804880	19.51219512195120	80.43478260869570
92	80.0	81.28342245989310	0.8048910154173310	0.8636363636363640	0.7676767676767680	23.232323232323200	84.21052631578950	15.789473684210500	76.76767676767680
93	81.35593220338980	82.35294117647060	0.8156572164948450	0.8555555555555560	0.7938144329896910	20.618556701030900	83.75	16.25	79.38144329896910
94	79.34782608695650	80.61224489795920	0.7931075460487230	0.8144329896907220	0.797979797979798	20.2020202020202	78.82352941176470	21.176470588235300	79.7979797979798
95	76.40449438202250	77.41935483870970	0.7661957490136180	0.8089887640449440	0.7422680412371130	25.773195876288700	79.01234567901230	20.98765432098770	74.22680412371140
96	77.71428571428570	78.68852459016390	0.778040451799317	0.8089887640449440	0.7659574468085110	23.404255319148900	79.01234567901230	20.98765432098770	76.59574468085110
97	77.58620689655170	76.36363636363640	0.7872751499000670	0.9	0.6631578947368420	33.68421052631580	91.13924050632910	8.860759493670890	66.3157894736842
98	82.18390804597700	83.7696335078534	0.8256756756756760	0.8791208791208790	0.8	20.0	85.13513513513510	14.864864864864900	80.0
99	84.61538461538460	86.40776699029130	0.8418286273071750	0.8476190476190480	0.8811881188118810	11.881188118811900	80.24691358024690	19.753086419753100	88.11881188118810
**Mean**	**79.02007553088440**	**79.7223057484691**	**79.3564054451716000%**	**83.7640135728559000%**	**76.2935430392280000%**	**23.706456960772**	**82.41926785111510**	**17.5807321488849**	**76.29354303922800**
**Standard Deviation**	2.81130561487120	2.98103134007662	2.7145882653722600%	3.7511423746803100%	4.8221294251427000%	4.8221294251427	3.98994527857815	3.98994527857815	4.82212942514270
**T Test Significance (Female to Male)**									

Experiment 3.1.1 (Unbalanced training data, no feature selection) - Logistic Regression Classifier

		UB_Accuracy	UB_FScore	UB_ROC_AUC	UB_Precision	UB_Recall	UB_F_Accuracy	UB_F_FScore	UB_F_ROC_AUC	UB_F_Precision	UB_F_Recall	UB_F_FNR
0		71.6	70.78189300411520	0.720744168112589	0.781818181818182	0.646616541353383	64.7887323943662	41.86046511627910	0.614983974358974	0.818181818181818	0.28125	71.875
1		74.0	68.59903381642510	0.737436783816657	0.845238095238095	0.577235772357724	77.21518987341770	57.14285714285720	0.696896551724138	0.923076923076923	0.413793103448276	58.62068965517240
2		71.6	68.72246696035240	0.717655719864285	0.78	0.614173228346457	72.05882352941180	53.65853658536580	0.663919413919414	0.733333333333333	0.423076923076923	57.69230769230770
3		67.60000000000000	63.34841628959280	0.681730769230769	0.769230769230769	0.538461538461538	75.0	50.0	0.654835847382431	0.692307692307692	0.391304347826087	60.86956521739130
4		72.40000000000000	71.12970711297070	0.734997739164137	0.833333333333333	0.620437956204380	76.05633802816900	63.82978723404260	0.725609756097561	0.882352941176471	0.5	50.0
5		70.8	67.26457399103140	0.705814549180328	0.742574257425743	0.614754098360656	75.36231884057970	58.53658536585370	0.706896551724138	1.0	0.413793103448276	58.62068965517240
6		70.0	67.53246753246750	0.704166666666667	0.772277227722772	0.6	69.51219512195120	52.83018867924530	0.64875	0.666666666666667	0.4375	56.25
7		68.4	63.59447004608300	0.682062608027655	0.734042553191489	0.560975609756098	68.67469879518070	43.47826086956520	0.632440476190476	0.909090909090909	0.285714285714286	71.42857142857140
8		73.2	71.48936170212770	0.733147685807567	0.777777777777778	0.661417322834646	77.77777777777780	57.89473684210530	0.698314108251996	0.733333333333333	0.478260869565217	52.17391304347830
9		72.40000000000000	70.88607594936710	0.725665983606557	0.770642201834862	0.65625	77.33333333333330	58.53658536585370	0.700156985871272	0.8	0.461538461538462	53.84615384615390
10		73.2	69.95515695067260	0.740369799691834	0.857142857142857	0.590909090909091	73.97260273972600	61.22448979591840	0.709984756097561	0.882352941176471	0.46875	53.125
11		70.0	66.66666666666670	0.696805439733145	0.707547169811321	0.630252100840336	64.0625	34.28571428571430	0.552083333333333	0.315789473684211	0.375	62.5
12		71.6	69.7872340425532	0.719551282051282	0.780952380952381	0.630769230769231	71.01449275362320	54.54545454545450	0.680390492359932	0.923076923076923	0.387096774193548	61.29032258064520
13		72.0	70.58823529411770	0.720000000000000	0.743362831858407	0.672	74.6268656716418	58.53658536585370	0.701465201465201	0.923076923076923	0.428571428571429	57.14285714285710
14		69.6	64.48598130841120	0.690679325165181	0.726315789473684	0.579831932773109	72.0	63.1578947368421	0.716927453769559	0.9	0.486486486486487	51.35135135135140
15		71.2	68.14159292035400	0.713718711990270	0.777777777777778	0.606299212598425	78.37837837837840	61.90476190476190	0.721273291925466	0.928571428571429	0.464285714285714	53.57142857142860
16		72.0	71.54471544715450	0.722432484444159	0.765217391304348	0.671755725190840	72.85714285714290	55.81395348837210	0.6875	0.923076923076923	0.4	60.0
17		74.8	72.48908296943230	0.747375832053251	0.790476190476190	0.669354838709677	74.6268656716418	58.53658536585370	0.692380952380952	0.75	0.48	52.0
18		68.8	66.66666666666670	0.690755333461464	0.742857142857143	0.604651162790698	69.84126984126980	57.77777777777780	0.671296296296296	0.722222222222222	0.481481481481481	51.85185185185190
19		73.6	71.55172413793100	0.736000000000000	0.775700934579439	0.664	71.42857142857140	47.61904761904760	0.637755102040816	0.714285714285714	0.357142857142857	64.28571428571430
20		72.40000000000000	69.33333333333330	0.720860863429341	0.735849056603774	0.655462184873950	72.22222222222220	50.0	0.649859943977591	0.526315789473684	0.476190476190476	52.38095238095240
21		70.8	68.6695278969957	0.711858974358974	0.776699029126214	0.615384615384615	75.71428571428570	63.82978723404260	0.729114971050455	0.9375	0.483870967741936	51.61290322580650
22		71.6	68.99563318777290	0.714395491803279	0.738317757009346	0.647540983606557	73.61111111111110	53.65853658536580	0.666666666666667	0.647058823529412	0.458333333333333	54.16666666666670
23		72.8	70.17543859649120	0.733914940021810	0.824742268041237	0.610687022900763	71.42857142857140	55.00000000000000	0.670997920997921	0.785714285714286	0.423076923076923	57.69230769230770
24		74.0	71.86147186147190	0.745365321701200	0.83	0.633587786259542	76.92307692307690	57.14285714285720	0.696138211382114	0.909090909090909	0.416666666666667	58.33333333333330
25		76.4	71.77033492822970	0.756111682964488	0.806451612903226	0.646551724137931	74.32432432432430	53.65853658536590	0.678544061302682	0.916666666666667	0.379310344827586	62.06896551724140
26		70.8	66.3594470046083	0.703846153846154	0.742268041237113	0.6	75.0	48.48484848484850	0.647922998986829	0.666666666666667	0.380952380952381	61.90476190476190
27		74.8	72.96137339055790	0.750064036885246	0.809523809523810	0.6640625	69.73684210526320	54.90196078431370	0.681944444444444	0.933333333333333	0.388888888888889	61.11111111111110
28		71.6	68.44444444444450	0.716845878136201	0.777777777777778	0.611111111111111	75.0	50.00000000000000	0.658008658008658	0.8	0.363636363636364	63.63636363636360
29		72.0	66.98113207547170	0.713193239509029	0.747368421052632	0.606837606837607	71.95121951219510	51.06382978723410	0.6575	0.8	0.375	62.5
30		71.6	68.72246696035240	0.716781874039939	0.772277227722772	0.619047619047619	73.33333333333330	55.55555555555560	0.685714285714286	0.909090909090909	0.4	60.0
31		71.6	66.35071090047390	0.708051529790660	0.729166666666667	0.608695652173913	80.76923076923080	66.66666666666670	0.748366013071895	0.833333333333333	0.555555555555556	44.44444444444440
32		69.6	68.33333333333330	0.699531721085381	0.752293577981651	0.625954198473283	70.83333333333330	58.82352941176470	0.694272445820434	0.882352941176471	0.441176470588235	55.88235294117650
33		73.6	71.7948717948718	0.734615384615385	0.736842105263158	0.7	73.01587301587300	54.05405405405410	0.666666666666667	0.625	0.476190476190476	52.38095238095240
34		75.2	72.07207207207210	0.749350649350649	0.714285714285714	0.727272727272727	77.33333333333330	56.41025641025640	0.689950980392157	0.733333333333333	0.458333333333333	54.16666666666670
35		74.8	69.85645933014350	0.743513357678263	0.829545454545455	0.603305785123967	78.37837837837840	61.90476190476190	0.721273291925466	0.928571428571429	0.464285714285714	53.57142857142860
36		69.6	67.24137931034480	0.704837879567679	0.795918367346939	0.582089552238806	67.5	40.90909090909090	0.600000000000000	0.642857142857143	0.3	70.0
37		72.40000000000000	68.20276497695850	0.721247438524590	0.778947368421053	0.606557377049180	75.90361445783130	56.52173913043480	0.687100893997446	0.764705882352941	0.448275862068966	55.17241379310340
38		73.2	70.74235807860260	0.737731733914940	0.826530612244898	0.618320610687023	74.6268656716418	51.42857142857140	0.661561264822134	0.75	0.391304347826087	60.86956521739130
39		68.4	65.19823788546260	0.682702771909609	0.711538461538462	0.601626016260163	74.6268656716418	66.66666666666670	0.727313974591652	0.772727272727273	0.586206896551724	41.37931034482760
40		68.4	65.8008658008658	0.690421160760144	0.767676767676768	0.575757575757576	70.83333333333330	43.24324324324320	0.637931034482759	1.0	0.275862068965517	72.41379310344830
41		73.6	71.05263157894740	0.737596824787146	0.801980198019802	0.637795275590551	75.0	62.96296296296300	0.729729729729730	1.0	0.459459459459460	54.05405405405410
42		70.0	66.3677130044843	0.701907688368222	0.770833333333333	0.582677165354331	72.6027397260274	52.38095238095240	0.660225442834139	0.733333333333333	0.407407407407407	59.25925925925930
43		68.4	63.92694063926940	0.684	0.744680851063830	0.56	64.7887323943662	35.8974358974359	0.584975369458128	0.7	0.241379310344828	75.86206896551720
44		73.2	70.48458149779740	0.731310803891449	0.776699029126214	0.645161290322581	70.58823529411770	54.54545454545450	0.680034873583261	0.923076923076923	0.387096774193548	61.29032258064520
45		68.0	62.26415094339620	0.682606747327316	0.776470588235294	0.519685039370079	69.56521739130430	51.16279069767440	0.647648083623693	0.733333333333333	0.392857142857143	60.71428571428570
46		71.6	69.26406926406930	0.730023900264841	0.851063829787234	0.583941605839416	72.3076923076923	50.0	0.660256410256410	0.9	0.346153846153846	65.38461538461540
47		74.0	69.48356807511740	0.732850241545894	0.755102040816327	0.643478260869565	79.71014492753620	56.25	0.693452380952381	0.818181818181818	0.428571428571429	57.14285714285710
48		73.6	67.0	0.723020186335404	0.761363636363636	0.598214285714286	76.31578947368420	57.14285714285710	0.691609977324263	0.8	0.444444444444444	55.55555555555560
49		70.0	67.81115879828330	0.702036372950820	0.752380952380952	0.6171875	71.42857142857140	56.52173913043480	0.679166666666667	0.8125	0.433333333333333	56.66666666666670
50		74.8	73.41772151898740	0.750432442821449	0.805555555555556	0.674418604651163	76.92307692307690	60.86956521739130	0.710239651416122	0.736842105263158	0.518518518518519	48.14814814814810
51		72.40000000000000	68.20276497695850	0.718862352336929	0.747474747474748	0.627118644067797	75.67567567567570	62.5	0.722470238095238	0.9375	0.46875	53.125
52		73.2	70.74235807860260	0.735441091677878	0.81	0.627906976744186	77.33333333333330	65.3061224489796	0.732383808095952	0.8	0.551724137931035	44.82758620689660
53		72.40000000000000	68.77828054298640	0.722328916202548	0.775510204081633	0.617886178861789	78.125	56.25000000000000	0.695652173913044	0.642857142857143	0.5	50.0
54		70.8	65.72769953051640	0.708000000000000	0.795454545454545	0.56	75.30864197530870	58.33333333333330	0.693396226415094	0.7	0.5	50.0
55		70.8	66.9683257918552	0.703457361352098	0.711538461538462	0.632478632478633	78.94736842105260	53.84615384615390	0.680882352941176	0.777777777777778	0.411764705882353	58.82352941176470
56		71.2	68.14159292035400	0.710105020491803	0.740384615384615	0.631147540983607	76.71232876712330	56.41025641025640	0.692834138486313	0.916666666666667	0.407407407407407	59.25925925925930
57		74.4	70.37037037037040	0.738311688311688	0.716981132075472	0.690909090909091	76.47058823529410	57.89473684210530	0.695075757575758	0.785714285714286	0.458333333333333	54.16666666666670
58		71.2	69.49152542372880	0.713755122950820	0.759259259259259	0.640625	76.31578947368420	62.5	0.717391304347826	0.833333333333333	0.5	50.0
59		68.4	66.38297872340430	0.688466226185131	0.75	0.595419847328244	68.75	50.98039215686270	0.643778207607995	0.722222222222222	0.393939393939394	60.60606060606060
60		67.60000000000000	64.62882096069870	0.682845403184386	0.762886597938144	0.560606060606061	63.63636363636360	45.45454545454550	0.636363636363636	0.909090909090909	0.303030303030303	69.6969696969697
61		74.0	71.86147186147190	0.745365321701200	0.83	0.633587786259542	78.57142857142860	66.66666666666670	0.743006993006993	0.789473684210526	0.576923076923077	42.30769230769230
62		68.4	63.92694063926940	0.685035842293907	0.752688172043011	0.555555555555556	73.17073170731710	57.6923076923077	0.689338235294118	0.833333333333333	0.441176470588235	55.88235294117650
63		75.2	72.80701754385970	0.749502854576945	0.761467889908257	0.697478991596639	82.08955223880600	71.42857142857140	0.780632411067194	0.789473684210526	0.652173913043478	34.78260869565220
64		72.8	69.36936936936940	0.723687596500257	0.726415094339623	0.663793103448276	76.82926829268290	59.57446808510640	0.703703703703704	0.736842105263158	0.5	50.0
65		71.6	69.52789699570820	0.718045594262295	0.771428571428572	0.6328125	71.42857142857140	47.05882352941180	0.646842105263158	0.888888888888889	0.32	68.0
66		69.6	70.07874015748030	0.706898800208659	0.794642857142857	0.626760563380282	77.94117647058820	70.58823529411760	0.763295553618134	0.9	0.580645161290323	41.93548387096770
67		71.2	66.66666666666670	0.707101161075117	0.742268041237113	0.605042016806723	73.13432835820900	55.00000000000000	0.674953095684803	0.785714285714286	0.423076923076923	57.69230769230770
68		70.40000000000000	66.66666666666670	0.709615384615385	0.804347826086957	0.569230769230769	66.66666666666670	44.44444444444440	0.627705627705628	0.833333333333333	0.303030303030303	69.6969696969697
69		68.8	65.17857142857140	0.685899077868853	0.715686274509804	0.598360655737705	69.44444444444440	54.16666666666670	0.665625	0.8125	0.40625	59.375
70		72.0	67.28971962616820	0.717879777222969	0.791208791208791	0.585365853658537	76.81159420289860	52.94117647058820	0.672630560928433	0.75	0.409090909090909	59.09090909090910
71		72.0	68.18181818181820	0.716346153846154	0.75	0.625	74.11764705882350	59.25925925925930	0.696078431372549	0.8	0.470588235294118	52.94117647058820
72		71.6	67.57990867579910	0.718167850969848	0.804347826086957	0.582677165354331	71.23287671232880	43.24324324324320	0.631746031746032	0.888888888888889	0.285714285714286	71.42857142857140
73		70.40000000000000	67.82608695652170	0.707284259081299	0.772277227722772	0.604651162790698	71.21212121212120	61.22448979591840	0.688034188034188	0.681818181818182	0.555555555555556	44.44444444444440
74		72.8	70.17543859649120	0.725641025641026	0.740740740740741	0.666666666666667	73.68421052631580	56.52173913043480	0.684057971014493	0.8125	0.433333333333333	56.66666666666670
75		67.2	63.06306306306310	0.681961313540261	0.786516853932584	0.526315789473684	64.70588235294120	40.0	0.592857142857143	0.666666666666667	0.285714285714286	71.42857142857140
76		75.2	72.32142857142860	0.749038461538462	0.778846153846154	0.675	76.78571428571430	51.85185185185190	0.670697012802276	0.875	0.368421052631579	63.1578947368421
77		69.6	66.66666666666670	0.703133025166923	0.791666666666667	0.575757575757576	67.64705882352940	38.88888888888890	0.6125	0.875	0.25	75.0
78		68.8	66.37931034482760	0.690125512295082	0.740384615384615	0.6015625	73.84615384615390	60.46511627906980	0.710249042145594	0.928571428571429	0.448275862068966	55.17241379310340
79		75.6	73.81974248927040	0.758068647540984	0.819047619047619	0.671875	79.41176470588240	68.18181818181820	0.753387533875339	0.882352941176471	0.555555555555556	44.44444444444440
80		71.2	66.66666666666670	0.713133640552995	0.8	0.571428571428571	72.3076923076923	59.09090909090910	0.691602316602317	0.8125	0.464285714285714	53.57142857142860
81		67.2	65.83333333333330	0.685073562771405	0.782178217821782	0.568345323741007	66.23376623376620	51.85185185185190	0.645663956639566	0.777777777777778	0.388888888888889	61.11111111111110
82		68.8	63.20754716981130	0.680584148224395	0.697916666666667	0.577586206896552	72.72727272727270	55.31914893617020	0.677068723702665	0.8125	0.419354838709677	58.06451612903230
83		69.6	65.45454545454550	0.690941842511580	0.692307692307692	0.620689655172414	75.0	57.14285714285710	0.691558441558442	0.857142857142857	0.428571428571429	57.14285714285710
84		73.2	68.83720930232560	0.732000000000000	0.822222222222222	0.592	69.86301369863010	47.61904761904760	0.638322884012539	0.769230769230769	0.344827586206897	65.51724137931040
85		65.60000000000000	62.93103448275860	0.654019003595275	0.640350877192982	0.618644067796610	64.28571428571430	50.98039215686270	0.614381833473507	0.590909090909091	0.448275862068966	55.17241379310340
86		74.4	74.4	0.744762636940227	0.768595041322314	0.720930232558140	75.36231884057970	63.82978723404260	0.720454545454546	0.681818181818182	0.6	40.0
87		68.8	65.48672566371680	0.703701311284801	0.831460674157303	0.540145985401460	72.97297297297300	54.54545454545450	0.68192048012003	0.923076923076923	0.387096774193548	61.29032258064520
88		67.2	66.39344262295080	0.689758839291550	0.801980198019802	0.566433566433567	65.75342465753420	56.14035087719300	0.675716440422323	0.888888888888889	0.410256410256410	58.97435897435900
89		66.4	61.11111111111110	0.660324171952079	0.694736842105263	0.545454545454545	73.33333333333330	54.54545454545450	0.671732522796353	0.75	0.428571428571429	57.14285714285710
90		72.40000000000000	69.60352422907490	0.722713014531720	0.759615384615385	0.642276422764228	75.67567567567570	57.14285714285720	0.7	1.0	0.4	60.0
91		70.40000000000000	67.2566371681416	0.704813108038915	0.76	0.603174603174603	74.66666666666670	55.81395348837210	0.688888888888889	0.923076923076923	0.4	60.0
92		69.6	66.3716814159292	0.697714614941425	0.757575757575758	0.590551181102362	74.68354430379750	61.53846153846150	0.713071895424837	0.888888888888889	0.470588235294118	52.94117647058820
93		76.0	72.97297297297300	0.760944700460830	0.84375	0.642857142857143	75.75757575757580	60.0	0.709401709401709	0.923076923076923	0.444444444444444	55.55555555555560
94		70.0	64.45497630331760	0.695720417707733	0.755555555555556	0.561983471074380	70.27027027027030	47.61904761904760	0.635093167701863	0.714285714285714	0.357142857142857	64.28571428571430
95		69.6	67.79661016949150	0.706398348813210	0.8	0.588235294117647	77.02702702702700	60.46511627906980	0.713026819923372	0.928571428571429	0.448275862068966	55.17241379310340
96		70.0	66.9603524229075	0.700780849974398	0.752475247524753	0.603174603174603	75.67567567567570	66.66666666666670	0.739705882352941	0.9	0.529411764705882	47.05882352941180
97		72.0	69.82758620689650	0.735442546583851	0.861702127659574	0.586956521739131	73.52941176470590	60.8695652173913	0.702917771883289	0.823529411764706	0.482758620689655	51.72413793103450
98		66.8	62.10045662100460	0.668	0.723404255319149	0.544	70.96774193548390	50.0	0.666666666666667	1.0	0.333333333333333	66.66666666666670
99		70.40000000000000	65.74074074074080	0.702956989247312	0.771739130434783	0.572580645161290	72.46376811594200	45.71428571428570	0.633333333333333	0.727272727272727	0.333333333333333	66.66666666666670
	**Mean**	**71.308**	**68.1839411116999**	**71.4222927643159%**	**77.03547165049840**	**61.3715967069092%**	**73.3344725921511**	**55.2439703311362**	**68.03614462365760**	**81.3104486629641%**	**42.6972761599435%**	**57.3027238400565**
	**Standard Deviation**	2.37076810636161	2.88348513473803	2.27504437576139%	3.979417586767020	4.19153972746257%	3.9458648141194	7.2766427785954	3.910034780511530	11.3111542049613%	7.84582774201899%	7.84582774201899
	**T Test Significance (Female to Male)**						**0.010069363087628**	**0.000000000000000**	**0.001284939176859**	**0.297372715582856**	**0.000000000000000**	**0.000000000000000**

		UB_F_TNR	UB_F_FPR	UB_F_TPR	UB_M_Accuracy	UB_M_FScore	UB_M_ROC_AUC	UB_M_Precision	UB_M_Recall	UB_M_FNR	UB_M_TNR	UB_M_FPR	UB_M_TPR
0		94.87179487179490	5.128205128205130	28.125	74.30167597765360	77.00000000000000	0.740162477786240	0.777777777777778	0.762376237623762	23.76237623762380	71.7948717948718	28.20512820512820	76.23762376237620
1		98.0	2.0	41.37931034482760	72.51461988304090	71.51515151515150	0.735907709311965	0.830985915492958	0.627659574468085	37.23404255319150	84.4155844155844	15.58441558441560	62.76595744680850
2		90.47619047619050	9.523809523809520	42.30769230769230	71.42857142857140	72.04301075268820	0.720572057205721	0.788235294117647	0.663366336633663	33.66336633663370	77.77777777777780	22.22222222222220	66.33663366336630
3		91.83673469387760	8.16326530612245	39.13043478260870	64.60674157303370	65.94594594594590	0.665328419112808	0.782051282051282	0.570093457943925	42.99065420560750	76.05633802816900	23.94366197183100	57.00934579439250
4		95.1219512195122	4.878048780487810	50.0	70.94972067039110	72.91666666666670	0.722936137071651	0.823529411764706	0.654205607476636	34.57943925233650	79.16666666666670	20.83333333333330	65.42056074766350
5		100.0	0.0	41.37931034482760	69.06077348066300	69.23076923076920	0.690982404692082	0.707865168539326	0.677419354838710	32.25806451612900	70.45454545454550	29.54545454545450	67.74193548387100
6		86.0	14.00000000000000	43.75	70.23809523809520	71.91011235955060	0.712244897959184	0.8	0.653061224489796	34.69387755102040	77.14285714285720	22.85714285714290	65.3061224489796
7		97.91666666666670	2.083333333333330	28.57142857142860	68.26347305389220	69.00584795321640	0.683328538550057	0.710843373493976	0.670454545454545	32.95454545454550	69.62025316455700	30.37974683544300	67.04545454545460
8		91.83673469387760	8.16326530612245	47.82608695652170	71.34831460674160	74.11167512690360	0.715826403326403	0.784946236559140	0.701923076923077	29.80769230769230	72.97297297297300	27.02702702702700	70.1923076923077
9		93.87755102040820	6.122448979591840	46.15384615384620	70.28571428571430	73.46938775510210	0.702256244963739	0.765957446808511	0.705882352941177	29.41176470588240	69.86301369863010	30.13698630136990	70.58823529411770
10		95.1219512195122	4.878048780487810	46.875	72.88135593220340	72.41379310344830	0.743571428571429	0.851351351351351	0.63	37.0	85.71428571428570	14.28571428571430	63.0
11		72.91666666666670	27.08333333333330	37.5	72.04301075268820	72.63157894736840	0.726517721370921	0.793103448275862	0.669902912621359	33.00970873786410	78.3132530120482	21.68674698795180	66.99029126213590
12		97.36842105263160	2.631578947368420	38.70967741935480	71.8232044198895	73.29842931937170	0.719389012071939	0.760869565217391	0.707070707070707	29.29292929292930	73.17073170731710	26.82926829268290	70.70707070707070
13		97.43589743589740	2.564102564102560	42.85714285714290	71.03825136612020	73.09644670050760	0.708343322944138	0.72	0.742268041237113	25.77319587628870	67.44186046511630	32.55813953488370	74.22680412371140
14		94.73684210526320	5.263157894736840	48.64864864864870	68.57142857142860	64.96815286624210	0.681943351691581	0.68	0.621951219512195	37.80487804878050	74.19354838709680	25.80645161290320	62.19512195121950
15		97.82608695652170	2.173913043478260	46.42857142857140	68.18181818181820	69.56521739130430	0.686868686868687	0.752941176470588	0.646464646464647	35.35353535353540	72.72727272727270	27.27272727272730	64.64646464646470
16		97.5	2.5	40.0	71.66666666666670	74.8768472906404	0.711680661737060	0.745098039215686	0.752475247524753	24.75247524752480	67.08860759493670	32.91139240506330	75.24752475247520
17		90.47619047619050	9.523809523809520	48.0	74.86338797814210	75.53191489361700	0.751443001443001	0.797752808988764	0.717171717171717	28.28282828282830	78.57142857142860	21.42857142857140	71.71717171717170
18		86.11111111111110	13.88888888888890	48.14814814814810	68.44919786096260	68.78306878306880	0.689215686274510	0.747126436781609	0.637254901960784	36.27450980392160	74.11764705882350	25.88235294117650	63.72549019607840
19		91.83673469387760	8.16326530612245	35.71428571428570	74.56647398843930	76.8421052631579	0.744709712425393	0.784946236559140	0.752577319587629	24.74226804123710	73.68421052631580	26.31578947368420	75.25773195876290
20		82.35294117647060	17.64705882352940	47.61904761904760	72.47191011235960	73.51351351351350	0.728188775510204	0.781609195402299	0.693877551020408	30.61224489795920	76.25	23.75	69.38775510204080
21		97.43589743589740	2.564102564102560	48.38709677419360	68.88888888888890	69.89247311827960	0.692480359147026	0.747126436781609	0.656565656565657	34.34343434343430	72.8395061728395	27.16049382716050	65.65656565656570
22		87.5	12.5	45.83333333333330	70.78651685393260	72.3404255319149	0.709438775510204	0.755555555555556	0.693877551020408	30.61224489795920	72.5	27.50000000000000	69.38775510204080
23		91.8918918918919	8.108108108108110	42.30769230769230	73.2620320855615	73.40425531914890	0.743205574912892	0.831325301204819	0.657142857142857	34.28571428571430	82.92682926829270	17.07317073170730	65.71428571428570
24		97.5609756097561	2.439024390243900	41.66666666666670	72.97297297297300	74.48979591836730	0.738557392763000	0.820224719101124	0.682242990654206	31.77570093457940	79.48717948717950	20.51282051282050	68.22429906542060
25		97.77777777777780	2.222222222222220	37.93103448275860	77.27272727272730	76.1904761904762	0.772310473976495	0.790123456790123	0.735632183908046	26.43678160919540	80.89887640449440	19.10112359550560	73.5632183908046
26		91.48936170212770	8.51063829787234	38.09523809523810	69.23076923076920	69.56521739130430	0.696726299135938	0.752941176470588	0.646464646464647	35.35353535353540	74.69879518072290	25.30120481927710	64.64646464646470
27		97.5	2.5	38.88888888888890	77.01149425287360	78.02197802197800	0.770015906680806	0.788888888888889	0.771739130434783	22.82608695652170	76.82926829268290	23.17073170731710	77.17391304347830
28		95.23809523809520	4.761904761904760	36.36363636363640	70.43010752688170	71.50259067357510	0.709779549718574	0.775280898876405	0.663461538461538	33.65384615384620	75.60975609756100	24.39024390243900	66.34615384615380
29		94.0	6.0	37.5	72.02380952380950	71.51515151515150	0.720552799433026	0.7375	0.694117647058824	30.58823529411770	74.69879518072290	25.30120481927710	69.41176470588240
30		97.14285714285710	2.857142857142860	40.0	71.05263157894740	71.20418848167540	0.713038157748359	0.755555555555556	0.673267326732673	32.67326732673270	75.28089887640450	24.71910112359550	67.32673267326730
31		94.11764705882350	5.88235294117647	55.55555555555560	67.44186046511630	66.26506024096390	0.675595238095238	0.705128205128205	0.625	37.5	72.61904761904760	27.38095238095240	62.5
32		94.73684210526320	5.263157894736840	44.11764705882350	69.10112359550560	70.89947089947090	0.691039837087947	0.728260869565217	0.690721649484536	30.92783505154640	69.1358024691358	30.86419753086420	69.0721649484536
33		85.71428571428570	14.28571428571430	47.61904761904760	73.79679144385030	75.12690355329950	0.737373737373737	0.755102040816327	0.747474747474748	25.25252525252530	72.72727272727270	27.27272727272730	74.74747474747480
34		92.15686274509800	7.843137254901960	45.83333333333330	74.28571428571430	75.40983606557380	0.743859419911158	0.711340206185567	0.802325581395349	19.76744186046510	68.53932584269660	31.46067415730340	80.23255813953490
35		97.82608695652170	2.173913043478260	46.42857142857140	73.29545454545460	71.8562874251497	0.7382432957637	0.810810810810811	0.645161290322581	35.48387096774190	83.13253012048190	16.86746987951810	64.51612903225810
36		90.0	10.0	30.0	70.58823529411770	73.40425531914890	0.718094405594406	0.821428571428571	0.663461538461538	33.65384615384620	77.27272727272730	22.72727272727270	66.34615384615380
37		92.5925925925926	7.407407407407410	44.82758620689660	70.65868263473050	71.34502923976610	0.713092124382447	0.782051282051282	0.655913978494624	34.40860215053760	77.02702702702700	22.97297297297300	65.59139784946240
38		93.18181818181820	6.818181818181820	39.13043478260870	72.6775956284153	74.22680412371140	0.740000000000000	0.837209302325581	0.666666666666667	33.33333333333330	81.33333333333330	18.66666666666670	66.66666666666670
39		86.8421052631579	13.15789473684210	58.62068965517240	66.12021857923500	64.77272727272730	0.662742051159455	0.695121951219512	0.606382978723404	39.36170212765960	71.91011235955060	28.08988764044940	60.63829787234040
40		100.0	0.0	27.58620689655170	67.41573033707870	70.10309278350520	0.676763754045308	0.747252747252747	0.660194174757282	33.98058252427180	69.33333333333330	30.66666666666670	66.01941747572820
41		100.0	0.0	45.94594594594600	72.94117647058820	73.5632183908046	0.730555555555556	0.761904761904762	0.711111111111111	28.88888888888890	75.0	25.0	71.11111111111110
42		91.30434782608700	8.695652173913040	40.74074074074070	68.92655367231640	69.61325966850830	0.698116883116883	0.777777777777778	0.63	37.0	76.62337662337660	23.37662337662340	63.0
43		92.85714285714290	7.142857142857140	24.13793103448280	69.83240223463690	70.0	0.701618975903614	0.75	0.65625	34.375	74.69879518072290	25.30120481927710	65.625
44		97.2972972972973	2.702702702702700	38.70967741935480	74.17582417582420	74.31693989071040	0.741995892231485	0.755555555555556	0.731182795698925	26.88172043010750	75.28089887640450	24.71910112359550	73.11827956989250
45		90.2439024390244	9.75609756097561	39.28571428571430	67.40331491712710	65.08875739644970	0.686314363143632	0.785714285714286	0.555555555555556	44.44444444444440	81.70731707317070	18.29268292682930	55.55555555555560
46		97.43589743589740	2.564102564102560	34.61538461538460	71.35135135135140	72.82051282051280	0.731981981981982	0.845238095238095	0.639639639639640	36.03603603603600	82.43243243243240	17.56756756756760	63.96396396396400
47		95.83333333333330	4.166666666666670	42.85714285714290	71.8232044198895	71.8232044198895	0.719307899241868	0.747126436781609	0.691489361702128	30.85106382978720	74.71264367816090	25.28735632183910	69.14893617021280
48		93.87755102040820	6.122448979591840	44.44444444444440	72.41379310344830	69.62025316455700	0.722405816259088	0.753424657534247	0.647058823529412	35.29411764705880	79.7752808988764	20.22471910112360	64.70588235294120
49		92.5	7.5	43.33333333333330	69.44444444444440	70.58823529411760	0.696490791438527	0.741573033707865	0.673469387755102	32.6530612244898	71.95121951219510	28.04878048780490	67.3469387755102
50		90.19607843137260	9.803921568627450	51.85185185185190	73.83720930232560	76.43979057591620	0.743557422969188	0.820224719101124	0.715686274509804	28.43137254901960	77.14285714285720	22.85714285714290	71.56862745098040
51		97.61904761904760	2.380952380952380	46.875	71.02272727272730	69.8224852071006	0.709689922480620	0.710843373493976	0.686046511627907	31.3953488372093	73.33333333333330	26.66666666666670	68.6046511627907
52		91.30434782608700	8.695652173913040	55.17241379310340	71.42857142857140	72.22222222222220	0.725000000000000	0.8125	0.65	35.0	80.0	20.0	65.0
53		89.13043478260870	10.86956521739130	50.0	70.43010752688170	70.8994708994709	0.714109347442681	0.797619047619048	0.638095238095238	36.19047619047620	79.01234567901230	20.98765432098770	63.8095238095238
54		88.67924528301890	11.32075471698110	50.0	68.63905325443790	67.87878787878790	0.705326460481100	0.823529411764706	0.577319587628866	42.2680412371134	83.33333333333330	16.66666666666670	57.73195876288660
55		95.0	5.0	41.17647058823530	68.39378238341970	68.71794871794870	0.684462365591398	0.705263157894737	0.67	33.0	69.89247311827960	30.10752688172040	67.0
56		97.82608695652170	2.173913043478260	40.74074074074070	68.92655367231640	70.58823529411770	0.688831835686778	0.717391304347826	0.694736842105263	30.52631578947370	68.29268292682930	31.70731707317070	69.47368421052630
57		93.18181818181820	6.818181818181820	45.83333333333330	73.62637362637360	73.03370786516850	0.737281976744186	0.706521739130435	0.755813953488372	24.41860465116280	71.875	28.125	75.5813953488372
58		93.47826086956520	6.521739130434780	50.0	68.96551724137930	71.27659574468090	0.690520945220193	0.744444444444445	0.683673469387755	31.63265306122450	69.73684210526320	30.26315789473680	68.36734693877550
59		89.36170212765960	10.63829787234040	39.39393939393940	68.23529411764710	70.65217391304350	0.685799319727891	0.755813953488372	0.663265306122449	33.6734693877551	70.83333333333330	29.16666666666670	66.3265306122449
60		96.96969696969700	3.030303030303030	30.30303030303030	69.02173913043480	69.18918918918920	0.693820558526441	0.744186046511628	0.646464646464647	35.35353535353540	74.11764705882350	25.88235294117650	64.64646464646470
61		90.9090909090909	9.090909090909090	57.69230769230770	72.22222222222220	73.11827956989250	0.737142857142857	0.839506172839506	0.647619047619048	35.23809523809520	82.66666666666670	17.33333333333330	64.76190476190480
62		93.75	6.25	44.11764705882350	66.07142857142860	65.86826347305390	0.667334096109840	0.733333333333333	0.597826086956522	40.21739130434780	73.68421052631580	26.31578947368420	59.78260869565220
63		90.9090909090909	9.090909090909090	65.21739130434780	72.6775956284153	73.11827956989250	0.727729885057471	0.755555555555556	0.708333333333333	29.16666666666670	74.71264367816090	25.28735632183910	70.83333333333330
64		90.74074074074080	9.25925925925926	50.0	70.83333333333330	72.0	0.707954545454545	0.724137931034483	0.715909090909091	28.40909090909090	70.0	30.0	71.5909090909091
65		97.36842105263160	2.631578947368420	32.0	71.65775401069520	73.36683417085430	0.717464170134073	0.760416666666667	0.708737864077670	29.12621359223300	72.61904761904760	27.38095238095240	70.87378640776700
66		94.5945945945946	5.405405405405410	58.06451612903230	66.48351648351650	69.95073891625620	0.671932495876158	0.771739130434783	0.639639639639640	36.03603603603600	70.4225352112676	29.57746478873240	63.96396396396400
67		92.6829268292683	7.317073170731710	42.30769230769230	70.49180327868850	69.31818181818180	0.705734767025090	0.734939759036145	0.655913978494624	34.40860215053760	75.55555555555560	24.44444444444440	65.59139784946240
68		95.23809523809520	4.761904761904760	30.30303030303030	72.0	72.31638418079100	0.727332804652392	0.8	0.659793814432990	34.02061855670100	79.48717948717950	20.51282051282050	65.97938144329900
69		92.5	7.5	40.625	68.53932584269660	68.18181818181820	0.685606060606061	0.697674418604651	0.666666666666667	33.33333333333330	70.45454545454550	29.54545454545450	66.66666666666670
70		93.61702127659580	6.382978723404260	40.90909090909090	70.1657458563536	70.0	0.711881188118812	0.797468354430380	0.623762376237624	37.62376237623760	80.0	20.0	62.37623762376240
71		92.15686274509800	7.843137254901960	47.05882352941180	70.9090909090909	71.08433734939760	0.710111863408890	0.7375	0.686046511627907	31.3953488372093	73.41772151898740	26.58227848101270	68.6046511627907
72		97.77777777777780	2.222222222222220	28.57142857142860	71.75141242937850	72.52747252747250	0.724358974358974	0.795180722891566	0.666666666666667	33.33333333333330	78.2051282051282	21.79487179487180	66.66666666666670
73		82.05128205128200	17.94871794871800	55.55555555555560	70.1086956521739	69.61325966850830	0.711262553802009	0.797468354430380	0.617647058823529	38.23529411764710	80.48780487804880	19.51219512195120	61.76470588235290
74		93.47826086956520	6.521739130434780	43.33333333333330	72.41379310344830	73.62637362637360	0.723412698412698	0.728260869565217	0.744444444444445	25.55555555555560	70.23809523809520	29.76190476190480	74.44444444444440
75		90.0	10.0	28.57142857142860	68.13186813186810	68.13186813186810	0.697835497835498	0.805194805194805	0.590476190476191	40.95238095238100	80.51948051948050	19.48051948051950	59.04761904761910
76		97.2972972972973	2.702702702702700	36.84210526315790	74.74226804123710	75.1269035532995	0.748057063770893	0.770833333333333	0.732673267326733	26.73267326732670	76.34408602150540	23.65591397849460	73.26732673267330
77		97.5	2.5	25.0	70.32967032967030	71.875	0.709935897435897	0.784090909090909	0.663461538461538	33.65384615384620	75.64102564102560	24.35897435897440	66.34615384615380
78		97.22222222222220	2.777777777777780	44.82758620689660	67.02702702702700	67.72486772486770	0.672069532534649	0.711111111111111	0.646464646464647	35.35353535353540	69.76744186046510	30.23255813953490	64.64646464646470
79		95.1219512195122	4.878048780487810	55.55555555555560	74.17582417582420	75.13227513227510	0.746546876909913	0.806818181818182	0.702970297029703	29.7029702970297	79.01234567901230	20.98765432098770	70.29702970297030
80		91.8918918918919	8.108108108108110	46.42857142857140	70.8108108108108	68.6046511627907	0.714813511611541	0.797297297297297	0.602040816326531	39.79591836734690	82.75862068965520	17.24137931034480	60.20408163265310
81		90.2439024390244	9.75609756097561	38.88888888888890	67.63005780346820	69.89247311827960	0.686962552011096	0.783132530120482	0.631067961165049	36.89320388349520	74.28571428571430	25.71428571428570	63.10679611650490
82		93.47826086956520	6.521739130434780	41.93548387096770	67.05202312138730	65.45454545454550	0.669919786096257	0.675	0.635294117647059	36.47058823529410	70.45454545454550	29.54545454545450	63.52941176470590
83		95.45454545454550	4.545454545454550	42.85714285714290	67.41573033707870	67.41573033707870	0.674242424242424	0.666666666666667	0.681818181818182	31.81818181818180	66.66666666666670	33.33333333333330	68.18181818181820
84		93.18181818181820	6.818181818181820	34.48275862068970	74.57627118644070	73.98843930635840	0.753086419753086	0.831168831168831	0.666666666666667	33.33333333333330	83.9506172839506	16.04938271604940	66.66666666666670
85		78.04878048780490	21.95121951219510	44.82758620689660	66.11111111111110	66.29834254143650	0.661254475861217	0.652173913043478	0.674157303370787	32.58426966292140	64.83516483516480	35.16483516483520	67.41573033707870
86		84.0909090909091	15.90909090909090	60.0	74.03314917127070	76.84729064039410	0.738636363636364	0.787878787878788	0.75	25.0	72.72727272727270	27.27272727272730	75.0
87		97.67441860465120	2.325581395348840	38.70967741935480	67.04545454545460	68.13186813186810	0.692452830188679	0.815789473684211	0.584905660377359	41.50943396226420	80.0	20.0	58.49056603773580
88		94.11764705882350	5.88235294117647	41.02564102564100	67.79661016949150	69.5187165775401	0.689212328767123	0.783132530120482	0.625	37.5	75.34246575342470	24.65753424657530	62.5
89		91.48936170212770	8.51063829787234	42.85714285714290	63.42857142857140	62.7906976744186	0.637883556254917	0.683544303797468	0.580645161290323	41.93548387096770	69.51219512195120	30.48780487804880	58.06451612903230
90		100.0	0.0	40.0	71.02272727272730	72.43243243243240	0.709612644124887	0.728260869565217	0.720430107526882	27.95698924731180	69.87951807228920	30.12048192771080	72.04301075268820
91		97.77777777777780	2.222222222222220	40.0	68.57142857142860	69.94535519125680	0.687763713080169	0.735632183908046	0.666666666666667	33.33333333333330	70.88607594936710	29.11392405063290	66.66666666666670
92		95.55555555555560	4.444444444444450	47.05882352941180	67.2514619883041	67.81609195402300	0.676178660049628	0.728395061728395	0.634408602150538	36.55913978494620	71.7948717948718	28.20512820512820	63.44086021505380
93		97.43589743589740	2.564102564102560	44.44444444444440	76.08695652173910	75.82417582417580	0.766131907308378	0.831325301204819	0.696969696969697	30.30303030303030	83.52941176470590	16.47058823529410	69.6969696969697
94		91.30434782608700	8.695652173913040	35.71428571428570	69.88636363636360	68.63905325443790	0.703394222049488	0.763157894736842	0.623655913978495	37.63440860215050	78.3132530120482	21.68674698795180	62.36559139784950
95		97.77777777777780	2.222222222222220	44.82758620689660	66.47727272727270	69.43005181347150	0.675402952729243	0.779069767441861	0.626168224299065	37.38317757009350	72.46376811594200	27.53623188405800	62.61682242990650
96		95.0	5.0	52.94117647058820	67.61363636363640	67.05202312138730	0.678312629399586	0.716049382716049	0.630434782608696	36.95652173913040	72.61904761904760	27.38095238095240	63.04347826086960
97		92.3076923076923	7.692307692307690	48.27586206896550	71.42857142857140	72.04301075268820	0.738846298856353	0.870129870129870	0.614678899082569	38.53211009174310	86.3013698630137	13.69863013698630	61.46788990825690
98		100.0	0.0	33.33333333333330	65.42553191489360	64.48087431693990	0.656575963718821	0.694117647058824	0.602040816326531	39.79591836734690	71.11111111111110	28.88888888888890	60.20408163265310
99		93.33333333333330	6.666666666666670	33.33333333333330	69.61325966850830	69.61325966850830	0.703888888888889	0.777777777777778	0.63	37.0	77.77777777777780	22.22222222222220	63.0
	**Mean**	**93.3750130873717**	**6.62498691262834**	**42.6972761599435**	**70.48716131576840**	**71.10660863885450**	**70.96979007434640**	**76.4427996423034%**	**66.76423076957090**	**33.2357692304291**	**75.1753493791219**	**24.8246506208781**	**66.7642307695709**
	**Standard Deviation**	4.6447133643851	4.6447133643851	7.84582774201899	2.74456383804250	3.13948182539336	2.713754737356130	4.51656859250594%	4.796441155391480	4.79644115539148	4.76660700305726	4.76660700305726	4.79644115539148
	**T Test Significance (Female to Male)**	**0.000000000000000**	**0.000000000000000**	**0.000000000000000**									

Experiment 3.1.1 (Unbalanced training data, no feature selection) - Support Vector Classifier

	UB_Accuracy	UB_FScore	UB_ROC_AUC	UB_Precision	UB_Recall	UB_F_Accuracy	UB_F_FScore	UB_F_ROC_AUC	UB_F_Precision	UB_F_Recall
0	76.4	74.23580786026200	0.764720942140297	0.825242718446602	0.674603174603175	79.72972972972970	73.6842105263158	0.784291187739464	0.75	0.724137931034483
1	81.6	81.4516129032258	0.816116231438812	0.827868852459016	0.801587301587302	83.78378378378380	77.77777777777780	0.838367346938776	0.724137931034483	0.84
2	76.4	73.30316742081450	0.763120839733743	0.835051546391753	0.653225806451613	78.75	63.82978723404260	0.734375	1.0	0.46875
3	74.0	73.68421052631580	0.741666666666667	0.777777777777778	0.7	75.71428571428570	72.1311475409836	0.757359125315391	0.6875	0.758620689655172
4	80.80000000000000	80.95238095238100	0.808273565573771	0.822580645161290	0.796875	88.1578947368421	83.0188679245283	0.861607142857143	0.88	0.785714285714286
5	82.40000000000000	83.33333333333330	0.823314768580757	0.802919708029197	0.866141732283465	83.09859154929580	80.0	0.860434782608696	0.685714285714286	0.96
6	79.2	77.58620689655170	0.790505477609072	0.810810810810811	0.743801652892562	75.71428571428570	71.18644067796610	0.748966087675765	0.75	0.677419354838710
7	82.8	83.39768339768340	0.828582434514638	0.850393700787402	0.818181818181818	84.28571428571430	80.70175438596490	0.832506203473945	0.884615384615385	0.741935483870968
8	78.0	75.9825327510917	0.784615384615385	0.878787878787879	0.669230769230769	77.38095238095240	57.77777777777780	0.696865203761755	0.8125	0.448275862068966
9	79.60000000000000	78.48101265822780	0.796	0.830357142857143	0.744	82.75862068965520	79.16666666666670	0.834595959595960	0.730769230769231	0.863636363636364
10	84.8	83.33333333333330	0.846050355564098	0.887850467289720	0.785123966942149	87.5	81.81818181818180	0.854718981972429	0.857142857142857	0.782608695652174
11	80.0	79.50819672131150	0.799820754113053	0.801652892561984	0.788617886178862	85.71428571428570	83.0188679245283	0.861052631578947	0.785714285714286	0.88
12	80.80000000000000	81.81818181818180	0.807034403228906	0.8	0.837209302325581	82.43243243243240	79.36507936507940	0.821830457614404	0.78125	0.806451612903226
13	73.2	72.65306122448980	0.732902151639344	0.760683760683761	0.6953125	72.15189873417720	63.33333333333330	0.709137709137709	0.826086956521739	0.513513513513514
14	75.6	76.62835249042150	0.756755018013381	0.787401574803150	0.746268656716418	79.36507936507940	72.3404255319149	0.773148148148148	0.85	0.629629629629630
15	81.6	81.6	0.816470286885246	0.796875	0.836065573770492	83.54430379746840	78.68852459016400	0.833793103448276	0.75	0.827586206896552
16	79.2	79.36507936507940	0.794042116076014	0.833333333333333	0.757575757575758	80.59701492537310	74.50980392156860	0.792213883677298	0.76	0.730769230769231
17	82.8	82.15767634854770	0.828341013824885	0.860869565217391	0.785714285714286	82.35294117647060	75.0	0.806818181818182	0.75	0.75
18	80.4	78.96995708154510	0.802564102564103	0.814159292035398	0.766666666666667	91.78082191780820	90.32258064516130	0.92015503875969	0.875	0.933333333333333
19	79.2	79.36507936507940	0.799818840579710	0.877192982456140	0.724637681159420	80.28169014084510	63.1578947368421	0.730769230769231	1.0	0.461538461538462
20	78.0	75.77092511013220	0.777295803611593	0.781818181818182	0.735042735042735	71.23287671232880	63.1578947368421	0.695348837209302	0.666666666666667	0.6
21	78.0	79.24528301886790	0.779722079258878	0.801526717557252	0.783582089552239	84.05797101449280	80.0	0.831881533101045	0.814814814814815	0.785714285714286
22	76.8	79.28571428571430	0.763516568697113	0.776223776223776	0.810218978102190	79.41176470588240	68.18181818181820	0.764822134387352	0.681818181818182	0.681818181818182
23	81.2	79.4759825327511	0.812	0.875	0.728	80.82191780821920	69.56521739130430	0.763492063492063	0.888888888888889	0.571428571428571
24	77.2	76.5432098765432	0.772645797324115	0.801724137931035	0.732283464566929	76.71232876712330	70.17543859649120	0.751524390243902	0.8	0.625
25	80.4	79.83539094650210	0.803827444956477	0.815126050420168	0.782258064516129	78.94736842105260	71.42857142857140	0.778533635676493	0.689655172413793	0.740740740740741
26	78.0	78.4313725490196	0.780209933435740	0.763358778625954	0.806451612903226	76.0	71.875	0.766116941529235	0.657142857142857	0.793103448275862
27	82.8	83.5249042145594	0.827420594262295	0.819548872180451	0.8515625	86.36363636363640	81.6326530612245	0.851219512195122	0.833333333333333	0.8
28	82.0	83.14606741573030	0.819508448540707	0.787234042553192	0.880952380952381	85.33333333333330	83.5820895522388	0.882978723404255	0.717948717948718	1.0
29	82.0	83.39483394833950	0.818149785380229	0.795774647887324	0.875968992248062	92.5925925925926	92.3076923076923	0.927172582619339	0.9	0.947368421052632
30	80.80000000000000	81.25	0.808243727598566	0.787878787878788	0.838709677419355	79.48717948717950	75.0	0.801548205489092	0.685714285714286	0.827586206896552
31	72.40000000000000	71.60493827160500	0.728777070882334	0.790909090909091	0.654135338345865	76.5625	72.72727272727270	0.785714285714286	1.0	0.571428571428571
32	80.0	79.50819672131150	0.8	0.815126050420168	0.776	82.85714285714290	71.42857142857140	0.816305469556244	0.652173913043478	0.789473684210526
33	79.60000000000000	79.6812749003984	0.796362704918033	0.813008130081301	0.78125	85.07462686567170	82.14285714285720	0.843920145190563	0.851851851851852	0.793103448275862
34	80.80000000000000	81.25	0.807822802637475	0.806201550387597	0.818897637795276	80.59701492537310	74.50980392156860	0.792213883677298	0.76	0.730769230769231
35	78.0	73.93364928909950	0.772774060730829	0.821052631578947	0.672413793103448	78.48101265822780	56.41025641025640	0.692670537010160	0.846153846153846	0.423076923076923
36	78.8	78.54251012145750	0.787954429083462	0.788617886178862	0.782258064516129	82.66666666666670	79.36507936507940	0.818866571018652	0.862068965517241	0.735294117647059
37	76.4	76.30522088353420	0.764259650470521	0.778688524590164	0.748031496062992	76.0	67.85714285714290	0.738888888888889	0.730769230769231	0.633333333333333
38	77.60000000000000	78.6259541984733	0.775744735490498	0.792307692307692	0.780303030303030	77.5	76.31578947368420	0.774436090225564	0.763157894736842	0.763157894736842
39	80.4	80.7843137254902	0.804487179487179	0.824	0.792307692307692	86.56716417910450	84.7457627118644	0.862612612612613	0.862068965517241	0.833333333333333
40	74.0	73.89558232931730	0.741346153846154	0.773109243697479	0.707692307692308	77.33333333333330	72.1311475409836	0.762354651162791	0.758620689655172	0.6875
41	78.4	80.2919708029197	0.783282130056324	0.743243243243243	0.873015873015873	77.92207792207790	76.05633802816900	0.809267241379310	0.642857142857143	0.931034482758621
42	78.8	80.0	0.790307971014493	0.834645669291339	0.768115942028986	82.85714285714290	80.00000000000000	0.825	0.8	0.8
43	80.80000000000000	81.67938931297710	0.807556610430432	0.816793893129771	0.816793893129771	83.54430379746840	80.0	0.836021505376344	0.764705882352941	0.838709677419355
44	81.6	82.17054263565890	0.817299659404923	0.848	0.796992481203008	85.29411764705880	83.33333333333330	0.865401987353207	0.757575757575758	0.925925925925926
45	76.8	74.78260869565220	0.770363729508197	0.843137254901961	0.671875	73.52941176470590	60.86956521739130	0.71875	1.0	0.4375
46	79.60000000000000	76.7123287671233	0.791305186042028	0.823529411764706	0.717948717948718	81.94444444444440	75.47169811320760	0.800324675324675	0.8	0.714285714285714
47	77.2	74.66666666666670	0.772000000000000	0.84	0.672	79.22077922077920	66.66666666666670	0.744612068965517	0.842105263157895	0.551724137931035
48	80.4	80.63241106719370	0.804	0.796875	0.816	84.61538461538460	82.75862068965520	0.847490347490348	0.8	0.857142857142857
49	78.8	78.36734693877550	0.787890424987199	0.793388429752066	0.774193548387097	82.6086956521739	77.77777777777780	0.829090909090909	0.724137931034483	0.84
50	81.6	78.50467289719630	0.810853015811048	0.8	0.770642201834862	83.33333333333330	72.34042553191490	0.783571428571429	0.894736842105263	0.607142857142857
51	84.0	85.07462686567170	0.842504374878476	0.883720930232558	0.820143884892086	85.71428571428570	84.84848484848490	0.857142857142857	0.903225806451613	0.8
52	81.6	82.03125000000000	0.815860215053764	0.807692307692308	0.833333333333333	73.01587301587300	69.0909090909091	0.735263157894737	0.633333333333333	0.76
53	79.2	78.86178861788620	0.803110156809161	0.708029197080292	0.889908256880734	73.86363636363640	66.66666666666670	0.793333333333333	0.522727272727273	0.92
54	80.4	80.7843137254902	0.804000000000000	0.792307692307692	0.824	83.33333333333330	80.0	0.842439024390244	0.733333333333333	0.88
55	79.2	79.36507936507940	0.792948717948718	0.819672131147541	0.769230769230769	85.71428571428570	80.0	0.851449275362319	0.769230769230769	0.833333333333333
56	82.0	80.17621145374450	0.82	0.892156862745098	0.728	81.94444444444440	68.29268292682930	0.758723404255319	0.875	0.56
57	78.8	80.0	0.789894979508197	0.741258741258741	0.868852459016393	83.56164383561640	81.25	0.836021505376344	0.787878787878788	0.838709677419355
58	80.80000000000000	81.95488721804510	0.806928790983607	0.789855072463768	0.8515625	82.85714285714290	82.3529411764706	0.836228287841191	0.756756756756757	0.903225806451613
59	76.8	76.61290322580650	0.768634733606557	0.791666666666667	0.7421875	74.28571428571430	70.0	0.736145574855252	0.724137931034483	0.677419354838710
60	84.0	84.375	0.839907786885246	0.84375	0.84375	90.66666666666670	86.79245283018870	0.901491365777080	0.851851851851852	0.884615384615385
61	77.60000000000000	76.06837606837610	0.777016988566630	0.735537190082645	0.787610619469027	85.07462686567170	79.16666666666670	0.865424430641822	0.703703703703704	0.904761904761905
62	80.80000000000000	82.97872340425530	0.803707742639040	0.774834437086093	0.893129770992367	82.85714285714290	82.35294117647060	0.848612279226241	0.717948717948718	0.965517241379310
63	82.0	81.92771084337350	0.822354904982024	0.778625954198473	0.864406779661017	90.76923076923080	88.0	0.930232558139535	0.785714285714286	1.0
64	79.60000000000000	77.72925764192140	0.797548172332117	0.872549019607843	0.700787401574803	77.27272727272730	72.72727272727270	0.768382352941177	0.869565217391304	0.625
65	76.4	78.3882783882784	0.761229998072103	0.764285714285714	0.804511278195489	81.25	78.57142857142860	0.812812812812813	0.758620689655172	0.814814814814815
66	78.0	80.28673835125450	0.775799793601651	0.783216783216783	0.823529411764706	75.0	66.66666666666670	0.755928853754941	0.586206896551724	0.772727272727273
67	78.0	77.91164658634540	0.784354091610911	0.843478260869565	0.723880597014925	89.23076923076920	85.71428571428570	0.8825	0.875	0.84
68	76.4	78.3882783882784	0.764784946236559	0.718120805369128	0.862903225806452	67.64705882352940	63.33333333333330	0.702651515151515	0.527777777777778	0.791666666666667
69	83.2	83.72093023255810	0.832000000000000	0.812030075187970	0.864	82.27848101265820	78.7878787878788	0.838275862068966	0.702702702702703	0.896551724137931
70	75.2	75.59055118110240	0.753283362162855	0.721804511278195	0.793388429752066	80.0	76.19047619047620	0.811550151975684	0.685714285714286	0.857142857142857
71	76.0	74.13793103448280	0.762167008196721	0.826923076923077	0.671875	73.68421052631580	62.96296296296300	0.711064129668781	0.809523809523810	0.515151515151515
72	78.8	78.18930041152260	0.788472640964783	0.766129032258065	0.798319327731093	73.84615384615390	71.18644067796610	0.739864864864865	0.677419354838710	0.75
73	77.2	77.47035573122530	0.772005633442161	0.777777777777778	0.771653543307087	86.07594936708860	83.58208955223880	0.859025032938076	0.823529411764706	0.848484848484849
74	81.6	81.30081300813010	0.815924219150026	0.819672131147541	0.806451612903226	84.21052631578950	79.3103448275862	0.832721936903889	0.793103448275862	0.793103448275862
75	80.80000000000000	80.16528925619840	0.808	0.829059829059829	0.776	80.82191780821920	75.0	0.791090629800307	0.84	0.677419354838710
76	77.2	78.81040892193310	0.770291112396376	0.779411764705882	0.796992481203008	79.6875	79.36507936507940	0.796875	0.806451612903226	0.78125
77	78.8	78.88446215139440	0.793795149638803	0.860869565217391	0.727941176470588	81.15942028985510	73.46938775510210	0.784843205574913	0.857142857142857	0.642857142857143
78	84.40000000000000	85.39325842696630	0.844556243550052	0.870229007633588	0.838235294117647	88.88888888888890	86.20689655172410	0.889610389610390	0.833333333333333	0.892857142857143
79	82.0	82.49027237354090	0.820276497695853	0.796992481203008	0.854838709677419	76.0	73.52941176470590	0.807299843014129	0.595238095238095	0.961538461538462
80	79.2	80.30303030303030	0.792000000000000	0.762589928057554	0.848	80.28169014084510	75.86206896551720	0.805134680134680	0.709677419354839	0.814814814814815
81	80.4	78.41409691629950	0.802733499775943	0.855769230769231	0.723577235772358	82.43243243243240	71.11111111111110	0.776442307692308	0.842105263157895	0.615384615384615
82	80.0	81.06060606060610	0.799370826913200	0.810606060606061	0.810606060606061	80.59701492537310	75.47169811320760	0.804761904761905	0.714285714285714	0.8
83	75.6	74.8971193415638	0.758333333333333	0.805309734513274	0.7	81.25	77.77777777777780	0.805882352941176	0.875	0.7
84	78.4	78.57142857142860	0.787738577212262	0.733333333333333	0.846153846153846	78.08219178082190	75.0	0.803140096618358	0.648648648648649	0.888888888888889
85	81.2	81.42292490118580	0.81321673393555	0.780303030303030	0.851239669421488	83.07692307692310	79.24528301886790	0.839939024390244	0.724137931034483	0.875
86	79.2	78.33333333333330	0.792842967799757	0.831858407079646	0.740157480314961	82.6086956521739	75.0	0.797619047619048	0.857142857142857	0.666666666666667
87	75.6	75.6972111553785	0.759650025733402	0.811965811965812	0.708955223880597	80.3030303030303	72.34042553191490	0.777255639097744	0.894736842105263	0.607142857142857
88	80.0	81.06060606060610	0.801805840163934	0.753521126760563	0.877049180327869	84.72222222222220	79.24528301886790	0.864685004436557	0.7	0.913043478260870
89	82.40000000000000	80.7017543859649	0.821669125665533	0.844036697247707	0.773109243697479	85.52631578947370	77.55102040816330	0.825384615384616	0.826086956521739	0.730769230769231
90	78.4	79.54545454545460	0.784498207885305	0.75	0.846774193548387	76.71232876712330	71.1864406779661	0.784583333333333	0.617647058823529	0.84
91	78.8	79.6934865900383	0.787698412698413	0.770370370370370	0.825396825396825	86.48648648648650	82.14285714285710	0.856366459627329	0.821428571428571	0.821428571428571
92	80.0	79.83870967741940	0.799987199180748	0.798387096774194	0.798387096774194	81.53846153846150	80.0	0.816761363636364	0.888888888888889	0.727272727272727
93	82.8	82.73092369477910	0.828148005889508	0.817460317460317	0.837398373983740	88.73239436619720	87.09677419354840	0.885483870967742	0.870967741935484	0.870967741935484
94	81.6	81.74603174603180	0.816080916714679	0.824	0.811023622047244	91.80327868852460	88.8888888888889	0.939024390243902	0.8	1.0
95	76.4	76.49402390438250	0.764536372950820	0.744186046511628	0.786885245901639	85.91549295774650	80.76923076923080	0.863031914893617	0.75	0.875
96	79.60000000000000	80.1556420233463	0.801073195810038	0.735714285714286	0.880341880341880	83.82352941176470	78.43137254901960	0.845893719806763	0.714285714285714	0.869565217391304
97	79.60000000000000	77.13004484304930	0.795204632702193	0.754385964912281	0.788990825688073	78.75	69.0909090909091	0.755431204739961	0.791666666666667	0.612903225806452
98	81.2	81.12449799196790	0.812019969278034	0.808	0.814516129032258	85.52631578947370	82.53968253968250	0.852688172043011	0.8125	0.838709677419355
99	81.2	81.12449799196790	0.812960471522839	0.841666666666667	0.782945736434109	81.81818181818180	78.12500000000000	0.816269284712482	0.757575757575758	0.806451612903226
**Mean**	**79.404**	**79.2876419548158**	**79.4405696273066%**	**80.46477847294940**	**78.58966827757590**	**81.5526306498671**	**76.029371312013**	**80.9079392872451%**	**78.0123521012167%**	**76.6827739787778%**
**Standard Deviation**	2.49734889736126	2.90699404637983	2.46500927890993%	3.946594943011090	6.013867295250980	4.79720474490492	7.18180184663061	5.46350022379216%	9.48323477905909%	13.4675850265288%
**T Test Significance (Female to Male)**						**0.018229380910461**	**0.000006303264316**	**8.40212163022413%**	**0.10661622888339%**	**4.01632521221236%**

	UB_F_FNR	UB_F_TNR	UB_F_FPR	UB_F_TPR	UB_M_Accuracy	UB_M_FScore	UB_M_ROC_AUC	UB_M_Precision	UB_M_Recall	UB_M_FNR	UB_M_TNR	UB_M_FPR	UB_M_TPR
0	27.58620689655170	84.44444444444440	15.55555555555560	72.41379310344830	75.0	74.4186046511628	0.760276654051938	0.853333333333333	0.659793814432990	34.02061855670100	86.07594936708860	13.92405063291140	65.97938144329900
1	16.0	83.6734693877551	16.3265306122449	84.0	80.68181818181820	82.47422680412370	0.809372937293729	0.860215053763441	0.792079207920792	20.79207920792080	82.66666666666670	17.33333333333330	79.20792079207920
2	53.125	100.0	0.0	46.875	75.29411764705880	75.86206896551720	0.756131549609811	0.804878048780488	0.717391304347826	28.26086956521740	79.48717948717950	20.51282051282050	71.73913043478260
3	24.13793103448280	75.60975609756100	24.39024390243900	75.86206896551720	73.33333333333330	74.19354838709680	0.740318335631032	0.811764705882353	0.683168316831683	31.68316831683170	79.74683544303800	20.25316455696200	68.31683168316830
4	21.42857142857140	93.75	6.25	78.57142857142860	77.58620689655170	80.40201005025130	0.771621621621622	0.808080808080808	0.8	20.0	74.32432432432430	25.67567567567570	80.0
5	4.0	76.08695652173910	23.91304347826090	96.0	82.12290502793300	84.31372549019610	0.817672523554877	0.843137254901961	0.843137254901961	15.68627450980390	79.22077922077920	20.77922077922080	84.31372549019610
6	32.25806451612900	82.05128205128200	17.94871794871800	67.74193548387100	80.55555555555560	79.76878612716760	0.805555555555556	0.831325301204819	0.766666666666667	23.33333333333330	84.44444444444440	15.55555555555560	76.66666666666670
7	25.80645161290320	92.3076923076923	7.692307692307690	74.19354838709680	82.22222222222220	84.15841584158420	0.819526256423111	0.841584158415842	0.841584158415842	15.84158415841580	79.74683544303800	20.25316455696200	84.15841584158420
8	55.17241379310340	94.54545454545460	5.454545454545450	44.82758620689660	78.3132530120482	80.43478260869570	0.797105864432597	0.891566265060241	0.732673267326733	26.73267326732670	86.15384615384620	13.84615384615380	73.26732673267330
9	13.63636363636360	80.55555555555560	19.44444444444440	86.36363636363640	78.64583333333330	78.30687830687830	0.791807570633795	0.860465116279070	0.718446601941748	28.15533980582520	86.51685393258430	13.48314606741570	71.84466019417480
10	21.73913043478260	92.6829268292683	7.317073170731710	78.26086956521740	83.87096774193550	83.69565217391300	0.841720779220779	0.895348837209302	0.785714285714286	21.42857142857140	89.77272727272730	10.22727272727270	78.57142857142860
11	12.0	84.21052631578950	15.78947368421050	88.0	78.07486631016040	78.53403141361260	0.781529465718872	0.806451612903226	0.765306122448980	23.46938775510200	79.7752808988764	20.22471910112360	76.53061224489800
12	19.35483870967740	83.72093023255820	16.27906976744190	80.64516129032260	80.11363636363640	82.5870646766169	0.795264259549974	0.805825242718447	0.846938775510204	15.30612244897960	74.35897435897440	25.64102564102560	84.6938775510204
13	48.64864864864870	90.47619047619050	9.523809523809520	51.35135135135140	73.68421052631580	75.67567567567570	0.734615384615385	0.744680851063830	0.769230769230769	23.07692307692310	70.0	30.0	76.92307692307690
14	37.03703703703700	91.66666666666670	8.333333333333330	62.96296296296300	74.33155080213900	77.57009345794390	0.737850467289720	0.775700934579439	0.775700934579439	22.42990654205610	70.0	30.0	77.57009345794390
15	17.24137931034480	84.0	16.0	82.75862068965520	80.7017543859649	82.53968253968250	0.803970223325062	0.8125	0.838709677419355	16.12903225806450	76.92307692307690	23.07692307692310	83.87096774193550
16	26.92307692307690	85.36585365853660	14.63414634146340	73.07692307692310	78.68852459016390	80.59701492537310	0.791166380789022	0.852631578947368	0.764150943396227	23.58490566037740	81.81818181818180	18.18181818181820	76.41509433962260
17	25.0	86.36363636363640	13.63636363636360	75.0	82.96703296703300	83.93782383419690	0.834558823529412	0.890109890109890	0.794117647058824	20.58823529411760	87.5	12.5	79.41176470588240
18	6.666666666666670	90.69767441860470	9.30232558139535	93.33333333333330	75.70621468926550	74.85380116959060	0.757854406130268	0.790123456790123	0.711111111111111	28.88888888888890	80.45977011494250	19.54022988505750	71.11111111111110
19	53.84615384615390	100.0	0.0	46.15384615384620	78.77094972067040	82.24299065420560	0.788379530916844	0.862745098039216	0.785714285714286	21.42857142857140	79.1044776119403	20.8955223880597	78.57142857142860
20	40.0	79.06976744186050	20.93023255813950	60.0	80.7909604519774	80.0	0.807471264367816	0.819277108433735	0.781609195402299	21.83908045977010	83.33333333333330	16.66666666666670	78.16091954022990
21	21.42857142857140	87.8048780487805	12.19512195121950	78.57142857142860	75.69060773480660	79.04761904761910	0.751509433962264	0.798076923076923	0.783018867924528	21.69811320754720	72.0	28.00000000000000	78.30188679245280
22	31.81818181818180	84.78260869565220	15.21739130434780	68.18181818181820	75.82417582417580	81.35593220338980	0.730824140168722	0.793388429752066	0.834782608695652	16.52173913043480	62.68656716417910	37.3134328358209	83.47826086956520
23	42.85714285714290	95.55555555555560	4.444444444444450	57.14285714285710	81.35593220338980	81.9672131147541	0.81784793814433	0.872093023255814	0.773195876288660	22.68041237113400	86.25	13.75000000000000	77.31958762886600
24	37.5	87.8048780487805	12.19512195121950	62.5	77.40112994350280	78.49462365591400	0.774454428754814	0.802197802197802	0.768421052631579	23.15789473684210	78.04878048780490	21.95121951219510	76.84210526315790
25	25.92592592592590	81.63265306122450	18.36734693877550	74.07407407407410	81.03448275862070	82.35294117647060	0.812491632079261	0.855555555555556	0.793814432989691	20.61855670103090	83.11688311688310	16.88311688311690	79.38144329896910
26	20.68965517241380	73.91304347826090	26.08695652173910	79.3103448275862	78.85714285714290	80.6282722513089	0.786513157894737	0.802083333333333	0.810526315789474	18.94736842105260	76.25	23.75	81.05263157894740
27	20.0	90.2439024390244	9.75609756097561	80.0	81.52173913043480	83.9622641509434	0.808582044828000	0.816513761467890	0.864077669902913	13.59223300970870	75.30864197530870	24.69135802469140	86.40776699029130
28	0.0	76.59574468085110	23.40425531914890	100.0	80.57142857142860	83.00000000000000	0.800092764378479	0.813725490196078	0.846938775510204	15.30612244897960	75.32467532467530	24.67532467532470	84.6938775510204
29	5.263157894736840	90.69767441860470	9.30232558139535	94.73684210526320	76.92307692307690	79.79274611398960	0.762820512820513	0.754901960784314	0.846153846153846	15.38461538461540	67.94871794871800	32.05128205128210	84.61538461538460
30	17.24137931034480	77.55102040816330	22.44897959183670	82.75862068965520	81.3953488372093	83.33333333333330	0.810663021189337	0.824742268041237	0.842105263157895	15.78947368421050	77.92207792207790	22.07792207792210	84.21052631578950
31	42.85714285714290	100.0	0.0	57.14285714285710	70.96774193548390	71.27659574468090	0.711154916512059	0.744444444444445	0.683673469387755	31.63265306122450	73.86363636363640	26.13636363636360	68.36734693877550
32	21.05263157894740	84.31372549019610	15.68627450980390	78.94736842105260	78.88888888888890	81.18811881188120	0.792197858235594	0.854166666666667	0.773584905660377	22.64150943396230	81.08108108108110	18.91891891891890	77.35849056603770
33	20.68965517241380	89.47368421052630	10.52631578947370	79.3103448275862	77.59562841530050	78.97435897435900	0.775793650793651	0.802083333333333	0.777777777777778	22.22222222222220	77.38095238095240	22.61904761904760	77.77777777777780
34	26.92307692307690	85.36585365853660	14.63414634146340	73.07692307692310	80.87431693989070	82.92682926829270	0.804938420671335	0.817307692307692	0.841584158415842	15.84158415841580	76.82926829268290	23.17073170731710	84.15841584158420
35	57.69230769230770	96.22641509433960	3.773584905660380	42.30769230769230	77.77777777777780	77.90697674418610	0.779629629629630	0.817073170731707	0.744444444444445	25.55555555555560	81.48148148148150	18.51851851851850	74.44444444444440
36	26.47058823529410	90.2439024390244	9.75609756097561	73.52941176470590	77.14285714285720	78.26086956521740	0.770588235294118	0.765957446808511	0.8	20.0	74.11764705882350	25.88235294117650	80.0
37	36.66666666666670	84.44444444444440	15.55555555555560	63.33333333333330	76.57142857142860	78.75647668393780	0.763547449114459	0.791666666666667	0.783505154639175	21.64948453608250	74.35897435897440	25.64102564102560	78.35051546391750
38	23.68421052631580	78.57142857142860	21.42857142857140	76.31578947368420	77.64705882352940	79.5698924731183	0.775195968645017	0.804347826086957	0.787234042553192	21.27659574468090	76.31578947368420	23.68421052631580	78.72340425531920
39	16.66666666666670	89.1891891891892	10.81081081081080	83.33333333333330	78.14207650273220	79.59183673469390	0.781566265060241	0.8125	0.78	22.0	78.3132530120482	21.68674698795180	78.0
40	31.25	83.72093023255820	16.27906976744190	68.75	72.57142857142860	74.46808510638300	0.727272727272727	0.777777777777778	0.714285714285714	28.57142857142860	74.02597402597400	25.97402597402600	71.42857142857140
41	6.896551724137930	68.75	31.25	93.10344827586210	78.61271676300580	81.77339901477830	0.776519262072708	0.783018867924528	0.855670103092784	14.43298969072160	69.73684210526320	30.26315789473680	85.56701030927830
42	20.0	85.0	15.0	80.0	77.22222222222220	80.0	0.775462962962963	0.845360824742268	0.759259259259259	24.07407407407410	79.16666666666670	20.83333333333330	75.92592592592590
43	16.12903225806450	83.33333333333330	16.66666666666670	83.87096774193550	79.53216374269010	82.23350253807110	0.792323943661972	0.835051546391753	0.81	19.0	77.46478873239440	22.53521126760560	81.0
44	7.407407407407410	80.48780487804880	19.51219512195120	92.5925925925926	80.21978021978020	81.81818181818180	0.809707050645482	0.880434782608696	0.764150943396227	23.58490566037740	85.52631578947370	14.47368421052630	76.41509433962260
45	56.25	100.0	0.0	43.75	78.02197802197800	78.26086956521740	0.781976744186047	0.818181818181818	0.75	25.0	81.3953488372093	18.6046511627907	75.0
46	28.57142857142860	88.63636363636360	11.36363636363640	71.42857142857140	78.65168539325840	77.10843373493980	0.786516853932584	0.831168831168831	0.719101123595506	28.08988764044940	85.39325842696630	14.60674157303370	71.91011235955060
47	44.82758620689660	93.75	6.25	55.17241379310340	76.30057803468210	76.8361581920904	0.769751082251083	0.839506172839506	0.708333333333333	29.16666666666670	83.11688311688310	16.88311688311690	70.83333333333330
48	14.28571428571430	83.78378378378380	16.21621621621620	85.71428571428570	78.91891891891890	80.0	0.788425492033740	0.795918367346939	0.804123711340206	19.58762886597940	77.27272727272730	22.72727272727270	80.41237113402060
49	16.0	81.81818181818180	18.18181818181820	84.0	77.34806629834250	78.53403141361260	0.775129342202513	0.815217391304348	0.757575757575758	24.24242424242420	79.26829268292680	20.73170731707320	75.75757575757580
50	39.28571428571430	96.0	4.0	60.71428571428570	80.81395348837210	80.23952095808380	0.809184642517976	0.779069767441861	0.827160493827161	17.28395061728400	79.12087912087910	20.87912087912090	82.71604938271610
51	20.0	91.42857142857140	8.571428571428570	80.0	83.33333333333330	85.14851485148510	0.834514170040486	0.877551020408163	0.826923076923077	17.30769230769230	84.21052631578950	15.78947368421050	82.6923076923077
52	24.0	71.05263157894740	28.94736842105260	76.0	84.49197860962570	85.57213930348260	0.844347225420216	0.86	0.851485148514852	14.85148514851490	83.72093023255820	16.27906976744190	85.14851485148520
53	8.0	66.66666666666670	33.33333333333330	92.0	82.09876543209880	83.61581920903950	0.818681318681319	0.795698924731183	0.880952380952381	11.90476190476190	75.64102564102560	24.35897435897440	88.09523809523810
54	12.0	80.48780487804880	19.51219512195120	88.0	79.34782608695650	81.0	0.791904761904762	0.81	0.81	19.0	77.38095238095240	22.61904761904760	81.0
55	16.66666666666670	86.95652173913040	13.04347826086960	83.33333333333330	76.66666666666670	79.20792079207920	0.769250382457930	0.833333333333333	0.754716981132076	24.52830188679250	78.37837837837840	21.62162162162160	75.47169811320760
56	44.0	95.74468085106380	4.25531914893617	56.00000000000000	82.02247191011240	82.79569892473120	0.827307692307692	0.895348837209302	0.77	23.0	88.46153846153850	11.53846153846150	77.0
57	16.12903225806450	83.33333333333330	16.66666666666670	83.87096774193550	76.8361581920904	79.60199004975130	0.765141834909277	0.727272727272727	0.879120879120879	12.08791208791210	65.11627906976740	34.88372093023260	87.91208791208790
58	9.67741935483871	76.92307692307690	23.07692307692310	90.32258064516130	80.0	81.81818181818180	0.797043845485033	0.801980198019802	0.835051546391753	16.49484536082470	75.90361445783130	24.09638554216870	83.50515463917530
59	32.25806451612900	79.48717948717950	20.51282051282050	67.74193548387100	77.77777777777780	78.72340425531910	0.779033660414855	0.813186813186813	0.762886597938144	23.71134020618560	79.51807228915660	20.48192771084340	76.28865979381440
60	11.53846153846150	91.83673469387760	8.16326530612245	88.46153846153850	81.14285714285710	83.74384236453200	0.807077625570776	0.841584158415842	0.833333333333333	16.66666666666670	78.08219178082190	21.91780821917810	83.33333333333330
61	9.523809523809520	82.6086956521739	17.39130434782610	90.47619047619050	74.86338797814210	75.26881720430110	0.748566650740564	0.744680851063830	0.760869565217391	23.91304347826090	73.62637362637360	26.37362637362640	76.08695652173910
62	3.448275862068970	73.17073170731710	26.82926829268290	96.55172413793100	80.0	83.17757009345790	0.788838612368024	0.794642857142857	0.872549019607843	12.74509803921570	70.51282051282050	29.48717948717950	87.25490196078430
63	0.0	86.04651162790700	13.95348837209300	100.0	78.91891891891890	80.40201005025130	0.787453183520599	0.776699029126214	0.833333333333333	16.66666666666670	74.15730337078650	25.84269662921350	83.33333333333330
64	37.5	91.17647058823530	8.823529411764710	62.5	80.43478260869570	79.3103448275862	0.806978119455943	0.873417721518987	0.726315789473684	27.36842105263160	88.76404494382020	11.23595505617980	72.63157894736840
65	18.51851851851850	81.08108108108110	18.91891891891890	81.48148148148150	74.73118279569890	78.3410138248848	0.738443396226415	0.765765765765766	0.801886792452830	19.81132075471700	67.5	32.5	80.18867924528300
66	22.72727272727270	73.91304347826090	26.08695652173910	77.27272727272730	79.12087912087910	83.33333333333330	0.776960784313726	0.833333333333333	0.833333333333333	16.66666666666670	72.05882352941180	27.94117647058820	83.33333333333330
67	16.0	92.5	7.5	84.0	74.05405405405410	76.00000000000000	0.749939642684693	0.835164835164835	0.697247706422018	30.27522935779820	80.26315789473690	19.73684210526320	69.72477064220180
68	20.83333333333330	61.36363636363640	38.63636363636360	79.16666666666670	79.67032967032970	82.62910798122070	0.787560975609756	0.778761061946903	0.88	12.0	69.51219512195120	30.48780487804880	88.0
69	10.34482758620690	78.0	22.0	89.65517241379310	83.62573099415210	85.41666666666670	0.83375	0.854166666666667	0.854166666666667	14.58333333333330	81.33333333333330	18.66666666666670	85.41666666666670
70	14.28571428571430	76.59574468085110	23.40425531914890	85.71428571428570	73.14285714285710	75.39267015706810	0.728560188827695	0.734693877551020	0.774193548387097	22.58064516129030	68.29268292682930	31.70731707317070	77.41935483870970
71	48.48484848484850	90.69767441860470	9.30232558139535	51.51515151515150	77.01149425287360	77.52808988764050	0.774550299800133	0.831325301204819	0.726315789473684	27.36842105263160	82.27848101265820	17.72151898734180	72.63157894736840
72	25.0	72.97297297297300	27.02702702702700	75.0	80.54054054054050	80.43478260869570	0.805529576806173	0.795698924731183	0.813186813186813	18.68131868131870	79.7872340425532	20.21276595744680	81.31868131868130
73	15.15151515151520	86.95652173913040	13.04347826086960	84.84848484848480	73.09941520467840	75.26881720430110	0.729483282674772	0.760869565217391	0.744680851063830	25.53191489361700	71.42857142857140	28.57142857142860	74.46808510638300
74	20.68965517241380	87.2340425531915	12.76595744680850	79.3103448275862	80.45977011494250	81.91489361702130	0.803997335109927	0.827956989247312	0.810526315789474	18.94736842105260	79.74683544303800	20.25316455696200	81.05263157894740
75	32.25806451612900	90.47619047619050	9.523809523809520	67.74193548387100	80.7909604519774	81.72043010752690	0.807869776980262	0.826086956521739	0.808510638297872	19.14893617021280	80.72289156626510	19.27710843373490	80.85106382978720
76	21.875	81.25	18.75	78.125	76.34408602150540	78.64077669902910	0.759813628421666	0.771428571428572	0.801980198019802	19.80198019801980	71.76470588235290	28.23529411764710	80.19801980198020
77	35.71428571428570	92.6829268292683	7.317073170731710	64.28571428571430	77.90055248618790	80.1980198019802	0.785958904109589	0.861702127659574	0.75	25.0	82.1917808219178	17.80821917808220	75.0
78	10.71428571428570	88.63636363636360	11.36363636363640	89.28571428571430	82.58426966292130	85.16746411483250	0.826322751322751	0.881188118811881	0.824074074074074	17.59259259259260	82.85714285714290	17.14285714285710	82.4074074074074
79	3.846153846153850	65.3061224489796	34.69387755102040	96.15384615384620	84.57142857142860	85.71428571428570	0.848330241187384	0.890109890109890	0.826530612244898	17.34693877551020	87.01298701298700	12.98701298701300	82.6530612244898
80	18.51851851851850	79.54545454545460	20.45454545454550	81.48148148148150	78.77094972067040	81.55339805825240	0.780423280423281	0.777777777777778	0.857142857142857	14.28571428571430	70.37037037037040	29.62962962962960	85.71428571428570
81	38.46153846153850	93.75	6.25	61.53846153846150	79.54545454545460	80.21978021978020	0.800339292705207	0.858823529411765	0.752577319587629	24.74226804123710	84.81012658227850	15.18987341772150	75.25773195876290
82	20.0	80.95238095238100	19.04761904761900	80.0	79.78142076502730	82.46445497630330	0.794699950811608	0.836538461538462	0.813084112149533	18.69158878504670	77.63157894736840	22.36842105263160	81.30841121495330
83	30.0	91.17647058823530	8.823529411764710	70.0	73.65591397849460	74.07407407407410	0.739534883720930	0.786516853932584	0.7	30.0	77.90697674418610	22.09302325581400	70.0
84	11.11111111111110	71.73913043478260	28.26086956521740	88.88888888888890	78.53107344632770	79.7872340425532	0.784482758620690	0.765306122448980	0.833333333333333	16.66666666666670	73.5632183908046	26.43678160919540	83.33333333333330
85	12.5	80.48780487804880	19.51219512195120	87.5	80.54054054054050	82.0	0.803362230552952	0.796116504854369	0.845360824742268	15.46391752577320	76.13636363636360	23.86363636363640	84.5360824742268
86	33.33333333333330	92.85714285714290	7.142857142857140	66.66666666666670	77.90055248618790	79.16666666666670	0.781234567901235	0.826086956521739	0.76	24.0	80.24691358024690	19.75308641975310	76.0
87	39.28571428571430	94.73684210526320	5.263157894736840	60.71428571428570	73.91304347826090	76.47058823529410	0.739719400096759	0.795918367346939	0.735849056603774	26.41509433962260	74.35897435897440	25.64102564102560	73.58490566037740
88	8.695652173913040	81.63265306122450	18.36734693877550	91.30434782608700	78.08988764044940	81.51658767772510	0.769786472318118	0.767857142857143	0.868686868686869	13.13131313131310	67.08860759493670	32.91139240506330	86.86868686868690
89	26.92307692307690	92.0	8.0	73.07692307692310	81.03448275862070	81.56424581005590	0.812226204699323	0.848837209302326	0.784946236559140	21.50537634408600	83.9506172839506	16.04938271604940	78.49462365591400
90	16.0	72.91666666666670	27.08333333333330	84.0	79.09604519774010	81.95121951219510	0.783216783216783	0.792452830188679	0.848484848484849	15.15151515151520	71.7948717948718	28.20512820512820	84.84848484848480
91	17.85714285714290	89.13043478260870	10.86956521739130	82.14285714285710	75.56818181818180	79.02439024390240	0.746598639455782	0.757009345794393	0.826530612244898	17.34693877551020	66.66666666666670	33.33333333333330	82.6530612244898
92	27.27272727272730	90.625	9.375	72.72727272727270	79.45945945945950	79.78723404255320	0.795066635492167	0.773195876288660	0.824175824175824	17.58241758241760	76.59574468085110	23.40425531914890	82.41758241758240
93	12.90322580645160	90.0	10.0	87.09677419354840	80.44692737430170	81.28342245989310	0.803848075962019	0.8	0.826086956521739	17.39130434782610	78.16091954022990	21.83908045977010	82.6086956521739
94	0.0	87.8048780487805	12.19512195121950	100.0	78.3068783068783	80.19323671497580	0.784191930704354	0.83	0.775700934579439	22.42990654205610	79.26829268292680	20.73170731707320	77.57009345794390
95	12.5	85.1063829787234	14.89361702127660	87.5	72.62569832402240	75.37688442211060	0.722159234063996	0.742574257425743	0.765306122448980	23.46938775510200	67.90123456790120	32.09876543209880	76.53061224489800
96	13.04347826086960	82.22222222222220	17.77777777777780	86.95652173913040	78.02197802197800	80.58252427184470	0.776716634429400	0.741071428571429	0.882978723404255	11.70212765957450	67.04545454545460	32.95454545454550	88.29787234042550
97	38.70967741935480	89.79591836734690	10.20408163265310	61.29032258064520	80.0	79.76190476190480	0.804487179487179	0.744444444444445	0.858974358974359	14.10256410256410	75.0	25.0	85.8974358974359
98	16.12903225806450	86.66666666666670	13.33333333333330	83.87096774193550	79.3103448275862	80.64516129032260	0.792114695340502	0.806451612903226	0.806451612903226	19.35483870967740	77.77777777777780	22.22222222222220	80.64516129032260
99	19.35483870967740	82.6086956521739	17.39130434782610	80.64516129032260	80.92485549132950	82.16216216216220	0.814421768707483	0.873563218390805	0.775510204081633	22.44897959183670	85.33333333333330	14.66666666666670	77.55102040816330
**Mean**	**23.3172260212222**	**85.1331045957123**	**14.8668954042877**	**76.6827739787778**	**78.5695840913929**	**80.1689773524819**	**78.4982919122398%**	**81.4194669957217%**	**79.26309523997670**	**20.7369047600233**	**77.73348858450280**	**22.2665114154972**	**79.2630952399767**
**Standard Deviation**	13.4675850265288	7.88886803820242	7.88886803820242	13.4675850265288	2.85016018457375	2.93574476556375	2.91512331496049%	4.08587394776946%	5.042569353657050	5.04256935365705	5.84950513145123	5.84950513145123	5.04256935365705
**T Test Significance (Female to Male)**	**0.081897049444585**	**0.000000247056858**	**0.000000000000852**	**0.040163252122122**									

Experiment 3.1.1 (Unbalanced training data, no feature selection) - Gaussian Naive Bayes Classifier

		UB_Accuracy	UB_FScore	UB_ROC_AUC	UB_Precision	UB_Recall	UB_F_Accuracy	UB_F_FScore	UB_F_ROC_AUC	UB_F_Precision	UB_F_Recall	UB_F_FNR
0		69.6	60.0	0.696	0.876923076923077	0.456	73.23943661971830	55.81395348837210	0.687804878048780	0.923076923076923	0.4	60.0
1		77.2	71.06598984771570	0.761551314182893	0.875	0.598290598290598	77.46478873239440	55.55555555555560	0.692307692307692	1.0	0.384615384615385	61.53846153846150
2		69.6	63.8095238095238	0.703525641025641	0.8375	0.515384615384615	75.67567567567570	55.00000000000000	0.689655172413793	1.0	0.379310344827586	62.06896551724140
3		72.40000000000000	68.20276497695850	0.748169032341694	0.948717948717949	0.532374100719425	76.38888888888890	56.41025641025640	0.692592592592593	0.916666666666667	0.407407407407407	59.25925925925930
4		73.2	67.6328502415459	0.736552254098361	0.886075949367089	0.546875	76.47058823529410	56.52173913043480	0.691158900836320	0.866666666666667	0.419354838709677	58.06451612903230
5		71.6	66.35071090047390	0.710148181409969	0.760869565217391	0.588235294117647	71.21212121212120	38.70967741935480	0.598412698412698	0.6	0.285714285714286	71.42857142857140
6		68.0	63.30275229357800	0.696658926728586	0.841463414634146	0.507352941176471	67.64705882352940	50.0	0.643859649122807	0.785714285714286	0.366666666666667	63.33333333333330
7		74.0	67.98029556650250	0.737180718263876	0.8625	0.560975609756098	81.15942028985510	66.66666666666670	0.75	1.0	0.5	50.0
8		72.40000000000000	68.4931506849315	0.734946340209498	0.872093023255814	0.563909774436090	73.01587301587300	51.42857142857140	0.673076923076923	1.0	0.346153846153846	65.38461538461540
9		71.2	65.38461538461540	0.714871006977786	0.839506172839506	0.535433070866142	73.52941176470590	43.75000000000000	0.629951690821256	0.777777777777778	0.304347826086957	69.56521739130430
10		72.0	61.95652173913040	0.710576923076923	0.890625	0.475	68.35443037974680	46.80851063829790	0.645779220779221	0.916666666666667	0.314285714285714	68.57142857142860
11		73.6	64.51612903225810	0.722575669944091	0.869565217391304	0.512820512820513	76.25	57.77777777777780	0.699473337722186	0.928571428571429	0.419354838709677	58.06451612903230
12		71.2	64.0	0.721153846153846	0.914285714285714	0.492307692307692	68.75	44.44444444444440	0.634634634634635	0.888888888888889	0.296296296296296	70.37037037037040
13		74.0	67.33668341708540	0.731894284431330	0.8375	0.563025210084034	83.09859154929580	68.42105263157900	0.761775362318841	0.866666666666667	0.565217391304348	43.47826086956520
14		68.0	61.90476190476190	0.697262479871176	0.866666666666667	0.481481481481481	62.85714285714290	40.90909090909090	0.618464052287582	0.9	0.264705882352941	73.52941176470590
15		74.4	63.2183908045977	0.720173395445135	0.887096774193548	0.491071428571429	76.11940298507460	46.66666666666670	0.652173913043478	1.0	0.304347826086957	69.56521739130430
16		66.4	56.70103092783510	0.674038461538462	0.859375	0.423076923076923	75.0	56.00000000000000	0.687363834422658	0.875	0.411764705882353	58.82352941176470
17		72.8	64.21052631578950	0.716807909604520	0.847222222222222	0.516949152542373	78.66666666666670	52.94117647058820	0.677696078431373	0.9	0.375	62.5
18		69.20000000000000	62.4390243902439	0.704288649203903	0.876712328767123	0.484848484848485	71.25	56.60377358490570	0.697368421052632	1.0	0.394736842105263	60.52631578947370
19		76.4	73.30316742081450	0.776376737004632	0.931034482758621	0.604477611940299	78.26086956521740	66.66666666666670	0.746120689655172	0.9375	0.517241379310345	48.27586206896550
20		70.40000000000000	65.74074074074070	0.715635241951032	0.855421686746988	0.533834586466165	75.75757575757580	61.90476190476190	0.715099715099715	0.866666666666667	0.481481481481481	51.85185185185190
21		68.0	61.53846153846150	0.697906602254428	0.876712328767123	0.474074074074074	68.11594202898550	45.00000000000000	0.630172413793103	0.818181818181818	0.310344827586207	68.96551724137930
22		73.2	65.64102564102560	0.727138831967213	0.876712328767123	0.524590163934426	78.46153846153850	50.00000000000000	0.663888888888889	0.875	0.35	65.0
23		65.60000000000000	55.67010309278350	0.663847780126850	0.830769230769231	0.418604651162791	69.56521739130430	43.24324324324320	0.630662020905923	0.888888888888889	0.285714285714286	71.42857142857140
24		72.8	64.58333333333330	0.715002573340196	0.815789473684211	0.534482758620690	70.4225352112676	40.0	0.618265993265993	0.875	0.259259259259259	74.07407407407410
25		74.4	70.64220183486240	0.750320512820513	0.875	0.592307692307692	65.21739130434780	42.85714285714290	0.627111486486487	0.9	0.28125	71.875
26		66.4	59.61538461538460	0.684081527347781	0.861111111111111	0.455882352941176	73.33333333333330	52.38095238095240	0.667916041979011	0.846153846153846	0.379310344827586	62.06896551724140
27		74.0	67.98029556650250	0.740000000000000	0.884615384615385	0.552	76.62337662337660	62.5	0.720196353436185	0.882352941176471	0.483870967741936	51.61290322580650
28		67.60000000000000	60.48780487804880	0.686221053306819	0.837837837837838	0.473282442748092	70.76923076923080	61.22448979591840	0.711647727272727	0.9375	0.454545454545455	54.54545454545450
29		72.40000000000000	65.32663316582920	0.718207444423089	0.833333333333333	0.537190082644628	75.67567567567570	57.14285714285710	0.692546583850932	0.857142857142857	0.428571428571429	57.14285714285710
30		78.4	72.44897959183670	0.774332306111967	0.910256410256410	0.601694915254237	80.82191780821920	63.1578947368421	0.729583333333333	0.923076923076923	0.48	52.0
31		75.6	69.95073891625620	0.750752770837338	0.865853658536585	0.586776859504132	78.66666666666670	65.21739130434780	0.738888888888889	0.9375	0.5	50.0
32		73.6	70.53571428571430	0.745710429920956	0.868131868131868	0.593984962406015	75.38461538461540	61.90476190476190	0.724137931034483	1.0	0.448275862068966	55.17241379310340
33		75.2	68.36734693877550	0.740285640761709	0.8375	0.577586206896552	75.0	48.64864864864870	0.650588235294118	0.75	0.36	64.0
34		68.8	60.60606060606060	0.695368056890256	0.869565217391304	0.465116279069767	68.4931506849315	46.51162790697670	0.644054878048781	0.909090909090909	0.3125	68.75
35		75.2	70.1923076923077	0.753392217101895	0.890243902439024	0.579365079365079	75.38461538461540	52.94117647058820	0.680000000000000	1.0	0.36	64.0
36		71.6	63.21243523316060	0.7109375	0.859154929577465	0.5	69.86301369863010	35.29411764705880	0.600241545893720	0.857142857142857	0.222222222222222	77.77777777777780
37		69.20000000000000	62.4390243902439	0.692000000000000	0.8	0.512	75.0	55.81395348837210	0.683035714285714	0.8	0.428571428571429	57.14285714285710
38		72.40000000000000	61.87845303867400	0.710772984078069	0.888888888888889	0.474576271186441	70.4225352112676	36.36363636363640	0.604273504273504	0.857142857142857	0.230769230769231	76.92307692307690
39		70.40000000000000	64.42307692307690	0.706868958453364	0.827160493827161	0.527559055118110	71.23287671232880	43.24324324324320	0.626409017713366	0.8	0.296296296296296	70.37037037037040
40		68.4	58.63874345549740	0.685931899641577	0.861538461538462	0.444444444444444	70.3125	34.48275862068970	0.596500530222694	0.833333333333333	0.217391304347826	78.26086956521740
41		67.2	60.57692307692310	0.673387096774194	0.768292682926829	0.5	71.42857142857140	42.10526315789470	0.618148148148148	0.727272727272727	0.296296296296296	70.37037037037040
42		70.0	61.53846153846150	0.701804915514593	0.869565217391304	0.476190476190476	72.6027397260274	56.52173913043480	0.690929878048781	0.928571428571429	0.40625	59.375
43		72.40000000000000	67.90697674418600	0.749076414544040	0.960526315789474	0.525179856115108	71.76470588235290	45.45454545454550	0.641899766899767	0.909090909090909	0.303030303030303	69.6969696969697
44		72.0	67.88990825688070	0.731186941713258	0.870588235294118	0.556390977443609	72.15189873417720	52.17391304347830	0.662298387096774	0.8	0.387096774193548	61.29032258064520
45		73.2	65.28497409326430	0.728538505857500	0.9	0.512195121951220	75.0	52.94117647058820	0.675000000000000	0.9	0.375	62.5
46		72.0	66.98113207547170	0.734752959341225	0.910256410256410	0.529850746268657	77.14285714285720	55.55555555555560	0.692307692307692	1.0	0.384615384615385	61.53846153846150
47		73.6	64.8936170212766	0.723089775721355	0.859154929577465	0.521367521367521	74.66666666666670	45.71428571428570	0.64	0.8	0.32	68.0
48		67.60000000000000	62.32558139534880	0.695852981073574	0.858974358974359	0.489051094890511	73.13432835820900	43.75000000000000	0.634205426356589	0.875	0.291666666666667	70.83333333333330
49		73.6	66.3265306122449	0.736	0.915492957746479	0.52	69.1358024691358	48.97959183673470	0.649689440993789	0.857142857142857	0.342857142857143	65.71428571428570
50		69.20000000000000	61.69154228855720	0.695314000384098	0.837837837837838	0.488188976377953	71.05263157894740	50.0	0.660511363636364	0.916666666666667	0.34375	65.625
51		70.40000000000000	64.76190476190480	0.709846883208405	0.839506172839506	0.527131782945737	72.36842105263160	51.16279069767440	0.657740278796772	0.785714285714286	0.379310344827586	62.06896551724140
52		74.8	66.66666666666670	0.729220779220779	0.797468354430380	0.572727272727273	79.22077922077920	57.89473684210530	0.700864779874214	0.785714285714286	0.458333333333333	54.16666666666670
53		74.0	67.00507614213200	0.736796619934703	0.891891891891892	0.536585365853659	71.01449275362320	47.36842105263160	0.648519163763066	0.9	0.321428571428571	67.85714285714290
54		69.20000000000000	64.51612903225810	0.699948681762782	0.813953488372093	0.534351145038168	66.19718309859160	47.82608695652170	0.640350877192982	0.846153846153846	0.333333333333333	66.66666666666670
55		74.8	67.6923076923077	0.744670635682735	0.916666666666667	0.536585365853659	76.92307692307690	50.0	0.666666666666667	1.0	0.333333333333333	66.66666666666670
56		74.4	64.0449438202247	0.715819283456270	0.770270270270270	0.548076923076923	79.48717948717950	55.55555555555560	0.689814814814815	0.833333333333333	0.416666666666667	58.33333333333330
57		72.40000000000000	61.45251396648040	0.707218219248585	0.873015873015873	0.474137931034483	73.75	46.15384615384620	0.641483516483516	0.818181818181818	0.321428571428571	67.85714285714290
58		72.40000000000000	66.98564593301440	0.739642305712815	0.933333333333333	0.522388059701493	66.66666666666670	46.15384615384620	0.640466531440162	0.9	0.310344827586207	68.96551724137930
59		70.0	62.68656716417910	0.698476702508961	0.818181818181818	0.508064516129032	73.91304347826090	50.0	0.661449016100179	0.9	0.346153846153846	65.38461538461540
60		72.40000000000000	64.61538461538460	0.733974358974359	0.969230769230769	0.484615384615385	72.72727272727270	40.0	0.625	1.0	0.25	75.0
61		70.40000000000000	60.63829787234040	0.688566827697263	0.780821917808219	0.495652173913044	70.1492537313433	47.36842105263160	0.632380952380952	0.692307692307692	0.36	64.0
62		73.6	67.32673267326730	0.739261250880225	0.906666666666667	0.535433070866142	76.92307692307690	55.00000000000000	0.689655172413793	1.0	0.379310344827586	62.06896551724140
63		72.40000000000000	60.57142857142860	0.700375258799172	0.841269841269841	0.473214285714286	71.23287671232880	40.0	0.613338788870704	0.777777777777778	0.269230769230769	73.07692307692310
64		71.2	61.29032258064520	0.694852941176471	0.791666666666667	0.5	81.42857142857140	43.47826086956520	0.638888888888889	1.0	0.277777777777778	72.22222222222220
65		71.6	65.3658536585366	0.730512177880599	0.930555555555556	0.503759398496241	72.72727272727270	46.15384615384620	0.65	1.0	0.3	70.0
66		69.6	63.46153846153850	0.705689909551607	0.857142857142857	0.503816793893130	66.66666666666670	50.00000000000000	0.657915057915058	0.923076923076923	0.342857142857143	65.71428571428570
67		69.6	60.82474226804120	0.688141025641026	0.797297297297297	0.491666666666667	73.33333333333330	47.36842105263160	0.645833333333333	0.818181818181818	0.333333333333333	66.66666666666670
68		66.8	60.66350710900470	0.680772443930339	0.820512820512821	0.481203007518797	68.0	50.00000000000000	0.646103896103896	0.8	0.363636363636364	63.63636363636360
69		71.6	63.58974358974360	0.711129610655738	0.849315068493151	0.508196721311475	69.44444444444440	52.17391304347830	0.6625	0.857142857142857	0.375	62.5
70		72.8	66.66666666666670	0.731131169579412	0.883116883116883	0.535433070866142	77.46478873239440	65.21739130434780	0.737804878048781	0.9375	0.5	50.0
71		68.4	57.75401069518720	0.686059907834101	0.885245901639344	0.428571428571429	75.0	48.64864864864860	0.656462585034014	0.9	0.333333333333333	66.66666666666670
72		74.8	66.66666666666670	0.733172302737520	0.851351351351351	0.547826086956522	78.57142857142860	48.27586206896550	0.659090909090909	1.0	0.318181818181818	68.18181818181820
73		73.6	64.1304347826087	0.719484702093398	0.855072463768116	0.513043478260870	78.26086956521740	65.11627906976740	0.737804878048781	0.933333333333333	0.5	50.0
74		73.2	64.17112299465240	0.717575913535769	0.845070422535211	0.517241379310345	76.38888888888890	51.42857142857140	0.669361702127660	0.9	0.36	64.0
75		74.0	65.24064171122990	0.732686270741239	0.924242424242424	0.504132231404959	76.5625	48.27586206896550	0.655038759689922	0.875	0.333333333333333	66.66666666666670
76		76.8	70.40816326530610	0.759381615241517	0.896103896103896	0.579831932773109	80.51948051948050	65.11627906976740	0.741379310344828	1.0	0.482758620689655	51.72413793103450
77		68.0	62.61682242990650	0.690228556753981	0.817073170731707	0.507575757575758	65.78947368421050	48.0	0.628851540616247	0.75	0.352941176470588	64.70588235294120
78		70.0	60.73298429319370	0.691666666666667	0.816901408450704	0.483333333333333	76.11940298507460	50.00000000000000	0.659595959595960	0.8	0.363636363636364	63.63636363636360
79		72.8	63.44086021505380	0.713265568708183	0.842857142857143	0.508620689655172	77.21518987341770	50.0	0.660227272727273	0.75	0.375	62.5
80		70.8	67.55555555555560	0.717338217338217	0.826086956521739	0.571428571428571	74.28571428571430	57.14285714285710	0.690476190476191	0.857142857142857	0.428571428571429	57.14285714285710
81		69.6	58.69565217391310	0.696	0.915254237288136	0.432	70.88607594936710	46.51162790697680	0.651515151515152	1.0	0.303030303030303	69.6969696969697
82		67.60000000000000	61.24401913875600	0.691392177045806	0.853333333333333	0.477611940298507	70.3125	53.65853658536590	0.675369458128079	0.916666666666667	0.379310344827586	62.06896551724140
83		70.40000000000000	63.72549019607840	0.712500000000000	0.878378378378378	0.5	75.38461538461540	57.89473684210530	0.698717948717949	0.916666666666667	0.423076923076923	57.69230769230770
84		70.8	60.96256684491980	0.693450849202265	0.802816901408451	0.491379310344828	72.72727272727270	50.0	0.660576923076923	0.9	0.346153846153846	65.38461538461540
85		72.8	66.99029126213590	0.728	0.851851851851852	0.552	67.6056338028169	46.51162790697670	0.636290322580645	0.833333333333333	0.322580645161290	67.74193548387100
86		71.2	62.88659793814430	0.710253456221198	0.871428571428571	0.491935483870968	83.60655737704920	58.33333333333330	0.705882352941176	1.0	0.411764705882353	58.82352941176470
87		71.2	62.5	0.700019278966647	0.8	0.512820512820513	77.77777777777780	60.8695652173913	0.713725490196078	0.875	0.466666666666667	53.33333333333330
88		68.0	61.53846153846150	0.687820512820513	0.820512820512821	0.492307692307692	69.51219512195120	48.9795918367347	0.645220588235294	0.8	0.352941176470588	64.70588235294120
89		70.40000000000000	57.95454545454550	0.678246753246753	0.772727272727273	0.463636363636364	77.02702702702700	48.48484848484850	0.656666666666667	0.888888888888889	0.333333333333333	66.66666666666670
90		68.8	59.375	0.688000000000000	0.850746268656716	0.456	66.19718309859160	42.85714285714290	0.627804487179487	0.9	0.28125	71.875
91		72.8	66.66666666666670	0.729518689196109	0.871794871794872	0.539682539682540	68.65671641791050	51.16279069767440	0.656306306306306	0.846153846153846	0.366666666666667	63.33333333333330
92		73.6	67.32673267326730	0.731813524590164	0.85	0.557377049180328	76.71232876712330	54.05405405405410	0.679166666666667	0.833333333333333	0.4	60.0
93		68.4	58.63874345549740	0.68795211574163	0.875	0.440944881889764	64.28571428571430	32.43243243243240	0.587500000000000	0.857142857142857	0.2	80.0
94		70.40000000000000	58.88888888888890	0.693910256410256	0.883333333333333	0.441666666666667	66.66666666666670	45.83333333333330	0.633887043189369	0.846153846153846	0.314285714285714	68.57142857142860
95		72.0	63.91752577319590	0.713562688192709	0.849315068493151	0.512396694214876	72.05882352941180	42.42424242424240	0.634615384615385	1.0	0.269230769230769	73.07692307692310
96		69.6	60.0	0.694124423963134	0.863636363636364	0.459677419354839	67.14285714285710	51.06382978723400	0.671428571428572	1.0	0.342857142857143	65.71428571428570
97		69.6	60.00000000000000	0.692337238333013	0.850746268656716	0.463414634146342	70.51282051282050	43.90243902439030	0.629166666666667	0.818181818181818	0.3	70.0
98		73.6	71.30434782608700	0.747181964573269	0.863157894736842	0.607407407407407	77.04918032786890	66.66666666666670	0.740659340659341	0.875	0.538461538461538	46.15384615384620
99		73.6	64.51612903225810	0.721307256819352	0.857142857142857	0.517241379310345	85.91549295774650	68.75000000000000	0.761904761904762	1.0	0.523809523809524	47.61904761904760
	**Mean**	**71.528**	**64.1781829695749**	**71.4927676862469%**	**85.980083693597800**	**51.3718009517937%**	**73.45403064711590**	**51.11395003689920**	**66.66135050095020**	**88.6581965420201%**	**36.3721123720638%**	**63.6278876279362**
	**Standard Deviation**	2.61302410044281	3.64866375940236	2.33242899631119%	4.115016966037420	4.35794679736022%	4.57460952475229	7.92592396319850	3.905199191385900	8.10565813114857%	7.58016733287887%	7.58016733287887
	**T Test Significance (Female to Male)**						**0.020062503508727**	**0.000000000000000**	**0.000000000091619**	**5.32036205988618%**	**0.000000000000000**	**0.000000000000000**

	UB_F_TNR	UB_F_FPR	UB_F_TPR	UB_M_Accuracy	UB_M_FScore	UB_M_ROC_AUC	UB_M_Precision	UB_M_Recall	UB_M_FNR	UB_M_TNR	UB_M_FPR	UB_M_TPR
0	97.5609756097561	2.439024390243900	40.0	68.15642458100560	61.22448979591840	0.695175438596491	0.865384615384615	0.473684210526316	52.63157894736840	91.66666666666670	8.333333333333330	47.36842105263160
1	100.0	0.0	38.46153846153850	77.09497206703910	74.53416149068320	0.772852147852148	0.857142857142857	0.659340659340659	34.06593406593410	88.63636363636360	11.36363636363640	65.93406593406590
2	100.0	0.0	37.93103448275860	67.04545454545460	65.88235294117650	0.690561056105611	0.811594202898551	0.554455445544555	44.55445544554460	82.66666666666670	17.33333333333330	55.44554455445550
3	97.77777777777780	2.222222222222220	40.74074074074070	70.78651685393260	70.7865168539326	0.758522727272727	0.954545454545455	0.5625	43.75	95.45454545454550	4.545454545454550	56.25
4	96.29629629629630	3.703703703703700	41.93548387096770	71.51515151515150	70.80745341614910	0.742343844754397	0.890625	0.587628865979382	41.23711340206190	89.70588235294120	10.29411764705880	58.76288659793820
5	91.11111111111110	8.88888888888889	28.57142857142860	71.73913043478260	71.1111111111111	0.721879449454200	0.780487804878049	0.653061224489796	34.69387755102040	79.06976744186050	20.93023255813950	65.3061224489796
6	92.10526315789470	7.894736842105260	36.66666666666670	68.13186813186810	66.66666666666670	0.707795431976167	0.852941176470588	0.547169811320755	45.28301886792450	86.8421052631579	13.15789473684210	54.71698113207550
7	100.0	0.0	50.0	71.27071823204420	68.29268292682930	0.723183603338243	0.835820895522388	0.577319587628866	42.2680412371134	86.90476190476190	13.09523809523810	57.73195876288660
8	100.0	0.0	34.61538461538460	72.19251336898400	71.7391304347826	0.739661214953271	0.857142857142857	0.616822429906542	38.31775700934580	86.25	13.75000000000000	61.6822429906542
9	95.55555555555560	4.444444444444450	30.43478260869570	70.32967032967030	69.31818181818180	0.722756410256410	0.847222222222222	0.586538461538462	41.34615384615390	85.8974358974359	14.10256410256410	58.65384615384620
10	97.72727272727270	2.272727272727270	31.42857142857140	73.68421052631580	67.15328467153290	0.735704514363885	0.884615384615385	0.541176470588235	45.88235294117650	93.02325581395350	6.976744186046510	54.11764705882350
11	97.95918367346940	2.040816326530610	41.93548387096770	72.35294117647060	66.66666666666670	0.725636766334441	0.854545454545455	0.546511627906977	45.34883720930230	90.47619047619050	9.523809523809520	54.65116279069770
12	97.2972972972973	2.702702702702700	29.62962962962960	72.04301075268820	68.29268292682930	0.741724178266464	0.918032786885246	0.543689320388350	45.63106796116510	93.97590361445780	6.024096385542170	54.36893203883500
13	95.83333333333330	4.166666666666670	56.52173913043480	70.39106145251400	67.0807453416149	0.714984939759036	0.830769230769231	0.5625	43.75	86.74698795180720	13.25301204819280	56.25
14	97.22222222222220	2.777777777777780	26.47058823529410	70.0	67.46987951807230	0.720265697455822	0.861538461538462	0.554455445544555	44.55445544554460	88.60759493670890	11.39240506329110	55.44554455445550
15	100.0	0.0	30.43478260869570	73.77049180327870	66.66666666666670	0.732428878795123	0.872727272727273	0.539325842696629	46.06741573033710	92.5531914893617	7.446808510638300	53.93258426966290
16	96.29629629629630	3.703703703703700	41.17647058823530	61.72839506172840	56.94444444444440	0.660511363636364	0.854166666666667	0.427083333333333	57.29166666666670	89.39393939393940	10.60606060606060	42.70833333333330
17	98.0392156862745	1.960784313725490	37.5	70.28571428571430	66.66666666666670	0.714867349619123	0.838709677419355	0.553191489361702	44.68085106382980	87.65432098765430	12.34567901234570	55.31914893617020
18	100.0	0.0	39.47368421052630	68.23529411764710	64.47368421052630	0.701427771556551	0.844827586206897	0.521276595744681	47.87234042553190	88.1578947368421	11.84210526315790	52.12765957446810
19	97.5	2.5	51.72413793103450	75.69060773480660	75.0	0.781390977443609	0.929577464788732	0.628571428571429	37.14285714285710	93.42105263157900	6.578947368421050	62.85714285714290
20	94.87179487179490	5.128205128205130	48.14814814814810	68.47826086956520	66.66666666666670	0.709482341557813	0.852941176470588	0.547169811320755	45.28301886792450	87.17948717948720	12.82051282051280	54.71698113207550
21	95.0	5.0	31.03448275862070	67.95580110497240	65.47619047619050	0.712767295597484	0.887096774193548	0.518867924528302	48.11320754716980	90.66666666666670	9.333333333333330	51.88679245283020
22	97.77777777777780	2.222222222222220	35.0	71.35135135135140	68.26347305389220	0.731218993621545	0.876923076923077	0.558823529411765	44.11764705882350	90.36144578313250	9.63855421686747	55.88235294117650
23	97.5609756097561	2.439024390243900	28.57142857142860	64.08839779005530	58.59872611464970	0.665222772277228	0.821428571428571	0.455445544554455	54.45544554455450	87.5	12.5	45.54455445544560
24	97.72727272727270	2.272727272727270	25.92592592592590	73.74301675977650	70.06369426751590	0.736766541822722	0.808823529411765	0.617977528089888	38.20224719101120	85.55555555555560	14.44444444444440	61.79775280898880
25	97.2972972972973	2.702702702702700	28.125	77.90055248618790	77.27272727272730	0.786697811654783	0.871794871794872	0.693877551020408	30.61224489795920	87.95180722891570	12.04819277108430	69.38775510204080
26	95.65217391304350	4.347826086956520	37.93103448275860	63.42857142857140	61.44578313253010	0.679494227597581	0.864406779661017	0.476635514018692	52.33644859813080	88.23529411764710	11.76470588235290	47.66355140186920
27	95.65217391304350	4.347826086956520	48.38709677419360	72.83236994219650	69.67741935483870	0.742930245084837	0.885245901639344	0.574468085106383	42.5531914893617	91.13924050632910	8.860759493670890	57.44680851063830
28	96.875	3.125	45.45454545454550	66.48648648648650	60.25641025641030	0.676577527562749	0.810344827586207	0.479591836734694	52.04081632653060	87.35632183908050	12.64367816091950	47.95918367346940
29	95.65217391304350	4.347826086956520	42.85714285714290	71.02272727272730	67.51592356687900	0.718681176318176	0.828125	0.569892473118280	43.01075268817200	86.74698795180720	13.25301204819280	56.98924731182800
30	97.91666666666670	2.083333333333330	48.0	77.40112994350280	74.68354430379750	0.781490015360983	0.907692307692308	0.634408602150538	36.55913978494620	92.85714285714290	7.142857142857140	63.44086021505380
31	97.77777777777780	2.222222222222220	50.0	74.28571428571430	71.33757961783440	0.748168498168498	0.848484848484849	0.615384615384615	38.46153846153850	88.09523809523810	11.90476190476190	61.53846153846150
32	100.0	0.0	44.82758620689660	72.97297297297300	72.52747252747250	0.743233618233618	0.846153846153846	0.634615384615385	36.53846153846150	85.18518518518520	14.81481481481480	63.46153846153850
33	94.11764705882350	5.88235294117647	36.0	75.28735632183910	72.95597484276730	0.758440354825897	0.852941176470588	0.637362637362637	36.26373626373630	87.95180722891570	12.04819277108430	63.73626373626370
34	97.5609756097561	2.439024390243900	31.25	68.92655367231640	64.51612903225810	0.707731958762887	0.862068965517241	0.515463917525773	48.45360824742270	90.0	10.0	51.54639175257730
35	100.0	0.0	36.0	75.13513513513510	73.56321839080460	0.763260254596888	0.876712328767123	0.633663366336634	36.63366336633660	89.28571428571430	10.71428571428570	63.36633663366340
36	97.82608695652170	2.173913043478260	22.22222222222220	72.31638418079100	69.18238993710690	0.734595635430039	0.859375	0.578947368421053	42.10526315789470	89.02439024390250	10.97560975609760	57.89473684210530
37	93.75	6.25	42.85714285714290	66.66666666666670	64.19753086419750	0.683625652697818	0.8	0.536082474226804	46.39175257731960	83.11688311688310	16.88311688311690	53.60824742268040
38	97.77777777777780	2.222222222222220	23.07692307692310	73.18435754189940	67.56756756756760	0.737256371814093	0.892857142857143	0.543478260869565	45.65217391304350	93.10344827586210	6.896551724137930	54.34782608695650
39	95.65217391304350	4.347826086956520	29.62962962962960	70.05649717514130	69.00584795321640	0.717077922077922	0.830985915492958	0.59	41.0	84.4155844155844	15.58441558441560	59.0
40	97.5609756097561	2.439024390243900	21.73913043478260	67.74193548387100	62.96296296296300	0.699380044449643	0.864406779661017	0.495145631067961	50.48543689320390	90.36144578313250	9.63855421686747	49.51456310679610
41	94.0	6.0	29.62962962962960	65.3179190751445	64.70588235294120	0.669669669669670	0.774647887323944	0.555555555555556	44.44444444444440	78.37837837837840	21.62162162162160	55.55555555555560
42	97.5609756097561	2.439024390243900	40.625	68.92655367231640	63.08724832214770	0.701807228915663	0.854545454545455	0.5	50.0	90.36144578313250	9.63855421686747	50.0
43	98.07692307692310	1.923076923076920	30.30303030303030	72.72727272727270	73.6842105263158	0.780220658778382	0.969230769230769	0.594339622641509	40.56603773584910	96.61016949152540	3.389830508474580	59.43396226415090
44	93.75	6.25	38.70967741935480	71.9298245614035	72.09302325581400	0.745950554134698	0.885714285714286	0.607843137254902	39.21568627450980	88.40579710144930	11.59420289855070	60.78431372549020
45	97.5	2.5	37.5	72.58064516129030	67.9245283018868	0.738244514106583	0.9	0.545454545454545	45.45454545454550	93.10344827586210	6.896551724137930	54.54545454545450
46	100.0	0.0	38.46153846153850	70.0	69.31818181818180	0.733796296296296	0.897058823529412	0.564814814814815	43.51851851851850	90.27777777777780	9.722222222222220	56.48148148148150
47	96.0	4.0	32.0	73.14285714285710	69.28104575163400	0.739850707176532	0.868852459016393	0.576086956521739	42.39130434782610	90.36144578313250	9.63855421686747	57.60869565217390
48	97.67441860465120	2.325581395348840	29.16666666666670	65.57377049180330	65.57377049180330	0.694058154235146	0.857142857142857	0.530973451327434	46.90265486725660	85.71428571428570	14.28571428571430	53.09734513274340
49	95.65217391304350	4.347826086956520	34.28571428571430	75.7396449704142	72.10884353741500	0.769127988748242	0.929824561403509	0.588888888888889	41.11111111111110	94.9367088607595	5.063291139240510	58.88888888888890
50	97.72727272727270	2.272727272727270	34.375	68.39080459770120	64.96815286624200	0.698800799467022	0.822580645161290	0.536842105263158	46.31578947368420	86.07594936708860	13.92405063291140	53.68421052631580
51	93.61702127659580	6.382978723404260	37.93103448275860	69.54022988505750	68.26347305389220	0.717432432432432	0.850746268656716	0.57	43.0	86.48648648648650	13.51351351351350	57.00000000000000
52	94.33962264150940	5.660377358490570	45.83333333333330	72.83236994219650	68.87417218543050	0.727612937717188	0.8	0.604651162790698	39.53488372093020	85.0574712643678	14.94252873563220	60.46511627906980
53	97.5609756097561	2.439024390243900	32.14285714285710	75.13812154696130	71.69811320754720	0.759302325581395	0.890625	0.6	40.0	91.86046511627910	8.13953488372093	60.0
54	94.73684210526320	5.263157894736840	33.33333333333330	70.39106145251400	69.00584795321640	0.714600655076846	0.808219178082192	0.602040816326531	39.79591836734690	82.71604938271610	17.28395061728400	60.20408163265310
55	100.0	0.0	33.33333333333330	73.83720930232560	71.69811320754720	0.757401315789474	0.904761904761905	0.59375	40.625	92.10526315789470	7.894736842105260	59.375
56	96.29629629629630	3.703703703703700	41.66666666666670	72.09302325581400	66.19718309859160	0.712228260869565	0.758064516129032	0.5875	41.25	83.69565217391310	16.30434782608700	58.75
57	96.15384615384620	3.846153846153850	32.14285714285710	71.76470588235290	65.71428571428570	0.724778270509978	0.884615384615385	0.522727272727273	47.72727272727270	92.6829268292683	7.317073170731710	52.27272727272730
58	97.05882352941180	2.941176470588240	31.03448275862070	74.33155080213900	71.76470588235300	0.766085946573752	0.938461538461539	0.580952380952381	41.90476190476190	95.1219512195122	4.878048780487810	58.0952380952381
59	97.67441860465120	2.325581395348840	34.61538461538460	68.50828729281770	65.45454545454540	0.697196951069585	0.805970149253731	0.551020408163265	44.89795918367350	84.33734939759040	15.66265060240960	55.10204081632650
60	100.0	0.0	25.0	72.28260869565220	69.09090909090910	0.756047411707789	0.966101694915254	0.537735849056604	46.22641509433960	97.43589743589740	2.564102564102560	53.77358490566040
61	90.47619047619050	9.523809523809520	36.0	70.49180327868850	64.0	0.702150537634409	0.8	0.533333333333333	46.66666666666670	87.09677419354840	12.90322580645160	53.33333333333330
62	100.0	0.0	37.93103448275860	72.09302325581400	70.37037037037040	0.743519029233315	0.890625	0.581632653061225	41.83673469387760	90.54054054054050	9.45945945945946	58.16326530612250
63	95.74468085106380	4.25531914893617	26.92307692307690	72.88135593220340	65.71428571428570	0.723485816509072	0.851851851851852	0.534883720930233	46.51162790697670	91.20879120879120	8.791208791208790	53.48837209302330
64	100.0	0.0	27.77777777777780	67.22222222222220	63.80368098159510	0.681547619047619	0.776119402985075	0.541666666666667	45.83333333333330	82.14285714285710	17.85714285714290	54.16666666666670
65	100.0	0.0	30.0	71.09826589595380	69.87951807228910	0.745839112343967	0.920634920634921	0.563106796116505	43.68932038834950	92.85714285714290	7.142857142857140	56.31067961165050
66	97.2972972972973	2.702702702702700	34.28571428571430	70.78651685393260	67.5	0.720274390243903	0.84375	0.5625	43.75	87.8048780487805	12.19512195121950	56.25
67	95.83333333333330	4.166666666666670	33.33333333333330	68.0	64.1025641025641	0.689548911618148	0.793650793650794	0.537634408602151	46.23655913978490	84.14634146341460	15.85365853658540	53.76344086021510
68	92.85714285714290	7.142857142857140	36.36363636363640	66.28571428571430	63.80368098159510	0.686666666666667	0.825396825396825	0.52	48.0	85.33333333333330	14.66666666666670	52.0
69	95.0	5.0	37.5	72.47191011235960	67.11409395973150	0.726641414141414	0.847457627118644	0.555555555555556	44.44444444444440	89.77272727272730	10.22727272727270	55.55555555555560
70	97.5609756097561	2.439024390243900	50.0	70.94972067039110	67.0886075949367	0.724415388483782	0.868852459016393	0.546391752577320	45.36082474226800	90.2439024390244	9.75609756097561	54.63917525773200
71	97.95918367346940	2.040816326530610	33.33333333333330	65.51724137931040	60.0	0.687272727272727	0.882352941176471	0.454545454545455	54.54545454545450	92.0	8.0	45.45454545454550
72	100.0	0.0	31.81818181818180	73.33333333333330	70.0	0.737856878012607	0.835820895522388	0.602150537634409	39.78494623655910	87.35632183908050	12.64367816091950	60.21505376344090
73	97.5609756097561	2.439024390243900	50.0	71.8232044198895	63.82978723404260	0.710748349229641	0.833333333333333	0.517241379310345	48.27586206896550	90.42553191489360	9.574468085106380	51.72413793103450
74	97.87234042553190	2.127659574468090	36.0	71.91011235955060	67.10526315789470	0.722748515851964	0.836065573770492	0.560439560439560	43.95604395604400	88.50574712643680	11.49425287356320	56.04395604395600
75	97.67441860465120	2.325581395348840	33.33333333333330	73.11827956989250	68.35443037974680	0.746744186046512	0.931034482758621	0.54	46.0	95.34883720930230	4.651162790697680	54.0
76	100.0	0.0	48.27586206896550	75.14450867052020	71.89542483660130	0.757362784471218	0.873015873015873	0.611111111111111	38.88888888888890	90.36144578313250	9.63855421686747	61.11111111111110
77	90.47619047619050	9.523809523809520	35.29411764705880	68.96551724137930	67.07317073170730	0.708243823845328	0.833333333333333	0.561224489795918	43.87755102040820	85.52631578947370	14.47368421052630	56.12244897959180
78	95.55555555555560	4.444444444444450	36.36363636363640	67.75956284153010	62.8930817610063	0.690396158463385	0.819672131147541	0.510204081632653	48.97959183673470	87.05882352941180	12.94117647058820	51.02040816326530
79	94.54545454545460	5.454545454545450	37.5	70.76023391812870	66.66666666666670	0.721106219042378	0.862068965517241	0.543478260869565	45.65217391304350	89.87341772151900	10.12658227848100	54.34782608695650
80	95.23809523809520	4.761904761904760	42.85714285714290	69.44444444444440	69.94535519125680	0.711428571428571	0.820512820512821	0.609523809523810	39.04761904761910	81.33333333333330	18.66666666666670	60.95238095238100
81	100.0	0.0	30.30303030303030	69.00584795321640	62.4113475177305	0.707484865162355	0.897959183673469	0.478260869565217	52.17391304347830	93.67088607594940	6.329113924050630	47.82608695652170
82	97.14285714285710	2.857142857142860	37.93103448275860	66.66666666666670	63.09523809523810	0.690652557319224	0.841269841269841	0.504761904761905	49.52380952380950	87.65432098765430	12.34567901234570	50.47619047619050
83	97.43589743589740	2.564102564102560	42.30769230769230	68.64864864864870	65.06024096385540	0.710232668566002	0.870967741935484	0.519230769230769	48.07692307692310	90.12345679012350	9.876543209876540	51.92307692307690
84	97.5	2.5	34.61538461538460	70.1086956521739	63.57615894039740	0.697517730496454	0.786885245901639	0.533333333333333	46.66666666666670	86.17021276595740	13.82978723404260	53.33333333333330
85	95.0	5.0	32.25806451612900	74.86033519553070	72.39263803680980	0.755006257822278	0.855072463768116	0.627659574468085	37.23404255319150	88.23529411764710	11.76470588235290	62.76595744680850
86	100.0	0.0	41.17647058823530	67.1957671957672	63.52941176470590	0.697458399817643	0.857142857142857	0.504672897196262	49.53271028037380	89.02439024390250	10.97560975609760	50.46728971962620
87	96.07843137254900	3.921568627450980	46.66666666666670	68.04733727810650	63.01369863013700	0.685099523409027	0.779661016949153	0.528735632183908	47.12643678160920	84.14634146341460	15.85365853658540	52.87356321839080
88	93.75	6.25	35.29411764705880	67.26190476190480	65.40880503144650	0.694444444444444	0.825396825396825	0.541666666666667	45.83333333333330	84.72222222222220	15.27777777777780	54.16666666666670
89	98.0	2.0	33.33333333333330	67.61363636363640	60.13986013986010	0.672222222222222	0.754385964912281	0.5	50.0	84.44444444444440	15.55555555555560	50.0
90	97.43589743589740	2.564102564102560	28.125	69.83240223463690	64.00000000000000	0.705738934733683	0.842105263157895	0.516129032258065	48.38709677419360	89.53488372093020	10.46511627906980	51.61290322580650
91	94.5945945945946	5.405405405405410	36.66666666666670	74.31693989071040	70.80745341614910	0.750897988505747	0.876923076923077	0.59375	40.625	90.80459770114940	9.195402298850570	59.375
92	95.83333333333330	4.166666666666670	40.0	72.31638418079100	70.3030303030303	0.736469072164949	0.852941176470588	0.597938144329897	40.20618556701030	87.5	12.5	59.79381443298970
93	97.5	2.5	20.0	70.0	64.93506493506490	0.715563284064091	0.877192982456140	0.515463917525773	48.45360824742270	91.56626506024100	8.433734939759040	51.54639175257730
94	95.34883720930230	4.651162790697680	31.42857142857140	72.09302325581400	63.63636363636360	0.718323191345504	0.893617021276596	0.494117647058824	50.58823529411760	94.25287356321840	5.747126436781610	49.41176470588240
95	100.0	0.0	26.92307692307690	71.97802197802200	68.32298136645960	0.726255293405929	0.833333333333333	0.578947368421053	42.10526315789470	87.35632183908050	12.64367816091950	57.89473684210530
96	100.0	0.0	34.28571428571430	70.55555555555560	62.93706293706290	0.703358439313496	0.833333333333333	0.505617977528090	49.43820224719100	90.10989010989010	9.89010989010989	50.56179775280900
97	95.83333333333330	4.166666666666670	30.0	69.18604651162790	64.42953020134230	0.707431604736627	0.857142857142857	0.516129032258065	48.38709677419360	89.87341772151900	10.12658227848100	51.61290322580650
98	94.28571428571430	5.714285714285710	53.84615384615390	72.4867724867725	72.3404255319149	0.743176605504587	0.860759493670886	0.623853211009174	37.61467889908260	86.25	13.75000000000000	62.38532110091740
99	100.0	0.0	52.38095238095240	68.71508379888270	63.63636363636360	0.698370927318296	0.830508474576271	0.515789473684211	48.42105263157890	88.09523809523810	11.90476190476190	51.57894736842110
**Mean**	**96.9505886298365**	**3.04941137016347**	**36.3721123720638**	**70.73700886594590**	**67.3013659362366**	**72.19568721009870**	**85.53053665455330**	**55.6774622972002%**	**44.3225377027998**	**88.71391212299720**	**11.28608787700280**	**55.6774622972002**
**Standard Deviation**	2.25891621160645	2.25891621160645	7.58016733287888	3.08222498517235	3.89636643842101	2.787609492185110	4.251572421935250	4.82750183077209%	4.82750183077209	3.65886074735686	3.658860747356870	4.82750183077208
**T Test Significance (Female to Male)**	**0.000000000056051**	**0.000000000000000**	**0.000000000000000**									

Summary of Results for Experiment 3.1.2

	**Random Forest Classifier**			**Logistic Regression Classifier**			**Support Vector Machine**			**Gaussian Naive Bayes**		
	Mean	Standard Deviation	T Test Significance (Female to Male)	Mean	Standard Deviation	T Test Significance (Female to Male)	Mean	Standard Deviation	T Test Significance (Female to Male)	Mean	Standard Deviation	T Test Significance (Female to Male)
**All Accuracy**	81.66	2.33		74.53	1.96		83.30	1.75		74.75	1.97	
**All FScore**	76.52	3.09		65.63	2.88		80.07	2.25		62.80	3.26	
**All ROC_AUC**	80.22%	2.41%		72.40%	1.97%		82.70%	1.85%		71.67%	1.97%	
**All Precision**	84.80%	3.38%		78.52%	4.30%		82.71%	3.84%		85.28%	3.80%	
**All Recall**	69.87%	4.42%		56.57%	3.85%		77.91%	4.71%		49.83%	3.77%	
Females Accuracy	84.75	3.03	0.00	77.71	2.42	0.00	89.03	2.23	0.00	78.47	2.35	0.00
Females FScore	71.41	5.74	0.00	51.69	5.87	0.00	82.18	3.72	0.00	51.55	5.38	0.00
Females ROC_AUC	78.47%	3.76%	0.13	67.10%	2.86%	0.00	86.87%	3.01%	0.00	67.12%	2.59%	0.00
Females Precision	85.44%	6.07%	0.88	82.22%	8.04%	0.00	84.22%	5.64%	0.14	85.13%	7.00%	0.37
Females Recall	61.70%	7.03%	0.00	38.12%	6.09%	0.00	80.88%	6.90%	0.00	37.19%	5.02%	0.00
**Females FNR**	38.30	7.03	0.00	61.88	6.09	0.00	19.12	6.90	0.00	62.81	5.02	0.00
**Females TNR**	95.24	2.03	0.00	96.09	2.04	0.00	92.86	3.00	0.00	97.05	1.48	0.00
**Females FPR**	4.76	2.03	0.00	3.91	2.04	0.00	7.14	3.00	0.00	2.95	1.48	0.00
**Females TPR**	61.70	7.03	0.00	38.12	6.09	0.00	80.88	6.90	0.00	37.19	5.02	0.00
**Males Accuracy**	78.58	3.05		71.35	3.22		77.56	2.93		71.04	3.22	
**Males FScore**	79.10	3.21		71.86	3.41		78.78	3.10		68.20	3.96	
**Males ROC_AUC**	79.07%	2.93%		71.89%	3.07%		77.80%	2.95%		72.57%	2.82%	
**Males Precision**	84.49%	3.92%		77.47%	4.44%		81.90%	4.62%		85.37%	4.05%	
**Males Recall**	74.59%	4.93%		67.34%	5.33%		76.24%	5.26%		57.01%	5.09%	
**Males FNR**	25.41	4.93		32.66	5.33		23.76	5.26		42.99	5.09	
**Males TNR**	83.55	4.16		76.44	4.82		79.36	5.69		88.12	3.48	
**Males FPR**	16.45	4.16		23.56	4.82		20.64	5.69		11.88	3.48	
**Males TPR**	74.59	4.93		67.34	5.33		76.24	5.26		57.01	5.09	
	**Random Forest Results**			**Logistic Regression Results**			**Support Vector Machine Results**			**Gaussian Naive Bayes Results**		
Averaged over 100 experiments	**Sex Disparity (Male Mean - Female Mean)**		**T Test P Value**	**Sex Disparity (Male Mean - Female Mean)**		**T Test P Value**	**Sex Disparity (Male Mean - Female Mean)**		**T Test P Value**	**Sex Disparity (Male Mean - Female Mean)**		**T Test P Value**
**Accuacy**	-6.17		0.00	-6.36		0.00	-11.47		0.00	-7.43		0.00
**FScore**	7.69		0.00	20.17		0.00	-3.40		0.00	16.65		0.00
**ROC_AUC**	0.60%		0.13	4.79%		0.00	-9.06%		0.00	5.45%		0.00
**Precision**	-0.94%		0.88	-4.75%		0.00	-2.32%		0.14	0.24%		0.37
**Recall**	12.88%		0.00	29.22%		0.00	-4.64%		0.00	19.82%		0.00
**False Negative Rate**	-12.88		0.00	-29.22		0.00	4.64		0.00	-19.82		0.00
**True Negative Rate**	-11.69		0.00	-19.65		0.00	-13.49		0.00	-8.93		0.00
**False Positive Rate**	11.69		0.00	19.65		0.00	13.49		0.00	8.93		0.00
**True Positive Rate**	12.88		0.00	29.22		0.00	-4.64		0.00	19.82		0.00

3.1.2 Balanced training data, no feature selection - Random Forest Classifier

	B_Accuracy	B_FScore	B_ROC_AUC	B_Precision	B_Recall	B_F_Accuracy	B_F_FScore	B_F_ROC_AUC	B_F_Precision	B_F_Recall	B_F_FNR
0	82.9608938547486	78.13620071684590	0.8146670904179250	0.8790322580645160	0.7032258064516130	83.79888268156430	68.81720430107530	0.7643083117630140	0.8888888888888890	0.5614035087719300	43.859649122807000
1	80.44692737430170	73.68421052631580	0.7845178077736220	0.7967479674796750	0.6853146853146850	83.24022346368710	68.75000000000000	0.7695555555555560	0.7857142857142860	0.6111111111111110	38.88888888888890
2	82.68156424581010	76.86567164179100	0.8094353155972870	0.8174603174603170	0.7253521126760560	87.15083798882680	75.26881720430110	0.8103474094040130	0.875	0.660377358490566	33.9622641509434
3	82.12290502793300	77.30496453900710	0.8073327629369080	0.872	0.6942675159235670	83.79888268156430	69.47368421052630	0.7717741935483870	0.825	0.6	40.0
4	79.05027932960890	72.72727272727270	0.7739556962025320	0.8547008547008550	0.6329113924050630	79.88826815642460	64.0	0.7326086956521740	0.8888888888888890	0.5	50.0
5	83.79888268156430	79.28571428571430	0.8258917032825020	0.8283582089552240	0.7602739726027400	85.47486033519550	73.46938775510210	0.7990469208211140	0.8372093023255810	0.6545454545454550	34.54545454545460
6	80.72625698324020	76.45051194539250	0.7969303797468350	0.8296296296296300	0.7088607594936710	86.59217877094970	75.00000000000000	0.8118448637316560	0.8372093023255810	0.6792452830188680	32.075471698113200
7	78.77094972067040	75.16339869281050	0.7823842302878600	0.8455882352941180	0.6764705882352940	78.2122905027933	64.22018348623850	0.7299440298507460	0.8333333333333330	0.5223880597014930	47.76119402985070
8	84.07821229050280	80.27681660899650	0.829625756567481	0.8787878787878790	0.7388535031847130	88.26815642458100	79.6116504854369	0.839157319528329	0.8913043478260870	0.7192982456140350	28.07017543859650
9	82.68156424581010	78.91156462585040	0.8185095777032320	0.9133858267716540	0.6946107784431140	88.8268156424581	80.0	0.834816384180791	0.975609756097561	0.6779661016949150	32.20338983050850
10	81.56424581005590	73.17073170731710	0.7844527565457800	0.8737864077669900	0.6293706293706290	83.24022346368710	65.11627906976740	0.7443096735549570	0.8484848484848490	0.5283018867924530	47.16981132075470
11	83.79888268156430	80.27210884353740	0.8284177215189870	0.8676470588235290	0.7468354430379750	89.3854748603352	80.41237113402060	0.8451111111111110	0.9069767441860470	0.7222222222222220	27.77777777777780
12	84.63687150837990	78.43137254901960	0.8279029230356660	0.8130081300813010	0.7575757575757580	86.03351955307260	65.75342465753430	0.7676258992805760	0.7272727272727270	0.6	40.0
13	83.79888268156430	80.0	0.8278654551251390	0.8467153284671530	0.7581699346405230	87.70949720670390	79.62962962962960	0.8373249299719890	0.8958333333333330	0.7166666666666670	28.333333333333300
14	79.3296089385475	72.18045112781960	0.7719870748952240	0.8347826086956520	0.6357615894039740	79.88826815642460	58.13953488372090	0.7029048029910840	0.8620689655172410	0.43859649122807000	56.14035087719300
15	83.5195530726257	79.1519434628975	0.8220505040927910	0.9032258064516130	0.7044025157232700	87.15083798882680	75.78947368421050	0.8111436950146630	0.9	0.6545454545454550	34.54545454545460
16	82.9608938547486	79.3220338983051	0.8217616249361270	0.8181818181818180	0.7697368421052630	87.15083798882680	78.0952380952381	0.8233537093637120	0.9318181818181820	0.6721311475409840	32.78688524590160
17	78.77094972067040	73.42657342657340	0.7747784810126580	0.8203125	0.6645569620253160	83.79888268156430	71.2871287128713	0.7800847457627120	0.8571428571428570	0.6101694915254240	38.983050847457600
18	80.44692737430170	74.82014388489210	0.7910176531671860	0.7761194029850750	0.7222222222222220	83.24022346368710	69.38775510204080	0.76831005984611	0.85	0.5862068965517240	41.37931034482760
19	82.12290502793300	75.57251908396950	0.8003725855476030	0.8181818181818180	0.7021276595744680	86.59217877094970	75.51020408163270	0.8121700879765400	0.8604651162790700	0.6727272727272730	32.72727272727270
20	78.49162011173190	73.35640138408300	0.772911906575273	0.7969924812030080	0.6794871794871800	79.88826815642460	64.0	0.7330532212885160	0.8	0.5333333333333330	46.666666666666700
21	80.16759776536310	76.72131147540980	0.7958892958892960	0.8602941176470590	0.6923076923076920	82.68156424581010	69.30693069306930	0.7664565826330530	0.8536585365853660	0.5833333333333330	41.66666666666670
22	80.44692737430170	73.48484848484850	0.7824411547477600	0.8434782608695650	0.6510067114093960	83.24022346368710	68.08510638297870	0.7582633053221290	0.9411764705882350	0.5333333333333330	46.666666666666700
23	85.19553072625700	81.66089965397920	0.8409703076971830	0.8939393939393940	0.7515923566878980	87.15083798882680	78.0952380952381	0.8286548874323170	0.8723404255319150	0.7068965517241380	29.310344827586200
24	78.77094972067040	75.48387096774190	0.78328173374613	0.841726618705036	0.6842105263157900	77.09497206703910	66.11570247933890	0.7353938445487740	0.8	0.5633802816901410	43.66197183098590
25	83.5195530726257	78.06691449814130	0.8187923839332290	0.8267716535433070	0.7394366197183100	85.47486033519550	72.3404255319149	0.7889296187683290	0.8717948717948720	0.6181818181818180	38.18181818181820
26	86.87150837988830	83.15412186379930	0.8561776061776060	0.8854961832061070	0.7837837837837840	91.62011173184360	84.21052631578950	0.876531862745098	0.9090909090909090	0.7843137254901960	21.568627450980400
27	80.72625698324020	75.09025270758120	0.7915770206480200	0.8125	0.697986577181208	85.47486033519550	71.1111111111111	0.7863817401960780	0.8205128205128210	0.6274509803921570	37.254901960784300
28	82.9608938547486	78.74564459930310	0.8175949367088610	0.875968992248062	0.7151898734177220	88.26815642458100	74.6987951807229	0.8226430976430980	0.7948717948717950	0.7045454545454550	29.545454545454500
29	82.40223463687150	77.41935483870970	0.8097845284351820	0.8307692307692310	0.7248322147651010	89.3854748603352	80.80808080808080	0.8475073313782990	0.9090909090909090	0.7272727272727270	27.27272727272730
30	83.5195530726257	77.90262172284640	0.8160700439390020	0.859504132231405	0.7123287671232880	82.68156424581010	66.66666666666670	0.755037037037037	0.7948717948717950	0.5740740740740740	42.592592592592600
31	83.5195530726257	78.54545454545460	0.8187948350071740	0.8852459016393440	0.7058823529411770	83.24022346368710	64.28571428571430	0.7399303452453060	0.84375	0.5192307692307690	48.07692307692310
32	77.6536312849162	71.63120567375890	0.7618484925907000	0.7769230769230770	0.6644736842105260	80.44692737430170	62.365591397849500	0.7285185185185190	0.7435897435897440	0.5370370370370370	46.2962962962963
33	77.6536312849162	71.63120567375890	0.762333680983534	0.8211382113821140	0.6352201257861640	78.77094972067040	57.77777777777780	0.7007407407407410	0.7222222222222220	0.48148148148148100	51.85185185185190
34	81.00558659217880	75.7142857142857	0.7969759627810800	0.7910447761194030	0.726027397260274	84.35754189944130	72.00000000000000	0.7889082462253190	0.8181818181818180	0.6428571428571430	35.714285714285700
35	78.77094972067040	73.79310344827590	0.7761075949367090	0.8106060606060610	0.6772151898734180	81.00558659217880	66.00000000000000	0.7463276836158190	0.8048780487804880	0.559322033898305	44.06779661016950
36	76.81564245810060	67.70428015564200	0.741923076923077	0.8130841121495330	0.58	82.12290502793300	61.904761904761900	0.7263779527559050	0.8125	0.5	50.0
37	79.88826815642460	74.64788732394370	0.7857954545454550	0.8548387096774190	0.6625	85.47486033519550	74.50980392156870	0.8067653890824620	0.8260869565217390	0.6785714285714290	32.142857142857100
38	79.05027932960890	74.0484429065744	0.7795029101777570	0.8492063492063490	0.656441717791411	85.47486033519550	75.47169811320750	0.8081232492997200	0.8695652173913040	0.6666666666666670	33.33333333333330
39	81.56424581005590	78.43137254901960	0.809145880574452	0.8333333333333330	0.7407407407407410	82.68156424581010	68.68686868686870	0.7687683284457480	0.7727272727272730	0.6181818181818180	38.18181818181820
40	82.40223463687150	78.64406779661020	0.8153488668194550	0.8226950354609930	0.7532467532467530	85.47486033519550	76.78571428571430	0.8168596636338570	0.86	0.6935483870967740	30.64516129032260
41	79.05027932960890	70.58823529411760	0.7639423076923080	0.8571428571428570	0.6	83.79888268156430	65.88235294117650	0.7464809384164220	0.9333333333333330	0.509090909090909	49.09090909090910
42	79.88826815642460	74.10071942446040	0.7829905467552380	0.8174603174603170	0.6776315789473690	82.12290502793300	65.95744680851060	0.7465802222855510	0.8611111111111110	0.5344827586206900	46.55172413793100
43	83.5195530726257	76.6798418972332	0.810540184453228	0.8434782608695650	0.7028985507246380	86.59217877094970	70.73170731707320	0.7805337519623230	0.8787878787878790	0.5918367346938780	40.816326530612200
44	85.19553072625700	81.13879003558720	0.838181702950217	0.9193548387096770	0.7261146496815290	85.47486033519550	72.34042553191490	0.7859505320678750	0.918918918918919	0.5964912280701750	40.35087719298250
45	77.93296089385480	71.48014440433210	0.7635820612496450	0.8389830508474580	0.6226415094339620	79.88826815642460	62.5	0.7247151986662960	0.8571428571428570	0.4918032786885250	50.81967213114750
46	83.5195530726257	79.15194346289750	0.8225517484083570	0.8484848484848490	0.7417218543046360	90.5027932960894	82.4742268041237	0.8555718475073310	0.9523809523809520	0.7272727272727270	27.27272727272730
47	83.5195530726257	79.44250871080140	0.8237906273620560	0.912	0.7037037037037040	87.15083798882680	77.22772277227720	0.8180084745762710	0.9285714285714290	0.6610169491525420	33.89830508474580
48	78.2122905027933	73.10344827586210	0.7710380448475690	0.828125	0.654320987654321	83.24022346368710	65.9090909090909	0.7485185185185190	0.8529411764705880	0.5370370370370370	46.2962962962963
49	80.44692737430170	75.86206896551720	0.7931481305900570	0.8396946564885500	0.6918238993710690	82.68156424581010	71.55963302752290	0.7793513957307060	0.8478260869565220	0.6190476190476190	38.095238095238100
50	77.09497206703910	74.05063291139240	0.7672389529974670	0.8068965517241380	0.6842105263157900	76.53631284916200	64.40677966101690	0.7265057113187950	0.8260869565217390	0.5277777777777780	47.22222222222220
51	79.88826815642460	74.10071942446040	0.7834493670886080	0.8583333333333330	0.6518987341772150	84.35754189944130	75.0	0.8000399786780380	0.9333333333333330	0.6268656716417910	37.3134328358209
52	79.60893854748600	73.6462093862816	0.7802848101265820	0.8571428571428570	0.6455696202531650	79.88826815642460	62.500000000000000	0.7250706214689270	0.8108108108108110	0.5084745762711860	49.152542372881400
53	76.25698324022350	68.86446886446890	0.746240186650692	0.8392857142857140	0.5838509316770190	79.3296089385475	59.34065934065930	0.7081932773109240	0.8709677419354840	0.45	55.00000000000000
54	82.40223463687150	76.22641509433960	0.8030128205128210	0.8782608695652170	0.6733333333333330	89.3854748603352	79.56989247311830	0.837151841868823	0.925	0.6981132075471700	30.18867924528300
55	81.00558659217880	75.7142857142857	0.7951374543143180	0.848	0.6838709677419360	84.91620111731840	70.32967032967030	0.7780772686433060	0.8421052631578950	0.6037735849056600	39.62264150943400
56	82.9608938547486	78.13620071684590	0.8155325776307760	0.8384615384615390	0.7315436241610740	87.70949720670390	78.43137254901960	0.8383703703703700	0.8333333333333330	0.7407407407407410	25.925925925925900
57	83.79888268156430	79.8611111111111	0.8266327665660260	0.8646616541353380	0.7419354838709680	86.03351955307260	73.11827956989250	0.7913763066202090	0.918918918918919	0.6071428571428570	39.285714285714300
58	82.12290502793300	77.62237762237760	0.8094293272864700	0.8951612903225810	0.6851851851851850	84.35754189944130	69.56521739130440	0.7684066724187520	0.9142857142857140	0.5614035087719300	43.859649122807000
59	83.79888268156430	80.66666666666670	0.8306036646586340	0.9029850746268660	0.7289156626506020	88.8268156424581	79.16666666666670	0.8311556329849010	0.95	0.6785714285714290	32.142857142857100
60	79.05027932960890	74.74747474747480	0.7812687202446640	0.8161764705882350	0.6894409937888200	81.56424581005590	69.72477064220180	0.767104542966612	0.8260869565217390	0.6031746031746030	39.682539682539700
61	82.9608938547486	79.46127946127950	0.8210963037465430	0.8872180451127820	0.7195121951219510	85.47486033519550	67.5	0.767162471395881	0.7941176470588240	0.5869565217391310	41.30434782608700
62	85.47486033519550	81.81818181818180	0.8433285509325680	0.8796992481203010	0.7647058823529410	88.26815642458100	80.37383177570090	0.8397471519866630	0.9347826086956520	0.7049180327868850	29.508196721311500
63	81.84357541899440	76.01476014760150	0.8008529335745670	0.824	0.7054794520547950	88.26815642458100	74.07407407407410	0.8149831649831650	0.8108108108108110	0.6818181818181820	31.818181818181800
64	81.56424581005590	75.55555555555560	0.7966855424384940	0.8571428571428570	0.6754966887417220	82.68156424581010	69.30693069306930	0.7674435028248590	0.8333333333333330	0.5932203389830510	40.67796610169490
65	83.5195530726257	78.85304659498210	0.8216216216216220	0.8396946564885500	0.7432432432432430	84.91620111731840	70.32967032967030	0.7824754901960780	0.8	0.6274509803921570	37.254901960784300
66	81.28491620111730	75.81227436823100	0.7975868725868730	0.813953488372093	0.7094594594594590	88.26815642458100	76.92307692307690	0.8302197802197800	0.8333333333333330	0.7142857142857140	28.57142857142860
67	78.2122905027933	72.3404255319149	0.7679908978856550	0.8292682926829270	0.6415094339622640	85.47486033519550	71.73913043478260	0.7824477351916380	0.9166666666666670	0.5892857142857140	41.07142857142860
68	83.24022346368710	79.45205479452060	0.8224694104560620	0.8467153284671530	0.7483870967741940	83.24022346368710	72.72727272727270	0.7883926109732560	0.8333333333333330	0.6451612903225810	35.483870967741900
69	82.12290502793300	76.81159420289860	0.8049896710630860	0.8760330578512400	0.6838709677419360	84.35754189944130	68.18181818181820	0.7689922480620160	0.7894736842105260	0.6	40.0
70	78.49162011173190	72.0	0.768022942611782	0.8389830508474580	0.6305732484076430	79.3296089385475	62.626262626262600	0.7268478573482890	0.7380952380952380	0.543859649122807	45.614035087719300
71	80.44692737430170	76.35135135135140	0.795044191919192	0.8308823529411770	0.70625	83.79888268156430	73.39449541284400	0.7939705473742710	0.8333333333333330	0.6557377049180330	34.42622950819670
72	80.16759776536310	73.60594795539030	0.7824373223055050	0.8048780487804880	0.678082191780822	83.79888268156430	71.84466019417480	0.7859076660017100	0.8222222222222220	0.6379310344827590	36.206896551724100
73	82.68156424581010	77.20588235294120	0.8094916344916350	0.8467741935483870	0.7094594594594590	85.47486033519550	69.76744186046510	0.7767441860465120	0.8333333333333330	0.6	40.0
74	84.91620111731840	80.98591549295770	0.8364849833147940	0.8914728682170540	0.7419354838709680	87.70949720670390	79.24528301886790	0.8331932773109240	0.9130434782608700	0.7	30.0
75	84.35754189944130	80.82191780821920	0.8361645419222250	0.8251748251748250	0.7919463087248320	88.26815642458100	80.37383177570090	0.8458962667426620	0.8775510204081630	0.7413793103448280	25.862068965517200
76	84.07821229050280	78.96678966789670	0.8228474706182930	0.8991596638655460	0.7039473684210530	87.15083798882680	73.5632183908046	0.7981004901960780	0.8888888888888890	0.6274509803921570	37.254901960784300
77	77.93296089385480	71.88612099644130	0.7641965877260000	0.7952755905511810	0.6558441558441560	81.00558659217880	65.3061224489796	0.7482962962962960	0.7272727272727270	0.5925925925925930	40.74074074074070
78	83.79888268156430	79.86111111111110	0.8264240506329110	0.8846153846153850	0.7278481012658230	86.59217877094970	73.33333333333330	0.8000919117647060	0.8461538461538460	0.6470588235294120	35.294117647058800
79	84.35754189944130	78.94736842105260	0.8235576923076920	0.9051724137931030	0.7	87.70949720670390	78.0	0.8221751412429380	0.9512195121951220	0.6610169491525420	33.89830508474580
80	82.40223463687150	78.49829351535840	0.8141342980451890	0.8394160583941610	0.7371794871794870	86.59217877094970	70.0	0.7817914351095130	0.8235294117647060	0.6086956521739130	39.130434782608700
81	80.72625698324020	75.78947368421050	0.7961846443711730	0.9	0.6545454545454550	85.47486033519550	73.46938775510210	0.7938159019663720	0.9	0.6206896551724140	37.93103448275860
82	81.84357541899440	76.36363636363640	0.8017883993985350	0.8467741935483870	0.695364238410596	86.03351955307260	71.26436781609200	0.789403453689168	0.8157894736842110	0.6326530612244900	36.734693877551000
83	83.24022346368710	78.26086956521740	0.8170095761828010	0.9230769230769230	0.6792452830188680	86.03351955307260	73.68421052631580	0.7916666666666670	1.0	0.5833333333333330	41.66666666666670
84	85.75418994413410	81.31868131868130	0.8411538461538460	0.9024390243902440	0.74	87.70949720670390	76.08695652173910	0.8084349593495940	0.9722222222222220	0.625	37.5
85	81.28491620111730	74.90636704119850	0.7942423056859680	0.8	0.704225352112676	83.24022346368710	62.500000000000000	0.7465800273597810	0.6756756756756760	0.5813953488372090	41.86046511627910
86	81.84357541899440	77.19298245614030	0.8065705128205130	0.8148148148148150	0.7333333333333330	84.35754189944130	73.58490566037740	0.7971751412429380	0.8297872340425530	0.6610169491525420	33.89830508474580
87	85.47486033519550	80.88235294117650	0.8389764797105200	0.873015873015873	0.7534246575342470	88.8268156424581	77.77777777777780	0.8275122549019610	0.8974358974358980	0.6862745098039220	31.372549019607800
88	83.79888268156430	78.19548872180450	0.8199008868022950	0.8387096774193550	0.7323943661971830	87.15083798882680	73.56321839080460	0.7958812840702610	0.9142857142857140	0.6153846153846150	38.46153846153850
89	79.88826815642460	72.93233082706770	0.7784204671857620	0.8738738738738740	0.6258064516129030	85.47486033519550	70.45454545454550	0.7783918837068440	0.8611111111111110	0.5961538461538460	40.38461538461540
90	82.9608938547486	77.97833935018050	0.8143269230769230	0.8503937007874020	0.72	84.35754189944130	73.07692307692310	0.7902889691580990	0.8837209302325580	0.6229508196721310	37.704918032786900
91	83.79888268156430	77.51937984496130	0.8174521914324410	0.8264462809917360	0.7299270072992700	88.8268156424581	77.27272727272730	0.8351869761444230	0.8292682926829270	0.723404255319149	27.659574468085100
92	82.12290502793300	77.62237762237760	0.8097215667767810	0.9024390243902440	0.6809815950920250	82.68156424581010	66.66666666666670	0.7502118644067800	0.9117647058823530	0.5254237288135590	47.4576271186441
93	84.91620111731840	80.1470588235294	0.8381093544137020	0.8134328358208960	0.7898550724637680	86.03351955307260	71.91011235955060	0.7989821882951650	0.7804878048780490	0.6666666666666670	33.33333333333330
94	79.88826815642460	75.0	0.7869238604532720	0.8059701492537310	0.7012987012987010	82.12290502793300	64.44444444444440	0.7473643410852710	0.725	0.58	42.0
95	81.56424581005590	75.91240875912410	0.798446651387828	0.8666666666666670	0.6753246753246750	83.79888268156430	71.2871287128713	0.7800847457627120	0.8571428571428570	0.6101694915254240	38.983050847457600
96	77.93296089385480	70.41198501872660	0.7586261777438250	0.831858407079646	0.6103896103896100	84.35754189944130	68.88888888888890	0.767037037037037	0.8611111111111110	0.5740740740740740	42.592592592592600
97	80.72625698324020	76.28865979381440	0.7975035327366930	0.8809523809523810	0.6727272727272730	84.35754189944130	74.54545454545460	0.7995347564313080	0.8723404255319150	0.6507936507936510	34.92063492063490
98	83.24022346368710	78.41726618705040	0.8182432432432430	0.8384615384615390	0.7364864864864870	84.91620111731840	72.72727272727270	0.7999401018268940	0.782608695652174	0.6792452830188680	32.075471698113200
99	82.68156424581010	75.0	0.8001399253731340	0.8157894736842110	0.6940298507462690	84.91620111731840	70.32967032967030	0.7762962962962960	0.8648648648648650	0.5925925925925930	40.74074074074070
**Mean**	**81.6648044692738**	**76.51605544475390**	**0.8022488884176600**	**0.848039421350223**	**0.6986951236443420**	**84.74860335195530**	**71.4083950799508**	**0.7847174623051110**	**0.854361849100874**	**0.61701073582221**	**38.298926417779**
**Standard Deviation**	2.32978410923985	3.09100686544122	0.0240583158973208	0.0338390028289972	0.0441545556375893	3.03455595663564	5.74398274719143	0.0375719364455850	0.0607427108396408	0.070274728374605	7.0274728374605
**T Test Significance (Female to Male)**						**0.000000292768275**	**0.000000000000012€**	**0.129706683642131**	**0.877003027073397**	**0.0000000000000000(**	**0.0000000000000000(**

	B_F_TNR	B_F_FPR	B_F_TPR	B_M_Accuracy	B_M_FScore	B_M_ROC_AUC	B_M_Precision	B_M_Recall	B_M_FNR	B_M_TNR	B_M_FPR	B_M_TPR
0	96.72131147540980	3.278688524590160	56.14035087719300	82.12290502793300	82.79569892473120	0.8249559082892420	0.875	0.7857142857142860	21.428571428571400	86.41975308641980	13.580246913580200	78.57142857142860
1	92.80000000000000	7.200000000000000	61.111111111111100	77.6536312849162	76.47058823529410	0.7762796504369540	0.8024691358024690	0.7303370786516850	26.96629213483150	82.22222222222220	17.77777777777780	73.03370786516850
2	96.03174603174600	3.968253968253970	66.0377358490566	78.2122905027933	77.71428571428570	0.7820224719101120	0.7906976744186050	0.7640449438202250	23.595505617977500	80.0	20.0	76.40449438202250
3	94.35483870967740	5.64516129032258	60.0	80.44692737430170	81.28342245989310	0.8141074611662850	0.8941176470588240	0.7450980392156860	25.49019607843140	88.31168831168830	11.688311688311700	74.50980392156860
4	96.52173913043480	3.4782608695652200	50.0	78.2122905027933	77.71428571428570	0.7852315394242800	0.8395061728395060	0.723404255319149	27.659574468085100	84.70588235294120	15.294117647058800	72.3404255319149
5	94.35483870967740	5.64516129032258	65.45454545454550	82.12290502793300	82.41758241758240	0.8211788211788210	0.8241758241758240	0.8241758241758240	17.582417582417600	81.81818181818180	18.181818181818200	82.41758241758240
6	94.44444444444440	5.555555555555560	67.9245283018868	74.86033519553070	77.15736040609140	0.7537966537966540	0.8260869565217390	0.7238095238095240	27.61904761904760	78.37837837837840	21.62162162162160	72.38095238095240
7	93.75	6.25	52.23880597014930	79.3296089385475	81.21827411167510	0.7962442514052120	0.851063829787234	0.7766990291262140	22.330097087378600	81.57894736842110	18.421052631578900	77.66990291262140
8	95.90163934426230	4.098360655737710	71.9298245614035	79.88826815642460	80.64516129032260	0.8053797468354430	0.872093023255814	0.75	25.0	86.07594936708860	13.924050632911400	75.0
9	99.16666666666670	0.8333333333333330	67.79661016949150	76.53631284916200	78.35051546391750	0.7814293166405840	0.8837209302325580	0.7037037037037040	29.629629629629600	85.91549295774650	14.084507042253500	70.37037037037040
10	96.03174603174600	3.968253968253970	52.83018867924530	79.88826815642460	77.5	0.7995006242197250	0.8857142857142860	0.6888888888888890	31.11111111111110	91.01123595505620	8.98876404494382	68.88888888888890
11	96.8	3.2	72.22222222222220	78.2122905027933	80.20304568527920	0.786474358974359	0.8494623655913980	0.7596153846153850	24.03846153846150	81.33333333333330	18.666666666666700	75.96153846153850
12	93.5251798561151	6.47482014388490	60.0	83.24022346368710	83.51648351648350	0.8325837081459270	0.8444444444444440	0.8260869565217390	17.391304347826100	83.9080459770115	16.091954022988500	82.6086956521739
13	95.7983193277311	4.201680672268910	71.66666666666670	79.88826815642460	80.21978021978020	0.7994498624656160	0.8202247191011240	0.7849462365591400	21.50537634408600	81.3953488372093	18.6046511627907	78.49462365591400
14	96.72131147540980	3.278688524590160	43.859649122807000	78.77094972067040	78.88888888888890	0.789424280350438	0.8255813953488370	0.7553191489361700	24.46808510638300	82.35294117647060	17.647058823529400	75.53191489361700
15	96.7741935483871	3.225806451612900	65.45454545454550	79.88826815642460	80.85106382978720	0.8120512820512820	0.9047619047619050	0.7307692307692310	26.923076923076900	89.33333333333330	10.666666666666700	73.07692307692310
16	97.45762711864410	2.5423728813559300	67.21311475409840	78.77094972067040	80.0	0.7869005994005990	0.7676767676767680	0.8351648351648350	16.483516483516500	73.86363636363640	26.136363636363600	83.51648351648350
17	95.0	5.0	61.016949152542400	73.74301675977650	74.5945945945946	0.7422348484848490	0.8023255813953490	0.696969696969697	30.303030303030300	78.75	21.25	69.6969696969697
18	95.0413223140496	4.958677685950410	58.620689655172400	77.6536312849162	77.77777777777780	0.7779444861215300	0.7446808510638300	0.813953488372093	18.6046511627907	74.19354838709680	25.806451612903200	81.3953488372093
19	95.16129032258070	4.838709677419360	67.27272727272730	77.6536312849162	75.60975609756100	0.7744436109027260	0.7948717948717950	0.7209302325581400	27.906976744186000	82.79569892473120	17.20430107526880	72.09302325581400
20	93.27731092436970	6.722689075630250	53.333333333333300	77.09497206703910	78.30687830687830	0.7709588353413660	0.7956989247311830	0.7708333333333330	22.916666666666700	77.10843373493980	22.89156626506020	77.08333333333330
21	94.9579831932773	5.042016806722690	58.333333333333300	77.6536312849162	80.39215686274510	0.7832896461336830	0.8631578947368420	0.7522935779816510	24.770642201834900	81.42857142857140	18.571428571428600	75.22935779816510
22	98.31932773109240	1.680672268907560	53.333333333333300	77.6536312849162	76.47058823529410	0.7762796504369540	0.8024691358024690	0.7303370786516850	26.96629213483150	82.22222222222220	17.77777777777780	73.03370786516850
23	95.0413223140496	4.958677685950410	70.6896551724138	83.24022346368710	83.69565217391310	0.8388888888888890	0.9058823529411770	0.7777777777777780	22.22222222222220	90.0	10.0	77.77777777777780
24	90.74074074074080	9.25925925925926	56.33802816901410	80.44692737430170	81.48148148148150	0.8090506329113920	0.8651685393258430	0.77	23.0	84.81012658227850	15.18987341772150	77.0
25	95.96774193548390	4.032258064516130	61.81818181818180	81.56424581005590	81.14285714285710	0.8156546726636680	0.8068181818181820	0.8160919540229890	18.39080459770120	81.52173913043480	18.478260869565200	81.60919540229890
26	96.875	3.125	78.43137254901960	82.12290502793300	82.6086956521739	0.8246794065878800	0.8735632183908050	0.7835051546391750	21.649484536082500	86.58536585365850	13.414634146341500	78.35051546391750
27	94.53125	5.46875	62.745098039215700	75.97765363128490	77.00534759358290	0.7624086671705720	0.8089887640449440	0.7346938775510200	26.53061224489800	79.01234567901230	20.98765432098770	73.46938775510210
28	94.07407407407410	5.9259259259259300	70.45454545454550	77.6536312849162	80.3921568627451	0.798110661268556	0.9111111111111110	0.7192982456140350	28.07017543859650	87.6923076923077	12.307692307692300	71.9298245614035
29	96.7741935483871	3.225806451612900	72.72727272727270	75.41899441340780	75.55555555555560	0.7558197747183980	0.7906976744186050	0.723404255319149	27.659574468085100	78.82352941176470	21.176470588235300	72.3404255319149
30	93.60000000000000	6.4	57.407407407407400	84.35754189944130	83.90804597701150	0.8450149925037480	0.8902439024390240	0.7934782608695650	20.652173913043500	89.65517241379310	10.344827586206900	79.34782608695650
31	96.06299212598430	3.937007874015750	51.92307692307690	83.79888268156430	84.81675392670160	0.8432977913175930	0.9	0.801980198019802	19.801980198019800	88.46153846153850	11.538461538461500	80.19801980198020
32	92.0	8.0	53.70370370370370	74.86033519553070	76.19047619047620	0.7500629881582260	0.7912087912087910	0.7346938775510200	26.53061224489800	76.5432098765432	23.456790123456800	73.46938775510210
33	92.0	8.0	48.148148148148100	76.53631284916200	78.12500000000000	0.7760617760617760	0.8620689655172410	0.7142857142857140	28.57142857142860	83.78378378378380	16.216216216216200	71.42857142857140
34	93.4959349593496	6.504065040650410	64.28571428571430	77.6536312849162	77.77777777777780	0.7765293383270910	0.7777777777777780	0.7777777777777780	22.22222222222220	77.52808988764050	22.47191011235960	77.77777777777780
35	93.33333333333330	6.666666666666670	55.932203389830500	76.53631284916200	77.89473684210530	0.7674873737373740	0.8131868131868130	0.7474747474747480	25.252525252525300	78.75	21.25	74.74747474747480
36	95.2755905511811	4.724409448818900	50.0	71.50837988826820	70.52023121387280	0.7248047367094990	0.8133333333333330	0.6224489795918370	37.755102040816300	82.71604938271610	17.28395061728400	62.244897959183700
37	93.4959349593496	6.504065040650410	67.85714285714290	74.30167597765360	74.72527472527470	0.7602564102564100	0.8717948717948720	0.6538461538461540	34.61538461538460	86.66666666666670	13.333333333333300	65.38461538461540
38	94.9579831932773	5.042016806722690	66.66666666666670	72.62569832402240	73.224043715847	0.7397164026571280	0.8375	0.6504854368932040	34.95145631067960	82.89473684210530	17.105263157894700	65.0485436893204
39	91.93548387096770	8.064516129032260	61.81818181818180	80.44692737430170	83.09178743961350	0.8046469366562820	0.86	0.8037383177570090	19.626168224299100	80.55555555555560	19.444444444444400	80.37383177570090
40	94.01709401709400	5.982905982905980	69.35483870967740	79.3296089385475	79.78142076502730	0.7932908545727140	0.8021978021978020	0.7934782608695650	20.652173913043500	79.3103448275862	20.689655172413800	79.34782608695650
41	98.38709677419360	1.6129032258064500	50.90909090909090	74.30167597765360	72.94117647058830	0.7489348370927320	0.8266666666666670	0.6526315789473690	34.73684210526320	84.52380952380950	15.476190476190500	65.26315789473690
42	95.86776859504130	4.132231404958680	53.44827586206900	77.6536312849162	78.26086956521740	0.7770963704630790	0.8	0.7659574468085110	23.404255319148900	78.82352941176470	21.176470588235300	76.59574468085110
43	96.92307692307690	3.076923076923080	59.183673469387800	80.44692737430170	79.53216374269010	0.8042446941323350	0.8292682926829270	0.7640449438202250	23.595505617977500	84.44444444444440	15.555555555555600	76.40449438202250
44	97.54098360655740	2.459016393442620	59.64912280701750	84.91620111731840	85.56149732620320	0.8556962025316460	0.9195402298850580	0.8	20.0	91.13924050632910	8.860759493670890	80.0
45	95.76271186440680	4.23728813559322	49.18032786885250	75.97765363128490	76.24309392265190	0.7656210632401110	0.8313253012048190	0.7040816326530610	29.591836734693900	82.71604938271610	17.28395061728400	70.40816326530610
46	98.38709677419360	1.6129032258064500	72.72727272727270	76.53631284916200	77.41935483870970	0.766566265060241	0.8	0.75	25.0	78.3132530120482	21.686746987951800	75.0
47	97.5	2.5	66.10169491525420	79.88826815642460	80.64516129032260	0.8114460909555440	0.9036144578313250	0.7281553398058250	27.184466019417500	89.47368421052630	10.526315789473700	72.81553398058250
48	96.0	4.0	53.70370370370370	73.18435754189940	76.23762376237620	0.7367631716223260	0.8191489361702130	0.7129629629629630	28.703703703703700	76.05633802816900	23.943661971831000	71.29629629629630
49	93.96551724137930	6.0344827586206900	61.904761904761900	78.2122905027933	78.45303867403320	0.7854543172690760	0.8352941176470590	0.7395833333333330	26.041666666666700	83.13253012048190	16.867469879518100	73.95833333333330
50	92.5233644859813	7.476635514018690	52.77777777777780	77.6536312849162	79.79797979797980	0.773989898989899	0.797979797979798	0.797979797979798	20.2020202020202	75.0	25.0	79.7979797979798
51	97.32142857142860	2.6785714285714300	62.68656716417910	75.41899441340780	73.49397590361450	0.7556193806193810	0.8133333333333330	0.6703296703296700	32.967032967033000	84.0909090909091	15.909090909090900	67.03296703296700
52	94.16666666666670	5.833333333333330	50.847457627118600	79.3296089385475	79.55801104972380	0.8011363636363640	0.8780487804878050	0.7272727272727270	27.27272727272730	87.5	12.5	72.72727272727270
53	96.63865546218490	3.361344537815130	45.0	73.18435754189940	73.62637362637360	0.741939578573242	0.8271604938271610	0.6633663366336630	33.663366336633700	82.05128205128200	17.94871794871800	66.33663366336630
54	97.61904761904760	2.380952380952380	69.81132075471700	75.41899441340780	74.4186046511628	0.7628237364847880	0.8533333333333330	0.6597938144329900	34.02061855670100	86.58536585365850	13.414634146341500	65.97938144329900
55	95.23809523809520	4.761904761904760	60.37735849056600	77.09497206703910	78.3068783068783	0.7783295136236310	0.8505747126436780	0.7254901960784310	27.450980392156900	83.11688311688310	16.883116883116900	72.54901960784310
56	93.60000000000000	6.4	74.07407407407410	78.2122905027933	77.96610169491530	0.7857769423558900	0.8414634146341460	0.7263157894736840	27.368421052631600	84.52380952380950	15.476190476190500	72.63157894736840
57	97.5609756097561	2.4390243902439000	60.71428571428570	81.56424581005590	83.07692307692310	0.8153409090909090	0.84375	0.8181818181818180	18.181818181818200	81.25	18.75	81.81818181818180
58	97.54098360655740	2.459016393442620	56.14035087719300	79.88826815642460	81.44329896907220	0.8086229086229090	0.8876404494382020	0.7523809523809520	24.761904761904800	86.48648648648650	13.513513513513500	75.23809523809520
59	98.3739837398374	1.6260162601626000	67.85714285714290	78.77094972067040	81.37254901960790	0.797562582345191	0.8829787234042550	0.7545454545454550	24.545454545454500	84.05797101449280	15.942028985507200	75.45454545454550
60	93.10344827586210	6.896551724137930	60.317460317460300	76.53631284916200	77.65957446808510	0.7675107079868990	0.8111111111111110	0.7448979591836740	25.510204081632700	79.01234567901230	20.98765432098770	74.48979591836740
61	94.73684210526320	5.263157894736840	58.69565217391310	80.44692737430170	83.87096774193550	0.8200194498471800	0.9191919191919190	0.7711864406779660	22.88135593220340	86.88524590163930	13.114754098360700	77.11864406779660
62	97.45762711864410	2.5423728813559300	70.49180327868850	82.68156424581010	82.68156424581010	0.8274612693653170	0.8505747126436780	0.8043478260869570	19.565217391304300	85.0574712643678	14.942528735632200	80.43478260869570
63	94.81481481481480	5.185185185185190	68.18181818181820	75.41899441340780	76.84210526315790	0.7604405398523050	0.8295454545454550	0.7156862745098040	28.431372549019600	80.51948051948050	19.480519480519500	71.56862745098040
64	94.16666666666670	5.833333333333330	59.32203389830510	80.44692737430170	79.28994082840240	0.8066591704147930	0.8701298701298700	0.7282608695652170	27.173913043478300	88.50574712643680	11.494252873563200	72.82608695652170
65	93.75	6.25	62.745098039215700	82.12290502793300	82.97872340425530	0.8227935629871760	0.8571428571428570	0.8041237113402060	19.587628865979400	84.14634146341460	15.853658536585400	80.41237113402060
66	94.61538461538460	5.384615384615390	71.42857142857140	74.30167597765360	75.26881720430110	0.7472853535353540	0.8045977011494250	0.7070707070707070	29.292929292929300	78.75	21.25	70.70707070707070
67	97.5609756097561	2.4390243902439000	58.92857142857140	70.94972067039110	72.63157894736840	0.7165304036791010	0.7931034482758620	0.6699029126213590	33.00970873786410	76.31578947368420	23.684210526315800	66.99029126213590
68	93.16239316239320	6.837606837606840	64.51612903225810	83.24022346368710	83.51648351648350	0.8330207551887970	0.8539325842696630	0.8172043010752690	18.27956989247310	84.88372093023260	15.11627906976740	81.72043010752690
69	93.7984496124031	6.2015503875969	60.0	79.88826815642460	80.85106382978720	0.8146074646074650	0.9156626506024100	0.7238095238095240	27.61904761904760	90.54054054054050	9.45945945945946	72.38095238095240
70	90.98360655737700	9.01639344262295	54.385964912280700	77.6536312849162	77.27272727272730	0.7893670886075950	0.8947368421052630	0.68	32.0	89.87341772151900	10.126582278481000	68.0
71	93.22033898305080	6.779661016949150	65.57377049180330	77.09497206703910	78.07486631016040	0.7749368686868690	0.8295454545454550	0.7373737373737370	26.262626262626300	81.25	18.75	73.73737373737370
72	93.38842975206610	6.6115702479338800	63.793103448275900	76.53631284916200	74.6987951807229	0.7643606393606390	0.7948717948717950	0.7045454545454550	29.545454545454500	82.41758241758240	17.582417582417600	70.45454545454550
73	95.34883720930230	4.651162790697680	60.0	79.88826815642460	80.64516129032260	0.8024061476442430	0.8522727272727270	0.7653061224489800	23.46938775510200	83.9506172839506	16.049382716049400	76.53061224489800
74	96.63865546218490	3.361344537815130	70.0	82.12290502793300	82.02247191011230	0.8246867167919800	0.8795180722891570	0.7684210526315790	23.157894736842100	88.09523809523810	11.904761904761900	76.84210526315790
75	95.0413223140496	4.958677685950410	74.13793103448280	80.44692737430170	81.08108108108110	0.8041333666333670	0.7978723404255320	0.8241758241758240	17.582417582417600	78.4090909090909	21.59090909090910	82.41758241758240
76	96.875	3.125	62.745098039215700	81.00558659217880	81.5217391304348	0.8200050774308200	0.9036144578313250	0.7425742574257430	25.742574257425700	89.74358974358980	10.256410256410300	74.25742574257430
77	90.4	9.6	59.25925925925930	74.86033519553070	75.40983606557380	0.7563924050632910	0.8313253012048190	0.69	31.0	82.27848101265820	17.72151898734180	69.0
78	95.3125	4.6875	64.70588235294120	81.00558659217880	82.82828282828280	0.8206775700934580	0.9010989010989010	0.7663551401869160	23.364485981308400	87.5	12.5	76.63551401869160
79	98.33333333333330	1.6666666666666700	66.10169491525420	81.00558659217880	79.51807228915660	0.811500999000999	0.88	0.7252747252747250	27.472527472527500	89.77272727272730	10.227272727272700	72.52747252747250
80	95.48872180451130	4.511278195488720	60.86956521739130	78.2122905027933	81.69014084507040	0.7795125164690380	0.8446601941747570	0.7909090909090910	20.909090909090900	76.81159420289860	23.18840579710150	79.0909090909091
81	96.69421487603310	3.3057851239669400	62.06896551724140	75.97765363128490	77.00534759358290	0.7808930425752860	0.9	0.6728971962616820	32.71028037383180	88.88888888888890	11.11111111111110	67.28971962616820
82	94.61538461538460	5.384615384615390	63.26530612244900	77.6536312849162	78.72340425531920	0.7848230201171380	0.8604651162790700	0.7254901960784310	27.450980392156900	84.4155844155844	15.584415584415600	72.54901960784310
83	100.0	0.0	58.333333333333300	80.44692737430170	80.66298342541440	0.8124368686868690	0.8902439024390240	0.7373737373737370	26.262626262626300	88.75	11.25	73.73737373737370
84	99.1869918699187	0.8130081300813010	62.5	83.79888268156430	83.97790055248620	0.839549436795995	0.8735632183908050	0.8085106382978720	19.148936170212800	87.05882352941180	12.941176470588200	80.85106382978720
85	91.17647058823530	8.823529411764710	58.139534883720900	79.3296089385475	80.21390374331550	0.7975378787878790	0.8522727272727270	0.7575757575757580	24.242424242424200	83.75	16.25	75.75757575757580
86	93.33333333333330	6.666666666666670	66.10169491525420	79.3296089385475	79.3296089385475	0.793518981018981	0.8068181818181820	0.7802197802197800	21.978021978022000	80.68181818181820	19.318181818181800	78.02197802197800
87	96.875	3.125	68.62745098039220	82.12290502793300	82.41758241758240	0.8233082706766920	0.8620689655172410	0.7894736842105260	21.052631578947400	85.71428571428570	14.285714285714300	78.94736842105260
88	97.63779527559060	2.3622047244094500	61.53846153846150	80.44692737430170	80.44692737430170	0.8044943820224720	0.8089887640449440	0.8	20.0	80.89887640449440	19.101123595505600	80.0
89	96.06299212598430	3.937007874015750	59.61538461538460	74.30167597765360	74.15730337078650	0.7611778231987740	0.88	0.6407766990291260	35.92233009708740	88.1578947368421	11.842105263157900	64.07766990291260
90	95.76271186440680	4.23728813559322	62.295081967213100	81.56424581005590	80.92485549132950	0.8154806491885150	0.8333333333333330	0.7865168539325840	21.34831460674160	84.44444444444440	15.555555555555600	78.65168539325840
91	94.6969696969697	5.303030303030300	72.3404255319149	78.77094972067040	77.6470588235294	0.7880149812734080	0.825	0.7333333333333330	26.666666666666700	84.26966292134830	15.730337078651700	73.33333333333330
92	97.5	2.5	52.54237288135590	81.56424581005590	82.90155440414510	0.8246153846153850	0.898876404494382	0.7692307692307690	23.076923076923100	88.0	12.0	76.92307692307690
93	93.12977099236640	6.870229007633590	66.66666666666670	83.79888268156430	84.15300546448090	0.8378901373283400	0.8279569892473120	0.8555555555555560	14.444444444444400	82.02247191011240	17.97752808988760	85.55555555555560
94	91.47286821705430	8.527131782945740	58.00000000000000	77.6536312849162	79.79797979797980	0.7798076923076920	0.8404255319148940	0.7596153846153850	24.03846153846150	80.0	20.0	75.96153846153850
95	95.0	5.0	61.016949152542400	79.3296089385475	78.61271676300580	0.7983709273182960	0.8717948717948720	0.7157894736842110	28.421052631578900	88.09523809523810	11.904761904761900	71.57894736842110
96	96.0	4.0	57.407407407407400	71.50837988826820	71.1864406779661	0.7263924050632910	0.8181818181818180	0.63	37.0	82.27848101265820	17.72151898734180	63.0
97	94.82758620689660	5.172413793103450	65.07936507936510	77.09497206703910	77.34806629834250	0.7846956964604020	0.8860759493670890	0.6862745098039220	31.372549019607800	88.31168831168830	11.688311688311700	68.62745098039220
98	92.06349206349210	7.936507936507940	67.9245283018868	81.56424581005590	81.56424581005590	0.818734335839599	0.8690476190476190	0.7684210526315790	23.157894736842100	86.90476190476190	13.095238095238100	76.84210526315790
99	96.0	4.0	59.25925925925930	80.44692737430170	77.70700636942680	0.8004419191919190	0.7922077922077920	0.7625	23.75	83.83838383838380	16.16161616161620	76.25
**Mean**	**95.24241887880130**	**4.75758112119873**	**61.701073582221**	**78.5810055865922**	**79.09522044703040**	**0.7906740065170280**	**0.84494493980242**	**0.745856081111063**	**25.4143918888937**	**83.5491931922992**	**16.4508068077008**	**74.5856081111063**
**Standard Deviation**	2.03122731632055	2.03122731632055	7.0274728374605	3.05187652756651	3.20543297948785	0.0293412237985650	0.0391866874508032	0.0493444951908078	4.93444951908078	4.16356144691541	4.16356144691541	4.93444951908078
**T Test Significance (Female to Male)**	**0.0000000000000000(**	**0.0000000000000000(**	**0.0000000000000000(**									

3.1.2 Balanced training data, no feature selection - Logistic Regression Classifier

	B_Accuracy	B_FScore	B_ROC_AUC	B_Precision	B_Recall	B_F_Accuracy	B_F_FScore	B_F_ROC_AUC	B_F_Precision	B_F_Recall	B_F_FNR
0	74.02234636871510	65.68265682656830	0.72011796588554	0.75423728813559	0.58169934640522	78.77094972067040	55.81395348837210	0.68995601173020	0.77419354838709	0.43636363636363	56.36363636363640
1	76.25698324022350	67.9245283018868	0.74004223054035	0.78947368421052	0.59602649006622	77.09497206703910	49.38271604938270	0.65762463343108	0.76923076923076	0.36363636363636	63.63636363636360
2	74.30167597765360	65.67164179104480	0.72304084672180	0.79279279279279	0.56050955414012	78.77094972067040	57.77777777777780	0.70040288969158	0.89655172413793	0.42622950819672	57.37704918032790
3	71.22905027932960	61.99261992619930	0.69487971750165	0.76363636363636	0.52173913043478	75.41899441340780	50.0	0.65659732117412	0.73333333333333	0.37931034482758	62.06896551724140
4	76.81564245810060	67.95366795366800	0.74308154973286	0.81481481481481	0.58278145695364	76.53631284916200	44.73684210526320	0.63841642228739	0.80952380952381	0.30909090909090	69.0909090909091
5	75.13966480446930	68.77192982456140	0.73794996949359	0.72058823529411	0.65771812080536	77.09497206703910	57.73195876288660	0.69562146892655	0.73684210526315	0.47457627118644	52.54237288135590
6	74.30167597765360	65.92592592592590	0.72255699027578	0.76068376068376	0.58169934640522	80.44692737430170	59.77011494252870	0.71583946078431	0.72222222222222	0.50980392156862	49.01960784313730
7	74.02234636871510	65.93406593406590	0.72271736038684	0.78947368421052	0.56603773584905	75.41899441340780	53.19148936170210	0.67476383265856	0.86206896551724	0.38461538461538	61.53846153846150
8	74.30167597765360	68.05555555555560	0.735747568564906	0.83760683760683	0.57309941520467	76.53631284916200	58.82352941176470	0.70511140235910	0.9375	0.42857142857142	57.14285714285710
9	74.30167597765360	64.0625	0.71512226512226	0.75925925925925	0.55405405405405	81.56424581005590	53.52112676056340	0.68396534815299	0.76	0.41304347826087	58.69565217391310
10	76.25698324022350	65.86345381526100	0.73129361592142	0.79611650485436	0.56164383561643	78.2122905027933	49.35064935064940	0.66256830601092	0.95	0.33333333333333	66.66666666666670
11	72.06703910614520	62.12121212121210	0.69895744208891	0.76635514018691	0.52229299363057	74.30167597765360	43.90243902439030	0.63482911919977	0.85714285714285	0.29508196721311	70.49180327868850
12	73.46368715083800	57.77777777777780	0.684265218557254	0.69892473118279	0.49242424242424	76.53631284916200	36.36363636363640	0.60449612403100	0.75	0.24	76.0
13	76.25698324022350	67.18146718146720	0.73760717453434	0.725	0.625899280575540	78.77094972067040	52.50000000000000	0.67478005865102	0.84	0.38181818181818	61.81818181818180
14	70.6703910614525	59.77011494252870	0.68798175239893	0.79591836734693	0.47852760736196	73.18435754189940	44.18604651162790	0.63355227148330	0.82608695652173	0.30158730158730	69.84126984126980
15	74.86033519553070	65.90909090909090	0.72487129987130	0.75	0.58783783783783	77.6536312849162	52.38095238095240	0.67170370370370	0.73333333333333	0.40740740740740	59.25925925925930
16	75.41899441340780	66.412213740458	0.730108455705922	0.78378378378378	0.57615894039735	79.3296089385475	58.42696629213480	0.70347677400968	0.83870967741935	0.44827586206896	55.17241379310340
17	77.09497206703910	70.92198581560280	0.75645569620253	0.80645161290322	0.63291139240506	78.77094972067040	58.69565217391310	0.70381355932203	0.81818181818181	0.45762711864406	54.23728813559320
18	73.74301675977650	64.39393939393940	0.71597426878347	0.794392523364486	0.541401273885350	74.30167597765360	34.28571428571430	0.59699413489736	0.8	0.21818181818181	78.18181818181820
19	74.02234636871510	65.16853932584270	0.71772844272844	0.731092436974790	0.58783783783783	78.77094972067040	48.64864864864870	0.65732889158086	0.81818181818181	0.34615384615384	65.38461538461540
20	77.37430167597770	68.72586872586870	0.74858974358974	0.816513761467890	0.59333333333333	81.00558659217880	56.41025641025640	0.69570370370370	0.91666666666666	0.40740740740740	59.25925925925930
21	72.3463687150838	64.25992779783390	0.71078062248996	0.80180180180180	0.536144578313253	75.97765363128490	49.41176470588230	0.66080783016266	0.913043478260870	0.33870967741935	66.12903225806450
22	76.81564245810060	66.66666666666670	0.736721418116767	0.78301886792452	0.580419580419580	79.3296089385475	49.31506849315070	0.660596546310832	0.75	0.367346938775510	63.26530612244900
23	74.86033519553070	66.4179104477612	0.72914556962025	0.80909090909090	0.56329113924050	78.77094972067040	52.5	0.67530487804878	0.875	0.375	62.5
24	76.53631284916200	66.66666666666670	0.73708289343187	0.83168316831683	0.55629139072847	77.6536312849162	48.71794871794870	0.66101694915254	1.0	0.322033898305085	67.79661016949150
25	75.69832402234640	66.92015209125480	0.73364844149208	0.79279279279279	0.57894736842105	77.6536312849162	48.71794871794870	0.65744773519163	0.86363636363636	0.33928571428571	66.07142857142860
26	75.97765363128490	69.50354609929080	0.74723409605230	0.83050847457627	0.59756097560975	75.97765363128490	50.57471264367820	0.66459884201819	0.88	0.35483870967741	64.51612903225810
27	76.25698324022350	68.40148698884760	0.74408204544736	0.83636363636363	0.578616352201258	78.2122905027933	48.0	0.65466666666666	0.85714285714285	0.33333333333333	66.66666666666670
28	72.3463687150838	65.50522648083620	0.71070719172683	0.746031746031746	0.58385093167701	74.86033519553070	52.63157894736840	0.67037786774628	0.83333333333333	0.38461538461538	61.53846153846150
29	74.02234636871510	64.90566037735850	0.71683541017653	0.71074380165289	0.59722222222222	77.6536312849162	48.71794871794870	0.65659824046920	0.82608695652173	0.34545454545454	65.45454545454550
30	75.97765363128490	68.84057971014490	0.74346575645523	0.811965811965812	0.597484276729560	77.6536312849162	54.54545454545450	0.68183779119931	0.77419354838709	0.42105263157894	57.89473684210530
31	75.13966480446930	65.90038314176250	0.72612432153011	0.74782608695652	0.589041095890411	76.53631284916200	44.73684210526320	0.63541666666666	0.68	0.33333333333333	66.66666666666670
32	73.18435754189940	62.20472440944880	0.705577625933577	0.797979797979798	0.50967741935483	75.97765363128490	50.57471264367820	0.66232492997198	0.81481481481481	0.36666666666666	63.33333333333330
33	75.13966480446930	65.63706563706560	0.72542628688866	0.77272727272727	0.57046979865771	81.00558659217880	58.53658536585370	0.70615563298490	0.92307692307692	0.42857142857142	57.14285714285710
34	77.37430167597770	68.72586872586870	0.74904980340760	0.74789915966386	0.63571428571428	82.12290502793300	51.51515151515150	0.67833156592435	0.68	0.41463414634146	58.53658536585370
35	73.74301675977650	64.39393939393940	0.71648734177215	0.801886792452830	0.53797468354430	72.62569832402240	43.67816091954020	0.63104619565217	0.82608695652173	0.296875	70.3125
36	71.50837988826820	60.76923076923080	0.69535777273872	0.80612244897959	0.48765432098765	76.53631284916200	50.0	0.66129943502824	0.84	0.35593220338983	64.40677966101690
37	79.60893854748600	72.24334600760460	0.77506553079947	0.77235772357723	0.67857142857142	82.12290502793300	62.7906976744186	0.73511627906976	0.75	0.54	46.0
38	74.30167597765360	65.15151515151520	0.71978794072560	0.76785714285714	0.56578947368421	77.09497206703910	50.60240963855420	0.66371872303710	0.80769230769230	0.36842105263157	63.1578947368421
39	74.58100558659220	64.31372549019610	0.72018115366280	0.82	0.52903225806451	78.2122905027933	40.0	0.62111459968602	0.8125	0.265306122448980	73.46938775510210
40	69.83240223463690	58.13953488372090	0.670673076923077	0.69444444444444	0.5	77.09497206703910	49.38271604938270	0.65718518518518	0.74074074074074	0.370370370370370	62.96296296296300
41	74.30167597765360	66.42335766423360	0.72677113220039	0.80530973451327	0.56521739130434	79.3296089385475	55.42168674698800	0.688962962962963	0.79310344827586	0.42592592592592	57.40740740740740
42	71.7877094972067	63.27272727272730	0.70031645569620	0.74358974358974	0.55063291139240	70.39106145251400	43.01075268817210	0.61875843454790	0.71428571428571	0.307692307692308	69.23076923076920
43	73.46368715083800	64.41947565543070	0.71214743589743	0.73504273504273	0.57333333333333	77.09497206703910	57.73195876288660	0.696360153256705	0.82352941176470	0.44444444444444	55.55555555555560
44	72.62569832402240	63.43283582089550	0.70945414503696	0.80952380952381	0.52147239263803	72.62569832402240	46.15384615384620	0.64139179404666	0.84	0.31818181818181	68.18181818181820
45	78.49162011173190	71.16104868913860	0.76380308880308	0.79831932773109	0.64189189189189	80.44692737430170	58.82352941176470	0.70700316364682	0.89285714285714	0.438596491228070	56.14035087719300
46	74.58100558659220	61.92468619246860	0.71004463568928	0.82222222222222	0.49664429530201	75.41899441340780	38.88888888888890	0.61223420185684	0.73684210526315	0.26415094339622	73.58490566037740
47	74.58100558659220	65.66037735849060	0.72253521126760	0.725	0.6	78.2122905027933	51.85185185185190	0.67074780058651	0.80769230769230	0.38181818181818	61.81818181818180
48	72.62569832402240	62.30769230769230	0.70663265306122	0.826530612244898	0.5	76.53631284916200	48.78048780487800	0.65826330532212	0.90909090909090	0.33333333333333	66.66666666666670
49	77.37430167597770	68.48249027237350	0.74745512347066	0.81481481481481	0.59060402684563	84.91620111731840	61.9718309859155	0.73375512995896	0.78571428571428	0.51162790697674	48.83720930232560
50	72.06703910614520	62.40601503759400	0.69877641824249	0.74774774774774	0.535483870967742	78.2122905027933	49.35064935064940	0.65893075980392	0.73076923076923	0.37254901960784	62.74509803921570
51	76.25698324022350	68.86446886446890	0.744112505959002	0.79661016949152	0.60645161290322	80.44692737430170	56.79012345679010	0.69753179890975	0.79310344827586	0.44230769230769	55.76923076923080
52	74.58100558659220	65.66037735849060	0.72916142821222	0.86138613861386	0.53048780487804	77.6536312849162	54.54545454545450	0.68500137854976	0.92307692307692	0.387096774193548	61.29032258064520
53	74.86033519553070	66.16541353383460	0.726890756302521	0.78571428571428	0.57142857142857	79.3296089385475	50.66666666666670	0.66869501466275	0.95	0.34545454545454	65.45454545454550
54	72.90502793296090	62.2568093385214	0.70147072990591	0.74074074074074	0.53691275167785	76.53631284916200	38.23529411764710	0.61318897637795	0.8125	0.25	75.0
55	71.22905027932960	63.08243727598570	0.70064582876132	0.78571428571428	0.52694610778443	73.74301675977650	52.52525252525250	0.66399456521739	0.74285714285714	0.40625	59.375
56	76.25698324022350	67.680608365019	0.73963016100749	0.80909090909090	0.58169934640522	77.09497206703910	49.38271604938270	0.66001709888857	0.86956521739130	0.34482758620689	65.51724137931040
57	76.25698324022350	68.86446886446890	0.74675610985134	0.84684684684684	0.58024691358024	78.2122905027933	53.01204819277110	0.67913165266106	0.95652173913043	0.36666666666666	63.33333333333330
58	72.3463687150838	63.46863468634690	0.70299697114618	0.72881355932203	0.56209150326797	75.97765363128490	51.68539325842700	0.66645658263305	0.79310344827586	0.38333333333333	61.66666666666670
59	72.06703910614520	62.40601503759400	0.69707207207207	0.70338983050847	0.56081081081081	78.2122905027933	57.14285714285710	0.695212311199772	0.78787878787878	0.44827586206896	55.17241379310340
60	78.2122905027933	72.91666666666670	0.77004419191919	0.8203125	0.65625	76.53631284916200	53.33333333333330	0.67645437000275	0.85714285714285	0.387096774193548	61.29032258064520
61	73.74301675977650	60.50420168067230	0.69898008742107	0.77419354838709	0.496551724137931	78.77094972067040	42.42424242424240	0.63056615776081	0.77777777777777	0.29166666666666	70.83333333333330
62	71.7877094972067	62.45353159851300	0.70125845524618	0.79245283018867	0.51533742331288	77.09497206703910	52.87356321839080	0.674859943977591	0.85185185185185	0.38333333333333	61.66666666666670
63	74.86033519553070	65.11627906976740	0.72522736635294	0.83168316831683	0.535031847133758	77.09497206703910	48.1012658227848	0.65437158469945	0.86363636363636	0.33333333333333	66.66666666666670
64	74.86033519553070	65.9090909090909	0.72686595582635	0.80555555555555	0.55769230769230	80.44692737430170	57.83132530120480	0.70222222222222	0.82758620689655	0.44444444444444	55.55555555555560
65	72.62569832402240	61.71875	0.69811502520792	0.73831775700934	0.53020134228187	78.2122905027933	48.0	0.65466666666666	0.85714285714285	0.33333333333333	66.66666666666670
66	74.30167597765360	67.37588652482270	0.73035579582599	0.80508474576271	0.57926829268292	76.53631284916200	55.31914893617020	0.68684210526315	0.896551724137931	0.4	60.0
67	75.97765363128490	68.84057971014490	0.743844696969697	0.81896551724137	0.59375	76.53631284916200	54.34782608695650	0.68353576248313	0.92592592592592	0.38461538461538	61.53846153846150
68	74.02234636871510	64.63878326996200	0.717543302081678	0.78703703703703	0.54838709677419	79.88826815642460	57.14285714285710	0.69823123382226	0.88888888888888	0.42105263157894	57.89473684210530
69	74.30167597765360	67.6056338028169	0.73082725672617	0.8	0.58536585365853	77.09497206703910	58.58585858585860	0.70047554347826	0.82857142857142	0.453125	54.6875
70	73.46368715083800	65.94982078853050	0.71837037994313	0.69172932330827	0.630136986301370	77.09497206703910	51.76470588235290	0.66881127450980	0.64705882352941	0.43137254901960	56.86274509803920
71	74.58100558659220	65.13409961685820	0.722052457346575	0.794392523364486	0.551948051948052	77.09497206703910	50.60240963855420	0.664505557138786	0.84	0.36206896551724	63.79310344827590
72	76.81564245810060	70.25089605734770	0.752899718719383	0.81666666666666	0.61635220125786	80.44692737430170	60.67415730337080	0.71659663865546	0.93103448275862	0.45	55.00000000000000
73	72.90502793296090	62.25680933852140	0.70074469807349	0.71428571428571	0.55172413793103	79.88826815642460	53.84615384615390	0.68223803755299	0.80769230769230	0.40384615384615	59.61538461538460
74	74.30167597765360	67.83216783216780	0.732272841365462	0.80833333333333	0.584337349397590	78.2122905027933	55.17241379310340	0.68927488282327	0.96	0.387096774193548	61.29032258064520
75	76.81564245810060	68.67924528301890	0.74847507980152	0.85849056603773	0.57232704402515	79.88826815642460	60.0	0.71239495798319	0.9	0.45	55.00000000000000
76	71.50837988826820	64.08450704225350	0.70378388554216	0.77118644067796	0.54819277108433	78.2122905027933	58.06451612903230	0.70012503473187	0.84375	0.44262295081967	55.73770491803280
77	74.30167597765360	65.92592592592590	0.72824619249489	0.847619047619048	0.53939393939393	79.3296089385475	56.47058823529410	0.69505649717514	0.92307692307692	0.40677966101694	59.32203389830510
78	75.41899441340780	63.93442622950820	0.71961620469083	0.70909090909090	0.582089552238806	82.12290502793300	57.89473684210530	0.70905524681268	0.73333333333333	0.47826086956521	52.17391304347830
79	75.13966480446930	68.98954703832750	0.74090110441767	0.81818181818181	0.59638554216867	75.97765363128490	55.67010309278350	0.68576248313090	0.84375	0.41538461538461	58.46153846153850
80	72.90502793296090	64.20664206642070	0.71517319277108	0.82857142857142	0.52409638554216	79.3296089385475	50.66666666666670	0.66792592592592	0.90476190476190	0.35185185185185	64.81481481481480
81	77.6536312849162	66.94214876033060	0.74153037383177	0.826530612244898	0.5625	82.68156424581010	55.07246376811590	0.69076402321083	0.86363636363636	0.404255319148936	59.57446808510640
82	74.58100558659220	66.17100371747210	0.72552041951374	0.78070175438596	0.57419354838709	78.2122905027933	54.11764705882350	0.68126258268622	0.82142857142857	0.40350877192982	59.64912280701750
83	73.74301675977650	66.42857142857140	0.72155147827740	0.756097560975610	0.59235668789808	77.09497206703910	55.91397849462370	0.68700564971751	0.76470588235294	0.44067796610169	55.93220338983050
84	75.41899441340780	66.41221374045800	0.73138407754648	0.81308411214953	0.56129032258064	79.88826815642460	53.84615384615390	0.68284457478005	0.913043478260870	0.38181818181818	61.81818181818180
85	76.25698324022350	66.13545816733070	0.73254989199471	0.79807692307692	0.564625850340136	78.77094972067040	52.50000000000000	0.67478005865102	0.84	0.38181818181818	61.81818181818180
86	72.06703910614520	61.53846153846150	0.69648816144922	0.76190476190476	0.51612903225806	75.41899441340780	45.00000000000000	0.63632404181184	0.75	0.32142857142857	67.85714285714290
87	74.86033519553070	66.91176470588240	0.72978547854785	0.784482758620690	0.58333333333333	75.41899441340780	48.83720930232560	0.65296610169491	0.77777777777777	0.35593220338983	64.40677966101690
88	75.97765363128490	66.14173228346460	0.73091900311526	0.76363636363636	0.58333333333333	78.77094972067040	45.71428571428570	0.64419568822553	0.64	0.35555555555555	64.44444444444440
89	75.97765363128490	65.60000000000000	0.733117542523483	0.872340425531915	0.52564102564102	79.3296089385475	50.66666666666670	0.66792592592592	0.90476190476190	0.35185185185185	64.81481481481480
90	74.02234636871510	64.9056603773585	0.71871033257171	0.78899082568807	0.55128205128205	74.30167597765360	45.23809523809520	0.63601694915254	0.76	0.322033898305085	67.79661016949150
91	71.50837988826820	61.36363636363640	0.69019352740283	0.66942148760330	0.56643356643356	73.74301675977650	35.61643835616440	0.58961513994910	0.52	0.27083333333333	72.91666666666670
92	71.7877094972067	62.73062730627310	0.70744680851063	0.84158415841584	0.5	78.2122905027933	58.06451612903230	0.70064791838985	0.87096774193548	0.43548387096774	56.45161290322580
93	78.49162011173190	70.0389105058366	0.75936590203794	0.84905660377358	0.59602649006622	79.3296089385475	55.42168674698800	0.68909698025551	0.85185185185185	0.41071428571428	58.92857142857140
94	77.09497206703910	67.71653543307090	0.74263375549237	0.79629629629629	0.589041095890411	77.09497206703910	49.38271604938270	0.66115819209039	0.90909090909090	0.33898305084745	66.10169491525420
95	75.97765363128490	68.61313868613140	0.74187611068799	0.79661016949152	0.60256410256410	81.00558659217880	59.52380952380950	0.716640502354788	0.71428571428571	0.51020408163265	48.97959183673470
96	77.09497206703910	69.40298507462690	0.75328688085254	0.86915887850467	0.577639751552795	80.44692737430170	53.33333333333330	0.680742737346511	0.90909090909090	0.37735849056603	62.26415094339620
97	73.46368715083800	65.2014652014652	0.71915998112317	0.80909090909090	0.546012269938650	74.86033519553070	52.63157894736840	0.66742486903777	0.75757575757575	0.40322580645161	59.67741935483870
98	72.62569832402240	64.23357664233580	0.71096429133239	0.79279279279279	0.53987730061349	72.06703910614520	47.91666666666670	0.64326897291499	0.76666666666666	0.34848484848484	65.15151515151520
99	71.7877094972067	62.45353159851300	0.69575479271699	0.7	0.56375838926174	76.53631284916200	54.34782608695650	0.678314639056658	0.71428571428571	0.438596491228070	56.14035087719300
**Mean**	**74.53072625698320**	**65.6299276430387**	**0.72397033434854**	**0.78518632801158**	**0.56572600342406**	**77.70949720670390**	**51.6875240431449**	**0.671031127571197**	**0.822183120479179**	**0.381204795334565**	**61.8795204665435**
Standard Deviation	1.95888328101459	2.88443397136394	0.01971871172663	0.04302695302221	0.03854263892973	2.42476017142929	5.86653850186726	0.02863446491295	0.08042858978150	0.06085333368361	6.08533336836159
**T Test Significance (Female to Male)**						**0.000000000536024**	**0.000000000000000**	**0.000000000038384**	**0.000708353867139**	**0.000000000000000**	**0.000000000000000**

	B_F_TNR	B_F_FPR	B_F_TPR	B_M_Accuracy	B_M_FScore	B_M_ROC_AUC	B_M_Precision	B_M_Recall	B_M_FNR	B_M_TNR	B_M_FPR	B_M_TPR
0	94.35483870967740	5.64516129032258	43.63636363636360	69.27374301675980	70.27027027027030	0.695830183925422	0.74712643678160	0.66326530612244	33.6734693877551	72.8395061728395	27.16049382716050	66.3265306122449
1	95.16129032258070	4.838709677419360	36.36363636363640	75.41899441340780	76.08695652173910	0.75614959839357	0.79545454545454	0.72916666666666	27.08333333333330	78.3132530120482	21.68674698795180	72.91666666666670
2	97.45762711864410	2.542372881355930	42.62295081967210	69.83240223463690	69.66292134831460	0.70243473895582	0.75609756097561	0.64583333333333	35.41666666666670	75.90361445783130	24.09638554216870	64.58333333333330
3	93.38842975206610	6.611570247933880	37.93103448275860	67.0391061452514	67.75956284153010	0.68254982115482	0.775	0.60194174757281	39.80582524271850	76.31578947368420	23.68421052631580	60.19417475728160
4	96.7741935483871	3.225806451612900	30.90909090909090	77.09497206703910	77.59562841530060	0.77340612449799	0.81609195402298	0.73958333333333	26.04166666666670	80.72289156626510	19.27710843373490	73.95833333333330
5	91.66666666666670	8.333333333333330	47.45762711864410	73.18435754189940	74.46808510638300	0.73158551810237	0.71428571428571	0.77777777777777	22.22222222222220	68.53932584269660	31.46067415730340	77.77777777777780
6	92.1875	7.8125	50.98039215686270	68.15642458100560	68.85245901639350	0.69194041252864	0.77777777777777	0.61764705882352	38.23529411764710	76.62337662337660	23.37662337662340	61.76470588235290
7	96.49122807017540	3.508771929824560	38.46153846153850	72.62569832402240	72.62569832402240	0.72809762202753	0.76470588235294	0.69148936170212	30.85106382978720	76.47058823529410	23.52941176470590	69.14893617021280
8	98.1651376146789	1.834862385321100	42.85714285714290	72.06703910614520	73.11827956989250	0.72765930439197	0.8	0.67326732673267	32.67326732673270	78.2051282051282	21.79487179487180	67.32673267326730
9	95.48872180451130	4.511278195488720	41.30434782608700	67.0391061452514	68.1081081081081	0.67895339954163	0.75903614457831	0.61764705882352	38.23529411764710	74.02597402597400	25.97402597402600	61.76470588235290
10	99.18032786885250	0.819672131147541	33.33333333333330	74.30167597765360	73.25581395348840	0.74282147315855	0.75903614457831	0.70786516853932	29.21348314606740	77.77777777777780	22.22222222222220	70.78651685393260
11	97.45762711864410	2.542372881355930	29.50819672131150	69.83240223463690	70.32967032967030	0.70080321285140	0.74418604651162	0.66666666666666	33.33333333333330	73.49397590361450	26.50602409638550	66.66666666666670
12	96.89922480620150	3.10077519379845	24.0	70.39106145251400	66.66666666666670	0.69945939150113	0.68831168831168	0.64634146341463	35.36585365853660	75.25773195876290	24.74226804123710	64.63414634146340
13	96.7741935483871	3.225806451612900	38.18181818181820	73.74301675977650	73.74301675977650	0.74022556390977	0.69473684210526	0.78571428571428	21.42857142857140	69.47368421052630	30.52631578947370	78.57142857142860
14	96.55172413793100	3.448275862068970	30.15873015873020	68.15642458100560	67.42857142857140	0.69373417721519	0.78666666666666	0.59	41.0	79.74683544303800	20.25316455696200	59.0
15	93.60000000000000	6.4	40.74074074074070	72.06703910614520	72.22222222222220	0.72221526908635	0.75581395348837	0.69148936170212	30.85106382978720	75.29411764705880	24.70588235294120	69.14893617021280
16	95.86776859504130	4.132231404958680	44.82758620689660	71.50837988826820	70.52023121387280	0.71749187296824	0.7625	0.65591397849462	34.40860215053760	77.90697674418610	22.09302325581400	65.59139784946240
17	95.0	5.0	45.76271186440680	75.41899441340780	76.8421052631579	0.75618686868686	0.80219780219780	0.73737373737373	26.26262626262630	77.5	22.5	73.73737373737370
18	97.58064516129030	2.419354838709680	21.81818181818180	73.18435754189940	75.25773195876290	0.73446651387827	0.79347826086956	0.71568627450980	28.43137254901960	75.32467532467530	24.67532467532470	71.56862745098040
19	96.8503937007874	3.149606299212600	34.61538461538460	69.27374301675980	71.50259067357510	0.69070030120481	0.711340206185567	0.71875	28.125	66.26506024096390	33.73493975903610	71.875
20	98.4	1.6	40.74074074074070	73.74301675977650	74.03314917127070	0.74052459839357	0.788235294117647	0.69791666666666	30.20833333333330	78.3132530120482	21.68674698795180	69.79166666666670
21	98.29059829059830	1.709401709401710	33.87096774193550	68.71508379888270	70.83333333333330	0.69358974358974	0.77272727272727	0.65384615384615	34.61538461538460	73.33333333333330	26.66666666666670	65.38461538461540
22	95.38461538461540	4.615384615384620	36.73469387755100	74.30167597765360	73.86363636363640	0.745744680851064	0.79268292682926	0.69148936170212	30.85106382978720	80.0	20.0	69.14893617021280
23	97.5609756097561	2.439024390243900	37.5	70.94972067039110	72.34042553191490	0.71645021645021	0.79069767441860	0.66666666666666	33.33333333333330	76.62337662337660	23.37662337662340	66.66666666666670
24	100.0	0.0	32.20338983050850	75.41899441340780	74.71264367816090	0.75555972013993	0.79268292682926	0.70652173913043	29.34782608695650	80.45977011494250	19.54022988505750	70.65217391304350
25	97.5609756097561	2.439024390243900	33.92857142857140	73.74301675977650	74.5945945945946	0.73889307228915	0.77528089887640	0.71875	28.125	75.90361445783130	24.09638554216870	71.875
26	97.43589743589740	2.564102564102560	35.48387096774190	75.97765363128490	77.94871794871800	0.76215940921823	0.81720430107526	0.74509803921568	25.49019607843140	77.92207792207790	22.07792207792210	74.50980392156860
27	97.6	2.4	33.33333333333330	74.30167597765360	76.28865979381450	0.751029601029601	0.83146067415730	0.70476190476190	29.52380952380950	79.72972972972970	20.27027027027030	70.47619047619050
28	95.6140350877193	4.385964912280700	38.46153846153850	69.83240223463690	71.875	0.69672439759036	0.71875	0.71875	28.125	67.46987951807230	32.53012048192770	71.875
29	96.7741935483871	3.225806451612900	34.54545454545460	70.39106145251400	71.6577540106952	0.70418227215980	0.68367346938775	0.75280898876404	24.71910112359550	65.55555555555560	34.44444444444440	75.28089887640450
30	94.26229508196720	5.737704918032790	42.10526315789470	74.30167597765360	75.53191489361700	0.75063661828367	0.82558139534883	0.696078431372549	30.39215686274510	80.51948051948050	19.48051948051950	69.6078431372549
31	93.75	6.25	33.33333333333330	73.74301675977650	74.5945945945946	0.73815789473684	0.76666666666666	0.72631578947368	27.36842105263160	75.0	25.0	72.63157894736840
32	95.7983193277311	4.201680672268910	36.66666666666670	70.39106145251400	68.26347305389220	0.71071428571428	0.79166666666666	0.6	40.0	82.14285714285710	17.85714285714290	60.0
33	98.3739837398374	1.626016260162600	42.85714285714290	69.27374301675980	68.92655367231640	0.69423605901475	0.72619047619047	0.65591397849462	34.40860215053760	73.25581395348840	26.74418604651160	65.59139784946240
34	94.20289855072460	5.797101449275360	41.46341463414630	72.62569832402240	74.61139896373060	0.72613636363636	0.76595744680851	0.72727272727272	27.27272727272730	72.5	27.50000000000000	72.72727272727270
35	96.52173913043480	3.478260869565220	29.6875	74.86033519553070	74.57627118644070	0.75106382978723	0.79518072289156	0.70212765957446	29.78723404255320	80.0	20.0	70.2127659574468
36	96.66666666666670	3.333333333333330	35.59322033898310	66.4804469273743	65.90909090909090	0.68286918753193	0.79452054794520	0.56310679611650	43.68932038834950	80.26315789473690	19.73684210526320	56.31067961165050
37	93.02325581395350	6.976744186046510	54.0	77.09497206703910	76.8361581920904	0.77103620474407	0.78160919540229	0.75555555555555	24.44444444444440	78.65168539325840	21.34831460674160	75.55555555555560
38	95.90163934426230	4.098360655737710	36.84210526315790	71.50837988826820	71.82320441988950	0.71710526315789	0.75581395348837	0.68421052631579	31.57894736842110	75.0	25.0	68.42105263157900
39	97.6923076923077	2.307692307692310	26.53061224489800	70.94972067039110	72.63157894736840	0.72273197208581	0.82142857142857	0.65094339622641	34.90566037735850	79.45205479452060	20.54794520547950	65.09433962264150
40	94.40000000000000	5.600000000000000	37.03703703703700	62.56983240223460	62.14689265536720	0.62983182730923	0.67901234567901	0.57291666666666	42.70833333333330	68.67469879518070	31.32530120481930	57.29166666666670
41	95.20000000000000	4.8	42.59259259259260	69.27374301675980	71.2041884816754	0.70664589823468	0.80952380952381	0.63551401869158	36.44859813084110	77.77777777777780	22.22222222222220	63.55140186915890
42	92.98245614035090	7.017543859649120	30.76923076923080	73.18435754189940	73.6263736263736	0.73230807701925	0.75280898876404	0.72043010752688	27.95698924731180	74.4186046511628	25.58139534883720	72.04301075268820
43	94.82758620689660	5.172413793103450	44.44444444444440	69.83240223463690	68.23529411764710	0.69746376811594	0.69879518072289	0.66666666666666	33.33333333333330	72.82608695652170	27.17391304347830	66.66666666666670
44	96.46017699115040	3.539823008849560	31.81818181818180	72.62569832402240	72.31638418079100	0.73233593160673	0.8	0.65979381443299	34.02061855670100	80.48780487804880	19.51219512195120	65.97938144329900
45	97.54098360655740	2.459016393442620	43.85964912280700	76.53631284916200	76.92307692307690	0.76529720279720	0.76923076923076	0.76923076923076	23.07692307692310	76.13636363636360	23.86363636363640	76.92307692307690
46	96.03174603174600	3.968253968253970	26.41509433962260	73.74301675977650	71.8562874251497	0.74623493975903	0.84507042253521	0.625	37.5	86.74698795180720	13.25301204819280	62.5
47	95.96774193548390	4.032258064516130	38.18181818181820	70.94972067039110	71.73913043478260	0.70936329588015	0.70212765957446	0.73333333333333	26.66666666666670	68.53932584269660	31.46067415730340	73.33333333333330
48	98.31932773109240	1.680672268907560	33.33333333333330	68.71508379888270	68.53932584269660	0.70161701044054	0.80263157894736	0.59803921568627	40.19607843137260	80.51948051948050	19.48051948051950	59.80392156862750
49	95.58823529411770	4.411764705882350	51.16279069767440	69.83240223463690	70.96774193548390	0.71543034375807	0.825	0.62264150943396	37.73584905660380	80.82191780821920	19.17808219178080	62.26415094339620
50	94.53125	5.46875	37.25490196078430	65.92178770949720	67.72486772486770	0.66769230769230	0.75294117647058	0.61538461538461	38.46153846153850	72.0	28.00000000000000	61.53846153846150
51	95.2755905511811	4.724409448818900	44.23076923076920	72.06703910614520	73.95833333333330	0.72623914154317	0.797752808988764	0.68932038834951	31.06796116504850	76.31578947368420	23.68421052631580	68.93203883495150
52	98.29059829059830	1.709401709401710	38.70967741935480	71.50837988826820	71.1864406779661	0.73090145148968	0.84	0.61764705882352	38.23529411764710	84.4155844155844	15.58441558441560	61.76470588235290
53	99.19354838709680	0.806451612903226	34.54545454545460	70.39106145251400	72.25130890052360	0.70473484848484	0.75	0.696969696969697	30.30303030303030	71.25	28.75000000000000	69.6969696969697
54	97.63779527559060	2.362204724409450	25.0	69.27374301675980	70.89947089947090	0.692921800352024	0.72826086956521	0.69072164948453	30.92783505154640	69.51219512195120	30.48780487804880	69.0721649484536
55	92.17391304347830	7.82608695652174	40.625	68.71508379888270	68.88888888888890	0.70228666326009	0.80519480519480	0.60194174757281	39.80582524271850	80.26315789473690	19.73684210526320	60.19417475728160
56	97.52066115702480	2.479338842975210	34.48275862068970	75.41899441340780	75.82417582417580	0.75601503759398	0.79310344827586	0.72631578947368	27.36842105263160	78.57142857142860	21.42857142857140	72.63157894736840
57	99.15966386554620	0.840336134453782	36.66666666666670	74.30167597765360	75.78947368421050	0.74904507257448	0.81818181818181	0.70588235294117	29.41176470588240	79.22077922077920	20.77922077922080	70.58823529411770
58	94.9579831932773	5.042016806722690	38.33333333333330	68.71508379888270	69.23076923076920	0.68754688672168	0.70786516853932	0.67741935483871	32.25806451612900	69.76744186046510	30.23255813953490	67.74193548387100
59	94.21487603305790	5.785123966942150	44.82758620689660	65.92178770949720	65.14285714285710	0.65936329588015	0.67058823529411	0.63333333333333	36.66666666666670	68.53932584269660	31.46067415730340	63.33333333333330
60	96.58119658119660	3.418803418803420	38.70967741935480	79.88826815642460	81.81818181818180	0.79598135550516	0.81	0.826530612244898	17.34693877551020	76.5432098765432	23.45679012345680	82.6530612244898
61	96.94656488549620	3.053435114503820	29.16666666666670	68.71508379888270	67.44186046511630	0.69531053557958	0.77333333333333	0.59793814432989	40.20618556701030	79.26829268292680	20.73170731707320	59.79381443298970
62	96.63865546218490	3.361344537815130	38.33333333333330	66.4804469273743	67.03296703296700	0.67769545222279	0.77215189873417	0.59223300970873	40.77669902912620	76.31578947368420	23.68421052631580	59.22330097087380
63	97.54098360655740	2.459016393442620	33.33333333333330	72.62569832402240	72.62569832402240	0.73639240506329	0.82278481012658	0.65	35.0	82.27848101265820	17.72151898734180	65.0
64	96.0	4.0	44.44444444444440	69.27374301675980	69.61325966850830	0.70492742551566	0.79746835443038	0.61764705882352	38.23529411764710	79.22077922077920	20.77922077922080	61.76470588235290
65	97.6	2.4	33.33333333333330	67.0391061452514	67.40331491712710	0.67224310776942	0.70930232558139	0.64210526315789	35.78947368421050	70.23809523809520	29.76190476190480	64.21052631578950
66	97.36842105263160	2.631578947368420	40.0	72.06703910614520	73.40425531914890	0.72348484848484	0.77528089887640	0.696969696969697	30.30303030303030	75.0	25.0	69.6969696969697
67	98.24561403508770	1.754385964912280	38.46153846153850	75.41899441340780	76.08695652173910	0.755325814536341	0.78651685393258	0.73684210526315	26.31578947368420	77.38095238095240	22.61904761904760	73.68421052631580
68	97.54098360655740	2.459016393442620	42.10526315789470	68.15642458100560	68.1564245810056	0.68776769967246	0.75308641975308	0.62244897959183	37.75510204081630	75.30864197530870	24.69135802469140	62.24489795918370
69	94.78260869565220	5.217391304347830	45.3125	71.50837988826820	72.43243243243240	0.72107594936708	0.788235294117647	0.67	33.0	77.21518987341770	22.78481012658230	67.0
70	90.625	9.375	43.13725490196080	69.83240223463690	72.16494845360820	0.69580200501253	0.70707070707070	0.73684210526315	26.31578947368420	65.47619047619050	34.52380952380950	73.68421052631580
71	96.69421487603310	3.305785123966940	36.20689655172410	72.06703910614520	71.91011235955060	0.72489959839357	0.78048780487804	0.66666666666666	33.33333333333330	78.3132530120482	21.68674698795180	66.66666666666670
72	98.31932773109240	1.680672268907560	45.0	73.18435754189940	74.73684210526320	0.73358585858585	0.78021978021978	0.71717171717171	28.28282828282830	75.0	25.0	71.71717171717170
73	96.06299212598430	3.937007874015750	40.38461538461540	65.92178770949720	65.92178770949720	0.66022755688922	0.686046511627907	0.63440860215053	36.55913978494620	68.6046511627907	31.3953488372093	63.44086021505380
74	99.14529914529920	0.854700854700855	38.70967741935480	70.39106145251400	73.36683417085430	0.70429487179487	0.76842105263157	0.70192307692307	29.80769230769230	70.66666666666670	29.33333333333330	70.1923076923077
75	97.47899159663870	2.521008403361340	45.0	73.74301675977650	73.14285714285710	0.74823232323232	0.84210526315789	0.64646464646464	35.35353535353540	85.0	15.0	64.64646464646470
76	95.76271186440680	4.23728813559322	44.26229508196720	64.80446927374300	67.01570680628270	0.65611325611325	0.74418604651162	0.60952380952381	39.04761904761910	70.27027027027030	29.72972972972970	60.95238095238100
77	98.33333333333330	1.666666666666670	40.67796610169490	69.27374301675980	70.27027027027030	0.71071336262600	0.82278481012658	0.61320754716981	38.67924528301890	80.82191780821920	19.17808219178080	61.32075471698110
78	93.98496240601500	6.015037593984960	47.82608695652170	68.71508379888270	66.66666666666670	0.68631368631368	0.7	0.63636363636363	36.36363636363640	73.62637362637360	26.37362637362640	63.63636363636360
79	95.6140350877193	4.385964912280700	41.53846153846150	74.30167597765360	75.78947368421050	0.74746128458999	0.80898876404494	0.71287128712871	28.71287128712870	78.2051282051282	21.79487179487180	71.28712871287130
80	98.4	1.6	35.18518518518520	66.4804469273743	69.38775510204080	0.68416844349680	0.80952380952381	0.60714285714285	39.28571428571430	76.11940298507460	23.88059701492540	60.71428571428570
81	97.72727272727270	2.272727272727270	40.42553191489360	72.62569832402240	71.67630057803470	0.73422177520744	0.81578947368421	0.63917525773195	36.08247422680410	82.92682926829270	17.07317073170730	63.91752577319590
82	95.90163934426230	4.098360655737710	40.35087719298250	70.94972067039110	71.7391304347826	0.71327790375409	0.76744186046511	0.673469387755102	32.6530612244898	75.30864197530870	24.69135802469140	67.3469387755102
83	93.33333333333330	6.666666666666670	44.06779661016950	70.39106145251400	71.6577540106952	0.706034265558075	0.75280898876404	0.68367346938775	31.63265306122450	72.8395061728395	27.16049382716050	68.36734693877550
84	98.38709677419360	1.612903225806450	38.18181818181820	70.94972067039110	71.73913043478260	0.71607594936708	0.78571428571428	0.66	34.0	77.21518987341770	22.78481012658230	66.0
85	96.7741935483871	3.225806451612900	38.18181818181820	73.74301675977650	72.51461988304090	0.73925537231384	0.78481012658227	0.67391304347826	32.60869565217390	80.45977011494250	19.54022988505750	67.3913043478261
86	95.1219512195122	4.878048780487810	32.14285714285710	68.71508379888270	68.8888888888889	0.69438131313131	0.76543209876543	0.62626262626262	37.37373737373740	76.25	23.75	62.62626262626260
87	95.0	5.0	35.59322033898310	74.30167597765360	75.26881720430110	0.74497108373145	0.78651685393258	0.72164948453608	27.83505154639180	76.82926829268290	23.17073170731710	72.16494845360830
88	93.28358208955220	6.716417910447760	35.55555555555560	73.18435754189940	73.91304347826090	0.73718434343434	0.8	0.68686868686868	31.31313131313130	78.75	21.25	68.68686868686870
89	98.4	1.6	35.18518518518520	72.62569832402240	72.00000000000000	0.743888464476700	0.863013698630137	0.61764705882352	38.23529411764710	87.01298701298700	12.98701298701300	61.76470588235290
90	95.0	5.0	32.20338983050850	73.74301675977650	74.03314917127070	0.74170228815690	0.79761904761904	0.69072164948453	30.92783505154640	79.26829268292680	20.73170731707320	69.0721649484536
91	90.83969465648860	9.16030534351145	27.08333333333330	69.27374301675980	71.20418848167540	0.69122807017543	0.70833333333333	0.71578947368421	28.42105263157890	66.66666666666670	33.33333333333330	71.57894736842110
92	96.58119658119660	3.418803418803420	43.54838709677420	65.36312849162010	65.1685393258427	0.68401147626499	0.82857142857142	0.53703703703703	46.2962962962963	83.09859154929580	16.90140845070420	53.70370370370370
93	96.7479674796748	3.252032520325200	41.07142857142860	77.6536312849162	77.01149425287360	0.781203007518797	0.84810126582278	0.70526315789473	29.47368421052630	85.71428571428570	14.28571428571430	70.52631578947370
94	98.33333333333330	1.666666666666670	33.89830508474580	77.09497206703910	76.30057803468210	0.77061469265367	0.76744186046511	0.75862068965517	24.13793103448280	78.26086956521740	21.73913043478260	75.86206896551720
95	92.3076923076923	7.692307692307690	51.02040816326530	70.94972067039110	72.63157894736840	0.72520768431983	0.83132530120481	0.64485981308411	35.51401869158880	80.55555555555560	19.44444444444440	64.48598130841120
96	98.4126984126984	1.587301587301590	37.73584905660380	73.74301675977650	75.64766839378240	0.75345592070944	0.85882352941176	0.67592592592592	32.40740740740740	83.09859154929580	16.90140845070420	67.5925925925926
97	93.16239316239320	6.837606837606840	40.32258064516130	72.06703910614520	71.91011235955060	0.73349834983498	0.83116883116883	0.63366336633663	36.63366336633660	83.33333333333330	16.66666666666670	63.36633663366340
98	93.80530973451330	6.194690265486730	34.84848484848490	73.18435754189940	73.03370786516860	0.73749057078199	0.80246913580246	0.67010309278350	32.98969072164950	80.48780487804880	19.51219512195120	67.0103092783505
99	91.80327868852460	8.19672131147541	43.85964912280700	67.0391061452514	66.66666666666670	0.67122688655672	0.69411764705882	0.64130434782608	35.86956521739130	70.11494252873560	29.88505747126440	64.13043478260870
**Mean**	**96.085745980783**	**3.91425401921701**	**38.1204795334565**	**71.3519553072626**	**71.86013999144010**	**0.718904778176102**	**0.774674372995634**	**0.67341304767914**	**32.6586952320856**	**76.439650867306**	**23.560349132694**	**67.3413047679144**
Standard Deviation	2.03615894558339	2.03615894558339	6.08533336836159	3.2166902738989	3.41235950681242	0.03066570477260	0.04436736063238	0.05328741194717	5.32874119471741	4.82221170364979	4.82221170364979	5.32874119471741
**T Test Significance (Female to Male)**	**0.000000000000000**	**0.000000000000000**	**0.000000000000000**									

3.1.2 Balanced training data, no feature selection - Support Vector Machine Classifier

		B_Accuracy	B_FScore	B_ROC_AUC	B_Precision	B_Recall	B_F_Accuracy	B_F_FScore	B_F_ROC_AUC	B_F_Precision	B_F_Recall	B_F_FNR	B_F_TNR	B_F_FPR	B_F_TPR	B_M_Accuracy	B_M_FScore	B_M_ROC_AUC	B_M_Precision	B_M_Recall	B_M_FNR	B_M_TNR	B_M_FPR	B_M_TPR
0		83.79888268156430	80.0	0.82998712998713	0.816901408450704	0.783783783783784	91.62011173184360	86.23853211009180	0.912552998182920	0.824561403508772	0.903846153846154	9.615384615384620	92.1259842519685	7.874015748031500	90.38461538461540	75.97765363128490	76.24309392265190	0.762989457831325	0.811764705882353	0.71875	28.125	80.72289156626510	19.27710843373490	71.875
1		85.75418994413410	82.71186440677970	0.848734036822258	0.884057971014493	0.777070063694268	91.06145251396650	86.4406779661017	0.892609058071687	0.894736842105263	0.836065573770492	16.39344262295080	94.91525423728810	5.084745762711870	83.60655737704920	80.44692737430170	80.22598870056500	0.809550702811245	0.876543209876543	0.739583333333333	26.04166666666670	87.95180722891570	12.04819277108430	73.95833333333330
2		82.9608938547486	80.38585209003220	0.827412783295136	0.796178343949045	0.811688311688312	87.15083798882680	80.9917355371901	0.868349151567443	0.765625	0.859649122807018	14.03508771929820	87.70491803278690	12.29508196721310	85.96491228070180	78.77094972067040	80.0	0.788094040734222	0.817204301075269	0.783505154639175	21.64948453608250	79.26829268292680	20.73170731707320	78.35051546391750
3		82.40223463687150	80.37383177570100	0.821617535903250	0.811320754716981	0.796296296296296	86.59217877094970	80.95238095238100	0.848448043184885	0.836065573770492	0.784615384615385	21.53846153846150	91.22807017543860	8.771929824561400	78.46153846153850	78.2122905027933	80.0	0.780110636157908	0.795918367346939	0.804123711340206	19.58762886597940	75.60975609756100	24.39024390243900	80.41237113402060
4		83.24022346368710	79.72972972972970	0.828637890927888	0.786666666666667	0.808219178082192	92.17877094972070	86.79245283018870	0.910811629315566	0.851851851851852	0.884615384615385	11.53846153846150	93.7007874015748	6.299212598425200	88.46153846153850	74.30167597765360	75.78947368421050	0.74180225281602	0.75	0.765957446808511	23.40425531914890	71.76470588235290	28.23529411764710	76.59574468085110
5		84.07821229050280	80.13937282229970	0.829745784363822	0.851851851851852	0.756578947368421	88.26815642458100	82.05128205128200	0.857182244279019	0.872727272727273	0.774193548387097	22.58064516129030	94.01709401709400	5.982905982905980	77.41935483870970	79.88826815642460	78.82352941176470	0.799188514357054	0.8375	0.744444444444445	25.55555555555560	85.39325842696630	14.60674157303370	74.44444444444440
6		81.56424581005590	79.50310559006210	0.812625502008032	0.820512820512821	0.771084337349398	88.26815642458100	82.05128205128210	0.868338557993730	0.813559322033898	0.827586206896552	17.24137931034480	90.9090909090909	9.090909090909090	82.75862068965520	74.86033519553070	78.04878048780490	0.750652060511216	0.824742268041237	0.740740740740741	25.92592592592590	76.05633802816900	23.94366197183100	74.07407407407410
7		81.56424581005590	76.42857142857140	0.801164539143232	0.829457364341085	0.708609271523179	87.70949720670390	77.08333333333330	0.825247079964061	0.860465116279070	0.698113207547170	30.18867924528300	95.23809523809520	4.761904761904760	69.81132075471700	75.41899441340780	76.08695652173910	0.758377425044092	0.813953488372093	0.714285714285714	28.57142857142860	80.24691358024690	19.75308641975310	71.42857142857140
8		83.79888268156430	81.04575163398690	0.832715403872358	0.832214765100671	0.789808917197452	89.94413407821230	84.21052631578950	0.880735252208606	0.857142857142857	0.827586206896552	17.24137931034480	93.38842975206610	6.611570247933880	82.75862068965520	77.6536312849162	79.16666666666670	0.777588383838384	0.817204301075269	0.767676767676768	23.23232323232320	78.75	21.25	76.76767676767680
9		81.00558659217880	76.22377622377620	0.798125219950731	0.807407407407408	0.721854304635762	87.15083798882680	78.0952380952381	0.828654887432317	0.872340425531915	0.706896551724138	29.31034482758620	95.0413223140496	4.958677685950410	70.6896551724138	74.86033519553070	75.13812154696130	0.749312328082021	0.772727272727273	0.731182795698925	26.88172043010750	76.74418604651160	23.25581395348840	73.11827956989250
10		84.07821229050280	81.31147540983610	0.836936075243105	0.815789473684211	0.810457516339869	89.3854748603352	83.47826086956520	0.873446327683616	0.857142857142857	0.813559322033898	18.64406779661020	93.33333333333330	6.666666666666670	81.35593220338980	78.77094972067040	80.0	0.786608260325407	0.791666666666667	0.808510638297872	19.14893617021280	76.47058823529410	23.52941176470590	80.85106382978720
11		82.40223463687150	78.35051546391750	0.818730529595016	0.775510204081633	0.791666666666667	89.94413407821230	80.85106382978720	0.887373737373738	0.76	0.863636363636364	13.63636363636360	91.11111111111110	8.88888888888889	86.36363636363640	74.86033519553070	77.15736040609140	0.747088607594937	0.783505154639175	0.76	24.0	73.41772151898740	26.58227848101270	76.0
12		81.28491620111730	78.86435331230280	0.811532114749937	0.776397515527950	0.801282051282051	88.8268156424581	81.81818181818180	0.868768328445748	0.818181818181818	0.818181818181818	18.18181818181820	91.93548387096770	8.064516129032260	81.81818181818180	73.74301675977650	77.29468599033820	0.729372937293729	0.754716981132076	0.792079207920792	20.79207920792080	66.66666666666670	33.33333333333330	79.20792079207920
13		83.24022346368710	81.25	0.829776597406655	0.884353741496599	0.751445086705202	88.26815642458100	80.0	0.837394957983193	0.933333333333333	0.7	30.0	97.47899159663870	2.521008403361340	70.0	78.2122905027933	81.86046511627910	0.783319924912845	0.862745098039216	0.778761061946903	22.12389380530970	78.78787878787880	21.21212121212120	77.87610619469030
14		82.12290502793300	79.2207792207792	0.815808602762239	0.818791946308725	0.767295597484277	89.94413407821230	84.48275862068970	0.881920903954802	0.859649122807018	0.830508474576271	16.94915254237290	93.33333333333330	6.666666666666670	83.05084745762710	74.30167597765360	76.04166666666670	0.744746835443038	0.793478260869565	0.73	27.0	75.9493670886076	24.05063291139240	73.0
15		85.47486033519550	82.43243243243240	0.847473298262395	0.853146853146853	0.797385620915033	88.26815642458100	79.6116504854369	0.863488372093023	0.773584905660377	0.82	18.0	90.69767441860470	9.30232558139535	82.0	82.68156424581010	83.93782383419690	0.833993357179356	0.9	0.786407766990291	21.35922330097090	88.1578947368421	11.84210526315790	78.64077669902910
16		84.91620111731840	80.71428571428570	0.836960376567689	0.849624060150376	0.768707482993197	89.3854748603352	80.8080808080808	0.857056329497274	0.851063829787234	0.769230769230769	23.07692307692310	94.48818897637800	5.511811023622050	76.92307692307690	80.44692737430170	80.66298342541440	0.806829573934837	0.848837209302326	0.768421052631579	23.15789473684210	84.52380952380950	15.47619047619050	76.84210526315790
17		82.12290502793300	79.74683544303800	0.818734177215190	0.797468354430380	0.797468354430380	91.06145251396650	86.4406779661017	0.898870056497175	0.864406779661017	0.864406779661017	13.55932203389830	93.33333333333330	6.666666666666670	86.4406779661017	73.18435754189940	75.75757575757580	0.728787878787879	0.757575757575758	0.757575757575758	24.24242424242420	70.0	30.0	75.75757575757580
18		84.07821229050280	82.3529411764706	0.838240810202551	0.880794701986755	0.773255813953488	89.3854748603352	84.5528455284553	0.875815217391304	0.881355932203390	0.8125	18.75	93.91304347826090	6.086956521739130	81.25	78.77094972067040	81.0	0.797535211267606	0.880434782608696	0.75	25.0	84.50704225352110	15.49295774647890	75.0
19		84.63687150837990	81.48148148148150	0.837720959595960	0.883211678832117	0.75625	91.06145251396650	85.71428571428570	0.904444444444445	0.827586206896552	0.888888888888889	11.11111111111110	92.0	8.0	88.88888888888890	78.2122905027933	78.9189189189189	0.803243732230551	0.924050632911392	0.688679245283019	31.13207547169810	91.78082191780820	8.21917808219178	68.86792452830190
20		82.12290502793300	80.36809815950920	0.818790895713973	0.834394904458599	0.775147928994083	87.15083798882680	79.64601769911510	0.846608720433172	0.818181818181818	0.775862068965517	22.41379310344830	91.73553719008270	8.264462809917360	77.58620689655170	77.09497206703910	80.75117370892020	0.769740328563858	0.843137254901961	0.774774774774775	22.52252252252250	76.47058823529410	23.52941176470590	77.47747747747750
21		81.84357541899440	76.36363636363640	0.801673840267815	0.860655737704918	0.686274509803922	83.79888268156430	70.10309278350510	0.772442291251069	0.871794871794872	0.586206896551724	41.37931034482760	95.86776859504130	4.132231404958680	58.62068965517240	79.88826815642460	79.7752808988764	0.802255639097744	0.855421686746988	0.747368421052632	25.26315789473680	85.71428571428570	14.28571428571430	74.73684210526320
22		84.35754189944130	81.20805369127520	0.843036085150571	0.785714285714286	0.840277777777778	92.17877094972070	87.71929824561400	0.913908246225319	0.862068965517241	0.892857142857143	10.71428571428570	93.4959349593496	6.504065040650410	89.28571428571430	76.53631284916200	77.17391304347830	0.766046453546453	0.739583333333333	0.806818181818182	19.31818181818180	72.52747252747250	27.47252747252750	80.68181818181820
23		84.91620111731840	80.71428571428570	0.838622793354102	0.830882352941177	0.784722222222222	88.26815642458100	79.6116504854369	0.847629629629630	0.836734693877551	0.759259259259259	24.07407407407410	93.60000000000000	6.4	75.92592592592590	81.56424581005590	81.35593220338980	0.815730337078652	0.827586206896552	0.8	20.0	83.14606741573030	16.85393258426970	80.0
24		84.91620111731840	81.63265306122450	0.847487396324606	0.794701986754967	0.839160839160839	91.62011173184360	85.98130841121500	0.897925925925926	0.867924528301887	0.851851851851852	14.81481481481480	94.40000000000000	5.600000000000000	85.18518518518520	78.2122905027933	79.14438502673800	0.782397003745318	0.755102040816327	0.831460674157303	16.85393258426970	73.33333333333330	26.66666666666670	83.14606741573030
25		79.05027932960890	74.22680412371140	0.779421579532814	0.794117647058824	0.696774193548387	86.03351955307260	74.22680412371140	0.805333333333333	0.837209302325581	0.666666666666667	33.33333333333330	94.40000000000000	5.600000000000000	66.66666666666670	72.06703910614520	74.22680412371140	0.721820258948972	0.774193548387097	0.712871287128713	28.71287128712870	73.07692307692310	26.92307692307690	71.28712871287130
26		81.00558659217880	76.38888888888890	0.798501514426909	0.814814814814815	0.718954248366013	90.5027932960894	84.68468468468470	0.880379025363351	0.886792452830189	0.810344827586207	18.96551724137930	95.0413223140496	4.958677685950410	81.03448275862070	71.50837988826820	71.18644067796610	0.718483709273183	0.768292682926829	0.663157894736842	33.68421052631580	77.38095238095240	22.61904761904760	66.3157894736842
27		81.56424581005590	77.85234899328860	0.806990929490218	0.834532374100720	0.729559748427673	89.94413407821230	84.21052631578950	0.884167385677308	0.842105263157895	0.842105263157895	15.78947368421050	92.62295081967210	7.377049180327870	84.21052631578950	73.18435754189940	73.91304347826090	0.742424242424242	0.829268292682927	0.666666666666667	33.33333333333330	81.81818181818180	18.18181818181820	66.66666666666670
28		84.91620111731840	80.98591549295770	0.840213695968917	0.827338129496403	0.793103448275862	88.26815642458100	80.37383177570100	0.858148148148148	0.811320754716981	0.796296296296296	20.37037037037040	92.0	8.0	79.62962962962960	81.56424581005590	81.35593220338980	0.816058941058941	0.837209302325581	0.791208791208791	20.87912087912090	84.0909090909091	15.90909090909090	79.12087912087910
29		82.40223463687150	78.2006920415225	0.813458099131324	0.824817518248175	0.743421052631579	86.03351955307260	78.26086956521740	0.830716865796055	0.833333333333333	0.737704918032787	26.22950819672130	92.37288135593220	7.627118644067800	73.77049180327870	78.77094972067040	78.16091954022990	0.788399100899101	0.819277108433735	0.747252747252747	25.27472527472530	82.95454545454550	17.04545454545450	74.72527472527470
30		85.47486033519550	80.74074074074070	0.839258416002602	0.858267716535433	0.762237762237762	92.17877094972070	85.41666666666670	0.875629629629630	0.976190476190476	0.759259259259259	24.07407407407410	99.2	0.8	75.92592592592590	78.77094972067040	78.16091954022990	0.787578027465668	0.8	0.764044943820225	23.59550561797750	81.11111111111110	18.88888888888890	76.40449438202250
31		82.12290502793300	79.61783439490450	0.822119845154685	0.766871165644172	0.827814569536424	88.26815642458100	82.92682926829270	0.881803932744372	0.784615384615385	0.879310344827586	12.06896551724140	88.42975206611570	11.57024793388430	87.93103448275860	75.97765363128490	77.48691099476440	0.758314578644661	0.755102040816327	0.795698924731183	20.43010752688170	72.09302325581400	27.90697674418600	79.56989247311830
32		84.07821229050280	80.41237113402060	0.836156542056075	0.795918367346939	0.8125	89.94413407821230	83.01886792452830	0.879380053908356	0.830188679245283	0.830188679245283	16.9811320754717	92.85714285714290	7.142857142857140	83.01886792452830	78.2122905027933	78.91891891891890	0.781780719280719	0.776595744680851	0.802197802197802	19.78021978021980	76.13636363636360	23.86363636363640	80.21978021978020
33		83.5195530726257	79.72508591065290	0.825426430734896	0.840579710144928	0.758169934640523	90.5027932960894	82.4742268041237	0.855571847507331	0.952380952380952	0.727272727272727	27.27272727272730	98.38709677419360	1.612903225806450	72.72727272727270	76.53631284916200	78.35051546391750	0.764298311917360	0.791666666666667	0.775510204081633	22.44897959183670	75.30864197530870	24.69135802469140	77.55102040816330
34		84.63687150837990	82.31511254019290	0.842966695186488	0.831168831168831	0.815286624203822	92.73743016759780	89.25619834710740	0.920588235294118	0.885245901639344	0.9	10.0	94.11764705882350	5.88235294117647	90.0	76.53631284916200	77.89473684210530	0.765589640432487	0.795698924731183	0.762886597938144	23.71134020618560	76.82926829268290	23.17073170731710	76.28865979381440
35		82.9608938547486	79.86798679867990	0.833463745435576	0.751552795031056	0.852112676056338	89.94413407821230	85.24590163934430	0.891316526610644	0.838709677419355	0.866666666666667	13.33333333333330	91.59663865546220	8.403361344537820	86.66666666666670	75.97765363128490	76.24309392265190	0.766092532059341	0.696969696969697	0.841463414634146	15.85365853658540	69.0721649484536	30.92783505154640	84.14634146341460
36		83.24022346368710	79.02097902097900	0.821032088812106	0.837037037037037	0.748344370860927	91.06145251396650	84.31372549019610	0.874780058651026	0.914893617021277	0.781818181818182	21.81818181818180	96.7741935483871	3.225806451612900	78.18181818181820	75.41899441340780	76.08695652173910	0.756149598393574	0.795454545454545	0.729166666666667	27.08333333333330	78.3132530120482	21.68674698795180	72.91666666666670
37		84.63687150837990	83.28267477203650	0.844492373093273	0.872611464968153	0.796511627906977	86.03351955307260	78.26086956521740	0.830716865796055	0.833333333333333	0.737704918032787	26.22950819672130	92.37288135593220	7.627118644067800	73.77049180327870	83.24022346368710	85.98130841121500	0.833532061473238	0.893203883495146	0.828828828828829	17.11711711711710	83.82352941176470	16.17647058823530	82.88288288288290
38		84.63687150837990	81.60535117056860	0.838446064873020	0.903703703703704	0.743902439024390	89.3854748603352	82.56880733944960	0.866048317515099	0.865384615384615	0.789473684210526	21.05263157894740	94.26229508196720	5.737704918032790	78.94736842105260	79.88826815642460	81.05263157894740	0.818146417445483	0.927710843373494	0.719626168224299	28.03738317757010	91.66666666666670	8.333333333333330	71.96261682242990
39		82.40223463687150	79.47882736156350	0.818149042229677	0.871428571428571	0.730538922155689	87.15083798882680	79.64601769911500	0.837262200165426	0.882352941176471	0.725806451612903	27.41935483870970	94.87179487179490	5.128205128205130	72.58064516129030	77.6536312849162	79.38144329896910	0.785585585585586	0.865168539325843	0.733333333333333	26.66666666666670	83.78378378378380	16.21621621621620	73.33333333333330
40		82.40223463687150	78.35051546391750	0.816665054647451	0.791666666666667	0.775510204081633	91.62011173184360	85.71428571428570	0.911240310077519	0.818181818181818	0.9	10.0	92.24806201550390	7.751937984496120	90.0	73.18435754189940	74.19354838709680	0.733718883580589	0.775280898876405	0.711340206185567	28.86597938144330	75.60975609756100	24.39024390243900	71.1340206185567
41		82.68156424581010	79.05405405405410	0.819334440470107	0.8125	0.769736842105263	87.70949720670390	81.03448275862070	0.849117728149986	0.870370370370370	0.758064516129032	24.19354838709680	94.01709401709400	5.982905982905980	75.80645161290320	77.6536312849162	77.77777777777780	0.776529338327091	0.777777777777778	0.777777777777778	22.22222222222220	77.52808988764050	22.47191011235960	77.77777777777780
42		82.12290502793300	77.62237762237760	0.809086561453850	0.834586466165414	0.725490196078431	87.15083798882680	79.27927927927930	0.848141695702671	0.8	0.785714285714286	21.42857142857140	91.05691056910570	8.94308943089431	78.57142857142860	77.09497206703910	76.57142857142860	0.778287654010561	0.858974358974359	0.690721649484536	30.92783505154640	86.58536585365850	13.41463414634150	69.0721649484536
43		84.35754189944130	80.82191780821920	0.833650672759584	0.867647058823529	0.756410256410256	91.06145251396650	84.90566037735850	0.884897360703812	0.882352941176471	0.818181818181818	18.18181818181820	95.16129032258070	4.838709677419360	81.81818181818180	77.6536312849162	78.49462365591400	0.784463061690784	0.858823529411765	0.722772277227723	27.72277227722770	84.61538461538460	15.38461538461540	72.27722772277230
44		84.35754189944130	82.38993710691820	0.84084245042091	0.834394904458599	0.813664596273292	88.8268156424581	81.48148148148150	0.867407407407408	0.814814814814815	0.814814814814815	18.51851851851850	92.0	8.0	81.48148148148150	79.88826815642460	82.85714285714290	0.795430944963655	0.844660194174757	0.813084112149533	18.69158878504670	77.77777777777780	22.22222222222220	81.30841121495330
45		82.68156424581010	79.19463087248320	0.818417721518987	0.842857142857143	0.746835443037975	88.8268156424581	83.33333333333330	0.869037772263579	0.862068965517241	0.806451612903226	19.35483870967740	93.16239316239320	6.837606837606840	80.64516129032260	76.53631284916200	76.40449438202250	0.769829317269076	0.829268292682927	0.708333333333333	29.16666666666670	83.13253012048190	16.86746987951810	70.83333333333330
46		84.35754189944130	82.38993710691820	0.840302029259085	0.845161290322581	0.803680981595092	87.70949720670390	81.03448275862070	0.859717868338558	0.810344827586207	0.810344827586207	18.96551724137930	90.9090909090909	9.090909090909090	81.03448275862070	81.00558659217880	83.16831683168320	0.812162162162162	0.865979381443299	0.8	20.0	82.43243243243240	17.56756756756760	80.0
47		85.47486033519550	83.64779874213840	0.851096491228070	0.886666666666667	0.791666666666667	92.73743016759780	88.49557522123890	0.922287390029326	0.862068965517241	0.909090909090909	9.090909090909090	93.54838709677420	6.451612903225810	90.9090909090909	78.2122905027933	80.97560975609760	0.799074818986324	0.902173913043478	0.734513274336283	26.54867256637170	86.36363636363640	13.63636363636360	73.45132743362830
48		82.68156424581010	79.33333333333330	0.822147435897436	0.793333333333333	0.793333333333333	87.15083798882680	80.67226890756300	0.856779661016949	0.8	0.813559322033898	18.64406779661020	90.0	10.0	81.35593220338980	78.2122905027933	78.45303867403320	0.782155344655345	0.788888888888889	0.780219780219780	21.97802197802200	78.4090909090909	21.59090909090910	78.02197802197800
49		82.68156424581010	78.6206896551724	0.817096970278658	0.820143884892086	0.754966887417219	88.26815642458100	79.6116504854369	0.851078167115903	0.82	0.773584905660377	22.64150943396230	92.85714285714290	7.142857142857140	77.35849056603770	77.09497206703910	78.07486631016040	0.773683547493071	0.820224719101124	0.744897959183674	25.51020408163270	80.24691358024690	19.75308641975310	74.48979591836740
50		85.75418994413410	82.94314381270900	0.853906425612537	0.826666666666667	0.832214765100671	91.62011173184360	86.72566371681420	0.913703703703704	0.830508474576271	0.907407407407407	9.25925925925926	92.0	8.0	90.74074074074080	79.88826815642460	80.64516129032260	0.799498746867168	0.824175824175824	0.789473684210526	21.05263157894740	80.95238095238100	19.04761904761900	78.94736842105260
51		88.26815642458100	85.51724137931030	0.876745010800529	0.867132867132867	0.843537414965986	94.41340782122900	90.74074074074080	0.933703703703704	0.907407407407407	0.907407407407407	9.25925925925926	96.0	4.0	90.74074074074080	82.12290502793300	82.41758241758240	0.821830457614404	0.842696629213483	0.806451612903226	19.35483870967740	83.72093023255820	16.27906976744190	80.64516129032260
52		85.75418994413410	83.16831683168320	0.851522641569224	0.863013698630137	0.802547770700637	93.29608938547490	87.5	0.920293359123146	0.857142857142857	0.893617021276596	10.63829787234040	94.6969696969697	5.303030303030300	89.36170212765960	78.2122905027933	81.15942028985510	0.787615283267457	0.865979381443299	0.763636363636364	23.63636363636360	81.15942028985510	18.84057971014490	76.36363636363640
53		84.63687150837990	81.22866894197950	0.838484179543782	0.838028169014085	0.788079470198676	88.8268156424581	81.48148148148150	0.854516956397834	0.88	0.758620689655172	24.13793103448280	95.0413223140496	4.958677685950410	75.86206896551720	80.44692737430170	81.08108108108110	0.804388597149287	0.815217391304348	0.806451612903226	19.35483870967740	80.23255813953490	19.76744186046510	80.64516129032260
54		82.68156424581010	79.33333333333330	0.829819916985325	0.748427672955975	0.843971631205674	86.59217877094970	79.3103448275862	0.871441550575409	0.71875	0.884615384615385	11.53846153846150	85.8267716535433	14.17322834645670	88.46153846153850	78.77094972067040	79.34782608695650	0.787890137328340	0.768421052631579	0.820224719101124	17.97752808988760	75.55555555555560	24.44444444444440	82.02247191011240
55		84.07821229050280	81.55339805825240	0.836597268434896	0.828947368421053	0.802547770700637	87.70949720670390	80.35714285714290	0.853753235547886	0.818181818181818	0.789473684210526	21.05263157894740	91.80327868852460	8.19672131147541	78.94736842105260	80.44692737430170	82.23350253807110	0.803734177215190	0.835051546391753	0.81	19.0	79.74683544303800	20.25316455696200	81.0
56		81.56424581005590	77.08333333333330	0.808313862928349	0.770833333333333	0.770833333333333	86.59217877094970	73.91304347826090	0.840201778385773	0.693877551020408	0.790697674418605	20.93023255813950	88.97058823529410	11.02941176470590	79.06976744186050	76.53631284916200	78.57142857142860	0.765803503427266	0.810526315789474	0.762376237623762	23.76237623762380	76.92307692307690	23.07692307692310	76.23762376237620
57		79.60893854748600	74.91408934707900	0.785925320593412	0.858267716535433	0.664634146341463	89.3854748603352	83.1858407079646	0.861938240970499	0.921568627450980	0.758064516129032	24.19354838709680	96.58119658119660	3.418803418803420	75.80645161290320	69.83240223463690	69.66292134831460	0.713012477718360	0.815789473684211	0.607843137254902	39.21568627450980	81.81818181818180	18.18181818181820	60.78431372549020
58		82.9608938547486	78.29181494661920	0.816495937831155	0.833333333333333	0.738255033557047	92.17877094972070	86.27450980392160	0.904028799019608	0.862745098039216	0.862745098039216	13.72549019607840	94.53125	5.46875	86.27450980392160	73.74301675977650	73.74301675977650	0.744142101284959	0.814814814814815	0.673469387755102	32.6530612244898	81.48148148148150	18.51851851851850	67.3469387755102
59		85.19553072625700	82.39202657807310	0.846224853577795	0.843537414965986	0.805194805194805	89.3854748603352	82.8828828828829	0.886343216531896	0.793103448275862	0.867924528301887	13.20754716981130	90.47619047619050	9.523809523809520	86.79245283018870	81.00558659217880	82.10526315789470	0.815625793348566	0.876404494382023	0.772277227722772	22.77227722772280	85.8974358974359	14.10256410256410	77.22772277227720
60		83.79888268156430	81.29032258064520	0.836154949784792	0.802547770700637	0.823529411764706	87.15083798882680	78.0952380952381	0.860675039246468	0.732142857142857	0.836734693877551	16.3265306122449	88.46153846153850	11.53846153846150	83.6734693877551	80.44692737430170	82.92682926829270	0.801987179487180	0.841584158415842	0.817307692307692	18.26923076923080	78.66666666666670	21.33333333333330	81.73076923076920
61		82.68156424581010	79.05405405405410	0.820288461538462	0.801369863013699	0.78	87.15083798882680	80.67226890756300	0.860074095183813	0.786885245901639	0.827586206896552	17.24137931034480	89.25619834710740	10.74380165289260	82.75862068965520	78.2122905027933	77.96610169491530	0.783045977011494	0.811764705882353	0.75	25.0	81.60919540229890	18.39080459770120	75.0
62		85.75418994413410	83.16831683168320	0.857481381178064	0.807692307692308	0.857142857142857	92.17877094972070	87.5	0.913196480938416	0.859649122807018	0.909090909090909	10.90909090909090	93.54838709677420	6.451612903225810	89.0909090909091	79.3296089385475	80.6282722513089	0.792041479260370	0.777777777777778	0.836956521739131	16.30434782608700	74.71264367816090	25.28735632183910	83.69565217391310
63		83.24022346368710	79.59183673469390	0.822698397648621	0.866666666666667	0.735849056603774	87.15083798882680	78.50467289719630	0.830932203389830	0.875	0.711864406779661	28.8135593220339	95.0	5.0	71.1864406779661	79.3296089385475	80.21390374331550	0.799050632911392	0.862068965517241	0.75	25.0	84.81012658227850	15.18987341772150	75.0
64		83.79888268156430	80.4054054054054	0.841299563089255	0.757961783439491	0.856115107913669	89.3854748603352	82.88288288288290	0.881925925925926	0.807017543859649	0.851851851851852	14.81481481481480	91.2	8.800000000000000	85.18518518518520	78.2122905027933	78.9189189189189	0.785794743429287	0.73	0.858823529411765	14.11764705882350	71.27659574468090	28.72340425531920	85.88235294117650
65		81.84357541899440	79.75077881619940	0.815229668674699	0.825806451612903	0.771084337349398	90.5027932960894	85.95041322314050	0.895518207282913	0.852459016393443	0.866666666666667	13.33333333333330	92.43697478991600	7.563025210084030	86.66666666666670	73.18435754189940	76.0	0.735202894804859	0.808510638297872	0.716981132075472	28.30188679245280	75.34246575342470	24.65753424657530	71.69811320754720
66		82.40223463687150	80.49535603715170	0.821849929211892	0.8125	0.797546012269939	87.15083798882680	80.99173553719010	0.855029174770770	0.816666666666667	0.803278688524590	19.67213114754100	90.67796610169490	9.322033898305090	80.32786885245900	77.6536312849162	80.19801980198020	0.773682200152788	0.81	0.794117647058824	20.58823529411760	75.32467532467530	24.67532467532470	79.41176470588240
67		82.12290502793300	78.2312925170068	0.814703989703990	0.787671232876712	0.777027027027027	88.8268156424581	79.59183673469390	0.859497645211931	0.795918367346939	0.795918367346939	20.40816326530610	92.3076923076923	7.692307692307690	79.59183673469390	75.41899441340780	77.55102040816330	0.752588383838384	0.783505154639175	0.767676767676768	23.23232323232320	73.75	26.25	76.76767676767680
68		83.24022346368710	81.0126582278481	0.832251082251082	0.790123456790123	0.831168831168831	86.59217877094970	81.25	0.859525778880618	0.787878787878788	0.838709677419355	16.12903225806450	88.03418803418800	11.96581196581200	83.87096774193550	79.88826815642460	80.85106382978720	0.798100949525238	0.791666666666667	0.826086956521739	17.39130434782610	77.01149425287360	22.98850574712640	82.6086956521739
69		84.63687150837990	81.60535117056860	0.839045443005839	0.853146853146853	0.782051282051282	89.3854748603352	82.88288288288290	0.870721886683923	0.851851851851852	0.807017543859649	19.29824561403510	93.44262295081970	6.557377049180330	80.7017543859649	79.88826815642460	80.85106382978720	0.802588383838384	0.853932584269663	0.767676767676768	23.23232323232320	83.75	16.25	76.76767676767680
70		84.63687150837990	81.84818481848190	0.841322892793481	0.832214765100671	0.805194805194805	92.73743016759780	88.28828828828830	0.905884867483614	0.924528301886793	0.844827586206897	15.51724137931030	96.69421487603310	3.305785123966940	84.48275862068970	76.53631284916200	78.125	0.764118975903614	0.78125	0.78125	21.875	74.69879518072290	25.30120481927710	78.125
71		85.19553072625700	82.39202657807310	0.844470662262073	0.898550724637681	0.760736196319019	89.94413407821230	83.92857142857140	0.866211745244003	0.94	0.758064516129032	24.19354838709680	97.43589743589740	2.564102564102560	75.80645161290320	80.44692737430170	81.4814814814815	0.810675298299061	0.875	0.762376237623762	23.76237623762380	85.8974358974359	14.10256410256410	76.23762376237620
72		84.07821229050280	83.38192419825070	0.852686115096445	0.752631578947368	0.934640522875817	88.26815642458100	82.64462809917360	0.900269541778976	0.735294117647059	0.943396226415094	5.660377358490570	85.71428571428570	14.28571428571430	94.33962264150940	79.88826815642460	83.78378378378380	0.781455696202532	0.762295081967213	0.93	7.000000000000000	63.29113924050630	36.70886075949370	93.0
73		83.5195530726257	80.26755852842810	0.828742228598757	0.821917808219178	0.784313725490196	89.3854748603352	82.24299065420560	0.867741935483871	0.846153846153846	0.8	20.0	93.54838709677420	6.451612903225810	80.0	77.6536312849162	79.16666666666670	0.776643990929705	0.808510638297872	0.775510204081633	22.44897959183670	77.77777777777780	22.22222222222220	77.55102040816330
74		79.88826815642460	77.0700636942675	0.797863860991551	0.751552795031056	0.790849673202614	87.70949720670390	81.35593220338980	0.867773943054357	0.786885245901639	0.842105263157895	15.78947368421050	89.34426229508200	10.65573770491800	84.21052631578950	72.06703910614520	74.48979591836740	0.717557730923695	0.73	0.760416666666667	23.95833333333330	67.46987951807230	32.53012048192770	76.04166666666670
75		83.24022346368710	80.13245033112580	0.831836391832515	0.775641025641026	0.828767123287671	89.3854748603352	82.56880733944950	0.895736434108527	0.762711864406780	0.9	10.0	89.14728682170540	10.85271317829460	90.0	77.09497206703910	78.75647668393780	0.769327309236948	0.783505154639175	0.791666666666667	20.83333333333330	74.69879518072290	25.30120481927710	79.16666666666670
76		84.07821229050280	81.7891373801917	0.837189090104611	0.831168831168831	0.805031446540881	89.3854748603352	83.47826086956520	0.892444444444444	0.786885245901639	0.888888888888889	11.11111111111110	89.60000000000000	10.4	88.88888888888890	78.77094972067040	80.8080808080808	0.793114543114543	0.860215053763441	0.761904761904762	23.80952380952380	82.43243243243240	17.56756756756760	76.19047619047620
77		80.72625698324020	78.63777089783280	0.805577450578554	0.783950617283951	0.788819875776398	87.70949720670390	79.62962962962960	0.847343205574913	0.826923076923077	0.767857142857143	23.21428571428570	92.6829268292683	7.317073170731710	76.78571428571430	73.74301675977650	78.13953488372090	0.724324324324324	0.763636363636364	0.8	20.0	64.86486486486490	35.13513513513510	80.0
78		83.79888268156430	78.98550724637680	0.822144864588656	0.879032258064516	0.717105263157895	88.8268156424581	80.3921568627451	0.843255680184067	0.911111111111111	0.719298245614035	28.07017543859650	96.72131147540980	3.278688524590160	71.9298245614035	78.77094972067040	78.16091954022990	0.792418546365915	0.860759493670886	0.715789473684211	28.42105263157890	86.90476190476190	13.09523809523810	71.57894736842110
79		84.35754189944130	80.55555555555560	0.833381322583741	0.846715328467153	0.768211920529801	93.85474860335200	90.59829059829060	0.928319209039548	0.913793103448276	0.898305084745763	10.16949152542370	95.83333333333330	4.166666666666670	89.83050847457630	74.86033519553070	73.6842105263158	0.750437281359320	0.797468354430380	0.684782608695652	31.52173913043480	81.60919540229890	18.39080459770120	68.47826086956520
80		82.68156424581010	79.19463087248320	0.825610072689512	0.766233766233766	0.819444444444444	89.94413407821230	83.63636363636360	0.895063597819503	0.793103448275862	0.884615384615385	11.53846153846150	90.5511811023622	9.448818897637790	88.46153846153850	75.41899441340780	76.59574468085110	0.753373313343328	0.75	0.782608695652174	21.73913043478260	72.41379310344830	27.58620689655170	78.26086956521740
81		86.59217877094970	83.09859154929580	0.859099040494389	0.836879432624114	0.825174825174825	92.17877094972070	86.00000000000000	0.889787361485475	0.914893617021277	0.811320754716981	18.86792452830190	96.82539682539680	3.174603174603170	81.13207547169810	81.00558659217880	81.5217391304348	0.809925093632959	0.797872340425532	0.833333333333333	16.66666666666670	78.65168539325840	21.34831460674160	83.33333333333330
82		83.24022346368710	81.70731707317070	0.830931072381850	0.822085889570552	0.812121212121212	90.5027932960894	86.8217054263566	0.908168935815504	0.823529411764706	0.918032786885246	8.19672131147541	89.83050847457630	10.16949152542370	91.80327868852460	75.97765363128490	78.39195979899500	0.761666666666667	0.821052631578947	0.75	25.0	77.33333333333330	22.66666666666670	75.0
83		79.88826815642460	76.62337662337660	0.793706626105143	0.781456953642384	0.751592356687898	84.91620111731840	76.10619469026550	0.821102878312910	0.781818181818182	0.741379310344828	25.86206896551720	90.08264462809920	9.917355371900830	74.13793103448280	74.86033519553070	76.92307692307690	0.747537878787879	0.78125	0.757575757575758	24.24242424242420	73.75	26.25	75.75757575757580
84		81.28491620111730	76.49122807017540	0.801178510645130	0.801470588235294	0.731543624161074	87.70949720670390	79.24528301886790	0.848888888888889	0.807692307692308	0.777777777777778	22.22222222222220	92.0	8.0	77.77777777777780	74.86033519553070	74.86033519553070	0.751441102756892	0.797619047619048	0.705263157894737	29.47368421052630	79.76190476190480	20.23809523809520	70.52631578947370
85		80.16759776536310	75.4325259515571	0.790036548546004	0.813432835820896	0.703225806451613	83.24022346368710	75.40983606557380	0.807201086956522	0.793103448275862	0.71875	28.125	89.56521739130440	10.43478260869570	71.875	77.09497206703910	75.44910179640720	0.772290209790210	0.828947368421053	0.692307692307692	30.76923076923080	85.22727272727270	14.77272727272730	69.23076923076920
86		82.9608938547486	78.13620071684590	0.814745735692651	0.865079365079365	0.712418300653595	88.26815642458100	77.89473684210530	0.824266862170088	0.925	0.672727272727273	32.72727272727270	97.58064516129030	2.419354838709680	67.27272727272730	77.6536312849162	78.26086956521740	0.780927185689091	0.837209302325581	0.734693877551020	26.53061224489800	82.71604938271610	17.28395061728400	73.46938775510210
87		85.19553072625700	81.66089965397920	0.840918428621093	0.907692307692308	0.742138364779874	89.94413407821230	82.0	0.853876306620209	0.931818181818182	0.732142857142857	26.78571428571430	97.5609756097561	2.439024390243900	73.21428571428570	80.44692737430170	81.4814814814815	0.814575881451201	0.895348837209302	0.747572815533981	25.24271844660190	88.1578947368421	11.84210526315790	74.75728155339810
88		84.63687150837990	80.28673835125450	0.833140283140283	0.854961832061069	0.756756756756757	92.73743016759780	85.71428571428570	0.886420722135008	0.928571428571429	0.795918367346939	20.40816326530610	97.6923076923077	2.307692307692310	79.59183673469390	76.53631284916200	77.6595744680851	0.768686868686869	0.820224719101124	0.737373737373737	26.26262626262630	80.0	20.0	73.73737373737370
89		86.31284916201120	85.71428571428570	0.869299326822793	0.798913043478261	0.924528301886793	86.59217877094970	80.64516129032260	0.873257839721254	0.735294117647059	0.892857142857143	10.71428571428570	85.36585365853660	14.63414634146340	89.28571428571430	86.03351955307260	88.58447488584470	0.845873786407767	0.836206896551724	0.941747572815534	5.825242718446600	75.0	25.0	94.1747572815534
90		82.9608938547486	79.18088737201360	0.819639310149131	0.878787878787879	0.720496894409938	87.70949720670390	78.84615384615380	0.837616144018583	0.854166666666667	0.732142857142857	26.78571428571430	94.3089430894309	5.691056910569110	73.21428571428570	78.2122905027933	79.36507936507940	0.796332046332046	0.892857142857143	0.714285714285714	28.57142857142860	87.83783783783780	12.16216216216220	71.42857142857140
91		82.40223463687150	79.87220447284350	0.819557934942550	0.868055555555556	0.739644970414201	89.3854748603352	83.76068376068380	0.874719887955182	0.859649122807018	0.816666666666667	18.33333333333330	93.27731092436970	6.722689075630250	81.66666666666670	75.41899441340780	77.55102040816330	0.770052424639581	0.873563218390805	0.697247706422018	30.27522935779820	84.28571428571430	15.71428571428570	69.72477064220180
92		83.5195530726257	81.15015974440890	0.831898734177215	0.819354838709677	0.803797468354430	90.5027932960894	86.82170542635660	0.898369565217391	0.861538461538462	0.875	12.5	92.17391304347830	7.82608695652174	87.5	76.53631284916200	77.17391304347830	0.765894868585732	0.788888888888889	0.755319148936170	24.46808510638300	77.64705882352940	22.35294117647060	75.53191489361700
93		84.63687150837990	82.31511254019290	0.842966695186488	0.831168831168831	0.815286624203822	86.03351955307260	78.63247863247860	0.839830508474576	0.793103448275862	0.779661016949153	22.03389830508470	90.0	10.0	77.96610169491530	83.24022346368710	84.5360824742268	0.831947593852356	0.854166666666667	0.836734693877551	16.3265306122449	82.71604938271610	17.28395061728400	83.6734693877551
94		80.16759776536310	75.43252595155710	0.790285966516379	0.8515625	0.677018633540373	86.59217877094970	76.92307692307690	0.815156988052237	0.930232558139535	0.655737704918033	34.42622950819670	97.45762711864410	2.542372881355930	65.57377049180330	73.74301675977650	74.59459459459460	0.743734177215190	0.811764705882353	0.69	31.0	79.74683544303800	20.25316455696200	69.0
95		86.87150837988830	83.84879725085910	0.858522170601435	0.924242424242424	0.767295597484277	88.8268156424581	78.26086956521740	0.830405814657783	0.9	0.692307692307692	30.76923076923080	96.8503937007874	3.149606299212600	69.23076923076920	84.91620111731840	86.4321608040201	0.860202492211838	0.934782608695652	0.803738317757009	19.62616822429910	91.66666666666670	8.333333333333330	80.37383177570090
96		82.40223463687150	79.6116504854369	0.820419513745431	0.798701298701299	0.793548387096774	87.15083798882680	78.89908256880740	0.854073075771189	0.767857142857143	0.811320754716981	18.86792452830190	89.68253968253970	10.31746031746030	81.13207547169810	77.6536312849162	80.0	0.775273745861981	0.816326530612245	0.784313725490196	21.56862745098040	76.62337662337660	23.37662337662340	78.43137254901960
97		80.44692737430170	78.125	0.803069620253165	0.771604938271605	0.791139240506329	84.91620111731840	77.68595041322320	0.855185185185185	0.701492537313433	0.870370370370370	12.96296296296300	84.0	16.0	87.03703703703700	75.97765363128490	78.39195979899500	0.761666666666667	0.821052631578947	0.75	25.0	77.33333333333330	22.66666666666670	75.0
98		83.5195530726257	79.2982456140351	0.822765469824293	0.862595419847328	0.733766233766234	90.5027932960894	84.40366972477070	0.875890567113138	0.901960784313726	0.793103448275862	20.68965517241380	95.86776859504130	4.132231404958680	79.3103448275862	76.53631284916200	76.13636363636360	0.770645080321285	0.8375	0.697916666666667	30.20833333333330	84.33734939759040	15.66265060240960	69.79166666666670
99		79.3296089385475	75.97402597402600	0.789501640368692	0.866666666666667	0.676300578034682	85.47486033519550	78.33333333333330	0.823960554371002	0.886792452830189	0.701492537313433	29.85074626865670	94.64285714285710	5.357142857142860	70.1492537313433	73.18435754189940	74.46808510638300	0.747996898423365	0.853658536585366	0.660377358490566	33.9622641509434	83.56164383561640	16.43835616438360	66.0377358490566
	**Mean**	**83.2988826815642**	**80.0694580964366**	**0.826985731330461**	**0.827097211380240**	**0.779105571431143**	**89.03351955307260**	**82.1803565054385**	**0.868668149365876**	**0.842210026891838**	**0.808777211362842**	**19.1222788637158**	**92.8559087368911**	**7.144091263108880**	**80.87772113628420**	**77.5642458100559**	**78.7777216339719**	**0.778026924590898**	**0.818960825445748**	**0.762426191216315**	**23.7573808783685**	**79.36276579654820**	**20.6372342034518**	**76.2426191216315**
	**Standard Deviation**	1.74748015596268	2.25123438279801	0.018501240025074	0.038434086362044	0.047066846423675	2.22991290084168	3.72465062682666	0.030143173744351	0.056399939763697	0.068970374525083	6.8970374525083	3.00145593774029	3.001455937740290	6.89703745250830	2.93229037240431	3.1036299483256	0.029462839565768	0.046178414367669	0.052603591900228	5.26035919002281	5.69148578668770	5.6914857866877	5.26035919002281
	**T Test Significance (Female to Male)**						**0.000000000000000**	**0.004319681431129**	**0.000000000000004**	**0.142669317291181**	**0.000645638429056**	**0.000000124431005**	**0.000000000000000**	**0.000000000000000**	**0.000645638429056**									

3.1.2 Balanced training data, no feature selection - Gaussian Naive Bayes Classifier

		B_Accuracy	B_FScore	B_ROC_AUC	B_Precision	B_Recall	B_F_Accuracy	B_F_FScore	B_F_ROC_AUC	B_F_Precision	B_F_Recall	B_F_FNR
0		75.69832402234640	65.0602409638554	0.731329113924051	0.890109890109890	0.512658227848101	79.88826815642460	59.09090909090910	0.708263305322129	0.928571428571429	0.433333333333333	56.66666666666670
1		74.02234636871510	60.75949367088610	0.701327505261454	0.782608695652174	0.496551724137931	78.2122905027933	46.57534246575340	0.647135416666667	0.772727272727273	0.333333333333333	66.66666666666670
2		78.49162011173190	67.78242677824270	0.752510760401722	0.941860465116279	0.529411764705882	83.24022346368710	62.5	0.727272727272727	1.0	0.454545454545455	54.54545454545450
3		74.58100558659220	63.45381526104420	0.720789482001201	0.877777777777778	0.496855345911950	77.6536312849162	48.71794871794870	0.658469945355191	0.904761904761905	0.333333333333333	66.66666666666670
4		72.62569832402240	62.3076923076923	0.705145202020202	0.81	0.50625	75.97765363128490	51.68539325842700	0.668389853873725	0.851851851851852	0.370967741935484	62.90322580645160
5		74.86033519553070	61.86440677966100	0.711473619986513	0.839080459770115	0.489932885906040	78.77094972067040	47.22222222222220	0.651041666666667	0.809523809523810	0.333333333333333	66.66666666666670
6		75.97765363128490	64.75409836065580	0.728901641957596	0.868131868131868	0.516339869281046	82.68156424581010	61.72839506172840	0.723481481481482	0.925925925925926	0.462962962962963	53.70370370370370
7		75.41899441340780	62.39316239316240	0.714622641509434	0.829545454545455	0.5	78.2122905027933	56.17977528089890	0.691526610644258	0.862068965517241	0.416666666666667	58.33333333333330
8		72.90502793296090	59.75103734439830	0.701289466198919	0.878048780487805	0.452830188679245	79.88826815642460	50.0	0.664442700156986	0.782608695652174	0.367346938775510	63.26530612244900
9		74.02234636871510	60.42553191489360	0.705196875498167	0.865853658536585	0.464052287581699	78.2122905027933	48.0	0.653938304881701	0.818181818181818	0.339622641509434	66.0377358490566
10		74.30167597765360	59.29203539823010	0.696563641105895	0.797619047619048	0.471830985915493	77.6536312849162	45.94594594594590	0.644504342617550	0.809523809523810	0.320754716981132	67.9245283018868
11		77.6536312849162	66.10169491525420	0.737843551797040	0.838709677419355	0.545454545454545	82.12290502793300	51.51515151515150	0.673963515754561	0.809523809523810	0.377777777777778	62.22222222222220
12		76.81564245810060	65.27196652719670	0.731745929180667	0.838709677419355	0.534246575342466	79.88826815642460	55.00000000000000	0.688298490127759	0.916666666666667	0.392857142857143	60.71428571428570
13		74.30167597765360	63.2	0.720867976820358	0.897727272727273	0.487654320987654	74.86033519553070	41.55844155844160	0.627259887005650	0.888888888888889	0.271186440677966	72.88135593220340
14		72.62569832402240	55.04587155963300	0.680769230769231	0.882352941176471	0.4	78.77094972067040	47.22222222222220	0.651650514839491	0.85	0.326923076923077	67.3076923076923
15		76.81564245810060	63.11111111111110	0.725579150579151	0.922077922077922	0.479729729729730	81.56424581005590	60.24096385542170	0.715199884958297	0.961538461538462	0.438596491228070	56.14035087719300
16		77.09497206703910	64.65517241379310	0.729679622702879	0.842696629213483	0.524475524475525	81.56424581005590	53.52112676056340	0.683965348152991	0.76	0.413043478260870	58.69565217391310
17		70.94972067039110	60.0	0.694265918424930	0.838709677419355	0.467065868263473	78.2122905027933	53.01204819277110	0.676103368176539	0.814814814814815	0.392857142857143	60.71428571428570
18		76.53631284916200	66.9291338582677	0.740849890674019	0.876288659793814	0.541401273885350	77.09497206703910	49.38271604938270	0.657624633431085	0.769230769230769	0.363636363636364	63.63636363636360
19		78.2122905027933	65.78947368421050	0.739841346932168	0.903614457831325	0.517241379310345	83.79888268156430	57.97101449275360	0.707296849087894	0.833333333333333	0.444444444444444	55.55555555555560
20		72.90502793296090	60.40816326530610	0.700050774308200	0.831460674157303	0.474358974358974	79.3296089385475	54.32098765432100	0.684233449477352	0.88	0.392857142857143	60.71428571428570
21		73.46368715083800	60.90534979423870	0.709509096068354	0.902439024390244	0.459627329192547	75.97765363128490	49.41176470588240	0.659419283134204	0.875	0.344262295081967	65.57377049180330
22		79.05027932960890	70.8171206225681	0.766914191419142	0.900990099009901	0.583333333333333	78.2122905027933	57.14285714285720	0.695658263305322	0.838709677419355	0.433333333333333	56.66666666666670
23		77.37430167597770	68.48249027237350	0.749388209121246	0.862745098039216	0.567741935483871	84.35754189944130	62.16216216216220	0.729529335912315	0.851851851851852	0.489361702127660	51.06382978723400
24		75.13966480446930	62.76150627615060	0.715283140283140	0.824175824175824	0.506756756756757	77.09497206703910	43.83561643835620	0.634161114476075	0.761904761904762	0.307692307692308	69.23076923076920
25		77.09497206703910	65.2542372881356	0.733038252778496	0.855555555555556	0.527397260273973	80.44692737430170	54.54545454545450	0.686208445642408	0.875	0.396226415094340	60.37735849056600
26		70.6703910614525	56.79012345679010	0.682350718065004	0.851851851851852	0.425925925925926	79.3296089385475	54.32098765432100	0.684785734828875	0.916666666666667	0.385964912280702	61.40350877192980
27		72.3463687150838	60.55776892430280	0.698792705666698	0.826086956521739	0.477987421383648	72.62569832402240	42.35294117647060	0.629689608636977	0.9	0.276923076923077	72.3076923076923
28		76.25698324022350	63.20346320346320	0.724102564102564	0.901234567901235	0.486666666666667	75.97765363128490	45.56962025316460	0.644209039548023	0.9	0.305084745762712	69.49152542372880
29		72.90502793296090	58.36909871244640	0.688065391574050	0.781609195402299	0.465753424657534	77.09497206703910	46.75324675324680	0.646666666666667	0.782608695652174	0.333333333333333	66.66666666666670
30		77.09497206703910	65.83333333333330	0.737434814601529	0.887640449438202	0.523178807947020	79.88826815642460	52.63157894736840	0.676559660811629	0.833333333333333	0.384615384615385	61.53846153846150
31		77.37430167597770	66.66666666666670	0.740315315315315	0.852631578947368	0.547297297297297	79.3296089385475	53.16455696202530	0.678444444444444	0.84	0.388888888888889	61.11111111111110
32		76.53631284916200	67.6923076923077	0.744696969696970	0.88	0.55	79.3296089385475	54.32098765432100	0.684233449477352	0.88	0.392857142857143	60.71428571428570
33		75.41899441340780	63.02521008403360	0.718185029382486	0.842696629213483	0.503355704697987	76.53631284916200	46.15384615384620	0.644454123112660	0.818181818181818	0.321428571428571	67.85714285714290
34		75.97765363128490	61.94690265486730	0.716141121736883	0.875	0.479452054794521	82.68156424581010	55.07246376811590	0.690283078880407	0.904761904761905	0.395833333333333	60.41666666666670
35		73.18435754189940	62.7906976744186	0.716336959588676	0.890109890109890	0.485029940119760	75.41899441340780	43.58974358974360	0.637464985994398	0.944444444444444	0.283333333333333	71.66666666666670
36		72.62569832402240	58.11965811965810	0.685706901008013	0.772727272727273	0.465753424657534	75.41899441340780	40.54054054054050	0.621733449477352	0.833333333333333	0.267857142857143	73.21428571428570
37		73.18435754189940	56.36363636363640	0.687435897435898	0.885714285714286	0.413333333333333	75.97765363128490	39.43661971830990	0.616202455825097	0.777777777777778	0.264150943396226	73.58490566037740
38		77.93296089385480	70.63197026022300	0.759653583346576	0.833333333333333	0.612903225806452	78.2122905027933	55.17241379310340	0.685830429732869	0.774193548387097	0.428571428571429	57.14285714285710
39		77.6536312849162	66.66666666666670	0.743161531816873	0.898876404494382	0.529801324503311	78.77094972067040	53.65853658536590	0.680687374173138	0.88	0.385964912280702	61.40350877192980
40		74.30167597765360	63.2	0.716766486041132	0.849462365591398	0.503184713375796	80.44692737430170	56.79012345679010	0.696962962962963	0.851851851851852	0.425925925925926	57.40740740740740
41		73.18435754189940	60.0	0.697347794094123	0.808988764044944	0.476821192052980	77.09497206703910	49.38271604938270	0.661158192090396	0.909090909090909	0.338983050847458	66.10169491525420
42		75.97765363128490	64.46280991735540	0.724104361370716	0.795918367346939	0.541666666666667	79.88826815642460	51.35135135135140	0.671824629271438	0.703703703703704	0.404255319148936	59.57446808510640
43		73.46368715083800	63.32046332046330	0.723723301122682	0.931818181818182	0.479532163742690	79.88826815642460	56.09756097560980	0.694915254237288	1.0	0.389830508474576	61.01694915254240
44		74.30167597765360	64.34108527131780	0.721496212121212	0.846938775510204	0.51875	78.77094972067040	52.50000000000000	0.674364021804967	0.75	0.403846153846154	59.61538461538460
45		76.81564245810060	67.19367588932810	0.743987341772152	0.894736842105263	0.537974683544304	81.56424581005590	59.25925925925930	0.710222222222222	0.888888888888889	0.444444444444444	55.55555555555560
46		74.02234636871510	64.63878326996200	0.723063867236611	0.858585858585859	0.518292682926829	78.2122905027933	58.06451612903230	0.70012503473187	0.84375	0.442622950819672	55.73770491803280
47		70.6703910614525	56.06694560669460	0.675139629347550	0.807228915662651	0.429487179487179	74.86033519553070	48.27586206896550	0.652260821615660	0.84	0.338709677419355	66.12903225806450
48		78.49162011173190	68.3127572016461	0.750995628946090	0.846938775510204	0.572413793103448	79.88826815642460	50.0	0.664442700156986	0.782608695652174	0.367346938775510	63.26530612244900
49		74.30167597765360	62.29508196721310	0.712439521263051	0.844444444444444	0.493506493506494	79.88826815642460	53.84615384615390	0.682844574780059	0.913043478260870	0.381818181818182	61.81818181818180
50		74.86033519553070	61.53846153846150	0.708839493409150	0.818181818181818	0.493150684931507	79.3296089385475	55.42168674698800	0.688962962962963	0.793103448275862	0.425925925925926	57.40740740740740
51		75.13966480446930	64.82213438735180	0.724567099567100	0.828282828282828	0.532467532467532	77.09497206703910	45.33333333333330	0.639322916666667	0.708333333333333	0.333333333333333	66.66666666666670
52		72.06703910614520	60.31746031746030	0.697953778730649	0.835164835164835	0.472049689440994	78.77094972067040	50.00000000000000	0.665577816492451	0.95	0.339285714285714	66.07142857142860
53		79.3296089385475	68.64406779661020	0.756170845179633	0.9	0.554794520547945	81.56424581005590	49.23076923076920	0.662637008381689	0.888888888888889	0.340425531914894	65.95744680851060
54		75.41899441340780	64.22764227642280	0.724023593177108	0.849462365591398	0.516339869281046	77.09497206703910	48.10126582278480	0.652565982404692	0.791666666666667	0.345454545454545	65.45454545454550
55		74.58100558659220	59.55555555555560	0.701824805751685	0.858974358974359	0.455782312925170	77.09497206703910	40.57971014492750	0.622804360993337	0.823529411764706	0.269230769230769	73.07692307692310
56		72.62569832402240	59.16666666666670	0.698144812110869	0.876543209876543	0.446540880503145	77.6536312849162	45.94594594594600	0.645407407407407	0.85	0.314814814814815	68.51851851851850
57		72.90502793296090	61.0441767068273	0.700826314953123	0.808510638297872	0.490322580645161	75.97765363128490	53.76344086021510	0.675257015837733	0.78125	0.409836065573771	59.01639344262300
58		74.30167597765360	63.20000000000000	0.719097222222222	0.877777777777778	0.49375	77.09497206703910	49.38271604938270	0.660017098888572	0.869565217391304	0.344827586206897	65.51724137931040
59		76.53631284916200	65.2892561983471	0.731607848174433	0.849462365591398	0.530201342281879	79.3296089385475	54.32098765432100	0.683703703703704	0.814814814814815	0.407407407407407	59.25925925925930
60		76.81564245810060	64.37768240343350	0.729035690105426	0.872093023255814	0.510204081632653	79.3296089385475	54.32098765432100	0.684233449477352	0.88	0.392857142857143	60.71428571428570
61		74.58100558659220	61.27659574468090	0.712553631018592	0.9	0.464516129032258	79.3296089385475	55.42168674698800	0.690011399259048	0.92	0.396551724137931	60.3448275862069
62		74.58100558659220	63.74501992031870	0.719281543538969	0.842105263157895	0.512820512820513	76.53631284916200	48.78048780487810	0.654181184668990	0.769230769230769	0.357142857142857	64.28571428571430
63		73.74301675977650	63.28125	0.719547809703250	0.890109890109890	0.490909090909091	77.6536312849162	57.44680851063830	0.697894021739131	0.9	0.421875	57.8125
64		74.30167597765360	63.4920634920635	0.718909010461111	0.860215053763441	0.503144654088050	78.77094972067040	58.69565217391300	0.706589673913044	0.964285714285714	0.421875	57.8125
65		73.18435754189940	62.50000000000000	0.705818691112809	0.784313725490196	0.519480519480519	75.97765363128490	44.15584415584420	0.634384164222874	0.772727272727273	0.309090909090909	69.0909090909091
66		75.69832402234640	66.92015209125480	0.737758930541981	0.862745098039216	0.546583850931677	75.97765363128490	58.25242718446600	0.698065712771595	0.857142857142857	0.441176470588235	55.88235294117650
67		74.58100558659220	62.24066390041490	0.710078344133862	0.797872340425532	0.510204081632653	77.6536312849162	51.21951219512200	0.666444444444444	0.75	0.388888888888889	61.11111111111110
68		71.22905027932960	58.96414342629480	0.688320707070707	0.813186813186813	0.4625	73.18435754189940	45.45454545454550	0.644789445628998	0.952380952380952	0.298507462686567	70.1492537313433
69		74.30167597765360	63.77952755905510	0.721095942239178	0.870967741935484	0.503105590062112	77.09497206703910	54.94505494505500	0.684518886131789	0.862068965517241	0.403225806451613	59.67741935483870
70		72.62569832402240	58.11965811965810	0.690514905149052	0.839506172839506	0.444444444444444	78.77094972067040	48.64864864864860	0.658666666666667	0.9	0.333333333333333	66.66666666666670
71		75.97765363128490	64.46280991735540	0.729445415541077	0.896551724137931	0.503225806451613	81.56424581005590	56.00000000000000	0.694049061175045	0.913043478260870	0.403846153846154	59.61538461538460
72		72.62569832402240	59.83606557377050	0.692334864005652	0.768421052631579	0.489932885906040	76.53631284916200	46.15384615384620	0.644454123112660	0.818181818181818	0.321428571428571	67.85714285714290
73		76.81564245810060	65.56016597510370	0.734487179487180	0.868131868131868	0.526666666666667	77.6536312849162	48.71794871794870	0.656598240469208	0.826086956521739	0.345454545454545	65.45454545454550
74		77.09497206703910	65.25423728813560	0.731248142154110	0.777777777777778	0.562043795620438	79.88826815642460	51.35135135135140	0.670800627943485	0.76	0.387755102040816	61.22448979591840
75		74.58100558659220	62.24066390041490	0.711538461538462	0.824175824175824	0.5	77.6536312849162	44.44444444444440	0.636744186046512	0.727272727272727	0.32	68.0
76		74.30167597765360	64.0625	0.722474174855127	0.872340425531915	0.506172839506173	77.09497206703910	56.84210526315790	0.694534412955466	0.9	0.415384615384615	58.46153846153850
77		73.74301675977650	61.78861788617890	0.709699908102798	0.853932584269663	0.484076433121019	77.6536312849162	51.21951219512200	0.668637788543745	0.875	0.362068965517241	63.79310344827590
78		78.49162011173190	68.57142857142860	0.754997608799617	0.913043478260870	0.549019607843137	81.56424581005590	60.24096385542170	0.715199884958297	0.961538461538462	0.438596491228070	56.14035087719300
79		73.74301675977650	60.16949152542370	0.702757851107923	0.855421686746988	0.464052287581699	80.44692737430170	54.54545454545460	0.686876832844575	0.954545454545455	0.381818181818182	61.81818181818180
80		73.74301675977650	61.78861788617890	0.71135551973705	0.873563218390805	0.477987421383648	79.88826815642460	52.63157894736840	0.676559660811629	0.833333333333333	0.384615384615385	61.53846153846150
81		72.90502793296090	56.50224215246640	0.678673499556519	0.75	0.453237410071942	75.97765363128490	39.43661971830990	0.612087912087912	0.636363636363636	0.285714285714286	71.42857142857140
82		72.62569832402240	63.70370370370370	0.709309649785840	0.796296296296296	0.530864197530864	76.53631284916200	55.31914893617020	0.683053221288515	0.764705882352941	0.433333333333333	56.66666666666670
83		73.74301675977650	60.83333333333330	0.703723811957077	0.829545454545455	0.480263157894737	75.97765363128490	45.56962025316460	0.640389082462253	0.782608695652174	0.321428571428571	67.85714285714290
84		72.3463687150838	59.91902834008100	0.698421717171717	0.850574712643678	0.4625	74.30167597765360	52.08333333333330	0.667270045588630	0.833333333333333	0.378787878787879	62.12121212121210
85		76.53631284916200	62.16216216216220	0.718555295950156	0.884615384615385	0.479166666666667	84.35754189944130	56.25	0.696268656716418	0.947368421052632	0.4	60.0
86		72.3463687150838	57.5107296137339	0.686414154317834	0.827160493827161	0.440789473684211	77.09497206703910	50.60240963855420	0.663718723037101	0.807692307692308	0.368421052631579	63.1578947368421
87		72.90502793296090	60.72874493927130	0.700780654988576	0.824175824175824	0.480769230769231	75.41899441340780	45.00000000000000	0.638643488173269	0.818181818181818	0.310344827586207	68.96551724137930
88		72.90502793296090	56.8888888888889	0.686891025641026	0.853333333333333	0.426666666666667	79.88826815642460	50.00000000000000	0.665843066786463	0.947368421052632	0.339622641509434	66.0377358490566
89		74.02234636871510	60.75949367088610	0.703942307692308	0.827586206896552	0.48	75.41899441340780	43.58974358974360	0.632729364394593	0.809523809523810	0.298245614035088	70.17543859649120
90		72.62569832402240	61.71875	0.704732477850995	0.831578947368421	0.490683229813665	78.2122905027933	56.17977528089890	0.691031073446328	0.833333333333333	0.423728813559322	57.6271186440678
91		74.30167597765360	62.29508196721310	0.712439521263051	0.844444444444444	0.493506493506494	80.44692737430170	53.33333333333330	0.680453431372549	0.833333333333333	0.392156862745098	60.78431372549020
92		74.86033519553070	62.18487394957980	0.715000797066794	0.870588235294118	0.483660130718954	77.09497206703910	48.1012658227848	0.654371584699453	0.863636363636364	0.333333333333333	66.66666666666670
93		74.86033519553070	63.11475409836070	0.720347308045759	0.885057471264368	0.490445859872611	78.2122905027933	50.63291139240510	0.667241875179753	0.909090909090909	0.350877192982456	64.91228070175440
94		76.53631284916200	62.5	0.717259552042161	0.813953488372093	0.507246376811594	79.3296089385475	51.94805194805200	0.672640931372549	0.769230769230769	0.392156862745098	60.78431372549020
95		75.97765363128490	66.14173228346460	0.735822784810127	0.875	0.531645569620253	78.2122905027933	51.85185185185190	0.672770019948703	0.913043478260870	0.362068965517241	63.79310344827590
96		75.41899441340780	67.88321167883210	0.742575187969925	0.877358490566038	0.553571428571429	76.53631284916200	58.82352941176470	0.702570217276100	0.882352941176471	0.441176470588235	55.88235294117650
97		75.13966480446930	64.2570281124498	0.725664556962025	0.879120879120879	0.506329113924051	82.68156424581010	60.75949367088610	0.718222222222222	0.96	0.444444444444444	55.55555555555560
98		71.50837988826820	58.19672131147540	0.691074577979340	0.865853658536585	0.438271604938272	77.6536312849162	52.38095238095240	0.673940677966102	0.88	0.372881355932203	62.71186440677970
99		75.69832402234640	63.29113924050630	0.723146819703491	0.892857142857143	0.490196078431373	81.56424581005590	54.79452054794520	0.688370684433677	0.952380952380952	0.384615384615385	61.53846153846150
	**Mean**	**74.75418994413410**	**62.7989473508903**	**0.716673057228117**	**0.852813984185844**	**0.498275690086451**	**78.4692737430168**	**51.5501936387342**	**0.671215100738811**	**0.8513198651512**	**0.371932185050901**	**62.8067814949099**
	**Standard Deviation**	1.97427257922838	3.2626726824962	0.019678714793475	0.037984181621175	0.037679379791179	2.34803415920626	5.37578559697148	0.025869878392181	0.069958604485800	0.050240525992289	5.02405259922896
	**T Test Significance (Female to Male)**						**0.000000000000835**	**0.000000000000000**	**0.000000000000134**	**0.366256016052956**	**0.000000000000000**	**0.000000000000000**

	B_F_TNR	B_F_FPR	B_F_TPR	B_M_Accuracy	B_M_FScore	B_M_ROC_AUC	B_M_Precision	B_M_Recall	B_M_FNR	B_M_TNR	B_M_FPR	B_M_TPR
0	98.31932773109240	1.680672268907560	43.33333333333330	71.50837988826820	68.32298136645960	0.731229528848576	0.873015873015873	0.561224489795918	43.87755102040820	90.12345679012350	9.876543209876540	56.12244897959180
1	96.09375	3.90625	33.33333333333330	69.83240223463690	67.07317073170730	0.704317897371715	0.785714285714286	0.585106382978723	41.48936170212770	82.35294117647060	17.64705882352940	58.51063829787230
2	100.0	0.0	45.45454545454550	73.74301675977650	70.44025157232710	0.754850088183422	0.918032786885246	0.571428571428571	42.85714285714290	93.82716049382720	6.172839506172840	57.14285714285710
3	98.36065573770490	1.639344262295080	33.33333333333330	71.50837988826820	70.17543859649120	0.735676088617265	0.869565217391304	0.588235294117647	41.17647058823530	88.31168831168830	11.68831168831170	58.82352941176470
4	96.58119658119660	3.418803418803420	37.09677419354840	69.27374301675980	67.83625730994150	0.703325774754346	0.794520547945205	0.591836734693878	40.81632653061220	81.48148148148150	18.51851851851850	59.18367346938780
5	96.875	3.125	33.33333333333330	70.94972067039110	68.29268292682930	0.723985890652557	0.848484848484849	0.571428571428571	42.85714285714290	87.65432098765430	12.34567901234570	57.14285714285710
6	98.4	1.6	46.2962962962963	69.27374301675980	66.25766871165640	0.710227272727273	0.84375	0.545454545454545	45.45454545454550	87.5	12.5	54.54545454545450
7	96.63865546218490	3.361344537815130	41.66666666666670	72.62569832402240	66.20689655172410	0.719929982495624	0.813559322033898	0.558139534883721	44.18604651162790	88.17204301075270	11.82795698924730	55.81395348837210
8	96.15384615384620	3.846153846153850	36.73469387755100	65.92178770949720	63.90532544378700	0.709222661396575	0.915254237288136	0.490909090909091	50.90909090909090	92.7536231884058	7.246376811594200	49.09090909090910
9	96.82539682539680	3.174603174603170	33.9622641509434	69.83240223463690	66.25	0.720696202531646	0.883333333333333	0.53	47.0	91.13924050632910	8.860759493670890	53.0
10	96.82539682539680	3.174603174603170	32.07547169811320	70.94972067039110	65.78947368421050	0.708676654182272	0.793650793650794	0.561797752808989	43.82022471910110	85.55555555555560	14.44444444444440	56.17977528089890
11	97.01492537313430	2.985074626865670	37.77777777777780	73.18435754189940	71.76470588235290	0.743323255228017	0.847222222222222	0.622448979591837	37.75510204081630	86.41975308641980	13.58024691358020	62.24489795918370
12	98.3739837398374	1.626016260162600	39.28571428571430	73.74301675977650	70.44025157232700	0.738077403245943	0.811594202898551	0.622222222222222	37.77777777777780	85.39325842696630	14.60674157303370	62.22222222222220
13	98.33333333333330	1.666666666666670	27.11864406779660	73.74301675977650	72.83236994219650	0.759772611139499	0.9	0.611650485436893	38.83495145631070	90.78947368421050	9.210526315789470	61.16504854368930
14	97.63779527559060	2.362204724409450	32.69230769230770	66.4804469273743	58.90410958904110	0.688523557571177	0.895833333333333	0.438775510204082	56.12244897959180	93.82716049382720	6.172839506172840	43.87755102040820
15	99.18032786885250	0.819672131147541	43.85964912280700	72.06703910614520	64.7887323943662	0.724338161838162	0.901960784313726	0.505494505494506	49.45054945054950	94.31818181818180	5.681818181818180	50.54945054945060
16	95.48872180451130	4.511278195488720	41.30434782608700	72.62569832402240	69.56521739130430	0.739879306009555	0.875	0.577319587628866	42.2680412371134	90.2439024390244	9.75609756097561	57.73195876288660
17	95.9349593495935	4.065040650406500	39.28571428571430	63.68715083798880	63.27683615819210	0.678722840487546	0.848484848484849	0.504504504504505	49.54954954954950	85.29411764705880	14.70588235294120	50.45045045045050
18	95.16129032258070	4.838709677419360	36.36363636363640	75.97765363128490	75.14450867052020	0.779666412019353	0.915492957746479	0.637254901960784	36.27450980392160	92.20779220779220	7.792207792207790	63.72549019607840
19	97.01492537313430	2.985074626865670	44.44444444444440	72.62569832402240	69.18238993710690	0.749683544303798	0.932203389830508	0.55	45.0	94.9367088607595	5.063291139240510	55.00000000000000
20	97.5609756097561	2.439024390243900	39.28571428571430	66.4804469273743	63.41463414634150	0.684050632911392	0.8125	0.52	48.0	84.81012658227850	15.18987341772150	52.0
21	97.45762711864410	2.542372881355930	34.42622950819670	70.94972067039110	67.08860759493670	0.733354430379747	0.913793103448276	0.53	47.0	93.67088607594940	6.329113924050630	53.0
22	95.7983193277311	4.201680672268910	43.33333333333330	79.88826815642460	78.31325301204820	0.808421184738956	0.928571428571429	0.677083333333333	32.29166666666670	93.97590361445780	6.024096385542170	67.70833333333330
23	96.96969696969700	3.030303030303030	48.93617021276600	70.39106145251400	71.03825136612020	0.730503390714658	0.866666666666667	0.601851851851852	39.81481481481480	85.91549295774650	14.08450704225350	60.18518518518520
24	96.06299212598430	3.937007874015750	30.76923076923080	73.18435754189940	71.08433734939760	0.741026606425703	0.842857142857143	0.614583333333333	38.54166666666670	86.74698795180720	13.25301204819280	61.45833333333330
25	97.61904761904760	2.380952380952380	39.62264150943400	73.74301675977650	70.44025157232710	0.742935733933483	0.848484848484849	0.602150537634409	39.78494623655910	88.37209302325580	11.62790697674420	60.21505376344090
26	98.36065573770490	1.639344262295080	38.59649122807020	62.01117318435750	58.0246913580247	0.656241956241956	0.824561403508772	0.447619047619048	55.23809523809520	86.48648648648650	13.51351351351350	44.76190476190480
27	98.24561403508770	1.754385964912280	27.69230769230770	72.06703910614520	69.87951807228920	0.726157697121402	0.805555555555556	0.617021276595745	38.29787234042550	83.52941176470590	16.47058823529410	61.70212765957450
28	98.33333333333330	1.666666666666670	30.50847457627120	76.53631284916200	72.36842105263160	0.768106893106893	0.901639344262295	0.604395604395604	39.56043956043960	93.18181818181820	6.818181818181820	60.43956043956040
29	96.0	4.0	33.33333333333330	68.71508379888270	64.1025641025641	0.69127936031984	0.78125	0.543478260869565	45.65217391304350	83.9080459770115	16.09195402298850	54.34782608695650
30	96.8503937007874	3.149606299212600	38.46153846153850	74.30167597765360	71.95121951219510	0.760479797979798	0.907692307692308	0.595959595959596	40.4040404040404	92.5	7.5	59.59595959595960
31	96.8	3.2	38.88888888888890	75.41899441340780	73.17073170731710	0.760325406758448	0.857142857142857	0.638297872340426	36.17021276595750	88.23529411764710	11.76470588235290	63.82978723404260
32	97.5609756097561	2.439024390243900	39.28571428571430	73.74301675977650	73.74301675977660	0.757307692307692	0.88	0.634615384615385	36.53846153846150	88.0	12.0	63.46153846153850
33	96.7479674796748	3.252032520325200	32.14285714285710	74.30167597765360	71.25000000000000	0.748312078019505	0.850746268656716	0.612903225806452	38.70967741935480	88.37209302325580	11.62790697674420	61.29032258064520
34	98.47328244274810	1.526717557251910	39.58333333333330	69.27374301675980	64.96815286624200	0.710821365583270	0.864406779661017	0.520408163265306	47.95918367346940	90.12345679012350	9.876543209876540	52.04081632653060
35	99.15966386554620	0.840336134453782	28.33333333333330	70.94972067039110	71.11111111111110	0.736565420560748	0.876712328767123	0.598130841121495	40.18691588785050	87.5	12.5	59.81308411214950
36	97.5609756097561	2.439024390243900	26.78571428571430	69.83240223463690	66.25	0.698938826466916	0.757142857142857	0.588888888888889	41.11111111111110	80.89887640449440	19.10112359550560	58.88888888888890
37	96.82539682539680	3.174603174603170	26.41509433962260	70.39106145251400	64.42953020134230	0.723032436509932	0.923076923076923	0.494845360824742	50.51546391752580	95.1219512195122	4.878048780487810	49.48453608247420
38	94.3089430894309	5.691056910569110	42.85714285714290	77.6536312849162	78.02197802197800	0.783585858585859	0.855421686746988	0.717171717171717	28.28282828282830	85.0	15.0	71.71717171717170
39	97.54098360655740	2.459016393442620	38.59649122807020	76.53631284916200	73.41772151898740	0.773216520650814	0.90625	0.617021276595745	38.29787234042550	92.94117647058820	7.058823529411760	61.70212765957450
40	96.8	3.2	42.59259259259260	68.15642458100560	66.27218934911240	0.706055186509964	0.848484848484849	0.543689320388350	45.63106796116510	86.8421052631579	13.15789473684210	54.36893203883500
41	98.33333333333330	1.666666666666670	33.89830508474580	69.27374301675980	65.40880503144650	0.696401799100450	0.776119402985075	0.565217391304348	43.47826086956520	82.75862068965520	17.24137931034480	56.52173913043480
42	93.93939393939390	6.060606060606060	40.42553191489360	72.06703910614520	70.23809523809520	0.730952979632889	0.830985915492958	0.608247422680412	39.17525773195880	85.36585365853660	14.63414634146340	60.82474226804120
43	100.0	0.0	38.98305084745760	67.0391061452514	66.66666666666670	0.718616737739872	0.907692307692308	0.526785714285714	47.32142857142860	91.04477611940300	8.955223880597010	52.67857142857140
44	94.48818897637800	5.511811023622050	40.38461538461540	69.83240223463690	69.66292134831460	0.730699008868023	0.885714285714286	0.574074074074074	42.59259259259260	88.73239436619720	11.26760563380280	57.40740740740740
45	97.6	2.4	44.44444444444440	72.06703910614520	70.93023255813950	0.746602564102564	0.897058823529412	0.586538461538462	41.34615384615390	90.66666666666670	9.333333333333330	58.65384615384620
46	95.76271186440680	4.23728813559322	44.26229508196720	69.83240223463690	68.23529411764700	0.722342871742463	0.865671641791045	0.563106796116505	43.68932038834950	88.1578947368421	11.84210526315790	56.31067961165050
47	96.58119658119660	3.418803418803420	33.87096774193550	66.4804469273743	60.52631578947370	0.674092615769712	0.793103448275862	0.489361702127660	51.06382978723400	85.88235294117650	14.11764705882350	48.93617021276600
48	96.15384615384620	3.846153846153850	36.73469387755100	77.09497206703910	76.0233918128655	0.778300702811245	0.866666666666667	0.677083333333333	32.29166666666670	87.95180722891570	12.04819277108430	67.70833333333330
49	98.38709677419360	1.612903225806450	38.18181818181820	68.71508379888270	66.26506024096390	0.702777777777778	0.820895522388060	0.555555555555556	44.44444444444440	85.0	15.0	55.55555555555560
50	95.20000000000000	4.8	42.59259259259260	70.39106145251400	64.90066225165560	0.708833083458271	0.830508474576271	0.532608695652174	46.73913043478260	88.50574712643680	11.49425287356320	53.2608695652174
51	94.53125	5.46875	33.33333333333330	73.18435754189940	73.03370786516850	0.749744506898314	0.866666666666667	0.631067961165049	36.89320388349520	86.8421052631579	13.15789473684210	63.10679611650490
52	99.1869918699187	0.813008130081301	33.92857142857140	65.36312849162010	64.77272727272730	0.676833976833977	0.802816901408451	0.542857142857143	45.71428571428570	81.08108108108110	18.91891891891890	54.28571428571430
53	98.48484848484850	1.515151515151520	34.04255319148940	77.09497206703910	76.0233918128655	0.784532828282828	0.902777777777778	0.656565656565657	34.34343434343430	91.25	8.75	65.65656565656570
54	95.96774193548390	4.032258064516130	34.54545454545460	73.74301675977650	71.8562874251497	0.750566893424036	0.869565217391304	0.612244897959184	38.77551020408160	88.88888888888890	11.11111111111110	61.22448979591840
55	97.63779527559060	2.362204724409450	26.92307692307690	72.06703910614520	67.94871794871800	0.731328320802005	0.868852459016393	0.557894736842105	44.21052631578950	90.47619047619050	9.523809523809520	55.78947368421050
56	97.6	2.4	31.48148148148150	67.59776536312850	65.0602409638554	0.709845559845560	0.885245901639344	0.514285714285714	48.57142857142860	90.54054054054050	9.45945945945946	51.42857142857140
57	94.0677966101695	5.932203389830510	40.98360655737710	69.83240223463690	65.38461538461540	0.706570713391740	0.822580645161290	0.542553191489362	45.74468085106380	87.05882352941180	12.94117647058820	54.25531914893620
58	97.52066115702480	2.479338842975210	34.48275862068970	71.50837988826820	69.8224852071006	0.737267634326458	0.880597014925373	0.578431372549020	42.15686274509800	89.6103896103896	10.38961038961040	57.84313725490200
59	96.0	4.0	40.74074074074070	73.74301675977650	70.80745341614910	0.746428571428572	0.863636363636364	0.6	40.0	89.28571428571430	10.71428571428570	60.0
60	97.5609756097561	2.439024390243900	39.28571428571430	74.30167597765360	69.73684210526320	0.745754245754246	0.868852459016393	0.582417582417583	41.75824175824180	90.9090909090909	9.090909090909090	58.24175824175830
61	98.34710743801650	1.652892561983470	39.6551724137931	69.83240223463690	64.47368421052630	0.715991953733970	0.890909090909091	0.505154639175258	49.48453608247420	92.6829268292683	7.317073170731710	50.51546391752580
62	95.1219512195122	4.878048780487810	35.71428571428570	72.62569832402240	71.00591715976330	0.743037974683544	0.869565217391304	0.6	40.0	88.60759493670890	11.39240506329110	60.0
63	97.3913043478261	2.608695652173910	42.1875	69.83240223463690	66.66666666666670	0.722454937801472	0.885245901639344	0.534653465346535	46.53465346534650	91.02564102564100	8.974358974358970	53.46534653465350
64	99.1304347826087	0.869565217391304	42.1875	69.83240223463690	66.25	0.707518796992481	0.815384615384615	0.557894736842105	44.21052631578950	85.71428571428570	14.28571428571430	55.78947368421050
65	95.96774193548390	4.032258064516130	30.90909090909090	70.39106145251400	70.39106145251400	0.711931818181818	0.7875	0.636363636363636	36.36363636363640	78.75	21.25	63.63636363636360
66	95.4954954954955	4.504504504504510	44.11764705882350	75.41899441340780	72.5	0.759502375593898	0.865671641791045	0.623655913978495	37.63440860215050	89.53488372093020	10.46511627906980	62.36559139784950
67	94.40000000000000	5.600000000000000	38.88888888888890	71.50837988826820	67.9245283018868	0.720555138784696	0.818181818181818	0.580645161290323	41.93548387096770	86.04651162790700	13.95348837209300	58.06451612903230
68	99.10714285714290	0.892857142857143	29.85074626865670	69.27374301675980	66.25766871165640	0.697299324831208	0.771428571428572	0.580645161290323	41.93548387096770	81.3953488372093	18.6046511627907	58.06451612903230
69	96.58119658119660	3.418803418803420	40.32258064516130	71.50837988826820	68.71165644171780	0.732828282828283	0.875	0.565656565656566	43.43434343434340	90.0	10.0	56.56565656565660
70	98.4	1.6	33.33333333333330	66.4804469273743	62.5	0.683775252525253	0.819672131147541	0.505050505050505	49.49494949494950	86.25	13.75000000000000	50.50505050505050
71	98.4251968503937	1.574803149606300	40.38461538461540	70.39106145251400	68.26347305389220	0.730646397547266	0.890625	0.553398058252427	44.66019417475730	90.78947368421050	9.210526315789470	55.33980582524270
72	96.7479674796748	3.252032520325200	32.14285714285710	68.71508379888270	66.26506024096390	0.691047761940485	0.753424657534247	0.591397849462366	40.86021505376340	79.06976744186050	20.93023255813950	59.13978494623660
73	96.7741935483871	3.225806451612900	34.54545454545460	75.97765363128490	73.61963190184050	0.768170426065163	0.882352941176471	0.631578947368421	36.84210526315790	90.47619047619050	9.523809523809520	63.1578947368421
74	95.38461538461540	4.615384615384620	38.77551020408160	74.30167597765360	71.60493827160490	0.741633366633367	0.783783783783784	0.659090909090909	34.09090909090910	82.41758241758240	17.58241758241760	65.9090909090909
75	95.34883720930230	4.651162790697680	32.0	71.50837988826820	69.82248520710060	0.731708860759494	0.855072463768116	0.59	41.0	87.34177215189870	12.65822784810130	59.0
76	97.36842105263160	2.631578947368420	41.53846153846150	71.50837988826820	68.32298136645960	0.728627105858688	0.859375	0.567010309278351	43.29896907216500	89.02439024390250	10.97560975609760	56.70103092783510
77	97.52066115702480	2.479338842975210	36.20689655172410	69.83240223463690	67.07317073170730	0.715277777777778	0.846153846153846	0.555555555555556	44.44444444444440	87.5	12.5	55.55555555555560
78	99.18032786885250	0.819672131147541	43.85964912280700	75.41899441340780	72.83950617283950	0.765122991967872	0.893939393939394	0.614583333333333	38.54166666666670	91.56626506024100	8.433734939759040	61.45833333333330
79	99.19354838709680	0.806451612903226	38.18181818181820	67.0391061452514	62.8930817610063	0.687200806248425	0.819672131147541	0.510204081632653	48.97959183673470	86.41975308641980	13.58024691358020	51.02040816326530
80	96.8503937007874	3.149606299212600	38.46153846153850	67.59776536312850	65.88235294117650	0.713071131879543	0.888888888888889	0.523364485981308	47.66355140186920	90.27777777777780	9.722222222222220	52.33644859813080
81	93.84615384615380	6.153846153846150	28.57142857142860	69.83240223463690	64.4736842105263	0.699188514357054	0.790322580645161	0.544444444444444	45.55555555555560	85.39325842696630	14.60674157303370	54.44444444444440
82	93.27731092436970	6.722689075630250	43.33333333333330	68.71508379888270	68.18181818181820	0.703208556149733	0.810810810810811	0.588235294117647	41.17647058823530	81.81818181818180	18.18181818181820	58.82352941176470
83	95.9349593495935	4.065040650406500	32.14285714285710	71.50837988826820	68.32298136645960	0.726217369477912	0.846153846153846	0.572916666666667	42.70833333333330	87.95180722891570	12.04819277108430	57.29166666666670
84	95.57522123893810	4.424778761061950	37.87878787878790	70.39106145251400	64.90066225165560	0.713579474342929	0.859649122807018	0.521276595744681	47.87234042553190	90.58823529411770	9.411764705882350	52.12765957446810
85	99.25373134328360	0.746268656716418	40.0	68.71508379888270	64.55696202531650	0.707575757575758	0.864406779661017	0.515151515151515	48.48484848484850	90.0	10.0	51.51515151515150
86	95.90163934426230	4.098360655737710	36.84210526315790	67.59776536312850	61.33333333333330	0.688533834586466	0.836363636363636	0.484210526315790	51.57894736842110	89.28571428571430	10.71428571428570	48.42105263157890
87	96.69421487603310	3.305785123966940	31.03448275862070	70.39106145251400	68.26347305389220	0.716742252456538	0.826086956521739	0.581632653061225	41.83673469387760	85.18518518518520	14.81481481481480	58.16326530612250
88	99.20634920634920	0.793650793650794	33.9622641509434	65.92178770949720	60.13071895424840	0.676137792305758	0.821428571428571	0.474226804123711	52.57731958762890	87.8048780487805	12.19512195121950	47.42268041237110
89	96.72131147540980	3.278688524590160	29.82456140350880	72.62569832402240	69.18238993710690	0.731745436359090	0.833333333333333	0.591397849462366	40.86021505376340	87.20930232558140	12.79069767441860	59.13978494623660
90	95.83333333333330	4.166666666666670	42.3728813559322	67.0391061452514	64.67065868263470	0.693277310924370	0.830769230769231	0.529411764705882	47.05882352941180	85.71428571428570	14.28571428571430	52.94117647058820
91	96.875	3.125	39.21568627450980	68.15642458100560	66.27218934911240	0.706055186509964	0.848484848484849	0.543689320388350	45.63106796116510	86.8421052631579	13.15789473684210	54.36893203883500
92	97.54098360655740	2.459016393442620	33.33333333333330	72.62569832402240	69.18238993710690	0.738265562248996	0.873015873015873	0.572916666666667	42.70833333333330	90.36144578313250	9.63855421686747	57.29166666666670
93	98.36065573770490	1.639344262295080	35.08771929824560	71.50837988826820	69.0909090909091	0.734367088607595	0.876923076923077	0.57	43.0	89.87341772151900	10.12658227848100	57.00000000000000
94	95.3125	4.6875	39.21568627450980	73.74301675977650	68.02721088435370	0.733008495752124	0.833333333333333	0.574712643678161	42.5287356321839	89.13043478260870	10.86956521739130	57.47126436781610
95	98.34710743801650	1.652892561983470	36.20689655172410	73.74301675977650	72.83236994219650	0.751708860759494	0.863013698630137	0.63	37.0	87.34177215189870	12.65822784810130	63.0
96	96.3963963963964	3.603603603603600	44.11764705882350	74.30167597765360	73.25581395348840	0.758037974683544	0.875	0.63	37.0	88.60759493670890	11.39240506329110	63.0
97	99.2	0.8	44.44444444444440	67.59776536312850	65.88235294117650	0.702564102564103	0.848484848484849	0.538461538461538	46.15384615384620	86.66666666666670	13.33333333333330	53.84615384615390
98	97.5	2.5	37.28813559322030	65.36312849162010	61.25000000000000	0.685232498722535	0.859649122807018	0.475728155339806	52.42718446601940	89.47368421052630	10.52631578947370	47.57281553398060
99	99.21259842519690	0.787401574803150	38.46153846153850	69.83240223463690	67.07317073170730	0.720995176440721	0.873015873015873	0.544554455445545	45.54455445544560	89.74358974358980	10.25641025641030	54.45544554455450
**Mean**	**97.0498016426721**	**2.95019835732786**	**37.1932185050901**	**71.03910614525140**	**68.20244316984530**	**0.725671807288817**	**0.853677298665055**	**0.570111141696765**	**42.9888858303236**	**88.12324728808690**	**11.8767527119131**	**57.0111141696765**
**Standard Deviation**	1.4803638384749	1.48036383847489	5.02405259922896	3.22221303096379	3.95787457394657	0.028178679396597	0.040466819351135	0.050881299021780	5.0881299021781	3.48014435618900	3.480144356189	5.0881299021781
**T Test Significance (Female to Male)**	**0.000000000001057**	**0.000000000000000**	**0.000000000000000**									

Summary of Results for Experiment 3.1.3 - All Classifiers

	**Random Forest Classifier**			**Logistic Regression Classifier**			**Support Vector Machine**			**Gaussian Naive Bayes**		
	Mean	Standard Deviation	T Test Significance (Female to Male)	Mean	Standard Deviation	T Test Significance (Female to Male)	Mean	Standard Deviation	T Test Significance (Female to Male)	Mean	Standard Deviation	T Test Significance (Female to Male)
**All Accuracy**	76.88	2.68		70.34	2.31		70.24	2.36		71.56	2.60	
**All FScore**	75.65	2.94		65.88	2.86		64.00	3.24		64.57	3.62	
**All ROC_AUC**	76.92%	2.65%		70.35%	2.21%		70.38%	2.25%		71.67%	2.24%	
**All Precision**	80.32%	4.24%		77.38%	4.23%		81.75%	4.61%		86.07%	3.68%	
**All Recall**	71.72%	4.10%		57.56%	3.84%		52.77%	3.98%		51.86%	4.50%	
Females Accuracy	74.45	4.79	0.00	72.40	4.26	0.01	72.22	4.03	0.01	73.93	4.45	0.00
Females FScore	63.50	7.71	0.00	53.13	7.06	0.00	50.66	7.56	0.00	52.19	8.13	0.00
Females ROC_AUC	71.49%	5.08%	0.00	66.65%	3.92%	0.00	65.92%	3.96%	0.00	67.24%	4.06%	0.00
Females Precision	72.51%	9.58%	0.00	77.21%	8.66%	0.44	82.53%	9.80%	0.90	88.92%	7.98%	0.03
Females Recall	57.62%	10.12%	0.00	41.05%	7.62%	0.00	36.98%	7.22%	0.00	37.43%	7.92%	0.00
**Females FNR**	42.38	10.12	0.00	58.95	7.62	0.00	63.02	7.22	0.00	62.57	7.92	0.00
**Females TNR**	85.37	6.39	0.00	92.26	3.47	0.00	94.86	3.28	0.00	97.06	2.18	0.00
**Females FPR**	14.63	6.39	0.00	7.74	3.47	0.00	5.14	3.28	0.00	2.94	2.18	0.00
**Females TPR**	57.62	10.12	0.00	41.05	7.62	0.00	36.98	7.22	0.00	37.43	7.92	0.00
**Males Accuracy**	77.87	3.12		69.51	3.19		69.47	2.85		70.62	3.04	
**Males FScore**	78.87	3.14		68.92	3.34		67.16	3.36		67.48	3.98	
**Males ROC_AUC**	78.10%	3.19%		70.25%	3.15%		70.82%	2.67%		72.23%	2.73%	
**Males Precision**	82.37%	4.43%		77.45%	4.66%		81.66%	4.77%		85.51%	4.30%	
**Males Recall**	75.83%	3.85%		62.29%	4.25%		57.27%	4.47%		55.97%	5.15%	
**Males FNR**	24.17	3.85		37.71	4.25		42.73	4.47		44.03	5.15	
**Males TNR**	80.38	5.24		78.22	5.08		84.36	4.50		88.49	3.54	
**Males FPR**	19.62	5.24		21.78	5.08		15.64	4.50		11.51	3.54	
**Males TPR**	75.83	3.85		62.29	4.25		57.27	4.47		55.97	5.15	
	**Random Forest Results**			**Logistic Regression Results**			**Support Vector Machine Results**			**Gaussian Naive Bayes Results**		
**Sex Performance Disparities (Male Mean - Female Mean, n=100)**	Sex Performance Disparities (Male Mean - Female Mean, n=100)		**T Test P Value**	Sex Performance Disparities (Male Mean - Female Mean, n=100)		**T Test P Value**	Sex Performance Disparities (Male Mean - Female Mean, n=100)		**T Test P Value**	Sex Performance Disparities (Male Mean - Female Mean, n=100)		**T Test P Value**
**Accuacy**	**3.42**		0.00	**-2.90**		0.01	**-2.75**		0.01	**-3.31**		0.00
**FScore**	**15.36**		0.00	**15.79**		0.00	**16.50**		0.00	**15.29**		0.00
**ROC_AUC**	**6.61%**		0.00	**3.60%**		0.00	**4.90%**		0.00	**4.99%**		0.00
**Precision**	**9.85%**		0.00	**0.24%**		0.44	**-0.87%**		0.90	**-3.41%**		0.03
**Recall**	**18.21%**		0.00	**21.24%**		0.00	**20.30%**		0.00	**18.54%**		0.00
**False Negative Rate**	**-18.21**		0.00	**-21.24**		0.00	**-20.30**		0.00	**-18.54**		0.00
**True Negative Rate**	**-4.99**		0.00	**-14.04**		0.00	**-10.50**		0.00	**-8.57**		0.00
**False Positive Rate**	**4.99**		0.00	**14.04**		0.00	**10.50**		0.00	**8.57**		0.00
**True Positive Rate**	**18.21**		0.00	**21.24**		0.00	**20.30**		0.00	**18.54**		0.00

Experiment 3.1.3 (Unbalanced training data, with feature selection) - Random Forest Classifier

		UB_Accuracy	UB_FScore	UB_ROC_AUC	UB_Precision	UB_Recall	UB_F_Accuracy	UB_F_FScore	UB_F_ROC_AUC	UB_F_Precision	UB_F_Recall	UB_F_FNR	UB_F_TNR	UB_F_FPR	UB_F_TPR	UB_M_Accuracy	UB_M_FScore	UB_M_ROC_AUC	UB_M_Precision	UB_M_Recall	UB_M_FNR	UB_M_TNR	UB_M_FPR	UB_M_TPR
0		75.6	74.26160337552740	0.756	0.7857142857142860	0.704	75.36231884057970	71.18644067796610	0.74660441426146	0.75	0.6774193548387100	32.25806451612900	81.57894736842110	18.421052631578900	67.74193548387100	75.69060773480660	75.28089887640450	0.758681829298117	0.7976190476190480	0.7127659574468090	28.723404255319200	80.45977011494250	19.54022988505750	71.27659574468090
1		81.2	79.82832618025750	0.81329620382818	0.8773584905660380	0.7322834645669290	79.71014492753620	65.0	0.7383720930232560	0.9285714285714290	0.5	50.0	97.67441860465120	2.3255813953488400	50.0	81.76795580110500	82.90155440414510	0.821039603960396	0.8695652173913040	0.7920792079207920	20.792079207920800	85.0	15.0	79.20792079207920
2		73.6	72.5	0.7482531055900620	0.8529411764705880	0.6304347826086960	73.17073170731710	57.692307692307700	0.686456400742115	0.7894736842105260	0.4545454545454500	54.54545454545450	91.83673469387760	8.16326530612245	45.45454545454550	73.80952380952380	76.59574468085110	0.7555555555555560	0.8674698795180720	0.6857142857142860	31.428571428571400	82.53968253968250	17.46031746031750	68.57142857142860
3		74.4	73.109243697479	0.7463002114164900	0.7981651376146790	0.6744186046511630	67.64705882352940	50.0	0.6339285714285710	0.6875	0.39285714285714300	60.71428571428570	87.5	12.5	39.285714285714300	76.92307692307690	78.35051546391750	0.771299352157438	0.8172043010752690	0.7524752475247530	24.752475247524800	79.01234567901230	20.98765432098770	75.24752475247520
4		76.0	74.13793103448280	0.7594726062468000	0.7962962962962960	0.6935483870967740	80.82191780821920	76.66666666666670	0.7984848484848490	0.8518518518518520	0.696969696969697	30.303030303030300	90.0	10.0	69.6969696969697	74.01129943502830	73.25581395348840	0.7415026833631480	0.7777777777777780	0.6923076923076920	30.76923076923080	79.06976744186050	20.930232558139500	69.23076923076920
5		75.2	73.04347826086960	0.7526881720430110	0.8076923076923080	0.6666666666666670	72.15189873417720	59.25925925925930	0.6861702127659580	0.7272727272727270	0.5	50.0	87.2340425531915	12.76595744680850	50.0	76.60818713450290	77.27272727272730	0.7707930367504840	0.8292682926829270	0.723404255319149	27.659574468085100	81.81818181818180	18.181818181818200	72.3404255319149
6		80.0	79.50819672131150	0.8038649203903440	0.8660714285714290	0.7348484848484850	76.81159420289860	71.42857142857140	0.7613636363636360	0.8695652173913040	0.6060606060606060	39.39393939393940	91.66666666666670	8.333333333333330	60.60606060606060	81.21546961325970	81.91489361702130	0.8157181571815720	0.8651685393258430	0.7777777777777780	22.22222222222220	85.36585365853660	14.634146341463400	77.77777777777780
7		78.8	76.01809954751130	0.7850919341405600	0.84	0.6942148760330580	74.35897435897440	56.52173913043480	0.6833216045038710	0.7647058823529410	0.4482758620689660	55.172413793103400	91.83673469387760	8.16326530612245	44.827586206896600	80.81395348837210	81.14285714285710	0.8108695652173910	0.8554216867469880	0.7717391304347830	22.82608695652170	85.0	15.0	77.17391304347830
8		76.0	74.57627118644070	0.7592983803853790	0.7787610619469030	0.7154471544715450	80.0	68.29268292682930	0.765151515151515	0.7	0.6666666666666670	33.33333333333330	86.36363636363640	13.636363636363600	66.66666666666670	74.5945945945946	75.89743589743590	0.7482872667139150	0.7956989247311830	0.7254901960784310	27.450980392156900	77.10843373493980	22.89156626506020	72.54901960784310
9		79.2	78.33333333333330	0.7934170081967210	0.8392857142857140	0.734375	72.0	60.377358490566000	0.6899696048632220	0.64	0.5714285714285710	42.857142857142900	80.85106382978720	19.148936170212800	57.14285714285710	82.28571428571430	83.42245989304810	0.83	0.896551724137931	0.78	22.0	88.0	12.0	78.0
10		80.4	80.32128514056230	0.8084791559444160	0.8695652173913040	0.746268656716418	79.22077922077920	73.33333333333330	0.7765151515151510	0.8148148148148150	0.6666666666666670	33.33333333333330	88.63636363636360	11.363636363636400	66.66666666666670	80.92485549132950	82.53968253968250	0.8166941694169420	0.8863636363636360	0.7722772277227720	22.772277227722800	86.11111111111110	13.88888888888890	77.22772277227720
11		74.8	71.74887892376680	0.7453071945672370	0.7843137254901960	0.6611570247933880	68.42105263157900	47.826086956521700	0.6220710506424790	0.5789473684210530	0.4074074074074070	59.25925925925930	83.6734693877551	16.3265306122449	40.74074074074070	77.58620689655170	77.96610169491530	0.7795212765957450	0.8313253012048190	0.7340425531914890	26.595744680851100	82.5	17.5	73.40425531914890
12		74.4	74.60317460317460	0.7445063745275160	0.7642276422764230	0.7286821705426360	70.83333333333330	61.81818181818180	0.6888276947285600	0.7083333333333330	0.5483870967741940	45.16129032258060	82.92682926829270	17.073170731707300	54.83870967741940	75.84269662921350	78.1725888324873	0.7553571428571430	0.7777777777777780	0.7857142857142860	21.428571428571400	72.5	27.500000000000000	78.57142857142860
13		76.0	75.80645161290320	0.7636398689030270	0.8173913043478260	0.706766917293233	75.75757575757580	69.23076923076920	0.7424812030075190	0.75	0.6428571428571430	35.714285714285700	84.21052631578950	15.789473684210500	64.28571428571430	76.08695652173910	77.55102040816330	0.7669680530440030	0.8351648351648350	0.7238095238095240	27.61904761904760	81.0126582278481	18.9873417721519	72.38095238095240
14		74.0	69.19431279620850	0.7330184435447590	0.776595744680851	0.6239316239316240	70.76923076923080	48.648648648648600	0.638719512195122	0.6923076923076920	0.375	62.5	90.2439024390244	9.75609756097561	37.5	75.13513513513510	73.5632183908046	0.7516947171575500	0.7901234567901230	0.6881720430107530	31.182795698924700	81.52173913043480	18.478260869565200	68.81720430107530
15		77.60000000000000	76.27118644067800	0.7755928282244070	0.7563025210084030	0.7692307692307690	84.61538461538460	78.26086956521740	0.839323467230444	0.75	0.8181818181818180	18.181818181818200	86.04651162790700	13.953488372093000	81.81818181818180	75.13513513513510	75.78947368421050	0.7511695906432750	0.7578947368421050	0.7578947368421050	24.210526315789500	74.44444444444440	25.555555555555600	75.78947368421050
16		79.60000000000000	79.68127490039840	0.796	0.7936507936507940	0.8	84.05797101449280	74.4186046511628	0.8043478260869570	0.8	0.6956521739130440	30.434782608695700	91.30434782608700	8.695652173913040	69.56521739130430	77.90055248618790	80.76923076923080	0.772524199553239	0.7924528301886790	0.8235294117647060	17.647058823529400	72.15189873417720	27.848101265822800	82.35294117647060
17		76.8	76.42276422764230	0.7684527238973180	0.7899159663865550	0.7401574803149610	77.33333333333330	75.36231884057970	0.7745376955903270	0.8387096774193550	0.6842105263157900	31.57894736842110	86.48648648648650	13.513513513513500	68.42105263157900	76.57142857142860	76.8361581920904	0.7657434021426710	0.7727272727272730	0.7640449438202250	23.595505617977500	76.74418604651160	23.25581395348840	76.40449438202250
18		79.2	76.57657657657660	0.7884405670665210	0.8252427184466020	0.7142857142857140	73.13432835820900	57.14285714285710	0.6804761904761910	0.7058823529411770	0.48	52.0	88.09523809523810	11.904761904761900	48.0	81.4207650273224	81.11111111111110	0.815264164475257	0.8488372093023260	0.776595744680851	22.340425531914900	85.39325842696630	14.606741573033700	77.6595744680851
19		80.80000000000000	80.48780487804880	0.8081797235023040	0.825	0.7857142857142860	80.0	71.69811320754720	0.7722222222222220	0.8260869565217390	0.6333333333333330	36.666666666666700	91.11111111111110	8.88888888888889	63.33333333333330	81.14285714285710	82.90155440414510	0.8090717299578060	0.8247422680412370	0.8333333333333330	16.666666666666700	78.48101265822780	21.518987341772200	83.33333333333330
20		80.4	76.99530516431920	0.7974781266083380	0.845360824742268	0.7068965517241380	78.2051282051282	54.05405405405410	0.6810276679841900	0.7142857142857140	0.43478260869565200	56.52173913043480	92.72727272727270	7.2727272727272700	43.47826086956520	81.3953488372093	81.81818181818180	0.8174765210289910	0.8674698795180720	0.7741935483870970	22.58064516129030	86.07594936708860	13.924050632911400	77.41935483870970
21		72.8	69.36936936936940	0.7251585623678650	0.7623762376237620	0.6363636363636360	72.85714285714290	48.648648648648600	0.6550387596899220	0.9	0.3333333333333330	66.66666666666670	97.67441860465120	2.3255813953488400	33.33333333333330	72.77777777777780	73.51351351351350	0.7279811974270160	0.7472527472527470	0.723404255319149	27.659574468085100	73.25581395348840	26.744186046511600	72.3404255319149
22		79.60000000000000	77.92207792207790	0.7949875168043020	0.8333333333333330	0.7317073170731710	75.67567567567570	60.8695652173913	0.7067307692307690	0.7	0.5384615384615380	46.15384615384620	87.5	12.5	53.84615384615390	81.25	82.16216216216220	0.81580321023098	0.8636363636363640	0.7835051546391750	21.649484536082500	84.81012658227850	15.18987341772150	78.35051546391750
23		76.4	75.7201646090535	0.7700077200205870	0.8440366972477070	0.6865671641791050	73.23943661971830	65.45454545454550	0.7241653418124010	0.8571428571428570	0.5294117647058820	47.05882352941180	91.8918918918919	8.108108108108110	52.94117647058820	77.6536312849162	78.72340425531920	0.7813924050632910	0.8409090909090910	0.74	26.0	82.27848101265820	17.72151898734180	74.0
24		74.8	73.19148936170210	0.7468447690435010	0.7543859649122810	0.7107438016528930	80.0	72.34042553191490	0.7756410256410260	0.8095238095238100	0.6538461538461540	34.61538461538460	89.74358974358980	10.256410256410300	65.38461538461540	72.97297297297300	73.40425531914890	0.7298245614035090	0.7419354838709680	0.7263157894736840	27.368421052631600	73.33333333333330	26.666666666666700	72.63157894736840
25		84.8	84.16666666666670	0.8488573074707130	0.8938053097345130	0.7952755905511810	90.41095890410960	84.44444444444440	0.8695833333333330	0.95	0.76	24.0	97.91666666666670	2.083333333333330	76.0	82.48587570621470	84.1025641025641	0.828627450980392	0.8817204301075270	0.803921568627451	19.607843137254900	85.33333333333330	14.666666666666700	80.3921568627451
26		76.0	73.68421052631580	0.7576923076923080	0.7777777777777780	0.7	80.59701492537310	68.29268292682930	0.7567829457364340	0.8235294117647060	0.5833333333333330	41.66666666666670	93.02325581395350	6.976744186046510	58.333333333333300	74.31693989071040	74.8663101604278	0.7438936781609190	0.7692307692307690	0.7291666666666670	27.083333333333300	75.86206896551720	24.137931034482800	72.91666666666670
27		79.2	77.39130434782610	0.790249215196361	0.8165137614678900	0.7355371900826450	76.05633802816900	60.46511627906980	0.7070035460992910	0.6842105263157900	0.5416666666666670	45.83333333333330	87.2340425531915	12.76595744680850	54.166666666666700	80.44692737430170	81.28342245989310	0.8063867236610510	0.8444444444444440	0.7835051546391750	21.649484536082500	82.92682926829270	17.073170731707300	78.35051546391750
28		74.8	72.72727272727270	0.7469752256577690	0.7777777777777780	0.6829268292682930	73.61111111111110	62.7450980392157	0.7074074074074070	0.6666666666666670	0.5925925925925930	40.74074074074070	82.22222222222220	17.77777777777780	59.25925925925930	75.28089887640450	75.55555555555560	0.7566056910569110	0.8095238095238100	0.7083333333333330	29.166666666666700	80.48780487804880	19.51219512195120	70.83333333333330
29		79.60000000000000	79.68127490039840	0.7960189452124940	0.8	0.7936507936507940	78.33333333333330	71.1111111111111	0.771531100478469	0.6956521739130440	0.7272727272727270	27.27272727272730	81.57894736842110	18.421052631578900	72.72727272727270	80.0	81.55339805825240	0.7991949910554560	0.8235294117647060	0.8076923076923080	19.230769230769200	79.06976744186050	20.930232558139500	80.76923076923080
30		77.60000000000000	75.22123893805310	0.7752816180235540	0.8333333333333330	0.6854838709677420	75.0	57.777777777777800	0.6900000000000000	0.6842105263157900	0.5	50.0	88.0	12.0	50.0	78.73563218390800	79.55801104972380	0.7949785177228790	0.8674698795180720	0.7346938775510200	26.53061224489800	85.52631578947370	14.473684210526300	73.46938775510210
31		77.60000000000000	76.85950413223140	0.7812479917743080	0.8532110091743120	0.6992481203007520	73.23943661971830	68.85245901639350	0.7277424483306840	0.7777777777777780	0.6176470588235290	38.23529411764710	83.78378378378380	16.216216216216200	61.76470588235290	79.3296089385475	79.55801104972380	0.8011363636363640	0.8780487804878050	0.7272727272727270	27.27272727272730	87.5	12.5	72.72727272727270
32		73.6	73.6	0.7364241803278690	0.7540983606557380	0.71875	76.92307692307690	67.85714285714290	0.7459535538353270	0.7037037037037040	0.6551724137931030	34.48275862068970	83.6734693877551	16.3265306122449	65.51724137931040	72.09302325581400	75.25773195876290	0.7180019371800200	0.7684210526315790	0.7373737373737370	26.262626262626300	69.86301369863010	30.136986301369900	73.73737373737370
33		75.6	73.12775330396480	0.753319648470075	0.7685185185185190	0.6974789915966390	66.66666666666670	44.89795918367350	0.602088948787062	0.5238095238095240	0.39285714285714300	60.71428571428570	81.13207547169810	18.867924528301900	39.285714285714300	79.88165680473370	80.89887640449440	0.7994505494505490	0.8275862068965520	0.7912087912087910	20.87912087912090	80.76923076923080	19.230769230769200	79.12087912087910
34		79.2	78.33333333333330	0.7923707117255510	0.8245614035087720	0.746031746031746	72.15189873417720	64.51612903225810	0.7052287581699350	0.7142857142857140	0.5882352941176470	41.17647058823530	82.22222222222220	17.77777777777780	58.82352941176470	82.45614035087720	83.14606741573030	0.8262245459548710	0.8604651162790700	0.8043478260869570	19.565217391304300	84.81012658227850	15.18987341772150	80.43478260869570
35		80.4	80.7843137254902	0.8041834838875010	0.8174603174603170	0.7984496124031010	76.66666666666670	75.0	0.765625	0.75	0.75	25.0	78.125	21.875	75.0	81.57894736842110	82.41206030150750	0.8160529536099680	0.8367346938775510	0.8118811881188120	18.81188118811880	82.02247191011240	17.97752808988760	81.1881188118812
36		77.2	78.65168539325840	0.7771257172665630	0.84	0.7394366197183100	73.97260273972600	65.45454545454550	0.7214285714285710	0.6666666666666670	0.6428571428571430	35.714285714285700	80.0	20.0	64.28571428571430	78.53107344632770	82.0754716981132	0.7942773600668340	0.8877551020408160	0.7631578947368420	23.684210526315800	82.53968253968250	17.46031746031750	76.31578947368420
37		74.8	73.41772151898740	0.7532742681047770	0.8285714285714290	0.6590909090909090	72.36842105263160	64.40677966101700	0.7236842105263160	0.9047619047619050	0.5	50.0	94.73684210526320	5.263157894736840	50.0	75.86206896551720	76.40449438202250	0.7617021276595750	0.8095238095238100	0.723404255319149	27.659574468085100	80.0	20.0	72.3404255319149
38		78.4	75.45454545454550	0.7797252182845400	0.8137254901960780	0.7033898305084750	71.83098591549300	52.38095238095240	0.6615448504983390	0.7857142857142860	0.39285714285714300	60.71428571428570	93.02325581395350	6.976744186046510	39.285714285714300	81.00558659217880	80.89887640449440	0.8101123595505620	0.8181818181818180	0.8	20.0	82.02247191011240	17.97752808988760	80.0
39		78.0	76.39484978540770	0.7820824795081970	0.8476190476190480	0.6953125	73.13432835820900	62.500000000000000	0.7037545787545790	0.75	0.5357142857142860	46.42857142857140	87.17948717948720	12.82051282051280	53.57142857142860	79.78142076502730	80.0	0.8037349397590360	0.8705882352941180	0.74	26.0	86.74698795180720	13.253012048192800	74.0
40		76.8	76.22950819672130	0.7678662909836070	0.7622950819672130	0.7622950819672130	82.1917808219178	71.1111111111111	0.7878260869565220	0.7272727272727270	0.6956521739130440	30.434782608695700	88.0	12.0	69.56521739130430	74.57627118644070	77.38693467336680	0.7414529914529910	0.77	0.7777777777777780	22.22222222222220	70.51282051282050	29.48717948717950	77.77777777777780
41		75.2	75.2	0.7520481310803890	0.7580645161290320	0.746031746031746	75.71428571428570	71.18644067796610	0.7559523809523810	0.6774193548387100	0.75	25.0	76.19047619047620	23.809523809523800	75.0	75.0	76.43979057591620	0.7504977600796420	0.7849462365591400	0.7448979591836740	25.510204081632700	75.60975609756100	24.390243902439000	74.48979591836740
42		76.4	75.72016460905350	0.7638248847926270	0.773109243697479	0.7419354838709680	70.76923076923080	61.22448979591840	0.7013742071881610	0.5555555555555560	0.6818181818181820	31.818181818181800	72.09302325581400	27.906976744186000	68.18181818181820	78.37837837837840	79.38144329896910	0.7870895346090240	0.8369565217391310	0.7549019607843140	24.509803921568600	81.92771084337350	18.072289156626500	75.49019607843140
43		75.6	75.50200803212850	0.7569030687423920	0.7833333333333330	0.7286821705426360	70.4225352112676	64.40677966101690	0.6943108974358980	0.7037037037037040	0.59375	40.625	79.48717948717950	20.51282051282050	59.375	77.6536312849162	78.94736842105260	0.7768418405833540	0.8064516129032260	0.7731958762886600	22.68041237113400	78.04878048780490	21.951219512195100	77.31958762886600
44		74.0	74.10358565737050	0.74	0.7380952380952380	0.744	74.4186046511628	63.33333333333330	0.7223230490018150	0.6129032258064520	0.6551724137931030	34.48275862068970	78.94736842105260	21.052631578947400	65.51724137931040	73.78048780487810	77.4869109947644	0.7310049019607840	0.7789473684210530	0.7708333333333330	22.916666666666700	69.11764705882350	30.88235294117650	77.08333333333330
45		79.2	76.99115044247790	0.7906664106011140	0.8446601941747570	0.7073170731707320	77.27272727272730	66.66666666666670	0.7384615384615390	0.7894736842105260	0.5769230769230770	42.30769230769230	90.0	10.0	57.692307692307700	79.8913043478261	79.55801104972380	0.8021685033771770	0.8571428571428570	0.7422680412371130	25.773195876288700	86.20689655172410	13.793103448275900	74.22680412371140
46		74.8	72.4890829694323	0.7456343091936310	0.7477477477477480	0.7033898305084750	73.5632183908046	62.295081967213100	0.7039400665926750	0.7037037037037040	0.5588235294117650	44.11764705882350	84.90566037735850	15.09433962264150	55.88235294117650	75.4601226993865	76.19047619047620	0.7543701024713680	0.7619047619047620	0.7619047619047620	23.809523809523800	74.68354430379750	25.31645569620250	76.19047619047620
47		76.4	75.31380753138080	0.7634615384615390	0.7563025210084030	0.75	75.67567567567570	62.500000000000000	0.7280477408354650	0.6	0.6521739130434780	34.78260869565220	80.3921568627451	19.607843137254900	65.21739130434780	76.70454545454550	78.53403141361260	0.766344773587368	0.7978723404255320	0.7731958762886600	22.68041237113400	75.9493670886076	24.050632911392400	77.31958762886600
48		72.40000000000000	74.34944237918220	0.7281550225475460	0.7936507936507940	0.6993006993006990	69.11764705882350	63.1578947368421	0.6815789473684210	0.6666666666666670	0.6	40.0	76.31578947368420	23.684210526315800	60.0	73.62637362637360	77.35849056603770	0.7396434526099780	0.8282828282828280	0.7256637168141590	27.43362831858410	75.36231884057970	24.637681159420300	72.56637168141590
49		81.6	82.30769230769230	0.8197144887281180	0.8699186991869920	0.781021897810219	79.71014492753620	78.7878787878788	0.7966386554621850	0.8125	0.7647058823529410	23.52941176470590	82.85714285714290	17.142857142857100	76.47058823529410	82.32044198895030	83.50515463917530	0.8291013193925820	0.8901098901098900	0.7864077669902910	21.35922330097090	87.17948717948720	12.82051282051280	78.64077669902910
50		72.8	69.91150442477880	0.7356831022085260	0.8404255319148940	0.5984848484848490	64.0	50.909090909090900	0.6365576102418210	0.7777777777777780	0.3783783783783780	62.16216216216220	89.47368421052630	10.526315789473700	37.83783783783780	76.57142857142860	76.0233918128655	0.7733552631578950	0.8552631578947370	0.6842105263157900	31.57894736842110	86.25	13.750000000000000	68.42105263157900
51		76.4	75.31380753138080	0.768875192604006	0.8411214953271030	0.6818181818181820	67.10526315789470	52.83018867924530	0.642353770260747	0.7	0.42424242424242400	57.57575757575760	86.04651162790700	13.953488372093000	42.42424242424240	80.45977011494250	81.72043010752690	0.8105050505050510	0.8735632183908050	0.7676767676767680	23.232323232323200	85.33333333333330	14.666666666666700	76.76767676767680
52		81.6	79.82456140350880	0.8167562724014340	0.8921568627450980	0.7222222222222220	78.37837837837840	65.21739130434780	0.7386363636363640	0.9375	0.5	50.0	97.72727272727270	2.272727272727270	50.0	82.95454545454550	83.51648351648350	0.8333333333333330	0.8837209302325580	0.7916666666666670	20.833333333333300	87.5	12.5	79.16666666666670
53		78.4	76.10619469026550	0.7814102564102570	0.8113207547169810	0.7166666666666670	69.41176470588240	58.06451612903230	0.6671428571428570	0.6666666666666670	0.5142857142857140	48.57142857142860	82.0	18.0	51.42857142857140	83.03030303030300	82.9268292682927	0.8312500000000000	0.8607594936708860	0.8	20.0	86.25	13.750000000000000	80.0
54		73.2	72.65306122448980	0.7354262968669750	0.7876106194690270	0.6742424242424240	73.33333333333330	68.75	0.7259684361549500	0.7333333333333330	0.6470588235294120	35.294117647058800	80.48780487804880	19.51219512195120	64.70588235294120	73.14285714285710	74.03314917127070	0.7379406307977740	0.8072289156626510	0.6836734693877550	31.63265306122450	79.22077922077920	20.77922077922080	68.36734693877550
55		68.8	68.0327868852459	0.6877920747711410	0.6859504132231410	0.6747967479674800	66.12903225806450	57.14285714285710	0.644973544973545	0.6363636363636360	0.5185185185185190	48.148148148148100	77.14285714285720	22.857142857142900	51.85185185185190	69.68085106382980	70.76923076923080	0.6963315217391300	0.696969696969697	0.71875	28.125	67.3913043478261	32.608695652173900	71.875
56		78.4	76.52173913043480	0.783410138248848	0.8301886792452830	0.7096774193548390	75.0	60.46511627906980	0.7041553748870820	0.8125	0.48148148148148100	51.85185185185190	92.6829268292683	7.317073170731710	48.148148148148100	79.67032967032970	80.21390374331550	0.7983626440266830	0.8333333333333330	0.7731958762886600	22.68041237113400	82.35294117647060	17.647058823529400	77.31958762886600
57		74.4	74.8031496062992	0.7449483610237990	0.7723577235772360	0.7251908396946570	68.75	62.96296296296300	0.6803921568627450	0.7083333333333330	0.5666666666666670	43.333333333333300	79.41176470588240	20.588235294117600	56.666666666666700	76.34408602150540	78.0	0.7626092020966800	0.7878787878787880	0.7722772277227720	22.772277227722800	75.29411764705880	24.705882352941200	77.22772277227720
58		77.2	75.10917030567680	0.7699433865156970	0.7610619469026550	0.7413793103448280	74.28571428571430	66.66666666666670	0.7249789739276700	0.72	0.6206896551724140	37.93103448275860	82.92682926829270	17.073170731707300	62.06896551724140	78.33333333333330	77.71428571428570	0.7832777159807190	0.7727272727272730	0.7816091954022990	21.839080459770100	78.49462365591400	21.50537634408600	78.16091954022990
59		78.8	76.65198237885460	0.78539354673167	0.8055555555555560	0.7310924369747900	73.61111111111110	61.22448979591840	0.7	0.6818181818181820	0.5555555555555560	44.44444444444440	84.44444444444440	15.555555555555600	55.55555555555560	80.89887640449440	80.89887640449440	0.8099089989888780	0.8372093023255810	0.782608695652174	21.73913043478260	83.72093023255820	16.27906976744190	78.26086956521740
60		78.8	78.00829875518670	0.7887779271493500	0.8245614035087720	0.7401574803149610	77.92207792207790	70.17543859649120	0.7573632538569420	0.7692307692307690	0.6451612903225810	35.483870967741900	86.95652173913040	13.043478260869600	64.51612903225810	79.1907514450867	80.43478260869570	0.7945075757575760	0.8409090909090910	0.7708333333333330	22.916666666666700	81.81818181818180	18.181818181818200	77.08333333333330
61		74.8	74.07407407407410	0.7477434223161130	0.75	0.7317073170731710	70.49180327868850	60.86956521739130	0.6899766899766900	0.5833333333333330	0.6363636363636360	36.36363636363640	74.35897435897440	25.64102564102560	63.63636363636360	76.19047619047620	77.15736040609140	0.7626012601260130	0.7916666666666670	0.7524752475247530	24.752475247524800	77.27272727272730	22.727272727272700	75.24752475247520
62		79.60000000000000	77.5330396475771	0.7938689217758990	0.8301886792452830	0.7272727272727270	76.71232876712330	62.22222222222220	0.7157809983896940	0.7777777777777780	0.5185185185185190	48.148148148148100	91.30434782608700	8.695652173913040	51.85185185185190	80.7909604519774	81.31868131868130	0.8092796718790050	0.8409090909090910	0.7872340425531920	21.27659574468090	83.13253012048190	16.867469879518100	78.72340425531920
63		75.6	74.8971193415638	0.7562724014336920	0.7777777777777780	0.7222222222222220	78.75	73.015873015873	0.7730158730158730	0.8214285714285710	0.6571428571428570	34.285714285714300	88.88888888888890	11.11111111111110	65.71428571428570	74.11764705882350	75.55555555555550	0.7407149812213100	0.7640449438202250	0.7472527472527470	25.274725274725300	73.41772151898740	26.582278481012700	74.72527472527470
64		78.8	78.18930041152260	0.7903846153846160	0.8407079646017700	0.7307692307692310	78.08219178082190	66.66666666666670	0.7417711598746080	0.8421052631578950	0.5517241379310350	44.827586206896600	93.18181818181820	6.8181818181818200	55.172413793103400	79.09604519774010	81.02564102564100	0.7924048983845750	0.8404255319148940	0.7821782178217820	21.782178217821800	80.26315789473690	19.736842105263200	78.21782178217820
65		78.0	78.43137254901960	0.7834138486312400	0.8333333333333330	0.7407407407407410	67.6923076923077	57.14285714285710	0.6539961013645230	0.6363636363636360	0.5185185185185190	48.148148148148100	78.94736842105260	21.052631578947400	51.85185185185190	81.62162162162160	83.49514563106800	0.8202260702260700	0.8775510204081630	0.7962962962962960	20.37037037037040	84.4155844155844	15.584415584415600	79.62962962962960
66		70.8	70.68273092369480	0.708974358974359	0.6821705426356590	0.7333333333333330	70.0	61.81818181818180	0.6955555555555560	0.5666666666666670	0.68	32.0	71.11111111111110	28.888888888888900	68.0	71.11111111111110	73.19587628865980	0.7089783281733750	0.7171717171717170	0.7473684210526320	25.263157894736800	67.05882352941180	32.94117647058820	74.73684210526320
67		78.8	77.82426778242680	0.7874975993854430	0.8017241379310350	0.7560975609756100	77.02702702702700	67.9245283018868	0.7482269503546100	0.6923076923076920	0.6666666666666670	33.33333333333330	82.97872340425530	17.02127659574470	66.66666666666670	79.54545454545460	80.64516129032260	0.796875	0.8333333333333330	0.78125	21.875	81.25	18.75	78.125
68		73.6	73.17073170731710	0.7360399590163940	0.7258064516129030	0.7377049180327870	75.0	65.38461538461540	0.7240259740259740	0.7083333333333330	0.6071428571428570	39.285714285714300	84.0909090909091	15.909090909090900	60.71428571428570	73.03370786516850	75.25773195876290	0.7275835866261400	0.73	0.776595744680851	22.340425531914900	67.85714285714290	32.142857142857100	77.6595744680851
69		77.2	75.32467532467530	0.7701263711591510	0.7767857142857140	0.7310924369747900	77.27272727272730	66.66666666666670	0.7384615384615390	0.7894736842105260	0.5769230769230770	42.30769230769230	90.0	10.0	57.692307692307700	77.17391304347830	77.41935483870970	0.7717121588089330	0.7741935483870970	0.7741935483870970	22.58064516129030	76.92307692307690	23.076923076923100	77.41935483870970
70		78.8	78.00829875518670	0.7877624167946750	0.8034188034188040	0.7580645161290320	72.72727272727270	55.00000000000000	0.6696428571428570	0.6875	0.4583333333333330	54.166666666666700	88.09523809523810	11.904761904761900	45.83333333333330	80.97826086956520	82.58706467661690	0.8078571428571430	0.8217821782178220	0.83	17.0	78.57142857142860	21.428571428571400	83.0
71		74.8	71.74887892376680	0.7442595459236330	0.7339449541284400	0.7017543859649120	68.91891891891890	59.64912280701750	0.6708812260536400	0.6071428571428570	0.5862068965517240	41.37931034482760	75.55555555555560	24.444444444444400	58.620689655172400	77.27272727272730	75.90361445783130	0.7716871363930190	0.7777777777777780	0.7411764705882350	25.882352941176500	80.21978021978020	19.78021978021980	74.11764705882350
72		74.8	73.19148936170210	0.7485279057859700	0.7889908256880730	0.6825396825396830	75.71428571428570	66.66666666666670	0.7357320099255580	0.85	0.5483870967741940	45.16129032258060	92.3076923076923	7.6923076923076900	54.83870967741940	74.44444444444440	75.00000000000000	0.7455108359133130	0.7752808988764050	0.7263157894736840	27.368421052631600	76.47058823529410	23.52941176470590	72.63157894736840
73		78.0	77.17842323651450	0.7804314329738060	0.7560975609756100	0.788135593220339	76.92307692307690	71.69811320754720	0.7597465886939570	0.7307692307692310	0.7037037037037040	29.629629629629600	81.57894736842110	18.421052631578900	70.37037037037040	78.37837837837840	78.72340425531910	0.7842529810614920	0.7628865979381440	0.8131868131868130	18.681318681318700	75.53191489361700	24.46808510638300	81.31868131868130
74		76.4	76.11336032388670	0.7643876832469110	0.7833333333333330	0.7401574803149610	72.6027397260274	62.96296296296300	0.7035714285714290	0.6538461538461540	0.6071428571428570	39.285714285714300	80.0	20.0	60.71428571428570	77.96610169491530	79.79274611398960	0.7799145299145300	0.8191489361702130	0.7777777777777780	22.22222222222220	78.2051282051282	21.794871794871800	77.77777777777780
75		80.0	77.27272727272730	0.7982843607963640	0.8762886597938140	0.6910569105691060	80.51948051948050	70.58823529411760	0.7680851063829790	0.8571428571428570	0.6	40.0	93.61702127659580	6.382978723404260	60.0	79.76878612716760	79.28994082840240	0.8039650537634410	0.881578947368421	0.7204301075268820	27.956989247311800	88.75	11.25	72.04301075268820
76		71.6	70.53941908713690	0.7185897435897440	0.7657657657657660	0.6538461538461540	61.64383561643840	50.0	0.5948540706605220	0.56	0.45161290322580600	54.83870967741940	73.80952380952380	26.190476190476200	45.16129032258060	75.70621468926550	76.75675675675670	0.7624320124320120	0.8255813953488370	0.7171717171717170	28.28282828282830	80.76923076923080	19.230769230769200	71.71717171717170
77		77.2	76.15062761506280	0.7750000000000000	0.8348623853211010	0.7	67.90123456790120	55.172413793103400	0.6524844720496890	0.6956521739130440	0.45714285714285700	54.285714285714300	84.78260869565220	15.217391304347800	45.714285714285700	81.65680473372780	82.87292817679560	0.8204125177809390	0.872093023255814	0.7894736842105260	21.052631578947400	85.13513513513510	14.864864864864900	78.94736842105260
78		72.40000000000000	72.28915662650600	0.724	0.7258064516129030	0.72	67.16417910447760	65.625	0.6743243243243240	0.6176470588235290	0.7	30.0	64.86486486486490	35.13513513513510	70.0	74.31693989071040	74.5945945945946	0.7438397129186600	0.7666666666666670	0.7263157894736840	27.368421052631600	76.13636363636360	23.863636363636400	72.63157894736840
79		76.8	75.42372881355930	0.768	0.8018018018018020	0.712	72.05882352941180	53.65853658536590	0.661860465116279	0.6875	0.44	56.00000000000000	88.37209302325580	11.627906976744200	44.0	78.57142857142860	80.0	0.7863414634146340	0.8210526315789470	0.78	22.0	79.26829268292680	20.73170731707320	78.0
80		77.60000000000000	75.65217391304350	0.777479034632866	0.8446601941747570	0.6850393700787400	71.05263157894740	50.0	0.6426282051282050	0.55	0.4583333333333330	54.166666666666700	82.6923076923077	17.307692307692300	45.83333333333330	80.45977011494250	81.72043010752690	0.8196362641870640	0.9156626506024100	0.7378640776699030	26.21359223300970	90.14084507042250	9.859154929577460	73.7864077669903
81		76.4	74.235807860262	0.7618589743589740	0.7798165137614680	0.7083333333333330	77.63157894736840	60.46511627906980	0.7165709598031170	0.65	0.5652173913043480	43.47826086956520	86.79245283018870	13.20754716981130	56.52173913043480	75.86206896551720	77.41935483870970	0.7607444102289460	0.8089887640449440	0.7422680412371130	25.773195876288700	77.92207792207790	22.07792207792210	74.22680412371140
82		77.60000000000000	77.04918032786880	0.7789466931810890	0.831858407079646	0.7175572519083970	80.0	73.33333333333330	0.7800511508951410	0.8461538461538460	0.6470588235294120	35.294117647058800	91.30434782608700	8.695652173913040	64.70588235294120	76.47058823529410	78.26086956521740	0.7683942945911600	0.8275862068965520	0.7422680412371130	25.773195876288700	79.45205479452060	20.54794520547950	74.22680412371140
83		78.8	75.34883720930230	0.7850922131147540	0.8709677419354840	0.6639344262295080	81.94444444444440	68.29268292682930	0.7604166666666670	0.8235294117647060	0.5833333333333330	41.66666666666670	93.75	6.25	58.333333333333300	77.52808988764050	77.01149425287360	0.7855867346938780	0.881578947368421	0.6836734693877550	31.63265306122450	88.75	11.25	68.36734693877550
84		76.4	75.31380753138070	0.7738648090815270	0.8737864077669900	0.6617647058823530	77.77777777777780	66.66666666666670	0.7434292866082600	0.9	0.5294117647058820	47.05882352941180	95.74468085106380	4.25531914893617	52.94117647058820	75.7396449704142	77.83783783783780	0.7708516242317820	0.8674698795180720	0.7058823529411770	29.411764705882400	83.5820895522388	16.417910447761200	70.58823529411770
85		76.4	76.6798418972332	0.7641521516393440	0.776	0.7578125	71.21212121212120	55.81395348837210	0.6746031746031750	0.5454545454545450	0.5714285714285710	42.857142857142900	77.77777777777780	22.22222222222220	57.14285714285710	78.26086956521740	80.95238095238100	0.7803131447991260	0.8252427184466020	0.794392523364486	20.5607476635514	76.62337662337660	23.376623376623400	79.43925233644860
86		79.2	78.33333333333330	0.7928429677997570	0.831858407079646	0.7401574803149610	75.0	63.63636363636360	0.7165991902834010	0.7777777777777780	0.5384615384615380	46.15384615384620	89.47368421052630	10.526315789473700	53.84615384615390	80.64516129032260	81.63265306122450	0.807804309842749	0.8421052631578950	0.7920792079207920	20.792079207920800	82.35294117647060	17.647058823529400	79.20792079207920
87		70.8	66.9683257918552	0.7124415401371010	0.8043478260869570	0.5736434108527130	74.66666666666670	62.745098039215700	0.711111111111111	0.7619047619047620	0.5333333333333330	46.666666666666700	88.88888888888890	11.11111111111110	53.333333333333300	69.14285714285710	68.23529411764710	0.7074029771398190	0.8169014084507040	0.5858585858585860	41.41414141414140	82.89473684210530	17.105263157894700	58.58585858585860
88		78.0	77.55102040816330	0.7802099334357400	0.7983193277310930	0.753968253968254	72.97297297297300	61.53846153846150	0.7077551020408160	0.5925925925925930	0.64	36.0	77.55102040816330	22.448979591836700	64.0	80.11363636363640	81.86528497409330	0.8044224422442240	0.8586956521739130	0.7821782178217820	21.782178217821800	82.66666666666670	17.333333333333300	78.21782178217820
89		79.2	76.57657657657660	0.7881997945557270	0.8173076923076920	0.7203389830508470	73.91304347826090	59.09090909090910	0.6918604651162790	0.7222222222222220	0.5	50.0	88.37209302325580	11.627906976744200	50.0	81.21546961325970	80.89887640449440	0.8126526624328290	0.8372093023255810	0.782608695652174	21.73913043478260	84.26966292134830	15.730337078651700	78.26086956521740
90		76.4	75.10548523206750	0.764	0.7946428571428570	0.712	70.83333333333330	60.377358490566000	0.6833333333333330	0.6956521739130440	0.5333333333333330	46.666666666666700	83.33333333333330	16.666666666666700	53.333333333333300	78.65168539325840	79.34782608695650	0.7878249841471150	0.8202247191011240	0.7684210526315790	23.157894736842100	80.72289156626510	19.27710843373490	76.84210526315790
91		76.0	76.56250000000000	0.7602564102564100	0.7777777777777780	0.7538461538461540	74.64788732394370	66.66666666666670	0.7404347826086960	0.6206896551724140	0.72	28.000000000000000	76.08695652173910	23.91304347826090	72.0	76.53631284916200	79.20792079207920	0.7660875160875160	0.8247422680412370	0.7619047619047620	23.809523809523800	77.02702702702700	22.972972972973000	76.19047619047620
92		73.6	72.26890756302520	0.7354202675885030	0.7478260869565220	0.6991869918699190	63.23529411764710	44.44444444444450	0.5837209302325580	0.5	0.4	60.0	76.74418604651160	23.25581395348840	40.0	77.47252747252750	78.75647668393780	0.7746598639455780	0.8	0.7755102040816330	22.448979591836700	77.38095238095240	22.61904761904760	77.55102040816330
93		78.0	75.77092511013220	0.7778525209814850	0.8113207547169810	0.7107438016528930	70.4225352112676	60.377358490566000	0.6813008130081300	0.6956521739130440	0.5333333333333330	46.666666666666700	82.92682926829270	17.073170731707300	53.333333333333300	81.00558659217880	80.45977011494250	0.8107517482517480	0.8433734939759040	0.7692307692307690	23.076923076923100	85.22727272727270	14.772727272727300	76.92307692307690
94		78.4	78.74015748031500	0.7861962598804700	0.8264462809917360	0.7518796992481200	72.85714285714290	65.45454545454550	0.7125	0.72	0.6	40.0	82.5	17.5	60.0	80.55555555555560	82.41206030150750	0.8071491615180940	0.8541666666666670	0.7961165048543690	20.388349514563100	81.81818181818180	18.181818181818200	79.6116504854369
95		76.8	76.03305785123970	0.7683051715309780	0.7931034482758620	0.7301587301587300	78.78787878787880	69.56521739130430	0.7590243902439030	0.7619047619047620	0.64	36.0	87.8048780487805	12.195121951219500	64.0	76.08695652173910	77.55102040816330	0.761779792437075	0.8	0.7524752475247530	24.752475247524800	77.10843373493980	22.89156626506020	75.24752475247520
96		78.0	74.4186046511628	0.7742111689480110	0.8163265306122450	0.6837606837606840	73.4375	60.46511627906980	0.6958333333333330	0.6842105263157900	0.5416666666666670	45.83333333333330	85.0	15.0	54.166666666666700	79.56989247311830	77.90697674418610	0.7956989247311830	0.8481012658227850	0.7204301075268820	27.956989247311800	87.09677419354840	12.903225806451600	72.04301075268820
97		77.2	76.92307692307690	0.7720056334421610	0.7661290322580650	0.7723577235772360	76.0	66.66666666666670	0.7395833333333330	0.6666666666666670	0.6666666666666670	33.33333333333330	81.25	18.75	66.66666666666670	77.71428571428570	79.79274611398960	0.7744593881856540	0.7938144329896910	0.8020833333333330	19.791666666666700	74.68354430379750	25.31645569620250	80.20833333333330
98		79.60000000000000	78.83817427385890	0.7956276806862560	0.8050847457627120	0.7723577235772360	82.08955223880600	70.0	0.7789855072463770	0.7368421052631580	0.6666666666666670	33.33333333333330	89.13043478260870	10.869565217391300	66.66666666666670	78.68852459016390	80.59701492537310	0.7859477124183010	0.8181818181818180	0.7941176470588240	20.588235294117600	77.77777777777780	22.22222222222220	79.41176470588240
99		80.0	78.63247863247860	0.800563236047107	0.8518518518518520	0.7301587301587300	75.30864197530870	66.66666666666670	0.7352941176470590	0.6666666666666670	0.6666666666666670	33.33333333333330	80.3921568627451	19.607843137254900	66.66666666666670	82.24852071005920	82.75862068965520	0.8339041095890410	0.9230769230769230	0.75	25.0	91.78082191780820	8.21917808219178	75.0
	**Mean**	**76.9**	**75.653126674233**	**0.769183861844112**	**0.8031868350920230**	**0.717159708636661**	**74.44893613509040**	**63.5031149524489**	**0.714913128884105**	**0.725115967482427**	**0.576174597400665**	**42.3825402599335**	**85.3651660367544**	**14.6348339632456**	**57.6174597400665**	**77.86680220052430**	**78.86683851384480**	**0.781031721048063**	**0.8236602674453410**	**0.7583014971612600**	**24.169850283874**	**80.3761944934866**	**19.6238055065134**	**75.83014971612600**
	**Standard Deviation**	2.7	2.941149535929	0.0265223004814519	0.0423565125883110	0.0409891625029152	4.79004295506506	7.71175988443235	0.050824722964194	0.0957551704202577	0.101211995116463	10.1211995116463	6.3941320252981	6.39413202529809	10.1211995116463	3.12315359157320	3.13516524912693	0.0319016850008687	0.0443225847595874	0.0385260255944459	3.8526025594446	5.24082996398904	5.24082996398904	3.85260255944459
	**T Test Significance (Female to Male)**						**0.0000087924095343**	**0.0000000000000000**	**0.00000000000014296**	**0.00000000000013797**	**0.0000000000000000**	**0.0000000000000000**	**0.0002556167457358**	**0.0000000380055426**	**0.0000000000000000**									

Experiment 3.1.3 (Unbalanced training data, with feature selection) - Logistic Regression Classifier

		UB_Accuracy	UB_FScore	UB_ROC_AUC	UB_Precision	UB_Recall	UB_F_Accuracy	UB_F_FScore	UB_F_ROC_AUC	UB_F_Precision	UB_F_Recall	UB_F_FNR
0		73.6	70.53571428571430	0.736000000000000	0.797979797979798	0.632	75.38461538461540	65.21739130434780	0.724358974358974	0.75	0.576923076923077	42.30769230769230
1		70.8	66.04651162790700	0.709165386584741	0.797752808988764	0.563492063492064	73.61111111111110	51.28205128205130	0.662962962962963	0.833333333333333	0.370370370370370	62.96296296296300
2		69.20000000000000	65.77777777777780	0.695912614517266	0.770833333333333	0.573643410852713	70.76923076923080	57.77777777777780	0.682471264367816	0.8125	0.448275862068966	55.17241379310340
3		70.40000000000000	67.2566371681416	0.710244403104754	0.8	0.580152671755725	68.57142857142860	50.0	0.651778329197684	0.846153846153846	0.354838709677419	64.51612903225810
4		70.40000000000000	65.09433962264150	0.705261136712750	0.802325581395349	0.547619047619048	76.71232876712330	58.53658536585370	0.700483091787440	0.857142857142857	0.444444444444444	55.55555555555560
5		68.0	62.26415094339620	0.678827444956477	0.75	0.532258064516129	69.44444444444440	52.17391304347830	0.652380952380952	0.75	0.4	60.0
6		65.2	62.00873362445420	0.652713773681516	0.689320388349515	0.563492063492064	68.57142857142860	47.61904761904760	0.624125874125874	0.625	0.384615384615385	61.53846153846150
7		74.4	71.42857142857140	0.741931352459016	0.784313725490196	0.655737704918033	78.57142857142860	65.11627906976740	0.737318840579710	0.736842105263158	0.583333333333333	41.66666666666670
8		69.20000000000000	62.06896551724140	0.693548387096774	0.818181818181818	0.5	71.64179104477610	45.71428571428570	0.636190476190476	0.8	0.32	68.0
9		72.8	68.80733944954130	0.728000000000000	0.806451612903226	0.6	70.4225352112676	46.15384615384620	0.632575757575758	0.75	0.333333333333333	66.66666666666670
10		68.0	62.61682242990650	0.681195596518177	0.761363636363636	0.531746031746032	68.4931506849315	46.51162790697670	0.623015873015873	0.666666666666667	0.357142857142857	64.28571428571430
11		70.40000000000000	65.09433962264150	0.705261136712750	0.802325581395349	0.547619047619048	67.53246753246750	41.86046511627910	0.603089080459770	0.642857142857143	0.310344827586207	68.96551724137930
12		69.6	67.24137931034480	0.696620583717358	0.735849056603774	0.619047619047619	76.81159420289860	61.90476190476190	0.715116279069768	0.8125	0.5	50.0
13		70.0	64.78873239436620	0.692337461300310	0.696969696969697	0.605263157894737	75.0	53.65853658536580	0.671538461538462	0.733333333333333	0.423076923076923	57.69230769230770
14		69.20000000000000	66.07929515418500	0.698024247867086	0.78125	0.572519083969466	72.05882352941180	59.57446808510640	0.690097259062776	0.777777777777778	0.482758620689655	51.72413793103450
15		69.6	65.13761467889910	0.708890890375708	0.845238095238095	0.529850746268657	66.66666666666670	46.80851063829790	0.637374461979914	0.846153846153846	0.323529411764706	67.64705882352940
16		72.40000000000000	69.33333333333330	0.724000000000000	0.78	0.624	71.08433734939760	53.84615384615390	0.659926470588235	0.7	0.4375	56.25
17		69.20000000000000	66.95278969957080	0.694031762295082	0.742857142857143	0.609375	66.66666666666670	46.80851063829790	0.620601173020528	0.6875	0.354838709677419	64.51612903225810
18		66.8	62.78026905829600	0.679297478126608	0.786516853932584	0.522388059701493	65.0	40.0	0.605203619909502	0.777777777777778	0.269230769230769	73.07692307692310
19		68.0	63.96396396396400	0.685576923076923	0.771739130434783	0.546153846153846	65.75342465753420	48.9795918367347	0.638009049773756	0.8	0.352941176470588	64.70588235294120
20		72.8	68.22429906542060	0.722753223426775	0.768421052631579	0.613445378151261	79.6875	55.17241379310340	0.692740926157697	0.666666666666667	0.470588235294118	52.94117647058820
21		69.6	66.66666666666670	0.700641025641026	0.775510204081633	0.584615384615385	67.6923076923077	51.16279069767440	0.647988505747126	0.785714285714286	0.379310344827586	62.06896551724140
22		74.0	68.29268292682930	0.730273752012883	0.777777777777778	0.608695652173913	70.4225352112676	55.31914893617020	0.664614121510673	0.722222222222222	0.448275862068966	55.17241379310340
23		74.0	68.89952153110050	0.735505157281056	0.818181818181818	0.595041322314050	68.75	50.98039215686280	0.652380952380952	0.8125	0.371428571428571	62.85714285714290
24		72.40000000000000	68.4931506849315	0.727394979508197	0.824175824175824	0.5859375	76.0	59.09090909090910	0.699074074074074	0.764705882352941	0.481481481481481	51.85185185185190
25		72.0	65.68627450980390	0.714844000256263	0.807228915662651	0.553719008264463	74.66666666666670	53.65853658536580	0.675151975683891	0.846153846153846	0.392857142857143	60.71428571428570
26		68.8	64.22018348623850	0.702737520128825	0.843373493975904	0.518518518518519	73.86363636363640	58.18181818181820	0.690835579514825	0.8	0.457142857142857	54.28571428571430
27		68.8	63.88888888888890	0.685130635245902	0.734042553191489	0.565573770491803	76.0	60.8695652173913	0.708770614692654	0.823529411764706	0.482758620689655	51.72413793103450
28		67.2	64.03508771929830	0.690517855985482	0.820224719101124	0.525179856115108	67.5	50.0	0.641269841269841	0.764705882352941	0.371428571428571	62.85714285714290
29		71.6	69.7872340425532	0.720540124446725	0.788461538461538	0.625954198473283	71.83098591549300	54.54545454545460	0.675609756097561	0.857142857142857	0.4	60.0
30		73.2	67.3170731707317	0.725961538461538	0.811764705882353	0.575	70.66666666666670	47.61904761904760	0.644444444444445	0.833333333333333	0.333333333333333	66.66666666666670
31		66.0	62.882096069869	0.661513347416939	0.705882352941177	0.566929133858268	71.23287671232880	48.78048780487810	0.641706924315620	0.714285714285714	0.370370370370370	62.96296296296300
32		68.4	62.20095693779910	0.676938880328711	0.714285714285714	0.550847457627119	71.05263157894740	50.0	0.644345238095238	0.6875	0.392857142857143	60.71428571428570
33		71.2	66.35514018691590	0.709877728698547	0.780219780219780	0.577235772357724	80.28169014084510	61.11111111111110	0.718297101449275	0.846153846153846	0.478260869565217	52.17391304347830
34		71.6	67.8733031674208	0.721794871794872	0.824175824175824	0.576923076923077	73.52941176470590	57.14285714285710	0.689285714285714	0.857142857142857	0.428571428571429	57.14285714285710
35		68.4	61.83574879227050	0.675759912602018	0.711111111111111	0.547008547008547	77.46478873239440	57.89473684210530	0.697251773049645	0.785714285714286	0.458333333333333	54.16666666666670
36		68.0	62.61682242990650	0.681195596518177	0.761363636363636	0.531746031746032	72.72727272727270	43.24324324324320	0.624434389140272	0.727272727272727	0.307692307692308	69.23076923076920
37		69.20000000000000	63.1578947368421	0.687455954897815	0.75	0.545454545454545	68.18181818181820	27.58620689655170	0.550793650793651	0.5	0.190476190476190	80.95238095238100
38		70.0	65.75342465753430	0.700000000000000	0.765957446808511	0.576	72.6027397260274	54.54545454545450	0.669841269841270	0.75	0.428571428571429	57.14285714285710
39		67.2	63.06306306306310	0.691123188405797	0.833333333333333	0.507246376811594	64.38356164383560	40.90909090909090	0.604039634146341	0.75	0.28125	71.875
40		70.8	67.55555555555560	0.708845366103431	0.767676767676768	0.603174603174603	63.63636363636360	50.0	0.612301957129543	0.631578947368421	0.413793103448276	58.62068965517240
41		72.40000000000000	69.33333333333330	0.725785801165098	0.795918367346939	0.614173228346457	75.38461538461540	55.55555555555560	0.6875	0.909090909090909	0.4	60.0
42		70.8	63.31658291457290	0.693075117370892	0.692307692307692	0.583333333333333	77.33333333333330	37.03703703703700	0.604793233082707	0.625	0.263157894736842	73.68421052631580
43		68.8	64.54545454545450	0.682900154400412	0.682692307692308	0.612068965517241	72.05882352941180	57.77777777777780	0.682142857142857	0.764705882352941	0.464285714285714	53.57142857142860
44		67.2	64.03508771929820	0.679057524396507	0.760416666666667	0.553030303030303	81.57894736842110	70.83333333333330	0.772463768115942	0.944444444444444	0.566666666666667	43.33333333333330
45		74.0	67.00507614213200	0.729419703103914	0.825	0.564102564102564	74.35897435897440	50.0	0.655773420479303	0.769230769230769	0.370370370370370	62.96296296296300
46		73.6	68.26923076923080	0.732389856557377	0.825581395348837	0.581967213114754	75.0	54.54545454545450	0.675824175824176	0.75	0.428571428571429	57.14285714285710
47		70.0	66.66666666666670	0.702804815573771	0.773195876288660	0.5859375	68.65671641791050	48.78048780487800	0.653153153153153	0.909090909090909	0.333333333333333	66.66666666666670
48		67.2	64.95726495726500	0.682920536635707	0.775510204081633	0.558823529411765	69.86301369863010	50.0	0.648449612403101	0.785714285714286	0.366666666666667	63.33333333333330
49		70.8	65.40284360189570	0.701463790446841	0.741935483870968	0.584745762711864	71.23287671232880	53.33333333333330	0.657004830917874	0.666666666666667	0.444444444444444	55.55555555555560
50		71.6	67.57990867579910	0.718167850969848	0.804347826086957	0.582677165354331	70.27027027027030	54.16666666666670	0.659848484848485	0.722222222222222	0.433333333333333	56.66666666666670
51		70.8	66.66666666666670	0.710165802445426	0.793478260869565	0.574803149606299	69.62025316455700	52.00000000000000	0.653491436100132	0.764705882352941	0.393939393939394	60.60606060606060
52		71.2	65.04854368932040	0.705176727179421	0.770114942528736	0.563025210084034	73.75	55.31914893617020	0.686331399097357	0.928571428571429	0.393939393939394	60.60606060606060
53		74.0	70.04608294930880	0.735358265443582	0.775510204081633	0.638655462184874	76.47058823529410	60.0	0.705116279069767	0.8	0.48	52.0
54		68.8	63.20754716981130	0.695192307692308	0.817073170731707	0.515384615384615	67.12328767123290	47.82608695652170	0.641666666666667	0.846153846153846	0.333333333333333	66.66666666666670
55		70.0	63.05418719211820	0.697170475641764	0.8	0.520325203252033	72.72727272727270	51.16279069767440	0.662056737588653	0.846153846153846	0.366666666666667	63.33333333333330
56		70.0	63.76811594202900	0.696273053278689	0.776470588235294	0.540983606557377	72.22222222222220	56.52173913043480	0.690625	0.928571428571429	0.40625	59.375
57		72.8	69.36936936936940	0.724679487179487	0.754901960784314	0.641666666666667	80.95238095238100	66.66666666666670	0.75	0.8	0.571428571428571	42.85714285714290
58		70.40000000000000	67.2566371681416	0.702100409836066	0.730769230769231	0.622950819672131	75.0	58.53658536585370	0.695054945054945	0.8	0.461538461538462	53.84615384615390
59		75.6	73.36244541484720	0.756000000000000	0.807692307692308	0.672	81.15942028985510	62.85714285714290	0.728723404255319	0.846153846153846	0.5	50.0
60		64.4	58.98617511520740	0.641201331967213	0.673684210526316	0.524590163934426	70.83333333333330	55.31914893617020	0.663961038961039	0.684210526315790	0.464285714285714	53.57142857142860
61		67.2	60.19417475728160	0.669355354970873	0.746987951807229	0.504065040650407	75.67567567567570	52.63157894736840	0.668333333333333	0.714285714285714	0.416666666666667	58.33333333333330
62		67.60000000000000	65.82278481012660	0.681047765793528	0.742857142857143	0.590909090909091	65.21739130434780	47.82608695652170	0.619230769230769	0.6875	0.366666666666667	63.33333333333330
63		70.8	65.07177033492820	0.703472355692229	0.772727272727273	0.561983471074380	70.12987012987010	46.51162790697680	0.630747126436782	0.714285714285714	0.344827586206897	65.51724137931040
64		69.20000000000000	63.50710900473930	0.696144979508197	0.807228915662651	0.5234375	71.42857142857140	50.0	0.653684210526316	0.818181818181818	0.36	64.0
65		71.2	69.4915254237288	0.718527086948140	0.796116504854369	0.616541353383459	70.4225352112676	58.82352941176470	0.683092948717949	0.789473684210526	0.46875	53.125
66		70.8	68.3982683982684	0.721949486467282	0.840425531914894	0.576642335766423	72.8395061728395	56.00000000000000	0.680871212121212	0.823529411764706	0.424242424242424	57.57575757575760
67		71.6	69.26406926406930	0.714587602459016	0.733944954128440	0.655737704918033	72.85714285714290	53.65853658536580	0.668819982773471	0.785714285714286	0.407407407407407	59.25925925925930
68		72.0	67.88990825688070	0.716025641025641	0.755102040816327	0.616666666666667	82.05128205128200	72.00000000000000	0.779732582688248	0.857142857142857	0.620689655172414	37.93103448275860
69		72.0	69.02654867256640	0.721720760514692	0.787878787878788	0.614173228346457	76.0	64.00000000000000	0.723973607038123	0.842105263157895	0.516129032258065	48.38709677419360
70		67.2	61.6822429906542	0.674476666026503	0.758620689655172	0.519685039370079	68.25396825396830	47.36842105263160	0.632536382536383	0.75	0.346153846153846	65.38461538461540
71		68.8	62.13592233009710	0.681121303483225	0.735632183908046	0.537815126050420	73.41772151898740	51.16279069767440	0.657212885154062	0.733333333333333	0.392857142857143	60.71428571428570
72		71.2	66.97247706422020	0.707049306625578	0.73	0.618644067796610	79.16666666666670	59.45945945945950	0.723435948361470	0.611111111111111	0.578947368421053	42.10526315789470
73		70.40000000000000	64.76190476190480	0.694875776397516	0.693877551020408	0.607142857142857	71.83098591549300	50.0	0.644474637681159	0.588235294117647	0.434782608695652	56.52173913043480
74		70.0	67.24890829694320	0.706920903954802	0.793814432989691	0.583333333333333	66.17647058823530	51.06382978723410	0.645833333333333	0.8	0.375	62.5
75		74.0	70.85201793721970	0.743019979508197	0.831578947368421	0.6171875	70.96774193548390	50.0	0.659188034188034	0.9	0.346153846153846	65.38461538461540
76		74.4	69.52380952380950	0.738782051282051	0.811111111111111	0.608333333333333	77.33333333333330	56.41025641025640	0.691130298273155	0.846153846153846	0.423076923076923	57.69230769230770
77		70.8	65.40284360189570	0.705684655271750	0.784090909090909	0.560975609756098	68.05555555555560	43.90243902439030	0.611111111111111	0.642857142857143	0.333333333333333	66.66666666666670
78		69.6	63.46153846153850	0.691331923890063	0.758620689655172	0.545454545454545	69.35483870967740	51.28205128205130	0.656613756613757	0.833333333333333	0.370370370370370	62.96296296296300
79		67.60000000000000	59.70149253731340	0.668131374687280	0.731707317073171	0.504201680672269	72.85714285714290	45.71428571428570	0.637777777777778	0.8	0.32	68.0
80		75.6	69.34673366834170	0.752928749759939	0.907894736842105	0.560975609756098	75.0	47.05882352941180	0.649361702127660	0.888888888888889	0.32	68.0
81		67.2	60.57692307692310	0.662345201238390	0.670212765957447	0.552631578947369	78.46153846153850	53.33333333333330	0.677917620137300	0.727272727272727	0.421052631578947	57.89473684210530
82		71.6	66.02870813397130	0.712474385245902	0.793103448275862	0.565573770491803	75.75757575757580	61.90476190476190	0.712500000000000	0.8125	0.5	50.0
83		68.0	63.30275229357800	0.679019457245264	0.734042553191489	0.556451612903226	70.12987012987010	51.06382978723400	0.650070126227209	0.75	0.387096774193548	61.29032258064520
84		70.40000000000000	66.96428571428570	0.703213005632361	0.75	0.604838709677419	76.71232876712330	62.22222222222220	0.715780998389694	0.777777777777778	0.518518518518519	48.14814814814810
85		71.6	67.57990867579910	0.713434938524590	0.762886597938144	0.606557377049180	75.34246575342470	60.8695652173913	0.707288401253919	0.823529411764706	0.482758620689655	51.72413793103450
86		70.40000000000000	65.09433962264150	0.699038461538462	0.75	0.575	76.38888888888890	58.53658536585370	0.702922077922078	0.923076923076923	0.428571428571429	57.14285714285710
87		70.0	67.24890829694320	0.702420594262295	0.762376237623762	0.6015625	73.33333333333330	61.53846153846150	0.7	0.727272727272727	0.533333333333333	46.66666666666670
88		68.8	63.55140186915890	0.691854508196721	0.790697674418605	0.53125	78.87323943661970	59.45945945945950	0.707880434782609	0.785714285714286	0.478260869565217	52.17391304347830
89		70.40000000000000	67.82608695652170	0.706326844262295	0.764705882352941	0.609375	71.1864406779661	54.05405405405410	0.661835748792271	0.714285714285714	0.434782608695652	56.52173913043480
90		65.60000000000000	63.24786324786320	0.658722531872638	0.704761904761905	0.573643410852713	74.57627118644070	54.54545454545460	0.673717948717949	0.692307692307692	0.45	55.00000000000000
91		71.6	67.8733031674208	0.716	0.78125	0.6	67.64705882352940	54.16666666666670	0.650877192982456	0.722222222222222	0.433333333333333	56.66666666666670
92		70.8	63.68159203980100	0.701282051282051	0.790123456790123	0.533333333333333	72.0	46.15384615384620	0.632260596546311	0.692307692307692	0.346153846153846	65.38461538461540
93		70.8	66.35944700460830	0.709101382488479	0.791208791208791	0.571428571428571	71.21212121212120	55.81395348837210	0.686111111111111	0.923076923076923	0.4	60.0
94		72.40000000000000	67.90697674418610	0.718936429533646	0.760416666666667	0.613445378151261	74.02597402597400	58.33333333333330	0.690780141843972	0.777777777777778	0.466666666666667	53.33333333333330
95		69.6	65.1376146788991	0.703765475655911	0.816091954022989	0.541984732824428	66.15384615384620	47.61904761904760	0.630747126436782	0.769230769230769	0.344827586206897	65.51724137931040
96		71.2	66.66666666666670	0.718589743589744	0.837209302325581	0.553846153846154	72.85714285714290	48.64864864864860	0.655038759689922	0.9	0.333333333333333	66.66666666666670
97		74.8	73.41772151898740	0.752229135929181	0.820754716981132	0.664122137404580	63.23529411764710	41.86046511627910	0.577838827838828	0.529411764705882	0.346153846153846	65.38461538461540
98		68.8	64.54545454545460	0.687083973374296	0.739583333333333	0.572580645161290	69.1358024691358	44.44444444444440	0.617647058823529	0.666666666666667	0.333333333333333	66.66666666666670
99		71.6	65.70048309178740	0.707891523680998	0.755555555555556	0.581196581196581	77.77777777777780	57.89473684210530	0.698723404255319	0.846153846153846	0.44	56.00000000000000
	**Mean**	**70.344**	**65.8842605682503**	**0.703498316173683**	**0.77376236039132**	**0.575585748030395**	**72.40380626762950**	**53.1293261131455**	**0.666548763543034**	**0.772092649653408**	**0.41052722001394**	**58.947277998606**
	**Standard Deviation**	2.31151356247264	2.86005867936154	0.022120019789737	0.042271599647736	0.038424347707084	4.25725862308382	7.06228922894526	0.039153552780943	0.086613553440046	0.076180787295296	7.61807872952965
	**T Test Significance (Female to Male)**						**0.009696494285988**	**0.000000000000000**	**0.000000390470598**	**0.439466304076613**	**0.000000000000000**	**0.000000000000000**

		UB_F_TNR	UB_F_FPR	UB_F_TPR	UB_M_Accuracy	UB_M_FScore	UB_M_ROC_AUC	UB_M_Precision	UB_M_Recall	UB_M_FNR	UB_M_TNR	UB_M_FPR	UB_M_TPR
0		87.17948717948720	12.82051282051280	57.69230769230770	72.97297297297300	71.91011235955060	0.736023020906742	0.810126582278481	0.646464646464647	35.35353535353540	82.55813953488370	17.44186046511630	64.64646464646470
1		95.55555555555560	4.444444444444450	37.03703703703700	69.66292134831460	69.31818181818180	0.706814985295998	0.792207792207792	0.616161616161616	38.38383838383840	79.74683544303800	20.25316455696200	61.61616161616160
2		91.66666666666670	8.333333333333330	44.82758620689660	68.64864864864870	67.77777777777780	0.693235294117647	0.7625	0.61	39.0	77.64705882352940	22.35294117647060	61.0
3		94.87179487179490	5.128205128205130	35.48387096774190	71.11111111111110	71.42857142857140	0.71875	0.792682926829268	0.65	35.0	78.75	21.25	65.0
4		95.65217391304350	4.347826086956520	44.44444444444440	67.79661016949150	66.66666666666670	0.691724941724942	0.791666666666667	0.575757575757576	42.42424242424240	80.76923076923080	19.23076923076920	57.57575757575760
5		90.47619047619050	9.523809523809520	40.0	67.41573033707870	65.06024096385540	0.680091185410334	0.75	0.574468085106383	42.5531914893617	78.57142857142860	21.42857142857140	57.44680851063830
6		86.36363636363640	13.63636363636360	38.46153846153850	63.88888888888890	65.24064171123000	0.6425	0.701149425287356	0.61	39.0	67.5	32.5	61.0
7		89.13043478260870	10.86956521739130	58.33333333333330	72.77777777777780	72.9281767955801	0.733076157292185	0.795180722891566	0.673469387755102	32.6530612244898	79.26829268292680	20.73170731707320	67.3469387755102
8		95.23809523809520	4.761904761904760	32.0	68.30601092896170	65.47619047619050	0.699106496015455	0.820895522388060	0.544554455445545	45.54455445544560	85.36585365853660	14.63414634146340	54.45544554455450
9		93.18181818181820	6.818181818181820	33.33333333333330	73.74301675977650	73.74301675977650	0.744142101284959	0.814814814814815	0.673469387755102	32.6530612244898	81.48148148148150	18.51851851851850	67.3469387755102
10		88.88888888888890	11.11111111111110	35.71428571428570	67.79661016949150	66.66666666666670	0.689550503745802	0.780821917808219	0.581632653061225	41.83673469387760	79.74683544303800	20.25316455696200	58.16326530612250
11		89.58333333333330	10.41666666666670	31.03448275862070	71.67630057803470	71.00591715976330	0.730330982094411	0.833333333333333	0.618556701030928	38.14432989690720	84.21052631578950	15.78947368421050	61.85567010309280
12		93.02325581395350	6.976744186046510	50.0	66.85082872928180	68.42105263157900	0.670679012345679	0.722222222222222	0.65	35.0	69.1358024691358	30.86419753086420	65.0
13		92.0	8.0	42.30769230769230	67.81609195402300	67.44186046511630	0.678382663847780	0.690476190476191	0.659090909090909	34.09090909090910	69.76744186046510	30.23255813953490	65.9090909090909
14		89.74358974358980	10.25641025641030	48.27586206896550	68.13186813186810	67.77777777777780	0.692769607843137	0.782051282051282	0.598039215686275	40.19607843137260	78.75	21.25	59.80392156862750
15		95.1219512195122	4.878048780487810	32.35294117647060	70.85714285714290	70.17543859649120	0.726666666666667	0.845070422535211	0.6	40.0	85.33333333333330	14.66666666666670	60.0
16		88.23529411764710	11.76470588235290	43.75	73.05389221556890	73.98843930635840	0.735977913397268	0.8	0.688172043010753	31.18279569892470	78.37837837837840	21.62162162162160	68.81720430107530
17		88.63636363636360	11.36363636363640	35.48387096774190	70.28571428571430	72.04301075268820	0.704335183716627	0.752808988764045	0.690721649484536	30.92783505154640	71.7948717948718	28.20512820512820	69.0721649484536
18		94.11764705882350	5.88235294117647	26.92307692307690	67.36842105263160	67.02127659574470	0.688008130081301	0.7875	0.583333333333333	41.66666666666670	79.26829268292680	20.73170731707320	58.33333333333330
19		92.3076923076923	7.692307692307690	35.29411764705880	68.92655367231640	68.20809248554910	0.696180555555556	0.766233766233766	0.614583333333333	38.54166666666670	77.77777777777780	22.22222222222220	61.45833333333330
20		91.48936170212770	8.51063829787234	47.05882352941180	70.43010752688170	70.27027027027030	0.711484593837535	0.783132530120482	0.637254901960784	36.27450980392160	78.57142857142860	21.42857142857140	63.72549019607840
21		91.66666666666670	8.333333333333330	37.93103448275860	70.27027027027030	70.27027027027030	0.708686940122584	0.773809523809524	0.643564356435644	35.64356435643560	77.38095238095240	22.61904761904760	64.35643564356440
22		88.09523809523810	11.90476190476190	44.82758620689660	75.41899441340780	72.15189873417720	0.750750187546887	0.791666666666667	0.662790697674419	33.72093023255810	83.87096774193550	16.12903225806450	66.27906976744190
23		93.33333333333330	6.666666666666670	37.14285714285710	76.47058823529410	74.68354430379750	0.765642303433001	0.819444444444444	0.686046511627907	31.3953488372093	84.52380952380950	15.47619047619050	68.6046511627907
24		91.66666666666670	8.333333333333330	48.14814814814810	70.85714285714290	70.85714285714290	0.725849611988226	0.837837837837838	0.613861386138614	38.61386138613860	83.78378378378380	16.21621621621620	61.38613861386140
25		95.74468085106380	4.25531914893617	39.28571428571430	70.85714285714290	68.71165644171780	0.715709415158668	0.8	0.602150537634409	39.78494623655910	82.92682926829270	17.07317073170730	60.21505376344090
26		92.45283018867920	7.547169811320760	45.71428571428570	66.0493827160494	66.25766871165640	0.697419354838710	0.857142857142857	0.54	46.0	85.48387096774190	14.51612903225810	54.0
27		93.47826086956520	6.521739130434780	48.27586206896550	65.71428571428570	64.70588235294120	0.661552583267768	0.714285714285714	0.591397849462366	40.86021505376340	73.17073170731710	26.82926829268290	59.13978494623660
28		91.11111111111110	8.888888888888889	37.14285714285710	67.05882352941180	68.18181818181820	0.697552447552447	0.833333333333333	0.576923076923077	42.30769230769230	81.81818181818180	18.18181818181820	57.69230769230770
29		95.1219512195122	4.878048780487810	40.0	71.50837988826820	73.29842931937170	0.718329525260218	0.777777777777778	0.693069306930693	30.69306930693070	74.35897435897440	25.64102564102560	69.3069306930693
30		95.55555555555560	4.444444444444450	33.33333333333330	74.28571428571430	72.39263803680980	0.745424836601307	0.808219178082192	0.655555555555556	34.44444444444440	83.52941176470590	16.47058823529410	65.55555555555560
31		91.30434782608700	8.695652173913040	37.03703703703700	63.84180790960450	65.95744680851060	0.641168831168831	0.704545454545455	0.62	38.0	66.23376623376620	33.76623376623380	62.0
32		89.58333333333330	10.41666666666670	39.28571428571430	67.24137931034480	65.45454545454550	0.675	0.72	0.6	40.0	75.0	25.0	60.0
33		95.83333333333330	4.166666666666670	47.82608695652170	67.59776536312850	67.41573033707870	0.686075949367089	0.769230769230769	0.6	40.0	77.21518987341770	22.78481012658230	60.0
34		95.0	5.0	42.85714285714290	70.87912087912090	70.39106145251400	0.721323529411765	0.818181818181818	0.617647058823529	38.23529411764710	82.5	17.5	61.76470588235290
35		93.61702127659580	6.382978723404260	45.83333333333330	64.80446927374300	62.72189349112430	0.651225306326582	0.697368421052632	0.569892473118280	43.01075268817200	73.25581395348840	26.74418604651160	56.98924731182800
36		94.11764705882350	5.88235294117647	30.76923076923080	65.89595375722540	66.66666666666670	0.671712328767123	0.766233766233766	0.59	41.0	75.34246575342470	24.65753424657530	59.0
37		91.11111111111110	8.888888888888889	19.04761904761900	69.56521739130430	68.88888888888890	0.702857142857143	0.775	0.62	38.0	78.57142857142860	21.42857142857140	62.0
38		91.11111111111110	8.888888888888889	42.85714285714290	68.92655367231640	68.57142857142860	0.696778350515464	0.769230769230769	0.618556701030928	38.14432989690720	77.5	22.5	61.85567010309280
39		92.6829268292683	7.317073170731710	28.125	68.36158192090400	68.53932584269660	0.710271060324209	0.847222222222222	0.575471698113208	42.45283018867920	84.50704225352110	15.49295774647890	57.54716981132080
40		81.08108108108110	18.91891891891890	41.37931034482760	73.36956521739130	72.31638418079100	0.737942884227989	0.8	0.659793814432990	34.02061855670100	81.60919540229890	18.39080459770120	65.97938144329900
41		97.5	2.5	40.0	71.35135135135140	71.95767195767200	0.718875502008032	0.781609195402299	0.666666666666667	33.33333333333330	77.10843373493980	22.89156626506020	66.66666666666670
42		94.64285714285710	5.357142857142860	26.31578947368420	68.0	67.44186046511630	0.680493859419911	0.698795180722892	0.651685393258427	34.83146067415730	70.93023255813950	29.06976744186050	65.1685393258427
43		90.0	10.0	46.42857142857140	67.58241758241760	66.28571428571430	0.675290135396518	0.666666666666667	0.659090909090909	34.09090909090910	69.14893617021280	30.85106382978720	65.9090909090909
44		97.82608695652170	2.173913043478260	56.66666666666670	60.91954022988510	62.22222222222220	0.621732026143791	0.717948717948718	0.549019607843137	45.09803921568630	69.44444444444440	30.55555555555560	54.90196078431370
45		94.11764705882350	5.88235294117647	37.03703703703700	73.83720930232560	71.3375796178344	0.744037940379404	0.835820895522388	0.622222222222222	37.77777777777780	86.58536585365850	13.41463414634150	62.22222222222220
46		92.3076923076923	7.692307692307690	42.85714285714290	72.94117647058820	71.95121951219510	0.741461366181411	0.842857142857143	0.627659574468085	37.23404255319150	85.52631578947370	14.47368421052630	62.76595744680850
47		97.2972972972973	2.702702702702700	33.33333333333330	70.49180327868850	70.65217391304350	0.708103241296519	0.755813953488372	0.663265306122449	33.6734693877551	75.29411764705880	24.70588235294120	66.3265306122449
48		93.02325581395350	6.976744186046510	36.66666666666670	66.10169491525420	68.42105263157900	0.672800956683497	0.773809523809524	0.613207547169811	38.67924528301890	73.23943661971830	26.76056338028170	61.32075471698110
49		86.95652173913040	13.04347826086960	44.44444444444440	70.62146892655370	68.67469879518070	0.708535650396116	0.76	0.626373626373626	37.36263736263740	79.06976744186050	20.93023255813950	62.63736263736260
50		88.63636363636360	11.36363636363640	43.33333333333330	72.1590909090909	71.34502923976610	0.732154508678063	0.824324324324324	0.628865979381443	37.11340206185570	83.54430379746840	16.45569620253160	62.88659793814430
51		91.30434782608700	8.695652173913040	39.39393939393940	71.34502923976610	71.00591715976330	0.721746338767615	0.8	0.638297872340426	36.17021276595750	80.51948051948050	19.48051948051950	63.82978723404260
52		97.87234042553190	2.127659574468090	39.39393939393940	70.0	67.92452830188680	0.700858250276855	0.739726027397260	0.627906976744186	37.2093023255814	77.38095238095240	22.61904761904760	62.7906976744186
53		93.02325581395350	6.976744186046510	48.0	73.07692307692310	72.31638418079100	0.732470986460348	0.771084337349398	0.680851063829787	31.91489361702130	78.4090909090909	21.5909090909091	68.08510638297870
54		95.0	5.0	33.33333333333330	69.49152542372880	67.46987951807230	0.707409793814433	0.811594202898551	0.577319587628866	42.2680412371134	83.75	16.25	57.73195876288660
55		95.74468085106380	4.25531914893617	36.66666666666670	68.78612716763010	66.25	0.697446236559140	0.791044776119403	0.569892473118280	43.01075268817200	82.5	17.5	56.98924731182800
56		97.5	2.5	40.625	69.10112359550560	65.83850931677020	0.692171717171717	0.746478873239437	0.588888888888889	41.11111111111110	79.54545454545460	20.45454545454550	58.88888888888890
57		92.85714285714290	7.142857142857140	57.14285714285710	70.05347593582890	69.89247311827960	0.703282828282828	0.747126436781609	0.656565656565657	34.34343434343430	75.0	25.0	65.65656565656570
58		92.85714285714290	7.142857142857140	46.15384615384620	68.68131868131870	69.1891891891892	0.687984496124031	0.719101123595506	0.666666666666667	33.33333333333330	70.93023255813950	29.06976744186050	66.66666666666670
59		95.74468085106380	4.25531914893617	50.0	73.48066298342540	75.2577319587629	0.738984316654220	0.802197802197802	0.708737864077670	29.12621359223300	76.92307692307690	23.07692307692310	70.87378640776700
60		86.36363636363640	13.63636363636360	46.42857142857140	61.79775280898880	60.0	0.622467071935157	0.671052631578947	0.542553191489362	45.74468085106380	70.23809523809520	29.76190476190480	54.25531914893620
61		92.0	8.0	41.66666666666670	63.63636363636360	61.90476190476190	0.652236652236652	0.753623188405797	0.525252525252525	47.47474747474750	77.92207792207790	22.07792207792210	52.52525252525250
62		87.17948717948720	12.82051282051280	36.66666666666670	68.50828729281770	70.15706806282720	0.689190866219906	0.752808988764045	0.656862745098039	34.31372549019610	72.15189873417720	27.84810126582280	65.68627450980390
63		91.66666666666670	8.333333333333330	34.48275862068970	71.09826589595380	69.87951807228910	0.716451959205583	0.783783783783784	0.630434782608696	36.95652173913040	80.24691358024690	19.75308641975310	63.04347826086960
64		94.73684210526320	5.263157894736840	36.0	68.44919786096260	66.28571428571430	0.698220064724919	0.805555555555556	0.563106796116505	43.68932038834950	83.33333333333330	16.66666666666670	56.31067961165050
65		89.74358974358980	10.25641025641030	46.875	71.50837988826820	72.43243243243240	0.722708809342473	0.797619047619048	0.663366336633663	33.66336633663370	78.2051282051282	21.79487179487180	66.33663366336630
66		93.75	6.25	42.42424242424240	69.8224852071006	71.8232044198895	0.720192307692308	0.844155844155844	0.625	37.5	81.53846153846150	18.46153846153850	62.5
67		93.02325581395350	6.976744186046510	40.74074074074070	71.11111111111110	72.63157894736840	0.710216718266254	0.726315789473684	0.726315789473684	27.36842105263160	69.41176470588240	30.58823529411770	72.63157894736840
68		93.87755102040820	6.122448979591840	62.06896551724140	67.44186046511630	66.66666666666670	0.678062678062678	0.727272727272727	0.615384615384615	38.46153846153850	74.07407407407410	25.92592592592590	61.53846153846150
69		93.18181818181820	6.818181818181820	51.61290322580650	70.28571428571430	70.45454545454550	0.708992616033755	0.775	0.645833333333333	35.41666666666670	77.21518987341770	22.78481012658230	64.58333333333330
70		91.8918918918919	8.108108108108110	34.61538461538460	66.84491978609630	64.77272727272730	0.677527055031085	0.76	0.564356435643564	43.56435643564360	79.06976744186050	20.93023255813950	56.43564356435640
71		92.15686274509800	7.843137254901960	39.28571428571430	66.66666666666670	65.03067484662580	0.672458791208791	0.736111111111111	0.582417582417583	41.75824175824180	76.25	23.75	58.24175824175830
72		86.79245283018870	13.20754716981130	57.89473684210530	67.97752808988760	68.50828729281770	0.686549034650301	0.756097560975610	0.626262626262626	37.37373737373740	74.68354430379750	25.31645569620250	62.62626262626260
73		85.41666666666670	14.58333333333330	43.47826086956520	69.83240223463690	68.23529411764710	0.698064918851436	0.716049382716049	0.651685393258427	34.83146067415730	74.44444444444440	25.55555555555560	65.1685393258427
74		91.66666666666670	8.333333333333330	37.5	71.42857142857140	71.42857142857140	0.721341463414634	0.792682926829268	0.65	35.0	79.26829268292680	20.73170731707320	65.0
75		97.22222222222220	2.777777777777780	34.61538461538460	75.0	74.8663101604278	0.755927952576379	0.823529411764706	0.686274509803922	31.37254901960780	82.55813953488370	17.44186046511630	68.62745098039220
76		95.91836734693880	4.081632653061230	42.30769230769230	73.14285714285710	72.51461988304090	0.737194641449961	0.805194805194805	0.659574468085106	34.04255319148940	81.48148148148150	18.51851851851850	65.95744680851060
77		88.88888888888890	11.11111111111110	33.33333333333330	71.91011235955060	70.58823529411760	0.727134146341463	0.810810810810811	0.625	37.5	82.92682926829270	17.07317073170730	62.5
78		94.28571428571430	5.714285714285710	37.03703703703700	69.68085106382980	66.27218934911240	0.696808510638298	0.746666666666667	0.595744680851064	40.42553191489360	79.7872340425532	20.21276595744680	59.57446808510640
79		95.55555555555560	4.444444444444450	32.0	65.55555555555560	62.65060240963860	0.660316674913409	0.722222222222222	0.553191489361702	44.68085106382980	76.74418604651160	23.25581395348840	55.31914893617020
80		97.87234042553190	2.127659574468090	32.0	75.84269662921350	73.93939393939400	0.773724489795919	0.910447761194030	0.622448979591837	37.75510204081630	92.5	7.5	62.24489795918370
81		93.47826086956520	6.521739130434780	42.10526315789470	63.24324324324320	61.79775280898880	0.633918128654971	0.662650602409639	0.578947368421053	42.10526315789470	68.88888888888890	31.11111111111110	57.89473684210530
82		92.5	7.5	50.0	70.1086956521739	67.06586826347310	0.706439393939394	0.788732394366197	0.583333333333333	41.66666666666670	82.95454545454550	17.04545454545450	58.33333333333330
83		91.30434782608700	8.695652173913040	38.70967741935480	67.05202312138730	66.66666666666670	0.675201612903226	0.730769230769231	0.612903225806452	38.70967741935480	73.75	26.25	61.29032258064520
84		91.30434782608700	8.695652173913040	51.85185185185190	67.79661016949150	68.15642458100560	0.683182989690722	0.743902439024390	0.628865979381443	37.11340206185570	73.75	26.25	62.88659793814430
85		93.18181818181820	6.818181818181820	48.27586206896550	70.05649717514130	69.364161849711	0.703533026113671	0.75	0.645161290322581	35.48387096774190	76.19047619047620	23.80952380952380	64.51612903225810
86		97.72727272727270	2.272727272727270	42.85714285714290	67.97752808988760	66.66666666666670	0.681875631951466	0.721518987341772	0.619565217391304	38.04347826086960	74.4186046511628	25.58139534883720	61.95652173913040
87		86.66666666666670	13.33333333333330	53.33333333333330	68.57142857142860	68.92655367231640	0.694341372912802	0.772151898734177	0.622448979591837	37.75510204081630	76.62337662337660	23.37662337662340	62.24489795918370
88		93.75	6.25	47.82608695652170	64.80446927374300	64.40677966101690	0.67007722007722	0.791666666666667	0.542857142857143	45.71428571428570	79.72972972972970	20.27027027027030	54.28571428571430
89		88.88888888888890	11.11111111111110	43.47826086956520	70.15706806282720	70.46632124352330	0.707530454042082	0.772727272727273	0.647619047619048	35.23809523809520	76.74418604651160	23.25581395348840	64.76190476190480
90		89.74358974358980	10.25641025641030	45.0	62.82722513089010	64.67661691542290	0.633530991273215	0.706521739130435	0.596330275229358	40.36697247706420	67.07317073170730	32.92682926829270	59.63302752293580
91		86.8421052631579	13.15789473684210	43.33333333333330	73.07692307692310	71.67630057803470	0.734361766485179	0.794871794871795	0.652631578947369	34.73684210526320	81.60919540229890	18.39080459770120	65.26315789473690
92		91.83673469387760	8.16326530612245	34.61538461538460	70.28571428571430	67.90123456790120	0.712306277909115	0.808823529411765	0.585106382978723	41.48936170212770	83.9506172839506	16.04938271604940	58.51063829787230
93		97.22222222222220	2.777777777777780	40.0	70.65217391304350	68.96551724137930	0.710227272727273	0.769230769230769	0.625	37.5	79.54545454545460	20.45454545454550	62.5
94		91.48936170212770	8.51063829787234	46.66666666666670	71.67630057803470	70.65868263473050	0.718365436062065	0.756410256410256	0.662921348314607	33.70786516853930	77.38095238095240	22.61904761904760	66.29213483146070
95		91.66666666666670	8.333333333333330	34.48275862068970	70.8108108108108	69.31818181818180	0.720706354831089	0.824324324324324	0.598039215686275	40.19607843137260	84.33734939759040	15.66265060240960	59.80392156862750
96		97.67441860465120	2.325581395348840	33.33333333333330	70.55555555555560	70.39106145251400	0.721409658302862	0.828947368421053	0.611650485436893	38.83495145631070	83.11688311688310	16.88311688311690	61.16504854368930
97		80.95238095238100	19.04761904761900	34.61538461538460	79.12087912087910	80.41237113402060	0.8	0.876404494382023	0.742857142857143	25.71428571428570	85.71428571428570	14.28571428571430	74.28571428571430
98		90.19607843137260	9.803921568627450	33.33333333333330	68.63905325443790	69.71428571428570	0.691134751773050	0.753086419753086	0.648936170212766	35.1063829787234	73.33333333333330	26.66666666666670	64.8936170212766
99		95.74468085106380	4.25531914893617	44.0	69.10112359550560	67.45562130177520	0.693503538928210	0.740259740259740	0.619565217391304	38.04347826086960	76.74418604651160	23.25581395348840	61.95652173913040
	**Mean**	**92.25703070721270**	**7.74296929278727**	**41.052722001394**	**69.50568200738710**	**68.92167000765400**	**0.702543760357795**	**0.774514049856371**	**0.622914404832418**	**37.7085595167583**	**78.2173115883172**	**21.7826884116828**	**62.2914404832418**
	**Standard Deviation**	3.47312914818426	3.47312914818426	7.61807872952965	3.18650849777672	3.34064486755615	0.031546048287847	0.046647983501081	0.042461087024595	4.24610870245951	5.07614064185606	5.07614064185606	4.24610870245951
	**T Test Significance (Female to Male)**	**0.000000000000000**	**0.000000000000000**	**0.000000000000000**									

Experiment 3.1.3 (Unbalanced training data, with feature selection) - Support Vector Classifier

		UB_Accuracy	UB_FScore	UB_ROC_AUC	UB_Precision	UB_Recall	UB_F_Accuracy	UB_F_FScore	UB_F_ROC_AUC	UB_F_Precision	UB_F_Recall	UB_F_FNR
0		73.2	67.6328502415459	0.743965074473549	0.933333333333333	0.530303030303030	73.17073170731710	57.69230769230770	0.702702702702703	1.0	0.405405405405405	59.45945945945950
1		72.40000000000000	66.98564593301440	0.720478995901639	0.804597701149425	0.573770491803279	74.28571428571430	65.38461538461540	0.726151315789474	0.85	0.53125	46.875
2		68.0	63.96396396396400	0.697177184936374	0.835294117647059	0.518248175182482	65.51724137931040	47.36842105263160	0.655172413793103	1.0	0.310344827586207	68.96551724137930
3		68.0	63.30275229357800	0.698727472385505	0.851851851851852	0.503649635036496	71.42857142857140	41.17647058823530	0.613224637681160	0.7	0.291666666666667	70.83333333333330
4		69.20000000000000	61.69154228855720	0.692000000000000	0.815789473684211	0.496	70.1492537313433	52.38095238095240	0.669819819819820	0.916666666666667	0.366666666666667	63.33333333333330
5		66.4	59.22330097087380	0.670318406047793	0.792207792207792	0.472868217054264	69.86301369863010	45.00000000000000	0.632445141065831	0.818181818181818	0.310344827586207	68.96551724137930
6		70.0	64.45497630331750	0.701292882744496	0.8	0.539682539682540	71.42857142857140	50.0	0.655680224403927	0.846153846153846	0.354838709677419	64.51612903225810
7		64.8	57.28155339805830	0.652599897540984	0.756410256410256	0.4609375	57.97101449275360	38.29787234042550	0.553056027164686	0.5625	0.290322580645161	70.96774193548390
8		69.20000000000000	63.1578947368421	0.699679487179487	0.835443037974684	0.507692307692308	68.29268292682930	48.0	0.644927536231884	0.857142857142857	0.333333333333333	66.66666666666670
9		67.2	58.58585858585860	0.658097022156192	0.682352941176471	0.513274336283186	67.53246753246750	44.44444444444440	0.613475177304965	0.666666666666667	0.333333333333333	66.66666666666670
10		70.40000000000000	65.09433962264150	0.702828981054787	0.784090909090909	0.556451612903226	82.08955223880600	71.42857142857140	0.777616279069768	0.833333333333333	0.625	37.5
11		70.0	64.78873239436620	0.695976680120443	0.75	0.570247933884298	70.4225352112676	53.33333333333330	0.656146179401993	0.705882352941177	0.428571428571429	57.14285714285710
12		66.8	64.68085106382980	0.680027130030360	0.775510204081633	0.554744525547445	70.66666666666670	50.0	0.65	0.785714285714286	0.366666666666667	63.33333333333330
13		70.8	60.96256684491980	0.697511065494900	0.838235294117647	0.478991596638656	73.80952380952380	54.16666666666670	0.674278846153846	0.8125	0.40625	59.375
14		71.6	66.97674418604650	0.718423916522630	0.818181818181818	0.566929133858268	76.11940298507460	52.94117647058820	0.672924901185771	0.818181818181818	0.391304347826087	60.86956521739130
15		69.6	62.74509803921570	0.697516641065028	0.820512820512821	0.507936507936508	66.26506024096390	46.15384615384620	0.629553466509988	0.8	0.324324324324324	67.56756756756760
16		66.8	61.75115207373270	0.675893258066586	0.779069767441861	0.511450381679389	67.53246753246750	50.98039215686280	0.65	0.8125	0.371428571428571	62.85714285714290
17		70.40000000000000	61.45833333333330	0.704	0.880597014925373	0.472	76.05633802816900	54.05405405405410	0.681196581196581	0.909090909090909	0.384615384615385	61.53846153846150
18		73.6	67.96116504854370	0.734639016897081	0.853658536585366	0.564516129032258	75.36231884057970	51.42857142857140	0.665277777777778	0.818181818181818	0.375	62.5
19		68.8	64.54545454545450	0.695362114311373	0.797752808988764	0.541984732824428	75.0	63.41463414634150	0.720588235294118	0.866666666666667	0.5	50.0
20		72.40000000000000	68.4931506849315	0.736747297992795	0.882352941176471	0.559701492537313	77.19298245614030	64.86486486486490	0.74	1.0	0.48	52.0
21		71.2	65.0485436893204	0.704352850539291	0.761363636363636	0.567796610169492	71.83098591549300	47.36842105263160	0.634308510638298	0.642857142857143	0.375	62.5
22		69.20000000000000	65.15837104072400	0.696425139342687	0.782608695652174	0.558139534883721	76.19047619047620	63.41463414634150	0.726851851851852	0.928571428571429	0.481481481481481	51.85185185185190
23		69.20000000000000	63.50710900473930	0.694673836502145	0.797619047619048	0.527559055118110	75.67567567567570	62.5	0.715909090909091	0.833333333333333	0.5	50.0
24		72.40000000000000	67.6056338028169	0.722862263184844	0.808988764044944	0.580645161290323	73.4375	56.41025641025640	0.690190190190190	0.916666666666667	0.407407407407407	59.25925925925930
25		70.40000000000000	61.85567010309280	0.689628482972136	0.75	0.526315789473684	70.0	40.0	0.611888111888112	0.777777777777778	0.269230769230769	73.07692307692310
26		66.0	61.88340807174890	0.662973872950820	0.726315789473684	0.5390625	69.35483870967740	48.64864864864860	0.652380952380952	0.9	0.333333333333333	66.66666666666670
27		73.6	67.0	0.724006707946337	0.779069767441861	0.587719298245614	71.60493827160490	43.90243902439030	0.620370370370370	0.642857142857143	0.333333333333333	66.66666666666670
28		69.6	62.0	0.692977402214967	0.805194805194805	0.504065040650407	71.42857142857140	44.44444444444440	0.636520241171404	0.888888888888889	0.296296296296296	70.37037037037040
29		65.60000000000000	59.04761904761910	0.658728634530440	0.746987951807229	0.488188976377953	72.94117647058820	56.60377358490570	0.679195804195804	0.75	0.454545454545455	54.54545454545450
30		70.40000000000000	62.24489795918370	0.704	0.859154929577465	0.488	71.7948717948718	52.17391304347830	0.661633493479753	0.8	0.387096774193548	61.29032258064520
31		74.8	71.74887892376680	0.756420133538778	0.879120879120879	0.606060606060606	76.05633802816900	62.22222222222220	0.721138211382114	0.933333333333333	0.466666666666667	53.33333333333330
32		72.0	66.34615384615380	0.724449282786885	0.8625	0.5390625	68.33333333333330	51.28205128205130	0.648190045248869	0.769230769230769	0.384615384615385	61.53846153846150
33		71.6	66.02870813397130	0.721987315010571	0.8625	0.534883720930233	70.3125	42.42424242424240	0.627179487179487	0.875	0.28	72.0
34		72.40000000000000	66.66666666666670	0.720286885245902	0.811764705882353	0.565573770491803	77.61194029850750	61.53846153846150	0.722222222222222	1.0	0.444444444444444	55.55555555555560
35		70.0	62.31155778894470	0.707252226279710	0.885714285714286	0.480620155038760	75.67567567567570	50.0	0.659591836734694	0.818181818181818	0.36	64.0
36		68.8	61.0	0.691376992510083	0.835616438356164	0.480314960629921	71.25	48.88888888888890	0.651041666666667	0.846153846153846	0.34375	65.625
37		72.8	67.9245283018868	0.744927536231884	0.935064935064935	0.533333333333333	69.1358024691358	44.44444444444450	0.636420525657071	0.909090909090909	0.294117647058824	70.58823529411770
38		70.40000000000000	62.24489795918370	0.695233818718327	0.792207792207792	0.512605042016807	71.21212121212120	51.28205128205130	0.659544159544160	0.833333333333333	0.370370370370370	62.96296296296300
39		74.0	68.89952153110050	0.736488217213115	0.827586206896552	0.590163934426230	77.94117647058820	61.53846153846150	0.716744186046512	0.857142857142857	0.48	52.0
40		70.0	66.0633484162896	0.705769230769231	0.802197802197802	0.561538461538462	67.64705882352940	47.61904761904760	0.620879120879121	0.625	0.384615384615385	61.53846153846150
41		74.0	70.31963470319640	0.742173996543115	0.836956521739131	0.606299212598425	72.36842105263160	57.14285714285720	0.7	1.0	0.4	60.0
42		68.8	64.22018348623850	0.689068100358423	0.760869565217391	0.555555555555556	72.22222222222220	56.52173913043480	0.680952380952381	0.8125	0.433333333333333	56.66666666666670
43		69.6	64.81481481481480	0.696	0.769230769230769	0.56	68.35443037974680	44.44444444444440	0.619623655913978	0.714285714285714	0.322580645161290	67.74193548387100
44		68.4	61.46341463414630	0.687055886306895	0.807692307692308	0.496062992125984	65.0	39.13043478260870	0.593810444874275	0.692307692307692	0.272727272727273	72.72727272727270
45		68.8	62.85714285714290	0.688000000000000	0.776470588235294	0.528	70.83333333333330	51.16279069767440	0.659523809523810	0.846153846153846	0.366666666666667	63.33333333333330
46		70.0	63.41463414634150	0.698604710701485	0.802469135802469	0.524193548387097	70.4225352112676	40.0	0.607391304347826	0.7	0.28	72.0
47		70.40000000000000	61.45833333333330	0.686629075118284	0.728395061728395	0.531531531531532	67.14285714285710	43.90243902439030	0.608527131782946	0.642857142857143	0.333333333333333	66.66666666666670
48		76.0	69.38775510204080	0.749244907139644	0.860759493670886	0.581196581196581	79.1044776119403	61.11111111111110	0.717538759689922	0.916666666666667	0.458333333333333	54.16666666666670
49		66.4	58.41584158415840	0.676874678993323	0.842857142857143	0.446969696969697	71.21212121212120	34.48275862068970	0.604166666666667	1.0	0.208333333333333	79.16666666666670
50		73.2	65.98984771573610	0.727330942622951	0.866666666666667	0.532786885245902	74.24242424242430	56.41025641025640	0.686538461538462	0.846153846153846	0.423076923076923	57.69230769230770
51		72.0	67.28971962616820	0.723872950819672	0.837209302325581	0.5625	79.66101694915250	53.84615384615390	0.682249322493225	0.875	0.388888888888889	61.11111111111110
52		72.0	65.68627450980390	0.710434894493052	0.761363636363636	0.577586206896552	73.13432835820900	47.05882352941180	0.643410852713178	0.8	0.333333333333333	66.66666666666670
53		68.8	62.85714285714290	0.703165208440556	0.868421052631579	0.492537313432836	70.66666666666670	45.00000000000000	0.638888888888889	0.9	0.3	70.0
54		64.8	54.63917525773200	0.653752561475410	0.803030303030303	0.4140625	75.36231884057970	45.16129032258070	0.641304347826087	0.875	0.304347826086957	69.56521739130430
55		68.0	59.59595959595960	0.675397028688525	0.776315789473684	0.483606557377049	72.72727272727270	35.71428571428570	0.589025755879059	0.555555555555556	0.263157894736842	73.68421052631580
56		70.8	65.40284360189570	0.712154200819672	0.831325301204819	0.5390625	72.22222222222220	47.36842105263160	0.649350649350649	0.9	0.321428571428571	67.85714285714290
57		68.4	59.06735751295340	0.678919057377049	0.802816901408451	0.467213114754098	76.0	47.05882352941180	0.647058823529412	0.8	0.333333333333333	66.66666666666670
58		72.40000000000000	68.49315068493150	0.730128205128205	0.842696629213483	0.576923076923077	73.61111111111110	45.71428571428570	0.642976588628763	0.888888888888889	0.307692307692308	69.23076923076920
59		70.0	64.11483253588520	0.696465163934426	0.770114942528736	0.549180327868853	72.22222222222220	44.44444444444440	0.628085106382979	0.727272727272727	0.32	68.0
60		70.8	67.55555555555560	0.722394220846233	0.853932584269663	0.558823529411765	77.04918032786890	65.0	0.735714285714286	0.928571428571429	0.5	50.0
61		73.2	66.99507389162560	0.732000000000000	0.871794871794872	0.544	75.36231884057970	54.05405405405410	0.680679785330948	0.909090909090909	0.384615384615385	61.53846153846150
62		68.4	57.75401069518720	0.668115942028986	0.75	0.469565217391304	72.22222222222220	44.44444444444440	0.632107023411371	0.8	0.307692307692308	69.23076923076920
63		67.2	58.16326530612250	0.667200307377049	0.770270270270270	0.467213114754098	66.66666666666670	38.09523809523810	0.591666666666667	0.666666666666667	0.266666666666667	73.33333333333330
64		72.40000000000000	65.67164179104480	0.722478238607271	0.857142857142857	0.532258064516129	78.37837837837840	55.55555555555560	0.689795918367347	0.909090909090909	0.4	60.0
65		70.40000000000000	67.2566371681416	0.714616572310859	0.826086956521739	0.567164179104478	74.24242424242430	56.41025641025640	0.683414634146342	0.785714285714286	0.44	56.00000000000000
66		69.6	64.48598130841120	0.710048893463716	0.8625	0.514925373134328	72.22222222222220	44.44444444444440	0.637037037037037	0.888888888888889	0.296296296296296	70.37037037037040
67		72.40000000000000	68.20276497695850	0.718494955337061	0.74	0.632478632478633	76.81159420289860	57.89473684210530	0.695833333333333	0.785714285714286	0.458333333333333	54.16666666666670
68		70.40000000000000	65.09433962264150	0.699852649112691	0.758241758241758	0.570247933884298	70.58823529411770	47.36842105263160	0.637362637362637	0.75	0.346153846153846	65.38461538461540
69		67.2	61.32075471698110	0.690724974200207	0.855263157894737	0.477941176470588	72.22222222222220	56.52173913043480	0.680952380952381	0.8125	0.433333333333333	56.66666666666670
70		72.40000000000000	64.24870466321240	0.713469440164355	0.826666666666667	0.525423728813559	74.66666666666670	55.81395348837210	0.693548387096774	1.0	0.387096774193548	61.29032258064520
71		68.8	64.54545454545450	0.695362114311373	0.797752808988764	0.541984732824428	72.58064516129030	54.05405405405410	0.685185185185185	1.0	0.370370370370370	62.96296296296300
72		69.20000000000000	64.51612903225810	0.701592193117617	0.823529411764706	0.530303030303030	69.86301369863010	54.16666666666670	0.671969696969697	0.866666666666667	0.393939393939394	60.60606060606060
73		68.4	64.57399103139010	0.70082681997287	0.837209302325581	0.525547445255475	65.15151515151520	41.02564102564100	0.596866096866097	0.666666666666667	0.296296296296296	70.37037037037040
74		68.0	61.90476190476190	0.681323604710702	0.773809523809524	0.515873015873016	69.11764705882350	48.78048780487810	0.636404697380307	0.714285714285714	0.370370370370370	62.96296296296300
75		76.8	69.14893617021280	0.750323498964803	0.855263157894737	0.580357142857143	82.35294117647060	50.0	0.668269230769231	0.75	0.375	62.5
76		65.60000000000000	54.73684210526320	0.652326995710902	0.776119402985075	0.422764227642276	67.56756756756760	42.85714285714290	0.621905476369092	0.818181818181818	0.290322580645161	70.96774193548390
77		70.40000000000000	63.72549019607840	0.699987192622951	0.792682926829268	0.532786885245902	69.56521739130430	48.78048780487800	0.653846153846154	0.909090909090909	0.333333333333333	66.66666666666670
78		72.40000000000000	66.66666666666670	0.728547643442623	0.873417721518987	0.5390625	72.3076923076923	55.00000000000000	0.677387914230020	0.846153846153846	0.407407407407407	59.25925925925930
79		74.0	70.31963470319630	0.751012145748988	0.895348837209302	0.578947368421053	72.3076923076923	50.0	0.655000000000000	0.818181818181818	0.36	64.0
80		70.0	63.41463414634150	0.701484895033282	0.822784810126582	0.515873015873016	66.21621621621620	48.97959183673470	0.633037694013304	0.75	0.363636363636364	63.63636363636360
81		68.4	62.91079812206570	0.694465844889574	0.827160493827161	0.507575757575758	67.64705882352940	52.17391304347830	0.667532467532468	0.923076923076923	0.363636363636364	63.63636363636360
82		70.40000000000000	63.36633663366340	0.701107483515780	0.810126582278481	0.520325203252033	75.30864197530870	62.96296296296300	0.718085106382979	0.85	0.5	50.0
83		71.2	64.0	0.707607581967213	0.820512820512821	0.524590163934426	77.63157894736840	56.41025641025640	0.690588235294118	0.785714285714286	0.44	56.00000000000000
84		72.0	67.5925925925926	0.718958013312852	0.793478260869565	0.588709677419355	70.0	57.14285714285710	0.674524400330852	0.777777777777778	0.451612903225806	54.83870967741940
85		71.2	62.88659793814430	0.702867406504587	0.813333333333333	0.512605042016807	70.12987012987010	51.06382978723400	0.659090909090909	0.857142857142857	0.363636363636364	63.63636363636360
86		70.8	61.37566137566140	0.702548668032787	0.865671641791045	0.475409836065574	71.42857142857140	33.33333333333330	0.587419056429232	0.714285714285714	0.217391304347826	78.26086956521740
87		73.2	64.9214659685864	0.716782765737874	0.805194805194805	0.543859649122807	74.28571428571430	55.00000000000000	0.684523809523810	0.916666666666667	0.392857142857143	60.71428571428570
88		68.8	61.38613861386140	0.685103386466936	0.784810126582278	0.504065040650407	63.88888888888890	38.09523809523810	0.592446892210858	0.727272727272727	0.258064516129032	74.19354838709680
89		72.40000000000000	66.00985221674880	0.725550435227855	0.870129870129870	0.531746031746032	72.5	50.0	0.657011191573404	0.846153846153846	0.354838709677419	64.51612903225810
90		69.20000000000000	63.15789473684210	0.696337090163934	0.814814814814815	0.515625	74.28571428571430	55.00000000000000	0.680447889750215	0.846153846153846	0.407407407407407	59.25925925925930
91		68.8	63.55140186915890	0.715584415584416	0.918918918918919	0.485714285714286	68.65671641791050	43.24324324324320	0.630036630036630	0.888888888888889	0.285714285714286	71.42857142857140
92		70.0	61.53846153846150	0.693670318406048	0.810810810810811	0.495867768595041	77.77777777777780	60.8695652173913	0.712533156498674	0.823529411764706	0.482758620689655	51.72413793103450
93		69.20000000000000	63.1578947368421	0.707475553268142	0.88	0.492537313432836	77.94117647058820	54.54545454545450	0.684541062801932	0.9	0.391304347826087	60.86956521739130
94		69.20000000000000	62.4390243902439	0.696721311475410	0.831168831168831	0.5	67.6056338028169	43.90243902439030	0.619458128078818	0.75	0.310344827586207	68.96551724137930
95		70.40000000000000	63.0	0.702444956477215	0.828947368421053	0.508064516129032	71.42857142857140	47.61904761904760	0.645390070921986	0.833333333333333	0.333333333333333	66.66666666666670
96		71.6	63.95939086294420	0.707453973955995	0.807692307692308	0.529411764705882	77.10843373493980	48.64864864864870	0.654137931034483	0.75	0.36	64.0
97		68.4	63.92694063926940	0.691545320418244	0.795454545454545	0.534351145038168	75.0	50.0	0.658723404255319	0.818181818181818	0.36	64.0
98		73.2	66.99507389162560	0.732000000000000	0.871794871794872	0.544	74.24242424242430	58.53658536585370	0.701127819548872	0.923076923076923	0.428571428571429	57.14285714285710
99		70.8	63.681592039801	0.706477214541731	0.831168831168831	0.516129032258065	71.23287671232880	43.24324324324320	0.626409017713366	0.8	0.296296296296296	70.37037037037040
	**Mean**	**70.24**	**63.9996988776347**	**0.703802399594020**	**0.817454864251935**	**0.527724238733291**	**72.2166300157096**	**50.6593048895012**	**0.659188717062238**	**0.825261441989383**	**0.369761707276093**	**63.0238292723907**
	**Standard Deviation**	2.35787954730431	3.2386286312348	0.022543011323680	0.046078200365584	0.039812290940609	4.02925651098054	7.55770677778197	0.039600863196027	0.098026363663767	0.072244869719648	7.22448697196481
	**T Test Significance (Female to Male)**						**0.013090291699709**	**0.000000000000000**	**0.000000000056323**	**0.90443348846576**	**0.000000000000000**	**0.000000000000000**

		UB_F_TNR	UB_F_FPR	UB_F_TPR	UB_M_Accuracy	UB_M_FScore	UB_M_ROC_AUC	UB_M_Precision	UB_M_Recall	UB_M_FNR	UB_M_TNR	UB_M_FPR	UB_M_TPR
0		100.0	0.0	40.54054054054050	73.21428571428570	70.96774193548390	0.755227108868061	0.916666666666667	0.578947368421053	42.10526315789470	93.15068493150690	6.849315068493150	57.89473684210530
1		92.10526315789470	7.894736842105260	53.125	71.66666666666670	67.51592356687900	0.716666666666667	0.791044776119403	0.588888888888889	41.11111111111110	84.44444444444440	15.55555555555560	58.88888888888890
2		100.0	0.0	31.03448275862070	68.75	67.3913043478261	0.703703703703704	0.815789473684211	0.574074074074074	42.59259259259260	83.33333333333330	16.66666666666670	57.40740740740740
3		93.47826086956520	6.521739130434780	29.16666666666670	66.66666666666670	67.3913043478261	0.707172104081363	0.873239436619718	0.548672566371681	45.13274336283190	86.56716417910450	13.43283582089550	54.86725663716810
4		97.2972972972973	2.702702702702700	36.66666666666670	68.85245901639340	64.15094339622640	0.694557416267943	0.796875	0.536842105263158	46.31578947368420	85.22727272727270	14.77272727272730	53.68421052631580
5		95.45454545454550	4.545454545454550	31.03448275862070	64.97175141242940	62.65060240963860	0.669090909090909	0.787878787878788	0.52	48.0	81.81818181818180	18.18181818181820	52.0
6		95.65217391304350	4.347826086956520	35.48387096774190	69.36416184971100	68.26347305389220	0.703846153846154	0.791666666666667	0.6	40.0	80.76923076923080	19.23076923076920	60.0
7		81.57894736842110	18.42105263157890	29.03225806451610	67.40331491712710	62.89308176100630	0.686303387334315	0.806451612903226	0.515463917525773	48.45360824742270	85.71428571428570	14.28571428571430	51.54639175257730
8		95.65217391304350	4.347826086956520	33.33333333333330	69.64285714285710	67.92452830188680	0.712909718228867	0.830769230769231	0.574468085106383	42.5531914893617	85.13513513513510	14.86486486486490	57.44680851063830
9		89.36170212765960	10.63829787234040	33.33333333333330	67.05202312138730	62.74509803921570	0.666934404283802	0.685714285714286	0.578313253012048	42.16867469879520	75.55555555555560	24.44444444444440	57.83132530120480
10		93.02325581395350	6.976744186046510	62.5	66.12021857923500	63.52941176470590	0.673614457831325	0.771428571428572	0.54	46.0	80.72289156626510	19.27710843373490	54.0
11		88.37209302325580	11.62790697674420	42.85714285714290	69.83240223463690	67.85714285714290	0.701800450112528	0.76	0.612903225806452	38.70967741935480	79.06976744186050	20.93023255813950	61.29032258064520
12		93.33333333333330	6.666666666666670	36.66666666666670	65.14285714285720	68.06282722513090	0.664032435404068	0.773809523809524	0.607476635514019	39.25233644859810	72.05882352941180	27.94117647058820	60.74766355140190
13		94.23076923076920	5.769230769230770	40.625	69.27710843373490	63.30935251798560	0.702240651825986	0.846153846153846	0.505747126436782	49.42528735632180	89.87341772151900	10.12658227848100	50.57471264367820
14		95.45454545454550	4.545454545454550	39.13043478260870	69.94535519125680	69.61325966850830	0.714277020447907	0.818181818181818	0.605769230769231	39.42307692307690	82.27848101265820	17.72151898734180	60.57692307692310
15		93.47826086956520	6.521739130434780	32.43243243243240	71.25748502994010	68.42105263157900	0.721622010947854	0.825396825396825	0.584269662921348	41.57303370786520	85.8974358974359	14.10256410256410	58.42696629213480
16		92.85714285714290	7.142857142857140	37.14285714285710	66.47398843930640	65.06024096385540	0.677353896103896	0.771428571428572	0.5625	43.75	79.22077922077920	20.77922077922080	56.25
17		97.77777777777780	2.222222222222220	38.46153846153850	68.15642458100560	63.22580645161290	0.703724747474747	0.875	0.494949494949495	50.50505050505050	91.25	8.75	49.49494949494950
18		95.55555555555560	4.444444444444450	37.5	72.92817679558010	71.34502923976610	0.743271604938272	0.859154929577465	0.61	39.0	87.65432098765430	12.34567901234570	61.0
19		94.11764705882350	5.88235294117647	50.0	66.84210526315790	64.80446927374300	0.682072829131653	0.783783783783784	0.552380952380952	44.76190476190480	81.17647058823530	18.82352941176470	55.23809523809520
20		100.0	0.0	48.0	70.98445595854920	69.23076923076920	0.729467016164264	0.863013698630137	0.577981651376147	42.20183486238530	88.09523809523810	11.90476190476190	57.79816513761470
21		89.36170212765960	10.63829787234040	37.5	70.94972067039110	69.04761904761910	0.714392991239049	0.783783783783784	0.617021276595745	38.29787234042550	81.17647058823530	18.82352941176470	61.70212765957450
22		97.22222222222220	2.777777777777780	48.14814814814810	66.84491978609630	65.55555555555560	0.677450980392157	0.756410256410256	0.578431372549020	42.15686274509800	77.64705882352940	22.35294117647060	57.84313725490200
23		93.18181818181820	6.818181818181820	50.0	66.47727272727270	63.80368098159510	0.679433642176693	0.787878787878788	0.536082474226804	46.39175257731960	82.27848101265820	17.72151898734180	53.60824742268040
24		97.2972972972973	2.702702702702700	40.74074074074070	72.04301075268820	70.11494252873560	0.724545349241283	0.792207792207792	0.628865979381443	37.11340206185570	82.02247191011240	17.97752808988760	62.88659793814430
25		95.45454545454550	4.545454545454550	26.92307692307690	70.55555555555560	66.66666666666670	0.703310276679842	0.746478873239437	0.602272727272727	39.77272727272730	80.43478260869570	19.56521739130430	60.22727272727270
26		97.14285714285710	2.857142857142860	33.33333333333330	64.8936170212766	64.51612903225810	0.653351542050757	0.705882352941177	0.594059405940594	40.5940594059406	71.26436781609200	28.73563218390800	59.4059405940594
27		90.74074074074080	9.25925925925926	33.33333333333330	74.55621301775150	72.95597484276730	0.747967479674797	0.805555555555556	0.666666666666667	33.33333333333330	82.92682926829270	17.07317073170730	66.66666666666670
28		97.67441860465120	2.325581395348840	29.62962962962960	68.88888888888890	65.85365853658540	0.697916666666667	0.794117647058824	0.5625	43.75	83.33333333333330	16.66666666666670	56.25
29		90.38461538461540	9.615384615384620	45.45454545454550	61.81818181818180	59.87261146496820	0.637323943661972	0.746031746031746	0.5	50.0	77.46478873239440	22.53521126760560	50.0
30		93.61702127659580	6.382978723404260	38.70967741935480	69.76744186046510	65.33333333333330	0.715766503000546	0.875	0.521276595744681	47.87234042553190	91.02564102564100	8.974358974358970	52.12765957446810
31		97.5609756097561	2.439024390243900	46.66666666666670	74.30167597765360	74.15730337078650	0.758594346829641	0.868421052631579	0.647058823529412	35.29411764705880	87.01298701298700	12.98701298701300	64.70588235294120
32		91.17647058823530	8.823529411764710	38.46153846153850	73.15789473684210	69.8224852071006	0.743761140819964	0.880597014925373	0.578431372549020	42.15686274509800	90.9090909090909	9.090909090909090	57.84313725490200
33		97.43589743589740	2.564102564102560	28.00000000000000	72.04301075268820	70.45454545454550	0.737101313320826	0.861111111111111	0.596153846153846	40.38461538461540	87.8048780487805	12.19512195121950	59.61538461538460
34		100.0	0.0	44.44444444444440	70.49180327868850	67.85714285714290	0.709090909090909	0.780821917808219	0.6	40.0	81.81818181818180	18.18181818181820	60.0
35		95.91836734693880	4.081632653061230	36.0	67.61363636363640	65.03067484662580	0.713141025641026	0.898305084745763	0.509615384615385	49.03846153846150	91.66666666666670	8.333333333333330	50.96153846153850
36		95.83333333333330	4.166666666666670	34.375	67.64705882352940	64.51612903225810	0.696491228070175	0.833333333333333	0.526315789473684	47.36842105263160	86.66666666666670	13.33333333333330	52.63157894736840
37		97.87234042553190	2.127659574468090	29.41176470588240	74.55621301775150	74.25149700598800	0.777518928363425	0.939393939393939	0.613861386138614	38.61386138613860	94.11764705882350	5.88235294117647	61.38613861386140
38		94.87179487179490	5.128205128205130	37.03703703703700	70.1086956521739	64.96815286624210	0.701086956521739	0.784615384615385	0.554347826086957	44.56521739130430	84.78260869565220	15.21739130434780	55.43478260869570
39		95.34883720930230	4.651162790697680	48.0	72.52747252747250	70.58823529411760	0.732807762280170	0.821917808219178	0.618556701030928	38.14432989690720	84.70588235294120	15.29411764705880	61.85567010309280
40		85.71428571428570	14.28571428571430	38.46153846153850	70.87912087912090	70.39106145251400	0.725961538461538	0.84	0.605769230769231	39.42307692307690	84.61538461538460	15.38461538461540	60.57692307692310
41		100.0	0.0	40.0	74.71264367816090	74.11764705882360	0.75092788971368	0.807692307692308	0.684782608695652	31.52173913043480	81.70731707317070	18.29268292682930	68.47826086956520
42		92.85714285714290	7.142857142857140	43.33333333333330	67.41573033707870	66.27906976744190	0.681021341463415	0.75	0.59375	40.625	76.82926829268290	23.17073170731710	59.375
43		91.66666666666670	8.333333333333330	32.25806451612900	70.17543859649120	70.17543859649120	0.708759325780602	0.779220779220779	0.638297872340426	36.17021276595750	77.92207792207790	22.07792207792210	63.82978723404260
44		91.48936170212770	8.51063829787234	27.27272727272730	70.0	67.92452830188680	0.714865621500560	0.830769230769231	0.574468085106383	42.5531914893617	85.52631578947370	14.47368421052630	57.44680851063830
45		95.23809523809520	4.761904761904760	36.66666666666670	67.97752808988760	65.86826347305390	0.687064045656309	0.763888888888889	0.578947368421053	42.10526315789470	79.51807228915660	20.48192771084340	57.89473684210530
46		93.47826086956520	6.521739130434780	28.00000000000000	69.83240223463690	68.23529411764710	0.711679292929293	0.816901408450704	0.585858585858586	41.41414141414140	83.75	16.25	58.58585858585860
47		88.37209302325580	11.62790697674420	33.33333333333330	71.66666666666670	66.2251655629139	0.709077380952381	0.746268656716418	0.595238095238095	40.47619047619050	82.29166666666670	17.70833333333330	59.52380952380950
48		97.67441860465120	2.325581395348840	45.83333333333330	74.86338797814210	71.25000000000000	0.750896057347670	0.850746268656716	0.612903225806452	38.70967741935480	88.88888888888890	11.11111111111110	61.29032258064520
49		100.0	0.0	20.83333333333330	64.67391304347830	62.42774566473990	0.677631578947369	0.830769230769231	0.5	50.0	85.52631578947370	14.47368421052630	50.0
50		95.0	5.0	42.30769230769230	72.82608695652170	68.35443037974680	0.735795454545455	0.870967741935484	0.5625	43.75	90.9090909090909	9.090909090909090	56.25
51		97.5609756097561	2.439024390243900	38.88888888888890	69.63350785340320	69.14893617021280	0.715207631874299	0.833333333333333	0.590909090909091	40.90909090909090	83.9506172839506	16.04938271604940	59.09090909090910
52		95.34883720930230	4.651162790697680	33.33333333333330	71.58469945355190	69.41176470588240	0.716256569517439	0.756410256410256	0.641304347826087	35.86956521739130	79.12087912087910	20.87912087912090	64.13043478260870
53		97.77777777777780	2.222222222222220	30.0	68.0	67.05882352941180	0.710658179848321	0.863636363636364	0.548076923076923	45.19230769230770	87.32394366197180	12.67605633802820	54.80769230769230
54		97.82608695652170	2.173913043478260	30.43478260869570	60.77348066298340	56.44171779141110	0.640100250626566	0.793103448275862	0.438095238095238	56.19047619047620	84.21052631578950	15.78947368421050	43.80952380952380
55		91.48936170212770	8.51063829787234	26.31578947368420	66.30434782608700	63.52941176470590	0.681889008749850	0.805970149253731	0.524271844660194	47.57281553398060	83.9506172839506	16.04938271604940	52.42718446601940
56		97.72727272727270	2.272727272727270	32.14285714285710	70.2247191011236	69.364161849711	0.716666666666667	0.821917808219178	0.6	40.0	83.33333333333330	16.66666666666670	60.0
57		96.07843137254900	3.921568627450980	33.33333333333330	65.14285714285720	61.63522012578620	0.672077922077922	0.803278688524590	0.5	50.0	84.4155844155844	15.58441558441560	50.0
58		97.82608695652170	2.173913043478260	30.76923076923080	71.91011235955060	72.82608695652170	0.734277546777547	0.8375	0.644230769230769	35.57692307692310	82.43243243243240	17.56756756756760	64.42307692307690
59		93.61702127659580	6.382978723404260	32.0	69.10112359550560	68.20809248554910	0.699185439735268	0.776315789473684	0.608247422680412	39.17525773195880	79.01234567901230	20.98765432098770	60.82474226804120
60		97.14285714285710	2.857142857142860	50.0	68.78306878306880	68.10810810810810	0.710414269275029	0.84	0.572727272727273	42.72727272727270	84.81012658227850	15.18987341772150	57.27272727272730
61		97.67441860465120	2.325581395348840	38.46153846153850	72.37569060773480	69.87951807228910	0.738051244148805	0.865671641791045	0.585858585858586	41.41414141414140	89.02439024390250	10.97560975609760	58.58585858585860
62		95.65217391304350	4.347826086956520	30.76923076923080	66.85393258426970	60.9271523178808	0.668539325842697	0.741935483870968	0.516853932584270	48.31460674157300	82.02247191011240	17.97752808988760	51.68539325842700
63		91.66666666666670	8.333333333333330	26.66666666666670	67.44186046511630	63.63636363636360	0.685054347826087	0.790322580645161	0.532608695652174	46.73913043478260	83.75	16.25	53.2608695652174
64		97.95918367346940	2.040816326530610	40.0	69.88636363636360	67.87878787878790	0.717893217893218	0.848484848484849	0.565656565656566	43.43434343434340	87.01298701298700	12.98701298701300	56.56565656565660
65		92.6829268292683	7.317073170731710	44.0	69.02173913043480	69.5187165775401	0.711498470948012	0.833333333333333	0.596330275229358	40.36697247706420	82.66666666666670	17.33333333333330	59.63302752293580
66		97.77777777777780	2.222222222222220	29.62962962962960	68.53932584269660	68.53932584269660	0.714624193760695	0.859154929577465	0.570093457943925	42.99065420560750	85.91549295774650	14.08450704225350	57.00934579439250
67		93.33333333333330	6.666666666666670	45.83333333333330	70.71823204419890	70.39106145251400	0.708027859237537	0.732558139534884	0.677419354838710	32.25806451612900	73.86363636363640	26.13636363636360	67.74193548387100
68		92.85714285714290	7.142857142857140	34.61538461538460	70.32967032967030	68.9655172413793	0.706594071385360	0.759493670886076	0.631578947368421	36.84210526315790	78.16091954022990	21.83908045977010	63.1578947368421
69		92.85714285714290	7.142857142857140	43.33333333333330	65.1685393258427	62.65060240963860	0.689727463312369	0.866666666666667	0.490566037735849	50.94339622641510	88.88888888888890	11.11111111111110	49.05660377358490
70		100.0	0.0	38.70967741935480	71.42857142857140	66.66666666666670	0.713492685475444	0.793650793650794	0.574712643678161	42.5287356321839	85.22727272727270	14.77272727272730	57.47126436781610
71		100.0	0.0	37.03703703703700	67.5531914893617	66.66666666666670	0.686126373626374	0.772151898734177	0.586538461538462	41.34615384615390	78.57142857142860	21.42857142857140	58.65384615384620
72		95.0	5.0	39.39393939393940	68.92655367231640	67.45562130177520	0.704545454545455	0.814285714285714	0.575757575757576	42.42424242424240	83.33333333333330	16.66666666666670	57.57575757575760
73		89.74358974358980	10.25641025641030	29.62962962962960	69.56521739130430	69.56521739130430	0.723341523341523	0.864864864864865	0.581818181818182	41.81818181818180	86.48648648648650	13.51351351351350	58.18181818181820
74		90.2439024390244	9.75609756097561	37.03703703703700	67.58241758241760	65.08875739644970	0.687416331994645	0.785714285714286	0.555555555555556	44.44444444444440	81.92771084337350	18.07228915662650	55.55555555555560
75		96.15384615384620	3.846153846153850	37.5	74.72527472527470	71.95121951219510	0.754966085271318	0.867647058823529	0.614583333333333	38.54166666666670	89.53488372093020	10.46511627906980	61.45833333333330
76		95.34883720930230	4.651162790697680	29.03225806451610	64.77272727272730	58.10810810810810	0.656314699792961	0.767857142857143	0.467391304347826	53.2608695652174	84.52380952380950	15.47619047619050	46.73913043478260
77		97.43589743589740	2.564102564102560	33.33333333333330	70.71823204419890	67.48466257668710	0.709025403028823	0.774647887323944	0.597826086956522	40.21739130434780	82.02247191011240	17.97752808988760	59.78260869565220
78		94.73684210526320	5.263157894736840	40.74074074074070	72.43243243243240	69.46107784431140	0.739509665252240	0.878787878787879	0.574257425742574	42.57425742574260	90.47619047619050	9.523809523809520	57.42574257425740
79		95.0	5.0	36.0	74.5945945945946	74.31693989071040	0.769360269360270	0.906666666666667	0.629629629629630	37.03703703703700	90.9090909090909	9.090909090909090	62.96296296296300
80		90.2439024390244	9.75609756097561	36.36363636363640	71.5909090909091	67.94871794871800	0.724705272703718	0.841269841269841	0.569892473118280	43.01075268817200	87.95180722891570	12.04819277108430	56.98924731182800
81		97.14285714285710	2.857142857142860	36.36363636363640	68.68131868131870	65.86826347305390	0.699464524765730	0.808823529411765	0.555555555555556	44.44444444444440	84.33734939759040	15.66265060240960	55.55555555555560
82		93.61702127659580	6.382978723404260	50.0	68.04733727810650	63.51351351351350	0.689044943820225	0.796610169491525	0.528089887640449	47.19101123595510	85.0	15.0	52.80898876404490
83		94.11764705882350	5.88235294117647	44.0	68.39080459770120	65.83850931677020	0.701767304860088	0.828125	0.546391752577320	45.36082474226800	85.71428571428570	14.28571428571430	54.63917525773200
84		89.74358974358980	10.25641025641030	45.16129032258060	72.77777777777780	70.65868263473050	0.730997404523545	0.797297297297297	0.634408602150538	36.55913978494620	82.75862068965520	17.24137931034480	63.44086021505380
85		95.45454545454550	4.545454545454550	36.36363636363640	71.67630057803470	66.66666666666670	0.715918203688853	0.803278688524590	0.569767441860465	43.02325581395350	86.20689655172410	13.79310344827590	56.97674418604650
86		95.74468085106380	4.25531914893617	21.73913043478260	70.55555555555560	66.66666666666670	0.724466891133558	0.883333333333333	0.535353535353535	46.46464646464650	91.35802469135800	8.641975308641980	53.53535353535350
87		97.61904761904760	2.380952380952380	39.28571428571430	72.77777777777780	67.5496688741722	0.722043542800594	0.784615384615385	0.593023255813954	40.69767441860470	85.1063829787234	14.89361702127660	59.30232558139540
88		92.6829268292683	7.317073170731710	25.80645161290320	70.78651685393260	67.5	0.712082912032356	0.794117647058824	0.586956521739131	41.30434782608700	83.72093023255820	16.27906976744190	58.69565217391310
89		95.91836734693880	4.081632653061230	35.48387096774190	72.35294117647060	70.44025157232700	0.741403508771930	0.875	0.589473684210526	41.05263157894740	89.33333333333330	10.66666666666670	58.94736842105260
90		95.34883720930230	4.651162790697680	40.74074074074070	67.22222222222220	65.08875739644970	0.689998746710114	0.808823529411765	0.544554455445545	45.54455445544560	83.54430379746840	16.45569620253160	54.45544554455450
91		97.43589743589740	2.564102564102560	28.57142857142860	68.85245901639340	67.79661016949150	0.732645875251509	0.923076923076923	0.535714285714286	46.42857142857140	92.95774647887320	7.042253521126760	53.57142857142860
92		94.23076923076920	5.769230769230770	48.27586206896550	66.27218934911240	61.74496644295300	0.678571428571429	0.807017543859649	0.5	50.0	85.71428571428570	14.28571428571430	50.0
93		97.77777777777780	2.222222222222220	39.13043478260870	65.93406593406590	64.77272727272730	0.700418728587743	0.876923076923077	0.513513513513514	48.64864864864870	88.73239436619720	11.26760563380280	51.35135135135140
94		92.85714285714290	7.142857142857140	31.03448275862070	69.83240223463690	67.07317073170730	0.715277777777778	0.846153846153846	0.555555555555556	44.44444444444440	87.5	12.5	55.55555555555560
95		95.74468085106380	4.25531914893617	33.33333333333330	69.94219653179190	67.0886075949367	0.712294640452464	0.828125	0.563829787234043	43.61702127659580	86.07594936708860	13.92405063291140	56.38297872340430
96		94.82758620689660	5.172413793103450	36.0	68.8622754491018	67.5	0.705042261731274	0.818181818181818	0.574468085106383	42.5531914893617	83.56164383561640	16.43835616438360	57.44680851063830
97		95.74468085106380	4.25531914893617	36.0	65.73033707865170	66.66666666666670	0.676624737945493	0.792207792207792	0.575471698113208	42.45283018867920	77.77777777777780	22.22222222222220	57.54716981132080
98		97.36842105263160	2.631578947368420	42.85714285714290	72.82608695652170	69.1358024691358	0.736935655883399	0.861538461538462	0.577319587628866	42.2680412371134	89.65517241379310	10.34482758620690	57.73195876288660
99		95.65217391304350	4.347826086956520	29.62962962962960	70.62146892655370	68.29268292682930	0.719909793814433	0.835820895522388	0.577319587628866	42.2680412371134	86.25	13.75000000000000	57.73195876288660
	**Mean**	**94.8615726848382**	**5.1384273151618**	**36.9761707276093**	**69.4663539229027**	**67.1566358389361**	**0.708150775327932**	**0.816582365619085**	**0.572732391654006**	**42.7267608345994**	**84.35691590018580**	**15.6430840998143**	**57.2732391654006**
	**Standard Deviation**	3.27680530843872	3.27680530843873	7.22448697196481	2.85337511705476	3.36047513530075	0.026733250835983	0.047651693650272	0.044665575152352	4.46655751523528	4.50350725237181	4.50350725237181	4.46655751523528
	**T Test Significance (Female to Male)**	**0.000000000000000**	**0.000000000000000**	**0.000000000000000**									

Experiment 3.1.3 (Unbalanced training data, with feature selection) - Gaussian Naive Bayes Classifier

		UB_Accuracy	UB_FScore	UB_ROC_AUC	UB_Precision	UB_Recall	UB_F_Accuracy	UB_F_FScore	UB_F_ROC_AUC	UB_F_Precision	UB_F_Recall
0		76.4	69.43005181347150	0.750563607085346	0.858974358974359	0.582608695652174	82.8125	64.51612903225810	0.740935672514620	0.833333333333333	0.526315789473684
1		67.60000000000000	61.61137440758290	0.707560674084196	0.928571428571429	0.460992907801418	76.92307692307690	54.54545454545450	0.683747412008282	0.9	0.391304347826087
2		74.8	67.6923076923077	0.741719520789288	0.891891891891892	0.545454545454545	83.33333333333330	62.5	0.727272727272727	1.0	0.454545454545455
3		68.8	62.13592233009710	0.688	0.790123456790123	0.512	63.41463414634150	40.0	0.595410628019324	0.714285714285714	0.277777777777778
4		70.40000000000000	62.24489795918370	0.69921875	0.824324324324324	0.5	74.64788732394370	57.14285714285720	0.7	1.0	0.4
5		71.2	66.35514018691590	0.718910256410256	0.845238095238095	0.546153846153846	69.84126984126980	48.64864864864860	0.652777777777778	0.9	0.333333333333333
6		74.0	64.08839779005530	0.721297729618163	0.865671641791045	0.508771929824561	75.34246575342470	55.00000000000000	0.681964573268921	0.846153846153846	0.407407407407407
7		75.2	70.47619047619050	0.752	0.870588235294118	0.592	81.42857142857140	64.86486486486490	0.739130434782609	0.923076923076923	0.5
8		69.20000000000000	63.84976525821600	0.710526315789474	0.883116883116883	0.5	78.37837837837840	66.66666666666670	0.75	1.0	0.5
9		68.4	60.69651741293530	0.697162300975860	0.884057971014493	0.462121212121212	73.01587301587300	54.05405405405410	0.678794178794179	0.909090909090909	0.384615384615385
10		66.8	62.78026905829600	0.688735498088016	0.833333333333333	0.503597122302158	69.35483870967740	45.71428571428570	0.648148148148148	1.0	0.296296296296296
11		68.4	58.20105820105820	0.682091653865848	0.846153846153846	0.443548387096774	73.4375	54.05405405405410	0.674358974358974	0.833333333333333	0.4
12		72.8	66.0	0.726446492575525	0.868421052631579	0.532258064516129	74.19354838709680	55.55555555555560	0.682017543859649	0.833333333333333	0.416666666666667
13		72.40000000000000	67.60563380281690	0.727971311475410	0.847058823529412	0.5625	74.28571428571430	47.05882352941180	0.644927536231884	0.8	0.333333333333333
14		71.2	67.85714285714290	0.730719461697723	0.883720930232558	0.550724637681159	72.22222222222220	50.00000000000000	0.660785886126704	0.909090909090909	0.344827586206897
15		70.8	65.07177033492820	0.725764895330113	0.918918918918919	0.503703703703704	69.86301369863010	47.61904761904760	0.65625	1.0	0.3125
16		74.4	68.62745098039210	0.741245758914282	0.864197530864198	0.569105691056911	76.92307692307690	57.14285714285710	0.693581780538302	0.833333333333333	0.434782608695652
17		71.6	66.66666666666670	0.717229902713774	0.816091954022989	0.563492063492064	71.25	54.90196078431370	0.683080808080808	0.933333333333333	0.388888888888889
18		70.0	63.41463414634150	0.706483439041579	0.855263157894737	0.503875968992248	75.80645161290320	54.54545454545460	0.679545454545455	0.818181818181818	0.409090909090909
19		71.2	66.35514018691590	0.715868340163934	0.825581395348837	0.5546875	79.66101694915250	66.66666666666670	0.746980676328503	0.923076923076923	0.521739130434783
20		70.40000000000000	60.21505376344090	0.700083221304654	0.888888888888889	0.455284552845528	67.12328767123290	40.0	0.617127496159754	0.888888888888889	0.258064516129032
21		70.8	65.07177033492820	0.725764895330113	0.918918918918919	0.503703703703704	69.11764705882350	48.78048780487810	0.646772767462423	0.833333333333333	0.344827586206897
22		74.8	69.26829268292680	0.748000000000000	0.8875	0.568	69.44444444444440	47.61904761904760	0.642857142857143	0.833333333333333	0.333333333333333
23		74.8	68.65671641791050	0.748	0.907894736842105	0.552	82.1917808219178	64.86486486486490	0.74	1.0	0.48
24		71.2	65.38461538461540	0.721726858682404	0.883116883116883	0.519083969465649	70.76923076923080	53.65853658536590	0.675766283524904	0.916666666666667	0.379310344827586
25		71.2	64.35643564356440	0.718880133256455	0.890410958904110	0.503875968992248	75.34246575342470	50.0	0.662438625204583	0.9	0.346153846153846
26		73.6	67.0	0.725360267627380	0.797619047619048	0.577586206896552	82.43243243243240	71.11111111111110	0.776442307692308	0.842105263157895	0.615384615384615
27		75.2	70.47619047619050	0.745377503852080	0.804347826086957	0.627118644067797	80.88235294117650	68.29268292682930	0.756744186046512	0.875	0.56
28		74.0	67.33668341708540	0.735527663934426	0.870129870129870	0.549180327868853	79.71014492753620	65.0	0.740740740740741	1.0	0.481481481481481
29		76.0	68.08510638297870	0.747188484030589	0.901408450704225	0.547008547008547	82.85714285714290	66.66666666666670	0.75	1.0	0.5
30		76.4	73.05936073059360	0.770192307692308	0.898876404494382	0.615384615384615	74.24242424242430	58.53658536585370	0.701127819548872	0.923076923076923	0.428571428571429
31		74.8	65.94594594594600	0.728012184846717	0.824324324324324	0.549549549549550	76.92307692307690	48.27586206896550	0.651601830663616	0.7	0.368421052631579
32		76.0	70.58823529411760	0.757249855963127	0.888888888888889	0.585365853658537	75.71428571428570	56.41025641025640	0.688811188811189	0.846153846153846	0.423076923076923
33		68.0	56.04395604395600	0.668484187568157	0.809523809523810	0.428571428571429	71.42857142857140	40.0	0.614814814814815	0.8	0.266666666666667
34		72.40000000000000	62.29508196721310	0.708376222336593	0.850746268656716	0.491379310344828	76.71232876712330	45.16129032258070	0.642173913043478	0.875	0.304347826086957
35		73.6	69.44444444444440	0.73836502144549	0.842696629213483	0.590551181102362	69.04761904761910	56.66666666666670	0.690476190476191	0.944444444444444	0.404761904761905
36		67.2	58.16326530612250	0.675628961014020	0.826086956521739	0.448818897637795	73.33333333333330	60.0	0.714285714285714	1.0	0.428571428571429
37		72.8	65.3061224489796	0.720512820512820	0.842105263157895	0.533333333333333	77.94117647058820	57.14285714285720	0.696969696969697	0.909090909090909	0.416666666666667
38		74.0	67.00507614213200	0.738351254480287	0.904109589041096	0.532258064516129	75.0	43.75000000000000	0.635416666666667	0.875	0.291666666666667
39		72.40000000000000	66.34146341463420	0.721304653991422	0.829268292682927	0.552845528455285	78.48101265822780	56.41025641025640	0.692670537010160	0.846153846153846	0.423076923076923
40		69.6	64.15094339622640	0.706728299948639	0.85	0.515151515151515	62.68656716417910	35.89743589743590	0.589639639639640	0.777777777777778	0.233333333333333
41		69.6	62.37623762376240	0.704807692307692	0.875	0.484615384615385	74.32432432432430	53.65853658536580	0.683333333333333	1.0	0.366666666666667
42		72.40000000000000	65.32663316582910	0.722414234511009	0.866666666666667	0.524193548387097	77.02702702702700	63.82978723404260	0.730307576894224	0.9375	0.483870967741936
43		74.0	68.29268292682930	0.744748975409836	0.909090909090909	0.546875	70.66666666666670	45.00000000000000	0.638888888888889	0.9	0.3
44		69.20000000000000	62.4390243902439	0.700320512820513	0.853333333333333	0.492307692307692	69.86301369863010	57.69230769230770	0.694819819819820	0.9375	0.416666666666667
45		74.0	67.33668341708540	0.735527663934426	0.870129870129870	0.549180327868853	77.96610169491530	51.85185185185190	0.671710526315790	0.875	0.368421052631579
46		70.0	63.76811594202900	0.711864406779661	0.88	0.5	71.62162162162160	55.31914893617020	0.684774575018478	0.928571428571429	0.393939393939394
47		70.0	62.68656716417910	0.701612903225806	0.84	0.5	76.19047619047620	48.27586206896550	0.654761904761905	0.875	0.333333333333333
48		71.6	66.35071090047390	0.718679982075411	0.833333333333333	0.551181102362205	74.64788732394370	60.8695652173913	0.708943089430894	0.875	0.466666666666667
49		69.6	61.61616161616160	0.701396004098361	0.871428571428571	0.4765625	75.67567567567570	52.63157894736840	0.674546887312845	0.909090909090909	0.370370370370370
50		70.8	63.31658291457290	0.711446130209334	0.875	0.496062992125984	71.23287671232880	43.24324324324320	0.631746031746032	0.888888888888889	0.285714285714286
51		68.4	59.8984771573604	0.693589743589744	0.880597014925373	0.453846153846154	70.3125	38.70967741935480	0.6125	0.857142857142857	0.25
52		62.0	51.28205128205130	0.632016165244724	0.78125	0.381679389312977	66.66666666666670	47.82608695652170	0.648606811145511	0.916666666666667	0.323529411764706
53		72.40000000000000	61.87845303867400	0.712393354288280	0.903225806451613	0.470588235294118	72.46376811594200	34.48275862068970	0.597826086956522	0.833333333333333	0.217391304347826
54		71.2	61.70212765957450	0.69899106741212	0.816901408450704	0.495726495726496	73.13432835820900	47.05882352941180	0.643410852713178	0.8	0.333333333333333
55		69.20000000000000	62.06896551724140	0.698731501057083	0.851351351351351	0.488372093023256	64.61538461538460	43.90243902439030	0.613505747126437	0.75	0.310344827586207
56		76.4	71.4975845410628	0.760309938524590	0.870588235294118	0.606557377049180	72.22222222222220	47.36842105263160	0.649350649350649	0.9	0.321428571428571
57		74.8	68.96551724137930	0.749551971326165	0.909090909090909	0.555555555555556	78.94736842105260	61.90476190476190	0.720332577475435	0.866666666666667	0.481481481481481
58		72.8	65.65656565656570	0.729710701484895	0.902777777777778	0.515873015873016	76.71232876712330	60.46511627906980	0.716666666666667	1.0	0.433333333333333
59		72.8	66.66666666666670	0.731131169579412	0.883116883116883	0.535433070866142	76.71232876712330	54.05405405405410	0.681669394435352	0.909090909090909	0.384615384615385
60		72.40000000000000	67.6056338028169	0.729482990582356	0.857142857142857	0.558139534883721	75.34246575342470	57.14285714285710	0.692063492063492	0.857142857142857	0.428571428571429
61		68.8	62.85714285714290	0.699152542372881	0.846153846153846	0.5	69.11764705882350	36.36363636363640	0.596744186046512	0.75	0.24
62		72.40000000000000	61.45251396648040	0.713782051282051	0.932203389830508	0.458333333333333	73.61111111111110	48.64864864864870	0.660714285714286	1.0	0.321428571428571
63		72.8	67.61904761904760	0.732261782786885	0.865853658536585	0.5546875	74.07407407407410	48.78048780487800	0.656862745098039	0.909090909090909	0.333333333333333
64		70.8	62.17616580310880	0.704532360284233	0.857142857142857	0.487804878048781	78.26086956521740	48.27586206896550	0.656250000000000	0.875	0.333333333333333
65		71.2	64.70588235294120	0.718623870843744	0.88	0.511627906976744	72.97297297297300	52.38095238095240	0.671969696969697	0.916666666666667	0.366666666666667
66		69.20000000000000	60.10362694300520	0.685405855596130	0.805555555555556	0.479338842975207	68.0	33.33333333333330	0.574568288854003	0.6	0.230769230769231
67		75.6	70.53140096618360	0.751265295662759	0.848837209302326	0.603305785123967	82.43243243243240	68.29268292682930	0.758814102564102	0.933333333333333	0.538461538461538
68		72.40000000000000	63.4920634920635	0.718557889344262	0.895522388059702	0.491803278688525	73.52941176470590	43.75000000000000	0.634469696969697	0.875	0.291666666666667
69		70.8	60.96256684491980	0.697511065494900	0.838235294117647	0.478991596638656	73.75	55.31914893617020	0.686331399097357	0.928571428571429	0.393939393939394
70		70.0	61.53846153846150	0.689843348741654	0.779220779220779	0.508474576271186	76.81159420289860	52.94117647058820	0.673913043478261	0.818181818181818	0.391304347826087
71		71.2	63.63636363636360	0.702555213148434	0.7875	0.533898305084746	75.36231884057970	56.41025641025640	0.685909090909091	0.785714285714286	0.44
72		69.6	64.48598130841120	0.714202786377709	0.884615384615385	0.507352941176471	72.15189873417720	52.17391304347830	0.666223404255319	0.857142857142857	0.375
73		73.6	65.625	0.719035582344935	0.777777777777778	0.567567567567568	71.875	52.63157894736840	0.678571428571429	1.0	0.357142857142857
74		72.40000000000000	69.05829596412560	0.739228586171311	0.885057471264368	0.566176470588235	75.34246575342470	55.00000000000000	0.678146258503401	0.6875	0.458333333333333
75		68.4	64.25339366515840	0.699625902992776	0.835294117647059	0.522058823529412	64.19753086419750	38.29787234042550	0.610257985257985	0.9	0.243243243243243
76		71.2	60.8695652173913	0.704273175731950	0.888888888888889	0.462809917355372	73.23943661971830	38.70967741935480	0.62	1.0	0.24
77		68.8	62.85714285714290	0.688000000000000	0.776470588235294	0.528	74.64788732394370	47.05882352941180	0.645390070921986	0.8	0.333333333333333
78		71.2	63.63636363636360	0.71551117085974	0.887323943661972	0.496062992125984	74.64788732394370	59.09090909090910	0.704471544715447	0.928571428571429	0.433333333333333
79		70.0	61.53846153846150	0.701804915514593	0.869565217391304	0.476190476190476	71.21212121212120	45.71428571428570	0.648148148148148	1.0	0.296296296296296
80		70.8	60.10928961748630	0.700140944326991	0.887096774193548	0.454545454545455	71.42857142857140	50.0	0.660763888888889	0.916666666666667	0.34375
81		71.6	60.33519553072630	0.695142432659389	0.818181818181818	0.477876106194690	75.0	51.28205128205130	0.672413793103448	1.0	0.344827586206897
82		69.20000000000000	65.47085201793720	0.715259740259740	0.879518072289157	0.521428571428572	63.49206349206350	46.51162790697670	0.630040322580645	0.833333333333333	0.322580645161290
83		75.6	68.06282722513090	0.738835958260419	0.8125	0.585585585585586	71.83098591549300	44.44444444444440	0.636784511784512	0.888888888888889	0.296296296296296
84		71.2	64.70588235294120	0.713517665130568	0.846153846153846	0.523809523809524	74.28571428571430	50.0	0.666666666666667	1.0	0.333333333333333
85		68.4	59.06735751295340	0.684000000000000	0.838235294117647	0.456	77.14285714285720	57.89473684210530	0.700174825174825	0.916666666666667	0.423076923076923
86		67.2	58.16326530612250	0.683943806530246	0.876923076923077	0.435114503816794	66.66666666666670	42.55319148936170	0.627777777777778	0.909090909090909	0.277777777777778
87		73.2	65.98984771573610	0.727330942622951	0.866666666666667	0.532786885245902	73.91304347826090	59.09090909090910	0.703846153846154	0.928571428571429	0.433333333333333
88		73.6	68.57142857142860	0.749309170361802	0.935064935064935	0.541353383458647	73.84615384615390	56.41025641025640	0.6825	0.785714285714286	0.44
89		75.2	68.3673469387755	0.750320020481311	0.930555555555556	0.540322580645161	77.33333333333330	54.05405405405410	0.685185185185185	1.0	0.370370370370370
90		70.8	66.96832579185520	0.723813209494324	0.870588235294118	0.544117647058824	70.3125	53.65853658536580	0.668650793650794	0.846153846153846	0.392857142857143
91		68.4	62.55924170616110	0.698854863612970	0.857142857142857	0.492537313432836	71.1864406779661	54.05405405405410	0.677156177156177	0.909090909090909	0.384615384615385
92		73.6	67.32673267326730	0.731813524590164	0.85	0.557377049180328	74.07407407407410	51.16279069767440	0.663725490196078	0.846153846153846	0.366666666666667
93		73.6	65.26315789473690	0.724833076527992	0.861111111111111	0.525423728813559	80.26315789473690	54.54545454545460	0.686026936026936	0.818181818181818	0.409090909090909
94		71.6	63.21243523316060	0.7109375	0.859154929577465	0.5	73.07692307692310	46.15384615384620	0.65	1.0	0.3
95		66.8	59.11330049261080	0.695069875776398	0.923076923076923	0.434782608695652	68.75	41.86046511627910	0.625725338491296	0.9	0.272727272727273
96		70.8	62.94416243654820	0.701934781215965	0.815789473684211	0.512396694214876	74.28571428571430	55.00000000000000	0.684523809523810	0.916666666666667	0.392857142857143
97		69.6	62.74509803921570	0.699122975481723	0.831168831168831	0.503937007874016	77.5	59.09090909090910	0.706666666666667	0.928571428571429	0.433333333333333
98		73.2	65.98984771573600	0.728794571410281	0.878378378378378	0.528455284552846	77.92207792207790	45.16129032258060	0.640909090909091	0.777777777777778	0.318181818181818
99		72.40000000000000	64.97461928934010	0.717951182010379	0.842105263157895	0.528925619834711	71.7948717948718	52.17391304347830	0.670707070707071	0.923076923076923	0.363636363636364
	**Mean**	**71.556**	**64.5742618809272**	**0.716734574512024**	**0.860688165743776**	**0.518576576835636**	**73.9320354015206**	**52.1900464274983**	**0.672443525601415**	**0.889216215801742**	**0.374277177170425**
	**Standard Deviation**	2.6040496822209	3.62365507712517	0.022447400059545	0.036839933481112	0.044964344536164	4.45094212543587	8.1290290707689	0.040596429819345	0.079775216423663	0.079231951096477
	**T Test Significance (Female to Male)**						**0.002834348791405**	**0.000000000000000**	**0.000000000062756**	**0.030231019371983**	**0.000000000000000**

		UB_F_FNR	UB_F_TNR	UB_F_FPR	UB_F_TPR	UB_M_Accuracy	UB_M_FScore	UB_M_ROC_AUC	UB_M_Precision	UB_M_Recall	UB_M_FNR	UB_M_TNR	UB_M_FPR	UB_M_TPR
0		47.36842105263160	95.55555555555560	4.444444444444450	52.63157894736840	74.19354838709680	70.37037037037040	0.746875	0.863636363636364	0.59375	40.625	90.0	10.0	59.375
1		60.86956521739130	97.61904761904760	2.380952380952380	39.13043478260870	64.32432432432430	62.92134831460670	0.707437389324564	0.933333333333333	0.474576271186441	52.54237288135590	94.02985074626870	5.970149253731340	47.45762711864410
2		54.54545454545450	100.0	0.0	45.45454545454550	71.34831460674160	68.71165644171780	0.732195371435878	0.875	0.565656565656566	43.43434343434340	89.87341772151900	10.12658227848100	56.56565656565660
3		72.22222222222220	91.30434782608700	8.695652173913040	27.77777777777780	71.42857142857140	69.23076923076920	0.721092305504196	0.805970149253731	0.606741573033708	39.32584269662920	83.54430379746840	16.45569620253160	60.67415730337080
4		60.0	100.0	0.0	40.0	68.71508379888270	63.63636363636360	0.691591704147926	0.790322580645161	0.532608695652174	46.73913043478260	85.0574712643678	14.94252873563220	53.2608695652174
5		66.66666666666670	97.22222222222220	2.777777777777780	33.33333333333330	71.65775401069520	70.05649717514120	0.729542302357836	0.837837837837838	0.601941747572816	39.80582524271850	85.71428571428570	14.28571428571430	60.19417475728160
6		59.25925925925930	95.65217391304350	4.347826086956520	40.74074074074070	73.44632768361580	66.66666666666670	0.731226053639847	0.870370370370370	0.540229885057471	45.97701149425290	92.22222222222220	7.777777777777780	54.02298850574710
7		50.0	97.82608695652170	2.173913043478260	50.0	72.77777777777780	71.67630057803470	0.743639553828801	0.861111111111111	0.613861386138614	38.61386138613860	87.34177215189870	12.65822784810130	61.38613861386140
8		50.0	100.0	0.0	50.0	65.3409090909091	63.03030303030300	0.6875	0.852459016393443	0.5	50.0	87.5	12.5	50.0
9		61.53846153846150	97.2972972972973	2.702702702702700	38.46153846153850	66.84491978609630	62.19512195121950	0.697356161192639	0.879310344827586	0.481132075471698	51.88679245283020	91.35802469135800	8.641975308641980	48.11320754716980
10		70.37037037037040	100.0	0.0	29.62962962962960	65.95744680851060	65.95744680851060	0.684680451127820	0.815789473684211	0.553571428571429	44.64285714285710	81.57894736842110	18.42105263157890	55.35714285714290
11		60.0	94.87179487179490	5.128205128205130	40.0	66.66666666666670	59.21052631578950	0.681295715778475	0.849056603773585	0.454545454545455	54.54545454545450	90.80459770114940	9.195402298850570	45.45454545454550
12		58.33333333333330	94.73684210526320	5.263157894736840	41.66666666666670	72.3404255319149	68.29268292682930	0.734545454545455	0.875	0.56	44.0	90.9090909090909	9.090909090909090	56.00000000000000
13		66.66666666666670	95.65217391304350	4.347826086956520	33.33333333333330	71.66666666666670	71.50837988826820	0.735323886639676	0.853333333333333	0.615384615384615	38.46153846153850	85.52631578947370	14.47368421052630	61.53846153846150
14		65.51724137931040	97.67441860465120	2.325581395348840	34.48275862068970	70.78651685393260	71.73913043478260	0.737534902273634	0.88	0.605504587155963	39.44954128440370	86.95652173913040	13.04347826086960	60.55045871559630
15		68.75	100.0	0.0	31.25	71.1864406779661	69.46107784431140	0.741012857517712	0.90625	0.563106796116505	43.68932038834950	91.8918918918919	8.108108108108110	56.31067961165050
16		56.52173913043480	95.23809523809520	4.761904761904760	43.47826086956520	73.51351351351350	71.00591715976330	0.747058823529412	0.869565217391304	0.6	40.0	89.41176470588240	10.58823529411760	60.0
17		61.11111111111110	97.72727272727270	2.272727272727270	38.88888888888890	71.76470588235290	70.37037037037040	0.722916666666667	0.791666666666667	0.633333333333333	36.66666666666670	81.25	18.75	63.33333333333330
18		59.09090909090910	95.0	5.0	40.90909090909090	68.08510638297870	65.11627906976740	0.706126687435099	0.861538461538462	0.523364485981308	47.66355140186920	88.88888888888890	11.11111111111110	52.33644859813080
19		47.82608695652170	97.22222222222220	2.777777777777780	52.17391304347830	68.58638743455500	66.29213483146070	0.699557032115172	0.808219178082192	0.561904761904762	43.80952380952380	83.72093023255820	16.27906976744190	56.19047619047620
20		74.19354838709680	97.61904761904760	2.380952380952380	25.80645161290320	71.75141242937850	65.75342465753420	0.725575447570333	0.888888888888889	0.521739130434783	47.82608695652170	92.94117647058820	7.058823529411760	52.17391304347830
21		65.51724137931040	94.87179487179490	5.128205128205130	34.48275862068970	71.42857142857140	69.04761904761910	0.747269116186693	0.935483870967742	0.547169811320755	45.28301886792450	94.73684210526320	5.263157894736840	54.71698113207550
22		66.66666666666670	95.23809523809520	4.761904761904760	33.33333333333330	76.96629213483150	74.84662576687120	0.778883956880152	0.897058823529412	0.642105263157895	35.78947368421050	91.56626506024100	8.433734939759040	64.21052631578950
23		52.0	100.0	0.0	48.0	71.75141242937850	69.51219512195120	0.739545454545454	0.890625	0.57	43.0	90.9090909090909	9.090909090909090	57.00000000000000
24		62.06896551724140	97.22222222222220	2.777777777777780	37.93103448275860	71.35135135135140	68.26347305389220	0.731218993621545	0.876923076923077	0.558823529411765	44.11764705882350	90.36144578313250	9.63855421686747	55.88235294117650
25		65.38461538461540	97.87234042553190	2.127659574468090	34.61538461538460	69.49152542372880	67.46987951807230	0.724547362896877	0.888888888888889	0.543689320388350	45.63106796116510	90.54054054054050	9.45945945945946	54.36893203883500
26		38.46153846153850	93.75	6.25	61.53846153846150	69.88636363636360	65.80645161290320	0.701937984496124	0.784615384615385	0.566666666666667	43.33333333333330	83.72093023255820	16.27906976744190	56.66666666666670
27		44.0	95.34883720930230	4.651162790697680	56.00000000000000	73.07692307692310	71.00591715976330	0.732693004711852	0.789473684210526	0.645161290322581	35.48387096774190	82.02247191011240	17.97752808988760	64.51612903225810
28		51.85185185185190	100.0	0.0	48.14814814814810	71.8232044198895	67.9245283018868	0.726070991432069	0.84375	0.568421052631579	43.15789473684210	88.37209302325580	11.62790697674420	56.84210526315790
29		50.0	100.0	0.0	50.0	73.33333333333330	68.42105263157900	0.739340007415647	0.881355932203390	0.559139784946237	44.08602150537630	91.95402298850570	8.045977011494250	55.91397849462370
30		57.14285714285710	97.36842105263160	2.631578947368420	42.85714285714290	77.17391304347830	76.40449438202250	0.784552845528455	0.894736842105263	0.666666666666667	33.33333333333330	90.2439024390244	9.75609756097561	66.66666666666670
31		63.1578947368421	93.47826086956520	6.521739130434780	36.84210526315790	74.05405405405410	69.23076923076920	0.739714820009350	0.84375	0.586956521739131	41.30434782608700	89.24731182795700	10.75268817204300	58.69565217391310
32		57.69230769230770	95.45454545454550	4.545454545454550	42.30769230769230	76.11111111111110	73.93939393939390	0.772264314991927	0.897058823529412	0.628865979381443	37.11340206185570	91.56626506024100	8.433734939759040	62.88659793814430
33		73.33333333333330	96.29629629629630	3.703703703703700	26.66666666666670	66.26506024096390	60.56338028169010	0.676637968772800	0.811320754716981	0.483146067415730	51.68539325842700	87.01298701298700	12.98701298701300	48.31460674157300
34		69.56521739130430	98.0	2.0	30.43478260869570	70.62146892655370	65.78947368421050	0.715245775729647	0.847457627118644	0.537634408602151	46.23655913978490	89.28571428571430	10.71428571428570	53.76344086021510
35		59.52380952380950	97.61904761904760	2.380952380952380	40.47619047619050	75.90361445783130	74.35897435897440	0.760929557007988	0.816901408450704	0.682352941176471	31.76470588235290	83.9506172839506	16.04938271604940	68.23529411764710
36		57.14285714285710	100.0	0.0	42.85714285714290	64.57142857142860	57.53424657534250	0.655971712938711	0.777777777777778	0.456521739130435	54.34782608695650	85.54216867469880	14.45783132530120	45.65217391304350
37		58.33333333333330	97.72727272727270	2.272727272727270	41.66666666666670	70.87912087912090	67.0807453416149	0.717296511627907	0.830769230769231	0.5625	43.75	87.20930232558140	12.79069767441860	56.25
38		70.83333333333330	97.91666666666670	2.083333333333330	29.16666666666670	73.59550561797750	71.51515151515150	0.756538461538462	0.907692307692308	0.59	41.0	92.3076923076923	7.692307692307690	59.0
39		57.69230769230770	96.22641509433960	3.773584905660380	42.30769230769230	69.5906432748538	68.67469879518070	0.712733351908610	0.826086956521739	0.587628865979382	41.23711340206190	83.78378378378380	16.21621621621620	58.76288659793820
40		76.66666666666670	94.5945945945946	5.405405405405410	23.33333333333330	72.1311475409836	70.52023121387290	0.737291212781409	0.859154929577465	0.598039215686275	40.19607843137260	87.65432098765430	12.34567901234570	59.80392156862750
41		63.33333333333330	100.0	0.0	36.66666666666670	67.61363636363640	64.59627329192550	0.700789473684210	0.852459016393443	0.52	48.0	88.1578947368421	11.84210526315790	52.0
42		51.61290322580650	97.67441860465120	2.325581395348840	48.38709677419360	70.45454545454550	65.78947368421050	0.714600336831196	0.847457627118644	0.537634408602151	46.23655913978490	89.1566265060241	10.84337349397590	53.76344086021510
43		70.0	97.77777777777780	2.222222222222220	30.0	75.42857142857140	73.93939393939400	0.772263450834880	0.910447761194030	0.622448979591837	37.75510204081630	92.20779220779220	7.792207792207790	62.24489795918370
44		58.33333333333330	97.2972972972973	2.702702702702700	41.66666666666670	68.92655367231640	64.05228758169940	0.700397334016919	0.830508474576271	0.521276595744681	47.87234042553190	87.95180722891570	12.04819277108430	52.12765957446810
45		63.1578947368421	97.5	2.5	36.84210526315790	72.77486910994760	69.76744186046510	0.740125772285966	0.869565217391304	0.582524271844660	41.74757281553400	89.77272727272730	10.22727272727270	58.25242718446600
46		60.60606060606060	97.5609756097561	2.439024390243900	39.39393939393940	69.31818181818180	66.25	0.715728715728716	0.868852459016393	0.535353535353535	46.46464646464650	89.6103896103896	10.38961038961040	53.53535353535350
47		66.66666666666670	97.61904761904760	2.380952380952380	33.33333333333330	67.9144385026738	65.11627906976740	0.699593495934959	0.835820895522388	0.533333333333333	46.66666666666670	86.58536585365850	13.41463414634150	53.33333333333330
48		53.33333333333330	95.1219512195122	4.878048780487810	46.66666666666670	70.39106145251400	67.87878787878790	0.715489062107116	0.823529411764706	0.577319587628866	42.2680412371134	85.36585365853660	14.63414634146340	57.73195876288660
49		62.96296296296300	97.87234042553190	2.127659574468090	37.03703703703700	67.04545454545460	63.75000000000000	0.699141914191419	0.864406779661017	0.504950495049505	49.50495049504950	89.33333333333330	10.66666666666670	50.49504950495050
50		71.42857142857140	97.77777777777780	2.222222222222220	28.57142857142860	70.62146892655370	67.90123456790120	0.726495726495727	0.873015873015873	0.555555555555556	44.44444444444440	89.74358974358980	10.25641025641030	55.55555555555560
51		75.0	97.5	2.5	25.0	67.74193548387100	63.85542168674700	0.706250000000000	0.883333333333333	0.5	50.0	91.25	8.75	50.0
52		67.64705882352940	97.36842105263160	2.631578947368420	32.35294117647060	60.1123595505618	52.34899328859060	0.620784014254805	0.75	0.402061855670103	59.79381443298970	83.9506172839506	16.04938271604940	40.20618556701030
53		78.26086956521740	97.82608695652170	2.173913043478260	21.73913043478260	72.37569060773480	67.10526315789470	0.736213235294118	0.910714285714286	0.53125	46.875	94.11764705882350	5.88235294117647	53.125
54		66.66666666666670	95.34883720930230	4.651162790697680	33.33333333333330	70.49180327868850	64.93506493506490	0.707706093189964	0.819672131147541	0.537634408602151	46.23655913978490	87.77777777777780	12.22222222222220	53.76344086021510
55		68.96551724137930	91.66666666666670	8.333333333333330	31.03448275862070	70.8108108108108	66.66666666666670	0.722941176470588	0.870967741935484	0.54	46.0	90.58823529411770	9.411764705882350	54.0
56		67.85714285714290	97.72727272727270	2.272727272727270	32.14285714285710	78.08988764044940	76.92307692307690	0.786220871327254	0.866666666666667	0.691489361702128	30.85106382978720	88.09523809523810	11.90476190476190	69.14893617021280
57		51.85185185185190	95.91836734693880	4.081632653061230	48.14814814814810	72.98850574712640	70.80745341614910	0.754545454545455	0.919354838709677	0.575757575757576	42.42424242424240	93.33333333333330	6.666666666666670	57.57575757575760
58		56.66666666666670	100.0	0.0	43.33333333333330	71.1864406779661	67.09677419354840	0.727623456790123	0.881355932203390	0.541666666666667	45.83333333333330	91.35802469135800	8.641975308641980	54.16666666666670
59		61.53846153846150	97.87234042553190	2.127659574468090	38.46153846153850	71.1864406779661	69.46107784431140	0.734497133923919	0.878787878787879	0.574257425742574	42.57425742574260	89.47368421052630	10.52631578947370	57.42574257425740
60		57.14285714285710	95.55555555555560	4.444444444444450	42.85714285714290	71.1864406779661	70.17543859649120	0.731240229286087	0.857142857142857	0.594059405940594	40.5940594059406	86.8421052631579	13.15789473684210	59.4059405940594
61		76.0	95.34883720930230	4.651162790697680	24.0	68.68131868131870	67.79661016949150	0.713707165109034	0.857142857142857	0.560747663551402	43.92523364485980	86.66666666666670	13.33333333333330	56.07476635514020
62		67.85714285714290	100.0	0.0	32.14285714285710	71.91011235955060	64.7887323943662	0.726744186046512	0.92	0.5	50.0	95.34883720930230	4.651162790697680	50.0
63		66.66666666666670	98.0392156862745	1.960784313725490	33.33333333333330	72.18934911242600	72.18934911242600	0.740801954584651	0.859154929577465	0.622448979591837	37.75510204081630	85.91549295774650	14.08450704225350	62.24489795918370
64		66.66666666666670	97.91666666666670	2.083333333333330	33.33333333333330	67.95580110497240	64.63414634146340	0.702841896252172	0.854838709677419	0.519607843137255	48.03921568627450	88.60759493670890	11.39240506329110	51.9607843137255
65		63.33333333333330	97.72727272727270	2.272727272727270	36.66666666666670	70.45454545454550	67.90123456790120	0.725829725829726	0.873015873015873	0.555555555555556	44.44444444444440	89.6103896103896	10.38961038961040	55.55555555555560
66		76.92307692307690	91.83673469387760	8.16326530612245	23.07692307692310	69.71428571428570	66.2420382165605	0.711184210526316	0.838709677419355	0.547368421052632	45.26315789473680	87.5	12.5	54.73684210526320
67		46.15384615384620	97.91666666666670	2.083333333333330	53.84615384615390	72.72727272727270	71.0843373493976	0.736452241715400	0.830985915492958	0.621052631578947	37.89473684210530	85.18518518518520	14.81481481481480	62.10526315789470
68		70.83333333333330	97.72727272727270	2.272727272727270	29.16666666666670	71.97802197802200	67.51592356687900	0.734693877551020	0.898305084745763	0.540816326530612	45.91836734693880	92.85714285714290	7.142857142857140	54.08163265306120
69		60.60606060606060	97.87234042553190	2.127659574468090	39.39393939393940	69.41176470588240	62.85714285714290	0.696290143964563	0.814814814814815	0.511627906976744	48.83720930232560	88.09523809523810	11.90476190476190	51.16279069767440
70		60.86956521739130	95.65217391304350	4.347826086956520	39.13043478260870	67.40331491712710	63.35403726708070	0.681211750305998	0.772727272727273	0.536842105263158	46.31578947368420	82.55813953488370	17.44186046511630	53.68421052631580
71		56.00000000000000	93.18181818181820	6.818181818181820	44.0	69.61325966850830	65.40880503144650	0.700024437927664	0.787878787878788	0.559139784946237	44.08602150537630	84.0909090909091	15.90909090909090	55.91397849462370
72		62.5	95.74468085106380	4.25531914893617	37.5	68.42105263157900	67.85714285714290	0.721799655568312	0.890625	0.548076923076923	45.19230769230770	89.55223880597020	10.44776119402990	54.80769230769230
73		64.28571428571430	100.0	0.0	35.71428571428570	74.19354838709680	68.83116883116880	0.731898467657036	0.746478873239437	0.638554216867470	36.14457831325300	82.52427184466020	17.47572815533980	63.85542168674700
74		54.16666666666670	89.79591836734690	10.20408163265310	45.83333333333330	71.1864406779661	72.13114754098360	0.756181318681319	0.929577464788732	0.589285714285714	41.07142857142860	92.3076923076923	7.692307692307690	58.92857142857140
75		75.67567567567570	97.72727272727270	2.272727272727270	24.32432432432430	70.41420118343200	71.26436781609200	0.720274170274170	0.826666666666667	0.626262626262626	37.37373737373740	81.42857142857140	18.57142857142860	62.62626262626260
76		76.0	100.0	0.0	24.0	70.39106145251400	65.359477124183	0.718247991967872	0.877192982456140	0.520833333333333	47.91666666666670	91.56626506024100	8.433734939759040	52.08333333333330
77		66.66666666666670	95.74468085106380	4.25531914893617	33.33333333333330	66.4804469273743	65.9090909090909	0.678154353896928	0.773333333333333	0.574257425742574	42.57425742574260	78.2051282051282	21.79487179487180	57.42574257425740
78		56.66666666666670	97.5609756097561	2.439024390243900	43.33333333333330	69.83240223463690	64.93506493506490	0.715049031933618	0.877192982456140	0.515463917525773	48.45360824742270	91.46341463414640	8.536585365853660	51.54639175257730
79		70.37037037037040	100.0	0.0	29.62962962962960	69.56521739130430	65.00000000000000	0.709685086155675	0.852459016393443	0.525252525252525	47.47474747474750	89.41176470588240	10.58823529411760	52.52525252525250
80		65.625	97.77777777777780	2.222222222222220	34.375	70.52023121387280	63.30935251798560	0.711476725521669	0.88	0.494382022471910	50.56179775280900	92.85714285714290	7.142857142857140	49.43820224719100
81		65.51724137931040	100.0	0.0	34.48275862068970	70.11494252873560	62.85714285714290	0.695238095238095	0.785714285714286	0.523809523809524	47.61904761904760	86.66666666666670	13.33333333333330	52.38095238095240
82		67.74193548387100	93.75	6.25	32.25806451612900	71.12299465240640	70.0	0.737708774406022	0.887323943661972	0.577981651376147	42.20183486238530	89.74358974358980	10.25641025641030	57.79816513761470
83		70.37037037037040	97.72727272727270	2.272727272727270	29.62962962962960	77.09497206703910	73.5483870967742	0.765601503759399	0.802816901408451	0.678571428571429	32.14285714285710	85.26315789473680	14.73684210526320	67.85714285714290
84		66.66666666666670	100.0	0.0	33.33333333333330	70.0	67.85714285714290	0.713804713804714	0.826086956521739	0.575757575757576	42.42424242424240	85.18518518518520	14.81481481481480	57.57575757575760
85		57.69230769230770	97.72727272727270	2.272727272727270	42.30769230769230	65.0	59.35483870967740	0.670594837261504	0.821428571428571	0.464646464646465	53.53535353535350	87.65432098765430	12.34567901234570	46.46464646464650
86		72.22222222222220	97.77777777777780	2.222222222222220	27.77777777777780	67.45562130177520	63.08724832214770	0.700071123755334	0.870370370370370	0.494736842105263	50.52631578947370	90.54054054054050	9.45945945945946	49.47368421052630
87		56.66666666666670	97.43589743589740	2.564102564102560	43.33333333333330	72.92817679558010	67.97385620915030	0.732046897899365	0.852459016393443	0.565217391304348	43.47826086956520	89.8876404494382	10.11235955056180	56.52173913043480
88		56.00000000000000	92.5	7.5	44.0	73.51351351351350	71.34502923976610	0.769420394420394	0.968253968253968	0.564814814814815	43.51851851851850	97.40259740259740	2.597402597402600	56.48148148148150
89		62.96296296296300	100.0	0.0	37.03703703703700	74.28571428571430	71.69811320754720	0.761763150938409	0.919354838709677	0.587628865979382	41.23711340206190	93.58974358974360	6.41025641025641	58.76288659793820
90		60.71428571428570	94.44444444444440	5.555555555555560	39.28571428571430	70.96774193548390	70.0	0.733974358974359	0.875	0.583333333333333	41.66666666666670	88.46153846153850	11.53846153846150	58.33333333333330
91		61.53846153846150	96.96969696969700	3.030303030303030	38.46153846153850	67.5392670157068	64.36781609195400	0.699018295403838	0.848484848484849	0.518518518518519	48.14814814814810	87.95180722891570	12.04819277108430	51.85185185185190
92		63.33333333333330	96.07843137254900	3.921568627450980	36.66666666666670	73.37278106508880	71.69811320754720	0.744847543760587	0.850746268656716	0.619565217391304	38.04347826086960	87.01298701298700	12.98701298701300	61.95652173913040
93		59.09090909090910	96.29629629629630	3.703703703703700	40.90909090909090	70.6896551724138	67.51592356687900	0.724759615384616	0.868852459016393	0.552083333333333	44.79166666666670	89.74358974358980	10.25641025641030	55.20833333333330
94		70.0	100.0	0.0	30.0	70.93023255813950	67.53246753246750	0.720108695652174	0.838709677419355	0.565217391304348	43.47826086956520	87.5	12.5	56.52173913043480
95		72.72727272727270	97.87234042553190	2.127659574468090	27.27272727272730	65.88235294117650	63.75000000000000	0.712087912087912	0.927272727272727	0.485714285714286	51.42857142857140	93.84615384615380	6.153846153846150	48.57142857142860
96		60.71428571428570	97.61904761904760	2.380952380952380	39.28571428571430	69.44444444444440	64.96815286624200	0.699480904708936	0.796875	0.548387096774194	45.16129032258060	85.0574712643678	14.94252873563220	54.83870967741940
97		56.66666666666670	98.0	2.0	43.33333333333330	65.88235294117650	63.75000000000000	0.680694817116227	0.809523809523810	0.525773195876289	47.42268041237110	83.56164383561640	16.43835616438360	52.57731958762890
98		68.18181818181820	96.36363636363640	3.636363636363640	31.81818181818180	71.09826589595380	69.87951807228910	0.738517601760176	0.892307692307692	0.574257425742574	42.57425742574260	90.27777777777780	9.722222222222220	57.42574257425740
99		63.63636363636360	97.77777777777780	2.222222222222220	36.36363636363640	72.67441860465120	68.87417218543050	0.729978354978355	0.825396825396825	0.590909090909091	40.90909090909090	86.90476190476190	13.09523809523810	59.09090909090910
	**Mean**	**62.5722822829575**	**97.0609874032405**	**2.93901259675946**	**37.4277177170425**	**70.62089057366100**	**67.47955563246060**	**0.722296801016263**	**0.85511677834028**	**0.559675147508725**	**44.0324852491275**	**88.4918454523801**	**11.5081545476199**	**55.9675147508725**
	**Standard Deviation**	7.9231951096477	2.17604982483912	2.17604982483912	7.9231951096477	3.03770076624728	3.98044951917968	0.027303596619814	0.042975164493108	0.051473882302335	5.14738823023349	3.5398801737506	3.5398801737506	5.1473882302335
	**T Test Significance (Female to Male)**	**0.000000000000000**	**0.000000000009121**	**0.000000000000000**	**0.000000000000000**									

Summary of Results for Experiment 3.1.4

	Random Forest Classifier			Logistic Regression Classifier			Support Vector Machine			Gaussian Naive Bayes		
	Mean	Standard Deviation	T Test Significance (Female to Male)	Mean	Standard Deviation	T Test Significance (Female to Male)	Mean	Standard Deviation	T Test Significance (Female to Male)	Mean	Standard Deviation	T Test Significance (Female to Male)
**All Accuracy**	80.37	2.83		73.28	2.03		71.60	2.20		73.04	1.77	
**All FScore**	75.34	3.77		62.61	3.27		57.44	3.28		60.22	3.19	
**All ROC_AUC**	79.14%	2.95%		70.72%	2.11%		68.43%	1.92%		69.99%	1.75%	
**All Precision**	81.66%	4.30%		78.15%	4.40%		81.91%	4.45%		83.13%	4.49%	
**All Recall**	70.14%	4.99%		52.42%	4.14%		44.37%	3.66%		47.44%	4.10%	
Females Accuracy	83.18	3.55	0.00	76.69	2.55	0.00	74.70	3.06	0.00	75.36	2.89	0.00
Females FScore	70.21	6.99	0.00	53.18	5.80	0.00	46.78	5.96	0.00	45.78	6.45	0.00
Females ROC_AUC	78.02%	4.71%	0.46	67.48%	3.15%	0.00	64.32%	2.97%	0.00	64.09%	3.10%	0.00
Females Precision	78.65%	6.76%	0.00	71.91%	7.85%	0.00	73.34%	8.33%	0.00	76.37%	9.99%	0.00
Females Recall	63.99%	9.10%	0.00	42.59%	6.27%	0.00	34.74%	5.85%	0.00	33.05%	5.84%	0.00
**Females FNR**	36.01	9.10	0.00	57.41	6.27	0.00	65.26	5.85	0.00	66.95	5.84	0.00
**Females TNR**	92.05	2.72	0.00	92.37	2.27	0.00	93.90	2.23	0.01	95.12	2.35	0.00
**Females FPR**	7.95	2.72	0.00	7.63	2.27	0.00	6.10	2.23	0.00	4.88	2.35	0.00
**Females TPR**	63.99	9.10	0.00	42.59	6.27	0.00	34.74	5.85	0.00	33.05	5.84	0.00
**Males Accuracy**	77.56	3.23		69.88	2.92		68.50	2.93		70.73	2.73	
**Males FScore**	78.07	3.31		67.57	3.45		63.24	3.93		67.41	3.87	
**Males ROC_AUC**	77.98%	3.24%		71.05%	2.79%		70.27%	2.67%		72.25%	2.33%	
**Males Precision**	83.25%	4.48%		81.19%	4.86%		86.16%	4.79%		85.72%	3.83%	
**Males Recall**	73.69%	4.33%		58.10%	4.50%		50.11%	4.35%		55.83%	5.34%	
**Males FNR**	26.31	4.33		41.90	4.50		49.89	4.35		44.17	5.34	
**Males TNR**	82.26	5.02		84.00	4.40		90.43	3.39		88.68	3.56	
**Males FPR**	17.74	5.02		16.00	4.40		9.57	3.39		11.32	3.56	
**Males TPR**	73.69	4.33		58.10	4.50		50.11	4.35		55.83	5.34	
	**Random Forest Results**			**Logistic Regression Results**			**Support Vector Machine Results**			**Gaussian Naive Bayes Results**		
**Sex Performance Disparities (Male Mean - Female Mean, n=100)**	Sex Performance Disparities (Male Mean - Female Mean, n=100)		**T Test P Value**	Sex Performance Disparities (Male Mean - Female Mean, n=100)		**T Test P Value**	Sex Performance Disparities (Male Mean - Female Mean, n=100)		**T Test P Value**	Sex Performance Disparities (Male Mean - Female Mean, n=100)		**T Test P Value**
**Accuacy**	-5.62		0.00	-6.80		0.00	-6.19		0.00	-4.64		0.00
**FScore**	7.86		0.00	14.39		0.00	16.46		0.00	21.63		0.00
**ROC_AUC**	-0.05%		0.46	3.57%		0.00	5.95%		0.00	8.17%		0.00
**Precision**	4.60%		0.00	9.28%		0.00	12.82%		0.00	9.35%		0.00
**Recall**	9.70%		0.00	15.51%		0.00	15.38%		0.00	22.78%		0.00
**False Negative Rate**	-9.70		0.00	-15.51		0.00	-15.38		0.00	-22.78		0.00
**True Negative Rate**	-9.79		0.00	-8.37		0.00	-3.47		0.00	-6.44		0.00
**False Positive Rate**	9.79		0.00	8.37		0.00	3.47		0.00	6.44		0.00
**True Positive Rate**	9.70		0.00	15.51		0.00	15.38		0.00	22.78		0.00

3.1.4 Balanced training data, with feature selection - Random Forest Classifier

		UB_Accuracy	UB_FScore	UB_ROC_AUC	UB_Precision	UB_Recall	UB_F_Accuracy	UB_F_FScore	UB_F_ROC_AUC	UB_F_Precision	UB_F_Recall	UB_F_FNR	UB_F_TNR	UB_F_FPR	UB_F_TPR	UB_M_Accuracy	UB_M_FScore	UB_M_ROC_AUC	UB_M_Precision	UB_M_Recall	UB_M_FNR	UB_M_TNR	UB_M_FPR	UB_M_TPR
0		82.9608938547486	78.89273356401390	0.818659281894576	0.8444444444444440	0.7402597402597400	83.79888268156430	72.38095238095240	0.7903961242519240	0.8085106382978720	0.6551724137931030	34.48275862068970	92.56198347107440	7.43801652892562	65.51724137931040	82.12290502793300	82.6086956521739	0.8235441767068270	0.8636363636363640	0.7916666666666670	20.83333333333330	85.54216867469880	14.457831325301200	79.16666666666670
1		84.63687150837990	80.7017543859649	0.8339037297588490	0.8984375	0.732484076433121	86.03351955307260	73.11827956989250	0.7948148148148150	0.8717948717948720	0.6296296296296300	37.03703703703700	96.0	4.0	62.96296296296300	83.24022346368710	84.375	0.8405723045477770	0.9101123595505620	0.7864077669902910	21.35922330097090	89.47368421052630	10.526315789473700	78.64077669902910
2		77.93296089385480	71.68458781362010	0.7637449694204140	0.819672131147541	0.6369426751592360	81.56424581005590	71.79487179487180	0.7777185501066100	0.84	0.6268656716417910	37.3134328358209	92.85714285714290	7.142857142857140	62.68656716417910	74.30167597765360	71.60493827160490	0.7435705368289640	0.8055555555555560	0.6444444444444450	35.55555555555560	84.26966292134830	15.730337078651700	64.44444444444440
3		78.77094972067040	72.66187050359710	0.7715572304547780	0.8015873015873020	0.6644736842105260	82.68156424581010	69.30693069306930	0.771849593495935	0.7777777777777780	0.625	37.5	91.869918699187	8.130081300813010	62.5	74.86033519553070	74.57627118644070	0.7533885542168680	0.8148148148148150	0.6875	31.25	81.92771084337350	18.072289156626500	68.75
4		85.75418994413410	81.72043010752690	0.8434466019417480	0.8976377952755910	0.75	87.70949720670390	76.08695652173910	0.8228682170542640	0.8333333333333330	0.7	30.0	94.57364341085270	5.426356589147290	70.0	83.79888268156430	84.49197860962570	0.8482938629997450	0.9294117647058820	0.7745098039215690	22.54901960784310	92.20779220779220	7.792207792207790	77.45098039215690
5		81.00558659217880	75.88652482269510	0.7980421297492890	0.7867647058823530	0.7328767123287670	84.35754189944130	75.43859649122810	0.8121148459383750	0.7962962962962960	0.7166666666666670	28.333333333333300	90.75630252100840	9.243697478991600	71.66666666666670	77.6536312849162	76.1904761904762	0.7753188297074270	0.7804878048780490	0.7441860465116280	25.58139534883720	80.64516129032260	19.35483870967740	74.4186046511628
6		83.24022346368710	79.72972972972970	0.8237496826605740	0.8428571428571430	0.7564102564102560	87.70949720670390	78.43137254901960	0.8383703703703700	0.8333333333333330	0.7407407407407410	25.925925925925900	93.60000000000000	6.4	74.07407407407410	78.77094972067040	80.41237113402060	0.7914438502673800	0.8478260869565220	0.7647058823529410	23.52941176470590	81.81818181818180	18.181818181818200	76.47058823529410
7		81.28491620111730	76.97594501718210	0.801961529929968	0.835820895522388	0.7133757961783440	87.15083798882680	78.09523809523810	0.824859943977591	0.9111111111111110	0.6833333333333330	31.666666666666700	96.63865546218490	3.361344537815130	68.33333333333330	75.41899441340780	76.34408602150540	0.7562232838823230	0.797752808988764	0.7319587628865980	26.80412371134020	78.04878048780490	21.951219512195100	73.19587628865980
8		74.02234636871510	67.82006920415230	0.7283805915031630	0.7	0.6577181208053690	75.41899441340780	58.490566037735800	0.696045197740113	0.6595744680851060	0.5254237288135590	47.45762711864410	86.66666666666670	13.333333333333300	52.54237288135590	72.62569832402240	73.22404371584700	0.7261548064918850	0.7204301075268820	0.7444444444444450	25.55555555555560	70.78651685393260	29.213483146067400	74.44444444444440
9		82.68156424581010	78.16901408450710	0.8138209829386300	0.8538461538461540	0.7207792207792210	87.70949720670390	77.55102040816330	0.8210382513661200	0.926829268292683	0.6666666666666670	33.33333333333330	97.54098360655740	2.459016393442620	66.66666666666670	77.6536312849162	78.49462365591400	0.7787276841840580	0.8202247191011240	0.7525773195876290	24.74226804123710	80.48780487804880	19.51219512195120	75.25773195876290
10		85.47486033519550	82.66666666666670	0.8486758339699520	0.8493150684931510	0.8051948051948050	88.26815642458100	82.05128205128200	0.8621848739495800	0.8421052631578950	0.8	20.0	92.43697478991600	7.563025210084030	80.0	82.68156424581010	83.06010928961750	0.827784730913642	0.8539325842696630	0.8085106382978720	19.14893617021280	84.70588235294120	15.294117647058800	80.85106382978720
11		83.24022346368710	78.72340425531920	0.8188426590148260	0.8604651162790700	0.7254901960784310	86.03351955307260	73.11827956989250	0.7969451931716080	0.85	0.6415094339622640	35.84905660377360	95.23809523809520	4.761904761904760	64.15094339622640	80.44692737430170	81.48148148148150	0.8090506329113920	0.8651685393258430	0.77	23.0	84.81012658227850	15.18987341772150	77.0
12		82.68156424581010	77.6978417266187	0.8115738375063870	0.8571428571428570	0.7105263157894740	86.03351955307260	77.06422018348620	0.8188385662684080	0.875	0.6885245901639340	31.147540983606600	94.91525423728810	5.084745762711870	68.85245901639340	79.3296089385475	78.10650887573970	0.7944555444555450	0.8461538461538460	0.7252747252747250	27.47252747252750	86.36363636363640	13.636363636363600	72.52747252747250
13		84.63687150837990	80.56537102473500	0.8361656758852420	0.8321167883211680	0.7808219178082190	87.70949720670390	78.0	0.8473643410852710	0.78	0.78	22.0	91.47286821705430	8.52713178294574	78.0	81.56424581005590	81.96721311475410	0.818335843373494	0.8620689655172410	0.78125	21.875	85.54216867469880	14.457831325301200	78.125
14		83.5195530726257	77.73584905660380	0.8227736006683380	0.7803030303030300	0.7744360902255640	85.47486033519550	73.46938775510210	0.8261605415860740	0.7058823529411770	0.7659574468085110	23.404255319148900	88.63636363636360	11.363636363636400	76.59574468085110	81.56424581005590	80.23952095808380	0.8142660665166290	0.8271604938271610	0.7790697674418610	22.09302325581400	84.94623655913980	15.053763440860200	77.90697674418610
15		81.28491620111730	74.52471482889730	0.792218191325947	0.8032786885245900	0.6950354609929080	88.26815642458100	76.92307692307690	0.8302197802197800	0.8333333333333330	0.7142857142857140	28.57142857142860	94.61538461538460	5.384615384615390	71.42857142857140	74.30167597765360	73.25581395348840	0.7446901549225390	0.7875	0.6847826086956520	31.52173913043480	80.45977011494250	19.54022988505750	68.47826086956520
16		77.93296089385480	72.08480565371030	0.7651392717938970	0.8095238095238100	0.6496815286624200	81.00558659217880	64.58333333333330	0.7373249299719890	0.8611111111111110	0.5166666666666670	48.333333333333300	95.7983193277311	4.201680672268910	51.66666666666670	74.86033519553070	75.93582887700530	0.7501257229067140	0.7888888888888890	0.7319587628865980	26.80412371134020	76.82926829268290	23.170731707317100	73.19587628865980
17		82.68156424581010	78.91156462585030	0.8188885689605530	0.8111888111888110	0.7682119205298010	88.26815642458100	79.61165048543690	0.8548606904906120	0.803921568627451	0.7884615384615380	21.153846153846200	92.1259842519685	7.874015748031500	78.84615384615380	77.09497206703910	78.53403141361260	0.7725378787878790	0.8152173913043480	0.7575757575757580	24.24242424242420	78.75	21.25	75.75757575757580
18		72.3463687150838	65.74394463667820	0.7123145587125970	0.76	0.5792682926829270	74.30167597765360	56.60377358490570	0.6825236167341430	0.7317073170731710	0.46153846153846200	53.84615384615390	90.35087719298250	9.649122807017540	46.15384615384620	70.39106145251400	71.03825136612020	0.7095328282828280	0.7738095238095240	0.6565656565656570	34.34343434343430	76.25	23.75	65.65656565656570
19		78.49162011173190	72.20216606498200	0.7683934530430640	0.819672131147541	0.6451612903225810	81.00558659217880	68.51851851851850	0.7599255583126550	0.8043478260869570	0.5967741935483870	40.32258064516130	92.3076923076923	7.6923076923076900	59.67741935483870	75.97765363128490	74.55621301775150	0.7631282820705180	0.8289473684210530	0.6774193548387100	32.25806451612900	84.88372093023260	15.11627906976740	67.74193548387100
20		81.28491620111730	76.32508833922260	0.7991787624140570	0.8372093023255810	0.7012987012987010	83.79888268156430	72.38095238095240	0.7887005649717510	0.8260869565217390	0.6440677966101690	35.59322033898310	93.33333333333330	6.666666666666670	64.40677966101690	78.77094972067040	78.65168539325840	0.7910401002506270	0.8433734939759040	0.7368421052631580	26.31578947368420	84.52380952380950	15.476190476190500	73.68421052631580
21		80.44692737430170	76.35135135135130	0.7964385365394860	0.875968992248062	0.6766467065868260	79.88826815642460	64.70588235294120	0.736302294197031	0.8918918918918920	0.5076923076923080	49.23076923076920	96.49122807017540	3.508771929824560	50.76923076923080	81.00558659217880	82.47422680412370	0.81423478482302	0.8695652173913040	0.7843137254901960	21.56862745098040	84.4155844155844	15.584415584415600	78.43137254901960
22		80.44692737430170	76.1904761904762	0.7944303797468360	0.8235294117647060	0.7088607594936710	83.24022346368710	73.68421052631580	0.7959746346843120	0.8076923076923080	0.6774193548387100	32.25806451612900	91.45299145299150	8.547008547008550	67.74193548387100	77.6536312849162	77.77777777777780	0.780245983935743	0.8333333333333330	0.7291666666666670	27.08333333333330	83.13253012048190	16.867469879518100	72.91666666666670
23		80.44692737430170	74.26470588235290	0.7869281468079610	0.8015873015873020	0.6917808219178080	81.56424581005590	67.32673267326730	0.7628148148148150	0.723404255319149	0.6296296296296300	37.03703703703700	89.60000000000000	10.4	62.96296296296300	79.3296089385475	78.36257309941520	0.7951649175412290	0.8481012658227850	0.7282608695652170	27.17391304347830	86.20689655172410	13.793103448275900	72.82608695652170
24		78.77094972067040	73.42657342657340	0.7746572734196500	0.8076923076923080	0.6730769230769230	81.56424581005590	70.27027027027030	0.7717810862972150	0.7959183673469390	0.6290322580645160	37.096774193548400	91.45299145299150	8.547008547008550	62.903225806451600	75.97765363128490	75.42857142857140	0.762828535669587	0.8148148148148150	0.7021276595744680	29.78723404255320	82.35294117647060	17.647058823529400	70.2127659574468
25		76.25698324022350	71.57190635451510	0.7540545637415140	0.7925925925925930	0.6524390243902440	77.09497206703910	60.19417475728160	0.7072649572649570	0.7560975609756100	0.5	50.0	91.45299145299150	8.547008547008550	50.0	75.41899441340780	77.55102040816330	0.7556659027247260	0.8085106382978720	0.7450980392156860	25.49019607843140	76.62337662337660	23.376623376623400	74.50980392156860
26		85.75418994413410	82.71186440677970	0.8513782051282050	0.8413793103448280	0.8133333333333330	87.70949720670390	78.84615384615380	0.8436296296296300	0.82	0.7592592592592590	24.074074074074100	92.80000000000000	7.200000000000000	75.92592592592590	83.79888268156430	84.81675392670160	0.8375376506024100	0.8526315789473680	0.84375	15.625	83.13253012048190	16.867469879518100	84.375
27		83.5195530726257	79.2982456140351	0.8231668369954010	0.849624060150376	0.743421052631579	87.70949720670390	77.55102040816330	0.8278518518518520	0.8636363636363640	0.7037037037037040	29.629629629629600	95.20000000000000	4.8	70.37037037037040	79.3296089385475	80.21390374331550	0.7962333081380700	0.8426966292134830	0.7653061224489800	23.46938775510200	82.71604938271610	17.28395061728400	76.53061224489800
28		81.28491620111730	77.59197324414720	0.8059142356129440	0.7945205479452050	0.7581699346405230	82.68156424581010	71.55963302752290	0.8004642108415690	0.6964285714285710	0.7358490566037740	26.41509433962260	86.5079365079365	13.492063492063500	73.58490566037740	79.88826815642460	81.05263157894740	0.8027215189873420	0.8555555555555560	0.77	23.0	83.54430379746840	16.455696202531600	77.0
29		79.3296089385475	72.38805970149250	0.779351012793177	0.7238805970149250	0.7238805970149250	85.47486033519550	74.50980392156860	0.8257364341085270	0.7307692307692310	0.76	24.0	89.14728682170540	10.852713178294600	76.0	73.18435754189940	71.0843373493976	0.7301378446115290	0.7195121951219510	0.7023809523809520	29.76190476190480	75.78947368421050	24.210526315789500	70.23809523809520
30		79.88826815642460	75.34246575342470	0.7912112712383530	0.7586206896551720	0.7482993197278910	83.79888268156430	70.70707070707070	0.7957364341085270	0.7142857142857140	0.7	30.0	89.14728682170540	10.852713178294600	70.0	75.97765363128490	77.72020725388600	0.7585491576565250	0.78125	0.7731958762886600	22.68041237113400	74.39024390243900	25.609756097561000	77.31958762886600
31		77.37430167597770	70.96774193548390	0.758072693855563	0.8114754098360660	0.6305732484076430	77.6536312849162	57.446808510638300	0.6980000000000000	0.675	0.5	50.0	89.60000000000000	10.4	50.0	77.09497206703910	77.83783783783780	0.7837250894225860	0.8780487804878050	0.6990291262135920	30.09708737864080	86.8421052631579	13.157894736842100	69.90291262135920
32		77.6536312849162	74.19354838709680	0.7714536176074640	0.8156028368794330	0.6804733727810650	81.56424581005590	72.26890756302520	0.782523616734143	0.7962962962962960	0.6615384615384620	33.84615384615390	90.35087719298250	9.649122807017540	66.15384615384620	73.74301675977650	75.39267015706810	0.7461538461538460	0.8275862068965520	0.6923076923076920	30.76923076923080	80.0	20.0	69.23076923076920
33		78.2122905027933	72.91666666666670	0.7708038382885010	0.84	0.6441717791411040	81.56424581005590	66.66666666666670	0.7504943502824860	0.825	0.559322033898305	44.06779661016950	94.16666666666670	5.833333333333330	55.932203389830500	74.86033519553070	76.1904761904762	0.7594871794871790	0.8470588235294120	0.6923076923076920	30.76923076923080	82.66666666666670	17.333333333333300	69.23076923076920
34		81.56424581005590	75.18796992481200	0.7952959421039030	0.8333333333333330	0.684931506849315	84.91620111731840	73.26732673267330	0.7941721288116270	0.8604651162790700	0.6379310344827590	36.206896551724100	95.0413223140496	4.958677685950410	63.793103448275900	78.2122905027933	76.36363636363640	0.7810314685314690	0.8181818181818180	0.7159090909090910	28.40909090909090	84.61538461538460	15.384615384615400	71.5909090909091
35		78.77094972067040	72.26277372262770	0.7722026604068860	0.75	0.6971830985915490	84.35754189944130	65.0	0.7500805931657000	0.7878787878787880	0.5531914893617020	44.680851063829800	94.6969696969697	5.303030303030300	55.319148936170200	73.18435754189940	75.25773195876290	0.7294486215538850	0.7373737373737370	0.7684210526315790	23.15789473684210	69.04761904761910	30.952380952381000	76.84210526315790
36		83.79888268156430	79.28571428571430	0.825	0.8409090909090910	0.75	85.47486033519550	72.91666666666670	0.8011053906723200	0.7954545454545450	0.6730769230769230	32.69230769230770	92.91338582677170	7.086614173228350	67.3076923076923	82.12290502793300	82.6086956521739	0.8235441767068270	0.8636363636363640	0.7916666666666670	20.83333333333330	85.54216867469880	14.457831325301200	79.16666666666670
37		82.12290502793300	77.62237762237760	0.8087658227848100	0.8671875	0.7025316455696200	86.59217877094970	78.94736842105260	0.8349541539316480	0.8490566037735850	0.7377049180327870	26.229508196721300	93.22033898305080	6.779661016949150	73.77049180327870	77.6536312849162	76.74418604651160	0.7853281367865230	0.88	0.6804123711340210	31.95876288659790	89.02439024390250	10.975609756097600	68.04123711340210
38		80.16759776536310	75.26132404181180	0.7897291773122130	0.8	0.7105263157894740	82.68156424581010	71.55963302752290	0.78662011969222	0.7647058823529410	0.6724137931034480	32.758620689655200	90.08264462809920	9.917355371900830	67.24137931034480	77.6536312849162	77.52808988764040	0.7787859824780980	0.8214285714285710	0.7340425531914890	26.59574468085110	82.35294117647060	17.647058823529400	73.40425531914890
39		82.9608938547486	78.29181494661920	0.8159722222222220	0.9090909090909090	0.6875	86.03351955307260	76.63551401869160	0.8135511441963060	0.9111111111111110	0.6612903225806450	33.87096774193550	96.58119658119660	3.418803418803420	66.12903225806450	79.88826815642460	79.3103448275862	0.8088309397833210	0.9078947368421050	0.7040816326530610	29.59183673469390	91.35802469135800	8.641975308641980	70.40816326530610
40		79.88826815642460	76.31578947368420	0.7929127602097570	0.7785234899328860	0.7483870967741940	84.91620111731840	78.4	0.8480336278141920	0.7313432835820900	0.8448275862068970	15.517241379310300	85.12396694214880	14.87603305785120	84.48275862068970	74.86033519553070	74.86033519553070	0.7538974101081220	0.8170731707317070	0.6907216494845360	30.92783505154640	81.70731707317070	18.29268292682930	69.0721649484536
41		80.72625698324020	75.26881720430110	0.7922784810126580	0.8677685950413220	0.6645569620253160	84.91620111731840	70.96774193548390	0.7835429769392030	0.825	0.6226415094339620	37.735849056603800	94.44444444444440	5.555555555555560	62.264150943396200	76.53631284916200	77.41935483870970	0.7820463320463320	0.8888888888888890	0.6857142857142860	31.42857142857140	87.83783783783780	12.162162162162200	68.57142857142860
42		75.13966480446930	68.32740213523130	0.736560159287001	0.7868852459016390	0.6037735849056600	77.6536312849162	59.183673469387800	0.7071847507331380	0.6744186046511630	0.5272727272727270	47.27272727272730	88.70967741935480	11.29032258064520	52.72727272727270	72.62569832402240	73.224043715847	0.7421153846153850	0.8481012658227850	0.6442307692307690	35.57692307692310	84.0	16.0	64.42307692307690
43		77.09497206703910	70.28985507246380	0.7559011476265000	0.7238805970149250	0.6830985915492960	77.6536312849162	58.333333333333300	0.7102325581395350	0.6086956521739130	0.56	44.0	86.04651162790700	13.953488372093000	56.00000000000000	76.53631284916200	76.66666666666670	0.7658045977011490	0.7840909090909090	0.75	25.0	78.16091954022990	21.839080459770100	75.0
44		84.91620111731840	81.25000000000000	0.8377737745330430	0.9069767441860470	0.7358490566037740	87.70949720670390	78.84615384615390	0.8350589588725910	0.8723404255319150	0.7192982456140350	28.07017543859650	95.08196721311480	4.918032786885250	71.9298245614035	82.12290502793300	82.60869565217390	0.8335879806468040	0.926829268292683	0.7450980392156860	25.49019607843140	92.20779220779220	7.792207792207790	74.50980392156860
45		75.97765363128490	69.50354609929080	0.7449377317235480	0.784	0.6242038216560510	76.53631284916200	58.00000000000000	0.6954094292803970	0.7631578947368420	0.46774193548387100	53.2258064516129	92.3076923076923	7.6923076923076900	46.774193548387100	75.41899441340780	75.82417582417580	0.7560150375939850	0.7931034482758620	0.7263157894736840	27.36842105263160	78.57142857142860	21.428571428571400	72.63157894736840
46		75.69832402234640	68.81720430107530	0.741919191919192	0.8067226890756300	0.6	78.77094972067040	57.77777777777780	0.7007407407407410	0.7222222222222220	0.48148148148148100	51.85185185185190	92.0	8.0	48.148148148148100	72.62569832402240	74.07407407407410	0.7411475833548720	0.8433734939759040	0.660377358490566	33.9622641509434	82.1917808219178	17.80821917808220	66.0377358490566
47		77.09497206703910	69.40298507462690	0.7502630320420850	0.808695652173913	0.6078431372549020	81.00558659217880	61.36363636363640	0.720448662640207	0.8709677419354840	0.47368421052631600	52.63157894736840	96.72131147540980	3.278688524590160	47.368421052631600	73.18435754189940	73.33333333333330	0.735316265060241	0.7857142857142860	0.6875	31.25	78.3132530120482	21.686746987951800	68.75
48		81.56424581005590	75.91240875912410	0.798871882625091	0.8888888888888890	0.6624203821656050	84.91620111731840	70.32967032967030	0.7780772686433060	0.8421052631578950	0.6037735849056600	39.62264150943400	95.23809523809520	4.761904761904760	60.37735849056600	78.2122905027933	78.68852459016390	0.7994871794871790	0.9113924050632910	0.6923076923076920	30.76923076923080	90.66666666666670	9.333333333333330	69.23076923076920
49		77.93296089385480	72.85223367697600	0.7704968243221460	0.7361111111111110	0.7210884353741500	79.88826815642460	64.70588235294120	0.7455555555555560	0.6875	0.6111111111111110	38.88888888888890	88.0	12.0	61.111111111111100	75.97765363128490	77.24867724867720	0.7587521880470120	0.7604166666666670	0.7849462365591400	21.50537634408600	73.25581395348840	26.744186046511600	78.49462365591400
50		81.84357541899440	76.36363636363640	0.8085510453479540	0.7608695652173910	0.7664233576642340	84.35754189944130	70.83333333333330	0.7903645833333330	0.7555555555555560	0.6666666666666670	33.33333333333330	91.40625	8.59375	66.66666666666670	79.3296089385475	79.3296089385475	0.7945111277819460	0.7634408602150540	0.8255813953488370	17.44186046511630	76.34408602150540	23.655913978494600	82.55813953488370
51		80.72625698324020	75.61837455830390	0.7941666666666670	0.8045112781954890	0.7133333333333330	83.79888268156430	70.10309278350520	0.7896124031007750	0.723404255319149	0.68	32.0	89.92248062015500	10.077519379845000	68.0	77.6536312849162	78.49462365591400	0.7827215189873420	0.8488372093023260	0.73	27.0	83.54430379746840	16.455696202531600	73.0
52		84.63687150837990	81.60535117056860	0.838320707070707	0.8776978417266190	0.7625	86.03351955307260	77.06422018348620	0.8188385662684080	0.875	0.6885245901639340	31.147540983606600	94.91525423728810	5.084745762711870	68.85245901639340	83.24022346368710	84.21052631578950	0.835290404040404	0.8791208791208790	0.8080808080808080	19.19191919191920	86.25	13.750000000000000	80.8080808080808
53		83.79888268156430	79.43262411347520	0.8252564102564100	0.8484848484848490	0.7466666666666670	87.70949720670390	78.43137254901960	0.8354105571847510	0.851063829787234	0.7272727272727270	27.27272727272730	94.35483870967740	5.64516129032258	72.72727272727270	79.88826815642460	80.0	0.8015664160401000	0.8470588235294120	0.7578947368421050	24.21052631578950	84.52380952380950	15.476190476190500	75.78947368421050
54		77.6536312849162	70.58823529411760	0.7575000000000000	0.7868852459016390	0.64	78.77094972067040	63.46153846153850	0.7287815126050420	0.75	0.55	45.0	90.75630252100840	9.243697478991600	55.00000000000000	76.53631284916200	75.0	0.7657303370786520	0.8076923076923080	0.7	30.0	83.14606741573030	16.853932584269700	70.0
55		81.00558659217880	75.53956834532370	0.7944238630556980	0.8333333333333330	0.6907894736842110	83.79888268156430	71.84466019417480	0.7859076660017100	0.8222222222222220	0.6379310344827590	36.206896551724100	93.38842975206610	6.6115702479338800	63.793103448275900	78.2122905027933	77.71428571428570	0.7852315394242800	0.8395061728395060	0.723404255319149	27.65957446808510	84.70588235294120	15.294117647058800	72.3404255319149
56		79.60893854748600	75.58528428093650	0.7874799003688870	0.8188405797101450	0.7018633540372670	83.79888268156430	72.38095238095240	0.7903961242519240	0.8085106382978720	0.6551724137931030	34.48275862068970	92.56198347107440	7.43801652892562	65.51724137931040	75.41899441340780	77.31958762886600	0.7588145120081760	0.8241758241758240	0.7281553398058250	27.18446601941750	78.94736842105260	21.052631578947400	72.81553398058250
57		77.6536312849162	70.1492537313433	0.7559700302885380	0.8173913043478260	0.6143790849673200	79.88826815642460	56.09756097560980	0.6931716082659480	0.7931034482758620	0.4339622641509430	56.60377358490570	95.23809523809520	4.761904761904760	43.39622641509430	75.41899441340780	76.34408602150540	0.7600632911392410	0.8255813953488370	0.71	29.00000000000000	81.0126582278481	18.9873417721519	71.0
58		78.77094972067040	72.85714285714290	0.7742593491986400	0.7555555555555560	0.7034482758620690	83.24022346368710	63.414634146341500	0.75453146374829	0.6666666666666670	0.6046511627906980	39.53488372093020	90.44117647058820	9.558823529411760	60.46511627906980	74.30167597765360	76.76767676767680	0.7426788897377130	0.7916666666666670	0.7450980392156860	25.49019607843140	74.02597402597400	25.97402597402600	74.50980392156860
59		81.28491620111730	76.32508833922260	0.7999038461538460	0.8120300751879700	0.72	83.79888268156430	67.41573033707870	0.7676609105180540	0.75	0.6122448979591840	38.775510204081600	92.3076923076923	7.6923076923076900	61.224489795918400	78.77094972067040	80.41237113402060	0.78998476770754	0.8387096774193550	0.7722772277227720	22.77227722772280	80.76923076923080	19.230769230769200	77.22772277227720
60		80.44692737430170	76.02739726027400	0.7937658227848100	0.8283582089552240	0.7025316455696200	85.47486033519550	75.00000000000000	0.8055084745762710	0.8666666666666670	0.6610169491525420	33.89830508474580	95.0	5.0	66.10169491525420	75.41899441340780	76.59574468085110	0.7573863636363640	0.8089887640449440	0.7272727272727270	27.27272727272730	78.75	21.25	72.72727272727270
61		85.47486033519550	82.5503355704698	0.8465914478050630	0.8848920863309350	0.7735849056603770	88.26815642458100	81.41592920353980	0.8539215686274510	0.8679245283018870	0.7666666666666670	23.333333333333300	94.11764705882350	5.88235294117647	76.66666666666670	82.68156424581010	83.24324324324320	0.8326388888888890	0.8953488372093020	0.7777777777777780	22.22222222222220	88.75	11.25	77.77777777777780
62		83.24022346368710	79.02097902097900	0.8203144894321370	0.8560606060606060	0.7337662337662340	88.8268156424581	81.48148148148150	0.8545169563978340	0.88	0.7586206896551720	24.137931034482800	95.0413223140496	4.958677685950410	75.86206896551720	77.6536312849162	77.52808988764050	0.7810617469879520	0.8414634146341460	0.71875	28.125	84.33734939759040	15.66265060240960	71.875
63		77.6536312849162	73.33333333333330	0.7680225024493540	0.7801418439716310	0.6918238993710690	79.3296089385475	64.76190476190480	0.738135593220339	0.7391304347826090	0.576271186440678	42.3728813559322	90.0	10.0	57.6271186440678	75.97765363128490	77.94871794871800	0.759746835443038	0.8	0.76	24.0	75.9493670886076	24.050632911392400	76.0
64		78.2122905027933	70.6766917293233	0.7609683281412250	0.7704918032786890	0.6527777777777780	84.35754189944130	65.85365853658540	0.7596436744034000	0.75	0.5869565217391310	41.30434782608700	93.23308270676690	6.7669172932330800	58.69565217391310	72.06703910614520	72.82608695652170	0.7245527840765940	0.7790697674418610	0.6836734693877550	31.63265306122450	76.5432098765432	23.456790123456800	68.36734693877550
65		82.9608938547486	76.62835249042150	0.8077898845340710	0.847457627118644	0.6993006993006990	83.79888268156430	68.81720430107530	0.7682962962962960	0.8205128205128210	0.5925925925925930	40.74074074074070	94.40000000000000	5.6000000000000000	59.25925925925930	82.12290502793300	80.95238095238100	0.8209113607990010	0.8607594936708860	0.7640449438202250	23.59550561797750	87.77777777777780	12.222222222222200	76.40449438202250
66		78.77094972067040	74.14965986394560	0.7775767961411530	0.7898550724637680	0.6987179487179490	85.47486033519550	75.47169811320760	0.8164924506387920	0.8	0.7142857142857140	28.57142857142860	91.869918699187	8.130081300813010	71.42857142857140	72.06703910614520	73.40425531914890	0.724746835443038	0.7840909090909090	0.69	31.0	75.9493670886076	24.050632911392400	69.0
67		79.88826815642460	75.0	0.7869238604532720	0.8059701492537310	0.7012987012987010	84.35754189944130	72.54901960784320	0.8014375561545370	0.7551020408163270	0.6981132075471700	30.18867924528300	90.47619047619050	9.523809523809520	69.81132075471700	75.41899441340780	76.34408602150540	0.7617415587712620	0.8352941176470590	0.7029702970297030	29.7029702970297	82.05128205128200	17.94871794871800	70.29702970297030
68		84.07821229050280	80.8080808080808	0.8332378405907820	0.8391608391608390	0.7792207792207790	84.35754189944130	72.00000000000000	0.798909751665657	0.75	0.6923076923076920	30.76923076923080	90.5511811023622	9.448818897637790	69.23076923076920	83.79888268156430	85.27918781725890	0.8403361344537820	0.8842105263157890	0.8235294117647060	17.64705882352940	85.71428571428570	14.285714285714300	82.35294117647060
69		75.69832402234640	71.09634551495020	0.7486142605190230	0.7697841726618710	0.6604938271604940	74.30167597765360	59.64912280701750	0.6959459459459460	0.7391304347826090	0.5	50.0	89.1891891891892	10.81081081081080	50.0	77.09497206703910	78.07486631016040	0.7706508135168960	0.7849462365591400	0.776595744680851	22.34042553191490	76.47058823529410	23.52941176470590	77.6595744680851
70		79.3296089385475	75.0	0.7841728905132690	0.7872340425531920	0.7161290322580650	87.15083798882680	80.34188034188030	0.8411005434782610	0.8867924528301890	0.734375	26.5625	94.78260869565220	5.217391304347830	73.4375	71.50837988826820	71.50837988826820	0.7152847152847150	0.7272727272727270	0.7032967032967030	29.67032967032970	72.72727272727270	27.27272727272730	70.32967032967030
71		81.84357541899440	78.54785478547860	0.812061403508772	0.8814814814814820	0.7083333333333330	84.91620111731840	76.92307692307690	0.8183497536945810	0.8333333333333330	0.7142857142857140	28.57142857142860	92.24137931034480	7.758620689655170	71.42857142857140	78.77094972067040	79.56989247311830	0.8050836550836550	0.9135802469135800	0.7047619047619050	29.52380952380950	90.54054054054050	9.45945945945946	70.47619047619050
72		79.05027932960890	73.11827956989250	0.7750961538461540	0.7906976744186050	0.68	82.68156424581010	67.36842105263160	0.7643852210781350	0.7441860465116280	0.6153846153846150	38.46153846153850	91.33858267716540	8.661417322834650	61.53846153846150	75.41899441340780	76.08695652173910	0.7583774250440920	0.813953488372093	0.7142857142857140	28.57142857142860	80.24691358024690	19.753086419753100	71.42857142857140
73		82.9608938547486	78.4452296819788	0.8183093142470260	0.8161764705882350	0.7551020408163270	84.91620111731840	71.57894736842100	0.8007849293563580	0.7391304347826090	0.6938775510204080	30.612244897959200	90.76923076923080	9.230769230769230	69.38775510204080	81.00558659217880	81.91489361702130	0.8126102292768960	0.8555555555555560	0.7857142857142860	21.42857142857140	83.9506172839506	16.049382716049400	78.57142857142860
74		81.56424581005590	77.3972602739726	0.8052487941101800	0.8308823529411770	0.7243589743589740	84.91620111731840	74.28571428571430	0.8013418079096050	0.8478260869565220	0.6610169491525420	33.89830508474580	94.16666666666670	5.833333333333330	66.10169491525420	78.2122905027933	79.14438502673800	0.7838823233593160	0.8222222222222220	0.7628865979381440	23.71134020618560	80.48780487804880	19.51219512195120	76.28865979381440
75		72.90502793296090	63.94052044609670	0.7096518987341770	0.7747747747747750	0.5443037974683540	74.30167597765360	46.51162790697680	0.6374633431085040	0.6451612903225810	0.36363636363636400	63.63636363636360	91.12903225806450	8.870967741935480	36.36363636363640	71.50837988826820	72.1311475409836	0.7282830863566680	0.825	0.6407766990291260	35.92233009708740	81.57894736842110	18.421052631578900	64.07766990291260
76		79.3296089385475	74.82993197278910	0.7830978793337760	0.8148148148148150	0.6918238993710690	82.68156424581010	65.1685393258427	0.7473500908540280	0.7837837837837840	0.5576923076923080	44.230769230769200	93.7007874015748	6.299212598425200	55.769230769230800	75.97765363128490	79.02439024390240	0.7604491173416410	0.826530612244898	0.7570093457943930	24.29906542056070	76.38888888888890	23.61111111111110	75.70093457943930
77		77.93296089385480	70.63197026022300	0.7591019417475730	0.811965811965812	0.625	81.00558659217880	63.043478260869600	0.7313782991202350	0.7837837837837840	0.5272727272727270	47.27272727272730	93.54838709677420	6.451612903225810	52.72727272727270	74.86033519553070	74.57627118644070	0.7548403319084740	0.825	0.6804123711340210	31.95876288659790	82.92682926829270	17.073170731707300	68.04123711340210
78		80.44692737430170	76.1904761904762	0.7953524886167370	0.8682170542635660	0.6787878787878790	83.24022346368710	70.58823529411770	0.7807781649245060	0.782608695652174	0.6428571428571430	35.714285714285700	91.869918699187	8.130081300813010	64.28571428571430	77.6536312849162	79.16666666666670	0.7986238532110090	0.9156626506024100	0.6972477064220180	30.27522935779820	90.0	10.0	69.72477064220180
79		81.56424581005590	79.375	0.812893675988914	0.8037974683544300	0.7839506172839510	86.59217877094970	80.64516129032260	0.8519437551695620	0.8064516129032260	0.8064516129032260	19.35483870967740	89.74358974358980	10.256410256410300	80.64516129032260	76.53631284916200	78.57142857142860	0.764746835443038	0.8020833333333330	0.77	23.0	75.9493670886076	24.050632911392400	77.0
80		77.37430167597770	71.57894736842110	0.7600667408231370	0.7846153846153850	0.6580645161290320	77.6536312849162	62.264150943396200	0.7255371660859470	0.66	0.5892857142857140	41.07142857142860	86.1788617886179	13.821138211382100	58.92857142857140	77.09497206703910	77.09497206703910	0.7797348484848490	0.8625	0.696969696969697	30.30303030303030	86.25	13.750000000000000	69.6969696969697
81		81.28491620111730	76.49122807017540	0.7998700763697440	0.8515625	0.6942675159235670	83.79888268156430	71.84466019417480	0.7859076660017100	0.8222222222222220	0.6379310344827590	36.206896551724100	93.38842975206610	6.6115702479338800	63.793103448275900	78.77094972067040	79.12087912087910	0.7948863636363640	0.8674698795180720	0.7272727272727270	27.27272727272730	86.25	13.750000000000000	72.72727272727270
82		81.56424581005590	78.1456953642384	0.8087907953726060	0.8740740740740740	0.7065868263473050	83.79888268156430	73.39449541284400	0.7973163841807910	0.8	0.6779661016949150	32.20338983050850	91.66666666666670	8.333333333333330	67.79661016949150	79.3296089385475	80.82901554404150	0.8118153364632240	0.9176470588235290	0.7222222222222220	27.77777777777780	90.14084507042250	9.859154929577460	72.22222222222220
83		81.56424581005590	75.91240875912410	0.7985897435897440	0.8387096774193550	0.6933333333333330	84.35754189944130	73.58490566037740	0.7990168139070960	0.8125	0.6724137931034480	32.758620689655200	92.56198347107440	7.43801652892562	67.24137931034480	78.77094972067040	77.38095238095240	0.7900424787606200	0.8552631578947370	0.7065217391304350	29.34782608695650	87.35632183908050	12.643678160919500	70.65217391304350
84		77.93296089385480	71.48014440433210	0.7625538020086080	0.7983870967741940	0.6470588235294120	80.44692737430170	60.67415730337080	0.7217371323529410	0.7105263157894740	0.5294117647058820	47.05882352941180	91.40625	8.59375	52.94117647058820	75.41899441340780	76.59574468085110	0.7620320855614970	0.8372093023255810	0.7058823529411770	29.41176470588240	81.81818181818180	18.181818181818200	70.58823529411770
85		76.53631284916200	70.42253521126760	0.7511876295233540	0.7633587786259540	0.6535947712418300	75.41899441340780	57.6923076923077	0.6911764705882350	0.6818181818181820	0.5	50.0	88.23529411764710	11.76470588235290	50.0	77.6536312849162	77.77777777777780	0.7775068767191800	0.8045977011494250	0.7526881720430110	24.73118279569890	80.23255813953490	19.767441860465100	75.26881720430110
86		81.56424581005590	77.70270270270270	0.8064688838498360	0.8582089552238810	0.7098765432098770	79.3296089385475	67.82608695652170	0.7508662046908320	0.8125	0.582089552238806	41.7910447761194	91.96428571428570	8.035714285714290	58.2089552238806	83.79888268156430	83.97790055248620	0.8404761904761900	0.8837209302325580	0.8	20.0	88.09523809523810	11.904761904761900	80.0
87		80.72625698324020	76.28865979381440	0.7962927846781080	0.8409090909090910	0.6981132075471700	83.79888268156430	74.3362831858407	0.7988505747126440	0.84	0.6666666666666670	33.33333333333330	93.10344827586210	6.896551724137930	66.66666666666670	77.6536312849162	77.52808988764050	0.7810617469879520	0.8414634146341460	0.71875	28.125	84.33734939759040	15.66265060240960	71.875
88		79.05027932960890	73.30960854092530	0.775625697433445	0.8046875	0.673202614379085	81.56424581005590	66.66666666666670	0.7525884383088870	0.7857142857142860	0.5789473684210530	42.10526315789470	92.62295081967210	7.377049180327870	57.89473684210530	76.53631284916200	76.92307692307690	0.7681977911646590	0.813953488372093	0.7291666666666670	27.08333333333330	80.72289156626510	19.27710843373490	72.91666666666670
89		77.09497206703910	73.54838709677420	0.765340909090909	0.76	0.7125	78.2122905027933	66.66666666666670	0.7493697478991600	0.6842105263157900	0.65	35.0	84.87394957983190	15.126050420168100	65.0	75.97765363128490	77.720207253886	0.7610759493670890	0.8064516129032260	0.75	25.0	77.21518987341770	22.78481012658230	75.0
90		82.12290502793300	77.46478873239440	0.808974358974359	0.8208955223880600	0.7333333333333330	86.59217877094970	77.35849056603770	0.8356296296296300	0.7884615384615380	0.7592592592592590	24.074074074074100	91.2	8.800000000000000	75.92592592592590	77.6536312849162	77.52808988764050	0.7810617469879520	0.8414634146341460	0.71875	28.125	84.33734939759040	15.66265060240960	71.875
91		79.05027932960890	75.08305647840530	0.784800204754135	0.7533333333333330	0.7483443708609270	81.56424581005590	69.1588785046729	0.7815962863132680	0.6851851851851850	0.6981132075471700	30.18867924528300	86.5079365079365	13.492063492063500	69.81132075471700	76.53631284916200	78.35051546391750	0.7642983119173600	0.7916666666666670	0.7755102040816330	22.44897959183670	75.30864197530870	24.691358024691400	77.55102040816330
92		83.79888268156430	80.13698630136990	0.8299038461538460	0.823943661971831	0.78	84.91620111731840	70.32967032967030	0.7913486005089060	0.7441860465116280	0.6666666666666670	33.33333333333330	91.6030534351145	8.396946564885500	66.66666666666670	82.68156424581010	84.57711442786070	0.8257575757575760	0.8585858585858590	0.8333333333333330	16.66666666666670	81.81818181818180	18.181818181818200	83.33333333333330
93		81.28491620111730	77.59197324414720	0.8044108837531920	0.8405797101449280	0.7204968944099380	83.24022346368710	71.69811320754720	0.7845338983050850	0.8085106382978720	0.6440677966101690	35.59322033898310	92.5	7.5	64.40677966101690	79.3296089385475	80.82901554404140	0.7979373567608860	0.8571428571428570	0.7647058823529410	23.52941176470590	83.11688311688310	16.883116883116900	76.47058823529410
94		82.68156424581010	77.53623188405800	0.8106785317018910	0.8842975206611570	0.6903225806451610	79.88826815642460	67.85714285714290	0.7523320895522390	0.8444444444444440	0.5671641791044780	43.28358208955220	93.75	6.25	56.71641791044780	85.47486033519550	84.14634146341460	0.8535839160839160	0.9078947368421050	0.7840909090909090	21.59090909090910	92.3076923076923	7.6923076923076900	78.4090909090909
95		80.16759776536310	76.0942760942761	0.7954625734562150	0.7635135135135140	0.7583892617449670	84.91620111731840	74.28571428571430	0.8131111111111110	0.7647058823529410	0.7222222222222220	27.77777777777780	90.4	9.6	72.22222222222220	75.41899441340780	77.08333333333330	0.7525689223057640	0.7628865979381440	0.7789473684210530	22.10526315789470	72.61904761904760	27.380952380952400	77.89473684210530
96		82.9608938547486	76.80608365019010	0.8093375065206050	0.8347107438016530	0.7112676056338030	87.70949720670390	74.4186046511628	0.8219734660033170	0.7804878048780490	0.7111111111111110	28.888888888888900	93.28358208955220	6.7164179104477600	71.11111111111110	78.2122905027933	77.96610169491530	0.7885969323610760	0.8625	0.711340206185567	28.86597938144330	86.58536585365850	13.414634146341500	71.1340206185567
97		81.00558659217880	75.88652482269500	0.7960146660290130	0.8294573643410850	0.6993464052287580	82.12290502793300	69.81132075471700	0.7718926553672320	0.7872340425531920	0.6271186440677970	37.28813559322030	91.66666666666670	8.333333333333330	62.71186440677970	79.88826815642460	79.54545454545460	0.8017521902377970	0.8536585365853660	0.7446808510638300	25.53191489361700	85.88235294117650	14.117647058823500	74.46808510638300
98		78.2122905027933	71.1111111111111	0.7643886655554470	0.7441860465116280	0.6808510638297870	79.88826815642460	62.5	0.7355238970588240	0.6666666666666670	0.5882352941176470	41.17647058823530	88.28125	11.71875	58.82352941176470	76.53631284916200	75.86206896551720	0.7655430711610490	0.7857142857142860	0.7333333333333330	26.66666666666670	79.7752808988764	20.224719101123600	73.33333333333330
99		80.44692737430170	76.51006711409400	0.7966004583651640	0.7916666666666670	0.7402597402597400	83.24022346368710	69.38775510204080	0.7825520833333330	0.723404255319149	0.6666666666666670	33.33333333333330	89.84375	10.15625	66.66666666666670	77.6536312849162	80.0	0.7765074092999490	0.8247422680412370	0.7766990291262140	22.33009708737860	77.63157894736840	22.36842105263160	77.66990291262140
	**Mean**	**80.3743016759777**	**75.3442554593868**	**79.13815481536110**	**81.6556427022318%**	**70.1393862557266000**	**83.18435754189940**	**70.21249239982200**	**78.02368682833150**	**78.6547296210368000**	**63.993615636079%**	**36.006384363921**	**92.0537580205839**	**7.94624197941607**	**63.993615636079**	**77.56424581005590**	**78.0713318417493**	**77.9769954782467000**	**83.2519920380509%**	**73.6930023214483%**	**26.3069976785517**	**82.26098863504520**	**17.739011364954800**	**73.6930023214483**
	**Standard Deviation**	2.83130285623444	3.7746403297207	2.9541649561058800	4.29774258360564%	4.98898544984493300%	3.55240249795660	6.99089770901459	4.71312611008020	6.7598717659091100%	9.10359650142748%	9.10359650142748	2.717760827221	2.717760827221	9.10359650142748	3.22612414962120	3.31490819618677	3.2359789424471300%	4.48458798072872%	4.33314596788736%	4.33314596788736	5.01884592691757	5.018845926917580	4.33314596788736
	**T Test Significance (Female to Male)**						**0.000004934164333**	**0.0000000000000212**	**46.3333740051835%**	**0.000187582136555943**	**0.00000000000124167**	**0.0000000000000042**	**0.000000000000111**	**0.0000000000000000**	**0.000000000000124**									

3.1.4 Balanced training data, with feature selection - Logistic Regression Classifier

		UB_Accuracy	UB_FScore	UB_ROC_AUC	UB_Precision	UB_Recall	UB_F_Accuracy	UB_F_FScore	UB_F_ROC_AUC	UB_F_Precision	UB_F_Recall	UB_F_FNR	UB_F_TNR	UB_F_FPR	UB_F_TPR	UB_M_Accuracy	UB_M_FScore	UB_M_ROC_AUC	UB_M_Precision	UB_M_Recall	UB_M_FNR	UB_M_TNR	UB_M_FPR	UB_M_TPR
0		75.69832402234640	63.59832635983270	0.718812301859659	0.775510204081633	0.539007092198582	78.77094972067040	54.76190476190480	0.68523510032944	0.741935483870968	0.433962264150943	56.60377358490570	93.65079365079360	6.349206349206350	43.39622641509430	72.62569832402240	68.38709677419360	0.724213286713287	0.791044776119403	0.602272727272727	39.77272727272730	84.61538461538460	15.38461538461540	60.22727272727270
1		70.6703910614525	62.89752650176680	0.695155622489960	0.760683760683761	0.536144578313253	73.74301675977650	53.46534653465350	0.666871921182266	0.710526315789474	0.428571428571429	57.14285714285710	90.51724137931040	9.482758620689660	42.85714285714290	67.59776536312850	68.13186813186810	0.689128768523250	0.784810126582278	0.601941747572816	39.80582524271850	77.63157894736840	22.36842105263160	60.19417475728160
2		73.74301675977650	65.69343065693430	0.721655328798186	0.803571428571429	0.555555555555556	77.09497206703910	57.73195876288660	0.695621468926554	0.736842105263158	0.474576271186441	52.54237288135590	91.66666666666670	8.333333333333330	47.45762711864410	70.39106145251400	70.05649717514120	0.722023505365355	0.837837837837838	0.601941747572816	39.80582524271850	84.21052631578950	15.78947368421050	60.19417475728160
3		72.3463687150838	62.3574144486692	0.702585253310578	0.788461538461538	0.515723270440252	77.09497206703910	53.93258426966290	0.677859237536657	0.705882352941177	0.436363636363636	56.36363636363640	91.93548387096770	8.064516129032260	43.63636363636360	67.59776536312850	66.66666666666670	0.698846153846154	0.828571428571429	0.557692307692308	44.23076923076920	84.0	16.0	55.76923076923080
4		76.25698324022350	65.3061224489796	0.729161281985009	0.808080808080808	0.547945205479452	83.79888268156430	65.88235294117650	0.754945054945055	0.777777777777778	0.571428571428571	42.85714285714290	93.84615384615380	6.153846153846150	57.14285714285710	68.71508379888270	65.00000000000000	0.700968066381695	0.825396825396825	0.536082474226804	46.39175257731960	86.58536585365850	13.41463414634150	53.60824742268040
5		72.90502793296090	61.66007905138340	0.708494572911751	0.866666666666667	0.478527607361963	77.09497206703910	48.10126582278480	0.655528640638359	0.904761904761905	0.327586206896552	67.24137931034480	98.34710743801650	1.652892561983470	32.75862068965520	68.71508379888270	67.816091954023	0.713384813384813	0.855072463768116	0.561904761904762	43.80952380952380	86.48648648648650	13.51351351351350	56.19047619047620
6		75.13966480446930	65.36964980544750	0.723991987593694	0.756756756756757	0.575342465753425	81.00558659217880	63.82978723404260	0.735336817653891	0.789473684210526	0.535714285714286	46.42857142857140	93.4959349593496	6.504065040650410	53.57142857142860	69.27374301675980	66.25766871165640	0.693258426966292	0.739726027397260	0.6	40.0	78.65168539325840	21.34831460674160	60.0
7		73.46368715083800	63.32046332046330	0.711395253034192	0.803921568627451	0.522292993630573	77.09497206703910	55.91397849462370	0.687691025284801	0.8125	0.426229508196721	57.37704918032790	94.91525423728810	5.084745762711870	42.62295081967210	69.83240223463690	67.46987951807230	0.707329317269076	0.8	0.583333333333333	41.66666666666670	83.13253012048190	16.86746987951810	58.33333333333330
8		72.3463687150838	56.76855895196510	0.676416337285902	0.714285714285714	0.471014492753623	75.41899441340780	46.34146341463410	0.639562743336328	0.655172413793103	0.358490566037736	64.15094339622640	92.06349206349210	7.936507936507940	35.84905660377360	69.27374301675980	62.58503401360540	0.685481852315394	0.741935483870968	0.541176470588235	45.88235294117650	82.97872340425530	17.02127659574470	54.11764705882350
9		72.90502793296090	60.72874493927130	0.694462647444299	0.700934579439252	0.535714285714286	75.41899441340780	48.83720930232560	0.653864503816794	0.552631578947369	0.4375	56.25	87.02290076335880	12.97709923664120	43.75	70.39106145251400	67.08074534161490	0.707271364317841	0.782608695652174	0.586956521739131	41.30434782608700	82.75862068965520	17.24137931034480	58.69565217391310
10		74.86033519553070	66.91176470588240	0.730107424660139	0.791304347826087	0.579617834394904	78.2122905027933	62.85714285714290	0.723881633787163	0.75	0.540983606557377	45.90163934426230	90.67796610169490	9.322033898305090	54.09836065573770	71.50837988826820	69.46107784431140	0.723770080321285	0.816901408450704	0.604166666666667	39.58333333333330	84.33734939759040	15.66265060240960	60.41666666666670
11		72.90502793296090	60.72874493927130	0.694795901772646	0.721153846153846	0.524475524475525	77.6536312849162	53.48837209302330	0.677846759539673	0.676470588235294	0.442307692307692	55.76923076923080	91.33858267716540	8.661417322834650	44.23076923076920	68.15642458100560	64.59627329192550	0.683441558441558	0.742857142857143	0.571428571428571	42.85714285714290	79.54545454545460	20.45454545454550	57.14285714285710
12		75.41899441340780	66.41221374045800	0.731384077546480	0.813084112149533	0.561290322580645	79.88826815642460	59.09090909090910	0.708740740740741	0.764705882352941	0.481481481481481	51.85185185185190	93.60000000000000	6.4	48.14814814814810	70.94972067039110	70.11494252873560	0.725057121096725	0.835616438356164	0.603960396039604	39.60396039603960	84.61538461538460	15.38461538461540	60.39603960396040
13		77.09497206703910	68.70229007633590	0.746989499373816	0.796460176991151	0.604026845637584	79.3296089385475	60.21505376344090	0.719822303921569	0.666666666666667	0.549019607843137	45.09803921568630	89.0625	10.9375	54.90196078431370	74.86033519553070	73.37278106508880	0.760770975056689	0.873239436619718	0.632653061224490	36.73469387755100	88.88888888888890	11.11111111111110	63.26530612244900
14		73.18435754189940	62.79069767441860	0.708210539658396	0.801980198019802	0.515923566878981	75.97765363128490	52.74725274725270	0.669542709232097	0.705882352941177	0.421052631578947	57.89473684210530	91.80327868852460	8.19672131147541	42.10526315789470	70.39106145251400	68.26347305389220	0.721708860759494	0.850746268656716	0.57	43.0	87.34177215189870	12.65822784810130	57.00000000000000
15		74.30167597765360	64.34108527131780	0.716119691119691	0.754545454545455	0.560810810810811	74.30167597765360	54.90196078431370	0.676722982032717	0.777777777777778	0.424242424242424	57.57575757575760	92.92035398230090	7.079646017699120	42.42424242424240	74.30167597765360	70.51282051282050	0.737427709328640	0.743243243243243	0.670731707317073	32.92682926829270	80.41237113402060	19.58762886597940	67.07317073170730
16		73.18435754189940	62.5	0.703603603603604	0.740740740740741	0.540540540540541	78.2122905027933	46.57534246575340	0.647844112769486	0.607142857142857	0.377777777777778	62.22222222222220	91.7910447761194	8.208955223880600	37.77777777777780	68.15642458100560	68.85245901639340	0.693983137455289	0.7875	0.611650485436893	38.83495145631070	77.63157894736840	22.36842105263160	61.16504854368930
17		72.62569832402240	61.11111111111110	0.702746212121212	0.836956521739131	0.48125	77.09497206703910	49.38271604938270	0.658246225319396	0.8	0.357142857142857	64.28571428571430	95.9349593495935	4.065040650406500	35.71428571428570	68.15642458100560	66.66666666666670	0.707371794871795	0.850746268656716	0.548076923076923	45.19230769230770	86.66666666666670	13.33333333333330	54.80769230769230
18		75.13966480446930	65.63706563706560	0.725589777195282	0.714285714285714	0.607142857142857	79.3296089385475	54.32098765432100	0.690258254331481	0.628571428571429	0.478260869565217	52.17391304347830	90.22556390977440	9.774436090225560	47.82608695652170	70.94972067039110	70.78651685393260	0.711576971214018	0.75	0.670212765957447	32.97872340425530	75.29411764705880	24.70588235294120	67.02127659574470
19		72.3463687150838	64.25992779783390	0.705815986016209	0.729508196721312	0.574193548387097	77.6536312849162	59.18367346938780	0.710092842168314	0.644444444444445	0.547169811320755	45.28301886792450	87.3015873015873	12.69841269841270	54.71698113207550	67.0391061452514	67.03910614525140	0.683728036669213	0.779220779220779	0.588235294117647	41.17647058823530	77.92207792207790	22.07792207792210	58.82352941176470
20		72.62569832402240	63.1578947368421	0.706944444444444	0.792452830188679	0.525	74.30167597765360	50.0	0.654626285079189	0.741935483870968	0.377049180327869	62.29508196721310	93.22033898305080	6.779661016949150	37.70491803278690	70.94972067039110	70.11494252873560	0.720580808080808	0.813333333333333	0.616161616161616	38.38383838383840	82.5	17.5	61.61616161616160
21		74.30167597765360	64.34108527131780	0.724698265023887	0.882978723404255	0.506097560975610	74.86033519553070	51.61290322580650	0.664614121510673	0.8	0.380952380952381	61.90476190476190	94.82758620689660	5.172413793103450	38.09523809523810	73.74301675977650	71.51515151515150	0.760027925869510	0.921875	0.584158415841584	41.58415841584160	93.58974358974360	6.41025641025641	58.41584158415840
22		74.02234636871510	62.34817813765180	0.709600153295861	0.810526315789474	0.506578947368421	75.97765363128490	50.57471264367820	0.660729552579082	0.758620689655172	0.379310344827586	62.06896551724140	94.21487603305790	5.785123966942150	37.93103448275860	72.06703910614520	68.75	0.727847309136421	0.833333333333333	0.585106382978723	41.48936170212770	87.05882352941180	12.94117647058820	58.51063829787230
23		73.74301675977650	61.78861788617890	0.704794322597219	0.783505154639175	0.510067114093960	78.77094972067040	53.65853658536580	0.679703703703704	0.785714285714286	0.407407407407407	59.25925925925930	95.20000000000000	4.8	40.74074074074070	68.71508379888270	65.85365853658540	0.694924812030075	0.782608695652174	0.568421052631579	43.15789473684210	82.14285714285710	17.85714285714290	56.84210526315790
24		74.58100558659220	64.03162055335970	0.720011424219345	0.835051546391753	0.519230769230769	81.00558659217880	63.82978723404260	0.736436950146628	0.769230769230769	0.545454545454545	45.45454545454550	92.74193548387100	7.258064516129030	54.54545454545450	68.15642458100560	64.15094339622640	0.707603452652958	0.879310344827586	0.504950495049505	49.50495049504950	91.02564102564100	8.974358974358970	50.49504950495050
25		73.74301675977650	64.39393939393940	0.719710555293377	0.841584158415842	0.521472392638037	76.53631284916200	58.00000000000000	0.695675971538041	0.783783783783784	0.460317460317460	53.96825396825400	93.10344827586210	6.896551724137930	46.03174603174600	70.94972067039110	68.29268292682930	0.729367088607595	0.875	0.56	44.0	89.87341772151900	10.12658227848100	56.00000000000000
26		75.41899441340780	64.51612903225810	0.724322943280531	0.833333333333333	0.526315789473684	79.3296089385475	56.47058823529410	0.694222222222222	0.774193548387097	0.444444444444444	55.55555555555560	94.40000000000000	5.600000000000000	44.44444444444440	71.50837988826820	68.71165644171780	0.730158730158730	0.861538461538462	0.571428571428571	42.85714285714290	88.88888888888890	11.11111111111110	57.14285714285710
27		73.18435754189940	61.60000000000000	0.700019384853968	0.740384615384615	0.527397260273973	75.97765363128490	52.74725274725280	0.669794721407625	0.666666666666667	0.436363636363636	56.36363636363640	90.32258064516130	9.67741935483871	43.63636363636360	70.39106145251400	66.66666666666670	0.705981518481519	0.779411764705882	0.582417582417583	41.75824175824180	82.95454545454550	17.04545454545450	58.24175824175830
28		74.02234636871510	61.72839506172840	0.708512673362028	0.833333333333333	0.490196078431373	78.2122905027933	55.17241379310340	0.686235394699345	0.827586206896552	0.413793103448276	58.62068965517240	95.86776859504130	4.132231404958680	41.37931034482760	69.83240223463690	65.38461538461540	0.708897243107769	0.836065573770492	0.536842105263158	46.31578947368420	88.09523809523810	11.90476190476190	53.68421052631580
29		73.18435754189940	62.20472440944880	0.7075	0.822916666666667	0.5	75.41899441340780	55.10204081632650	0.679802955665025	0.771428571428572	0.428571428571429	57.14285714285710	93.10344827586210	6.896551724137930	42.85714285714290	70.94972067039110	66.66666666666670	0.720112781954887	0.852459016393443	0.547368421052632	45.26315789473680	89.28571428571430	10.71428571428570	54.73684210526320
30		68.99441340782120	56.47058823529410	0.669576844423470	0.782608695652174	0.441717791411043	75.97765363128490	52.74725274725270	0.669542709232097	0.705882352941177	0.421052631578947	57.89473684210530	91.80327868852460	8.19672131147541	42.10526315789470	62.01117318435750	58.53658536585370	0.657921943654691	0.827586206896552	0.452830188679245	54.71698113207550	86.3013698630137	13.69863013698630	45.28301886792450
31		75.97765363128490	69.50354609929080	0.753507264489923	0.899082568807340	0.566473988439306	78.77094972067040	59.57446808510640	0.708321756043346	0.848484848484849	0.459016393442623	54.09836065573770	95.76271186440680	4.23728813559322	45.90163934426230	73.18435754189940	74.46808510638300	0.767723880597015	0.921052631578947	0.625	37.5	91.04477611940300	8.955223880597010	62.5
32		75.13966480446930	67.63636363636360	0.733989289222677	0.788135593220339	0.592356687898089	77.6536312849162	56.52173913043480	0.694881889763779	0.65	0.5	50.0	88.9763779527559	11.02362204724410	50.0	72.62569832402240	73.22404371584700	0.744723294723295	0.858974358974359	0.638095238095238	36.19047619047620	85.13513513513510	14.86486486486490	63.8095238095238
33		73.46368715083800	64.15094339622640	0.713486706594417	0.787037037037037	0.541401273885350	76.53631284916200	55.31914893617020	0.683053221288515	0.764705882352941	0.433333333333333	56.66666666666670	93.27731092436970	6.722689075630250	43.33333333333330	70.39106145251400	69.00584795321640	0.712660296706060	0.797297297297297	0.608247422680412	39.17525773195880	81.70731707317070	18.29268292682930	60.82474226804120
34		72.90502793296090	61.04417670682730	0.700184619302266	0.8	0.493506493506494	74.30167597765360	46.51162790697670	0.640324858757062	0.740740740740741	0.338983050847458	66.10169491525420	94.16666666666670	5.833333333333330	33.89830508474580	71.50837988826820	68.71165644171780	0.723308270676692	0.823529411764706	0.589473684210526	41.05263157894740	85.71428571428570	14.28571428571430	58.94736842105260
35		72.90502793296090	62.54826254826250	0.703730272596844	0.764150943396227	0.529411764705882	75.97765363128490	55.6701030927835	0.683175882189497	0.75	0.442622950819672	55.73770491803280	92.37288135593220	7.627118644067800	44.26229508196720	69.83240223463690	66.66666666666670	0.701524237881059	0.771428571428572	0.586956521739131	41.30434782608700	81.60919540229890	18.39080459770120	58.69565217391310
36		73.46368715083800	63.03501945525290	0.710110434120335	0.801980198019802	0.519230769230769	78.2122905027933	60.60606060606060	0.712635869565217	0.857142857142857	0.46875	53.125	95.65217391304350	4.347826086956520	46.875	68.71508379888270	64.55696202531650	0.690967016491754	0.772727272727273	0.554347826086957	44.56521739130430	82.75862068965520	17.24137931034480	55.43478260869570
37		70.39106145251400	56.91056910569110	0.674082313681869	0.769230769230769	0.451612903225806	73.74301675977650	44.70588235294120	0.628372434017595	0.633333333333333	0.345454545454545	65.45454545454550	91.12903225806450	8.870967741935480	34.54545454545460	67.0391061452514	63.35403726708080	0.691708860759494	0.836065573770492	0.51	49.0	87.34177215189870	12.65822784810130	51.0
38		71.7877094972067	61.30268199233720	0.695664556962025	0.776699029126214	0.506329113924051	73.18435754189940	47.82608695652170	0.643231320650676	0.733333333333333	0.354838709677419	64.51612903225810	93.16239316239320	6.837606837606840	35.48387096774190	70.39106145251400	68.63905325443790	0.711721887550201	0.794520547945205	0.604166666666667	39.58333333333330	81.92771084337350	18.07228915662650	60.41666666666670
39		71.50837988826820	57.50000000000000	0.677821839636329	0.741935483870968	0.469387755102041	72.06703910614520	37.5	0.594888888888889	0.576923076923077	0.277777777777778	72.22222222222220	91.2	8.800000000000000	27.77777777777780	70.94972067039110	67.5	0.714741185296324	0.805970149253731	0.580645161290323	41.93548387096770	84.88372093023260	15.11627906976740	58.06451612903230
40		75.41899441340780	64.22764227642280	0.721019643318687	0.79	0.541095890410959	79.88826815642460	57.14285714285710	0.700137867647059	0.727272727272727	0.470588235294118	52.94117647058820	92.96875	7.03125	47.05882352941180	70.94972067039110	67.90123456790120	0.718045112781955	0.820895522388060	0.578947368421053	42.10526315789470	85.71428571428570	14.28571428571430	57.89473684210530
41		72.3463687150838	61.47859922178990	0.700000000000000	0.797979797979798	0.5	77.09497206703910	53.93258426966290	0.678222222222222	0.685714285714286	0.444444444444444	55.55555555555560	91.2	8.800000000000000	44.44444444444440	67.59776536312850	65.47619047619050	0.704423076923077	0.859375	0.528846153846154	47.11538461538460	88.0	12.0	52.88461538461540
42		73.46368715083800	62.15139442231080	0.703076323987539	0.728971962616822	0.541666666666667	79.88826815642460	52.63157894736840	0.678675048355900	0.689655172413793	0.425531914893617	57.44680851063830	93.18181818181820	6.818181818181820	42.5531914893617	67.0391061452514	66.28571428571430	0.677017852652753	0.743589743589744	0.597938144329897	40.20618556701030	75.60975609756100	24.39024390243900	59.79381443298970
43		73.74301675977650	64.92537313432840	0.722985692771084	0.852941176470588	0.524096385542169	77.6536312849162	55.55555555555560	0.687968880244512	0.862068965517241	0.409836065573771	59.01639344262300	96.61016949152540	3.389830508474580	40.98360655737710	69.83240223463690	69.6629213483146	0.720913770913771	0.849315068493151	0.590476190476191	40.95238095238100	85.13513513513510	14.86486486486490	59.04761904761910
44		69.55307262569830	57.25490196078430	0.668981407913555	0.73	0.470967741935484	70.39106145251400	44.21052631578950	0.616176470588235	0.6	0.35	65.0	88.23529411764710	11.76470588235290	35.0	68.71508379888270	65.0	0.696303258145363	0.8	0.547368421052632	45.26315789473680	84.52380952380950	15.47619047619050	54.73684210526320
45		75.41899441340780	63.93442622950820	0.720656370656371	0.8125	0.527027027027027	77.6536312849162	52.38095238095240	0.671703703703704	0.733333333333333	0.407407407407407	59.25925925925930	93.60000000000000	6.4	40.74074074074070	73.18435754189940	70.0	0.739048811013767	0.848484848484849	0.595744680851064	40.42553191489360	88.23529411764710	11.76470588235290	59.57446808510640
46		70.11173184357540	57.70750988142290	0.678484724280354	0.793478260869565	0.453416149068323	74.30167597765360	50.0	0.655569341053212	0.766666666666667	0.370967741935484	62.90322580645160	94.01709401709400	5.982905982905980	37.09677419354840	65.92178770949720	62.11180124223600	0.677525252525253	0.806451612903226	0.505050505050505	49.49494949494950	85.0	15.0	50.50505050505050
47		74.30167597765360	61.98347107438020	0.708140283140283	0.797872340425532	0.506756756756757	75.97765363128490	50.57471264367820	0.659928122192273	0.647058823529412	0.415094339622642	58.49056603773580	90.47619047619050	9.523809523809520	41.50943396226420	72.62569832402240	68.38709677419360	0.737280701754386	0.883333333333333	0.557894736842105	44.21052631578950	91.66666666666670	8.333333333333330	55.78947368421050
48		69.83240223463690	54.62184873949580	0.657937435098650	0.691489361702128	0.451388888888889	72.06703910614520	37.50000000000000	0.592371323529412	0.517241379310345	0.294117647058824	70.58823529411770	89.0625	10.9375	29.41176470588240	67.59776536312850	63.29113924050630	0.681607901975494	0.769230769230769	0.537634408602151	46.23655913978490	82.55813953488370	17.44186046511630	53.76344086021510
49		73.46368715083800	61.84738955823290	0.703782051282051	0.777777777777778	0.513333333333333	75.41899441340780	53.19148936170210	0.671019727702140	0.757575757575758	0.409836065573771	59.01639344262300	93.22033898305080	6.779661016949150	40.98360655737710	71.50837988826820	67.0967741935484	0.714357053682897	0.787878787878788	0.584269662921348	41.57303370786520	84.44444444444440	15.55555555555560	58.42696629213480
50		75.13966480446930	64.25702811244980	0.720609485886773	0.8	0.536912751677852	76.53631284916200	53.33333333333330	0.673826979472141	0.685714285714286	0.436363636363636	56.36363636363640	91.12903225806450	8.870967741935480	43.63636363636360	73.74301675977650	70.44025157232700	0.744931163954944	0.861538461538462	0.595744680851064	40.42553191489360	89.41176470588240	10.58823529411760	59.57446808510640
51		69.55307262569830	55.87044534412960	0.668354430379747	0.775280898876405	0.436708860759494	72.62569832402240	42.35294117647060	0.622117254792998	0.75	0.295081967213115	70.49180327868850	94.91525423728810	5.084745762711870	29.50819672131150	66.4804469273743	62.96296296296300	0.677520744279608	0.784615384615385	0.525773195876289	47.42268041237110	82.92682926829270	17.07317073170730	52.57731958762890
52		70.6703910614525	55.69620253164560	0.666553343046787	0.717391304347826	0.455172413793103	74.86033519553070	40.0	0.610753532182104	0.576923076923077	0.306122448979592	69.38775510204080	91.53846153846150	8.461538461538460	30.61224489795920	66.4804469273743	62.96296296296300	0.675263554216868	0.772727272727273	0.53125	46.875	81.92771084337350	18.07228915662650	53.125
53		72.62569832402240	62.01550387596900	0.701954810865702	0.784313725490196	0.512820512820513	77.09497206703910	57.73195876288660	0.695621468926554	0.736842105263158	0.474576271186441	52.54237288135590	91.66666666666670	8.333333333333330	47.45762711864410	68.15642458100560	64.59627329192550	0.694870505406085	0.8125	0.536082474226804	46.39175257731960	85.36585365853660	14.63414634146340	53.60824742268040
54		77.6536312849162	68.0	0.746607378129117	0.758928571428571	0.615942028985507	79.88826815642460	60.86956521739130	0.723728553921569	0.682926829268293	0.549019607843137	45.09803921568630	89.84375	10.15625	54.90196078431370	75.41899441340780	72.15189873417720	0.751499250374813	0.802816901408451	0.655172413793103	34.48275862068970	84.78260869565220	15.21739130434780	65.51724137931040
55		75.41899441340780	65.89147286821710	0.728997289972900	0.809523809523810	0.555555555555556	79.3296089385475	60.21505376344090	0.714222873900293	0.736842105263158	0.509090909090909	49.09090909090910	91.93548387096770	8.064516129032260	50.90909090909090	71.50837988826820	69.09090909090910	0.729087931468884	0.850746268656716	0.581632653061225	41.83673469387760	87.65432098765430	12.34567901234570	58.16326530612250
56		75.69832402234640	66.14785992217900	0.731061573837506	0.809523809523810	0.559210526315790	79.88826815642460	60.86956521739130	0.717479674796748	0.777777777777778	0.5	50.0	93.4959349593496	6.504065040650410	50.0	71.50837988826820	69.09090909090910	0.724585843373494	0.826086956521739	0.59375	40.625	85.54216867469880	14.45783132530120	59.375
57		76.25698324022350	65.86345381526100	0.730983904465213	0.780952380952381	0.569444444444444	77.6536312849162	55.55555555555560	0.688230008984726	0.675675675675676	0.471698113207547	52.83018867924530	90.47619047619050	9.523809523809520	47.16981132075470	74.86033519553070	71.69811320754720	0.750686813186813	0.838235294117647	0.626373626373626	37.36263736263740	87.5	12.5	62.63736263736260
58		75.69832402234640	66.66666666666670	0.734291698400609	0.828571428571429	0.557692307692308	78.77094972067040	50.0	0.663108320251177	0.703703703703704	0.387755102040816	61.22448979591840	93.84615384615380	6.153846153846150	38.77551020408160	72.62569832402240	73.5135135135135	0.74831256490135	0.871794871794872	0.635514018691589	36.44859813084110	86.11111111111110	13.88888888888890	63.55140186915890
59		68.43575418994410	57.99256505576210	0.667468931886110	0.735849056603774	0.478527607361963	72.06703910614520	45.65217391304350	0.627315474494158	0.617647058823529	0.362068965517241	63.79310344827590	89.25619834710740	10.74380165289260	36.20689655172410	64.80446927374300	64.40677966101690	0.67007722007722	0.791666666666667	0.542857142857143	45.71428571428570	79.72972972972970	20.27027027027030	54.28571428571430
60		69.83240223463690	57.48031496062990	0.669258086188694	0.708737864077670	0.483443708609272	72.62569832402240	46.15384615384620	0.633995554320645	0.7	0.344262295081967	65.57377049180330	92.37288135593220	7.627118644067800	34.42622950819670	67.0391061452514	63.80368098159510	0.670911360799001	0.712328767123288	0.577777777777778	42.22222222222220	76.40449438202250	23.59550561797750	57.77777777777780
61		73.18435754189940	60.33057851239670	0.694779638965686	0.737373737373737	0.510489510489511	75.41899441340780	40.54054054054050	0.614119922630561	0.555555555555556	0.319148936170213	68.08510638297870	90.9090909090909	9.090909090909090	31.91489361702130	70.94972067039110	69.04761904761910	0.717745983935743	0.805555555555556	0.604166666666667	39.58333333333330	83.13253012048190	16.86746987951810	60.41666666666670
62		74.58100558659220	65.39923954372620	0.721508307372794	0.722689075630252	0.597222222222222	81.56424581005590	61.17647058823530	0.722440944881890	0.787878787878788	0.5	50.0	94.48818897637800	5.511811023622050	50.0	67.59776536312850	67.41573033707870	0.676661669165417	0.697674418604651	0.652173913043478	34.78260869565220	70.11494252873560	29.88505747126440	65.21739130434780
63		76.25698324022350	68.40148698884760	0.742587001430161	0.807017543859649	0.593548387096774	78.2122905027933	58.06451612903230	0.701099706744868	0.710526315789474	0.490909090909091	50.90909090909090	91.12903225806450	8.870967741935480	49.09090909090910	74.30167597765360	73.86363636363640	0.755379746835443	0.855263157894737	0.65	35.0	86.07594936708860	13.92405063291140	65.0
64		75.97765363128490	63.86554621848740	0.721507106462859	0.716981132075472	0.575757575757576	77.6536312849162	52.38095238095240	0.674489795918367	0.628571428571429	0.448979591836735	55.10204081632650	90.0	10.0	44.89795918367350	74.30167597765360	70.12987012987010	0.736759538152610	0.760563380281690	0.650602409638554	34.93975903614460	82.29166666666670	17.70833333333330	65.06024096385540
65		72.3463687150838	60.24096385542170	0.693878527020564	0.78125	0.490196078431373	75.97765363128490	51.68539325842700	0.665748587570622	0.766666666666667	0.389830508474576	61.01694915254240	94.16666666666670	5.833333333333330	38.98305084745760	68.71508379888270	65.0	0.694242803504381	0.787878787878788	0.553191489361702	44.68085106382980	83.52941176470590	16.47058823529410	55.31914893617020
66		74.58100558659220	66.9090909090909	0.732837600401607	0.844036697247707	0.554216867469880	77.09497206703910	60.19417475728160	0.708299812282113	0.837837837837838	0.469696969696970	53.03030303030300	94.69026548672570	5.309734513274340	46.96969696969700	72.06703910614520	70.93023255813950	0.735379746835443	0.847222222222222	0.61	39.0	86.07594936708860	13.92405063291140	61.0
67		73.74301675977650	63.56589147286820	0.715148067380930	0.828282828282828	0.515723270440252	73.18435754189940	47.82608695652170	0.641316526610644	0.6875	0.366666666666667	63.33333333333330	91.59663865546220	8.403361344537820	36.66666666666670	74.30167597765360	72.28915662650600	0.759280303030303	0.895522388059702	0.606060606060606	39.39393939393940	91.25	8.75	60.60606060606060
68		73.74301675977650	60.83333333333330	0.701383526383526	0.793478260869565	0.493243243243243	75.41899441340780	50.0	0.655645161290323	0.666666666666667	0.4	60.0	91.12903225806450	8.870967741935480	40.0	72.06703910614520	67.10526315789470	0.72768192048012	0.864406779661017	0.548387096774194	45.16129032258060	90.69767441860470	9.30232558139535	54.83870967741940
69		72.90502793296090	61.96078431372550	0.699539200415369	0.711711711711712	0.548611111111111	77.6536312849162	47.36842105263160	0.651492537313433	0.580645161290323	0.4	60.0	90.29850746268660	9.701492537313430	40.0	68.15642458100560	68.15642458100560	0.689330808080808	0.7625	0.616161616161616	38.38383838383840	76.25	23.75	61.61616161616160
70		69.55307262569830	56.57370517928290	0.668900719333270	0.755319148936170	0.452229299363057	70.94972067039110	46.93877551020410	0.631861413043478	0.676470588235294	0.359375	64.0625	90.43478260869570	9.565217391304350	35.9375	68.15642458100560	62.74509803921570	0.688297074268567	0.8	0.516129032258065	48.38709677419360	86.04651162790700	13.95348837209300	51.61290322580650
71		72.06703910614520	58.33333333333330	0.683592868426992	0.752688172043011	0.476190476190476	76.53631284916200	54.34782608695650	0.678531073446328	0.757575757575758	0.423728813559322	57.6271186440678	93.33333333333330	6.666666666666670	42.3728813559322	67.59776536312850	60.81081081081080	0.673264235764236	0.75	0.511363636363636	48.86363636363640	83.51648351648350	16.48351648351650	51.13636363636360
72		72.06703910614520	61.83206106870230	0.698829113924051	0.778846153846154	0.512658227848101	75.41899441340780	46.34146341463420	0.640469208211144	0.703703703703704	0.345454545454545	65.45454545454550	93.54838709677420	6.451612903225810	34.54545454545460	68.71508379888270	68.88888888888890	0.702286663260092	0.805194805194805	0.601941747572816	39.80582524271850	80.26315789473690	19.73684210526320	60.19417475728160
73		70.94972067039110	60.90225563909770	0.688310042145958	0.743119266055046	0.515923566878981	77.09497206703910	46.75324675324680	0.645515267175573	0.620689655172414	0.375	62.5	91.6030534351145	8.396946564885500	37.5	64.80446927374300	66.66666666666670	0.667562254259502	0.7875	0.577981651376147	42.20183486238530	75.71428571428570	24.28571428571430	57.79816513761470
74		74.02234636871510	66.4259927797834	0.728328682948240	0.836363636363636	0.550898203592814	76.53631284916200	58.00000000000000	0.695332036676855	0.743589743589744	0.475409836065574	52.45901639344260	91.52542372881360	8.47457627118644	47.54098360655740	71.50837988826820	71.1864406779661	0.742375290772809	0.887323943661972	0.594339622641509	40.56603773584910	89.04109589041100	10.95890410958900	59.43396226415090
75		70.39106145251400	60.74074074074080	0.686759889140842	0.759259259259259	0.506172839506173	72.62569832402240	51.48514851485150	0.654121863799283	0.666666666666667	0.419354838709677	58.06451612903230	88.88888888888890	11.11111111111110	41.93548387096770	68.15642458100560	66.27218934911240	0.697721518987342	0.811594202898551	0.56	44.0	83.54430379746840	16.45569620253160	56.00000000000000
76		71.7877094972067	61.00386100386100	0.692768016297428	0.752380952380952	0.512987012987013	77.09497206703910	57.73195876288660	0.695518207282913	0.756756756756757	0.466666666666667	53.33333333333330	92.43697478991600	7.563025210084030	46.66666666666670	66.4804469273743	62.96296296296300	0.671276595744681	0.75	0.542553191489362	45.74468085106380	80.0	20.0	54.25531914893620
77		70.94972067039110	63.38028169014090	0.696145124716553	0.737704918032787	0.555555555555556	73.18435754189940	54.71698113207550	0.670238095238095	0.630434782608696	0.483333333333333	51.66666666666670	85.71428571428570	14.28571428571430	48.33333333333330	68.71508379888270	68.53932584269660	0.701617010440540	0.802631578947369	0.598039215686275	40.19607843137260	80.51948051948050	19.48051948051950	59.80392156862750
78		71.7877094972067	59.43775100401610	0.690149784209190	0.795698924731183	0.474358974358974	77.09497206703910	52.87356321839080	0.673065861374748	0.766666666666667	0.403508771929825	59.64912280701750	94.26229508196720	5.737704918032790	40.35087719298250	66.4804469273743	62.96296296296300	0.682575757575758	0.809523809523810	0.515151515151515	48.48484848484850	85.0	15.0	51.51515151515150
79		74.58100558659220	64.86486486486490	0.720490546755238	0.785046728971963	0.552631578947369	81.00558659217880	61.36363636363640	0.727364341085271	0.710526315789474	0.54	46.0	91.47286821705430	8.527131782945740	54.0	68.15642458100560	66.66666666666670	0.701489686783804	0.826086956521739	0.558823529411765	44.11764705882350	84.4155844155844	15.58441558441560	55.88235294117650
80		70.39106145251400	59.84848484848480	0.685921031933302	0.782178217821782	0.484662576687117	74.86033519553070	52.63157894736840	0.666031073446328	0.694444444444444	0.423728813559322	57.6271186440678	90.83333333333330	9.166666666666670	42.3728813559322	65.92178770949720	63.90532544378700	0.686282051282051	0.830769230769231	0.519230769230769	48.07692307692310	85.33333333333330	14.66666666666670	51.92307692307690
81		74.02234636871510	64.90566037735850	0.725181977911647	0.868686868686869	0.518072289156627	72.06703910614520	52.83018867924530	0.666407061266874	0.823529411764706	0.388888888888889	61.11111111111110	94.39252336448600	5.607476635514020	38.88888888888890	75.97765363128490	72.95597484276730	0.767334167709637	0.892307692307692	0.617021276595745	38.29787234042550	91.76470588235290	8.235294117647060	61.70212765957450
82		72.62569832402240	62.01550387596900	0.700916730328495	0.769230769230769	0.519480519480519	75.41899441340780	53.19148936170210	0.670197740112994	0.714285714285714	0.423728813559322	57.6271186440678	91.66666666666670	8.333333333333330	42.3728813559322	69.83240223463690	67.07317073170730	0.706140350877193	0.797101449275362	0.578947368421053	42.10526315789470	83.33333333333330	16.66666666666670	57.89473684210530
83		78.2122905027933	69.53125000000000	0.755930537352556	0.767241379310345	0.635714285714286	78.77094972067040	56.81818181818180	0.697077528770442	0.694444444444444	0.480769230769231	51.92307692307690	91.33858267716540	8.661417322834650	48.07692307692310	77.6536312849162	76.1904761904762	0.775724275724276	0.8	0.727272727272727	27.27272727272730	82.41758241758240	17.58241758241760	72.72727272727270
84		73.18435754189940	62.7906976744186	0.710195707070707	0.826530612244898	0.50625	77.6536312849162	54.54545454545450	0.686165845648604	0.96	0.380952380952381	61.90476190476190	99.13793103448280	0.862068965517241	38.09523809523810	68.71508379888270	67.05882352941180	0.696253457379935	0.780821917808219	0.587628865979382	41.23711340206190	80.48780487804880	19.51219512195120	58.76288659793820
85		74.58100558659220	67.38351254480290	0.738309577394349	0.878504672897196	0.546511627906977	78.77094972067040	61.22448979591840	0.716240622395110	0.810810810810811	0.491803278688525	50.81967213114750	94.0677966101695	5.932203389830510	49.18032786885250	70.39106145251400	70.71823204419890	0.744170641229465	0.914285714285714	0.576576576576577	42.34234234234230	91.17647058823530	8.823529411764710	57.65765765765770
86		74.58100558659220	64.31372549019610	0.717047422949962	0.719298245614035	0.581560283687943	77.09497206703910	51.76470588235300	0.669612403100775	0.628571428571429	0.44	56.00000000000000	89.92248062015500	10.07751937984500	44.0	72.06703910614520	70.58823529411770	0.721715784215784	0.759493670886076	0.659340659340659	34.06593406593410	78.40909090909090	21.59090909090910	65.93406593406590
87		74.02234636871510	62.34817813765180	0.707402392236515	0.77	0.523809523809524	76.53631284916200	50.0	0.658430667864630	0.677419354838710	0.396226415094340	60.37735849056600	92.06349206349210	7.936507936507940	39.62264150943400	71.50837988826820	68.71165644171780	0.721401752190238	0.811594202898551	0.595744680851064	40.42553191489360	84.70588235294120	15.29411764705880	59.57446808510640
88		74.58100558659220	61.92468619246860	0.707111808347224	0.755102040816327	0.524822695035461	79.3296089385475	59.34065934065930	0.708153580672994	0.794117647058824	0.473684210526316	52.63157894736840	94.26229508196720	5.737704918032790	47.36842105263160	69.83240223463690	63.51351351351350	0.690288220551379	0.734375	0.559523809523810	44.04761904761900	82.10526315789470	17.89473684210530	55.95238095238100
89		73.18435754189940	64.17910447761190	0.709588159588160	0.716666666666667	0.581081081081081	77.6536312849162	55.55555555555560	0.689203513022411	0.657894736842105	0.480769230769231	51.92307692307690	89.76377952755910	10.23622047244090	48.07692307692310	68.71508379888270	68.53932584269660	0.691202309236948	0.743902439024390	0.635416666666667	36.45833333333330	74.69879518072290	25.30120481927710	63.54166666666670
90		72.62569832402240	58.47457627118640	0.685444787770369	0.741935483870968	0.482517482517483	78.77094972067040	56.81818181818180	0.694759001161440	0.78125	0.446428571428571	55.35714285714290	94.3089430894309	5.691056910569110	44.64285714285710	66.4804469273743	59.45945945945950	0.660482258870565	0.721311475409836	0.505747126436782	49.42528735632180	81.52173913043480	18.47826086956520	50.57471264367820
91		72.06703910614520	60.62992125984250	0.696912234126608	0.810526315789474	0.484276729559748	79.3296089385475	49.31506849315070	0.660782442748092	0.72	0.375	62.5	94.65648854961830	5.343511450381680	37.5	64.80446927374300	65.19337016574590	0.684883412824589	0.842857142857143	0.531531531531532	46.84684684684680	83.82352941176470	16.17647058823530	53.15315315315320
92		69.55307262569830	56.57370517928290	0.666793990323402	0.731958762886598	0.461038961038961	78.2122905027933	51.85185185185190	0.670335429769392	0.75	0.396226415094340	60.37735849056600	94.44444444444440	5.555555555555560	39.62264150943400	60.89385474860330	58.82352941176470	0.625729880680376	0.724637681159420	0.495049504950495	50.49504950495050	75.64102564102560	24.35897435897440	49.50495049504950
93		76.53631284916200	65.57377049180330	0.731048805815161	0.8	0.555555555555556	79.3296089385475	51.94805194805190	0.674887169568021	0.666666666666667	0.425531914893617	57.44680851063830	92.42424242424240	7.575757575757580	42.5531914893617	73.74301675977650	71.8562874251497	0.748302740759366	0.857142857142857	0.618556701030928	38.14432989690720	87.8048780487805	12.19512195121950	61.85567010309280
94		72.90502793296090	64.98194945848380	0.714002267573696	0.782608695652174	0.555555555555556	75.97765363128490	57.42574257425740	0.691094748541261	0.725	0.475409836065574	52.45901639344260	90.67796610169490	9.322033898305090	47.54098360655740	69.83240223463690	69.31818181818180	0.712236608276212	0.813333333333333	0.603960396039604	39.60396039603960	82.05128205128200	17.94871794871800	60.39603960396040
95		69.55307262569830	59.17602996254680	0.684480600750939	0.814432989690722	0.464705882352941	69.83240223463690	42.5531914893617	0.611316365626710	0.645161290322581	0.317460317460317	68.25396825396830	90.51724137931040	9.482758620689660	31.74603174603170	69.27374301675980	68.20809248554910	0.727089823468328	0.893939393939394	0.551401869158879	44.85981308411220	90.27777777777780	9.722222222222220	55.14018691588780
96		74.86033519553070	66.4179104477612	0.731324011085916	0.839622641509434	0.549382716049383	76.53631284916200	56.25	0.687827405569341	0.794117647058824	0.435483870967742	56.45161290322580	94.01709401709400	5.982905982905980	43.54838709677420	73.18435754189940	72.09302325581400	0.746708860759494	0.861111111111111	0.62	38.0	87.34177215189870	12.65822784810130	62.0
97		74.58100558659220	62.24066390041490	0.708102766798419	0.728155339805825	0.543478260869565	77.09497206703910	50.60240963855420	0.662398921832884	0.7	0.396226415094340	60.37735849056600	92.85714285714290	7.142857142857140	39.62264150943400	72.06703910614520	68.35443037974680	0.716583229036295	0.739726027397260	0.635294117647059	36.47058823529410	79.7872340425532	20.21276595744680	63.52941176470590
98		71.7877094972067	62.17228464419480	0.701502388735228	0.805825242718447	0.506097560975610	77.09497206703910	60.19417475728160	0.707759784075574	0.815789473684211	0.476923076923077	52.30769230769230	93.85964912280700	6.140350877192980	47.69230769230770	66.4804469273743	63.41463414634150	0.681376262626263	0.8	0.525252525252525	47.47474747474750	83.75	16.25	52.52525252525250
99		72.62569832402240	63.15789473684210	0.704465278881297	0.756756756756757	0.541935483870968	75.97765363128490	48.19277108433740	0.649203431372549	0.625	0.392156862745098	60.78431372549020	90.625	9.375	39.21568627450980	69.27374301675980	69.94535519125680	0.707692307692308	0.810126582278481	0.615384615384615	38.46153846153850	80.0	20.0	61.53846153846150
	**Mean**	**73.2849162011173**	**62.6096329517131**	**70.71609271339450**	**78.15241270761420**	**52.41662648190570**	**76.68715083798880**	**53.1840902032164**	**67.4825132888657%**	**71.9129439039358%**	**42.5936801146927%**	**57.4063198853073**	**92.37134646303870**	**7.628653536961300**	**42.5936801146927**	**69.88268156424580**	**67.57379757607130**	**71.05004109239080**	**81.1900526367124%**	**58.1030822988922%**	**41.8969177011078**	**83.9969998858894**	**16.0030001141106**	**58.1030822988922**
	**Standard Deviation**	2.03239497842304	3.26907003833559	2.108702667301590	4.400677721414960	4.141329841208770	2.55313738998084	5.80032953394705	3.15198810077143%	7.84950304806081%	6.265196884149 69%	6.2651968841497	2.26849659906108	2.268496599061080	6.26519688414969	2.92300286456929	3.44941966676559	2.787418211307560	4.86236783556119%	4.49818330930216%	4.49818330930216	4.39648105408186	4.39648105408186	4.49818330930216
	**T Test Significance (Female to Male)**						**0.000000000016049**	**0.000000000000000**	**0.000014638467332**	**0.000000000000139**	**0.000000000000000**	**0.000000000000000**	**0.000000000009704**	**0.000000000000000**	**0.000000000000000**									

3.1.4 Balanced training data, with feature selection - Support Vector Classifier

	UB_Accuracy	UB_FScore	UB_ROC_AUC	UB_Precision	UB_Recall	UB_F_Accuracy	UB_F_FScore	UB_F_ROC_AUC	UB_F_Precision	UB_F_Recall	UB_F_FNR	UB_F_TNR	UB_F_FPR	UB_F_TPR	UB_M_Accuracy	UB_M_FScore	UB_M_ROC_AUC	UB_M_Precision	UB_M_Recall	UB_M_FNR	UB_M_TNR	UB_M_FPR	UB_M_TPR
	71.27071823204420	55.17241379310340	0.673265159333271	0.810126582278481	0.418300653594771	73.48066298342540	42.85714285714290	0.622652088589851	0.692307692307692	0.310344827586207	68.96551724137930	93.4959349593496	6.504065040650410	31.03448275862070	69.06077348066300	62.16216216216220	0.701407588739290	0.867924528301887	0.484210526315790	51.57894736842110	91.86046511627910	8.13953488372093	48.42105263157890
	72.92817679558010	62.01550387596900	0.714460784313726	0.909090909090909	0.470588235294118	76.79558011049720	53.33333333333330	0.676741664407699	0.857142857142857	0.387096774193548	61.29032258064520	96.63865546218490	3.361344537815130	38.70967741935480	69.06077348066300	66.66666666666670	0.731861998985287	0.933333333333333	0.518518518518519	48.14814814814810	94.52054794520550	5.47945205479452	51.85185185185190
	72.92817679558010	62.01550387596900	0.704144949118888	0.8	0.506329113924051	77.90055248618790	53.48837209302330	0.677560837577816	0.793103448275862	0.403508771929825	59.64912280701750	95.16129032258070	4.838709677419360	40.35087719298250	67.95580110497240	66.27906976744190	0.694678217821782	0.802816901408451	0.564356435643564	43.56435643564360	82.5	17.5	56.43564356435640
	71.54696132596690	55.02183406113540	0.673903508771930	0.818181818181818	0.414473684210526	76.24309392265190	46.91358024691360	0.647532940846650	0.826086956521739	0.327586206896552	67.24137931034480	96.7479674796748	3.252032520325200	32.75862068965520	66.85082872928180	59.45945945945950	0.676571288823673	0.814814814814815	0.468085106382979	53.19148936170210	88.50574712643680	11.49425287356320	46.80851063829790
	70.44198895027620	54.85232067510550	0.666196741854637	0.764705882352941	0.427631578947368	74.03314917127070	44.70588235294120	0.630376344086021	0.678571428571429	0.333333333333333	66.66666666666670	92.74193548387100	7.258064516129030	33.33333333333330	66.85082872928180	60.52631578947370	0.678151774785802	0.807017543859649	0.484210526315790	51.57894736842110	87.20930232558140	12.79069767441860	48.42105263157890
	70.99447513812150	58.82352941176470	0.681549010441016	0.75	0.483870967741936	75.69060773480660	48.83720930232560	0.651137357830271	0.65625	0.388888888888889	61.11111111111110	91.33858267716540	8.661417322834650	38.88888888888890	66.29834254143650	63.90532544378700	0.679826732673267	0.794117647058824	0.534653465346535	46.53465346534650	82.5	17.5	53.46534653465350
	72.37569060773480	60.31746031746030	0.697896039603961	0.826086956521739	0.475	75.69060773480660	50.00000000000000	0.657752153375938	0.758620689655172	0.372881355932203	62.71186440677970	94.26229508196720	5.737704918032790	37.28813559322030	69.06077348066300	65.85365853658540	0.711076732673267	0.857142857142857	0.534653465346535	46.53465346534650	88.75	11.25	53.46534653465350
	69.88950276243100	55.51020408163270	0.673413077658230	0.829268292682927	0.417177914110429	72.92817679558010	44.9438202247191	0.634882478632479	0.8	0.3125	68.75	95.72649572649570	4.273504273504270	31.25	66.85082872928180	61.53846153846150	0.687546193643755	0.842105263157895	0.484848484848485	51.51515151515150	89.02439024390250	10.97560975609760	48.48484848484850
	74.58563535911600	63.4920634920635	0.715137629076300	0.792079207920792	0.529801324503311	79.00552486187850	54.76190476190480	0.686270125223614	0.71875	0.442307692307692	55.76923076923080	93.02325581395350	6.976744186046510	44.23076923076920	70.1657458563536	67.85714285714290	0.714708056171471	0.826086956521739	0.575757575757576	42.42424242424240	85.36585365853660	14.63414634146340	57.57575757575760
	76.79558011049720	63.79310344827590	0.723745173745174	0.804347826086957	0.528571428571429	81.76795580110500	62.06896551724140	0.730090497737557	0.75	0.529411764705882	47.05882352941180	93.07692307692310	6.923076923076920	52.94117647058820	71.8232044198895	64.82758620689660	0.715131900341964	0.839285714285714	0.528089887640449	47.19101123595510	90.21739130434780	9.782608695652170	52.80898876404490
	69.88950276243100	56.57370517928290	0.677080449161667	0.825581395348837	0.430303030303030	75.69060773480660	53.19148936170210	0.672201138519924	0.78125	0.403225806451613	59.67741935483870	94.11764705882350	5.88235294117647	40.32258064516130	64.08839779005530	58.59872611464970	0.672018919591735	0.851851851851852	0.446601941747573	55.33980582524270	89.74358974358980	10.25641025641030	44.66019417475730
	71.27071823204420	58.4	0.686045792079208	0.811111111111111	0.45625	70.71823204419890	39.08045977011490	0.598985829397055	0.607142857142857	0.288135593220339	71.1864406779661	90.98360655737700	9.01639344262295	28.8135593220339	71.8232044198895	68.71165644171780	0.739727722772277	0.903225806451613	0.554455445544555	44.55445544554460	92.5	7.5	55.44554455445550
	74.30939226519340	64.63878326996200	0.725411851487583	0.876288659793814	0.512048192771084	72.92817679558010	44.9438202247191	0.633306429916599	0.769230769230769	0.317460317460317	68.25396825396830	94.91525423728810	5.084745762711870	31.74603174603170	75.69060773480660	74.71264367816090	0.777072442120986	0.915492957746479	0.631067961165049	36.89320388349520	92.3076923076923	7.692307692307690	63.10679611650490
	69.06077348066300	51.30434782608700	0.658354191774049	0.855072463768116	0.366459627329193	72.37569060773480	34.21052631578950	0.591743762265209	0.722222222222222	0.224137931034483	77.58620689655170	95.9349593495935	4.065040650406500	22.41379310344830	65.74585635359120	59.74025974025970	0.691249688822504	0.901960784313726	0.446601941747573	55.33980582524270	93.58974358974360	6.41025641025641	44.66019417475730
	70.71823204419890	54.70085470085470	0.666896833118860	0.771084337349398	0.423841059602649	73.48066298342540	48.93617021276600	0.646212121212121	0.676470588235294	0.383333333333333	61.66666666666670	90.9090909090909	9.090909090909090	38.33333333333330	67.95580110497240	58.57142857142860	0.680830280830281	0.836734693877551	0.450549450549451	54.94505494505500	91.11111111111110	8.88888888888889	45.05494505494510
	68.23204419889500	51.88284518828450	0.650633578089661	0.775	0.389937106918239	70.71823204419890	43.01075268817210	0.621080368906456	0.740740740740741	0.303030303030303	69.6969696969697	93.91304347826090	6.086956521739130	30.30303030303030	65.74585635359120	57.53424657534250	0.663306451612903	0.792452830188679	0.451612903225806	54.83870967741940	87.5	12.5	45.16129032258060
	72.37569060773480	59.67741935483870	0.700357230845036	0.880952380952381	0.451219512195122	79.55801104972380	55.42168674698800	0.690145780768153	0.92	0.396551724137931	60.3448275862069	98.3739837398374	1.626016260162600	39.6551724137931	65.19337016574590	61.81818181818180	0.687232704402516	0.864406779661017	0.481132075471698	51.88679245283020	89.33333333333330	10.66666666666670	48.11320754716980
	71.8232044198895	57.85123966942150	0.689047029702970	0.853658536585366	0.4375	75.13812154696130	54.54545454545460	0.678458498023716	0.818181818181818	0.409090909090909	59.09090909090910	94.78260869565220	5.217391304347830	40.90909090909090	68.50828729281770	60.13986013986010	0.694240645634630	0.877551020408163	0.457446808510638	54.25531914893620	93.10344827586210	6.896551724137930	45.74468085106380
	69.61325966850830	57.69230769230770	0.676511305952930	0.789473684210526	0.454545454545455	71.27071823204420	45.83333333333330	0.631884057971014	0.733333333333333	0.333333333333333	66.66666666666670	93.04347826086960	6.956521739130440	33.33333333333330	67.95580110497240	64.63414634146340	0.694506035969451	0.815384615384615	0.535353535353535	46.46464646464650	85.36585365853660	14.63414634146340	53.53535353535350
	70.99447513812150	58.82352941176470	0.694546568627451	0.882352941176471	0.441176470588235	70.71823204419890	46.46464646464650	0.637258687258687	0.793103448275862	0.328571428571429	67.14285714285710	94.5945945945946	5.405405405405410	32.85714285714290	71.27071823204420	66.66666666666670	0.735308641975309	0.928571428571429	0.52	48.0	95.06172839506170	4.938271604938270	52.0
	74.03314917127070	61.47540983606560	0.709585210501787	0.862068965517241	0.477707006369427	80.66298342541440	56.79012345679010	0.697214931466900	0.851851851851852	0.425925925925926	57.40740740740740	96.8503937007874	3.149606299212600	42.59259259259260	67.40331491712710	63.80368098159510	0.701145133183968	0.866666666666667	0.504854368932039	49.51456310679610	89.74358974358980	10.25641025641030	50.48543689320390
	69.61325966850830	55.28455284552850	0.667945544554455	0.790697674418605	0.425	74.58563535911600	52.08333333333330	0.663797777175386	0.735294117647059	0.403225806451613	59.67741935483870	92.43697478991600	7.563025210084030	40.32258064516130	64.64088397790060	57.33333333333330	0.665170887632161	0.826923076923077	0.438775510204082	56.12244897959180	89.1566265060241	10.84337349397590	43.87755102040820
	74.30939226519340	62.34817813765180	0.709093487097079	0.785714285714286	0.516778523489933	80.66298342541440	54.54545454545450	0.687099236641221	0.777777777777778	0.42	58.00000000000000	95.41984732824430	4.580152671755730	42.0	67.95580110497240	65.88235294117650	0.691364868194137	0.788732394366197	0.565656565656566	43.43434343434340	81.70731707317070	18.29268292682930	56.56565656565660
	74.58563535911600	58.18181818181820	0.695344033052598	0.853333333333333	0.441379310344828	77.90055248618790	42.85714285714290	0.630916030534351	0.75	0.3	70.0	96.18320610687020	3.816793893129770	30.0	71.27071823204420	65.33333333333330	0.723011015911873	0.890909090909091	0.515789473684211	48.42105263157890	93.02325581395350	6.976744186046510	51.57894736842110
	69.06077348066300	50.44247787610620	0.649007242757243	0.791666666666667	0.370129870129870	72.37569060773480	32.43243243243240	0.585101867572156	0.705882352941177	0.210526315789474	78.94736842105260	95.96774193548390	4.032258064516130	21.05263157894740	65.74585635359120	59.21052631578950	0.672434953362789	0.818181818181818	0.463917525773196	53.60824742268040	88.09523809523810	11.90476190476190	46.39175257731960
	70.99447513812150	56.43153526970950	0.677536119310238	0.809523809523810	0.433121019108280	74.03314917127070	52.52525252525250	0.665517241379310	0.764705882352941	0.4	60.0	93.10344827586210	6.896551724137930	40.0	67.95580110497240	59.15492957746480	0.683317049340498	0.84	0.456521739130435	54.34782608695650	91.01123595505620	8.98876404494382	45.65217391304350
	71.54696132596690	58.63453815261040	0.684128371628372	0.768421052631579	0.474025974025974	77.90055248618790	48.71794871794870	0.655560524746571	0.730769230769231	0.365384615384615	63.46153846153850	94.57364341085270	5.426356589147290	36.53846153846150	65.19337016574590	63.1578947368421	0.669769173492182	0.782608695652174	0.529411764705882	47.05882352941180	81.0126582278481	18.9873417721519	52.94117647058820
	72.65193370165750	58.57740585774060	0.687950972051549	0.777777777777778	0.469798657718121	76.79558011049720	47.5	0.647812971342383	0.655172413793103	0.372549019607843	62.74509803921570	92.3076923076923	7.692307692307690	37.25490196078430	68.50828729281770	64.15094339622640	0.699963117777231	0.836065573770492	0.520408163265306	47.95918367346940	87.95180722891570	12.04819277108430	52.04081632653060
	71.54696132596690	55.02183406113540	0.667285531370038	0.724137931034483	0.443661971830986	75.13812154696130	44.44444444444440	0.630748820754717	0.642857142857143	0.339622641509434	66.0377358490566	92.1875	7.8125	33.9622641509434	67.95580110497240	60.81081081081080	0.676722032242306	0.762711864406780	0.505617977528090	49.43820224719100	84.78260869565220	15.21739130434780	50.56179775280900
	71.8232044198895	55.26315789473690	0.670051409777437	0.741176470588235	0.440559440559441	77.90055248618790	52.38095238095240	0.673378582202112	0.666666666666667	0.431372549019608	56.86274509803920	91.53846153846150	8.461538461538460	43.13725490196080	65.74585635359120	56.94444444444440	0.661028334147533	0.788461538461538	0.445652173913043	55.43478260869570	87.64044943820230	12.35955056179780	44.56521739130430
	73.74301675977650	61.47540983606560	0.703830962396840	0.789473684210526	0.503355704697987	75.41899441340780	38.88888888888890	0.607061131088591	0.538461538461538	0.304347826086957	69.56521739130430	90.97744360902260	9.022556390977440	30.43478260869570	72.06703910614520	70.93023255813950	0.743484925907000	0.884057971014493	0.592233009708738	40.77669902912620	89.47368421052630	10.52631578947370	59.22330097087380
	70.94972067039110	57.37704918032790	0.687641438270053	0.875	0.426829268292683	70.39106145251400	40.44943820224720	0.612145748987854	0.75	0.276923076923077	72.3076923076923	94.73684210526320	5.263157894736840	27.69230769230770	71.50837988826820	67.0967741935484	0.737626262626263	0.928571428571429	0.525252525252525	47.47474747474750	95.0	5.0	52.52525252525250
	70.39106145251400	57.25806451612900	0.679887757354100	0.816091954022989	0.440993788819876	73.74301675977650	41.9753086419753	0.621758335708179	0.739130434782609	0.293103448275862	70.6896551724138	95.0413223140496	4.958677685950410	29.31034482758620	67.0391061452514	64.67065868263470	0.696346448645887	0.84375	0.524271844660194	47.57281553398060	86.8421052631579	13.15789473684210	52.42718446601940
	71.7877094972067	55.89519650655020	0.680831423478482	0.853333333333333	0.415584415584416	75.97765363128490	45.56962025316460	0.640389082462253	0.782608695652174	0.321428571428571	67.85714285714290	95.9349593495935	4.065040650406500	32.14285714285710	67.59776536312850	61.33333333333330	0.697656840513984	0.884615384615385	0.469387755102041	53.06122448979590	92.5925925925926	7.407407407407410	46.93877551020410
	72.3463687150838	56.76855895196510	0.683830821895895	0.833333333333333	0.430463576158940	77.09497206703910	50.60240963855420	0.662683284457478	0.75	0.381818181818182	61.81818181818180	94.35483870967740	5.64516129032258	38.18181818181820	67.59776536312850	60.27397260273970	0.693022088353414	0.88	0.458333333333333	54.16666666666670	92.7710843373494	7.228915662650600	45.83333333333330
	71.22905027932960	58.96414342629480	0.685917546027823	0.787234042553192	0.471337579617834	70.39106145251400	40.44943820224720	0.602542372881356	0.6	0.305084745762712	69.49152542372880	90.0	10.0	30.50847457627120	72.06703910614520	69.1358024691358	0.736331569664903	0.875	0.571428571428571	42.85714285714290	90.12345679012350	9.876543209876540	57.14285714285710
	72.06703910614520	59.34959349593500	0.695296717171717	0.848837209302326	0.45625	76.53631284916200	48.78048780487810	0.652964959568733	0.689655172413793	0.377358490566038	62.26415094339620	92.85714285714290	7.142857142857140	37.73584905660380	67.59776536312850	64.63414634146340	0.719885773624091	0.929824561403509	0.495327102803738	50.46728971962620	94.44444444444440	5.555555555555560	49.53271028037380
	69.83240223463690	57.14285714285720	0.678275081719889	0.818181818181818	0.439024390243902	73.18435754189940	42.85714285714290	0.621009490940466	0.666666666666667	0.315789473684211	68.42105263157900	92.62295081967210	7.377049180327870	31.57894736842110	66.4804469273743	64.28571428571430	0.703725337487020	0.885245901639344	0.504672897196262	49.53271028037380	90.27777777777780	9.722222222222220	50.46728971962620
	68.43575418994410	52.32067510548520	0.653702531645570	0.784810126582278	0.392405063291139	73.74301675977650	47.19101123595510	0.643713813068652	0.777777777777778	0.338709677419355	66.12903225806450	94.87179487179490	5.128205128205130	33.87096774193550	63.12849162011170	55.4054054054054	0.647276606425703	0.788461538461538	0.427083333333333	57.29166666666670	86.74698795180720	13.25301204819280	42.70833333333330
	72.06703910614520	58.67768595041320	0.691288779034763	0.835294117647059	0.452229299363057	77.09497206703910	56.84210526315790	0.691386554621849	0.771428571428572	0.45	55.00000000000000	93.27731092436970	6.722689075630250	45.0	67.0391061452514	59.86394557823130	0.690218757857682	0.88	0.453608247422680	54.63917525773200	92.6829268292683	7.317073170731710	45.36082474226800
	71.7877094972067	53.02325581395350	0.662287024901704	0.76	0.407142857142857	77.09497206703910	45.33333333333330	0.638915394402036	0.629629629629630	0.354166666666667	64.58333333333330	92.36641221374050	7.633587786259540	35.41666666666670	66.4804469273743	57.14285714285710	0.671414292853573	0.833333333333333	0.434782608695652	56.52173913043480	90.80459770114940	9.195402298850570	43.47826086956520
	75.69832402234640	62.66094420600860	0.716981132075472	0.839080459770115	0.5	79.3296089385475	54.32098765432100	0.683737646001797	0.785714285714286	0.415094339622642	58.49056603773580	95.23809523809520	4.761904761904760	41.50943396226420	72.06703910614520	67.10526315789470	0.72768192048012	0.864406779661017	0.548387096774194	45.16129032258060	90.69767441860470	9.30232558139535	54.83870967741940
	73.46368715083800	59.91561181434600	0.701938662005403	0.865853658536585	0.458064516129032	75.41899441340780	50.00000000000000	0.657274011299435	0.758620689655172	0.372881355932203	62.71186440677970	94.16666666666670	5.833333333333330	37.28813559322030	71.50837988826820	65.77181208053690	0.731111947791165	0.924528301886793	0.510416666666667	48.95833333333330	95.18072289156630	4.819277108433740	51.04166666666670
	70.39106145251400	56.19834710743800	0.674271952340210	0.8	0.433121019108280	71.50837988826820	41.37931034482760	0.612184873949580	0.666666666666667	0.3	70.0	92.43697478991600	7.563025210084030	30.0	69.27374301675980	64.51612903225810	0.708951470958009	0.862068965517241	0.515463917525773	48.45360824742270	90.2439024390244	9.75609756097561	51.54639175257730
	72.3463687150838	60.24096385542170	0.695137454314318	0.797872340425532	0.483870967741936	74.86033519553070	51.61290322580650	0.661723163841808	0.705882352941177	0.406779661016949	59.32203389830510	91.66666666666670	8.333333333333330	40.67796610169490	69.83240223463690	65.38461538461540	0.711408132530121	0.85	0.53125	46.875	89.1566265060241	10.84337349397590	53.125
	71.22905027932960	56.54008438818570	0.673388786794339	0.744444444444445	0.455782312925170	74.86033519553070	47.05882352941180	0.641126589945488	0.606060606060606	0.384615384615385	61.53846153846150	89.76377952755910	10.23622047244090	38.46153846153850	67.59776536312850	61.84210526315790	0.687844611528822	0.824561403508772	0.494736842105263	50.52631578947370	88.09523809523810	11.90476190476190	49.47368421052630
	72.62569832402240	54.20560747663550	0.669235836627141	0.763157894736842	0.420289855072464	76.53631284916200	44.73684210526320	0.635116279069767	0.653846153846154	0.34	66.0	93.02325581395350	6.976744186046510	34.0	68.71508379888270	59.42028985507250	0.683503996003996	0.82	0.465909090909091	53.40909090909090	90.10989010989010	9.89010989010989	46.59090909090910
	75.97765363128490	61.60714285714290	0.713882398753894	0.8625	0.479166666666667	77.6536312849162	52.38095238095240	0.673126246793959	0.846153846153846	0.379310344827586	62.06896551724140	96.69421487603310	3.305785123966940	37.93103448275860	74.30167597765360	67.14285714285710	0.735621405351338	0.870370370370370	0.546511627906977	45.34883720930230	92.47311827956990	7.526881720430110	54.65116279069770
	70.39106145251400	58.26771653543310	0.684372642695499	0.822222222222222	0.451219512195122	70.39106145251400	41.75824175824180	0.613654891304348	0.703703703703704	0.296875	70.3125	93.04347826086960	6.956521739130440	29.6875	70.39106145251400	67.48466257668710	0.724367088607595	0.873015873015873	0.55	45.0	89.87341772151900	10.12658227848100	55.00000000000000
	69.27374301675980	55.28455284552850	0.662704592404259	0.747252747252747	0.438709677419355	68.71508379888270	40.42553191489360	0.595308123249300	0.558823529411765	0.316666666666667	68.33333333333330	87.39495798319330	12.60504201680670	31.66666666666670	69.83240223463690	64.47368421052630	0.710275689223058	0.859649122807018	0.515789473684211	48.42105263157890	90.47619047619050	9.523809523809520	51.57894736842110
	72.62569832402240	59.16666666666670	0.694549499443826	0.835294117647059	0.458064516129032	76.53631284916200	50.0	0.659620362381363	0.777777777777778	0.368421052631579	63.1578947368421	95.08196721311480	4.918032786885250	36.84210526315790	68.71508379888270	64.1025641025641	0.705719324766944	0.862068965517241	0.510204081632653	48.97959183673470	90.12345679012350	9.876543209876540	51.02040816326530
	70.11173184357540	54.46808510638300	0.668995785404189	0.820512820512821	0.407643312101911	73.74301675977650	49.46236559139780	0.650388996943595	0.71875	0.377049180327869	62.29508196721310	92.37288135593220	7.627118644067800	37.70491803278690	66.4804469273743	57.74647887323940	0.683421184738956	0.891304347826087	0.427083333333333	57.29166666666670	93.97590361445780	6.024096385542170	42.70833333333330
	73.74301675977650	58.7719298245614	0.693475797312611	0.807228915662651	0.462068965517241	77.09497206703910	51.76470588235300	0.670728291316527	0.88	0.366666666666667	63.33333333333330	97.47899159663870	2.521008403361340	36.66666666666670	70.39106145251400	62.93706293706290	0.695556946182728	0.775862068965517	0.529411764705882	47.05882352941180	86.17021276595740	13.82978723404260	52.94117647058820
	71.7877094972067	57.02127659574470	0.682368882512355	0.817073170731707	0.437908496732026	73.74301675977650	48.35164835164840	0.646429563767713	0.733333333333333	0.360655737704918	63.93442622950820	93.22033898305080	6.779661016949150	36.0655737704918	69.83240223463690	62.5	0.704335332333833	0.865384615384615	0.489130434782609	51.08695652173910	91.95402298850570	8.045977011494250	48.91304347826090
	70.6703910614525	52.05479452054790	0.651000264970853	0.686746987951807	0.419117647058824	74.86033519553070	45.78313253012050	0.635448213204119	0.612903225806452	0.365384615384615	63.46153846153850	90.5511811023622	9.448818897637790	36.53846153846150	66.4804469273743	55.88235294117650	0.652506265664160	0.730769230769231	0.452380952380952	54.76190476190480	85.26315789473680	14.73684210526320	45.23809523809520
	69.55307262569830	52.81385281385280	0.655256410256410	0.753086419753086	0.406666666666667	76.53631284916200	43.24324324324320	0.629518995098039	0.695652173913044	0.313725490196078	68.62745098039220	94.53125	5.46875	31.37254901960780	62.56983240223460	57.32484076433120	0.646022727272727	0.775862068965517	0.454545454545455	54.54545454545450	83.75	16.25	45.45454545454550
	74.02234636871510	58.29596412556050	0.691836202399583	0.802469135802469	0.457746478873239	79.88826815642460	53.84615384615390	0.684398854961832	0.7	0.4375	56.25	93.12977099236640	6.870229007633590	43.75	68.15642458100560	60.68965517241380	0.692866082603254	0.862745098039216	0.468085106382979	53.19148936170210	91.76470588235290	8.235294117647060	46.80851063829790
	76.53631284916200	63.79310344827590	0.723857537811026	0.831460674157303	0.517482517482518	81.56424581005590	58.22784810126580	0.707770800627944	0.766666666666667	0.469387755102041	53.06122448979590	94.61538461538460	5.384615384615390	46.93877551020410	71.50837988826820	66.66666666666670	0.724217772215269	0.864406779661017	0.542553191489362	45.74468085106380	90.58823529411770	9.411764705882350	54.25531914893620
	74.02234636871510	60.42553191489360	0.705196875498167	0.865853658536585	0.464052287581699	74.86033519553070	49.43820224719100	0.654904140038900	0.785714285714286	0.360655737704918	63.93442622950820	94.91525423728810	5.084745762711870	36.0655737704918	73.18435754189940	67.12328767123290	0.737568715642179	0.907407407407407	0.532608695652174	46.73913043478260	94.25287356321840	5.747126436781610	53.2608695652174
	70.94972067039110	55.55555555555560	0.673393910409692	0.802469135802469	0.424836601307190	74.30167597765360	46.51162790697670	0.638553350589589	0.689655172413793	0.350877192982456	64.91228070175440	92.62295081967210	7.377049180327870	35.08771929824560	67.59776536312850	60.81081081081080	0.692206325301205	0.865384615384615	0.46875	53.125	91.56626506024100	8.433734939759040	46.875
	70.11173184357540	55.96707818930040	0.676713277903754	0.839506172839506	0.419753086419753	72.62569832402240	43.67816091954020	0.627584780810587	0.76	0.306451612903226	69.35483870967740	94.87179487179490	5.128205128205130	30.64516129032260	67.59776536312850	62.82051282051280	0.700696202531646	0.875	0.49	51.0	91.13924050632910	8.860759493670890	49.0
	68.43575418994410	54.25101214574900	0.658869949494950	0.770114942528736	0.41875	66.4804469273743	33.33333333333330	0.569361413043478	0.576923076923077	0.234375	76.5625	90.43478260869570	9.565217391304350	23.4375	70.39106145251400	66.2420382165605	0.716616465863454	0.852459016393443	0.541666666666667	45.83333333333330	89.1566265060241	10.84337349397590	54.16666666666670
	70.94972067039110	55.17241379310340	0.674431908469728	0.831168831168831	0.412903225806452	75.41899441340780	48.83720930232560	0.655181994998611	0.84	0.344262295081967	65.57377049180330	96.61016949152540	3.389830508474580	34.42622950819670	66.4804469273743	58.9041095890411	0.675782227784731	0.826923076923077	0.457446808510638	54.25531914893620	89.41176470588240	10.58823529411760	45.74468085106380
	69.55307262569830	52.81385281385280	0.654458752127420	0.743902439024390	0.409395973154362	72.06703910614520	43.18181818181820	0.615925925925926	0.558823529411765	0.351851851851852	64.81481481481480	88.0	12.0	35.18518518518520	67.0391061452514	58.74125874125870	0.685338345864662	0.875	0.442105263157895	55.78947368421050	92.85714285714290	7.142857142857140	44.21052631578950
	71.50837988826820	55.65217391304350	0.674871794871795	0.8	0.426666666666667	73.18435754189940	41.46341463414630	0.619067796610170	0.739130434782609	0.288135593220339	71.1864406779661	95.0	5.0	28.8135593220339	69.83240223463690	63.51351351351350	0.701423576423576	0.824561403508772	0.516483516483517	48.35164835164840	88.63636363636360	11.36363636363640	51.64835164835170
	74.58100558659220	60.60606060606060	0.705534105534106	0.843373493975904	0.472972972972973	77.6536312849162	55.55555555555560	0.686864406779661	0.806451612903226	0.423728813559322	57.6271186440678	95.0	5.0	42.3728813559322	71.50837988826820	63.82978723404260	0.713920099875156	0.865384615384615	0.505617977528090	49.43820224719100	92.22222222222220	7.777777777777780	50.56179775280900
	71.22905027932960	56.17021276595750	0.680340336533891	0.846153846153846	0.420382165605096	72.62569832402240	39.50617283950620	0.609005414648048	0.695652173913044	0.275862068965517	72.41379310344830	94.21487603305790	5.785123966942150	27.58620689655170	69.83240223463690	64.93506493506490	0.721275252525253	0.909090909090909	0.505050505050505	49.49494949494950	93.75	6.25	50.50505050505050
	68.71508379888270	54.83870967741940	0.667200502433663	0.819277108433735	0.412121212121212	74.30167597765360	46.51162790697670	0.641456582633053	0.769230769230769	0.333333333333333	66.66666666666670	94.9579831932773	5.042016806722690	33.33333333333330	63.12849162011170	59.25925925925930	0.667760617760618	0.842105263157895	0.457142857142857	54.28571428571430	87.83783783783780	12.16216216216220	45.71428571428570
	73.46368715083800	58.14977973568280	0.692768376095822	0.846153846153846	0.442953020134228	70.94972067039110	36.58536585365850	0.592120262182958	0.625	0.258620689655172	74.13793103448280	92.56198347107440	7.43801652892562	25.86206896551720	75.97765363128490	70.34482758620690	0.763174325674326	0.944444444444444	0.560439560439560	43.95604395604400	96.5909090909091	3.409090909090910	56.04395604395600
	67.31843575419000	49.35064935064940	0.641556840807813	0.791666666666667	0.358490566037736	69.83240223463690	40.0	0.612006929637527	0.782608695652174	0.268656716417910	73.13432835820900	95.53571428571430	4.464285714285710	26.86567164179100	64.80446927374300	55.3191489361702	0.654485257371314	0.795918367346939	0.423913043478261	57.60869565217390	88.50574712643680	11.49425287356320	42.39130434782610
	69.55307262569830	55.87044534412960	0.675412152614225	0.841463414634146	0.418181818181818	73.74301675977650	45.97701149425290	0.636158192090396	0.714285714285714	0.338983050847458	66.10169491525420	93.33333333333330	6.666666666666670	33.89830508474580	65.36312849162010	61.25000000000000	0.696885500129232	0.907407407407407	0.462264150943396	53.77358490566040	93.15068493150690	6.849315068493150	46.22641509433960
	66.20111731843580	52.54901960784310	0.658878796391672	0.858974358974359	0.378531073446328	67.59776536312850	32.55813953488370	0.582156183368870	0.736842105263158	0.208955223880597	79.1044776119403	95.53571428571430	4.464285714285710	20.8955223880597	64.80446927374300	62.72189349112430	0.697430830039526	0.898305084745763	0.481818181818182	51.81818181818180	91.30434782608700	8.695652173913040	48.18181818181820
	70.11173184357540	55.96707818930040	0.680153870309311	0.871794871794872	0.412121212121212	76.53631284916200	51.16279069767440	0.663908246225319	0.733333333333333	0.392857142857143	60.71428571428570	93.4959349593496	6.504065040650410	39.28571428571430	63.68715083798880	58.59872611464970	0.696723460026212	0.958333333333333	0.422018348623853	57.79816513761470	97.14285714285710	2.857142857142860	42.20183486238530
	70.11173184357540	55.96707818930040	0.675647129299745	0.829268292682927	0.422360248447205	73.18435754189940	47.82608695652170	0.645788043478261	0.785714285714286	0.34375	65.625	94.78260869565220	5.217391304347830	34.375	67.0391061452514	60.9271523178808	0.688332914256978	0.851851851851852	0.474226804123711	52.57731958762890	90.2439024390244	9.75609756097561	47.42268041237110
	74.30167597765360	58.92857142857140	0.699163449163449	0.868421052631579	0.445945945945946	77.09497206703910	50.60240963855420	0.663718723037101	0.807692307692308	0.368421052631579	63.1578947368421	95.90163934426230	4.098360655737710	36.84210526315790	71.50837988826820	63.82978723404260	0.718843656343656	0.9	0.494505494505495	50.54945054945060	94.31818181818180	5.681818181818180	49.45054945054950
	75.13966480446930	63.37448559670780	0.717719405285636	0.819148936170213	0.516778523489933	75.97765363128490	50.57471264367820	0.659928122192273	0.647058823529412	0.415094339622642	58.49056603773580	90.47619047619050	9.523809523809520	41.50943396226420	74.30167597765360	70.51282051282050	0.756337851405622	0.916666666666667	0.572916666666667	42.70833333333330	93.97590361445780	6.024096385542170	57.29166666666670
	73.74301675977650	59.82905982905980	0.70864898989899	0.945945945945946	0.4375	75.97765363128490	46.91358024691360	0.653225806451613	1.0	0.306451612903226	69.35483870967740	100.0	0.0	30.64516129032260	71.50837988826820	66.66666666666670	0.735512723607962	0.927272727272727	0.520408163265306	47.95918367346940	95.06172839506170	4.938271604938270	52.04081632653060
	71.7877094972067	61.00386100386100	0.703540100250627	0.868131868131868	0.470238095238095	75.41899441340780	50.0	0.656597321174124	0.733333333333333	0.379310344827586	62.06896551724140	93.38842975206610	6.611570247933880	37.93103448275860	68.15642458100560	66.66666666666670	0.730105401844532	0.934426229508197	0.518181818181818	48.18181818181820	94.20289855072460	5.797101449275360	51.81818181818180
	74.30167597765360	57.79816513761470	0.688824854266031	0.768292682926829	0.463235294117647	79.3296089385475	46.37681159420290	0.647597254004577	0.695652173913044	0.347826086956522	65.21739130434780	94.73684210526320	5.263157894736840	34.78260869565220	69.27374301675980	63.08724832214770	0.693695380774033	0.796610169491525	0.522222222222222	47.77777777777780	86.51685393258430	13.48314606741570	52.22222222222220
	68.43575418994410	54.61847389558230	0.668170426065163	0.839506172839506	0.404761904761905	70.94972067039110	44.68085106382980	0.631889242183360	0.807692307692308	0.308823529411765	69.11764705882350	95.4954954954955	4.504504504504510	30.88235294117650	65.92178770949720	60.64516129032260	0.684367088607595	0.854545454545455	0.47	53.0	89.87341772151900	10.12658227848100	47.0
	69.55307262569830	55.87044534412960	0.675412152614225	0.841463414634146	0.418181818181818	73.74301675977650	45.97701149425290	0.636158192090396	0.714285714285714	0.338983050847458	66.10169491525420	93.33333333333330	6.666666666666670	33.89830508474580	65.36312849162010	61.25000000000000	0.696885500129232	0.907407407407407	0.462264150943396	53.77358490566040	93.15068493150690	6.849315068493150	46.22641509433960
	66.20111731843580	52.54901960784310	0.658878796391672	0.858974358974359	0.378531073446328	67.59776536312850	32.55813953488370	0.582156183368870	0.736842105263158	0.208955223880597	79.1044776119403	95.53571428571430	4.464285714285710	20.8955223880597	64.80446927374300	62.72189349112430	0.697430830039526	0.898305084745763	0.481818181818182	51.81818181818180	91.30434782608700	8.695652173913040	48.18181818181820
	70.11173184357540	55.96707818930040	0.680153870309311	0.871794871794872	0.412121212121212	76.53631284916200	51.16279069767440	0.663908246225319	0.733333333333333	0.392857142857143	60.71428571428570	93.4959349593496	6.504065040650410	39.28571428571430	63.68715083798880	58.59872611464970	0.696723460026212	0.958333333333333	0.422018348623853	57.79816513761470	97.14285714285710	2.857142857142860	42.20183486238530
	70.11173184357540	55.96707818930040	0.675647129299745	0.829268292682927	0.422360248447205	73.18435754189940	47.82608695652170	0.645788043478261	0.785714285714286	0.34375	65.625	94.78260869565220	5.217391304347830	34.375	67.0391061452514	60.9271523178808	0.688332914256978	0.851851851851852	0.474226804123711	52.57731958762890	90.2439024390244	9.75609756097561	47.42268041237110
	74.30167597765360	58.92857142857140	0.699163449163449	0.868421052631579	0.445945945945946	77.09497206703910	50.60240963855420	0.663718723037101	0.807692307692308	0.368421052631579	63.1578947368421	95.90163934426230	4.098360655737710	36.84210526315790	71.50837988826820	63.82978723404260	0.718843656343656	0.9	0.494505494505495	50.54945054945060	94.31818181818180	5.681818181818180	49.45054945054950
	75.13966480446930	63.37448559670780	0.717719405285636	0.819148936170213	0.516778523489933	75.97765363128490	50.57471264367820	0.659928122192273	0.647058823529412	0.415094339622642	58.49056603773580	90.47619047619050	9.523809523809520	41.50943396226420	74.30167597765360	70.51282051282050	0.756337851405622	0.916666666666667	0.572916666666667	42.70833333333330	93.97590361445780	6.024096385542170	57.29166666666670
	73.74301675977650	59.82905982905980	0.70864898989899	0.945945945945946	0.4375	75.97765363128490	46.91358024691360	0.653225806451613	1.0	0.306451612903226	69.35483870967740	100.0	0.0	30.64516129032260	71.50837988826820	66.66666666666670	0.735512723607962	0.927272727272727	0.520408163265306	47.95918367346940	95.06172839506170	4.938271604938270	52.04081632653060
	71.7877094972067	61.00386100386100	0.703540100250627	0.868131868131868	0.470238095238095	75.41899441340780	50.0	0.656597321174124	0.733333333333333	0.379310344827586	62.06896551724140	93.38842975206610	6.611570247933880	37.93103448275860	68.15642458100560	66.66666666666670	0.730105401844532	0.934426229508197	0.518181818181818	48.18181818181820	94.20289855072460	5.797101449275360	51.81818181818180
	74.30167597765360	57.79816513761470	0.688824854266031	0.768292682926829	0.463235294117647	79.3296089385475	46.37681159420290	0.647597254004577	0.695652173913044	0.347826086956522	65.21739130434780	94.73684210526320	5.263157894736840	34.78260869565220	69.27374301675980	63.08724832214770	0.693695380774033	0.796610169491525	0.522222222222222	47.77777777777780	86.51685393258430	13.48314606741570	52.22222222222220
	68.43575418994410	54.61847389558230	0.668170426065163	0.839506172839506	0.404761904761905	70.94972067039110	44.68085106382980	0.631889242183360	0.807692307692308	0.308823529411765	69.11764705882350	95.4954954954955	4.504504504504510	30.88235294117650	65.92178770949720	60.64516129032260	0.684367088607595	0.854545454545455	0.47	53.0	89.87341772151900	10.12658227848100	47.0
	69.83240223463690	54.23728813559320	0.668594545052306	0.831168831168831	0.402515723270440	72.62569832402240	39.50617283950620	0.612324929971989	0.761904761904762	0.266666666666667	73.33333333333330	95.7983193277311	4.201680672268910	26.66666666666670	67.0391061452514	61.93548387096770	0.692424242424243	0.857142857142857	0.484848484848485	51.51515151515150	90.0	10.0	48.48484848484850
	74.86033519553070	61.20689655172410	0.707773326440941	0.825581395348837	0.486301369863014	75.41899441340780	46.34146341463420	0.639925925925926	0.678571428571429	0.351851851851852	64.81481481481480	92.80000000000000	7.200000000000000	35.18518518518520	74.30167597765360	69.33333333333330	0.748125937031484	0.896551724137931	0.565217391304348	43.47826086956520	93.10344827586210	6.896551724137930	56.52173913043480
	71.7877094972067	59.76095617529880	0.697730701533819	0.862068965517241	0.457317073170732	77.09497206703910	51.76470588235300	0.669774011299435	0.846153846153846	0.372881355932203	62.71186440677970	96.66666666666670	3.333333333333330	37.28813559322030	66.4804469273743	63.85542168674700	0.698326898326898	0.868852459016393	0.504761904761905	49.52380952380950	89.1891891891892	10.81081081081080	50.47619047619050
	74.30167597765360	62.90322580645160	0.716069334854390	0.857142857142857	0.496815286624204	73.74301675977650	45.97701149425290	0.636158192090396	0.714285714285714	0.338983050847458	66.10169491525420	93.33333333333330	6.666666666666670	33.89830508474580	74.86033519553070	72.04968944099380	0.765054169816075	0.920634920634921	0.591836734693878	40.81632653061220	93.82716049382720	6.172839506172840	59.18367346938780
	70.6703910614525	58.82352941176470	0.685563114134543	0.806451612903226	0.462962962962963	73.74301675977650	49.46236559139790	0.651295836779708	0.741935483870968	0.370967741935484	62.90322580645160	93.16239316239320	6.837606837606840	37.09677419354840	67.59776536312850	64.19753086419750	0.696708860759494	0.838709677419355	0.52	48.0	87.34177215189870	12.65822784810130	52.0
	72.3463687150838	59.25925925925920	0.690725600408789	0.791208791208791	0.473684210526316	73.74301675977650	50.52631578947370	0.653781512605042	0.685714285714286	0.4	60.0	90.75630252100840	9.243697478991600	40.0	70.94972067039110	64.86486486486490	0.714892553723138	0.857142857142857	0.521739130434783	47.82608695652170	90.80459770114940	9.195402298850570	52.17391304347830
	70.39106145251400	57.60000000000000	0.678676400872286	0.791208791208791	0.452830188679245	71.50837988826820	41.37931034482760	0.607868757259001	0.580645161290323	0.321428571428571	67.85714285714290	89.43089430894310	10.56910569105690	32.14285714285710	69.27374301675980	66.25766871165640	0.722662238119571	0.9	0.524271844660194	47.57281553398060	92.10526315789470	7.894736842105260	52.42718446601940
	70.6703910614525	52.91479820627800	0.662101409717093	0.797297297297297	0.395973154362416	73.74301675977650	41.9753086419753	0.624859943977591	0.809523809523810	0.283333333333333	71.66666666666670	96.63865546218490	3.361344537815130	28.33333333333330	67.59776536312850	59.15492957746480	0.674843945068664	0.792452830188679	0.471910112359551	52.80898876404490	87.77777777777780	12.22222222222220	47.19101123595510
	70.11173184357540	56.68016194331980	0.678826490482932	0.833333333333333	0.429447852760736	71.50837988826820	32.0	0.578687572590012	0.631578947368421	0.214285714285714	78.57142857142860	94.3089430894309	5.691056910569110	21.42857142857140	68.71508379888270	67.44186046511630	0.722416926272066	0.892307692307692	0.542056074766355	45.79439252336450	90.27777777777780	9.722222222222220	54.20560747663550
	69.55307262569830	55.87044534412960	0.667506416959787	0.766666666666667	0.439490445859873	72.62569832402240	46.15384615384620	0.633995554320645	0.7	0.344262295081967	65.57377049180330	92.37288135593220	7.627118644067800	34.42622950819670	66.4804469273743	61.53846153846150	0.677710843373494	0.8	0.5	50.0	85.54216867469880	14.45783132530120	50.0
**Mean**	**71.60031173801660**	**57.4380107929112**	**68.433791066603%**	**81.91049045793770**	**44.36965047714750**	**74.69674989968830**	**46.7815128640275**	**64.31818672143600**	**73.3421117441001%**	**34.73549683336980**	**65.26450316663020**	**93.9008766095023**	**6.0991233904977**	**34.7354968333698**	**68.50387357634500**	**63.2403490604121**	**70.2726650274924%**	**86.16171514529130**	**50.1120418546284%**	**49.8879581453716**	**90.4332882003564**	**9.56671179964362**	**50.1120418546284**
**Standard Deviation**	2.19941509369233	3.28312840736777	1.91797452696993%	4.450625352225390	3.655560779392830	3.05787379731354	5.96001723096667	2.971538949090540	8.33259099357494%	5.845873227395820	5.84587322739582	2.23249405499403	2.23249405499403	5.84587322739582	2.92747954840780	3.93155459869301	2.67254533130535%	4.788398569421720	4.35157684348591%	4.35157684348591	3.38844477944837	3.38844477944838	4.35157684348591
**T Test Significance (Female to Male)**						**0.000000000533318**	**0.000000000000000**	**0.00000000000113**	**0.000000000000000**	**0.000000000000000**	**0.000000000000000**	**0.010199882481145**	**0.000000000000003**	**0.000000000000000**									

3.1.4 Balanced training data, with feature selection - Gaussian Naive Bayes Classifier

		UB_Accuracy	UB_FScore	UB_ROC_AUC	UB_Precision	UB_Recall	UB_F_Accuracy	UB_F_FScore	UB_F_ROC_AUC	UB_F_Precision	UB_F_Recall	UB_F_FNR
0		74.02234636871510	64.0926640926641	0.714447963656141	0.768518518518519	0.549668874172186	72.06703910614520	45.65217391304350	0.626833477135462	0.6	0.368421052631579	63.1578947368421
1		71.7877094972067	60.39215686274510	0.692339426250317	0.777777777777778	0.493589743589744	71.50837988826820	37.03703703703700	0.594693701466782	0.625	0.263157894736842	73.68421052631580
2		73.46368715083800	59.22746781115880	0.693848539674335	0.793103448275862	0.472602739726027	73.18435754189940	38.46153846153850	0.600923682616596	0.576923076923077	0.288461538461538	71.15384615384620
3		70.94972067039110	58.73015873015870	0.688532326569136	0.831460674157303	0.453987730061350	70.39106145251400	39.08045977011490	0.606725543478261	0.739130434782609	0.265625	73.4375
4		72.06703910614520	59.34959349593500	0.691895151053567	0.811111111111111	0.467948717948718	73.18435754189940	35.13513513513510	0.592370370370370	0.65	0.240740740740741	75.92592592592590
5		73.46368715083800	58.87445887445890	0.697831978319783	0.871794871794872	0.444444444444444	78.77094972067040	42.42424242424240	0.629996776273372	0.736842105263158	0.297872340425532	70.2127659574468
6		69.55307262569830	58.55513307984790	0.686202800700175	0.846153846153846	0.447674418604651	71.50837988826820	42.69662921348310	0.622350543478261	0.76	0.296875	70.3125
7		76.25698324022350	63.20346320346320	0.724102564102564	0.901234567901235	0.486666666666667	79.88826815642460	53.84615384615390	0.683434959349593	0.954545454545455	0.375	62.5
8		71.7877094972067	53.88127853881280	0.675737286784633	0.893939393939394	0.385620915032680	70.39106145251400	37.64705882352940	0.610474413646056	0.888888888888889	0.238805970149254	76.11940298507460
9		72.06703910614520	61.24031007751940	0.698895202020202	0.806122448979592	0.49375	75.41899441340780	47.61904761904760	0.646750071901064	0.740740740740741	0.350877192982456	64.91228070175440
10		72.90502793296090	61.6600790513834	0.713721804511278	0.917647058823529	0.464285714285714	78.2122905027933	48.0	0.654666666666667	0.857142857142857	0.333333333333333	66.66666666666670
11		72.06703910614520	54.54545454545460	0.674131274131274	0.833333333333333	0.405405405405405	77.09497206703910	40.57971014492750	0.624138963761605	0.875	0.264150943396226	73.58490566037740
12		72.3463687150838	61.17647058823530	0.698656399531007	0.795918367346939	0.496815286624204	73.74301675977650	37.33333333333330	0.598192401960784	0.583333333333333	0.274509803921569	72.54901960784310
13		70.6703910614525	55.31914893617020	0.676764324768497	0.855263157894737	0.408805031446541	73.74301675977650	38.96103896103900	0.609538327526132	0.714285714285714	0.267857142857143	73.21428571428570
14		73.46368715083800	63.03501945525290	0.709065444359562	0.786407766990291	0.525974025974026	75.41899441340780	52.17391304347830	0.665574237674551	0.705882352941177	0.413793103448276	58.62068965517240
15		72.62569832402240	61.71875	0.704732477850995	0.831578947368421	0.490683229813665	75.41899441340780	52.17391304347830	0.667907361455749	0.8	0.387096774193548	61.29032258064520
16		70.11173184357540	54.07725321888410	0.671622474747475	0.863013698630137	0.39375	72.62569832402240	37.9746835443038	0.612420733388475	0.882352941176471	0.241935483870968	75.80645161290320
17		74.02234636871510	62.04081632653060	0.709341622827993	0.826086956521739	0.496732026143791	76.53631284916200	47.5	0.648533724340176	0.76	0.345454545454545	65.45454545454550
18		73.74301675977650	62.4	0.710396039603961	0.829787234042553	0.5	77.6536312849162	51.21951219512200	0.666715542521994	0.777777777777778	0.381818181818182	61.81818181818180
19		75.97765363128490	64.16666666666670	0.724420849420849	0.836956521739131	0.520270270270270	77.09497206703910	46.75324675324680	0.645220588235294	0.692307692307692	0.352941176470588	64.70588235294120
20		73.74301675977650	60.16949152542370	0.701282912627572	0.835294117647059	0.470198675496689	74.30167597765360	47.72727272727270	0.647987317342156	0.807692307692308	0.338709677419355	66.12903225806450
21		72.06703910614520	62.12121212121210	0.708212015904324	0.863157894736842	0.485207100591716	74.86033519553070	50.54945054945060	0.665392866720300	0.92	0.348484848484849	65.15151515151520
22		71.50837988826820	59.52380952380950	0.690622925950507	0.806451612903226	0.471698113207547	74.86033519553070	45.78313253012050	0.640183615819209	0.791666666666667	0.322033898305085	67.79661016949150
23		75.69832402234640	64.19753086419750	0.724461720574591	0.847826086956522	0.516556291390729	82.12290502793300	61.90476190476190	0.724740740740741	0.866666666666667	0.481481481481481	51.85185185185190
24		74.30167597765360	61.66666666666670	0.708205128205128	0.822222222222222	0.493333333333333	81.00558659217880	59.52380952380950	0.712039532794250	0.806451612903226	0.471698113207547	52.83018867924530
25		72.06703910614520	58.67768595041320	0.686185897435898	0.771739130434783	0.473333333333333	73.18435754189940	40.0	0.610336817653891	0.666666666666667	0.285714285714286	71.42857142857140
26		72.62569832402240	62.87878787878790	0.709234347498114	0.83	0.506097560975610	77.6536312849162	53.48837209302330	0.677164222030486	0.793103448275862	0.403508771929825	59.64912280701750
27		72.3463687150838	59.91902834008100	0.693691112808760	0.795698924731183	0.480519480519481	74.30167597765360	39.47368421052630	0.607996323529412	0.6	0.294117647058824	70.58823529411770
28		75.41899441340780	61.40350877192980	0.714102564102564	0.897435897435898	0.466666666666667	80.44692737430170	54.54545454545460	0.686876832844575	0.954545454545455	0.381818181818182	61.81818181818180
29		69.55307262569830	53.21888412017170	0.654012315826805	0.720930232558140	0.421768707482993	75.41899441340780	43.58974358974360	0.627604166666667	0.629629629629630	0.333333333333333	66.66666666666670
30		70.94972067039110	59.05511811023620	0.686395202020202	0.797872340425532	0.46875	74.30167597765360	39.47368421052630	0.609763402216232	0.652173913043478	0.283018867924528	71.69811320754720
31		73.18435754189940	61.6	0.704052121404736	0.810526315789474	0.496774193548387	72.62569832402240	42.35294117647060	0.619209039548023	0.692307692307692	0.305084745762712	69.49152542372880
32		73.74301675977650	60.50420168067230	0.705164468457016	0.867469879518072	0.464516129032258	77.09497206703910	46.75324675324680	0.646666666666667	0.782608695652174	0.333333333333333	66.66666666666670
33		72.62569832402240	55.45454545454550	0.681698717948718	0.871428571428571	0.406666666666667	74.30167597765360	41.02564102564100	0.621402108862924	0.8	0.275862068965517	72.41379310344830
34		74.86033519553070	65.11627906976740	0.724169712378834	0.815533980582524	0.541935483870968	74.86033519553070	41.55844155844160	0.621260997067449	0.727272727272727	0.290909090909091	70.9090909090909
35		72.3463687150838	59.59183673469390	0.698789292808273	0.869047619047619	0.453416149068323	72.06703910614520	45.65217391304350	0.640898251192369	0.875	0.308823529411765	69.11764705882350
36		75.13966480446930	59.3607305936073	0.698139843879754	0.773809523809524	0.481481481481481	81.00558659217880	52.77777777777780	0.680205949656751	0.730769230769231	0.413043478260870	58.69565217391310
37		70.6703910614525	61.53846153846150	0.700618440779610	0.848484848484849	0.482758620689655	71.50837988826820	48.48484848484850	0.648492791612058	0.827586206896552	0.342857142857143	65.71428571428570
38		72.90502793296090	61.6600790513834	0.701243424198948	0.78	0.509803921568627	73.74301675977650	43.37349397590360	0.626246793958393	0.72	0.310344827586207	68.96551724137930
39		73.46368715083800	64.41947565543070	0.716962711010330	0.819047619047619	0.530864197530864	72.62569832402240	44.9438202247191	0.630036121144762	0.714285714285714	0.327868852459016	67.21311475409840
40		73.46368715083800	61.22448979591840	0.703634624581540	0.815217391304348	0.490196078431373	73.74301675977650	38.96103896103900	0.608137829912024	0.681818181818182	0.272727272727273	72.72727272727270
41		73.46368715083800	60.90534979423870	0.703495034377387	0.831460674157303	0.480519480519481	76.53631284916200	47.5	0.647499251272836	0.703703703703704	0.358490566037736	64.15094339622640
42		73.46368715083800	61.53846153846150	0.703883495145631	0.8	0.5	74.86033519553070	44.44444444444440	0.630128781072177	0.642857142857143	0.339622641509434	66.0377358490566
43		73.46368715083800	60.25104602510460	0.701903488668195	0.847058823529412	0.467532467532468	74.86033519553070	48.27586206896550	0.650944706863017	0.807692307692308	0.344262295081967	65.57377049180330
44		73.74301675977650	62.09677419354840	0.707731547903714	0.810526315789474	0.503267973856209	78.2122905027933	54.11764705882350	0.680962962962963	0.741935483870968	0.425925925925926	57.40740740740740
45		71.50837988826820	56.41025641025640	0.682827898722946	0.857142857142857	0.420382165605096	73.18435754189940	36.8421052631579	0.604160729552579	0.777777777777778	0.241379310344828	75.86206896551720
46		72.06703910614520	59.34959349593500	0.688581757686278	0.768421052631579	0.483443708609272	73.18435754189940	47.82608695652170	0.64219227563212	0.709677419354839	0.360655737704918	63.93442622950820
47		72.06703910614520	60.0	0.696496212121212	0.833333333333333	0.46875	76.53631284916200	52.27272727272730	0.669915254237288	0.793103448275862	0.389830508474576	61.01694915254240
48		72.90502793296090	61.6600790513834	0.704335443037975	0.821052631578947	0.493670886075949	76.53631284916200	56.25	0.689198369565217	0.84375	0.421875	57.8125
49		72.62569832402240	56.25	0.679030373831776	0.7875	0.4375	75.41899441340780	38.88888888888890	0.608241758241758	0.608695652173913	0.285714285714286	71.42857142857140
50		74.86033519553070	64.28571428571430	0.728909982398793	0.920454545454545	0.493902439024390	75.41899441340780	43.5897435897436	0.635734463276836	0.894736842105263	0.288135593220339	71.1864406779661
51		81.00558659217880	71.42857142857140	0.773768670131059	0.876288659793814	0.602836879432624	84.35754189944130	60.00000000000000	0.720117845117845	0.807692307692308	0.477272727272727	52.27272727272730
52		71.22905027932960	55.02183406113540	0.679367088607595	0.887323943661972	0.398734177215190	74.30167597765360	42.5	0.632823269920044	0.944444444444444	0.274193548387097	72.58064516129030
53		72.3463687150838	55.20361990950230	0.679294871794872	0.859154929577465	0.406666666666667	70.94972067039110	27.77777777777780	0.572245762711864	0.769230769230769	0.169491525423729	83.05084745762710
54		72.90502793296090	61.04417670682730	0.702237221535634	0.826086956521739	0.484076433121019	74.86033519553070	44.44444444444440	0.634511256768310	0.782608695652174	0.310344827586207	68.96551724137930
55		70.6703910614525	60.07604562737640	0.688485134497404	0.79	0.484662576687117	71.50837988826820	50.4854368932039	0.652042160737813	0.764705882352941	0.376811594202899	62.31884057971010
56		71.50837988826820	60.76923076923080	0.690317035905271	0.745283018867925	0.512987012987013	74.30167597765360	45.23809523809520	0.632404692082111	0.655172413793103	0.345454545454545	65.45454545454550
57		72.62569832402240	60.48387096774190	0.697600508501510	0.806451612903226	0.483870967741936	74.30167597765360	47.72727272727270	0.647987317342156	0.807692307692308	0.338709677419355	66.12903225806450
58		71.50837988826820	61.36363636363640	0.695715231579279	0.786407766990291	0.503105590062112	70.94972067039110	42.22222222222220	0.616310892172961	0.703703703703704	0.301587301587302	69.84126984126980
59		71.22905027932960	56.54008438818570	0.684122474747475	0.870129870129870	0.41875	73.18435754189940	33.33333333333330	0.595183813052152	0.857142857142857	0.206896551724138	79.3103448275862
60		74.30167597765360	61.98347107438020	0.708140283140283	0.797872340425532	0.506756756756757	75.41899441340780	52.17391304347830	0.666386554621849	0.75	0.4	60.0
61		73.46368715083800	59.57446808510640	0.692660550458716	0.736842105263158	0.5	73.18435754189940	40.0	0.607292602575622	0.592592592592593	0.301886792452830	69.81132075471700
62		72.3463687150838	60.86956521739130	0.697289921299822	0.793814432989691	0.493589743589744	72.62569832402240	42.35294117647060	0.616911130284728	0.642857142857143	0.315789473684211	68.42105263157900
63		74.58100558659220	62.85714285714290	0.714454522227900	0.827956989247312	0.506578947368421	73.18435754189940	46.66666666666670	0.635030198446937	0.636363636363636	0.368421052631579	63.1578947368421
64		76.25698324022350	65.02057613168720	0.730742207460399	0.868131868131868	0.519736842105263	80.44692737430170	52.05479452054790	0.674818291944276	0.904761904761905	0.365384615384615	63.46153846153850
65		73.18435754189940	60.65573770491800	0.698082913201246	0.778947368421053	0.496644295302013	73.74301675977650	40.50632911392410	0.611260856543875	0.615384615384615	0.301886792452830	69.81132075471700
66		75.13966480446930	65.36964980544750	0.727151561309977	0.831683168316832	0.538461538461538	76.53631284916200	47.5	0.647259236826166	0.678571428571429	0.365384615384615	63.46153846153850
67		74.30167597765360	61.01694915254240	0.7046135989941	0.808988764044944	0.489795918367347	77.09497206703910	43.83561643835620	0.635070380353399	0.8	0.301886792452830	69.81132075471700
68		72.62569832402240	61.71875	0.706433852446122	0.849462365591398	0.484662576687117	74.30167597765360	48.88888888888890	0.650666851903306	0.758620689655172	0.360655737704918	63.93442622950820
69		73.18435754189940	58.97435897435900	0.697950103289369	0.873417721518987	0.445161290322581	78.77094972067040	52.5	0.675304878048780	0.875	0.375	62.5
70		74.58100558659220	62.24066390041490	0.714094728800611	0.862068965517241	0.487012987012987	78.2122905027933	56.17977528089890	0.693065894678798	0.925925925925926	0.403225806451613	59.67741935483870
71		75.69832402234640	67.41573033707870	0.735835243188184	0.796460176991151	0.584415584415584	77.6536312849162	47.36842105263160	0.649822695035461	0.620689655172414	0.382978723404255	61.70212765957450
72		72.3463687150838	56.38766519823790	0.677829698857736	0.771084337349398	0.444444444444444	75.41899441340780	38.88888888888890	0.609911151960784	0.666666666666667	0.274509803921569	72.54901960784310
73		72.90502793296090	59.75103734439830	0.7078191238813	0.947368421052632	0.436363636363636	78.77094972067040	54.76190476190480	0.687464985994398	0.958333333333333	0.383333333333333	61.66666666666670
74		71.7877094972067	58.77551020408160	0.684711538461538	0.757894736842105	0.48	73.18435754189940	40.0	0.610336817653891	0.666666666666667	0.285714285714286	71.42857142857140
75		73.74301675977650	59.13043478260870	0.697087440994188	0.839506172839506	0.456375838926175	75.41899441340780	46.34146341463420	0.644350282485876	0.826086956521739	0.322033898305085	67.79661016949150
76		74.30167597765360	60.68376068376070	0.707635899888411	0.876543209876543	0.464052287581699	77.09497206703910	51.76470588235290	0.668994015389000	0.814814814814815	0.379310344827586	62.06896551724140
77		70.94972067039110	61.48148148148150	0.691774127439544	0.761467889908257	0.515527950310559	67.59776536312850	40.81632653061220	0.598417806382408	0.625	0.303030303030303	69.6969696969697
78		73.74301675977650	59.13043478260870	0.698595514604729	0.860759493670886	0.450331125827815	78.2122905027933	48.0	0.653938304881701	0.818181818181818	0.339622641509434	66.0377358490566
79		71.7877094972067	59.10931174089070	0.692771464646465	0.839080459770115	0.45625	74.30167597765360	34.28571428571430	0.588992248062016	0.6	0.24	76.0
80		72.3463687150838	54.79452054794520	0.677418836903118	0.857142857142857	0.402684563758389	77.09497206703910	43.83561643835620	0.636148148148148	0.842105263157895	0.296296296296296	70.37037037037040
81		71.50837988826820	58.19672131147540	0.696868044016679	0.922077922077922	0.425149700598802	77.09497206703910	48.10126582278480	0.655528640638359	0.904761904761905	0.327586206896552	67.24137931034480
82		72.62569832402240	56.63716814159290	0.681442233135177	0.8	0.438356164383562	74.30167597765360	41.02564102564100	0.618466898954704	0.727272727272727	0.285714285714286	71.42857142857140
83		74.02234636871510	61.41078838174270	0.707683723896063	0.840909090909091	0.483660130718954	81.00558659217880	58.53658536585370	0.706573824498353	0.827586206896552	0.452830188679245	54.71698113207550
84		72.06703910614520	59.67741935483870	0.695896464646465	0.840909090909091	0.4625	76.53631284916200	51.16279069767440	0.665266106442258	0.846153846153846	0.366666666666667	63.33333333333330
85		75.69832402234640	64.77732793522270	0.730664556962025	0.898876404494382	0.506329113924051	74.30167597765360	47.72727272727270	0.6527665317139	0.913043478260870	0.323076923076923	67.6923076923077
86		74.02234636871510	55.50239234449760	0.681235833251207	0.828571428571429	0.417266187050360	77.6536312849162	41.17647058823530	0.623626373626374	0.736842105263158	0.285714285714286	71.42857142857140
87		76.25698324022350	67.18146718146720	0.741571969696970	0.878787878787879	0.54375	77.6536312849162	53.48837209302330	0.677614705044172	0.821428571428571	0.396551724137931	60.3448275862069
88		72.06703910614520	57.98319327731090	0.681172118380062	0.734042553191489	0.479166666666667	74.86033519553070	41.55844155844160	0.617800245098039	0.615384615384615	0.313725490196078	68.62745098039220
89		72.3463687150838	56.76855895196510	0.683830821895895	0.833333333333333	0.430463576158940	77.09497206703910	48.10126582278480	0.652565982404692	0.791666666666667	0.345454545454545	65.45454545454550
90		73.46368715083800	58.51528384279480	0.694487179487180	0.848101265822785	0.446666666666667	74.86033519553070	43.03797468354430	0.631567796610170	0.85	0.288135593220339	71.1864406779661
91		74.30167597765360	61.34453781512600	0.704775781123523	0.784946236559140	0.503448275862069	77.09497206703910	46.75324675324680	0.645220588235294	0.692307692307692	0.352941176470588	64.70588235294120
92		74.58100558659220	60.60606060606060	0.709245974812689	0.897435897435898	0.457516339869281	79.88826815642460	52.63157894736840	0.676774483378257	0.869565217391304	0.377358490566038	62.26415094339620
93		74.86033519553070	60.17699115044250	0.708257350353521	0.906666666666667	0.450331125827815	79.3296089385475	49.31506849315070	0.661874812818209	0.9	0.339622641509434	66.0377358490566
94		70.11173184357540	55.9670781893004	0.681381777108434	0.883116883116883	0.409638554216867	71.50837988826820	42.69662921348320	0.624224021592443	0.791666666666667	0.292307692307692	70.76923076923080
95		70.94972067039110	57.72357723577240	0.687022180273714	0.855421686746988	0.435582822085890	72.62569832402240	42.35294117647060	0.627581521739131	0.857142857142857	0.28125	71.875
96		73.18435754189940	58.62068965517240	0.691634491634492	0.809523809523810	0.459459459459460	72.06703910614520	32.43243243243240	0.580865102639296	0.631578947368421	0.218181818181818	78.18181818181820
97		71.7877094972067	61.00386100386100	0.702495532495219	0.858695652173913	0.473053892215569	74.30167597765360	52.08333333333330	0.665991902834008	0.806451612903226	0.384615384615385	61.53846153846150
98		69.55307262569830	53.21888412017170	0.667165873191033	0.861111111111111	0.385093167701863	75.97765363128490	49.41176470588240	0.658193277310924	0.84	0.35	65.0
99		76.25698324022350	64.43514644351460	0.726801801801802	0.846153846153846	0.520270270270270	76.53631284916200	50.0	0.659044715447155	0.75	0.375	62.5
	**Mean**	**73.04**	**60.22**	**69.99%**	**83.13%**	**47.44%**	**75.36**	**45.78**	**64.09%**	**76.37%**	**33.05%**	**66.95**
	**Standard Deviation**	1.77	3.19	1.75%	4.49%	4.10%	2.89	6.45	3.10%	9.99%	5.84%	5.84
	**T Test Significance (Female to Male)**						**0.00**	**0.00**	**0.00%**	**0.00%**	**0.00%**	**0.00**

		UB_F_TNR	UB_F_FPR	UB_F_TPR	UB_M_Accuracy	UB_M_FScore	UB_M_ROC_AUC	UB_M_Precision	UB_M_Recall	UB_M_FNR	UB_M_TNR	UB_M_FPR	UB_M_TPR
0		88.52459016393440	11.47540983606560	36.84210526315790	75.97765363128490	74.25149700598800	0.765081351689612	0.849315068493151	0.659574468085106	34.04255319148940	87.05882352941180	12.94117647058820	65.95744680851060
1		92.62295081967210	7.377049180327870	26.31578947368420	72.06703910614520	71.26436781609200	0.731881313131313	0.826666666666667	0.626262626262626	37.37373737373740	83.75	16.25	62.62626262626260
2		91.33858267716540	8.661417322834650	28.84615384615380	73.74301675977650	69.67741935483870	0.746057571964956	0.885245901639344	0.574468085106383	42.5531914893617	91.76470588235290	8.235294117647060	57.44680851063830
3		94.78260869565220	5.217391304347830	26.5625	71.50837988826820	69.0909090909091	0.731628787878788	0.863636363636364	0.575757575757576	42.42424242424240	88.75	11.25	57.57575757575760
4		94.40000000000000	5.600000000000000	24.07407407407410	70.94972067039110	69.76744186046510	0.729182582123759	0.857142857142857	0.588235294117647	41.17647058823530	87.01298701298700	12.98701298701300	58.82352941176470
5		96.21212121212120	3.787878787878790	29.78723404255320	68.15642458100560	65.45454545454550	0.720470405789610	0.915254237288136	0.509433962264151	49.05660377358490	93.15068493150690	6.849315068493150	50.94339622641510
6		94.78260869565220	5.217391304347830	29.6875	67.59776536312850	66.66666666666670	0.712180490349505	0.878787878787879	0.537037037037037	46.2962962962963	88.73239436619720	11.26760563380280	53.70370370370370
7		99.1869918699187	0.813008130081301	37.5	72.62569832402240	67.97385620915030	0.735419274092616	0.881355932203390	0.553191489361702	44.68085106382980	91.76470588235290	8.235294117647060	55.31914893617020
8		98.21428571428570	1.785714285714290	23.88059701492540	73.18435754189940	64.17910447761190	0.723118279569893	0.895833333333333	0.5	50.0	94.6236559139785	5.376344086021510	50.0
9		94.26229508196720	5.737704918032790	35.08771929824560	68.71508379888270	67.81609195402300	0.707460398569239	0.830985915492958	0.572815533980583	42.71844660194170	84.21052631578950	15.78947368421050	57.28155339805830
10		97.6	2.4	33.33333333333330	67.59776536312850	67.41573033707870	0.732388663967611	0.9375	0.526315789473684	47.36842105263160	93.84615384615380	6.153846153846150	52.63157894736840
11		98.4126984126984	1.587301587301590	26.41509433962260	67.0391061452514	60.9271523178808	0.682581453634085	0.821428571428571	0.484210526315790	51.57894736842110	88.09523809523810	11.90476190476190	48.42105263157890
12		92.1875	7.8125	27.45098039215690	70.94972067039110	71.11111111111110	0.733393641767899	0.864864864864865	0.603773584905660	39.62264150943400	86.3013698630137	13.69863013698630	60.37735849056600
13		95.1219512195122	4.878048780487810	26.78571428571430	67.59776536312850	63.29113924050630	0.709823709759836	0.909090909090909	0.485436893203884	51.45631067961170	93.42105263157900	6.578947368421050	48.54368932038840
14		91.73553719008270	8.264462809917360	41.37931034482760	71.50837988826820	69.09090909090910	0.724585843373494	0.826086956521739	0.59375	40.625	85.54216867469880	14.45783132530120	59.375
15		94.87179487179490	5.128205128205130	38.70967741935480	69.83240223463690	67.07317073170730	0.715277777777778	0.846153846153846	0.555555555555556	44.44444444444440	87.5	12.5	55.55555555555560
16		98.29059829059830	1.709401709401710	24.19354838709680	67.59776536312850	62.33766233766230	0.695515243134291	0.857142857142857	0.489795918367347	51.02040816326530	90.12345679012350	9.876543209876540	48.97959183673470
17		95.16129032258070	4.838709677419360	34.54545454545460	71.50837988826820	69.09090909090910	0.729087931468884	0.850746268656716	0.581632653061225	41.83673469387760	87.65432098765430	12.34567901234570	58.16326530612250
18		95.16129032258070	4.838709677419360	38.18181818181820	69.83240223463690	67.85714285714290	0.718075653719218	0.850746268656716	0.564356435643564	43.56435643564360	87.17948717948720	12.82051282051280	56.43564356435640
19		93.75	6.25	35.29411764705880	74.86033519553070	72.39263803680980	0.761440784510938	0.893939393939394	0.608247422680412	39.17525773195880	91.46341463414640	8.536585365853660	60.82474226804120
20		95.72649572649570	4.273504273504270	33.87096774193550	73.18435754189940	67.56756756756760	0.730898876404494	0.847457627118644	0.561797752808989	43.82022471910110	90.0	10.0	56.17977528089890
21		98.23008849557520	1.769911504424780	34.84848484848490	69.27374301675980	68.20809248554910	0.714039345937660	0.842857142857143	0.572815533980583	42.71844660194170	85.52631578947370	14.47368421052630	57.28155339805830
22		95.83333333333330	4.166666666666670	32.20338983050850	68.15642458100560	66.27218934911240	0.697721518987342	0.811594202898551	0.56	44.0	83.54430379746840	16.45569620253160	56.00000000000000
23		96.8	3.2	48.14814814814810	69.27374301675980	65.40880503144650	0.707065627357305	0.838709677419355	0.536082474226804	46.39175257731960	87.8048780487805	12.19512195121950	53.60824742268040
24		95.23809523809520	4.761904761904760	47.16981132075470	67.59776536312850	62.82051282051280	0.691601709831531	0.830508474576271	0.505154639175258	49.48453608247420	87.8048780487805	12.19512195121950	50.51546391752580
25		93.4959349593496	6.504065040650410	28.57142857142860	70.94972067039110	67.90123456790120	0.716082603254068	0.808823529411765	0.585106382978723	41.48936170212770	84.70588235294120	15.29411764705880	58.51063829787230
26		95.08196721311480	4.918032786885250	40.35087719298250	67.59776536312850	67.41573033707870	0.703984942886812	0.845070422535211	0.560747663551402	43.92523364485980	84.72222222222220	15.27777777777780	56.07476635514020
27		92.1875	7.8125	29.41176470588240	70.39106145251400	69.00584795321640	0.727197240674502	0.867647058823529	0.572815533980583	42.71844660194170	88.1578947368421	11.84210526315790	57.28155339805830
28		99.19354838709680	0.806451612903226	38.18181818181820	70.39106145251400	64.90066225165560	0.716228070175439	0.875	0.515789473684211	48.42105263157890	91.66666666666670	8.333333333333330	51.57894736842110
29		92.1875	7.8125	33.33333333333330	63.68715083798880	58.06451612903230	0.650037650602410	0.762711864406780	0.46875	53.125	83.13253012048190	16.86746987951810	46.875
30		93.65079365079360	6.349206349206350	28.30188679245280	67.59776536312850	67.41573033707870	0.703984942886812	0.845070422535211	0.560747663551402	43.92523364485980	84.72222222222220	15.27777777777780	56.07476635514020
31		93.33333333333330	6.666666666666670	30.50847457627120	73.74301675977650	71.51515151515150	0.747050702811245	0.855072463768116	0.614583333333333	38.54166666666670	87.95180722891570	12.04819277108430	61.45833333333330
32		96.0	4.0	33.33333333333330	70.39106145251400	67.08074534161490	0.728865194211729	0.9	0.534653465346535	46.53465346534650	92.3076923076923	7.692307692307690	53.46534653465350
33		96.69421487603310	3.305785123966940	27.58620689655170	70.94972067039110	63.38028169014080	0.715829585207396	0.9	0.489130434782609	51.08695652173910	94.25287356321840	5.747126436781610	48.91304347826090
34		95.16129032258070	4.838709677419360	29.09090909090910	74.86033519553070	75.13812154696130	0.757721518987342	0.839506172839506	0.68	32.0	83.54430379746840	16.45569620253160	68.0
35		97.2972972972973	2.702702702702700	30.88235294117650	72.62569832402240	67.97385620915030	0.733058264566142	0.866666666666667	0.559139784946237	44.08602150537630	90.69767441860470	9.30232558139535	55.91397849462370
36		94.73684210526320	5.263157894736840	41.30434782608700	69.27374301675980	62.58503401360550	0.691760299625468	0.793103448275862	0.516853932584270	48.31460674157300	86.66666666666670	13.33333333333330	51.68539325842700
37		95.41284403669730	4.587155963302750	34.28571428571430	69.83240223463690	68.9655172413793	0.721794871794872	0.857142857142857	0.576923076923077	42.30769230769230	86.66666666666670	13.33333333333330	57.69230769230770
38		94.21487603305790	5.785123966942150	31.03448275862070	72.06703910614520	70.58823529411770	0.726503759398496	0.8	0.631578947368421	36.84210526315790	82.14285714285710	17.85714285714290	63.1578947368421
39		93.22033898305080	6.779661016949150	32.78688524590160	74.30167597765360	74.15730337078650	0.756219852754506	0.857142857142857	0.653465346534654	34.65346534653470	85.8974358974359	14.10256410256410	65.34653465346540
40		94.35483870967740	5.64516129032258	27.27272727272730	73.18435754189940	71.42857142857140	0.744394053917864	0.857142857142857	0.612244897959184	38.77551020408160	87.65432098765430	12.34567901234570	61.22448979591840
41		93.65079365079360	6.349206349206350	35.84905660377360	70.39106145251400	67.48466257668710	0.727405432850977	0.887096774193548	0.544554455445545	45.54455445544560	91.02564102564100	8.974358974358970	54.45544554455450
42		92.06349206349210	7.936507936507940	33.9622641509434	72.06703910614520	69.87951807228910	0.736679292929293	0.865671641791045	0.585858585858586	41.41414141414140	88.75	11.25	58.58585858585860
43		95.76271186440680	4.23728813559322	34.42622950819670	72.06703910614520	67.10526315789470	0.72768192048012	0.864406779661017	0.548387096774194	45.16129032258060	90.69767441860470	9.30232558139535	54.83870967741940
44		93.60000000000000	6.4	42.59259259259260	69.27374301675980	66.25766871165640	0.710227272727273	0.84375	0.545454545454545	45.45454545454550	87.5	12.5	54.54545454545450
45		96.69421487603310	3.305785123966940	24.13793103448280	69.83240223463690	65.82278481012660	0.718876262626263	0.881355932203390	0.525252525252525	47.47474747474750	91.25	8.75	52.52525252525250
46		92.37288135593220	7.627118644067800	36.0655737704918	70.94972067039110	66.23376623376620	0.710299625468165	0.796875	0.566666666666667	43.33333333333330	85.39325842696630	14.60674157303370	56.66666666666670
47		95.0	5.0	38.98305084745760	67.59776536312850	64.19753086419750	0.699733434881950	0.852459016393443	0.514851485148515	48.51485148514850	88.46153846153850	11.53846153846150	51.48514851485150
48		95.65217391304350	4.347826086956520	42.1875	69.27374301675980	64.96815286624200	0.700688360450563	0.809523809523810	0.542553191489362	45.74468085106380	85.88235294117650	14.11764705882350	54.25531914893620
49		93.07692307692310	6.923076923076920	28.57142857142860	69.83240223463690	64.47368421052630	0.710275689223058	0.859649122807018	0.515789473684211	48.42105263157890	90.47619047619050	9.523809523809520	51.57894736842110
50		98.33333333333330	1.666666666666670	28.8135593220339	74.30167597765360	73.5632183908046	0.770978120978121	0.927536231884058	0.609523809523810	39.04761904761910	93.24324324324320	6.756756756756760	60.95238095238100
51		96.29629629629630	3.703703703703700	47.72727272727270	77.6536312849162	76.19047619047620	0.787213980387227	0.901408450704225	0.659793814432990	34.02061855670100	91.46341463414640	8.536585365853660	65.97938144329900
52		99.14529914529920	0.854700854700855	27.41935483870970	68.15642458100560	61.74496644295300	0.697414658634538	0.867924528301887	0.479166666666667	52.08333333333330	91.56626506024100	8.433734939759040	47.91666666666670
53		97.5	2.5	16.94915254237290	73.74301675977650	68.45637583892620	0.740447052947053	0.879310344827586	0.560439560439560	43.95604395604400	92.04545454545460	7.954545454545450	56.04395604395600
54		95.86776859504130	4.132231404958680	31.03448275862070	70.94972067039110	69.04761904761910	0.724179292929293	0.840579710144928	0.585858585858586	41.41414141414140	86.25	13.75000000000000	58.58585858585860
55		92.72727272727270	7.272727272727270	37.68115942028990	69.83240223463690	66.25	0.705444305381727	0.803030303030303	0.563829787234043	43.61702127659580	84.70588235294120	15.29411764705880	56.38297872340430
56		91.93548387096770	8.064516129032260	34.54545454545460	68.71508379888270	68.18181818181820	0.696780303030303	0.779220779220779	0.606060606060606	39.39393939393940	78.75	21.25	60.60606060606060
57		95.72649572649570	4.273504273504270	33.87096774193550	70.94972067039110	67.5	0.714741185296324	0.805970149253731	0.580645161290323	41.93548387096770	84.88372093023260	15.11627906976740	58.06451612903230
58		93.10344827586210	6.896551724137930	30.15873015873020	72.06703910614520	71.26436781609200	0.729906777525825	0.815789473684211	0.632653061224490	36.73469387755100	82.71604938271610	17.28395061728400	63.26530612244900
59		98.34710743801650	1.652892561983470	20.68965517241380	69.27374301675980	66.66666666666670	0.717659791189203	0.873015873015873	0.539215686274510	46.07843137254900	89.6103896103896	10.38961038961040	53.92156862745100
60		93.27731092436970	6.722689075630250	40.0	73.18435754189940	68.0	0.729333166833167	0.822580645161290	0.579545454545455	42.04545454545460	87.91208791208790	12.08791208791210	57.95454545454550
61		91.26984126984130	8.73015873015873	30.18867924528300	73.74301675977650	69.6774193548387	0.734257871064468	0.794117647058824	0.620689655172414	37.93103448275860	84.78260869565220	15.21739130434780	62.06896551724140
62		91.80327868852460	8.19672131147541	31.57894736842110	72.06703910614520	70.23809523809520	0.735479797979798	0.855072463768116	0.595959595959596	40.4040404040404	87.5	12.5	59.59595959595960
63		90.1639344262295	9.836065573770490	36.84210526315790	75.97765363128490	72.25806451612900	0.770927318295739	0.933333333333333	0.589473684210526	41.05263157894740	95.23809523809520	4.761904761904760	58.94736842105260
64		98.4251968503937	1.574803149606300	36.53846153846150	72.06703910614520	70.58823529411760	0.736708860759494	0.857142857142857	0.6	40.0	87.34177215189870	12.65822784810130	60.0
65		92.06349206349210	7.936507936507940	30.18867924528300	72.62569832402240	70.30303030303030	0.735818273092370	0.840579710144928	0.604166666666667	39.58333333333330	86.74698795180720	13.25301204819280	60.41666666666670
66		92.91338582677170	7.086614173228350	36.53846153846150	73.74301675977650	73.4463276836158	0.759166666666667	0.890410958904110	0.625	37.5	89.33333333333330	10.66666666666670	62.5
67		96.82539682539680	3.174603174603170	30.18867924528300	71.50837988826820	68.71165644171780	0.721401752190238	0.811594202898551	0.595744680851064	40.42553191489360	84.70588235294120	15.29411764705880	59.57446808510640
68		94.0677966101695	5.932203389830510	36.0655737704918	70.94972067039110	68.67469879518070	0.733957219251337	0.890625	0.558823529411765	44.11764705882350	90.9090909090909	9.090909090909090	55.88235294117650
69		97.5609756097561	2.439024390243900	37.5	67.59776536312850	62.33766233766230	0.698674242424243	0.872727272727273	0.484848484848485	51.51515151515150	91.25	8.75	48.48484848484850
70		98.29059829059830	1.709401709401710	40.32258064516130	70.94972067039110	65.78947368421050	0.714267866066967	0.833333333333333	0.543478260869565	45.65217391304350	88.50574712643680	11.49425287356320	54.34782608695650
71		91.66666666666670	8.333333333333330	38.29787234042550	73.74301675977650	75.39267015706810	0.753115264797508	0.857142857142857	0.672897196261682	32.71028037383180	83.33333333333330	16.66666666666670	67.28971962616820
72		94.53125	5.46875	27.45098039215690	69.27374301675980	64.51612903225810	0.699049762440610	0.806451612903226	0.537634408602151	46.23655913978490	86.04651162790700	13.95348837209300	53.76344086021510
73		99.15966386554620	0.840336134453782	38.33333333333330	67.0391061452514	62.42038216560510	0.713063063063063	0.942307692307692	0.466666666666667	53.33333333333330	95.94594594594590	4.054054054054050	46.66666666666670
74		93.4959349593496	6.504065040650410	28.57142857142860	70.39106145251400	67.87878787878790	0.709637046307885	0.788732394366197	0.595744680851064	40.42553191489360	82.35294117647060	17.64705882352940	59.57446808510640
75		96.66666666666670	3.333333333333330	32.20338983050850	72.06703910614520	66.21621621621620	0.721660424469413	0.844827586206897	0.544444444444444	45.55555555555560	89.8876404494382	10.11235955056180	54.44444444444440
76		95.86776859504130	4.132231404958680	37.93103448275860	71.50837988826820	65.77181208053690	0.728132832080201	0.907407407407407	0.515789473684211	48.42105263157890	94.04761904761910	5.952380952380950	51.57894736842110
77		89.38053097345130	10.61946902654870	30.30303030303030	74.30167597765360	73.25581395348840	0.748245614035088	0.818181818181818	0.663157894736842	33.68421052631580	83.33333333333330	16.66666666666670	66.3157894736842
78		96.82539682539680	3.174603174603170	33.9622641509434	69.27374301675980	64.51612903225810	0.711892164273117	0.877192982456140	0.510204081632653	48.97959183673470	91.35802469135800	8.641975308641980	51.02040816326530
79		93.7984496124031	6.2015503875969	24.0	69.27374301675980	68.92655367231640	0.733794466403162	0.910447761194030	0.554545454545455	44.54545454545460	91.30434782608700	8.695652173913040	55.45454545454550
80		97.6	2.4	29.62962962962960	67.59776536312850	60.27397260273970	0.689912280701754	0.862745098039216	0.463157894736842	53.68421052631580	91.66666666666670	8.333333333333330	46.31578947368420
81		98.34710743801650	1.652892561983470	32.75862068965520	65.92178770949720	63.03030303030300	0.709960681520315	0.928571428571429	0.477064220183486	52.29357798165140	94.28571428571430	5.714285714285710	47.70642201834860
82		95.1219512195122	4.878048780487810	28.57142857142860	70.94972067039110	64.86486486486490	0.710486891385768	0.827586206896552	0.533333333333333	46.66666666666670	88.76404494382020	11.23595505617980	53.33333333333330
83		96.03174603174600	3.968253968253970	45.28301886792450	67.0391061452514	62.89308176100630	0.693037974683544	0.847457627118644	0.5	50.0	88.60759493670890	11.39240506329110	50.0
84		96.63865546218490	3.361344537815130	36.66666666666670	67.59776536312850	64.19753086419750	0.696708860759494	0.838709677419355	0.52	48.0	87.34177215189870	12.65822784810130	52.0
85		98.24561403508770	1.754385964912280	32.30769230769230	77.09497206703910	74.21383647798740	0.776506626656664	0.893939393939394	0.634408602150538	36.55913978494620	91.86046511627910	8.13953488372093	63.44086021505380
86		96.15384615384620	3.846153846153850	28.57142857142860	70.39106145251400	62.41134751773050	0.705118601747815	0.862745098039216	0.488888888888889	51.11111111111110	92.13483146067420	7.865168539325840	48.88888888888890
87		95.86776859504130	4.132231404958680	39.6551724137931	74.86033519553070	73.98843930635840	0.768270944741533	0.901408450704225	0.627450980392157	37.25490196078430	90.9090909090909	9.090909090909090	62.74509803921570
88		92.1875	7.8125	31.37254901960780	69.27374301675980	65.83850931677020	0.697736934233558	0.779411764705882	0.569892473118280	43.01075268817200	82.55813953488370	17.44186046511630	56.98924731182800
89		95.96774193548390	4.032258064516130	34.54545454545460	67.59776536312850	61.33333333333330	0.691390562248996	0.851851851851852	0.479166666666667	52.08333333333330	90.36144578313250	9.63855421686747	47.91666666666670
90		97.5	2.5	28.8135593220339	72.06703910614520	66.66666666666670	0.723588911088911	0.847457627118644	0.549450549450550	45.05494505494510	89.77272727272730	10.22727272727270	54.94505494505500
91		93.75	6.25	35.29411764705880	71.50837988826820	68.32298136645960	0.721964956195244	0.820895522388060	0.585106382978723	41.48936170212770	85.88235294117650	14.11764705882350	58.51063829787230
92		97.61904761904760	2.380952380952380	37.73584905660380	69.27374301675980	64.51612903225810	0.718354430379747	0.909090909090909	0.5	50.0	93.67088607594940	6.329113924050630	50.0
93		98.4126984126984	1.587301587301590	33.9622641509434	70.39106145251400	65.359477124183	0.724237843285462	0.909090909090909	0.510204081632653	48.97959183673470	93.82716049382720	6.172839506172840	51.02040816326530
94		95.6140350877193	4.385964912280700	29.23076923076920	68.71508379888270	63.63636363636360	0.716933231784717	0.924528301886793	0.485148514851485	51.48514851485150	94.87179487179490	5.128205128205130	48.51485148514850
95		97.3913043478261	2.608695652173910	28.125	69.27374301675980	65.83850931677020	0.711426767676768	0.854838709677419	0.535353535353535	46.46464646464650	88.75	11.25	53.53535353535350
96		94.35483870967740	5.64516129032258	21.81818181818180	74.30167597765360	70.8860759493671	0.748749687421855	0.861538461538462	0.602150537634409	39.78494623655910	89.53488372093020	10.46511627906980	60.21505376344090
97		94.73684210526320	5.263157894736840	38.46153846153850	69.27374301675980	66.25766871165650	0.719251336898396	0.885245901639344	0.529411764705882	47.05882352941180	90.9090909090909	9.090909090909090	52.94117647058820
98		96.63865546218490	3.361344537815130	35.0	63.12849162011170	55.40540540540540	0.664508758568165	0.872340425531915	0.405940594059406	59.4059405940594	92.3076923076923	7.692307692307690	40.5940594059406
99		94.3089430894309	5.691056910569110	37.5	75.97765363128490	72.25806451612900	0.764117941029485	0.888888888888889	0.608695652173913	39.13043478260870	91.95402298850570	8.045977011494250	60.86956521739130
	**Mean**	**95.12**	**4.88**	**33.05**	**70.73**	**67.41**	**72.25%**	**85.72%**	**55.83%**	**44.17**	**88.68**	**11.32**	**55.83**
	**Standard Deviation**	2.35	2.35	5.84	2.73	3.87	2.33%	3.83%	5.34%	5.34	3.56	3.56	5.34
	**T Test Significance (Female to Male)**	**0.00**	**0.00**	**0.00**									
